# Superfamily Assignments for the Yeast Proteome through Integration of Structure Prediction with the Gene Ontology 

**DOI:** 10.1371/journal.pbio.0050076

**Published:** 2007-03-20

**Authors:** Lars Malmström, Michael Riffle, Charlie E. M Strauss, Dylan Chivian, Trisha N Davis, Richard Bonneau, David Baker

**Affiliations:** 1 Department of Biochemistry, University of Washington, Seattle, Washington, United States of America; 2 Los Alamos National Laboratory, Los Alamos, New Mexico, United States of America; 3 Department of Biology, Department of Computer Science, and Center for Comparative Functional Genomics, New York University, New York, New York, United States of America; 4 Howard Hughes Medical Institute, University of Washington, Seattle, Washington, United States of America; University of California San Francisco, United States of America

## Abstract

Saccharomyces cerevisiae is one of the best-studied model organisms, yet the three-dimensional structure and molecular function of many yeast proteins remain unknown. Yeast proteins were parsed into 14,934 domains, and those lacking sequence similarity to proteins of known structure were folded using the Rosetta de novo structure prediction method on the World Community Grid. This structural data was integrated with process, component, and function annotations from the *Saccharomyces* Genome Database to assign yeast protein domains to SCOP superfamilies using a simple Bayesian approach. We have predicted the structure of 3,338 putative domains and assigned SCOP superfamily annotations to 581 of them. We have also assigned structural annotations to 7,094 predicted domains based on fold recognition and homology modeling methods. The domain predictions and structural information are available in an online database at http://rd.plos.org/10.1371_journal.pbio.0050076_01.

## Author Summary

The three-dimensional structure of a protein can reveal much about that protein's evolutionary relationships and functions. Such information about all the proteins in an organism—the proteome—would offer a more global view of these relationships, but solving each structure individually would be a formidable task. In this study, we have parsed all Saccharomyces cerevisiae proteins into nearly 15,000 distinct domains and then used de novo structure prediction methods together with worldwide distributed computing to predict structures for all domains lacking sequence similarity to proteins of known structure. To overcome the uncertainties in de novo structure prediction, we combined these predictions with data on the biological process, function, and localization of the proteins from previous experimental studies to assign the domains to families of evolutionarily related proteins. Our genome-wide domain predictions and superfamily assignments provide the basis for the generation of experimentally testable hypotheses about the mechanism of action for a large number of yeast proteins.

## Abstract 3

Protein three-dimensional structures were predicted for all*Saccharomyces cerevisiae* domains that were found to have no sequence similarity to any proteins of known structure.

## Introduction

The yeast Saccharomyces cerevisiae is one of the most widely studied organisms, yet a large fraction of its proteins are of unknown structure and/or unknown function. Knowledge of the structure of a protein is critical to understand how it functions, and hence, a complete set of protein structures for yeast is desirable, but difficult to accomplish experimentally.

The accuracy of de novo structure prediction methods, although far from the accuracy of experimental structures, has improved in recent years. The Rosetta de novo structure prediction method [1–4] is currently one of the best methods available for predicting the structure of proteins lacking obvious homology to known structures [5–8]. Application of Rosetta to genome-wide annotation has been limited by the difficulty of distinguishing accurate from inaccurate predictions and the computational cost associated with scaling the procedure to whole genomes. Initial results have been encouraging, showing promise on subsets of protein families and prokaryotic genomes [9,10]. We have previously [11] predicted structures for short Pfam families without structural information, and showed that a simple confidence function could partially separate correct structure predictions from incorrect predictions.

There is a rich body of work on the relationship between superfamily (encoded in databases such as SCOP [12–14] and CATH [15]) and function (encoded in databases such as Kyoto Encyclopedia of Genes and Genomes [KEGG] [16] and Gene Ontology [GO] [17]). Although many superfamilies have been shown to carry out multiple functions, Hegyi and Gerstein [18] found that the majority of structure superfamilies carry out one or a few molecular functions, and conversely, that the majority of functions are carried out by one or a few SCOP superfamilies. This relationship can be exploited when predicting to which structure superfamily a protein belongs [9].

We describe an integrated approach for assigning protein domains to structure superfamilies that combines de novo structure predictions with GO function, process, and component annotations. We first parse all yeast proteins into putative structural domains using the Ginzu method [7,19]. Ginzu predicts domain boundaries by applying a hierarchy of sequence-based methods beginning with searching for homologs of known structure using PSI-BLAST [20] and ending by parsing block patterns in multiple sequence alignments (MSAs). After running Ginzu on the full proteome, we applied the Rosetta structure prediction method to domains shorter than 150 amino acids for which no homolog of known structure was found. The top structure predictions were compared to protein domains of known structure using the MAMMOTH protein structure comparison program [21]. The reliability of an assignment to a protein structure superfamily derived from these structure comparisons was evaluated using a logistic regression–based confidence function optimized on a large training set of Rosetta models for proteins of known structure. Superfamily predictions of increased accuracy were obtained by integrating GO function, component, and process annotations [17,22] from the *Saccharomyces* Genome Database [23] with the structure prediction data using a simple Bayesian approach. We predicted structures for 3,338 domains and have annotated 581 of them with novel SCOP superfamily assignments. The domain predictions, the predicted structures, and superfamily assignments are accessible at http://rd.plos.org/10.1371_journal.pbio.0050076_01.

## Results

### Predicting Structural Domains

A total of 6,238 open reading frames (ORFs) were parsed into structural domains using Ginzu [7,19]. Ginzu was used successfully in Critical Assessment of Techniques for Protein Structure Prediction 6 (CASP6) to delineate domains within query proteins by sequentially searching for (1) sequence-detectable homology to the Protein Data Bank (PDB) using PSI-BLAST [20], (2) more-remote fold recognition hits to PDB structures [24,25], (3) hits to Pfam-conserved sequence family domains [26,27], and (4) block patterns in MSAs. This hierarchical application of methods is organized so that methods providing more reliable information are applied first, thus accuracy is not sacrificed as we apply multiple methods in an attempt to maximize comprehensive coverage of the genome. A total of 14,934 domains were predicted, of which 38% had a sequence-detectable homolog of known structure, and an additional 9% could confidently be annotated by fold recognition methods. A summary of the genome-wide domain parses is presented in Table 1, and a complete list of domain predictions are presented in Table S1.

**Table 1:**
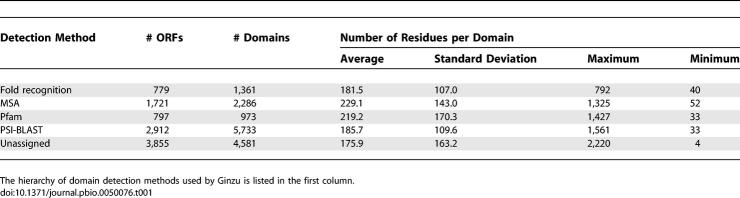
Summary of Domain Assignments for the Yeast Genome Made Using the Ginzu Method

### Fold Recognition

Although the confident fold recognition results generated as part of this study are not the main focus of this paper, they provide a wealth of information on proteins for which there are no detectable sequence homologs of known structure. The results for 1,361 domain annotations using fold recognition are detailed in Table S1 and are available at http://rd.plos.org/10.1371_journal.pbio.0050076_01.

### Protein Structure Prediction

A total of 4,006 yeast protein domains shorter than 150 amino acids (a practical length limit for the Rosetta method) and not linked to known structures by PSI-BLAST or fold recognition methods were identified by Ginzu: 668 of these contained predicted transmembrane helices and were omitted; the remaining 3,338 domains were folded using the Rosetta de novo method. Ten thousand structure models were generated for each of these remaining 3,338 domains using the Rosetta de novo method [1,2,28] and then condensed to 30 representative models by clustering. The size of the calculation is significant and is estimated at 12 million CPU hours, or 1,350 CPU years. This calculation was performed on the World Community Grid (WCG) parallel grid computing facility provided by IBM (http://wcgrid.org).

### Superfamily Assignment by Structure Comparison

**Figure 1:**
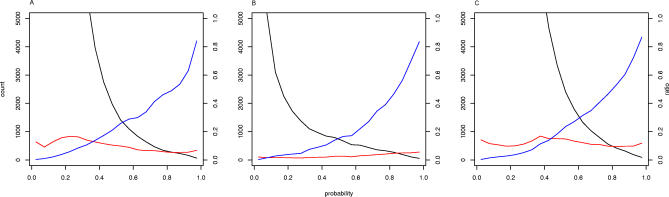
Performance of MAMMOTH Confidence Metric on Benchmark Set (A) Alpha-helical protein model, (B) beta-sheet protein model, and (C) alpha-helical/beta-sheet protein model. The *x*-axis is the *P*_MCM_; the *y*-axis the number of matches that are incorrect (black) and correct (red). The blue line is the ratio (correct/incorrect).

The 30 representative models for each domain were compared to a database of experimentally determined protein structure domains (based on ASTRAL; see Materials and Methods) with representatives from all SCOP (version 1.67) superfamilies and evaluated using a confidence function (referred to as the MAMMOTH Confidence Metric [MCM]) described below. The confidence of a given prediction for a given protein-domain is estimated based on features resulting from the Rosetta structure prediction, clustering, and structure–structure matching steps (using MAMMOTH [21]). The primary improvement in the confidence function over our previous work [11] is the inclusion of the contact order (CO; average sequence separation of contacting amino acids [29]) of the residues superimposed in the MAMMOTH structure–structure alignment of the predicted structure with the matched structure; this CO term penalizes less-significant matches dominated by local contacts such as single long alpha helices. Figure 1 describes the performance of the MCM on a large benchmark set developed for this study (see Materials and Methods). The MCM score, *P*_MCM_, ranges between 0 and 1 and is an estimate of the probability of the identification of the correct structure superfamily identification. A total of 404 domains in the yeast dataset (see Table 2) have a *P*_MCM_ above 0.8 (considered significant for the purpose of this discussion) and can be found in Table S2 (additional domains are annotated with superfamily via integration with GO as described below).

**Table 2:**
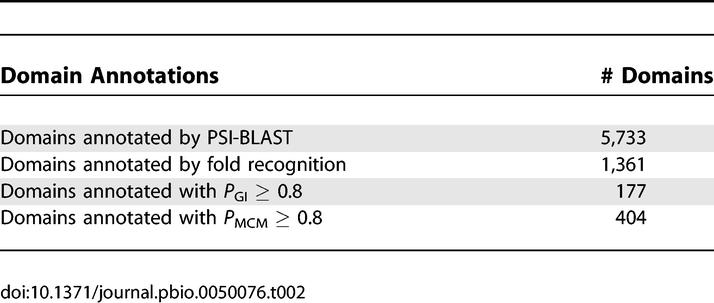
Overview of Domain Annotations Using All Methods Employed in This Work

**Table 3:**
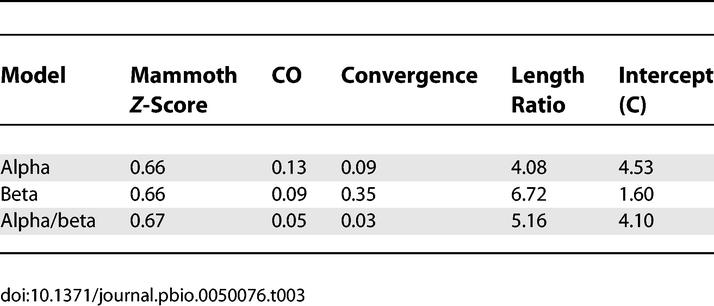
MAMMOTH Confidence Metric (MCM) Logistic Regression Model Parameters

The confidence estimates derived from our SCOP benchmark set are likely to be somewhat inflated when applied to the yeast protein set for two reasons; first, as discussed in the following section, the domain boundaries are derived directly from experimental structures in our SCOP benchmark, but are subject to error for the yeast proteins, and second, in the SCOP benchmark set, there is by construction always at least one closely related structure in the correct superfamily, whereas proteins with novel folds in yeast may not belong to any pre-existing superfamily. Below and in Materials and Methods, we describe tests on two additional validation sets that include the above sources of error (and thus allow for the estimation of the effects of such errors on structure superfamily prediction). Although there is a non-negligible presence of errors in domain parsing and superfamily assignment, our results show that the superfamily assignments generated herein (see Table S2) should be valuable for stimulating the generation of experimentally testable hypotheses about the structure and often the mechanism of action of these proteins.

### Superfamily Assignment through Integration of Structure Predictions with Function

There is a strong relationship between the function of a protein and its structural superfamily [18]. Most commonly, proteins in the same superfamily carry out one or a few functions. The reverse is also true; often only one or a few superfamilies are found to carry out a specific function. We derived probability distributions, *P*(GO|SF), that relate SCOP superfamily (SF) to molecular function, biological process, and cellular component (GO). We also constructed probability distributions, *P*(SF|D), that give the probability of a given superfamily, given the predicted structures (D), that is derived from the distributions of *P*_MCM_ for a target, as described in Materials and Methods. These distributions were integrated to determine the degree to which a superfamily prediction is simultaneously compatible with the structure predictions and the functional annotation available for a given protein, using:

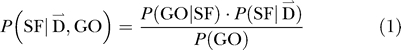
where *P*(SF|D,GO) is the probability that the domain belongs to SCOP superfamily SF, given the predicted structures, D, and the GO terms, GO, for the protein. The independence assumption underlying Equation 1 is described in Materials and Methods.

**Figure 2:**
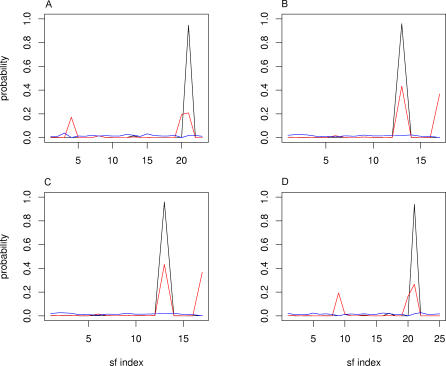
Integration of Structure Prediction with GO Annotations Red line represents the superfamily distribution for the predicted structures, *P*(SF|D); blue line, the superfamily distribution based on GO annotations, *P*(SF|GO). Black line represents the Bayesian combination (*P*(SF|D,GO); Equation 2). Only superfamilies with a probability over 0.001 in either category are displayed. The names of the proteins and the GO annotations for which the black line is derived are (A) 1KMDA (Vam7p Px Domain)/Golgi to vacuole transport (process), (B) 1IOUA (v-SNARE)/vesicle fusion (process), (C) 1F32 (*Ascaris* pepsin inhibitor-3)/endopeptidase inhibitor activity (function), and (D) 1DUJA (Spindle Assembly Checkpoint protein Human Mad2)/Chromosome (component).

**Figure 3:**
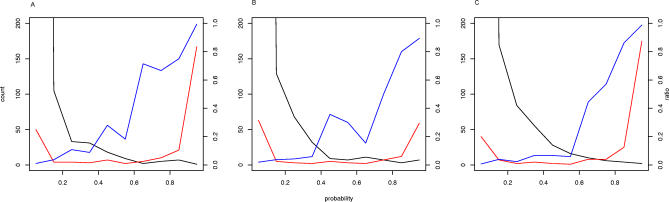
Performance of the GO Integration on Benchmark Set (A) Alpha-helical proteins, (B) beta-sheet proteins, and (C) alpha-helical/beta-sheet proteins. The *x*-axis is *P*(SF|D,GO); the *y*-axis the number of matches with that score that are incorrect (black) and correct (red). The blue line is the ratio (correct/incorrect).

The superfamily distributions derived from the structure prediction data alone (*P*(SF|D)), the GO annotations (*P*(SF|GO)), and from the two together (*P*(SF|D,GO)), are compared in Figure 2 for four proteins for which the true SCOP superfamilies are known, showing the synergy between the two sources of information. The ambiguities in *P*(SF|D) (red line) and *P*(SF|GO) (blue line) are reduced upon integration *P*(SF|D,GO) (black line), resulting in less ambiguous predictions for many difficult-to-annotate domains. The overall performance for the *P*(SF|D,GO) over the benchmark set (see Materials and Methods) is shown in Figure 3. A total of 177 yeast domains (see Table 2) were assigned a structural superfamily with a *P*(SF|D,GO) over 0.8 (Table S3).

### Internal Standards—Additional Validation of Confidence Metric Using Proteins Solved after Calculations Were Completed

**Figure 4:**
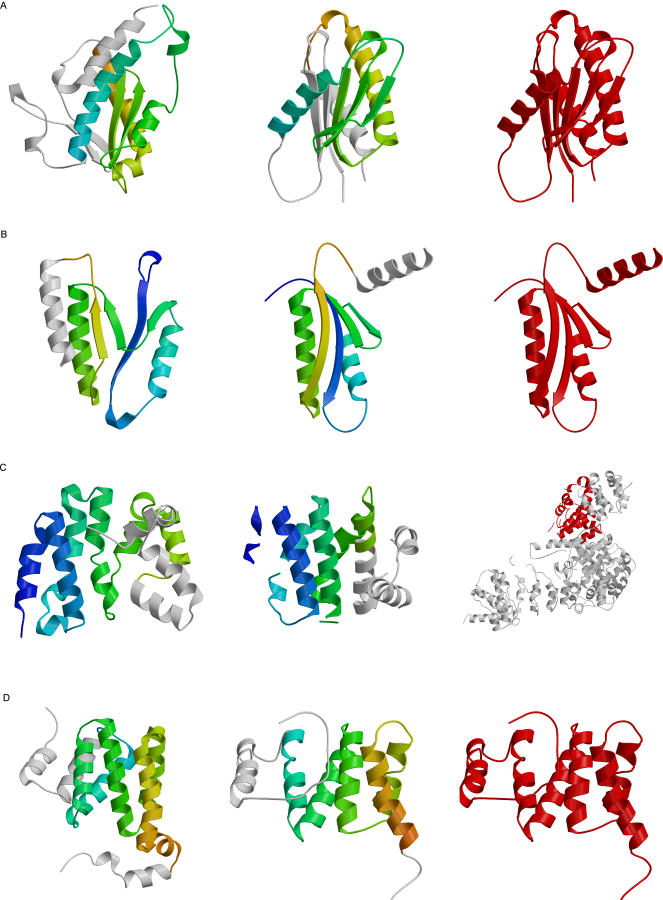
Predictions of Domains with Confident SCOP Superfamily Assignment Scores That Were Subsequently Solved The predicted structure is shown in panel 1 (left), and the native structure or the structure of the homolog is shown in panel 2 (middle). The section matching the domain in the solved structure is colored red in panel 3 (right). (A) TRS20/YBR254C is involved in the endoplasmic reticulum (ER) to Golgi transport and was predicted to belong to the SNARE-like (d.110.4) SCOP superfamily with a *P*(SF|D,GO) = 0.98 (*P*_MCM_ = 0.69). The *Z*-score between the predicted structure and the structure of a homolog with 33% sequence identity was 7.76. (B) ECM15/YBL001C is involved in cell wall organization and biogenesis, and was predicted to belong to the MTH1187/YkoF-like (d.58.48) superfamily with *P*(SF|D,GO) = 0.87 (*P*_MCM_ = 0.82; MAMMOTH *Z*-score to native structure was 8.18). (C) KAP104/YBR017C is involved in nuclear localization sequence binding and was predicted to belong to the ARM repeat (a.118.1) superfamily with *P*(SF|D,GO) = 0.99 (*P*_MCM_ = 0.8; MAMMOTH *Z*-score to homologous structure [31% sequence identity] was 4.41). (D) FIS1/YIL065C was predicted to belong to the SCOP superfamily TPR-like (a.118.8) with a *P*_MCM_ of 0.98. The Mammoth *Z*-score between the predicted structure and the experimental structure was 13.34.

True performance of these technologies cannot be assessed on the benchmark dataset because the domain boundaries of this set are perfect (derived from known structures in the ASTRAL database). A subset of the proteins without links to known structure at the start of this project now have strong homology to a structure that has since been solved, see Figure 4 for examples. These recently solved structures give us an opportunity to assess the performance of our technology without bias in the selection of the proteins, with real domain prediction error incorporated, and without the contamination of the results by weak homology to known structures. Twenty-seven domains from this project now have a homolog of known structure (see Materials and Methods for homology definition) and three of the 11 predictions with a *P*_MCM_ of 0.8 or higher are correct. Five of seven proteins from this set with a *P*(SF|D,GO) ≥ 0.8 are correct; five of which (three correct) also have a *P*_MCM_ of 0.8. The small sample size represented by this dataset makes it difficult to assess accurate upper and lower bounds of the estimated error. We have also generated a much larger dataset (see Materials and Methods), as part of the Human Proteome Folding Project (HPF). This dataset, containing proteins from over 150 organisms, was derived without the use of fold recognition (and thus is not identical to the protocol for yeast), but provides valuable information as to the effect of domain prediction on our procedure. A total of 44% of the 207 predictions that were made for recently solved structures with *P*_MCM_ above 0.8 in this dataset were correct; 84% of the 51 predictions with *P*(SF|D,GO) above 0.8 were correct; and 31 of these predictions had both a *P*_MCM_ and a *P*(SF|D,GO) above 0.8, and 27 of these were correct. Over all three validation sets, more than 40% of the predictions with a *P*_MCM_ above 0.8, and more than 75% of the predictions with a *P*(SF|D,GO) above 0.8, are correct, illustrating the value of data integration in this work.

Importantly, we were able to use these sets of recently solved proteins to better characterize the errors associated with different confidence Ginzu domain predictions. We found that a subset of the incorrect domain parses which significantly diminish the chances of correctly predicting fold and function are easily removed using a simple filter (described in Materials and Methods). This domain-prediction filter allows us to recover more-accurate predictions for multi-domain proteins. We were able to classify 50% of the amino acids from the 6,238 attempted ORFs to SCOP superfamilies which is significantly higher than the 35% coverage achieved by a sequence-based hidden Markov model approach [30].

### Novel SCOP Superfamily Assignments

In this section, we discuss several protein complexes with components assigned to superfamilies by both GO-integration and MCM approaches. These predictions and the much larger set of predictions in the database accompanying this paper provide a basis for hypothesis generation and experimental testing, but it must be borne in mind that there is a significant probability that any single prediction is incorrect, as indicated by our estimates of error.

The mediator complex, a large complex containing 24 polypeptides [31], has been shown to be required for transcriptional activation in many eukaryotic organisms and play key roles in transmitting regulatory information to the pre-initiation complex. During transcriptional initiation, it interacts with the RNA polymerase II holoenzyme and the promoter region. The role of the mediator complex in transcriptional regulation, and the complete makeup of this complex and its dynamic composition throughout different cell and developmental states (in response to specific regulators) are active areas of research. To date, several studies have explored the overall makeup of the complex by probing protein–protein interactions [31] and by electron microscopy of purified mediator complex, but to our knowledge, this complex has eluded higher resolution methods such as crystallographic analysis. Although there exists an extensive body of work on the overall function of this complex, the roles, positions, and structures of most of the individual polypeptide components remain undetermined.

**Figure 5:**
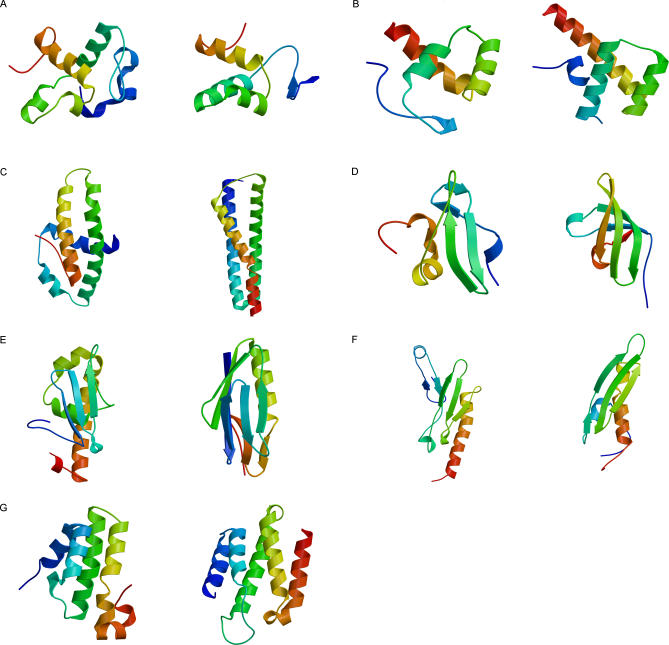
Structure Predictions with High *P*(SF|D,GO) The predicted structure with the highest *P*(SF|D,GO) (left) is shown for (A) ROX3, (B) MED4, (C) GAL11, (D) TIF35, (E) MRPL37, (F) MRPL44, and (G) INH1 displayed next to the matching SCOP representative (right).

We find confident superfamily predictions for several proteins within this complex that were not structurally annotated prior to this work. Table 4 outlines these predictions, as well as their sources and confidence estimates. Several proteins in the Mediator head domain are predicted to contain DNA-binding domains. In addition, multiple head domain proteins are predicted to be long helical bundles, potentially serving as scaffolds. ROX3 contains two predicted domains, see Figure 5A. The first domain is predicted to belong to the Homeodomain-like superfamily (*P*_MCM_ = 0.43; GO-term: transcription from RNA polymerase II promoter; *P*(SF|D,GO) = 0.83); implying DNA binding. MED4 (Figure 5B), in the middle region of the mediator complex, is also a two-domain protein with a N-terminal homeodomain-like superfamily assignment (*P*_MCM_ = 0.65; GO-term: transcription from RNA polymerase II promoter; *P*(SF|D,GO) = 0.98). Several superfamily predictions for this complex (such as hits to superfamilies like spectrin-like and Rossmann folds) are difficult to interpret unambiguously due to the large number of functions compatible with each of these superfamilies, but are not incompatible with DNA-binding functions. Gal11 (Figure 5C) contains a diverse mix of predicted domain structures: for the first domain, we find a PSI-BLAST match to the motor domain of myosin, and the second domain shows a strong fold recognition hit to a structural domain from sec24 (a component of the secretion system). Rosetta models for the third domain match the spectrin-like superfamily, and the models of the fourth domain match the DNA-binding lambda-repressor–like fold (with a competing hit to the tRNA-binding fold). The fifth domain shows a match to the RNA pol II–like fold (RPB1) by confident fold recognition. Although these domain and structure predictions are insufficient by themselves to localize specific molecular function to components of the mediator complex, it is encouraging that we can make some headway in localizing specific superfamilies and functions to components of this large complex.

**Table 4:**
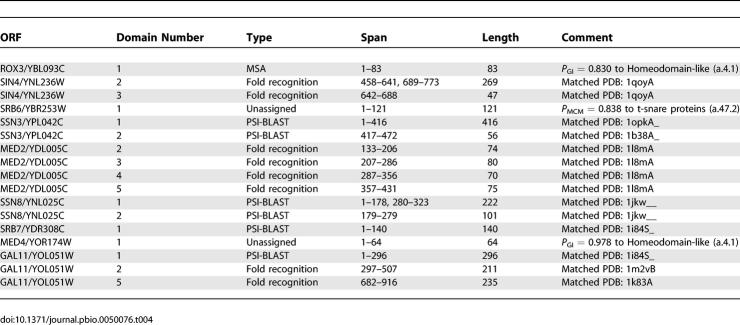
Predicted Domains and Structures for Components of the Mediator Complex

TIF35 (Figure 5D) is a subunit of the translation initiation factor complex, a complex essential for translation [32]. We predict three separate domains exist within this protein. The middle and C-terminal domains of this protein are both strongly predicted to belong to the RNA-binding domain, RDB superfamily (d.58.7; identified by PSI-BLAST). The N-terminal 65 amino acids lack sequence-detectable homologs of known structure, but the Rosetta-generated models and GO-selected structure prediction for this protein shows a strong match to the Translation proteins SH3-like domain (*P*_MCM_ = 0.44; GO-term: translation initiation factor activity; *P*(SF|D,GO) = 0.92).

The mitochondrial ribosome, or the mitoribosome, shares a number of protein components with bacterial ribosomes, but it is believed that the mitoribosomes have comparatively more proteins than their bacterial counterparts; many of the proteins associated with the mitoribosome have no detectable sequence similarity to other mitochondrial proteins [33]. We have predicted the structure for two components known to be associated with the mitoribosome [34,35]. MRPL37 (Figure 5E) is predicted to belong to the Ribosomal protein L6 superfamily (*P*_MCM_ = 0.31; GO-term structural constituent of ribosome; *P*(SF|D,GO) = 0.86), a superfamily involved in RNA binding. We predict that MRPL44 (Figure 5F) belongs to the dsRNA-binding domain–like superfamily (*P*_MCM_ = 0.78; GO-term: structural constituent of ribosome; *P*(SF|D,GO) = 0.86). Overall, the structure predictions for these mitoribosome proteins suggest that they belong to superfamilies compatible with known, although highly diverged, components of both the bacterial and eukaryotic ribosome.

INH1 (Figure 5G) is an ATPase inhibitor predicted to be a member of the ARM repeat superfamily (*P*_MCM_ = 0.73; GO-term: ATP synthesis couple protein transport; *P*(SF|D,GO) = 0.82). Inh1 dimerizes and binds to the F_1_ complex of the ATPase, thereby inhibiting its function [36,37]. Many members of the ARM repeat superfamily are involved in protein and peptide binding, which is consistent with both the dimerzation and the binding to the ATPase.

### Data Access

All data are accessible via the Yeast Resource Center (YRC) public data repository [38] at http://rd.plos.org/10.1371_journal.pbio.0050076_01. The data will also be made available in other formats upon request.

## Discussion

Comprehensive generation of three-dimensional structures with resolution or reliability of those determined by X-ray crystallography or nuclear magnetic resonance (NMR) is currently beyond the capabilities of any protein structure prediction method; these methods can, however, play an important role in generating structural annotations for whole genomes due to the much lower investment of resources required per protein domain. In this work, we have shown that it is possible to: (1) generate protein structure models on a genome-wide scale, (2) automate the assessment of the structure prediction quality, (3) convert the results into pre-existing encodings of structure in the form of SCOP superfamily classifications, and (4) augment the model-based assignment of SCOP superfamily by integrating with pre-existing function, process, and component information encoded in the GO database.

We were able to assign SCOP superfamilies to 7,094 of the 14,934 predicted domains in yeast using PSI-BLAST and fold recognition methodology. A total of 4,006 of the remaining 7,840 domains were short enough (less than 150 amino acids) for de novo structure prediction. Of these, 668 were omitted because they contained at least one predicted transmembrane helix. Low-resolution structure models were built for the remaining domains using Rosetta; of these, 404 were assigned to superfamilies with confidence using MCM, and an additional 177 were assigned with confidence after integrating with GO process, component, and function annotations.

A significant challenge in carrying out this work was the magnitude of the computation required for generating de novo structure predictions for large numbers of domains. Robust and fast methodology, efficient data storage, analysis tools, and data organization were required. Our use of distributed computing (http://wcgrid.org), innovative database architecture [39,40], and fully automatic methods were essential for this full-genome annotation. Yeast is particularly interesting because it is the focus of a vast global research effort. Future work will include an ongoing effort to scale this procedure to over 150 completely sequenced genomes as well as to employ recently developed higher resolution structure prediction methods [41] that produce more-accurate and reliable models, but require significantly greater computational resources per protein domain.

The information content in the predicted structures may be further leveraged by integration with other data such as global quantitative measurements of mRNA, protein expression levels, DNA–protein, and protein–protein interactions. Such datasets are available for yeast and several other organisms as part of ongoing functional genomics efforts, and integration of these data types with the predicted structures should contribute to the annotation of protein functions.

## Materials and Methods

### Benchmark set; folding representatives from SCOP.

Two representative domains from each SCOP [12–14] superfamily were folded using the Rosetta de novo method [1,2,28]. Superfamilies without members shorter than 200 amino acids were excluded, as were proteins for which Rosetta failed to produce predictions within a reasonable time. One thousand models were generated for each domain. This resulted in structure predictions for 998 domains for which the structures have been experimentally determined. The predicted structures were clustered by root mean square deviation (RMSD), and the centers of the top 30 clusters were compared to a domain database generated from ASTRAL 1.67 (reduced to 40% sequence identity) [42,43] using a modified version of MAMMOTH [21] that calculates the contact order of the aligned regions of the predicted structure and the ASTRAL domain. An overview of the statistics is presented in Table 5, and a detailed description of the results in Table S4.

**Table 5:**
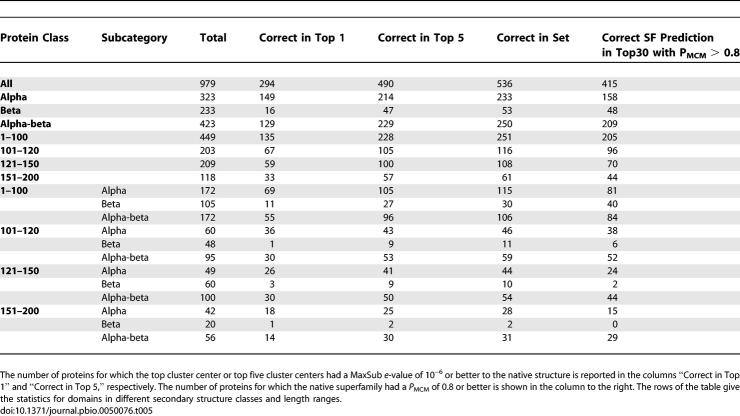
Summary of Benchmark Results

### The MAMMOTH Confidence Metric.

The MCM estimates the probability that the MAMMOTH match between predicted structure and the ASTRAL domain (see previous section) has identified the correct superfamily and is based on the closeness of match (MAMMOTH *Z*-score), the length of the two proteins involved, *L*_Astral_ and *L*_predicted_, the CO of the region of the predicted structure that was superimposable on the experimental structure, and the degree to which Rosetta converged during the generation of the set of predicted conformations (converg below; estimated during the clustering step). The general formula for the confidence functions is given in Equation 2, and the weights of the parameters (*a, b, c, d,* and the constant C) for the three models described in the following paragraph are presented in Table 3.

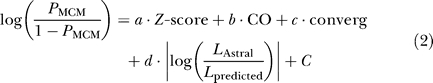



This model is similar to that used in previous studies [11], with two improvements. First, we have fit three separate logistic regression models, one for all alpha proteins, one for all beta proteins, and one for alpha and beta proteins; the size of the benchmark set and the fact that we are fitting a small number of parameters allows for this trifurcation of the benchmark set. Second, we compute the CO [29] over the matched region. This penalizes the scenario in which small numbers of long secondary structure elements (usually helices) are aligned; the CO term as well as the length ratio corrects for the overly confident score we would otherwise calculate based on convergence and MAMMOTH *Z*-score alone. We used 5-fold cross-validation to fit each of the three secondary structure class–specific confidence functions. For selecting between the three models for a query protein, we use secondary structure content predicted by PsiPred [44]. The alpha model is used for proteins with over 15% predicted alpha-helical content and under 15% beta-sheet content. The beta model is used for protein with more than 15% predicted beta strand and less than 15% alpha helical. The alpha/beta model was used for all other domains.

### Estimating superfamily probabilities, given the structure predictions.

Given a set of predicted structures D for a given protein, we estimate the probability the protein belongs to superfamily, SF, *P*(SF|D) as follows. Each superfamily is initially assigned a probability corresponding to the maximum *P*_MCM_ value for that superfamily over the top five *P*_MCM_ values for all predicted conformations for the query protein; probabilities less than 0.2 are set to zero. If the sum of the raw probabilities is greater than 0.8, they are scaled linearly so that the sum is 0.8. Because of the uncertainties of de novo structure prediction, these scaled probabilities, *P*_scaled_(SF|D), are then linearly combined with the background superfamily distribution, *P*(SF) (Equation 3):

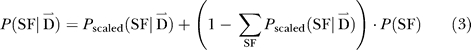



The final distributions, *P*(SF|D), are guaranteed to have non-zero probabilities for every superfamily, and to sum to 1. The background distribution *P*(SF) ensures that (1) we do not disregard useful functional information at the integration with GO stage and (2) that we do not over interpret the confidence values derived from the benchmark training set.

### Integration of function.

We obtain *P*(SF|D,GO) of a superfamily, SF, given both protein structure prediction, D, and GO annotations, GO, using Bayes' rule and the assumption that *P*(GO,D|SF) ∼ *P*(GO|SF)*P(D|SF):

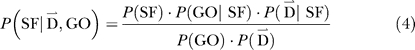
We obtain *P*(D|SF) via Equation 5:

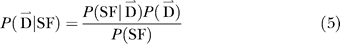



After substituting Equation 5 into Equation 4, both *P*(SF) and *P*(D) cancel. *P*(SF|D) is computed as described in the previous section, and *P*(GO|SF), *P*(GO), and *P*(SF) are computed from proteins in the PDB that are annotated with GO function, component, or process and also classified in SCOP. To deal with cases in which there is a single function annotation for a given superfamily, we allow for the possibility that the uniqueness of this mapping is due to under-sampling of superfamily space (as represented by the PDB) or function space (as represented by GO) by adding pseudo counts distributed according to the background superfamily distribution, *P*_astral95_(SF), computed from ASTRAL 1.67 culled so that no sequences are more then 95% identical.

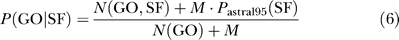



The parameter *M* (a regularization parameter controlling the relative contribution of our pseudo-counts) was estimated by carrying out function assignment given the superfamily over the benchmark set: we chose *M* to minimize the classification error estimated using 10-fold cross-validation. The overall procedure was relatively insensitive to the value of *M* ranging from one to ten with an optimal value of four. The *P*(SF|GO) are too diffuse for confident superfamily prediction from GO annotations alone, hence the integration with the structure prediction data is critical for accurate superfamily predictions.

Equation 6 relies on the assumption that the functional annotations are independent and mutually exclusive, which is not the case. (GO is a directed acyclic graph [DAG], with an implicit conditional dependence of lower nodes on parent nodes.) Nodes can have multiple parents, thus the probability of the child nodes of a more general term are not guaranteed to sum to the probability of the parent term. To circumvent this problem, we assigned the combined probability for each superfamily by taking the maximum probability for that superfamily given the predicted structures and all functions, i.e., Equation 7:

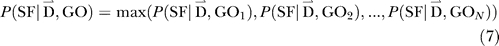



Finally, the sum of *P*(SF|D,GO) for any given protein domain is normalized to sum to one; thus confident assignments are not made when there are strong matches to more than one superfamily.

### Datasets for evaluation.

The performance of the MCM and the GO integration was evaluated on two independent datasets. The first dataset, from HPF project, consists of 768 predicted domains that now have a homolog with a known structure that is classified in SCOP 1.69. The homologs were identified by blasting predicted domains against all sequences from ASTRAL 1.69 and selecting those with a PSI-BLAST *e*-value less than 1 × 10^−3^. We also require that the shorter of the two sequences is more than 80% of the length of the longer one, and that 60% or more of the predicted domain is aligned with the ASTRAL domain. These domains are part of an ongoing project in which we predict structures for over 150 genomes; although domains with any homology to known structures are excluded, a number of structures have been solved and classified in SCOP during the 18 mo the project has been running. The scope of this separate project prohibited us from carrying out fold recognition calculations on these domains, and since domains that can be assigned using fold recognition methods will on average have higher MAMMOTH structural similarities to known structures than domains that cannot be assigned, results from this dataset represent an upper bound on performance on the dataset in this paper.

The second dataset was generated the same way the HPF set was generated, but limited to yeast domains. The proteins from which these domains are derived have been subjected to fold recognition and hence give a better estimate of the true performance. This dataset is, however, too small for statistically significant conclusions to be made.

### Domain filter.

Based on inspection of the results on the HPF dataset, domains from predicted two-domain proteins are excluded if both the domains are predicted using less-confident methods (MSA, unassigned, or Pfam domains), or if the domain under consideration is an MSA domain regardless of the neighboring domain type. A large fraction of these proteins have single domains, and correct superfamily matches are quite unlikely when models are only generated from domain fragments.

### Data production.

The generation of structure predictions was divided into three completely automated steps: pre-processing, production (the running of Rosetta), and post-processing (clustering, superfamily assignment, and function integration). The pre-processing protocol includes domain prediction, prediction of secondary structure, disordered regions, trans-membrane helices [45], and signal peptides [46], and the local structure fragments and other files necessary for running Rosetta. This step was conducted in-house on two 64-CPU Linux clusters. The production step, generating 10,000 structure predictions, was completed in collaboration with IBM running Rosetta on the World Community Grid as part of a larger effort, and is estimated to have used 12 million CPU hours, or 1,350 CPU years. The post-processing step was performed in-house (using the same hardware as the pre-processing step), and included clustering and superfamily assignment by MCM and GO integration. The resulting dataset is complex, and is stored, queried, organized, and analyzed using an open-source software package, 2DDB [39,40] of our own construction.

## Supporting Information

Table S1 Complete Listing of Domain Predictions for All ORFs in Yeast All 14,934 domains predicted from the 6,238 sequences are presented in detail.
(4.7 MB PDF)

Table S2 Protein Structure Predictions with *P*_MCM_ ≥ 0.8 The most confident predictions using the MCM are listed.
(133 KB PDF)

Table S3 Protein Structure Prediction with *P*(SF|D,GO) ≥ 0.8 The most confident predictions using the GO-integration strategy are listed.
(77 KB PDF)

Table S4 Benchmark Results Best predicted structure—the best predicted structure by RMS among the 1,000 created; Top5 is the cluster center from the five largest clusters; Best Match—the best domain match from all 30 cluster centers.
(476 KB PDF)

## Abbreviations

CO: contact order
GO: Gene Ontology
HPF: Human Proteome Folding Project
MCM: MAMMOTH confidence metric
MSA: multiple sequence alignment
ORF: open reading frame
PDB: the Protein Data Bank
