# Supplementary material for: Superfamily Assignments for the Yeast Proteome through Integration of Structure Prediction with the Gene Ontology
Source: PLoS Biol. 2007 Mar 20;5(4):e76. doi: 10.1371/journal.pbio.0050076 (PMC1828141; doi:10.1371/journal.pbio.0050076)
Supplement: Supplementary file 1 [file pbio.0050076.st001.pdf]

ac	gene name	domain nr	detect_metho	parent	length
TY1A_A		1	pfam	PF01021.9	157
TY1A_A		2	msa	na	172
TY1A_A		3	msa	na	111
TY1A_BL		1	pfam	PF01021.9	180
TY1A_BL		2	unassigned	na	148
TY1A_BL		3	msa	na	112
TY1A_BR		1	pfam	PF01021.9	161
TY1A_BR		2	unassigned	na	120
TY1A_BR		3	msa	na	159
TY1A_DR1		1	pfam	PF01021.9	158
TY1A_DR1		2	unassigned	na	175
TY1A_DR1		3	msa	na	107
TY1A_DR2		1	pfam	PF01021.9	176
TY1A_DR2		2	unassigned	na	153
TY1A_DR2		3	msa	na	111
TY1A_DR3		1	pfam	PF01021.9	173
TY1A_DR3		2	unassigned	na	155
TY1A_DR3		3	msa	na	112
TY1A_DR5		1	pfam	PF01021.9	171
TY1A_DR5		2	unassigned	na	110
TY1A_DR5		3	msa	na	159
TY1A_DR6		1	pfam	PF01021.9	180
TY1A_DR6		2	unassigned	na	101
TY1A_DR6		3	msa	na	159
TY1A_GR1		1	pfam	PF01021.9	122
TY1A_GR1		2	msa	na	158
TY1A_GR1		3	msa	na	160
TY1A_GR2		1	pfam	PF01021.9	136
TY1A_GR2		2	unassigned	na	197
TY1A_GR2		3	msa	na	107
TY1A_H		1	pfam	PF01021.9	193
TY1A_H		2	unassigned	na	178
TY1A_H		3	msa	na	107
TY1A_JR1		1	pfam	PF01021.9	176
TY1A_JR1		2	unassigned	na	126
TY1A_JR1		3	msa	na	138
TY1A_JR2		1	pfam	PF01021.9	176
TY1A_JR2		2	unassigned	na	157
TY1A_JR2		3	msa	na	107
TY1A_ML2		1	pfam	PF01021.9	176
TY1A_ML2		2	unassigned	na	105
TY1A_ML2		3	msa	na	159
TY1A_MR1		1	pfam	PF01021.9	166
TY1A_MR1		2	unassigned	na	164
TY1A_MR1		3	msa	na	110
TY1A_NL1		1	pfam	PF01021.9	149
TY1A_NL1		2	unassigned	na	184

TY1A_NL1		3	msa	na	107
TY1A_NL2		1	pfam	PF01021.9	176
TY1A_NL2		2	unassigned	na	152
TY1A_NL2		3	msa	na	112
TY1A_OL		1	pfam	PF01021.9	177
TY1A_OL		2	unassigned	na	103
TY1A_OL		3	msa	na	160
TY1A_OR		1	pfam	PF01021.9	156
TY1A_OR		2	unassigned	na	178
TY1A_OR		3	msa	na	106
TY2A_B		1	pfam	PF01021.9	193
TY2A_B		2	unassigned	na	84
TY2A_B		3	msa	na	161
TY2A_C		1	pfam	PF01021.9	172
TY2A_C		2	unassigned	na	126
TY2A_C		3	msa	na	140
TY2A_DR1		1	pfam	PF01021.9	175
TY2A_DR1		2	unassigned	na	100
TY2A_DR1		3	msa	na	163
TY2A_DR2		1	pfam	PF01021.9	169
TY2A_DR2		2	unassigned	na	158
TY2A_DR2		3	msa	na	111
TY2A_DR3		1	pfam	PF01021.9	173
TY2A_DR3		2	msa	na	98
TY2A_DR3		3	unassigned	na	56
TY2A_DR3		4	msa	na	111
TY2A_F		1	pfam	PF01021.9	193
TY2A_F		2	unassigned	na	65
TY2A_F		3	msa	na	180
TY2A_LR1		1	pfam	PF01021.9	160
TY2A_LR1		2	unassigned	na	168
TY2A_LR1		3	msa	na	110
TY2A_OR1		1	pfam	PF01021.9	156
TY2A_OR1		2	unassigned	na	171
TY2A_OR1		3	msa	na	111
TY2A_OR2		1	pfam	PF01021.9	171
TY2A_OR2		2	unassigned	na	127
TY2A_OR2		3	msa	na	140
TY3A_G		1	unassigned	na	290
TY3A_I		1	unassigned	na	290
TY4A_H		1	unassigned	na	153
TY4A_H		2	unassigned	na	260
TY4A_J		1	unassigned	na	153
TY4A_J		2	unassigned	na	261
YAL001C	TFC3	1	msa	na	262
YAL001C	TFC3	2	unassigned	na	898
YAL002W	VPS8	1	unassigned	na	392
YAL002W	VPS8	2	pfam	PF00637	196

YAL002W	VPS8	3	unassigned	na	212
YAL002W	VPS8	4	pfam	PF00637	214
YAL002W	VPS8	5	msa	na	162
YAL003W	EFB1	1	psiblast	1jlvA_	87
YAL003W	EFB1	2	psiblast	1f60B_	119
YAL004W	YAL004W	1	msa	na	215
YAL005C	SSA1	1	psiblast	1hpm__	191
YAL005C	SSA1	2	psiblast	1hpm__	116
YAL005C	SSA1	3	psiblast	1hpm__	75
YAL005C	SSA1	4	psiblast	1ckrA_	158
YAL005C	SSA1	5	fold_recogniti	1dkxA	102
YAL007C	ERP2	1	pfam	PF01105	215
YAL008W	FUN14	1	unassigned	na	198
YAL009W	SPO7	1	pfam	PF03907	259
YAL010C	MDM10	1	msa	na	326
YAL010C	MDM10	2	unassigned	na	167
YAL011W	SWC3	1	unassigned	na	52
YAL011W	SWC3	2	psiblast	1i84S_	392
YAL011W	SWC3	3	msa	na	194
YAL012W	CYS3	1	psiblast	1e5eA_	273
YAL012W	CYS3	2	psiblast	1e5eA_	121
YAL013W	DEP1	1	msa	na	162
YAL013W	DEP1	2	unassigned	na	200
YAL014C	SYN8	1	unassigned	na	205
YAL015C	NTG1	1	unassigned	na	119
YAL015C	NTG1	2	psiblast	2abk__	168
YAL015C	NTG1	3	psiblast	2abk__	112
YAL016W	TPD3	1	psiblast	1b3uA_	224
YAL016W	TPD3	2	psiblast	1b3uA_	72
YAL016W	TPD3	3	psiblast	1b3uA_	145
YAL016W	TPD3	4	psiblast	1b3uA_	194
YAL017W	PSK1	1	fold_recogniti	1qhbA	590
YAL017W	PSK1	2	unassigned	na	322
YAL017W	PSK1	3	psiblast	1dsyA_	142
YAL017W	PSK1	4	psiblast	1fotA_	117
YAL017W	PSK1	5	psiblast	1fotA_	185
YAL018C	YAL018C	1	unassigned	na	185
YAL018C	YAL018C	2	msa	na	140
YAL019W	FUN30	1	msa	na	215
YAL019W	FUN30	2	psiblast	1gm5A_	97
YAL019W	FUN30	3	psiblast	1gm5A_	123
YAL019W	FUN30	4	psiblast	1gm5A_	336
YAL019W	FUN30	5	unassigned	na	81
YAL019W	FUN30	6	psiblast	1hv8A_	213
YAL019W	FUN30	7	psiblast	1d9xA_	66
YAL020C	ATS1	1	psiblast	1a12A_	333
YAL021C	CCR4	1	psiblast	1jl5A_	397
YAL021C	CCR4	2	psiblast	1k14A_	61

YAL021C	CCR4	3	fold_recogniti	1hd7A	379
YAL022C	FUN26	1	msa	na	171
YAL022C	FUN26	2	pfam	PF01733	346
YAL023C	PMT2	1	pfam	PF02366	334
YAL023C	PMT2	2	pfam	PF02815	65
YAL023C	PMT2	3	pfam	PF02815	67
YAL023C	PMT2	4	pfam	PF02815	77
YAL023C	PMT2	5	msa	na	215
YAL024C	LTE1	1	pfam	PF00618	121
YAL024C	LTE1	2	unassigned	na	842
YAL024C	LTE1	3	msa	na	127
YAL024C	LTE1	4	unassigned	na	71
YAL024C	LTE1	5	psiblast	1bkdS_	274
YAL025C	MAK16	1	msa	na	247
YAL025C	MAK16	2	unassigned	na	59
YAL026C	DRS2	1	unassigned	na	136
YAL026C	DRS2	2	psiblast	1eulA_	219
YAL026C	DRS2	3	psiblast	1eulA_	414
YAL026C	DRS2	4	psiblast	1eulA_	251
YAL026C	DRS2	5	psiblast	1eulA_	245
YAL026C	DRS2	6	msa	na	90
YAL027W	YAL027W	1	unassigned	na	261
YAL028W	FRT2	1	unassigned	na	528
YAL029C	MYO4	1	psiblast	1jwyA_	64
YAL029C	MYO4	2	psiblast	1jwyA_	360
YAL029C	MYO4	3	psiblast	1jwyA_	259
YAL029C	MYO4	4	psiblast	1jwyA_	96
YAL029C	MYO4	5	psiblast	1wdcA_	70
YAL029C	MYO4	6	psiblast	2tmaA_	65
YAL029C	MYO4	7	psiblast	2tmaA_	82
YAL029C	MYO4	8	psiblast	2tmaA_	60
YAL029C	MYO4	9	psiblast	2tmaA_	78
YAL029C	MYO4	10	fold_recogniti	1wq1G	164
YAL029C	MYO4	11	fold_recogniti	1wq1G	173
YAL030W	SNC1	1	psiblast	1sfcA_	117
YAL031C	FUN21	1	unassigned	na	444
YAL031C	FUN21	2	fold_recogniti	1ldvA	54
YAL031C	FUN21	3	fold_recogniti	1ei3A	262
YAL032C	PRP45	1	msa	na	57
YAL032C	PRP45	2	pfam	PF02731	255
YAL032C	PRP45	3	unassigned	na	67
YAL033W	POP5	1	pfam	PF01900	173
YAL034C	FUN19	1	msa	na	227
YAL034C	FUN19	2	unassigned	na	78
YAL034C	FUN19	3	msa	na	158
YAL034C-B	YAL034C-B	1	unassigned	na	117
YAL034W-A	MTW1	1	fold_recogniti	1jchA	218
YAL034W-A	MTW1	2	fold_recogniti	1jchA	71

YAL035W	FUN12	1	psiblast	1i84S_	304
YAL035W	FUN12	2	unassigned	na	81
YAL035W	FUN12	3	psiblast	1g7sA_	238
YAL035W	FUN12	4	psiblast	1g7sA_	118
YAL035W	FUN12	5	psiblast	1g7sA_	113
YAL035W	FUN12	6	psiblast	1g7sA_	148
YAL036C	RBG1	1	psiblast	1egaA_	268
YAL036C	RBG1	2	msa	na	101
YAL037C-A	YAL037C-A	1	unassigned	na	30
YAL037W	YAL037W	1	unassigned	na	267
YAL038W	CDC19	1	psiblast	1a3wA_	264
YAL038W	CDC19	2	psiblast	1a3wA_	236
YAL039C	CYC3	1	pfam	PF01265	269
YAL040C	CLN3	1	psiblast	1vin__	225
YAL040C	CLN3	2	psiblast	1vin__	126
YAL040C	CLN3	3	unassigned	na	229
YAL041W	CDC24	1	msa	na	133
YAL041W	CDC24	2	psiblast	1h67A_	120
YAL041W	CDC24	3	psiblast	1foeA_	205
YAL041W	CDC24	4	psiblast	1foeA_	184
YAL041W	CDC24	5	msa	na	112
YAL041W	CDC24	6	unassigned	na	100
YAL042C-A	YAL042C-A	1	unassigned	na	125
YAL042W	ERV46	1	msa	na	152
YAL042W	ERV46	2	msa	na	263
YAL043C	PTA1	1	msa	na	261
YAL043C	PTA1	2	unassigned	na	244
YAL043C	PTA1	3	msa	na	280
YAL044C	GCV3	1	psiblast	1dxmA_	177
YAL044W-A	YAL044W-A	1	pfam	PF01722	110
YAL045C	YAL045C	1	unassigned	na	102
YAL046C	YAL046C	1	pfam	PF01722	118
YAL047C	SPC72	1	psiblast	1i84S_	216
YAL047C	SPC72	2	psiblast	2tmaA_	64
YAL047C	SPC72	3	psiblast	2tmaA_	82
YAL047C	SPC72	4	psiblast	2tmaA_	61
YAL047C	SPC72	5	psiblast	2tmaA_	81
YAL047C	SPC72	6	msa	na	118
YAL048C	GON1	1	psiblast	1i4dD_	197
YAL048C	GON1	2	fold_recogniti	1a2xA	92
YAL048C	GON1	3	fold_recogniti	1a2xA	141
YAL048C	GON1	4	psiblast	1ceeA_	232
YAL049C	YAL049C	1	psiblast	1din__	246
YAL051W	OAF1	1	psiblast	1hwtD_	178
YAL051W	OAF1	2	unassigned	na	141
YAL051W	OAF1	3	msa	na	342
YAL051W	OAF1	4	unassigned	na	401
YAL053W	YAL053W	1	msa	na	160

YAL053W	YAL053W	2	unassigned	na	163
YAL053W	YAL053W	3	msa	na	309
YAL053W	YAL053W	4	msa	na	151
YAL054C	ACS1	1	msa	na	102
YAL054C	ACS1	2	psiblast	1amuA_	203
YAL054C	ACS1	3	psiblast	1amuA_	255
YAL054C	ACS1	4	psiblast	1amuA_	153
YAL055W	PEX22	1	unassigned	na	180
YAL056C-A	YAL056C-A	1	unassigned	na	116
YAL056W	GPB2	1	unassigned	na	847
YAL058W	CNE1	1	psiblast	1jhnA_	374
YAL058W	CNE1	2	psiblast	1jhnA_	128
YAL059W	ECM1	1	unassigned	na	212
YAL060W	BDH1	1	psiblast	1e3jA_	213
YAL060W	BDH1	2	psiblast	1e3jA_	169
YAL061W	YAL061W	1	psiblast	1e3jA_	247
YAL061W	YAL061W	2	psiblast	1e3jA_	170
YAL062W	GDH3	1	psiblast	1bgvA_	224
YAL062W	GDH3	2	psiblast	1bgvA_	233
YAL063C	FLO9	1	msa	na	147
YAL063C	FLO9	2	unassigned	na	82
YAL063C	FLO9	3	pfam	PF00624	87
YAL063C	FLO9	4	pfam	PF00624	45
YAL063C	FLO9	5	pfam	PF00624	45
YAL063C	FLO9	6	pfam	PF00624	45
YAL063C	FLO9	7	pfam	PF00624	45
YAL063C	FLO9	8	pfam	PF00624	45
YAL063C	FLO9	9	pfam	PF00624	45
YAL063C	FLO9	10	pfam	PF00624	45
YAL063C	FLO9	11	pfam	PF00624	45
YAL063C	FLO9	12	pfam	PF00624	45
YAL063C	FLO9	13	pfam	PF00624	45
YAL063C	FLO9	14	pfam	PF00624	45
YAL063C	FLO9	15	pfam	PF00624	52
YAL063C	FLO9	16	msa	na	178
YAL063C	FLO9	17	msa	na	281
YAL064C-A	YAL064C-A	1	unassigned	na	126
YAL064W	YAL064W	1	unassigned	na	108
YAL065C	YAL065C	1	unassigned	na	128
YAL066W	YAL066W	1	unassigned	na	102
YAL067C	SEO1	1	unassigned	na	110
YAL067C	SEO1	2	psiblast	1ja5A_	483
YAL068C	YAL068C	1	pfam	PF00660	120
YAR002C-A	ERP1	1	pfam	PF01105	219
YAR002W	NUP60	1	msa	na	69
YAR002W	NUP60	2	unassigned	na	129
YAR002W	NUP60	3	fold_recogniti	1m2vB	341
YAR003W	SWD1	1	psiblast	1erjA_	343

YAR003W	SWD1	2	psiblast	1crzA_	83
YAR007C	RFA1	1	psiblast	1ewiA_	116
YAR007C	RFA1	2	unassigned	na	60
YAR007C	RFA1	3	psiblast	1fguA_	123
YAR007C	RFA1	4	psiblast	1fguA_	141
YAR007C	RFA1	5	psiblast	1l1oC_	181
YAR008W	SEN34	1	unassigned	na	149
YAR008W	SEN34	2	psiblast	1a79A_	126
YAR014C	BUD14	1	msa	na	156
YAR014C	BUD14	2	unassigned	na	97
YAR014C	BUD14	3	psiblast	1g83A_	85
YAR014C	BUD14	4	unassigned	na	364
YAR015W	ADE1	1	psiblast	1a48__	68
YAR015W	ADE1	2	psiblast	1a48__	238
YAR018C	KIN3	1	psiblast	1ctpE_	300
YAR018C	KIN3	2	psiblast	1ctpE_	135
YAR019C	CDC15	1	psiblast	1f3mC_	101
YAR019C	CDC15	2	psiblast	1f3mC_	246
YAR019C	CDC15	3	unassigned	na	517
YAR019C	CDC15	4	msa	na	110
YAR020C	PAU7	1	unassigned	na	55
YAR023C	YAR023C	1	pfam	PF00674	179
YAR027W	UIP3	1	pfam	PF00674	235
YAR028W	YAR028W	1	pfam	PF00674	234
YAR029W	YAR029W	1	unassigned	na	74
YAR030C	YAR030C	1	unassigned	na	113
YAR031W	PRM9	1	msa	na	125
YAR031W	PRM9	2	pfam	PF00674	173
YAR033W	MST28	1	pfam	PF00674	234
YAR035W	YAT1	1	pfam	PF00755	687
YAR042W	SWH1	1	psiblast	1k1aA_	121
YAR042W	SWH1	2	psiblast	1k1aA_	135
YAR044W		1	psiblast	1i84S_	332
YAR044W		2	unassigned	na	85
YAR044W		3	msa	na	442
YAR047C	YAR047C	1	unassigned	na	106
YAR050W	FLO1	1	msa	na	259
YAR050W	FLO1	2	pfam	PF00624	57
YAR050W	FLO1	3	pfam	PF00624	45
YAR050W	FLO1	4	pfam	PF00624	45
YAR050W	FLO1	5	pfam	PF00624	45
YAR050W	FLO1	6	pfam	PF00624	45
YAR050W	FLO1	7	pfam	PF00624	45
YAR050W	FLO1	8	pfam	PF00624	45
YAR050W	FLO1	9	pfam	PF00624	45
YAR050W	FLO1	10	pfam	PF00624	45
YAR050W	FLO1	11	pfam	PF00624	45
YAR050W	FLO1	12	pfam	PF00624	45

YAR050W	FLO1	13	pfam	PF00624	45
YAR050W	FLO1	14	pfam	PF00624	45
YAR050W	FLO1	15	pfam	PF00624	45
YAR050W	FLO1	16	pfam	PF00624	45
YAR050W	FLO1	17	pfam	PF00624	45
YAR050W	FLO1	18	pfam	PF00624	45
YAR050W	FLO1	19	pfam	PF00624	58
YAR050W	FLO1	20	unassigned	na	90
YAR050W	FLO1	21	msa	na	353
YAR053W	YAR053W	1	unassigned	na	98
YAR060C	YAR060C	1	unassigned	na	111
YAR061W	YAR061W	1	unassigned	na	67
YAR062W	YAR062W	1	msa	na	117
YAR062W	YAR062W	2	pfam	PF00624	81
YAR064W	YAR064W	1	unassigned	na	99
YAR066W	YAR066W	1	unassigned	na	203
YAR068W	YAR068W	1	unassigned	na	161
YAR069C	YAR069C	1	unassigned	na	97
YAR070C	YAR070C	1	unassigned	na	99
YAR071W	PHO11	1	psiblast	1qfxA_	169
YAR071W	PHO11	2	psiblast	1qfxA_	298
YAR073W	IMD1	1	psiblast	1b3oA_	403
YAR075W	YAR075W	1	psiblast	1b3oA_	157
YBL001C	ECM15	1	pfam	PF01910	104
YBL002W	HTB2	1	psiblast	1id3D_	131
YBL003C	HTA2	1	psiblast	1id3C_	59
YBL003C	HTA2	2	psiblast	1id3C_	73
YBL004W	UTP20	1	msa	na	351
YBL004W	UTP20	2	msa	na	631
YBL004W	UTP20	3	unassigned	na	701
YBL004W	UTP20	4	msa	na	810
YBL005W	PDR3	1	psiblast	1ajyA_	103
YBL005W	PDR3	2	unassigned	na	301
YBL005W	PDR3	3	msa	na	134
YBL005W	PDR3	4	unassigned	na	438
YBL006C	LDB7	1	unassigned	na	145
YBL007C	SLA1	1	fold_recogniti	1gbqA	73
YBL007C	SLA1	2	psiblast	1azeA_	94
YBL007C	SLA1	3	unassigned	na	172
YBL007C	SLA1	4	psiblast	1g83A_	76
YBL007C	SLA1	5	psiblast	1g83A_	87
YBL007C	SLA1	6	unassigned	na	289
YBL007C	SLA1	7	fold_recogniti	1jv2B	272
YBL007C	SLA1	8	fold_recogniti	1jv2B	58
YBL007C	SLA1	9	fold_recogniti	1ldvA	123
YBL008W	HIR1	1	psiblast	1gg2B_	77
YBL008W	HIR1	2	psiblast	1erjA_	53
YBL008W	HIR1	3	psiblast	1k32A_	405

YBL008W	HIR1	4	unassigned	na	72
YBL008W	HIR1	5	msa	na	233
YBL009W	YBL009W	1	unassigned	na	368
YBL009W	YBL009W	2	psiblast	1aq1__	121
YBL009W	YBL009W	3	psiblast	1aq1__	72
YBL009W	YBL009W	4	psiblast	1aq1__	51
YBL009W	YBL009W	5	psiblast	1aq1__	64
YBL010C	YBL010C	1	unassigned	na	280
YBL011W	SCT1	1	unassigned	na	80
YBL011W	SCT1	2	psiblast	1k30A_	314
YBL011W	SCT1	3	unassigned	na	212
YBL011W	SCT1	4	msa	na	153
YBL012C	YBL012C	1	unassigned	na	133
YBL013W	FMT1	1	psiblast	1fmtA_	276
YBL013W	FMT1	2	psiblast	1fmtA_	117
YBL014C	RRN6	1	unassigned	na	894
YBL015W	ACH1	1	psiblast	1k6dA_	234
YBL015W	ACH1	2	psiblast	1poiB_	292
YBL016W	FUS3	1	psiblast	1erk__	105
YBL016W	FUS3	2	psiblast	1erk__	248
YBL017C	PEP1	1	msa	na	217
YBL017C	PEP1	2	unassigned	na	74
YBL017C	PEP1	3	msa	na	303
YBL017C	PEP1	4	unassigned	na	91
YBL017C	PEP1	5	msa	na	736
YBL017C	PEP1	6	unassigned	na	158
YBL018C	POP8	1	unassigned	na	133
YBL019W	APN2	1	psiblast	1hd7A_	376
YBL019W	APN2	2	msa	na	144
YBL020W	RFT1	1	msa	na	497
YBL020W	RFT1	2	unassigned	na	77
YBL021C	HAP3	1	psiblast	1jfiB_	144
YBL022C	PIM1	1	unassigned	na	155
YBL022C	PIM1	2	pfam	PF02190	232
YBL022C	PIM1	3	fold_recogniti	1jqjC	62
YBL022C	PIM1	4	fold_recogniti	1jqjC	132
YBL022C	PIM1	5	psiblast	1e32A_	187
YBL022C	PIM1	6	psiblast	1e32A_	122
YBL022C	PIM1	7	msa	na	98
YBL022C	PIM1	8	msa	na	145
YBL023C	MCM2	1	msa	na	121
YBL023C	MCM2	2	unassigned	na	54
YBL023C	MCM2	3	msa	na	122
YBL023C	MCM2	4	pfam	PF00493	180
YBL023C	MCM2	5	psiblast	1iqpA_	132
YBL023C	MCM2	6	psiblast	1iqpA_	191
YBL023C	MCM2	7	msa	na	68
YBL024W	NCL1	1	unassigned	na	74

YBL024W	NCL1	2	psiblast	1fbnA_	72
YBL024W	NCL1	3	psiblast	1j4fA_	218
YBL024W	NCL1	4	msa	na	130
YBL024W	NCL1	5	msa	na	190
YBL025W	RRN10	1	unassigned	na	145
YBL026W	LSM2	1	psiblast	1b34A_	95
YBL027W	RPL19B	1	psiblast	1k5yP_	70
YBL027W	RPL19B	2	psiblast	1k5yP_	119
YBL028C	YBL028C	1	unassigned	na	106
YBL029C-A	YBL029C-A	1	unassigned	na	94
YBL029W	YBL029W	1	unassigned	na	376
YBL030C	PET9	1	pfam	PF00153	122
YBL030C	PET9	2	pfam	PF00153	102
YBL030C	PET9	3	pfam	PF00153	94
YBL031W	SHE1	1	unassigned	na	338
YBL032W	HEK2	1	psiblast	1e3pA_	116
YBL032W	HEK2	2	psiblast	1e3pA_	265
YBL033C	RIB1	1	psiblast	1g57A_	134
YBL033C	RIB1	2	unassigned	na	52
YBL033C	RIB1	3	msa	na	159
YBL034C	STU1	1	unassigned	na	216
YBL034C	STU1	2	msa	na	243
YBL034C	STU1	3	unassigned	na	60
YBL034C	STU1	4	msa	na	362
YBL034C	STU1	5	unassigned	na	632
YBL035C	POL12	1	unassigned	na	198
YBL035C	POL12	2	msa	na	507
YBL036C	YBL036C	1	psiblast	1b54__	257
YBL037W	APL3	1	psiblast	1gw5A_	135
YBL037W	APL3	2	psiblast	1gw5A_	84
YBL037W	APL3	3	psiblast	1gw5A_	162
YBL037W	APL3	4	psiblast	1gw5A_	76
YBL037W	APL3	5	psiblast	1gw5A_	119
YBL037W	APL3	6	unassigned	na	167
YBL037W	APL3	7	psiblast	1qtsA_	145
YBL037W	APL3	8	psiblast	1qtsA_	137
YBL038W	MRPL16	1	psiblast	1lnrK_	232
YBL039C	URA7	1	fold_recogniti	1cp2A	208
YBL039C	URA7	2	psiblast	1bxrB_	371
YBL040C	ERD2	1	pfam	PF00810	219
YBL041W	PRE7	1	psiblast	1g0uL_	241
YBL042C	FUI1	1	unassigned	na	83
YBL042C	FUI1	2	pfam	PF02133	556
YBL043W	ECM13	1	msa	na	257
YBL044W	YBL044W	1	unassigned	na	122
YBL045C	COR1	1	psiblast	1ezvA_	250
YBL045C	COR1	2	psiblast	1ezvA_	207
YBL046W	YBL046W	1	unassigned	na	95

YBL046W	YBL046W	2	fold_recogniti	1l8mA	99
YBL046W	YBL046W	3	fold_recogniti	1l8mA	79
YBL046W	YBL046W	4	fold_recogniti	1l8mA	70
YBL046W	YBL046W	5	fold_recogniti	1l8mA	98
YBL047C	EDE1	1	psiblast	1qjtA_	115
YBL047C	EDE1	2	psiblast	1eh2__	129
YBL047C	EDE1	3	psiblast	1i84S_	305
YBL047C	EDE1	4	psiblast	2tmaA_	41
YBL047C	EDE1	5	psiblast	2tmaA_	82
YBL047C	EDE1	6	psiblast	2tmaA_	62
YBL047C	EDE1	7	psiblast	2tmaA_	89
YBL047C	EDE1	8	unassigned	na	102
YBL047C	EDE1	9	fold_recogniti	1l8mA	74
YBL047C	EDE1	10	fold_recogniti	1l8mA	79
YBL047C	EDE1	11	fold_recogniti	1l8mA	70
YBL047C	EDE1	12	fold_recogniti	1l8mA	75
YBL047C	EDE1	13	fold_recogniti	1ldvA	112
YBL047C	EDE1	14	pfam	PF00627	46
YBL048W	YBL048W	1	unassigned	na	103
YBL049W	MOH1	1	pfam	PF03226	138
YBL050W	SEC17	1	psiblast	1qqeA_	157
YBL050W	SEC17	2	psiblast	1qqeA_	135
YBL051C	PIN4	1	psiblast	1cvjA_	74
YBL051C	PIN4	2	psiblast	1up1__	90
YBL051C	PIN4	3	psiblast	1up1__	136
YBL051C	PIN4	4	msa	na	143
YBL051C	PIN4	5	psiblast	1g9lA_	87
YBL051C	PIN4	6	msa	na	138
YBL052C	SAS3	1	unassigned	na	242
YBL052C	SAS3	2	psiblast	1fy7A_	81
YBL052C	SAS3	3	psiblast	1fy7A_	105
YBL052C	SAS3	4	psiblast	1fy7A_	63
YBL052C	SAS3	5	psiblast	1fy7A_	96
YBL052C	SAS3	6	unassigned	na	244
YBL053W	YBL053W	1	unassigned	na	124
YBL054W	YBL054W	1	unassigned	na	77
YBL054W	YBL054W	2	psiblast	1mbe__	91
YBL054W	YBL054W	3	unassigned	na	357
YBL055C	YBL055C	1	fold_recogniti	1pscA	418
YBL056W	PTC3	1	psiblast	1a6q__	303
YBL056W	PTC3	2	psiblast	1a6q__	74
YBL056W	PTC3	3	unassigned	na	91
YBL057C	PTH2	1	unassigned	na	89
YBL057C	PTH2	2	pfam	PF01981	125
YBL058W	SHP1	1	unassigned	na	121
YBL058W	SHP1	2	msa	na	219
YBL058W	SHP1	3	psiblast	1jruA_	83
YBL059C-A	YBL059C-A	1	unassigned	na	109

YBL059W	YBL059W	1	unassigned	na	193
YBL060W	YBL060W	1	unassigned	na	64
YBL060W	YBL060W	2	psiblast	1bc9__	63
YBL060W	YBL060W	3	psiblast	1bc9__	162
YBL060W	YBL060W	4	unassigned	na	221
YBL060W	YBL060W	5	msa	na	177
YBL061C	SKT5	1	unassigned	na	106
YBL061C	SKT5	2	psiblast	1fchA_	114
YBL061C	SKT5	3	psiblast	1fchA_	69
YBL061C	SKT5	4	psiblast	1fchA_	90
YBL061C	SKT5	5	psiblast	1a17__	45
YBL061C	SKT5	6	psiblast	1a17__	128
YBL061C	SKT5	7	unassigned	na	144
YBL062W	YBL062W	1	unassigned	na	126
YBL063W	KIP1	1	psiblast	1ii6A_	419
YBL063W	KIP1	2	unassigned	na	69
YBL063W	KIP1	3	fold_recogniti	1epwA	299
YBL063W	KIP1	4	psiblast	1i84S_	324
YBL064C	PRX1	1	psiblast	1prxA_	211
YBL064C	PRX1	2	psiblast	1prxA_	50
YBL065W	YBL065W	1	unassigned	na	114
YBL066C	SEF1	1	psiblast	1pyiA_	161
YBL066C	SEF1	2	unassigned	na	76
YBL066C	SEF1	3	msa	na	511
YBL066C	SEF1	4	unassigned	na	309
YBL067C	UBP13	1	msa	na	132
YBL067C	UBP13	2	pfam	PF00442	56
YBL067C	UBP13	3	msa	na	343
YBL067C	UBP13	4	unassigned	na	66
YBL067C	UBP13	5	pfam	PF00443	91
YBL068W	PRS4	1	psiblast	1dkrA_	211
YBL068W	PRS4	2	psiblast	1dkrA_	144
YBL069W	AST1	1	psiblast	1tehA_	265
YBL069W	AST1	2	psiblast	1tehA_	164
YBL070C	YBL070C	1	unassigned	na	106
YBL071C	YBL071C	1	unassigned	na	102
YBL071W-A	KTI11	1	unassigned	na	82
YBL072C	RPS8A	1	pfam	PF01201	128
YBL072C	RPS8A	2	msa	na	72
YBL073W	YBL073W	1	unassigned	na	103
YBL074C	AAR2	1	unassigned	na	164
YBL074C	AAR2	2	msa	na	191
YBL075C	SSA3	1	psiblast	1hjoA_	191
YBL075C	SSA3	2	psiblast	1hjoA_	115
YBL075C	SSA3	3	psiblast	1hjoA_	75
YBL075C	SSA3	4	psiblast	1ckrA_	173
YBL075C	SSA3	5	fold_recogniti	1dkxA	95
YBL076C	ILS1	1	psiblast	1ile__	380

YBL076C	ILS1	2	psiblast	1ile__	115
YBL076C	ILS1	3	psiblast	1ile__	156
YBL076C	ILS1	4	psiblast	1ile__	45
YBL076C	ILS1	5	psiblast	1ile__	195
YBL076C	ILS1	6	msa	na	181
YBL077W	YBL077W	1	unassigned	na	143
YBL078C	ATG8	1	psiblast	1eo6A_	117
YBL079W	NUP170	1	unassigned	na	75
YBL079W	NUP170	2	pfam	PF03177	1427
YBL080C	PET112	1	pfam	PF02934	271
YBL080C	PET112	2	fold_recogniti	1m9iA	92
YBL080C	PET112	3	fold_recogniti	1m9iA	178
YBL081W	YBL081W	1	unassigned	na	110
YBL081W	YBL081W	2	fold_recogniti	1m2vB	258
YBL082C	RHK1	1	msa	na	346
YBL082C	RHK1	2	msa	na	112
YBL083C	YBL083C	1	unassigned	na	141
YBL084C	CDC27	1	fold_recogniti	1hh8A	102
YBL084C	CDC27	2	fold_recogniti	1hh8A	60
YBL084C	CDC27	3	fold_recogniti	1hh8A	50
YBL084C	CDC27	4	fold_recogniti	1ldvA	216
YBL084C	CDC27	5	psiblast	1fchA_	160
YBL084C	CDC27	6	psiblast	1fchA_	68
YBL084C	CDC27	7	psiblast	1fchA_	102
YBL085W	BOI1	1	psiblast	1g83A_	127
YBL085W	BOI1	2	unassigned	na	82
YBL085W	BOI1	3	psiblast	1kw4A_	85
YBL085W	BOI1	4	fold_recogniti	1i3qA	317
YBL085W	BOI1	5	unassigned	na	133
YBL085W	BOI1	6	psiblast	1fhwA_	164
YBL085W	BOI1	7	unassigned	na	72
YBL086C	YBL086C	1	unassigned	na	466
YBL087C	RPL23A	1	psiblast	1k5yR_	137
YBL088C	TEL1	1	msa	na	1325
YBL088C	TEL1	2	unassigned	na	394
YBL088C	TEL1	3	pfam	PF02259	383
YBL088C	TEL1	4	psiblast	1e7uA_	652
YBL088C	TEL1	5	pfam	PF02260	33
YBL089W	AVT5	1	pfam	PF01490	509
YBL090W	MRP21	1	unassigned	na	177
YBL091C	MAP2	1	psiblast	1b59A_	421
YBL091C-A	SCS22	1	unassigned	na	99
YBL092W	RPL32	1	psiblast	1k5yX_	58
YBL092W	RPL32	2	psiblast	1k5yX_	72
YBL093C	ROX3	1	msa	na	83
YBL093C	ROX3	2	unassigned	na	137
YBL094C	YBL094C	1	unassigned	na	110
YBL095W	YBL095W	1	unassigned	na	60

YBL095W	YBL095W	2	msa	na	210
YBL096C	YBL096C	1	unassigned	na	102
YBL097W	BRN1	1	msa	na	118
YBL097W	BRN1	2	unassigned	na	610
YBL098W	BNA4	1	psiblast	1bgn__	224
YBL098W	BNA4	2	psiblast	1bgn__	236
YBL099W	ATP1	1	psiblast	1cowA_	131
YBL099W	ATP1	2	psiblast	1cowA_	285
YBL099W	ATP1	3	psiblast	1cowA_	129
YBL100C	YBL100C	1	unassigned	na	104
YBL101C	ECM21	1	unassigned	na	507
YBL101C	ECM21	2	msa	na	196
YBL101C	ECM21	3	unassigned	na	414
YBL102W	SFT2	1	msa	na	215
YBL103C	RTG3	1	msa	na	276
YBL103C	RTG3	2	psiblast	1am9A_	146
YBL103C	RTG3	3	unassigned	na	64
YBL104C	YBL104C	1	fold_recogniti	1scgB	352
YBL104C	YBL104C	2	unassigned	na	52
YBL104C	YBL104C	3	msa	na	521
YBL105C	PKC1	1	pfam	PF02185	95
YBL105C	PKC1	2	fold_recogniti	1cxzB	97
YBL105C	PKC1	3	psiblast	1gmiA_	141
YBL105C	PKC1	4	unassigned	na	54
YBL105C	PKC1	5	pfam	PF00130	77
YBL105C	PKC1	6	psiblast	1tbn__	82
YBL105C	PKC1	7	unassigned	na	81
YBL105C	PKC1	8	psiblast	1dsyA_	170
YBL105C	PKC1	9	psiblast	1fotA_	141
YBL105C	PKC1	10	psiblast	1fotA_	213
YBL106C	SRO77	1	psiblast	1gg2B_	301
YBL106C	SRO77	2	psiblast	1c5kA_	170
YBL106C	SRO77	3	msa	na	245
YBL106C	SRO77	4	unassigned	na	294
YBL107C	YBL107C	1	unassigned	na	196
YBL107W-A	YBL107W-A	1	unassigned	na	34
YBL108C-A	YBL108C-A	1	unassigned	na	42
YBL108W	YBL108W	1	unassigned	na	101
YBL109W	YBL109W	1	unassigned	na	111
YBL111C	YBL111C	1	msa	na	237
YBL111C	YBL111C	2	unassigned	na	107
YBL111C	YBL111C	3	pfam	PF00270	271
YBL111C	YBL111C	4	unassigned	na	52
YBL112C	YBL112C	1	unassigned	na	105
YBL113C	YBL113C	1	fold_recogniti	1i3qA	372
YBL113C	YBL113C	2	unassigned	na	150
YBL113C	YBL113C	3	msa	na	172
YBL113C	YBL113C	4	unassigned	na	98

YBR001C	NTH2	1	unassigned	na	120
YBR001C	NTH2	2	pfam	PF01204	660
YBR002C	RER2	1	psiblast	1jp3A_	286
YBR003W	COQ1	1	unassigned	na	101
YBR003W	COQ1	2	psiblast	1ubx__	220
YBR003W	COQ1	3	psiblast	1ubx__	152
YBR004C	FMP44	1	msa	na	145
YBR004C	FMP44	2	msa	na	288
YBR005W	YBR005W	1	unassigned	na	213
YBR006W	UGA2	1	psiblast	1bi9A_	299
YBR006W	UGA2	2	psiblast	1bi9A_	198
YBR007C	YBR007C	1	unassigned	na	151
YBR007C	YBR007C	2	fold_recogniti	1i3qA	377
YBR007C	YBR007C	3	unassigned	na	60
YBR007C	YBR007C	4	fold_recogniti	1klxA	54
YBR007C	YBR007C	5	fold_recogniti	1klxA	94
YBR008C	FLR1	1	unassigned	na	77
YBR008C	FLR1	2	psiblast	1lviA_	471
YBR009C	HHF1	1	psiblast	1id3B_	103
YBR010W	HHT1	1	psiblast	1id3A_	136
YBR011C	IPP1	1	psiblast	1yppA_	287
YBR012C	YBR012C	1	unassigned	na	139
YBR013C	YBR013C	1	unassigned	na	129
YBR014C	YBR014C	1	unassigned	na	81
YBR014C	YBR014C	2	psiblast	1b4qA_	122
YBR015C	MNN2	1	unassigned	na	119
YBR015C	MNN2	2	fold_recogniti	1ll0A	194
YBR015C	MNN2	3	unassigned	na	284
YBR016W	YBR016W	1	unassigned	na	128
YBR017C	KAP104	1	unassigned	na	96
YBR017C	KAP104	2	msa	na	592
YBR017C	KAP104	3	msa	na	170
YBR017C	KAP104	4	unassigned	na	60
YBR018C	GAL7	1	psiblast	1hxqA_	366
YBR019C	GAL10	1	psiblast	1hzjA_	196
YBR019C	GAL10	2	psiblast	1hzjA_	61
YBR019C	GAL10	3	psiblast	1hzjA_	109
YBR019C	GAL10	4	psiblast	1l7jA_	333
YBR020W	GAL1	1	psiblast	1kkhA_	187
YBR020W	GAL1	2	psiblast	1kkhA_	185
YBR020W	GAL1	3	fold_recogniti	1kvkA	156
YBR021W	FUR4	1	unassigned	na	92
YBR021W	FUR4	2	pfam	PF02133	486
YBR021W	FUR4	3	unassigned	na	55
YBR022W	YBR022W	1	unassigned	na	177
YBR023C	CHS3	1	unassigned	na	72
YBR023C	CHS3	2	msa	na	471
YBR023C	CHS3	3	unassigned	na	84

YBR023C	CHS3	4	pfam	PF03142	538
YBR024W	SCO2	1	unassigned	na	109
YBR024W	SCO2	2	psiblast	1qq2A_	192
YBR025C	YBR025C	1	psiblast	1egaA_	204
YBR025C	YBR025C	2	psiblast	1egaA_	109
YBR025C	YBR025C	3	msa	na	81
YBR026C	ETR1	1	psiblast	1qorA_	196
YBR026C	ETR1	2	psiblast	1qorA_	184
YBR027C	YBR027C	1	unassigned	na	110
YBR028C	YBR028C	1	unassigned	na	89
YBR028C	YBR028C	2	psiblast	1ctpE_	296
YBR028C	YBR028C	3	psiblast	1ctpE_	140
YBR029C	CDS1	1	unassigned	na	179
YBR029C	CDS1	2	pfam	PF01148	278
YBR030W	YBR030W	1	msa	na	295
YBR030W	YBR030W	2	msa	na	104
YBR030W	YBR030W	3	unassigned	na	153
YBR031W	RPL4A	1	psiblast	1k5yD_	244
YBR031W	RPL4A	2	psiblast	1k5yD_	56
YBR031W	RPL4A	3	msa	na	62
YBR032W	YBR032W	1	unassigned	na	100
YBR033W	EDS1	1	psiblast	1hwtC_	134
YBR033W	EDS1	2	msa	na	87
YBR033W	EDS1	3	msa	na	172
YBR033W	EDS1	4	unassigned	na	54
YBR033W	EDS1	5	fold_recogniti	1b8fA	360
YBR033W	EDS1	6	unassigned	na	112
YBR034C	HMT1	1	psiblast	1g6q1_	179
YBR034C	HMT1	2	psiblast	1g6q1_	169
YBR035C	PDX3	1	psiblast	1ci0A_	185
YBR035C	PDX3	2	psiblast	1ci0A_	43
YBR036C	CSG2	1	msa	na	96
YBR036C	CSG2	2	unassigned	na	314
YBR037C	SCO1	1	unassigned	na	109
YBR037C	SCO1	2	psiblast	1qq2A_	186
YBR038W	CHS2	1	fold_recogniti	1deqA	184
YBR038W	CHS2	2	unassigned	na	102
YBR038W	CHS2	3	fold_recogniti	1h7lA	267
YBR038W	CHS2	4	fold_recogniti	1h7lA	74
YBR038W	CHS2	5	msa	na	120
YBR038W	CHS2	6	msa	na	216
YBR039W	ATP3	1	psiblast	1bmfG_	311
YBR040W	FIG1	1	unassigned	na	298
YBR041W	FAT1	1	psiblast	1ba3__	360
YBR041W	FAT1	2	psiblast	1ba3__	263
YBR042C	YBR042C	1	fold_recogniti	1k30A	397
YBR043C	QDR3	1	unassigned	na	70
YBR043C	QDR3	2	psiblast	1ja5A_	436

YBR043C	QDR3	3	psiblast	1ja5A_	41
YBR043C	QDR3	4	unassigned	na	142
YBR044C	TCM62	1	psiblast	1iokA_	302
YBR044C	TCM62	2	psiblast	1iokA_	271
YBR045C	GIP1	1	unassigned	na	573
YBR046C	ZTA1	1	psiblast	1qorA_	163
YBR046C	ZTA1	2	psiblast	1qorA_	171
YBR047W	FMP23	1	unassigned	na	175
YBR048W	RPS11B	1	psiblast	1k5xQ_	156
YBR049C	REB1	1	fold_recogniti	1ldvA	248
YBR049C	REB1	2	unassigned	na	125
YBR049C	REB1	3	psiblast	1h88C_	99
YBR049C	REB1	4	psiblast	1h88C_	50
YBR049C	REB1	5	psiblast	1h88C_	58
YBR049C	REB1	6	fold_recogniti	1h88C	77
YBR049C	REB1	7	fold_recogniti	1h88C	63
YBR049C	REB1	8	fold_recogniti	1h88C	90
YBR050C	REG2	1	unassigned	na	338
YBR051W	YBR051W	1	unassigned	na	116
YBR052C	YBR052C	1	psiblast	1qrdA_	210
YBR053C	YBR053C	1	psiblast	1e1aA_	358
YBR054W	YRO2	1	psiblast	1gu8A_	86
YBR054W	YRO2	2	psiblast	1gu8A_	258
YBR055C	PRP6	1	msa	na	123
YBR055C	PRP6	2	fold_recogniti	1qqeA	151
YBR055C	PRP6	3	fold_recogniti	1qqeA	162
YBR055C	PRP6	4	psiblast	1hz4A_	123
YBR055C	PRP6	5	psiblast	1hz4A_	207
YBR055C	PRP6	6	psiblast	1fchA_	133
YBR056W	YBR056W	1	psiblast	1cz1A_	329
YBR056W	YBR056W	2	psiblast	1cz1A_	92
YBR056W	YBR056W	3	unassigned	na	80
YBR056W-A	YBR056W-A	1	unassigned	na	66
YBR057C	MUM2	1	psiblast	1c1gA_	84
YBR057C	MUM2	2	psiblast	1i84S_	282
YBR058C	UBP14	1	unassigned	na	212
YBR058C	UBP14	2	pfam	PF02148	106
YBR058C	UBP14	3	pfam	PF00442	59
YBR058C	UBP14	4	unassigned	na	124
YBR058C	UBP14	5	msa	na	100
YBR058C	UBP14	6	pfam	PF00627	60
YBR058C	UBP14	7	pfam	PF00627	65
YBR058C	UBP14	8	pfam	PF00443	77
YBR058C-A	TSC3	1	unassigned	na	80
YBR059C	AKL1	1	psiblast	1ql6A_	118
YBR059C	AKL1	2	psiblast	1ql6A_	68
YBR059C	AKL1	3	psiblast	1ql6A_	179
YBR059C	AKL1	4	psiblast	1cdmA_	105

YBR059C	AKL1	5	psiblast	1cdmA_	119
YBR059C	AKL1	6	unassigned	na	519
YBR060C	ORC2	1	fold_recogniti	1m2vB	260
YBR060C	ORC2	2	msa	na	360
YBR061C	TRM7	1	psiblast	1eizA_	213
YBR061C	TRM7	2	msa	na	97
YBR062C	YBR062C	1	psiblast	1chc__	115
YBR063C	YBR063C	1	unassigned	na	404
YBR064W	YBR064W	1	unassigned	na	142
YBR065C	ECM2	1	unassigned	na	91
YBR065C	ECM2	2	psiblast	1cvjA_	107
YBR065C	ECM2	3	psiblast	1cvjA_	89
YBR065C	ECM2	4	psiblast	1l3kA_	77
YBR066C	NRG2	1	psiblast	1tf6A_	220
YBR067C	TIP1	1	fold_recogniti	1ldvA	210
YBR068C	BAP2	1	unassigned	na	80
YBR068C	BAP2	2	pfam	PF00324	529
YBR069C	TAT1	1	unassigned	na	55
YBR069C	TAT1	2	pfam	PF00324	564
YBR070C	YBR070C	1	fold_recogniti	1f0kA	237
YBR071W	YBR071W	1	unassigned	na	211
YBR072W	HSP26	1	psiblast	1gmeA_	214
YBR073W	RDH54	1	msa	na	269
YBR073W	RDH54	2	unassigned	na	69
YBR073W	RDH54	3	fold_recogniti	1gm5A	186
YBR073W	RDH54	4	psiblast	1hv8A_	132
YBR073W	RDH54	5	psiblast	1c4oA_	302
YBR074W	YBR074W	1	unassigned	na	52
YBR074W	YBR074W	2	psiblast	1cp7A_	286
YBR074W	YBR074W	3	unassigned	na	75
YBR075W	425	1	msa	na	295
YBR075W	425	2	unassigned	na	165
YBR076W	ECM8	1	unassigned	na	339
YBR077C	YBR077C	1	unassigned	na	162
YBR078W	ECM33	1	psiblast	1dnqA_	91
YBR078W	ECM33	2	psiblast	1jl5A_	377
YBR079C	RPG1	1	fold_recogniti	1hz4A	158
YBR079C	RPG1	2	fold_recogniti	1hz4A	127
YBR079C	RPG1	3	fold_recogniti	1hz4A	166
YBR079C	RPG1	4	unassigned	na	70
YBR079C	RPG1	5	psiblast	1c1gA_	89
YBR079C	RPG1	6	psiblast	1i84S_	354
YBR080C	SEC18	1	psiblast	1cr5A_	103
YBR080C	SEC18	2	psiblast	1cr5A_	115
YBR080C	SEC18	3	psiblast	1hqcA_	192
YBR080C	SEC18	4	fold_recogniti	1e32A	96
YBR080C	SEC18	5	psiblast	1nsf__	184
YBR080C	SEC18	6	psiblast	1nsf__	68

YBR081C	SPT7	1	psiblast	1i84S_	329
YBR081C	SPT7	2	unassigned	na	60
YBR081C	SPT7	3	psiblast	1e6iA_	169
YBR081C	SPT7	4	fold_recogniti	1l8mA	74
YBR081C	SPT7	5	fold_recogniti	1l8mA	79
YBR081C	SPT7	6	fold_recogniti	1l8mA	70
YBR081C	SPT7	7	fold_recogniti	1l8mA	120
YBR081C	SPT7	8	unassigned	na	431
YBR082C	UBC4	1	psiblast	1qcqA_	148
YBR083W	TEC1	1	pfam	PF01285	486
YBR084W	MIS1	1	psiblast	1diaA_	199
YBR084W	MIS1	2	psiblast	1diaA_	137
YBR084W	MIS1	3	psiblast	1fp7A_	380
YBR084W	MIS1	4	psiblast	1fp7A_	259
YBR085C-A	YBR085C-A	1	unassigned	na	85
YBR085W	AAC3	1	pfam	PF00153	112
YBR085W	AAC3	2	pfam	PF00153	101
YBR085W	AAC3	3	pfam	PF00153	94
YBR086C	IST2	1	msa	na	548
YBR086C	IST2	2	fold_recogniti	1i3qA	398
YBR087W	RFC5	1	psiblast	1iqpA_	75
YBR087W	RFC5	2	psiblast	1iqpA_	279
YBR088C	POL30	1	psiblast	1plq__	127
YBR088C	POL30	2	psiblast	1plq__	131
YBR089C-A	NHP6B	1	psiblast	1cg7A_	99
YBR089W	YBR089W	1	unassigned	na	199
YBR090C	YBR090C	1	unassigned	na	122
YBR091C	MRS5	1	pfam	PF02953	109
YBR092C	PHO3	1	psiblast	1qfxA_	168
YBR092C	PHO3	2	psiblast	1qfxA_	299
YBR093C	PHO5	1	psiblast	1qfxA_	168
YBR093C	PHO5	2	psiblast	1qfxA_	299
YBR094W	YBR094W	1	psiblast	1j9jA_	241
YBR094W	YBR094W	2	unassigned	na	133
YBR094W	YBR094W	3	pfam	PF03133	379
YBR095C	RXT2	1	unassigned	na	453
YBR096W	YBR096W	1	msa	na	230
YBR097W	VPS15	1	psiblast	1cdkA_	156
YBR097W	VPS15	2	psiblast	1cdkA_	234
YBR097W	VPS15	3	psiblast	1b3uA_	90
YBR097W	VPS15	4	psiblast	1b3uA_	332
YBR097W	VPS15	5	unassigned	na	241
YBR097W	VPS15	6	psiblast	1gg2B_	120
YBR097W	VPS15	7	fold_recogniti	1k32A	281
YBR098W	MMS4	1	unassigned	na	471
YBR099C	YBR099C	1	unassigned	na	127
YBR100W	453	1	unassigned	na	112
YBR101C	FES1	1	psiblast	1ejlI_	118

YBR101C	FES1	2	psiblast	1ejlI_	56
YBR101C	FES1	3	psiblast	1ejlI_	116
YBR102C	EXO84	1	unassigned	na	157
YBR102C	EXO84	2	msa	na	122
YBR102C	EXO84	3	unassigned	na	62
YBR102C	EXO84	4	psiblast	1awe__	139
YBR102C	EXO84	5	msa	na	273
YBR103C-A	YBR103C-A	1	unassigned	na	47
YBR103W	SIF2	1	msa	na	187
YBR103W	SIF2	2	psiblast	1gg2B_	348
YBR104W	YMC2	1	pfam	PF00153	129
YBR104W	YMC2	2	pfam	PF00153	107
YBR104W	YMC2	3	pfam	PF00153	93
YBR105C	VID24	1	unassigned	na	105
YBR105C	VID24	2	msa	na	257
YBR106W	PHO88	1	unassigned	na	188
YBR107C	IML3	1	unassigned	na	245
YBR108W	YBR108W	1	msa	na	697
YBR108W	YBR108W	2	unassigned	na	151
YBR109C	CMD1	1	psiblast	1iq5A_	77
YBR109C	CMD1	2	psiblast	1iq5A_	70
YBR109W-A	YBR109W-A	1	unassigned	na	74
YBR110W	ALG1	1	psiblast	1f0kA_	224
YBR110W	ALG1	2	psiblast	1f0kA_	166
YBR110W	ALG1	3	unassigned	na	59
YBR111C	YSA1	1	unassigned	na	72
YBR111C	YSA1	2	psiblast	1f3yA_	159
YBR112C	CYC8	1	psiblast	1fchA_	94
YBR112C	CYC8	2	psiblast	1fchA_	70
YBR112C	CYC8	3	psiblast	1fchA_	110
YBR112C	CYC8	4	psiblast	1ft2A_	169
YBR112C	CYC8	5	psiblast	1ft2A_	66
YBR112C	CYC8	6	fold_recogniti	1m2vB	79
YBR112C	CYC8	7	fold_recogniti	1jv2B	246
YBR112C	CYC8	8	fold_recogniti	1jv2B	58
YBR112C	CYC8	9	unassigned	na	74
YBR113W	YBR113W	1	unassigned	na	160
YBR114W	RAD16	1	unassigned	na	82
YBR114W	RAD16	2	psiblast	1gm5A_	51
YBR114W	RAD16	3	psiblast	1gm5A_	235
YBR114W	RAD16	4	unassigned	na	160
YBR114W	RAD16	5	psiblast	1hv8A_	262
YBR115C	LYS2	1	msa	na	131
YBR115C	LYS2	2	unassigned	na	82
YBR115C	LYS2	3	psiblast	1amuA_	229
YBR115C	LYS2	4	psiblast	1amuA_	253
YBR115C	LYS2	5	psiblast	1amuA_	148
YBR115C	LYS2	6	psiblast	1dnyA_	93

YBR115C	LYS2	7	psiblast	1l5aA_	252
YBR115C	LYS2	8	msa	na	204
YBR116C	YBR116C	1	unassigned	na	175
YBR117C	TKL2	1	psiblast	1gpuA_	291
YBR117C	TKL2	2	psiblast	1gpuA_	49
YBR117C	TKL2	3	psiblast	1gpuA_	243
YBR117C	TKL2	4	psiblast	1gpuA_	98
YBR118W	TEF2	1	psiblast	1f60A_	195
YBR118W	TEF2	2	psiblast	1f60A_	137
YBR118W	TEF2	3	psiblast	1f60A_	126
YBR119W	MUD1	1	psiblast	1cvjA_	113
YBR119W	MUD1	2	psiblast	1cvjA_	99
YBR119W	MUD1	3	psiblast	2u1a__	86
YBR120C	CBP6	1	unassigned	na	162
YBR121C	GRS1	1	psiblast	1atiA_	460
YBR121C	GRS1	2	psiblast	1atiA_	131
YBR121C	GRS1	3	psiblast	1qf6A_	76
YBR122C	MRPL36	1	fold_recogniti	1lnrY	196
YBR123C	TFC1	1	msa	na	540
YBR123C	TFC1	2	unassigned	na	109
YBR124W	YBR124W	1	unassigned	na	119
YBR125C	PTC4	1	psiblast	1a6q__	393
YBR126C	TPS1	1	msa	na	115
YBR126C	TPS1	2	fold_recogniti	1f6dA	181
YBR126C	TPS1	3	fold_recogniti	1f6dA	199
YBR126W-A	YBR126W-A	1	unassigned	na	47
YBR127C	VMA2	1	psiblast	1fx0A_	99
YBR127C	VMA2	2	psiblast	1fx0A_	287
YBR127C	VMA2	3	psiblast	1fx0A_	131
YBR128C	ATG14	1	unassigned	na	174
YBR128C	ATG14	2	msa	na	170
YBR129C	OPY1	1	unassigned	na	170
YBR129C	OPY1	2	pfam	PF00169	158
YBR130C	SHE3	1	psiblast	1i84S_	355
YBR130C	SHE3	2	unassigned	na	70
YBR131W	CCZ1	1	unassigned	na	704
YBR132C	AGP2	1	unassigned	na	69
YBR132C	AGP2	2	pfam	PF00324	527
YBR133C	HSL7	1	unassigned	na	291
YBR133C	HSL7	2	psiblast	1f3lA_	205
YBR133C	HSL7	3	psiblast	1f3lA_	134
YBR133C	HSL7	4	unassigned	na	197
YBR134W	YBR134W	1	unassigned	na	133
YBR135W	CKS1	1	msa	na	150
YBR136W	MEC1	1	unassigned	na	555
YBR136W	MEC1	2	msa	na	419
YBR136W	MEC1	3	unassigned	na	267
YBR136W	MEC1	4	msa	na	135

YBR136W	MEC1	5	pfam	PF02259	441
YBR136W	MEC1	6	psiblast	1e8yA_	155
YBR136W	MEC1	7	psiblast	1e8yA_	190
YBR136W	MEC1	8	psiblast	1e8yA_	206
YBR137W	YBR137W	1	unassigned	na	179
YBR138C	YBR138C	1	unassigned	na	524
YBR139W	YBR139W	1	unassigned	na	87
YBR139W	YBR139W	2	psiblast	1ysc__	201
YBR139W	YBR139W	3	psiblast	1ysc__	220
YBR140C	IRA1	1	msa	na	401
YBR140C	IRA1	2	unassigned	na	792
YBR140C	IRA1	3	msa	na	378
YBR140C	IRA1	4	unassigned	na	98
YBR140C	IRA1	5	psiblast	1nf1A_	132
YBR140C	IRA1	6	psiblast	1nf1A_	216
YBR140C	IRA1	7	msa	na	725
YBR140C	IRA1	8	msa	na	350
YBR141C	YBR141C	1	unassigned	na	86
YBR141C	YBR141C	2	msa	na	251
YBR142W	MAK5	1	msa	na	163
YBR142W	MAK5	2	psiblast	1hv8A_	271
YBR142W	MAK5	3	psiblast	1hv8A_	163
YBR142W	MAK5	4	psiblast	1d2mA_	123
YBR142W	MAK5	5	unassigned	na	53
YBR143C	SUP45	1	psiblast	1dt9A_	172
YBR143C	SUP45	2	psiblast	1dt9A_	128
YBR143C	SUP45	3	psiblast	1dt9A_	137
YBR144C	YBR144C	1	unassigned	na	104
YBR145W	ADH5	1	psiblast	1e3jA_	188
YBR145W	ADH5	2	psiblast	1e3jA_	163
YBR146W	MRPS9	1	unassigned	na	140
YBR146W	MRPS9	2	psiblast	1fjgI_	138
YBR147W	YBR147W	1	msa	na	142
YBR147W	YBR147W	2	unassigned	na	154
YBR148W	YSW1	1	fold_recogniti	1iu4A	281
YBR148W	YSW1	2	unassigned	na	328
YBR149W	ARA1	1	psiblast	1llgA_	288
YBR149W	ARA1	2	psiblast	1llgA_	56
YBR150C	TBS1	1	unassigned	na	57
YBR150C	TBS1	2	psiblast	1cld__	121
YBR150C	TBS1	3	unassigned	na	160
YBR150C	TBS1	4	msa	na	286
YBR150C	TBS1	5	unassigned	na	291
YBR150C	TBS1	6	fold_recogniti	1l8mA	60
YBR150C	TBS1	7	fold_recogniti	1l8mA	119
YBR151W	APD1	1	msa	na	241
YBR151W	APD1	2	psiblast	1f37A_	75
YBR152W	SPP381	1	fold_recogniti	1l8mA	67

YBR152W	SPP381	2	fold_recogniti	1l8mA	79
YBR152W	SPP381	3	fold_recogniti	1l8mA	70
YBR152W	SPP381	4	fold_recogniti	1l8mA	75
YBR153W	RIB7	1	psiblast	1cz3A_	244
YBR154C	RPB5	1	psiblast	1dzfA_	144
YBR154C	RPB5	2	psiblast	1dzfA_	71
YBR155W	CNS1	1	psiblast	1ihgA_	134
YBR155W	CNS1	2	psiblast	1ihgA_	153
YBR155W	CNS1	3	unassigned	na	98
YBR156C	SLI15	1	unassigned	na	175
YBR156C	SLI15	2	msa	na	456
YBR156C	SLI15	3	unassigned	na	67
YBR157C	ICS2	1	unassigned	na	106
YBR158W	AMN1	1	unassigned	na	65
YBR158W	AMN1	2	psiblast	1jl5A_	91
YBR158W	AMN1	3	psiblast	2bnh__	77
YBR158W	AMN1	4	psiblast	2bnh__	63
YBR158W	AMN1	5	psiblast	2bnh__	51
YBR158W	AMN1	6	psiblast	2bnh__	202
YBR159W	YBR159W	1	psiblast	1i01A_	347
YBR160W	CDC28	1	psiblast	1aq1__	90
YBR160W	CDC28	2	psiblast	1aq1__	82
YBR160W	CDC28	3	psiblast	1aq1__	51
YBR160W	CDC28	4	psiblast	1aq1__	75
YBR161W	CSH1	1	msa	na	376
YBR162C	TOS1	1	unassigned	na	214
YBR162C	TOS1	2	msa	na	241
YBR162W-A	YSY6	1	unassigned	na	65
YBR163W	DEM1	1	unassigned	na	585
YBR164C	ARL1	1	psiblast	1rrf__	183
YBR165W	UBS1	1	unassigned	na	277
YBR166C	TYR1	1	psiblast	1pgn__	323
YBR166C	TYR1	2	unassigned	na	129
YBR167C	POP7	1	unassigned	na	140
YBR168W	PEX32	1	unassigned	na	174
YBR168W	PEX32	2	msa	na	239
YBR169C	SSE2	1	psiblast	1hjoA_	191
YBR169C	SSE2	2	psiblast	1hjoA_	119
YBR169C	SSE2	3	psiblast	1hjoA_	75
YBR169C	SSE2	4	psiblast	1dkyA_	181
YBR169C	SSE2	5	psiblast	1dkyA_	127
YBR170C	NPL4	1	unassigned	na	208
YBR170C	NPL4	2	msa	na	372
YBR171W	SEC66	1	unassigned	na	206
YBR172C	SMY2	1	unassigned	na	208
YBR172C	SMY2	2	psiblast	1i84S_	342
YBR172C	SMY2	3	msa	na	240
YBR173C	UMP1	1	unassigned	na	148

YBR174C	YBR174C	1	unassigned	na	104
YBR175W	SWD3	1	psiblast	1erjA_	315
YBR176W	ECM31	1	fold_recogniti	1a50A	312
YBR177C	EHT1	1	unassigned	na	109
YBR177C	EHT1	2	psiblast	1cqzA_	342
YBR178W	YBR178W	1	unassigned	na	124
YBR179C	FZO1	1	unassigned	na	128
YBR179C	FZO1	2	psiblast	1jwyB_	415
YBR179C	FZO1	3	msa	na	152
YBR179C	FZO1	4	unassigned	na	160
YBR180W	DTR1	1	unassigned	na	83
YBR180W	DTR1	2	psiblast	1ja5A_	489
YBR181C	RPS6B	1	pfam	PF01092	130
YBR181C	RPS6B	2	msa	na	106
YBR182C	SMP1	1	psiblast	1egwA_	80
YBR182C	SMP1	2	msa	na	197
YBR182C	SMP1	3	unassigned	na	175
YBR183W	YPC1	1	msa	na	186
YBR183W	YPC1	2	msa	na	130
YBR184W	YBR184W	1	unassigned	na	523
YBR185C	MBA1	1	unassigned	na	278
YBR186W	PCH2	1	msa	na	221
YBR186W	PCH2	2	psiblast	1e32A_	246
YBR186W	PCH2	3	psiblast	1e32A_	69
YBR187W	YBR187W	1	pfam	PF01169	127
YBR187W	YBR187W	2	unassigned	na	61
YBR187W	YBR187W	3	pfam	PF01169	92
YBR188C	NTC20	1	unassigned	na	140
YBR189W	RPS9B	1	psiblast	1k5xD_	65
YBR189W	RPS9B	2	psiblast	1k5xD_	130
YBR190W	YBR190W	1	unassigned	na	103
YBR191W	RPL21A	1	psiblast	1k5yQ_	160
YBR191W-A	YBR191W-A	1	unassigned	na	24
YBR192W	RIM2	1	pfam	PF00153	170
YBR192W	RIM2	2	pfam	PF00153	106
YBR192W	RIM2	3	pfam	PF00153	101
YBR193C	MED8	1	unassigned	na	223
YBR194W	SOY1	1	unassigned	na	123
YBR195C	MSI1	1	psiblast	1gxrA_	111
YBR195C	MSI1	2	psiblast	1gg2B_	311
YBR196C	PGI1	1	psiblast	1gzdA_	316
YBR196C	PGI1	2	psiblast	1gzdA_	238
YBR197C	YBR197C	1	unassigned	na	69
YBR197C	YBR197C	2	msa	na	148
YBR198C	TAF5	1	msa	na	165
YBR198C	TAF5	2	unassigned	na	237
YBR198C	TAF5	3	psiblast	1erjA_	396
YBR199W	KTR4	1	pfam	PF01793	464

YBR200W	BEM1	1	unassigned	na	66
YBR200W	BEM1	2	psiblast	2ptk__	67
YBR200W	BEM1	3	psiblast	2ptk__	105
YBR200W	BEM1	4	psiblast	2ptk__	128
YBR200W	BEM1	5	psiblast	1gd5A_	68
YBR200W	BEM1	6	psiblast	1ip9A_	117
YBR201W	DER1	1	msa	na	134
YBR201W	DER1	2	msa	na	77
YBR202W	CDC47	1	msa	na	206
YBR202W	CDC47	2	pfam	PF00493	211
YBR202W	CDC47	3	psiblast	1g8pA_	229
YBR202W	CDC47	4	psiblast	1g8pA_	114
YBR202W	CDC47	5	msa	na	85
YBR203W	COS111	1	unassigned	na	184
YBR203W	COS111	2	pfam	PF00646	113
YBR203W	COS111	3	unassigned	na	627
YBR204C	YBR204C	1	unassigned	na	85
YBR204C	YBR204C	2	pfam	PF00561	290
YBR205W	KTR3	1	pfam	PF01793	404
YBR206W	YBR206W	1	unassigned	na	107
YBR207W	FTH1	1	pfam	PF03239	465
YBR208C	DUR1,2	1	psiblast	1gr8A_	483
YBR208C	DUR1,2	2	unassigned	na	121
YBR208C	DUR1,2	3	psiblast	1bncA_	131
YBR208C	DUR1,2	4	psiblast	1bncA_	145
YBR208C	DUR1,2	5	psiblast	1bncA_	214
YBR208C	DUR1,2	6	pfam	PF02626	323
YBR208C	DUR1,2	7	pfam	PF02682	238
YBR208C	DUR1,2	8	unassigned	na	96
YBR208C	DUR1,2	9	psiblast	1dczA_	84
YBR209W	YBR209W	1	unassigned	na	105
YBR210W	ERV15	1	pfam	PF03311	142
YBR211C	AME1	1	unassigned	na	92
YBR211C	AME1	2	fold_recogniti	1qoyA	232
YBR212W	NGR1	1	psiblast	1cvjA_	86
YBR212W	NGR1	2	psiblast	1fxlA_	98
YBR212W	NGR1	3	psiblast	1fxlA_	122
YBR212W	NGR1	4	unassigned	na	52
YBR212W	NGR1	5	psiblast	1h6kX_	117
YBR212W	NGR1	6	unassigned	na	109
YBR212W	NGR1	7	psiblast	1g9lA_	88
YBR213W	MET8	1	psiblast	1kyqA_	179
YBR213W	MET8	2	psiblast	1kyqA_	95
YBR214W	SDS24	1	unassigned	na	95
YBR214W	SDS24	2	psiblast	1zfjA_	297
YBR214W	SDS24	3	psiblast	1zfjA_	135
YBR215W	HPC2	1	unassigned	na	82
YBR215W	HPC2	2	fold_recogniti	1m2vB	326

YBR215W	HPC2	3	msa	na	215
YBR216C	YBP1	1	fold_recogniti	1bgxT	92
YBR216C	YBP1	2	fold_recogniti	1bgxT	49
YBR216C	YBP1	3	fold_recogniti	1bgxT	481
YBR216C	YBP1	4	fold_recogniti	1bgxT	52
YBR217W	ATG12	1	unassigned	na	74
YBR217W	ATG12	2	fold_recogniti	1kjtA	112
YBR218C	PYC2	1	psiblast	1lsrA_	129
YBR218C	PYC2	2	psiblast	1lsrA_	80
YBR218C	PYC2	3	psiblast	1lsrA_	136
YBR218C	PYC2	4	psiblast	1lsrA_	130
YBR218C	PYC2	5	unassigned	na	61
YBR218C	PYC2	6	fold_recogniti	1lt7A	165
YBR218C	PYC2	7	fold_recogniti	1dhpA	231
YBR218C	PYC2	8	fold_recogniti	1dhpA	60
YBR218C	PYC2	9	unassigned	na	94
YBR218C	PYC2	10	psiblast	1dczA_	94
YBR220C	YBR220C	1	msa	na	244
YBR220C	YBR220C	2	msa	na	207
YBR220C	YBR220C	3	msa	na	109
YBR221C	PDB1	1	psiblast	1ik6A_	231
YBR221C	PDB1	2	psiblast	1ik6A_	135
YBR222C	PCS60	1	psiblast	1ba3__	180
YBR222C	PCS60	2	psiblast	1ba3__	253
YBR222C	PCS60	3	psiblast	1ba3__	110
YBR223C	TDP1	1	psiblast	1jy1A_	222
YBR223C	TDP1	2	psiblast	1jy1A_	46
YBR223C	TDP1	3	psiblast	1jy1A_	45
YBR223C	TDP1	4	psiblast	1jy1A_	231
YBR224W	YBR224W	1	unassigned	na	171
YBR225W	YBR225W	1	unassigned	na	900
YBR226C	YBR226C	1	unassigned	na	136
YBR227C	MCX1	1	psiblast	1g3iA_	520
YBR228W	SLX1	1	msa	na	167
YBR228W	SLX1	2	unassigned	na	137
YBR229C	ROT2	1	unassigned	na	82
YBR229C	ROT2	2	msa	na	308
YBR229C	ROT2	3	psiblast	2taaA_	196
YBR229C	ROT2	4	msa	na	228
YBR229C	ROT2	5	msa	na	140
YBR230C	YBR230C	1	unassigned	na	134
YBR231C	SWC5	1	msa	na	303
YBR232C	YBR232C	1	unassigned	na	119
YBR233W	PBP2	1	psiblast	1j4wA_	143
YBR233W	PBP2	2	psiblast	1j4wA_	100
YBR233W	PBP2	3	unassigned	na	79
YBR233W	PBP2	4	psiblast	1j5kA_	91
YBR233W-A	DAD3	1	unassigned	na	94

YBR234C	ARC40	1	psiblast	1k8kC_	384
YBR235W	YBR235W	1	msa	na	548
YBR235W	YBR235W	2	msa	na	202
YBR235W	YBR235W	3	msa	na	153
YBR235W	YBR235W	4	unassigned	na	217
YBR236C	ABD1	1	unassigned	na	94
YBR236C	ABD1	2	psiblast	1ic3A_	113
YBR236C	ABD1	3	psiblast	1ic3A_	229
YBR237W	PRP5	1	msa	na	218
YBR237W	PRP5	2	psiblast	1fuuA_	60
YBR237W	PRP5	3	psiblast	1gm5A_	271
YBR237W	PRP5	4	psiblast	1gm5A_	197
YBR237W	PRP5	5	fold_recogniti	1k1gA	103
YBR238C	YBR238C	1	fold_recogniti	1l8mA	97
YBR238C	YBR238C	2	fold_recogniti	1l8mA	70
YBR238C	YBR238C	3	fold_recogniti	1l8mA	126
YBR238C	YBR238C	4	unassigned	na	438
YBR239C	YBR239C	1	psiblast	1hwtC_	171
YBR239C	YBR239C	2	unassigned	na	182
YBR239C	YBR239C	3	pfam	PF00989	176
YBR240C	THI2	1	psiblast	1pyiA_	115
YBR240C	THI2	2	unassigned	na	335
YBR241C	YBR241C	1	psiblast	1ja5A_	423
YBR241C	YBR241C	2	psiblast	1ja5A_	65
YBR242W	YBR242W	1	unassigned	na	71
YBR242W	YBR242W	2	pfam	PF01966	167
YBR243C	ALG7	1	unassigned	na	115
YBR243C	ALG7	2	pfam	PF00953	224
YBR243C	ALG7	3	msa	na	109
YBR244W	GPX2	1	psiblast	1gp1A_	162
YBR245C	ISW1	1	msa	na	122
YBR245C	ISW1	2	psiblast	1gm5A_	253
YBR245C	ISW1	3	unassigned	na	58
YBR245C	ISW1	4	psiblast	1hv8A_	185
YBR245C	ISW1	5	msa	na	372
YBR245C	ISW1	6	unassigned	na	139
YBR246W	YBR246W	1	psiblast	1gg2B_	387
YBR247C	ENP1	1	msa	na	182
YBR247C	ENP1	2	msa	na	146
YBR247C	ENP1	3	msa	na	155
YBR248C	HIS7	1	psiblast	1jvnA_	237
YBR248C	HIS7	2	psiblast	1jvnA_	315
YBR249C	ARO4	1	psiblast	1gg1A_	370
YBR250W	YBR250W	1	unassigned	na	523
YBR251W	MRPS5	1	msa	na	136
YBR251W	MRPS5	2	psiblast	1fjgE_	171
YBR252W	DUT1	1	psiblast	1dud__	147
YBR253W	SRB6	1	unassigned	na	121

YBR254C	TRS20	1	unassigned	na	175
YBR255C-A	YBR255C-A	1	unassigned	na	40
YBR255W	YBR255W	1	unassigned	na	331
YBR255W	YBR255W	2	fold_recogniti	1l2qA	280
YBR255W	YBR255W	3	unassigned	na	83
YBR256C	RIB5	1	psiblast	1i8dA_	105
YBR256C	RIB5	2	psiblast	1i8dA_	133
YBR257W	POP4	1	unassigned	na	162
YBR257W	POP4	2	msa	na	117
YBR258C	SHG1	1	unassigned	na	142
YBR259W	YBR259W	1	unassigned	na	688
YBR260C	RGD1	1	psiblast	1i84S_	435
YBR260C	RGD1	2	psiblast	1rgp__	124
YBR260C	RGD1	3	psiblast	1rgp__	107
YBR261C	YBR261C	1	psiblast	1l1eA_	232
YBR262C	FMP51	1	unassigned	na	106
YBR263W	SHM1	1	unassigned	na	92
YBR263W	SHM1	2	psiblast	1cj0A_	200
YBR263W	SHM1	3	psiblast	1cj0A_	273
YBR264C	YPT10	1	psiblast	1huqA_	218
YBR265W	TSC10	1	psiblast	1gcoA_	320
YBR266C	YBR266C	1	unassigned	na	150
YBR267W	YBR267W	1	psiblast	1meyG_	94
YBR267W	YBR267W	2	psiblast	1tf6A_	196
YBR267W	YBR267W	3	unassigned	na	103
YBR268W	MRPL37	1	unassigned	na	105
YBR269C	FMP21	1	psiblast	1khoA_	130
YBR270C	YBR270C	1	unassigned	na	545
YBR271W	YBR271W	1	unassigned	na	89
YBR271W	YBR271W	2	msa	na	103
YBR271W	YBR271W	3	psiblast	1f3lA_	227
YBR272C	HSM3	1	fold_recogniti	1qoyA	360
YBR272C	HSM3	2	unassigned	na	120
YBR273C	UBX7	1	unassigned	na	78
YBR273C	UBX7	2	msa	na	116
YBR273C	UBX7	3	psiblast	1jruA_	129
YBR273C	UBX7	4	unassigned	na	113
YBR274W	CHK1	1	psiblast	1ia8A_	79
YBR274W	CHK1	2	psiblast	1ia8A_	102
YBR274W	CHK1	3	psiblast	1ia8A_	126
YBR274W	CHK1	4	psiblast	1iq5A_	88
YBR274W	CHK1	5	psiblast	1iq5A_	132
YBR275C	RIF1	1	msa	na	1127
YBR275C	RIF1	2	unassigned	na	789
YBR276C	PPS1	1	unassigned	na	446
YBR276C	PPS1	2	msa	na	137
YBR276C	PPS1	3	psiblast	1ikzA_	224
YBR277C	YBR277C	1	unassigned	na	133

YBR278W	DPB3	1	fold_recogniti	1jfiA	109
YBR278W	DPB3	2	unassigned	na	92
YBR279W	PAF1	1	unassigned	na	193
YBR279W	PAF1	2	msa	na	252
YBR280C	YBR280C	1	unassigned	na	287
YBR280C	YBR280C	2	psiblast	1a12A_	355
YBR281C	YBR281C	1	psiblast	1gg2B_	410
YBR281C	YBR281C	2	psiblast	1cg2A_	311
YBR281C	YBR281C	3	psiblast	1cg2A_	157
YBR282W	MRPL27	1	unassigned	na	146
YBR283C	SSH1	1	msa	na	74
YBR283C	SSH1	2	pfam	PF00344	416
YBR284W	YBR284W	1	unassigned	na	213
YBR284W	YBR284W	2	pfam	PF00962	584
YBR285W	YBR285W	1	unassigned	na	144
YBR286W	APE3	1	unassigned	na	56
YBR286W	APE3	2	fold_recogniti	1de4C	82
YBR286W	APE3	3	fold_recogniti	1de4C	86
YBR286W	APE3	4	psiblast	1cp7A_	313
YBR287W	ZSP1	1	msa	na	140
YBR287W	ZSP1	2	unassigned	na	287
YBR288C	APM3	1	psiblast	1gw5M_	166
YBR288C	APM3	2	psiblast	1gw5M_	317
YBR289W	SNF5	1	fold_recogniti	1i3qA	123
YBR289W	SNF5	2	fold_recogniti	1m2vB	415
YBR289W	SNF5	3	unassigned	na	367
YBR290W	BSD2	1	unassigned	na	126
YBR290W	BSD2	2	msa	na	108
YBR290W	BSD2	3	unassigned	na	87
YBR291C	CTP1	1	pfam	PF00153	105
YBR291C	CTP1	2	pfam	PF00153	106
YBR291C	CTP1	3	pfam	PF00153	88
YBR292C	YBR292C	1	unassigned	na	123
YBR293W	YBR293W	1	psiblast	1lviA_	191
YBR293W	YBR293W	2	msa	na	213
YBR293W	YBR293W	3	unassigned	na	70
YBR294W	SUL1	1	unassigned	na	79
YBR294W	SUL1	2	msa	na	119
YBR294W	SUL1	3	pfam	PF00916	374
YBR294W	SUL1	4	unassigned	na	55
YBR294W	SUL1	5	pfam	PF01740	160
YBR294W	SUL1	6	unassigned	na	72
YBR295W	PCA1	1	unassigned	na	313
YBR295W	PCA1	2	psiblast	1aw0__	76
YBR295W	PCA1	3	unassigned	na	67
YBR295W	PCA1	4	psiblast	1kqkA_	106
YBR295W	PCA1	5	psiblast	1eulA_	146
YBR295W	PCA1	6	psiblast	1eulA_	152

YBR295W	PCA1	7	psiblast	1eulA_	77
YBR295W	PCA1	8	psiblast	1f5sA_	64
YBR295W	PCA1	9	psiblast	1f5sA_	153
YBR295W	PCA1	10	msa	na	62
YBR296C	PHO89	1	pfam	PF01384	574
YBR297W	MAL33	1	psiblast	1ajyA_	76
YBR297W	MAL33	2	msa	na	210
YBR297W	MAL33	3	unassigned	na	182
YBR298C	MAL31	1	unassigned	na	76
YBR298C	MAL31	2	psiblast	1ja5A_	423
YBR298C	MAL31	3	psiblast	1ja5A_	115
YBR299W	MAL32	1	psiblast	1uok__	299
YBR299W	MAL32	2	psiblast	1uok__	285
YBR300C	YBR300C	1	unassigned	na	165
YBR301W	DAN3	1	pfam	PF00660	120
YBR302C	COS2	1	msa	na	61
YBR302C	COS2	2	pfam	PF00674	108
YBR302C	COS2	3	unassigned	na	72
YBR302C	COS2	4	pfam	PF00674	138
YCL001W	RER1	1	pfam	PF03248	188
YCL001W-A	YCL001W-A	1	fold_recogniti	1dt9A	153
YCL001W-B	YCL001W-B	1	psiblast	1dt9A_	84
YCL002C	YCL002C	1	unassigned	na	152
YCL004W	PGS1	1	msa	na	68
YCL004W	PGS1	2	psiblast	1f0iA_	453
YCL005W	YCL005W	1	unassigned	na	256
YCL007C	YCL007C	1	unassigned	na	130
YCL008C	STP22	1	psiblast	1kppA_	205
YCL008C	STP22	2	msa	na	91
YCL009C	ILV6	1	fold_recogniti	1in9A	190
YCL009C	ILV6	2	msa	na	119
YCL010C	SGF29	1	msa	na	135
YCL010C	SGF29	2	msa	na	124
YCL011C	GBP2	1	psiblast	1cvjA_	112
YCL011C	GBP2	2	psiblast	1cvjA_	98
YCL011C	GBP2	3	psiblast	1fxlA_	119
YCL011C	GBP2	4	psiblast	2mssA_	98
YCL014W	BUD3	1	unassigned	na	240
YCL014W	BUD3	2	msa	na	395
YCL014W	BUD3	3	msa	na	923
YCL014W	BUD3	4	unassigned	na	78
YCL016C	DCC1	1	msa	na	223
YCL016C	DCC1	2	msa	na	157
YCL017C	NFS1	1	unassigned	na	70
YCL017C	NFS1	2	psiblast	1ecxA_	174
YCL017C	NFS1	3	psiblast	1ecxA_	253
YCL018W	LEU2	1	psiblast	2ayqA_	364
YCL021W-A	YCL021W-A	1	unassigned	na	125

YCL023C	YCL023C	1	unassigned	na	115
YCL024W	KCC4	1	psiblast	1a06__	198
YCL024W	KCC4	2	psiblast	1a06__	198
YCL024W	KCC4	3	unassigned	na	229
YCL024W	KCC4	4	fold_recogniti	1iu4A	293
YCL024W	KCC4	5	unassigned	na	119
YCL025C	AGP1	1	unassigned	na	87
YCL025C	AGP1	2	pfam	PF00324	508
YCL026C-A	FRM2	1	psiblast	1bkjA_	193
YCL026C-B	YCL026C-B	1	unassigned	na	193
YCL027W	FUS1	1	unassigned	na	430
YCL027W	FUS1	2	pfam	PF00018	82
YCL028W	RNQ1	1	fold_recogniti	1i3qA	405
YCL029C	BIK1	1	psiblast	1lplA_	60
YCL029C	BIK1	2	psiblast	1i84S_	380
YCL030C	HIS4	1	msa	na	55
YCL030C	HIS4	2	unassigned	na	65
YCL030C	HIS4	3	pfam	PF01502	123
YCL030C	HIS4	4	pfam	PF01503	101
YCL030C	HIS4	5	psiblast	1k75A_	241
YCL030C	HIS4	6	psiblast	1k75A_	214
YCL031C	RRP7	1	msa	na	152
YCL031C	RRP7	2	msa	na	145
YCL032W	STE50	1	unassigned	na	196
YCL032W	STE50	2	pfam	PF00788	150
YCL033C	YCL033C	1	psiblast	1l1dA_	168
YCL034W	LSB5	1	fold_recogniti	1jwfA	91
YCL034W	LSB5	2	fold_recogniti	1jwfA	77
YCL034W	LSB5	3	unassigned	na	186
YCL035C	GRX1	1	psiblast	1jhb__	110
YCL036W	GFD2	1	unassigned	na	259
YCL036W	GFD2	2	msa	na	248
YCL036W	GFD2	3	unassigned	na	59
YCL037C	SRO9	1	fold_recogniti	1i3qA	279
YCL037C	SRO9	2	msa	na	187
YCL038C	ATG22	1	msa	na	194
YCL038C	ATG22	2	unassigned	na	89
YCL038C	ATG22	3	msa	na	245
YCL039W	GID7	1	unassigned	na	177
YCL039W	GID7	2	psiblast	1gg2B_	393
YCL039W	GID7	3	psiblast	1erjA_	175
YCL040W	GLK1	1	psiblast	1ig8A_	103
YCL040W	GLK1	2	psiblast	1ig8A_	397
YCL041C	YCL041C	1	unassigned	na	164
YCL042W	YCL042W	1	unassigned	na	119
YCL043C	PDI1	1	psiblast	1mek__	142
YCL043C	PDI1	2	psiblast	1bjx__	167
YCL043C	PDI1	3	unassigned	na	61

YCL043C	PDI1	4	psiblast	1t7pB_	152
YCL044C	YCL044C	1	msa	na	215
YCL044C	YCL044C	2	unassigned	na	202
YCL045C	YCL045C	1	unassigned	na	201
YCL045C	YCL045C	2	msa	na	559
YCL046W	YCL046W	1	unassigned	na	107
YCL047C	YCL047C	1	fold_recogniti	1k4kA	258
YCL048W	YCL048W	1	psiblast	1igrA_	166
YCL048W	YCL048W	2	psiblast	1jl5A_	245
YCL048W	YCL048W	3	unassigned	na	52
YCL049C	YCL049C	1	unassigned	na	133
YCL049C	YCL049C	2	msa	na	179
YCL050C	APA1	1	fold_recogniti	1gupA	185
YCL050C	APA1	2	unassigned	na	136
YCL051W	LRE1	1	msa	na	325
YCL051W	LRE1	2	unassigned	na	258
YCL052C	PBN1	1	unassigned	na	174
YCL052C	PBN1	2	msa	na	242
YCL054W	SPB1	1	psiblast	1eizA_	204
YCL054W	SPB1	2	psiblast	1i84S_	328
YCL054W	SPB1	3	psiblast	2tmaA_	97
YCL054W	SPB1	4	psiblast	2tmaA_	84
YCL054W	SPB1	5	msa	na	128
YCL055W	KAR4	1	unassigned	na	58
YCL055W	KAR4	2	msa	na	277
YCL056C	YCL056C	1	unassigned	na	144
YCL057C-A	YCL057C-A	1	unassigned	na	97
YCL057W	PRD1	1	psiblast	1i1iP_	311
YCL057W	PRD1	2	psiblast	1i1iP_	401
YCL058C	FYV5	1	unassigned	na	152
YCL059C	KRR1	1	fold_recogniti	1j4wA	123
YCL059C	KRR1	2	fold_recogniti	1j4wA	110
YCL059C	KRR1	3	unassigned	na	83
YCL061C	MRC1	1	unassigned	na	242
YCL061C	MRC1	2	msa	na	322
YCL061C	MRC1	3	unassigned	na	75
YCL061C	MRC1	4	msa	na	254
YCL061C	MRC1	5	unassigned	na	203
YCL063W	VAC17	1	msa	na	148
YCL063W	VAC17	2	unassigned	na	58
YCL063W	VAC17	3	msa	na	217
YCL064C	CHA1	1	psiblast	1tdj__	239
YCL064C	CHA1	2	psiblast	1tdj__	121
YCL065W	YCL065W	1	unassigned	na	122
YCL066W	HMLALPHA1	1	unassigned	na	175
YCL067C	HMLALPHA2	1	unassigned	na	101
YCL067C	HMLALPHA2	2	psiblast	1mnmC_	109
YCL068C	YCL068C	1	msa	na	260

YCL069W	YCL069W	1	msa	na	336
YCL069W	YCL069W	2	msa	na	122
YCL073C	YCL073C	1	psiblast	1ja5A_	406
YCL073C	YCL073C	2	msa	na	209
YCL074W	YCL074W	1	fold_recogniti	1c9rA	88
YCL074W	YCL074W	2	fold_recogniti	1c9rA	220
YCL075W	YCL075W	1	psiblast	1a6bB_	53
YCL075W	YCL075W	2	msa	na	93
YCL076W	YCL076W	1	msa	na	247
YCR001W	YCR001W	1	unassigned	na	104
YCR002C	CDC10	1	psiblast	1egaA_	244
YCR002C	CDC10	2	msa	na	78
YCR003W	MRPL32	1	unassigned	na	71
YCR003W	MRPL32	2	psiblast	1jzxM_	112
YCR004C	YCP4	1	psiblast	1ag9A_	247
YCR005C	CIT2	1	psiblast	1cts__	356
YCR005C	CIT2	2	psiblast	1cts__	104
YCR006C	YCR006C	1	unassigned	na	157
YCR007C	YCR007C	1	pfam	PF00674	239
YCR008W	SAT4	1	fold_recogniti	1i3qA	226
YCR008W	SAT4	2	unassigned	na	53
YCR008W	SAT4	3	psiblast	1ia8A_	109
YCR008W	SAT4	4	psiblast	1ia8A_	79
YCR008W	SAT4	5	psiblast	1ia8A_	136
YCR009C	RVS161	1	psiblast	1i49A_	265
YCR010C	ADY2	1	pfam	PF01184	283
YCR011C	ADP1	1	fold_recogniti	1bziA	286
YCR011C	ADP1	2	unassigned	na	91
YCR011C	ADP1	3	psiblast	1l2tA_	248
YCR011C	ADP1	4	msa	na	273
YCR011C	ADP1	5	unassigned	na	151
YCR012W	PGK1	1	psiblast	1qpg__	201
YCR012W	PGK1	2	psiblast	1qpg__	215
YCR013C	YCR013C	1	msa	na	215
YCR014C	POL4	1	msa	na	91
YCR014C	POL4	2	unassigned	na	58
YCR014C	POL4	3	psiblast	1jmsA_	115
YCR014C	POL4	4	psiblast	1jmsA_	54
YCR014C	POL4	5	psiblast	1jmsA_	264
YCR015C	YCR015C	1	msa	na	241
YCR015C	YCR015C	2	unassigned	na	76
YCR016W	YCR016W	1	msa	na	226
YCR016W	YCR016W	2	unassigned	na	64
YCR017C	CWH43	1	msa	na	242
YCR017C	CWH43	2	unassigned	na	389
YCR017C	CWH43	3	psiblast	1hd7A_	322
YCR018C	SRD1	1	unassigned	na	139
YCR018C	SRD1	2	psiblast	4gatA_	82

YCR018C-A	YCR018C-A	1	unassigned	na	84
YCR019W	MAK32	1	psiblast	1rk2A_	363
YCR020C	PET18	1	fold_recogniti	1h0nA	132
YCR020C	PET18	2	fold_recogniti	1h0nA	83
YCR020C-A	MAK31	1	pfam	PF01423	88
YCR020W-B	HTL1	1	unassigned	na	78
YCR021C	HSP30	1	psiblast	1c8rA_	332
YCR022C	YCR022C	1	unassigned	na	114
YCR023C	YCR023C	1	psiblast	1lviA_	271
YCR023C	YCR023C	2	unassigned	na	340
YCR024C	YCR024C	1	psiblast	1lkhA_	128
YCR024C	YCR024C	2	psiblast	1lkhA_	364
YCR024C-A	PMP1	1	unassigned	na	40
YCR025C	YCR025C	1	unassigned	na	136
YCR026C	YCR026C	1	msa	na	136
YCR026C	YCR026C	2	psiblast	1e1zP_	357
YCR026C	YCR026C	3	unassigned	na	57
YCR026C	YCR026C	4	msa	na	86
YCR026C	YCR026C	5	unassigned	na	106
YCR027C	RHB1	1	psiblast	1guaA_	209
YCR028C	FEN2	1	psiblast	1ja5A_	472
YCR028C	FEN2	2	psiblast	1ja5A_	40
YCR028C-A	RIM1	1	psiblast	3ullA_	135
YCR030C	SYP1	1	msa	na	150
YCR030C	SYP1	2	unassigned	na	227
YCR030C	SYP1	3	fold_recogniti	1deqA	227
YCR030C	SYP1	4	unassigned	na	126
YCR030C	SYP1	5	msa	na	140
YCR031C	RPS14A	1	psiblast	1k5xK_	137
YCR032W	BPH1	1	unassigned	na	1252
YCR032W	BPH1	2	msa	na	180
YCR032W	BPH1	3	unassigned	na	90
YCR032W	BPH1	4	pfam	PF02138	281
YCR032W	BPH1	5	psiblast	1gg2B_	364
YCR033W	SNT1	1	msa	na	551
YCR033W	SNT1	2	unassigned	na	105
YCR033W	SNT1	3	pfam	PF00249	123
YCR033W	SNT1	4	unassigned	na	96
YCR033W	SNT1	5	pfam	PF00249	93
YCR033W	SNT1	6	unassigned	na	258
YCR034W	FEN1	1	pfam	PF01151	347
YCR035C	RRP43	1	pfam	PF01138	277
YCR035C	RRP43	2	pfam	PF03725	117
YCR036W	RBK1	1	psiblast	1rk2A_	333
YCR037C	PHO87	1	pfam	PF03105	368
YCR037C	PHO87	2	unassigned	na	67
YCR037C	PHO87	3	msa	na	488
YCR038C	BUD5	1	unassigned	na	150

YCR038C	BUD5	2	psiblast	1bkdS_	236
YCR038C	BUD5	3	psiblast	1bkdS_	256
YCR038W-A	YCR038W-A	1	unassigned	na	41
YCR041W	YCR041W	1	unassigned	na	110
YCR042C	TAF2	1	fold_recogniti	1h19A	240
YCR042C	TAF2	2	psiblast	1hs6A_	147
YCR042C	TAF2	3	psiblast	1hs6A_	130
YCR042C	TAF2	4	psiblast	1hs6A_	172
YCR042C	TAF2	5	psiblast	1hs6A_	100
YCR042C	TAF2	6	msa	na	162
YCR042C	TAF2	7	unassigned	na	456
YCR043C	YCR043C	1	unassigned	na	127
YCR044C	PER1	1	unassigned	na	98
YCR044C	PER1	2	msa	na	259
YCR045C	YCR045C	1	psiblast	1scjB_	105
YCR045C	YCR045C	2	psiblast	1ic6A_	320
YCR045C	YCR045C	3	unassigned	na	66
YCR046C	IMG1	1	psiblast	1lnrN_	169
YCR047C	BUD23	1	psiblast	1d2gA_	202
YCR047C	BUD23	2	unassigned	na	73
YCR048W	ARE1	1	unassigned	na	131
YCR048W	ARE1	2	msa	na	184
YCR048W	ARE1	3	unassigned	na	52
YCR048W	ARE1	4	pfam	PF01800	243
YCR049C	YCR049C	1	unassigned	na	148
YCR050C	YCR050C	1	unassigned	na	102
YCR051W	YCR051W	1	psiblast	1bi8B_	69
YCR051W	YCR051W	2	psiblast	1bi8B_	80
YCR051W	YCR051W	3	psiblast	1ycsB_	73
YCR052W	RSC6	1	msa	na	266
YCR052W	RSC6	2	unassigned	na	60
YCR052W	RSC6	3	msa	na	157
YCR053W	THR4	1	psiblast	1kl7A_	146
YCR053W	THR4	2	psiblast	1kl7A_	368
YCR054C	CTR86	1	unassigned	na	418
YCR054C	CTR86	2	msa	na	145
YCR057C	PWP2	1	msa	na	84
YCR057C	PWP2	2	psiblast	1gxrA_	332
YCR057C	PWP2	3	psiblast	1gg2B_	85
YCR057C	PWP2	4	psiblast	1erjA_	175
YCR057C	PWP2	5	unassigned	na	67
YCR057C	PWP2	6	msa	na	180
YCR059C	YIH1	1	msa	na	108
YCR059C	YIH1	2	pfam	PF01205	150
YCR060W	TAH1	1	psiblast	1elwA_	111
YCR061W	YCR061W	1	msa	na	493
YCR061W	YCR061W	2	msa	na	138
YCR063W	BUD31	1	pfam	PF01125	157

YCR064C	YCR064C	1	unassigned	na	136
YCR065W	HCM1	1	unassigned	na	102
YCR065W	HCM1	2	psiblast	2hdcA_	107
YCR065W	HCM1	3	msa	na	289
YCR065W	HCM1	4	unassigned	na	66
YCR066W	RAD18	1	psiblast	1jm7A_	100
YCR066W	RAD18	2	psiblast	1fre__	42
YCR066W	RAD18	3	unassigned	na	124
YCR066W	RAD18	4	pfam	PF02037	77
YCR066W	RAD18	5	unassigned	na	144
YCR067C	SED4	1	psiblast	1scgB_	93
YCR067C	SED4	2	psiblast	1b9xA_	339
YCR067C	SED4	3	fold_recogniti	1m2vB	296
YCR067C	SED4	4	fold_recogniti	1deqA	198
YCR067C	SED4	5	msa	na	139
YCR068W	ATG15	1	unassigned	na	220
YCR068W	ATG15	2	psiblast	1dt3A_	151
YCR068W	ATG15	3	msa	na	149
YCR069W	CPR4	1	psiblast	1dywA_	264
YCR069W	CPR4	2	unassigned	na	54
YCR071C	IMG2	1	unassigned	na	146
YCR072C	YCR072C	1	psiblast	1gxrA_	111
YCR072C	YCR072C	2	psiblast	1gg2B_	319
YCR072C	YCR072C	3	psiblast	1erjA_	85
YCR073C	SSK22	1	unassigned	na	103
YCR073C	SSK22	2	fold_recogniti	1hciA	164
YCR073C	SSK22	3	fold_recogniti	1hciA	111
YCR073C	SSK22	4	fold_recogniti	1hciA	124
YCR073C	SSK22	5	fold_recogniti	1hciA	139
YCR073C	SSK22	6	unassigned	na	201
YCR073C	SSK22	7	psiblast	1f3mA_	100
YCR073C	SSK22	8	unassigned	na	57
YCR073C	SSK22	9	psiblast	1f3mC_	115
YCR073C	SSK22	10	psiblast	1f3mC_	217
YCR073W-A	SOL2	1	psiblast	1d9tA_	315
YCR075C	ERS1	1	unassigned	na	108
YCR075C	ERS1	2	msa	na	152
YCR076C	YCR076C	1	unassigned	na	89
YCR076C	YCR076C	2	fold_recogniti	1deqA	161
YCR077C	PAT1	1	fold_recogniti	1deqA	227
YCR077C	PAT1	2	msa	na	197
YCR077C	PAT1	3	unassigned	na	372
YCR079W	YCR079W	1	unassigned	na	93
YCR079W	YCR079W	2	psiblast	1a6q__	349
YCR081W	SRB8	1	msa	na	340
YCR081W	SRB8	2	unassigned	na	325
YCR081W	SRB8	3	msa	na	394
YCR081W	SRB8	4	unassigned	na	368

YCR082W	AHC2	1	unassigned	na	128
YCR083W	TRX3	1	psiblast	1erv__	127
YCR084C	TUP1	1	fold_recogniti	1ldvA	281
YCR084C	TUP1	2	psiblast	1erjA_	432
YCR085W	YCR085W	1	unassigned	na	117
YCR086W	CSM1	1	unassigned	na	190
YCR087C-A	YCR087C-A	1	unassigned	na	153
YCR087W	YCR087W	1	unassigned	na	171
YCR088W	ABP1	1	psiblast	1hqz1_	170
YCR088W	ABP1	2	fold_recogniti	1ei3A	274
YCR088W	ABP1	3	fold_recogniti	1m2vB	89
YCR088W	ABP1	4	psiblast	1jo8A_	59
YCR089W	FIG2	1	msa	na	174
YCR089W	FIG2	2	unassigned	na	134
YCR089W	FIG2	3	msa	na	317
YCR089W	FIG2	4	msa	na	251
YCR089W	FIG2	5	msa	na	418
YCR089W	FIG2	6	msa	na	315
YCR090C	YCR090C	1	unassigned	na	182
YCR091W	KIN82	1	unassigned	na	175
YCR091W	KIN82	2	psiblast	1qcfA_	125
YCR091W	KIN82	3	psiblast	1qcfA_	91
YCR091W	KIN82	4	psiblast	1qcfA_	84
YCR091W	KIN82	5	psiblast	1qcfA_	111
YCR091W	KIN82	6	psiblast	1ctpE_	79
YCR091W	KIN82	7	unassigned	na	55
YCR092C	MSH3	1	unassigned	na	116
YCR092C	MSH3	2	psiblast	1e3mA_	165
YCR092C	MSH3	3	psiblast	1e3mA_	163
YCR092C	MSH3	4	psiblast	1e3mA_	303
YCR092C	MSH3	5	psiblast	1e3mA_	213
YCR092C	MSH3	6	psiblast	1e3mA_	87
YCR093W	CDC39	1	unassigned	na	527
YCR093W	CDC39	2	msa	na	227
YCR093W	CDC39	3	unassigned	na	126
YCR093W	CDC39	4	msa	na	196
YCR093W	CDC39	5	unassigned	na	275
YCR093W	CDC39	6	msa	na	421
YCR093W	CDC39	7	unassigned	na	336
YCR094W	CDC50	1	pfam	PF03381	391
YCR095C	YCR095C	1	fold_recogniti	1vhrA	226
YCR095C	YCR095C	2	msa	na	136
YCR096C	HMRA2	1	psiblast	1mnmC_	119
YCR097W	HMRA1	1	psiblast	1f43A_	126
YCR097W-A	YCR097W-A	1	unassigned	na	88
YCR098C	GIT1	1	psiblast	1ja5A_	518
YCR099C	YCR099C	1	fold_recogniti	1jv2B	155
YCR100C	YCR100C	1	msa	na	108

YCR100C	YCR100C	2	msa	na	208
YCR101C	YCR101C	1	unassigned	na	182
YCR102C	YCR102C	1	psiblast	1tehA_	224
YCR102C	YCR102C	2	psiblast	1tehA_	144
YCR102W-A	YCR102W-A	1	unassigned	na	65
YCR104W	PAU3	1	pfam	PF00660	124
YCR105W	ADH7	1	psiblast	1hdzA_	224
YCR105W	ADH7	2	psiblast	1hdzA_	137
YCR106W	RDS1	1	psiblast	1hwtD_	100
YCR106W	RDS1	2	msa	na	414
YCR106W	RDS1	3	unassigned	na	318
YCR107W	AAD3	1	psiblast	1gveA_	270
YCR107W	AAD3	2	psiblast	1gveA_	93
YDL001W	RMD1	1	unassigned	na	126
YDL001W	RMD1	2	pfam	PF02582	304
YDL002C	NHP10	1	psiblast	1aab__	77
YDL002C	NHP10	2	psiblast	1cg7A_	126
YDL003W	MCD1	1	msa	na	210
YDL003W	MCD1	2	unassigned	na	356
YDL004W	ATP16	1	psiblast	1e79H_	117
YDL004W	ATP16	2	psiblast	1e79H_	43
YDL005C	MED2	1	unassigned	na	132
YDL005C	MED2	2	fold_recogniti	1l8mA	74
YDL005C	MED2	3	fold_recogniti	1l8mA	80
YDL005C	MED2	4	fold_recogniti	1l8mA	70
YDL005C	MED2	5	fold_recogniti	1l8mA	75
YDL006W	PTC1	1	psiblast	1a6q__	281
YDL007W	RPT2	1	psiblast	1e32A_	178
YDL007W	RPT2	2	psiblast	1e32A_	174
YDL007W	RPT2	3	psiblast	1e32A_	85
YDL008W	APC11	1	psiblast	1ldjB_	165
YDL009C	YDL009C	1	unassigned	na	107
YDL010W	YDL010W	1	psiblast	1h4oA_	105
YDL010W	YDL010W	2	psiblast	1kte__	126
YDL011C	YDL011C	1	unassigned	na	107
YDL012C	YDL012C	1	unassigned	na	107
YDL013W	HEX3	1	unassigned	na	619
YDL014W	NOP1	1	fold_recogniti	1la5A	83
YDL014W	NOP1	2	psiblast	1fbnA_	244
YDL015C	TSC13	1	msa	na	116
YDL015C	TSC13	2	pfam	PF02544	194
YDL016C	YDL016C	1	unassigned	na	100
YDL017W	CDC7	1	psiblast	1aq1__	122
YDL017W	CDC7	2	psiblast	1aq1__	78
YDL017W	CDC7	3	psiblast	1aq1__	52
YDL017W	CDC7	4	psiblast	1aq1__	52
YDL017W	CDC7	5	msa	na	118
YDL017W	CDC7	6	unassigned	na	85

YDL018C	ERP3	1	pfam	PF01105	225
YDL019C	OSH2	1	psiblast	1k1aA_	135
YDL019C	OSH2	2	psiblast	1k1aA_	138
YDL019C	OSH2	3	fold_recogniti	1faoA	128
YDL019C	OSH2	4	unassigned	na	141
YDL019C	OSH2	5	psiblast	1i84S_	346
YDL019C	OSH2	6	msa	na	395
YDL020C	RPN4	1	msa	na	305
YDL020C	RPN4	2	psiblast	1meyG_	72
YDL020C	RPN4	3	psiblast	1tf3A_	69
YDL020C	RPN4	4	psiblast	1a1iA_	85
YDL021W	GPM2	1	psiblast	1bq3D_	311
YDL022W	GPD1	1	psiblast	1evyA_	254
YDL022W	GPD1	2	psiblast	1evyA_	137
YDL023C	SRF4	1	unassigned	na	106
YDL024C	DIA3	1	psiblast	1qfxA_	169
YDL024C	DIA3	2	psiblast	1qfxA_	299
YDL025C	YDL025C	1	unassigned	na	88
YDL025C	YDL025C	2	psiblast	1f3mA_	80
YDL025C	YDL025C	3	unassigned	na	106
YDL025C	YDL025C	4	psiblast	1ia8A_	97
YDL025C	YDL025C	5	psiblast	1ia8A_	79
YDL025C	YDL025C	6	psiblast	1ia8A_	170
YDL026W	YDL026W	1	unassigned	na	103
YDL027C	YDL027C	1	unassigned	na	204
YDL027C	YDL027C	2	msa	na	216
YDL028C	MPS1	1	unassigned	na	189
YDL028C	MPS1	2	psiblast	1f3mA_	125
YDL028C	MPS1	3	unassigned	na	96
YDL028C	MPS1	4	psiblast	1ctpE_	98
YDL028C	MPS1	5	psiblast	1ctpE_	256
YDL029W	ARP2	1	psiblast	2btfA_	179
YDL029W	ARP2	2	psiblast	2btfA_	212
YDL030W	PRP9	1	msa	na	212
YDL030W	PRP9	2	unassigned	na	318
YDL031W	DBP10	1	unassigned	na	99
YDL031W	DBP10	2	psiblast	1hv8A_	248
YDL031W	DBP10	3	psiblast	1hv8A_	133
YDL031W	DBP10	4	fold_recogniti	1gkuB	97
YDL031W	DBP10	5	unassigned	na	88
YDL031W	DBP10	6	msa	na	234
YDL031W	DBP10	7	unassigned	na	96
YDL032W	YDL032W	1	unassigned	na	103
YDL033C	YDL033C	1	psiblast	1gpmA_	283
YDL033C	YDL033C	2	msa	na	134
YDL034W	YDL034W	1	unassigned	na	114
YDL035C	GPR1	1	unassigned	na	169
YDL035C	GPR1	2	psiblast	1epwA_	512

YDL035C	GPR1	3	unassigned	na	280
YDL036C	YDL036C	1	psiblast	1fjgD_	127
YDL036C	YDL036C	2	psiblast	1kskA_	66
YDL036C	YDL036C	3	psiblast	1kskA_	187
YDL036C	YDL036C	4	msa	na	82
YDL037C	BSC1	1	unassigned	na	72
YDL037C	BSC1	2	fold_recogniti	1m2vB	256
YDL038C	YDL038C	1	fold_recogniti	1m2vB	278
YDL038C	YDL038C	2	msa	na	71
YDL038C	YDL038C	3	fold_recogniti	1deqA	234
YDL039C	PRM7	1	unassigned	na	115
YDL040C	NAT1	1	psiblast	1fchA_	173
YDL040C	NAT1	2	psiblast	1fchA_	82
YDL040C	NAT1	3	psiblast	1fchA_	87
YDL040C	NAT1	4	msa	na	171
YDL040C	NAT1	5	unassigned	na	66
YDL040C	NAT1	6	msa	na	275
YDL041W	YDL041W	1	unassigned	na	117
YDL042C	SIR2	1	unassigned	na	191
YDL042C	SIR2	2	psiblast	1j8fA_	245
YDL042C	SIR2	3	psiblast	1j8fA_	126
YDL043C	PRP11	1	unassigned	na	121
YDL043C	PRP11	2	msa	na	145
YDL044C	MTF2	1	unassigned	na	440
YDL045C	FAD1	1	psiblast	1sur__	229
YDL045C	FAD1	2	unassigned	na	77
YDL045W-A	MRP10	1	unassigned	na	95
YDL046W	NPC2	1	unassigned	na	173
YDL047W	SIT4	1	psiblast	1fjmA_	311
YDL048C	STP4	1	msa	na	125
YDL048C	STP4	2	msa	na	112
YDL048C	STP4	3	psiblast	1meyC_	114
YDL048C	STP4	4	psiblast	1a1fA_	139
YDL049C	KNH1	1	unassigned	na	268
YDL050C	YDL050C	1	unassigned	na	123
YDL051W	LHP1	1	psiblast	1cvjA_	107
YDL051W	LHP1	2	psiblast	1cvjA_	168
YDL052C	SLC1	1	psiblast	1k30A_	217
YDL052C	SLC1	2	unassigned	na	86
YDL053C	PBP4	1	unassigned	na	185
YDL054C	MCH1	1	psiblast	1ja5A_	442
YDL054C	MCH1	2	psiblast	1ja5A_	44
YDL055C	PSA1	1	psiblast	1h5rB_	276
YDL055C	PSA1	2	psiblast	1qq0A_	85
YDL056W	MBP1	1	psiblast	1mb1__	131
YDL056W	MBP1	2	msa	na	174
YDL056W	MBP1	3	psiblast	1bu9A_	80
YDL056W	MBP1	4	psiblast	1sw6A_	170

YDL056W	MBP1	5	psiblast	1dcqA_	65
YDL056W	MBP1	6	msa	na	213
YDL057W	YDL057W	1	psiblast	1a8s__	328
YDL058W	USO1	1	fold_recogniti	1jpwA	186
YDL058W	USO1	2	fold_recogniti	1jpwA	102
YDL058W	USO1	3	fold_recogniti	1jpwA	84
YDL058W	USO1	4	psiblast	1i84S_	256
YDL058W	USO1	5	psiblast	1i84S_	385
YDL058W	USO1	6	psiblast	2tmaA_	111
YDL058W	USO1	7	psiblast	2tmaA_	83
YDL058W	USO1	8	psiblast	2tmaA_	62
YDL058W	USO1	9	psiblast	2tmaA_	80
YDL058W	USO1	10	fold_recogniti	1i84S	125
YDL058W	USO1	11	fold_recogniti	1i84S	316
YDL059C	RAD59	1	msa	na	173
YDL059C	RAD59	2	unassigned	na	65
YDL060W	TSR1	1	unassigned	na	79
YDL060W	TSR1	2	fold_recogniti	1d2eA	231
YDL060W	TSR1	3	msa	na	201
YDL060W	TSR1	4	msa	na	277
YDL061C	RPS29B	1	pfam	PF00253	56
YDL062W	YDL062W	1	unassigned	na	141
YDL063C	YDL063C	1	fold_recogniti	1qgkA	203
YDL063C	YDL063C	2	fold_recogniti	1qgkA	109
YDL063C	YDL063C	3	fold_recogniti	1qgkA	308
YDL064W	UBC9	1	psiblast	1a3s__	157
YDL065C	PEX19	1	msa	na	209
YDL065C	PEX19	2	unassigned	na	141
YDL066W	IDP1	1	psiblast	1a05A_	428
YDL067C	COX9	1	unassigned	na	59
YDL068W	YDL068W	1	unassigned	na	109
YDL069C	CBS1	1	unassigned	na	233
YDL070W	BDF2	1	unassigned	na	121
YDL070W	BDF2	2	pfam	PF00439	121
YDL070W	BDF2	3	unassigned	na	73
YDL070W	BDF2	4	psiblast	1e6iA_	130
YDL070W	BDF2	5	unassigned	na	57
YDL070W	BDF2	6	msa	na	136
YDL071C	YDL071C	1	unassigned	na	124
YDL072C	YET3	1	msa	na	203
YDL073W	YDL073W	1	unassigned	na	984
YDL074C	BRE1	1	psiblast	1i84S_	342
YDL074C	BRE1	2	unassigned	na	58
YDL074C	BRE1	3	psiblast	1c1gA_	70
YDL074C	BRE1	4	psiblast	1c1gA_	230
YDL075W	RPL31A	1	psiblast	1k5yW_	113
YDL076C	RXT3	1	unassigned	na	294
YDL077C	VAM6	1	unassigned	na	420

YDL077C	VAM6	2	msa	na	124
YDL077C	VAM6	3	psiblast	1b89A_	315
YDL077C	VAM6	4	msa	na	115
YDL077C	VAM6	5	msa	na	75
YDL078C	MDH3	1	psiblast	1mldA_	343
YDL079C	MRK1	1	unassigned	na	90
YDL079C	MRK1	2	psiblast	1i09A_	151
YDL079C	MRK1	3	psiblast	1i09A_	260
YDL080C	THI3	1	psiblast	1qpbA_	199
YDL080C	THI3	2	psiblast	1qpbA_	165
YDL080C	THI3	3	psiblast	1qpbA_	245
YDL081C	RPP1A	1	pfam	PF00428	106
YDL082W	RPL13A	1	pfam	PF01294	199
YDL083C	RPS16B	1	psiblast	1k5xI_	143
YDL084W	SUB2	1	psiblast	1fuuA_	446
YDL085C-A	YDL085C-A	1	unassigned	na	68
YDL085W	NDE2	1	unassigned	na	87
YDL085W	NDE2	2	psiblast	1dxlA_	201
YDL085W	NDE2	3	psiblast	1dxlA_	257
YDL086W	YDL086W	1	psiblast	1qfsA_	273
YDL087C	LUC7	1	pfam	PF03194	261
YDL088C	ASM4	1	msa	na	226
YDL088C	ASM4	2	msa	na	199
YDL088C	ASM4	3	unassigned	na	103
YDL089W	YDL089W	1	unassigned	na	484
YDL090C	RAM1	1	msa	na	342
YDL090C	RAM1	2	unassigned	na	89
YDL091C	UBX3	1	unassigned	na	124
YDL091C	UBX3	2	msa	na	164
YDL091C	UBX3	3	unassigned	na	68
YDL091C	UBX3	4	psiblast	1h8cA_	99
YDL092W	SRP14	1	fold_recogniti	1e8oB	146
YDL093W	PMT5	1	pfam	PF02366	317
YDL093W	PMT5	2	pfam	PF02815	61
YDL093W	PMT5	3	pfam	PF02815	74
YDL093W	PMT5	4	pfam	PF02815	73
YDL093W	PMT5	5	msa	na	218
YDL094C	YDL094C	1	unassigned	na	169
YDL095W	PMT1	1	pfam	PF02366	319
YDL095W	PMT1	2	pfam	PF02815	65
YDL095W	PMT1	3	pfam	PF02815	73
YDL095W	PMT1	4	pfam	PF02815	90
YDL095W	PMT1	5	unassigned	na	55
YDL095W	PMT1	6	msa	na	141
YDL095W	PMT1	7	unassigned	na	74
YDL096C	YDL096C	1	unassigned	na	108
YDL097C	RPN6	1	fold_recogniti	1hz4A	141
YDL097C	RPN6	2	fold_recogniti	1hz4A	122

YDL097C	RPN6	3	fold_recogniti	1hz4A	171
YDL098C	SNU23	1	unassigned	na	194
YDL099W	YDL099W	1	psiblast	1i84S_	99
YDL099W	YDL099W	2	psiblast	1dg3A_	242
YDL100C	ARR4	1	psiblast	1f48A_	354
YDL101C	DUN1	1	psiblast	1gxcA_	152
YDL101C	DUN1	2	psiblast	1a06__	148
YDL101C	DUN1	3	psiblast	1a06__	213
YDL102W	CDC2	1	unassigned	na	75
YDL102W	CDC2	2	psiblast	1tgoA_	410
YDL102W	CDC2	3	psiblast	1tgoA_	225
YDL102W	CDC2	4	psiblast	1tgoA_	123
YDL102W	CDC2	5	psiblast	1tgoA_	174
YDL102W	CDC2	6	unassigned	na	90
YDL103C	QRI1	1	unassigned	na	80
YDL103C	QRI1	2	fold_recogniti	1hm0A	283
YDL103C	QRI1	3	msa	na	114
YDL104C	QRI7	1	unassigned	na	75
YDL104C	QRI7	2	fold_recogniti	1jceA	332
YDL105W	QRI2	1	msa	na	122
YDL105W	QRI2	2	msa	na	280
YDL106C	PHO2	1	psiblast	1fjlA_	138
YDL106C	PHO2	2	fold_recogniti	1ruxA	169
YDL106C	PHO2	3	fold_recogniti	1ruxA	252
YDL107W	MSS2	1	unassigned	na	351
YDL108W	KIN28	1	psiblast	1lg3A_	85
YDL108W	KIN28	2	psiblast	1lg3A_	221
YDL109C	YDL109C	1	unassigned	na	154
YDL109C	YDL109C	2	psiblast	1hqdA_	163
YDL109C	YDL109C	3	msa	na	164
YDL109C	YDL109C	4	unassigned	na	166
YDL110C	YDL110C	1	unassigned	na	150
YDL111C	RRP42	1	psiblast	1e3pA_	119
YDL111C	RRP42	2	psiblast	1e3pA_	146
YDL112W	TRM3	1	unassigned	na	1178
YDL112W	TRM3	2	psiblast	1ipaA_	99
YDL112W	TRM3	3	psiblast	1ipaA_	159
YDL113C	ATG20	1	msa	na	157
YDL113C	ATG20	2	psiblast	1i84S_	361
YDL113C	ATG20	3	msa	na	122
YDL114W	YDL114W	1	psiblast	1i01A_	308
YDL114W-A	YDL114W-A	1	unassigned	na	37
YDL115C	IWR1	1	unassigned	na	240
YDL116W	NUP84	1	unassigned	na	56
YDL116W	NUP84	2	msa	na	445
YDL116W	NUP84	3	unassigned	na	225
YDL117W	CYK3	1	psiblast	1k9aA_	90
YDL117W	CYK3	2	msa	na	369

YDL117W	CYK3	3	fold_recogniti	1e2tA	162
YDL117W	CYK3	4	unassigned	na	264
YDL118W	YDL118W	1	unassigned	na	126
YDL119C	YDL119C	1	pfam	PF00153	105
YDL119C	YDL119C	2	pfam	PF00153	108
YDL119C	YDL119C	3	pfam	PF00153	94
YDL120W	YFH1	1	psiblast	1ekgA_	174
YDL121C	YDL121C	1	unassigned	na	149
YDL122W	UBP1	1	unassigned	na	81
YDL122W	UBP1	2	pfam	PF00442	66
YDL122W	UBP1	3	msa	na	372
YDL122W	UBP1	4	msa	na	154
YDL122W	UBP1	5	pfam	PF00443	69
YDL122W	UBP1	6	unassigned	na	67
YDL123W	SNA4	1	pfam	PF01679	80
YDL123W	SNA4	2	unassigned	na	60
YDL124W	YDL124W	1	psiblast	2alr__	312
YDL125C	HNT1	1	psiblast	1av5A_	158
YDL126C	CDC48	1	psiblast	1e32A_	117
YDL126C	CDC48	2	psiblast	1e32A_	93
YDL126C	CDC48	3	psiblast	1e32A_	171
YDL126C	CDC48	4	psiblast	1e32A_	78
YDL126C	CDC48	5	psiblast	1d2nA_	182
YDL126C	CDC48	6	fold_recogniti	1d2nA	84
YDL126C	CDC48	7	msa	na	110
YDL127W	PCL2	1	psiblast	1vin__	155
YDL127W	PCL2	2	psiblast	1vin__	124
YDL128W	VCX1	1	msa	na	238
YDL128W	VCX1	2	pfam	PF01699	173
YDL129W	YDL129W	1	unassigned	na	291
YDL130W	RPP1B	1	pfam	PF00428	106
YDL130W-A	STF1	1	unassigned	na	86
YDL131W	LYS21	1	fold_recogniti	1euaA	328
YDL131W	LYS21	2	unassigned	na	112
YDL132W	CDC53	1	psiblast	1ldjA_	129
YDL132W	CDC53	2	psiblast	1ldjA_	182
YDL132W	CDC53	3	psiblast	1ldjA_	215
YDL132W	CDC53	4	psiblast	1ldjA_	76
YDL132W	CDC53	5	psiblast	1ldjA_	140
YDL132W	CDC53	6	psiblast	1ldjA_	73
YDL133C-A	RPL41B	1	unassigned	na	25
YDL133W	YDL133W	1	unassigned	na	142
YDL133W	YDL133W	2	msa	na	295
YDL134C	PPH21	1	psiblast	1it6A_	369
YDL135C	RDI1	1	psiblast	1doaB_	202
YDL136W	RPL35B	1	psiblast	1k5yY_	120
YDL137W	ARF2	1	psiblast	1rrf__	181
YDL138W	RGT2	1	unassigned	na	72

YDL138W	RGT2	2	psiblast	1ja5A_	428
YDL138W	RGT2	3	psiblast	1ja5A_	119
YDL138W	RGT2	4	unassigned	na	144
YDL139C	SCM3	1	unassigned	na	247
YDL140C	RPO21	1	psiblast	1k83A_	1555
YDL140C	RPO21	2	fold_recogniti	1m2vB	178
YDL141W	BPL1	1	msa	na	263
YDL141W	BPL1	2	unassigned	na	85
YDL141W	BPL1	3	psiblast	1bia__	274
YDL141W	BPL1	4	psiblast	1bia__	68
YDL142C	CRD1	1	unassigned	na	58
YDL142C	CRD1	2	pfam	PF01066	225
YDL143W	CCT4	1	psiblast	1a6dA_	413
YDL143W	CCT4	2	psiblast	1a6dA_	115
YDL144C	YDL144C	1	psiblast	1ks9A_	214
YDL144C	YDL144C	2	psiblast	1ks9A_	145
YDL145C	COP1	1	psiblast	1erjA_	324
YDL145C	COP1	2	psiblast	1gxrA_	119
YDL145C	COP1	3	msa	na	203
YDL145C	COP1	4	fold_recogniti	1b89A	146
YDL145C	COP1	5	unassigned	na	188
YDL145C	COP1	6	fold_recogniti	1elrA	108
YDL145C	COP1	7	fold_recogniti	1elrA	52
YDL145C	COP1	8	unassigned	na	61
YDL146W	YDL146W	1	unassigned	na	164
YDL146W	YDL146W	2	msa	na	251
YDL146W	YDL146W	3	unassigned	na	76
YDL147W	RPN5	1	fold_recogniti	1hz4A	101
YDL147W	RPN5	2	fold_recogniti	1hz4A	121
YDL147W	RPN5	3	fold_recogniti	1hz4A	148
YDL147W	RPN5	4	msa	na	75
YDL148C	NOP14	1	msa	na	415
YDL148C	NOP14	2	unassigned	na	395
YDL149W	ATG9	1	unassigned	na	229
YDL149W	ATG9	2	msa	na	627
YDL149W	ATG9	3	unassigned	na	141
YDL150W	RPC53	1	unassigned	na	232
YDL150W	RPC53	2	msa	na	190
YDL151C	BUD30	1	unassigned	na	193
YDL152W	YDL152W	1	unassigned	na	121
YDL153C	SAS10	1	psiblast	1i84S_	329
YDL153C	SAS10	2	msa	na	281
YDL154W	MSH5	1	psiblast	1e3mA_	244
YDL154W	MSH5	2	psiblast	1e3mA_	351
YDL154W	MSH5	3	psiblast	1e3mA_	214
YDL154W	MSH5	4	psiblast	1e3mA_	92
YDL155W	CLB3	1	msa	na	162
YDL155W	CLB3	2	psiblast	1vin__	143

YDL155W	CLB3	3	psiblast	1vin__	122
YDL156W	YDL156W	1	unassigned	na	93
YDL156W	YDL156W	2	psiblast	1erjA_	429
YDL157C	YDL157C	1	unassigned	na	118
YDL158C	YDL158C	1	unassigned	na	102
YDL159W	STE7	1	unassigned	na	131
YDL159W	STE7	2	psiblast	1f3mC_	142
YDL159W	STE7	3	psiblast	1f3mC_	242
YDL159W-A	YDL159W-A	1	unassigned	na	43
YDL160C	DHH1	1	psiblast	1fuuA_	417
YDL160C	DHH1	2	psiblast	1d9xA_	89
YDL161W	ENT1	1	psiblast	1inzA_	155
YDL161W	ENT1	2	msa	na	299
YDL162C	YDL162C	1	unassigned	na	118
YDL163W	YDL163W	1	unassigned	na	100
YDL164C	CDC9	1	fold_recogniti	1ldvA	143
YDL164C	CDC9	2	fold_recogniti	1lshA	187
YDL164C	CDC9	3	unassigned	na	61
YDL164C	CDC9	4	psiblast	1a0i__	80
YDL164C	CDC9	5	psiblast	1a0i__	284
YDL165W	CDC36	1	unassigned	na	191
YDL166C	FAP7	1	psiblast	2ak3A_	197
YDL167C	NRP1	1	unassigned	na	173
YDL167C	NRP1	2	psiblast	1fxlA_	144
YDL167C	NRP1	3	msa	na	146
YDL167C	NRP1	4	msa	na	119
YDL167C	NRP1	5	unassigned	na	137
YDL168W	SFA1	1	psiblast	1tehA_	238
YDL168W	SFA1	2	psiblast	1tehA_	148
YDL169C	UGX2	1	unassigned	na	223
YDL170W	UGA3	1	psiblast	1pyiA_	58
YDL170W	UGA3	2	unassigned	na	394
YDL170W	UGA3	3	msa	na	76
YDL171C	GLT1	1	psiblast	1llwA_	499
YDL171C	GLT1	2	psiblast	1llwA_	357
YDL171C	GLT1	3	psiblast	1llwA_	436
YDL171C	GLT1	4	psiblast	1llwA_	316
YDL171C	GLT1	5	psiblast	1gt8A_	537
YDL172C	YDL172C	1	unassigned	na	159
YDL173W	YDL173W	1	unassigned	na	295
YDL174C	DLD1	1	unassigned	na	100
YDL174C	DLD1	2	psiblast	1diiA_	280
YDL174C	DLD1	3	psiblast	1diiA_	207
YDL175C	AIR2	1	psiblast	1baj__	66
YDL175C	AIR2	2	psiblast	1aaf__	54
YDL175C	AIR2	3	unassigned	na	52
YDL175C	AIR2	4	msa	na	106
YDL175C	AIR2	5	unassigned	na	66

YDL176W	YDL176W	1	unassigned	na	708
YDL177C	YDL177C	1	pfam	PF01205	170
YDL178W	DLD2	1	psiblast	1diiA_	318
YDL178W	DLD2	2	psiblast	1diiA_	212
YDL179W	PCL9	1	psiblast	1vin__	181
YDL179W	PCL9	2	psiblast	1vin__	123
YDL180W	YDL180W	1	unassigned	na	547
YDL181W	INH1	1	unassigned	na	85
YDL182W	LYS20	1	fold_recogniti	1euaA	261
YDL182W	LYS20	2	unassigned	na	167
YDL183C	YDL183C	1	msa	na	263
YDL183C	YDL183C	2	unassigned	na	57
YDL184W-B	1025	1	fold_recogniti	1bmfA	79
YDL184W-B	1025	2	psiblast	1skyE_	94
YDL184W-B	1025	3	psiblast	1skyE_	268
YDL184W-B	1025	4	psiblast	1skyE_	176
YDL185C-A	YDL185C-A	1	unassigned	na	75
YDL186W	YDL186W	1	unassigned	na	277
YDL187C	YDL187C	1	unassigned	na	109
YDL188C	PPH22	1	psiblast	1it6A_	377
YDL189W	RBS1	1	pfam	PF01424	120
YDL189W	RBS1	2	unassigned	na	105
YDL189W	RBS1	3	fold_recogniti	1i3qA	263
YDL190C	UFD2	1	msa	na	196
YDL190C	UFD2	2	unassigned	na	473
YDL190C	UFD2	3	psiblast	1i84S_	217
YDL190C	UFD2	4	psiblast	1jm7B_	75
YDL192W	ARF1	1	psiblast	1rrf__	181
YDL193W	YDL193W	1	unassigned	na	78
YDL193W	YDL193W	2	psiblast	1f75A_	297
YDL194W	SNF3	1	unassigned	na	86
YDL194W	SNF3	2	psiblast	1ja5A_	411
YDL194W	SNF3	3	psiblast	1ja5A_	120
YDL194W	SNF3	4	unassigned	na	267
YDL195W	SEC31	1	psiblast	1gg2B_	373
YDL195W	SEC31	2	msa	na	133
YDL195W	SEC31	3	msa	na	187
YDL195W	SEC31	4	msa	na	209
YDL195W	SEC31	5	fold_recogniti	1deqA	99
YDL195W	SEC31	6	fold_recogniti	1ei3A	272
YDL196W	YDL196W	1	fold_recogniti	1oczP	109
YDL197C	ASF2	1	psiblast	1i84S_	366
YDL197C	ASF2	2	msa	na	159
YDL198C	GGC1	1	fold_recogniti	1is2A	300
YDL199C	YDL199C	1	unassigned	na	85
YDL199C	YDL199C	2	psiblast	1ja5A_	428
YDL199C	YDL199C	3	psiblast	1ja5A_	110
YDL199C	YDL199C	4	unassigned	na	64

YDL200C	MGT1	1	psiblast	1sfe__	114
YDL200C	MGT1	2	psiblast	1sfe__	92
YDL201W	TRM8	1	unassigned	na	72
YDL201W	TRM8	2	psiblast	1j4fA_	214
YDL202W	MRPL11	1	psiblast	1jj2G_	249
YDL203C	YDL203C	1	msa	na	145
YDL203C	YDL203C	2	psiblast	1fchA_	216
YDL203C	YDL203C	3	psiblast	1fchA_	120
YDL203C	YDL203C	4	psiblast	1ihgA_	142
YDL204W	RTN2	1	pfam	PF02453	249
YDL204W	RTN2	2	unassigned	na	144
YDL205C	HEM3	1	psiblast	1pda__	127
YDL205C	HEM3	2	psiblast	1pda__	200
YDL206W	YDL206W	1	pfam	PF01699	225
YDL206W	YDL206W	2	msa	na	112
YDL206W	YDL206W	3	unassigned	na	246
YDL206W	YDL206W	4	pfam	PF01699	179
YDL207W	GLE1	1	psiblast	1i84S_	317
YDL207W	GLE1	2	unassigned	na	221
YDL208W	NHP2	1	psiblast	1e7kA_	173
YDL209C	CWC2	1	psiblast	3sxlA_	122
YDL209C	CWC2	2	psiblast	1ha1__	102
YDL209C	CWC2	3	psiblast	1ha1__	115
YDL210W	UGA4	1	pfam	PF00324	571
YDL211C	YDL211C	1	msa	na	278
YDL211C	YDL211C	2	unassigned	na	94
YDL212W	SHR3	1	unassigned	na	210
YDL213C	NOP6	1	fold_recogniti	1fj7A	225
YDL214C	PRR2	1	unassigned	na	320
YDL214C	PRR2	2	psiblast	1aq1__	122
YDL214C	PRR2	3	psiblast	1aq1__	127
YDL214C	PRR2	4	psiblast	1aq1__	51
YDL214C	PRR2	5	psiblast	1aq1__	79
YDL215C	GDH2	1	msa	na	377
YDL215C	GDH2	2	unassigned	na	94
YDL215C	GDH2	3	psiblast	1euzA_	313
YDL215C	GDH2	4	psiblast	1euzA_	231
YDL215C	GDH2	5	unassigned	na	77
YDL216C	RRI1	1	unassigned	na	54
YDL216C	RRI1	2	pfam	PF01398	162
YDL216C	RRI1	3	msa	na	152
YDL216C	RRI1	4	unassigned	na	87
YDL217C	TIM22	1	pfam	PF02466	207
YDL218W	YDL218W	1	msa	na	205
YDL218W	YDL218W	2	unassigned	na	112
YDL219W	DTD1	1	psiblast	1jkeA_	150
YDL220C	CDC13	1	unassigned	na	490
YDL220C	CDC13	2	psiblast	1kxlA_	151

YDL220C	CDC13	3	psiblast	1kxlA_	103
YDL220C	CDC13	4	unassigned	na	180
YDL221W	YDL221W	1	unassigned	na	183
YDL222C	FMP45	1	unassigned	na	309
YDL223C	HBT1	1	msa	na	556
YDL223C	HBT1	2	unassigned	na	490
YDL224C	WHI4	1	unassigned	na	326
YDL224C	WHI4	2	msa	na	121
YDL224C	WHI4	3	unassigned	na	64
YDL224C	WHI4	4	psiblast	2u1a__	138
YDL225W	SHS1	1	fold_recogniti	1h65A	223
YDL225W	SHS1	2	psiblast	1i84S_	328
YDL226C	GCS1	1	psiblast	1dcqA_	201
YDL226C	GCS1	2	unassigned	na	53
YDL226C	GCS1	3	msa	na	98
YDL227C	HO	1	psiblast	1ef0A_	214
YDL227C	HO	2	psiblast	1ef0A_	257
YDL227C	HO	3	unassigned	na	115
YDL228C	YDL228C	1	msa	na	213
YDL229W	SSB1	1	psiblast	1hx1A_	196
YDL229W	SSB1	2	psiblast	1hx1A_	116
YDL229W	SSB1	3	psiblast	1hx1A_	75
YDL229W	SSB1	4	psiblast	1ckrA_	160
YDL229W	SSB1	5	psiblast	1i84S_	66
YDL230W	PTP1	1	psiblast	1larA_	335
YDL231C	BRE4	1	unassigned	na	103
YDL231C	BRE4	2	msa	na	238
YDL231C	BRE4	3	unassigned	na	784
YDL232W	OST4	1	unassigned	na	36
YDL233W	YDL233W	1	fold_recogniti	1m2vB	192
YDL233W	YDL233W	2	unassigned	na	266
YDL234C	GYP7	1	msa	na	250
YDL234C	GYP7	2	unassigned	na	102
YDL234C	GYP7	3	psiblast	1fkmA_	227
YDL234C	GYP7	4	psiblast	1fkmA_	53
YDL234C	GYP7	5	msa	na	114
YDL235C	YPD1	1	psiblast	1c03A_	167
YDL236W	PHO13	1	msa	na	80
YDL236W	PHO13	2	psiblast	1jud__	87
YDL236W	PHO13	3	psiblast	1jud__	145
YDL237W	YDL237W	1	unassigned	na	390
YDL238C	YDL238C	1	psiblast	1ubpC_	68
YDL238C	YDL238C	2	psiblast	1ubpC_	421
YDL239C	ADY3	1	psiblast	1i84S_	347
YDL239C	ADY3	2	msa	na	151
YDL239C	ADY3	3	psiblast	2tmaA_	56
YDL239C	ADY3	4	psiblast	2tmaA_	83
YDL239C	ADY3	5	psiblast	2tmaA_	62

YDL239C	ADY3	6	psiblast	2tmaA_	91
YDL240C-A	YDL240C-A	1	unassigned	na	45
YDL240W	LRG1	1	psiblast	1b8tA_	93
YDL240W	LRG1	2	psiblast	1b8tA_	117
YDL240W	LRG1	3	msa	na	88
YDL240W	LRG1	4	unassigned	na	112
YDL240W	LRG1	5	pfam	PF00412	72
YDL240W	LRG1	6	msa	na	171
YDL240W	LRG1	7	psiblast	1am4A_	168
YDL240W	LRG1	8	psiblast	1am4A_	142
YDL240W	LRG1	9	unassigned	na	54
YDL241W	YDL241W	1	unassigned	na	123
YDL242W	YDL242W	1	unassigned	na	117
YDL243C	AAD4	1	psiblast	1gveB_	329
YDL244W	THI13	1	psiblast	1gggA_	190
YDL244W	THI13	2	psiblast	1gggA_	98
YDL244W	THI13	3	unassigned	na	52
YDL245C	HXT15	1	psiblast	1ja5A_	465
YDL245C	HXT15	2	psiblast	1ja5A_	102
YDL246C	SOR2	1	psiblast	1e3jA_	188
YDL246C	SOR2	2	psiblast	1e3jA_	169
YDL247W	MPH2	1	unassigned	na	68
YDL247W	MPH2	2	psiblast	1ja5A_	429
YDL247W	MPH2	3	psiblast	1ja5A_	112
YDL247W-A	YDL247W-A	1	unassigned	na	24
YDL248W	COS7	1	msa	na	61
YDL248W	COS7	2	pfam	PF00674	112
YDL248W	COS7	3	unassigned	na	71
YDL248W	COS7	4	pfam	PF00674	139
YDR001C	NTH1	1	unassigned	na	90
YDR001C	NTH1	2	pfam	PF01204	661
YDR002W	YRB1	1	psiblast	1rrpB_	201
YDR003W	YDR003W	1	unassigned	na	210
YDR004W	RAD57	1	psiblast	1b22A_	61
YDR004W	RAD57	2	psiblast	1rea__	298
YDR004W	RAD57	3	psiblast	1rea__	101
YDR005C	MAF1	1	unassigned	na	217
YDR005C	MAF1	2	msa	na	178
YDR006C	SOK1	1	unassigned	na	347
YDR006C	SOK1	2	fold_recogniti	1ldjA	369
YDR006C	SOK1	3	fold_recogniti	1ldjA	96
YDR006C	SOK1	4	unassigned	na	89
YDR007W	TRP1	1	psiblast	1dl3A_	66
YDR008C	YDR008C	1	unassigned	na	116
YDR009W	GAL3	1	psiblast	1kkhA_	179
YDR009W	GAL3	2	psiblast	1kkhA_	193
YDR009W	GAL3	3	fold_recogniti	1kvkA	148
YDR010C	YDR010C	1	unassigned	na	110

YDR011W	SNQ2	1	unassigned	na	150
YDR011W	SNQ2	2	psiblast	1l2tA_	282
YDR011W	SNQ2	3	unassigned	na	66
YDR011W	SNQ2	4	psiblast	1jsqA_	315
YDR011W	SNQ2	5	psiblast	1jsqA_	288
YDR011W	SNQ2	6	msa	na	291
YDR011W	SNQ2	7	unassigned	na	109
YDR012W	RPL4B	1	psiblast	1k5yD_	244
YDR012W	RPL4B	2	psiblast	1k5yD_	56
YDR012W	RPL4B	3	msa	na	62
YDR013W	PSF1	1	pfam	PF03651	208
YDR014W	RAD61	1	unassigned	na	74
YDR014W	RAD61	2	msa	na	280
YDR014W	RAD61	3	unassigned	na	293
YDR015C	YDR015C	1	unassigned	na	129
YDR016C	DAD1	1	unassigned	na	94
YDR017C	KCS1	1	unassigned	na	594
YDR017C	KCS1	2	pfam	PF03770	392
YDR017C	KCS1	3	unassigned	na	64
YDR018C	YDR018C	1	unassigned	na	102
YDR018C	YDR018C	2	psiblast	1k30A_	145
YDR018C	YDR018C	3	msa	na	149
YDR019C	GCV1	1	pfam	PF01571	400
YDR020C	YDR020C	1	psiblast	1a7j__	232
YDR021W	FAL1	1	psiblast	1fuuA_	399
YDR022C	CIS1	1	unassigned	na	196
YDR023W	SES1	1	psiblast	1serA_	462
YDR024W	FYV1	1	unassigned	na	161
YDR026C	YDR026C	1	msa	na	128
YDR026C	YDR026C	2	unassigned	na	146
YDR026C	YDR026C	3	psiblast	1h88C_	68
YDR026C	YDR026C	4	psiblast	1h88C_	50
YDR026C	YDR026C	5	psiblast	1h88C_	81
YDR026C	YDR026C	6	psiblast	1idy__	97
YDR027C	VPS54	1	fold_recogniti	3btaA	189
YDR027C	VPS54	2	fold_recogniti	3btaA	56
YDR027C	VPS54	3	fold_recogniti	1hciA	172
YDR027C	VPS54	4	fold_recogniti	1hciA	139
YDR027C	VPS54	5	fold_recogniti	1hciA	137
YDR027C	VPS54	6	msa	na	196
YDR028C	REG1	1	unassigned	na	111
YDR028C	REG1	2	msa	na	243
YDR028C	REG1	3	unassigned	na	420
YDR028C	REG1	4	msa	na	240
YDR029W	YDR029W	1	unassigned	na	104
YDR030C	RAD28	1	psiblast	1gxrA_	317
YDR030C	RAD28	2	psiblast	1gg2B_	88
YDR030C	RAD28	3	msa	na	101

YDR031W	YDR031W	1	unassigned	na	117
YDR032C	PST2	1	psiblast	1ag9A_	198
YDR033W	MRH1	1	psiblast	1gu8A_	86
YDR033W	MRH1	2	psiblast	1gu8A_	234
YDR034C	LYS14	1	msa	na	152
YDR034C	LYS14	2	pfam	PF00172	81
YDR034C	LYS14	3	unassigned	na	557
YDR034C-A	YDR034C-A	1	unassigned	na	58
YDR034W-B	YDR034W-B	1	unassigned	na	51
YDR035W	ARO3	1	psiblast	1gg1A_	370
YDR036C	EHD3	1	psiblast	1dubA_	247
YDR036C	EHD3	2	psiblast	1dubA_	98
YDR036C	EHD3	3	psiblast	3hdhA_	88
YDR036C	EHD3	4	unassigned	na	67
YDR037W	KRS1	1	psiblast	1e1oA_	220
YDR037W	KRS1	2	psiblast	1e1oA_	371
YDR038C	ENA5	1	psiblast	1eulA_	187
YDR038C	ENA5	2	psiblast	1eulA_	423
YDR038C	ENA5	3	psiblast	1eulA_	207
YDR038C	ENA5	4	psiblast	1eulA_	274
YDR039C	ENA2	1	psiblast	1eulA_	187
YDR039C	ENA2	2	psiblast	1eulA_	423
YDR039C	ENA2	3	psiblast	1eulA_	207
YDR039C	ENA2	4	psiblast	1eulA_	274
YDR040C	ENA1	1	psiblast	1eulA_	187
YDR040C	ENA1	2	psiblast	1eulA_	423
YDR040C	ENA1	3	psiblast	1eulA_	207
YDR040C	ENA1	4	psiblast	1eulA_	274
YDR041W	RSM10	1	psiblast	1fjgJ_	203
YDR042C	YDR042C	1	unassigned	na	200
YDR043C	NRG1	1	msa	na	127
YDR043C	NRG1	2	psiblast	1lu6A_	104
YDR044W	HEM13	1	pfam	PF01218	328
YDR045C	RPC11	1	pfam	PF02150	56
YDR045C	RPC11	2	psiblast	1qyp__	54
YDR046C	BAP3	1	unassigned	na	74
YDR046C	BAP3	2	pfam	PF00324	530
YDR047W	HEM12	1	psiblast	1uroA_	362
YDR048C	YDR048C	1	unassigned	na	104
YDR049W	YDR049W	1	msa	na	187
YDR049W	YDR049W	2	unassigned	na	118
YDR049W	YDR049W	3	psiblast	1nfiE_	157
YDR049W	YDR049W	4	psiblast	1nfiE_	80
YDR049W	YDR049W	5	unassigned	na	90
YDR050C	TPI1	1	psiblast	7timA_	248
YDR051C	YDR051C	1	fold_recogniti	1fztA	334
YDR052C	DBF4	1	msa	na	358
YDR052C	DBF4	2	fold_recogniti	1ldvA	236

YDR052C	DBF4	3	unassigned	na	110
YDR053W	YDR053W	1	unassigned	na	131
YDR054C	CDC34	1	psiblast	2ucz__	220
YDR054C	CDC34	2	unassigned	na	75
YDR055W	PST1	1	psiblast	1h6uA_	126
YDR055W	PST1	2	psiblast	1jl5A_	237
YDR055W	PST1	3	unassigned	na	81
YDR056C	YDR056C	1	unassigned	na	205
YDR057W	YOS9	1	msa	na	265
YDR057W	YOS9	2	unassigned	na	277
YDR058C	TGL2	1	psiblast	1tahB_	263
YDR058C	TGL2	2	psiblast	1tahB_	63
YDR059C	UBC5	1	psiblast	1qcqA_	148
YDR060W	MAK21	1	unassigned	na	213
YDR060W	MAK21	2	pfam	PF03914	709
YDR060W	MAK21	3	unassigned	na	103
YDR061W	YDR061W	1	psiblast	1g6hA_	296
YDR061W	YDR061W	2	psiblast	1jj7A_	243
YDR062W	LCB2	1	unassigned	na	99
YDR062W	LCB2	2	psiblast	1fc4A_	198
YDR062W	LCB2	3	psiblast	1fc4A_	264
YDR063W	YDR063W	1	psiblast	1f7sA_	149
YDR064W	RPS13	1	psiblast	1k5xO_	151
YDR065W	YDR065W	1	unassigned	na	365
YDR066C	YDR066C	1	unassigned	na	196
YDR067C	YDR067C	1	psiblast	1rpmA_	224
YDR068W	DOS2	1	unassigned	na	165
YDR068W	DOS2	2	pfam	PF03909	91
YDR068W	DOS2	3	unassigned	na	54
YDR069C	DOA4	1	unassigned	na	184
YDR069C	DOA4	2	psiblast	1d5vA_	151
YDR069C	DOA4	3	fold_recogniti	1ldvA	226
YDR069C	DOA4	4	msa	na	192
YDR069C	DOA4	5	msa	na	173
YDR070C	FMP16	1	unassigned	na	93
YDR071C	PAA1	1	psiblast	1kuvA_	191
YDR072C	IPT1	1	unassigned	na	212
YDR072C	IPT1	2	pfam	PF01569	315
YDR073W	SNF11	1	unassigned	na	169
YDR074W	TPS2	1	unassigned	na	120
YDR074W	TPS2	2	fold_recogniti	1f6dA	219
YDR074W	TPS2	3	fold_recogniti	1f6dA	206
YDR074W	TPS2	4	msa	na	351
YDR075W	PPH3	1	psiblast	1fjmA_	308
YDR076W	RAD55	1	psiblast	2recA_	262
YDR076W	RAD55	2	psiblast	2recA_	74
YDR076W	RAD55	3	unassigned	na	70
YDR077W	SED1	1	fold_recogniti	1m2vB	338

YDR078C	SHU2	1	unassigned	na	223
YDR079C-A	YDR079C-A	1	unassigned	na	72
YDR079W	PET100	1	unassigned	na	111
YDR080W	VPS41	1	psiblast	1erjA_	375
YDR080W	VPS41	2	psiblast	1gg2B_	103
YDR080W	VPS41	3	fold_recogniti	1b89A	514
YDR081C	PDC2	1	psiblast	1iufA_	68
YDR081C	PDC2	2	psiblast	1iufA_	80
YDR081C	PDC2	3	pfam	PF03184	284
YDR081C	PDC2	4	msa	na	372
YDR081C	PDC2	5	unassigned	na	121
YDR082W	STN1	1	unassigned	na	494
YDR083W	RRP8	1	unassigned	na	127
YDR083W	RRP8	2	psiblast	1ic3A_	143
YDR083W	RRP8	3	psiblast	1ic3A_	132
YDR084C	TVP23	1	unassigned	na	199
YDR085C	AFR1	1	unassigned	na	620
YDR086C	SSS1	1	pfam	PF00584	80
YDR087C	RRP1	1	unassigned	na	88
YDR087C	RRP1	2	msa	na	190
YDR088C	SLU7	1	msa	na	174
YDR088C	SLU7	2	unassigned	na	208
YDR089W	YDR089W	1	pfam	PF03105	167
YDR089W	YDR089W	2	unassigned	na	81
YDR089W	YDR089W	3	msa	na	621
YDR090C	YDR090C	1	msa	na	256
YDR090C	YDR090C	2	unassigned	na	54
YDR091C	RLI1	1	fold_recogniti	1kqfB	73
YDR091C	RLI1	2	psiblast	1g291_	294
YDR091C	RLI1	3	psiblast	1l7vC_	241
YDR092W	UBC13	1	psiblast	1jbbA_	153
YDR093W	DNF2	1	unassigned	na	172
YDR093W	DNF2	2	msa	na	147
YDR093W	DNF2	3	psiblast	1eulA_	196
YDR093W	DNF2	4	psiblast	1eulA_	430
YDR093W	DNF2	5	psiblast	1eulA_	225
YDR093W	DNF2	6	psiblast	1eulA_	290
YDR093W	DNF2	7	unassigned	na	152
YDR094W	YDR094W	1	unassigned	na	111
YDR095C	YDR095C	1	unassigned	na	136
YDR096W	GIS1	1	pfam	PF02375	79
YDR096W	GIS1	2	unassigned	na	57
YDR096W	GIS1	3	pfam	PF02373	171
YDR096W	GIS1	4	msa	na	88
YDR096W	GIS1	5	unassigned	na	70
YDR096W	GIS1	6	psiblast	2gliA_	150
YDR096W	GIS1	7	psiblast	1a1iA_	79
YDR096W	GIS1	8	msa	na	81

YDR096W	GIS1	9	psiblast	2drpA_	73
YDR096W	GIS1	10	psiblast	2adr__	46
YDR097C	MSH6	1	fold_recogniti	1ldvA	248
YDR097C	MSH6	2	fold_recogniti	1k83A	55
YDR097C	MSH6	3	psiblast	1e3mA_	114
YDR097C	MSH6	4	psiblast	1e3mA_	194
YDR097C	MSH6	5	psiblast	1e3mA_	300
YDR097C	MSH6	6	psiblast	1e3mA_	205
YDR097C	MSH6	7	psiblast	1e3mA_	74
YDR097C	MSH6	8	unassigned	na	52
YDR098C	GRX3	1	psiblast	1erv__	147
YDR098C	GRX3	2	psiblast	1bjx__	138
YDR099W	BMH2	1	psiblast	1a37A_	115
YDR099W	BMH2	2	psiblast	1a37A_	158
YDR100W	TVP15	1	unassigned	na	143
YDR101C	ARX1	1	psiblast	4matA_	355
YDR101C	ARX1	2	unassigned	na	238
YDR102C	YDR102C	1	unassigned	na	110
YDR103W	STE5	1	unassigned	na	917
YDR104C	SPO71	1	unassigned	na	996
YDR104C	SPO71	2	pfam	PF00169	249
YDR105C	TMS1	1	pfam	PF03348	473
YDR106W	ARP10	1	psiblast	1hluA_	99
YDR106W	ARP10	2	psiblast	1hluA_	185
YDR107C	YDR107C	1	pfam	PF02990	672
YDR108W	GSG1	1	unassigned	na	327
YDR108W	GSG1	2	msa	na	173
YDR108W	GSG1	3	unassigned	na	198
YDR109C	YDR109C	1	psiblast	1bwfY_	335
YDR109C	YDR109C	2	psiblast	1bwfY_	77
YDR109C	YDR109C	3	psiblast	1bwfY_	213
YDR109C	YDR109C	4	unassigned	na	90
YDR110W	FOB1	1	unassigned	na	566
YDR111C	ALT2	1	psiblast	1iaxA_	187
YDR111C	ALT2	2	psiblast	1iaxA_	320
YDR112W	YDR112W	1	unassigned	na	102
YDR113C	PDS1	1	unassigned	na	373
YDR114C	YDR114C	1	unassigned	na	100
YDR115W	YDR115W	1	psiblast	1lnr2_	105
YDR116C	MRPL1	1	psiblast	1eg0N_	201
YDR116C	MRPL1	2	psiblast	1eg0N_	84
YDR117C	YDR117C	1	psiblast	1k8wA_	59
YDR117C	YDR117C	2	psiblast	1it7A_	134
YDR117C	YDR117C	3	unassigned	na	243
YDR117C	YDR117C	4	fold_recogniti	1d1rA	129
YDR118W	APC4	1	unassigned	na	155
YDR118W	APC4	2	fold_recogniti	1qoyA	326
YDR118W	APC4	3	fold_recogniti	1qoyA	40

YDR118W	APC4	4	unassigned	na	131
YDR119W	YDR119W	1	unassigned	na	74
YDR119W	YDR119W	2	msa	na	170
YDR119W	YDR119W	3	psiblast	1ja5A_	307
YDR119W	YDR119W	4	psiblast	1ja5A_	81
YDR119W	YDR119W	5	psiblast	1kplA_	136
YDR120C	TRM1	1	psiblast	1jg1A_	264
YDR120C	TRM1	2	msa	na	306
YDR121W	DPB4	1	psiblast	1jfiB_	101
YDR121W	DPB4	2	psiblast	1jfiB_	95
YDR122W	KIN1	1	psiblast	1dsyA_	90
YDR122W	KIN1	2	psiblast	1a06__	138
YDR122W	KIN1	3	psiblast	1a06__	238
YDR122W	KIN1	4	unassigned	na	87
YDR122W	KIN1	5	fold_recogniti	1i3qA	384
YDR122W	KIN1	6	msa	na	127
YDR123C	INO2	1	unassigned	na	214
YDR123C	INO2	2	pfam	PF00010	90
YDR124W	YDR124W	1	unassigned	na	63
YDR124W	YDR124W	2	msa	na	102
YDR124W	YDR124W	3	unassigned	na	159
YDR125C	ECM18	1	unassigned	na	77
YDR125C	ECM18	2	psiblast	1cqzA_	376
YDR126W	SWF1	1	unassigned	na	118
YDR126W	SWF1	2	pfam	PF01529	88
YDR126W	SWF1	3	msa	na	130
YDR127W	ARO1	1	psiblast	1dqsA_	177
YDR127W	ARO1	2	psiblast	1dqsA_	75
YDR127W	ARO1	3	psiblast	1dqsA_	149
YDR127W	ARO1	4	psiblast	1ln5A_	111
YDR127W	ARO1	5	psiblast	1ln5A_	368
YDR127W	ARO1	6	psiblast	1shkA_	177
YDR127W	ARO1	7	psiblast	1qfeA_	122
YDR127W	ARO1	8	psiblast	1qfeA_	125
YDR127W	ARO1	9	pfam	PF01488	116
YDR127W	ARO1	10	psiblast	1gpjA_	168
YDR128W	YDR128W	1	psiblast	1gg2B_	346
YDR128W	YDR128W	2	msa	na	139
YDR128W	YDR128W	3	unassigned	na	77
YDR128W	YDR128W	4	msa	na	270
YDR128W	YDR128W	5	unassigned	na	231
YDR128W	YDR128W	6	msa	na	85
YDR129C	SAC6	1	psiblast	1a29__	121
YDR129C	SAC6	2	psiblast	1aoa__	140
YDR129C	SAC6	3	psiblast	1aoa__	130
YDR129C	SAC6	4	psiblast	1g8xA_	137
YDR129C	SAC6	5	fold_recogniti	1bkrA	114
YDR130C	FIN1	1	unassigned	na	291

YDR131C	YDR131C	1	unassigned	na	556
YDR132C	YDR132C	1	fold_recogniti	1qdvA	86
YDR132C	YDR132C	2	fold_recogniti	1qdvA	96
YDR132C	YDR132C	3	unassigned	na	313
YDR133C	YDR133C	1	unassigned	na	111
YDR134C	YDR134C	1	unassigned	na	66
YDR135C	YCF1	1	msa	na	267
YDR135C	YCF1	2	pfam	PF00664	342
YDR135C	YCF1	3	psiblast	1nbd__	256
YDR135C	YCF1	4	unassigned	na	69
YDR135C	YCF1	5	psiblast	1jsqA_	296
YDR135C	YCF1	6	psiblast	1jsqA_	285
YDR136C	VPS61	1	unassigned	na	190
YDR137W	RGP1	1	unassigned	na	187
YDR137W	RGP1	2	msa	na	163
YDR137W	RGP1	3	unassigned	na	105
YDR137W	RGP1	4	msa	na	208
YDR138W	HPR1	1	unassigned	na	752
YDR139C	RUB1	1	psiblast	1nddA_	77
YDR140W	YDR140W	1	psiblast	1aqjA_	221
YDR141C	DOP1	1	msa	na	235
YDR141C	DOP1	2	unassigned	na	657
YDR141C	DOP1	3	msa	na	806
YDR142C	PEX7	1	psiblast	1gg2B_	375
YDR143C	SAN1	1	msa	na	135
YDR143C	SAN1	2	unassigned	na	77
YDR143C	SAN1	3	psiblast	1chc__	123
YDR143C	SAN1	4	unassigned	na	52
YDR143C	SAN1	5	msa	na	223
YDR144C	MKC7	1	psiblast	1lcgA_	144
YDR144C	MKC7	2	psiblast	1ypsA_	132
YDR144C	MKC7	3	psiblast	1ypsA_	80
YDR144C	MKC7	4	psiblast	1ypsA_	139
YDR144C	MKC7	5	unassigned	na	101
YDR145W	TAF12	1	unassigned	na	232
YDR145W	TAF12	2	msa	na	173
YDR145W	TAF12	3	fold_recogniti	1h3oB	134
YDR146C	SWI5	1	unassigned	na	74
YDR146C	SWI5	2	msa	na	108
YDR146C	SWI5	3	msa	na	116
YDR146C	SWI5	4	psiblast	1bbo__	61
YDR146C	SWI5	5	msa	na	118
YDR146C	SWI5	6	psiblast	2gliA_	107
YDR146C	SWI5	7	psiblast	1meyG_	125
YDR147W	EKI1	1	unassigned	na	98
YDR147W	EKI1	2	psiblast	1j7iA_	94
YDR147W	EKI1	3	psiblast	1j7iA_	255
YDR147W	EKI1	4	unassigned	na	87

YDR148C	KGD2	1	unassigned	na	70
YDR148C	KGD2	2	psiblast	1lab__	112
YDR148C	KGD2	3	psiblast	2pdd__	49
YDR148C	KGD2	4	psiblast	1c4tA_	232
YDR149C	YDR149C	1	unassigned	na	235
YDR151C	CTH1	1	fold_recogniti	1k83A	325
YDR152W	GIR2	1	msa	na	112
YDR152W	GIR2	2	msa	na	153
YDR153C	ENT5	1	psiblast	1eyhA_	219
YDR153C	ENT5	2	psiblast	1deqA_	192
YDR154C	YDR154C	1	unassigned	na	116
YDR155C	CPR1	1	psiblast	1ak4A_	162
YDR156W	RPA14	1	unassigned	na	137
YDR157W	YDR157W	1	unassigned	na	133
YDR158W	HOM2	1	psiblast	1gpdG_	171
YDR158W	HOM2	2	psiblast	1gpdG_	194
YDR159W	SAC3	1	unassigned	na	187
YDR159W	SAC3	2	pfam	PF03399	364
YDR159W	SAC3	3	unassigned	na	58
YDR159W	SAC3	4	psiblast	1i84S_	224
YDR159W	SAC3	5	unassigned	na	468
YDR160W	SSY1	1	unassigned	na	231
YDR160W	SSY1	2	pfam	PF00324	621
YDR161W	YDR161W	1	fold_recogniti	1qsaA	127
YDR161W	YDR161W	2	fold_recogniti	1qsaA	90
YDR161W	YDR161W	3	fold_recogniti	1qsaA	170
YDR162C	NBP2	1	unassigned	na	103
YDR162C	NBP2	2	psiblast	1qlyA_	63
YDR162C	NBP2	3	psiblast	1aotF_	70
YDR163W	CWC15	1	unassigned	na	175
YDR164C	SEC1	1	psiblast	1fvfA_	132
YDR164C	SEC1	2	psiblast	1fvfA_	276
YDR164C	SEC1	3	psiblast	1fvfA_	120
YDR164C	SEC1	4	psiblast	1fvfA_	129
YDR164C	SEC1	5	unassigned	na	67
YDR165W	TRM82	1	psiblast	1erjA_	90
YDR165W	TRM82	2	psiblast	1gg2B_	354
YDR166C	SEC5	1	unassigned	na	86
YDR166C	SEC5	2	msa	na	76
YDR166C	SEC5	3	msa	na	230
YDR166C	SEC5	4	unassigned	na	140
YDR166C	SEC5	5	msa	na	139
YDR166C	SEC5	6	unassigned	na	54
YDR166C	SEC5	7	msa	na	246
YDR167W	TAF10	1	unassigned	na	66
YDR167W	TAF10	2	pfam	PF03540	140
YDR168W	CDC37	1	fold_recogniti	1bgxT	445
YDR168W	CDC37	2	fold_recogniti	1bgxT	61

YDR169C	STB3	1	msa	na	118
YDR169C	STB3	2	unassigned	na	395
YDR170C	SEC7	1	psiblast	1i84S_	355
YDR170C	SEC7	2	unassigned	na	422
YDR170C	SEC7	3	psiblast	1pbv__	120
YDR170C	SEC7	4	psiblast	1pbv__	135
YDR170C	SEC7	5	fold_recogniti	1b3uA	293
YDR170C	SEC7	6	fold_recogniti	1b3uA	76
YDR170C	SEC7	7	fold_recogniti	1b3uA	307
YDR170C	SEC7	8	unassigned	na	63
YDR170C	SEC7	9	msa	na	238
YDR171W	HSP42	1	unassigned	na	188
YDR171W	HSP42	2	psiblast	1gmeA_	187
YDR172W	SUP35	1	fold_recogniti	1ldvA	254
YDR172W	SUP35	2	psiblast	1jnyA_	228
YDR172W	SUP35	3	psiblast	1jnyA_	94
YDR172W	SUP35	4	psiblast	1jnyA_	109
YDR173C	ARG82	1	pfam	PF03770	355
YDR174W	HMO1	1	unassigned	na	92
YDR174W	HMO1	2	psiblast	1k99A_	154
YDR175C	RSM24	1	unassigned	na	127
YDR175C	RSM24	2	msa	na	192
YDR176W	NGG1	1	unassigned	na	702
YDR177W	UBC1	1	psiblast	1fxtA_	215
YDR178W	SDH4	1	unassigned	na	181
YDR179C	CSN9	1	unassigned	na	162
YDR179W-A	YDR179W-A	1	unassigned	na	268
YDR180W	SCC2	1	unassigned	na	354
YDR180W	SCC2	2	msa	na	1054
YDR180W	SCC2	3	unassigned	na	85
YDR181C	SAS4	1	unassigned	na	481
YDR182W	CDC1	1	psiblast	1kbpA_	161
YDR182W	CDC1	2	psiblast	1kbpA_	200
YDR182W	CDC1	3	unassigned	na	130
YDR183W	PLP1	1	psiblast	1b9yC_	230
YDR184C	ATC1	1	unassigned	na	131
YDR184C	ATC1	2	msa	na	163
YDR185C	YDR185C	1	unassigned	na	179
YDR186C	YDR186C	1	unassigned	na	77
YDR186C	YDR186C	2	fold_recogniti	1l9uD	337
YDR186C	YDR186C	3	unassigned	na	75
YDR186C	YDR186C	4	fold_recogniti	1iu4A	388
YDR187C	YDR187C	1	unassigned	na	172
YDR188W	CCT6	1	psiblast	1a6dA_	423
YDR188W	CCT6	2	psiblast	1a6dA_	123
YDR189W	SLY1	1	psiblast	1fvfA_	160
YDR189W	SLY1	2	psiblast	1fvfA_	223
YDR189W	SLY1	3	psiblast	1fvfA_	134

YDR189W	SLY1	4	psiblast	1fvfA_	149
YDR190C	RVB1	1	psiblast	1e32A_	230
YDR190C	RVB1	2	psiblast	1e32A_	95
YDR190C	RVB1	3	psiblast	1jr3A_	138
YDR191W	HST4	1	psiblast	1iciA_	257
YDR191W	HST4	2	psiblast	1iciA_	113
YDR192C	NUP42	1	fold_recogniti	1ei3A	328
YDR192C	NUP42	2	fold_recogniti	1deqA	102
YDR193W	YDR193W	1	unassigned	na	132
YDR194C	MSS116	1	unassigned	na	101
YDR194C	MSS116	2	psiblast	1hv8A_	236
YDR194C	MSS116	3	psiblast	1hv8A_	157
YDR194C	MSS116	4	psiblast	1d2mA_	170
YDR195W	REF2	1	fold_recogniti	1iwpA	465
YDR195W	REF2	2	unassigned	na	68
YDR196C	YDR196C	1	psiblast	1jjvA_	185
YDR196C	YDR196C	2	psiblast	1jjvA_	56
YDR197W	CBS2	1	psiblast	1ks9A_	245
YDR197W	CBS2	2	psiblast	1ks9A_	144
YDR198C	YDR198C	1	msa	na	357
YDR198C	YDR198C	2	unassigned	na	122
YDR199W	YDR199W	1	unassigned	na	121
YDR200C	VPS64	1	msa	na	152
YDR200C	VPS64	2	unassigned	na	67
YDR200C	VPS64	3	psiblast	1i84S_	385
YDR201W	SPC19	1	unassigned	na	165
YDR202C	RAV2	1	unassigned	na	351
YDR203W	YDR203W	1	psiblast	1crl__	105
YDR204W	COQ4	1	unassigned	na	79
YDR204W	COQ4	2	msa	na	256
YDR205W	MSC2	1	unassigned	na	379
YDR205W	MSC2	2	pfam	PF01545	361
YDR206W	EBS1	1	fold_recogniti	1hz4A	80
YDR206W	EBS1	2	fold_recogniti	1hz4A	184
YDR206W	EBS1	3	fold_recogniti	1hz4A	216
YDR206W	EBS1	4	unassigned	na	404
YDR207C	UME6	1	unassigned	na	251
YDR207C	UME6	2	fold_recogniti	1je5A	232
YDR207C	UME6	3	unassigned	na	136
YDR207C	UME6	4	msa	na	140
YDR207C	UME6	5	psiblast	1qp9A_	77
YDR208W	MSS4	1	unassigned	na	299
YDR208W	MSS4	2	psiblast	1bo1A_	257
YDR208W	MSS4	3	psiblast	1bo1A_	223
YDR209C	YDR209C	1	unassigned	na	137
YDR210W	YDR210W	1	unassigned	na	75
YDR211W	GCD6	1	psiblast	1hv9A_	299
YDR211W	GCD6	2	psiblast	1hv9A_	217

YDR211W	GCD6	3	unassigned	na	110
YDR211W	GCD6	4	pfam	PF02020	86
YDR212W	TCP1	1	psiblast	1a6dA_	432
YDR212W	TCP1	2	psiblast	1a6dA_	127
YDR213W	UPC2	1	psiblast	1pyiA_	166
YDR213W	UPC2	2	unassigned	na	479
YDR213W	UPC2	3	msa	na	149
YDR213W	UPC2	4	unassigned	na	119
YDR214W	AHA1	1	msa	na	166
YDR214W	AHA1	2	msa	na	184
YDR215C	YDR215C	1	unassigned	na	127
YDR216W	ADR1	1	psiblast	1a1lA_	100
YDR216W	ADR1	2	psiblast	2adr__	59
YDR216W	ADR1	3	psiblast	1ubdC_	106
YDR216W	ADR1	4	msa	na	154
YDR216W	ADR1	5	unassigned	na	403
YDR216W	ADR1	6	msa	na	501
YDR217C	RAD9	1	unassigned	na	991
YDR217C	RAD9	2	pfam	PF00533	144
YDR217C	RAD9	3	unassigned	na	174
YDR218C	SPR28	1	fold_recogniti	1egaA	246
YDR218C	SPR28	2	fold_recogniti	1egaA	105
YDR218C	SPR28	3	msa	na	72
YDR219C	YDR219C	1	pfam	PF00646	126
YDR219C	YDR219C	2	unassigned	na	339
YDR220C	YDR220C	1	unassigned	na	97
YDR221W	YDR221W	1	psiblast	1jv2B_	276
YDR221W	YDR221W	2	unassigned	na	158
YDR221W	YDR221W	3	msa	na	126
YDR221W	YDR221W	4	fold_recogniti	1keoA	142
YDR222W	YDR222W	1	unassigned	na	415
YDR223W	YDR223W	1	unassigned	na	202
YDR223W	YDR223W	2	msa	na	265
YDR224C	HTB1	1	psiblast	1id3D_	131
YDR225W	HTA1	1	psiblast	1id3C_	59
YDR225W	HTA1	2	psiblast	1id3C_	73
YDR226W	ADK1	1	psiblast	1aky__	222
YDR227W	SIR4	1	msa	na	123
YDR227W	SIR4	2	unassigned	na	1235
YDR228C	PCF11	1	msa	na	117
YDR228C	PCF11	2	msa	na	343
YDR228C	PCF11	3	unassigned	na	54
YDR228C	PCF11	4	msa	na	112
YDR229W	IVY1	1	unassigned	na	91
YDR229W	IVY1	2	fold_recogniti	1i49A	234
YDR229W	IVY1	3	unassigned	na	128
YDR230W	YDR230W	1	unassigned	na	115
YDR231C	COX20	1	unassigned	na	205

YDR232W	HEM1	1	unassigned	na	66
YDR232W	HEM1	2	psiblast	1fc4A_	211
YDR232W	HEM1	3	psiblast	1fc4A_	271
YDR233C	RTN1	1	pfam	PF02453	219
YDR233C	RTN1	2	unassigned	na	76
YDR234W	LYS4	1	psiblast	5acn__	517
YDR234W	LYS4	2	psiblast	5acn__	176
YDR235W	PRP42	1	psiblast	1fchA_	193
YDR235W	PRP42	2	psiblast	1fchA_	97
YDR235W	PRP42	3	psiblast	1fchA_	119
YDR235W	PRP42	4	unassigned	na	135
YDR236C	FMN1	1	unassigned	na	69
YDR236C	FMN1	2	pfam	PF01687	149
YDR237W	MRPL7	1	unassigned	na	102
YDR237W	MRPL7	2	psiblast	1iq4A_	190
YDR238C	SEC26	1	psiblast	1gw5B_	106
YDR238C	SEC26	2	psiblast	1gw5B_	105
YDR238C	SEC26	3	psiblast	1gw5B_	125
YDR238C	SEC26	4	psiblast	1gw5B_	100
YDR238C	SEC26	5	psiblast	1gw5B_	83
YDR238C	SEC26	6	psiblast	1jpwA_	96
YDR238C	SEC26	7	unassigned	na	174
YDR238C	SEC26	8	msa	na	184
YDR239C	YDR239C	1	unassigned	na	787
YDR240C	SNU56	1	unassigned	na	492
YDR241W	BUD26	1	unassigned	na	95
YDR242W	AMD2	1	psiblast	1gr8A_	549
YDR243C	PRP28	1	unassigned	na	139
YDR243C	PRP28	2	psiblast	1hv8A_	263
YDR243C	PRP28	3	psiblast	1hv8A_	186
YDR244W	PEX5	1	unassigned	na	195
YDR244W	PEX5	2	psiblast	1fchA_	200
YDR244W	PEX5	3	psiblast	1fchA_	76
YDR244W	PEX5	4	psiblast	1fchA_	60
YDR244W	PEX5	5	psiblast	1fchA_	81
YDR245W	MNN10	1	unassigned	na	105
YDR245W	MNN10	2	msa	na	140
YDR245W	MNN10	3	unassigned	na	148
YDR246W	TRS23	1	msa	na	74
YDR246W	TRS23	2	msa	na	145
YDR247W	VHS1	1	psiblast	1giiA_	127
YDR247W	VHS1	2	psiblast	1giiA_	81
YDR247W	VHS1	3	psiblast	1giiA_	128
YDR247W	VHS1	4	psiblast	1dtlA_	125
YDR248C	YDR248C	1	psiblast	1knqA_	193
YDR249C	YDR249C	1	unassigned	na	373
YDR250C	YDR250C	1	unassigned	na	91
YDR251W	PAM1	1	psiblast	1ks9A_	213

YDR251W	PAM1	2	psiblast	1ks9A_	158
YDR251W	PAM1	3	unassigned	na	90
YDR251W	PAM1	4	fold_recogniti	1i3qA	369
YDR252W	BTT1	1	pfam	PF01849	149
YDR253C	MET32	1	psiblast	1meyG_	191
YDR254W	CHL4	1	msa	na	228
YDR254W	CHL4	2	unassigned	na	230
YDR255C	RMD5	1	msa	na	72
YDR255C	RMD5	2	msa	na	208
YDR255C	RMD5	3	msa	na	141
YDR256C	CTA1	1	psiblast	1a4eA_	59
YDR256C	CTA1	2	psiblast	1a4eA_	456
YDR257C	SET7	1	msa	na	281
YDR257C	SET7	2	unassigned	na	213
YDR258C	HSP78	1	unassigned	na	91
YDR258C	HSP78	2	psiblast	1jbkA_	195
YDR258C	HSP78	3	fold_recogniti	1ksfX	139
YDR258C	HSP78	4	psiblast	1iqpA_	263
YDR258C	HSP78	5	fold_recogniti	1e94E	123
YDR259C	YAP6	1	unassigned	na	57
YDR259C	YAP6	2	psiblast	1i84S_	326
YDR260C	SWM1	1	unassigned	na	170
YDR261C	EXG2	1	psiblast	1cz1A_	369
YDR261C	EXG2	2	psiblast	1cz1A_	129
YDR261C	EXG2	3	unassigned	na	64
YDR262W	YDR262W	1	unassigned	na	272
YDR263C	DIN7	1	psiblast	1a76__	349
YDR263C	DIN7	2	unassigned	na	81
YDR264C	AKR1	1	psiblast	1k1aA_	170
YDR264C	AKR1	2	psiblast	1k1aA_	174
YDR264C	AKR1	3	msa	na	309
YDR264C	AKR1	4	msa	na	111
YDR265W	PEX10	1	unassigned	na	101
YDR265W	PEX10	2	psiblast	1fbvA_	184
YDR265W	PEX10	3	psiblast	1fbvA_	52
YDR266C	YDR266C	1	psiblast	1meyC_	181
YDR266C	YDR266C	2	psiblast	2gliA_	88
YDR266C	YDR266C	3	psiblast	2gliA_	78
YDR266C	YDR266C	4	psiblast	1f2iG_	126
YDR266C	YDR266C	5	unassigned	na	166
YDR267C	YDR267C	1	psiblast	1erjA_	330
YDR268W	MSW1	1	psiblast	1lkwA_	271
YDR268W	MSW1	2	psiblast	1lkwA_	108
YDR269C	YDR269C	1	unassigned	na	107
YDR270W	CCC2	1	psiblast	1fvqA_	75
YDR270W	CCC2	2	psiblast	1kqkA_	88
YDR270W	CCC2	3	fold_recogniti	1k0vA	119
YDR270W	CCC2	4	unassigned	na	53

YDR270W	CCC2	5	psiblast	1eulA_	525
YDR270W	CCC2	6	psiblast	1eulA_	144
YDR271C	YDR271C	1	unassigned	na	123
YDR272W	GLO2	1	psiblast	1qh3A_	120
YDR272W	GLO2	2	psiblast	1qh3A_	70
YDR272W	GLO2	3	psiblast	1qh3A_	84
YDR273W	DON1	1	fold_recogniti	1ej6B	231
YDR273W	DON1	2	unassigned	na	134
YDR274C	YDR274C	1	unassigned	na	123
YDR275W	BSC2	1	unassigned	na	235
YDR276C	PMP3	1	pfam	PF01679	55
YDR277C	MTH1	1	unassigned	na	433
YDR278C	YDR278C	1	unassigned	na	105
YDR279W	RNH202	1	unassigned	na	350
YDR280W	RRP45	1	psiblast	1e3pA_	114
YDR280W	RRP45	2	psiblast	1e3pA_	191
YDR281C	PHM6	1	unassigned	na	104
YDR282C	YDR282C	1	unassigned	na	90
YDR282C	YDR282C	2	pfam	PF02582	324
YDR283C	GCN2	1	msa	na	199
YDR283C	GCN2	2	unassigned	na	64
YDR283C	GCN2	3	psiblast	1pme__	316
YDR283C	GCN2	4	psiblast	1lwpA_	123
YDR283C	GCN2	5	psiblast	1lwpA_	98
YDR283C	GCN2	6	psiblast	1lwpA_	138
YDR283C	GCN2	7	psiblast	1a06__	91
YDR283C	GCN2	8	psiblast	1httA_	296
YDR283C	GCN2	9	psiblast	1httA_	192
YDR283C	GCN2	10	unassigned	na	142
YDR284C	DPP1	1	unassigned	na	60
YDR284C	DPP1	2	psiblast	1d2tA_	229
YDR285W	ZIP1	1	psiblast	1i84S_	321
YDR285W	ZIP1	2	psiblast	1c1gA_	73
YDR285W	ZIP1	3	unassigned	na	57
YDR285W	ZIP1	4	psiblast	2tmaA_	71
YDR285W	ZIP1	5	psiblast	2tmaA_	79
YDR285W	ZIP1	6	psiblast	2tmaA_	62
YDR285W	ZIP1	7	psiblast	2tmaA_	104
YDR285W	ZIP1	8	msa	na	108
YDR286C	YDR286C	1	unassigned	na	114
YDR287W	YDR287W	1	psiblast	1imaA_	148
YDR287W	YDR287W	2	psiblast	1imaA_	144
YDR288W	YDR288W	1	fold_recogniti	1dgsA	129
YDR288W	YDR288W	2	unassigned	na	174
YDR289C	RTT103	1	msa	na	115
YDR289C	RTT103	2	fold_recogniti	1l8mA	72
YDR289C	RTT103	3	fold_recogniti	1l8mA	79
YDR289C	RTT103	4	fold_recogniti	1l8mA	66

YDR289C	RTT103	5	fold_recogniti	1l8mA	77
YDR290W	SWS1	1	unassigned	na	109
YDR291W	YDR291W	1	msa	na	255
YDR291W	YDR291W	2	psiblast	1fuuA_	477
YDR291W	YDR291W	3	msa	na	151
YDR291W	YDR291W	4	msa	na	194
YDR292C	SRP101	1	msa	na	215
YDR292C	SRP101	2	unassigned	na	57
YDR292C	SRP101	3	psiblast	1fts__	280
YDR292C	SRP101	4	psiblast	1fts__	69
YDR293C	SSD1	1	psiblast	1deqA_	222
YDR293C	SSD1	2	msa	na	157
YDR293C	SSD1	3	unassigned	na	195
YDR293C	SSD1	4	msa	na	494
YDR293C	SSD1	5	psiblast	1go3E_	96
YDR293C	SSD1	6	unassigned	na	86
YDR294C	DPL1	1	unassigned	na	79
YDR294C	DPL1	2	psiblast	1js3A_	83
YDR294C	DPL1	3	psiblast	1js3A_	427
YDR295C	HDA2	1	unassigned	na	59
YDR295C	HDA2	2	psiblast	1i84S_	332
YDR295C	HDA2	3	psiblast	1c1gA_	107
YDR295C	HDA2	4	msa	na	176
YDR296W	MHR1	1	unassigned	na	226
YDR297W	SUR2	1	pfam	PF01598	349
YDR298C	ATP5	1	psiblast	1abv__	212
YDR299W	BFR2	1	fold_recogniti	1c1gB	534
YDR300C	PRO1	1	psiblast	1e19A_	292
YDR300C	PRO1	2	pfam	PF01472	136
YDR301W	CFT1	1	msa	na	722
YDR301W	CFT1	2	unassigned	na	264
YDR301W	CFT1	3	pfam	PF03178	371
YDR302W	GPI11	1	msa	na	120
YDR302W	GPI11	2	msa	na	99
YDR303C	RSC3	1	psiblast	1cld__	52
YDR303C	RSC3	2	msa	na	149
YDR303C	RSC3	3	unassigned	na	354
YDR303C	RSC3	4	msa	na	330
YDR304C	CPR5	1	psiblast	1cynA_	225
YDR305C	HNT2	1	psiblast	1fhi__	160
YDR305C	HNT2	2	unassigned	na	57
YDR306C	YDR306C	1	unassigned	na	478
YDR307W	YDR307W	1	msa	na	270
YDR307W	YDR307W	2	pfam	PF02815	83
YDR307W	YDR307W	3	pfam	PF02815	73
YDR307W	YDR307W	4	pfam	PF02815	71
YDR307W	YDR307W	5	unassigned	na	66
YDR307W	YDR307W	6	msa	na	99

YDR308C	SRB7	1	psiblast	1i84S_	140
YDR309C	GIC2	1	unassigned	na	100
YDR309C	GIC2	2	pfam	PF00786	100
YDR309C	GIC2	3	unassigned	na	183
YDR310C	SUM1	1	unassigned	na	189
YDR310C	SUM1	2	msa	na	222
YDR310C	SUM1	3	unassigned	na	651
YDR311W	TFB1	1	unassigned	na	156
YDR311W	TFB1	2	pfam	PF03909	82
YDR311W	TFB1	3	pfam	PF03909	114
YDR311W	TFB1	4	unassigned	na	290
YDR312W	SSF2	1	pfam	PF03877	290
YDR312W	SSF2	2	unassigned	na	82
YDR312W	SSF2	3	msa	na	81
YDR313C	PIB1	1	psiblast	1jocA_	89
YDR313C	PIB1	2	unassigned	na	70
YDR313C	PIB1	3	psiblast	1chc__	127
YDR314C	YDR314C	1	unassigned	na	269
YDR314C	YDR314C	2	pfam	PF03835	423
YDR315C	IPK1	1	unassigned	na	281
YDR316W	OMS1	1	unassigned	na	146
YDR316W	OMS1	2	psiblast	1kpiA_	325
YDR317W	YDR317W	1	unassigned	na	414
YDR318W	MCM21	1	unassigned	na	251
YDR319C	YDR319C	1	unassigned	na	170
YDR319C	YDR319C	2	msa	na	104
YDR320C	SWA2	1	unassigned	na	352
YDR320C	SWA2	2	psiblast	1elwA_	191
YDR320C	SWA2	3	unassigned	na	55
YDR320C	SWA2	4	psiblast	1xbl__	70
YDR320C-A	DAD4	1	unassigned	na	72
YDR321W	ASP1	1	psiblast	1hfjA_	264
YDR321W	ASP1	2	psiblast	1hfjA_	117
YDR322C-A	TIM11	1	unassigned	na	96
YDR322W	MRPL35	1	unassigned	na	135
YDR322W	MRPL35	2	psiblast	1kn3A_	232
YDR323C	PEP7	1	fold_recogniti	1dvpA	158
YDR323C	PEP7	2	psiblast	1vfyA_	149
YDR323C	PEP7	3	msa	na	208
YDR324C	UTP4	1	unassigned	na	73
YDR324C	UTP4	2	psiblast	1gg2B_	93
YDR324C	UTP4	3	psiblast	1erjA_	338
YDR324C	UTP4	4	msa	na	188
YDR324C	UTP4	5	unassigned	na	84
YDR325W	YCG1	1	msa	na	537
YDR325W	YCG1	2	unassigned	na	300
YDR325W	YCG1	3	msa	na	214
YDR326C	YDR326C	1	unassigned	na	469

YDR326C	YDR326C	2	msa	na	152
YDR326C	YDR326C	3	pfam	PF02893	149
YDR326C	YDR326C	4	unassigned	na	59
YDR326C	YDR326C	5	msa	na	87
YDR326C	YDR326C	6	unassigned	na	88
YDR326C	YDR326C	7	msa	na	259
YDR326C	YDR326C	8	unassigned	na	175
YDR327W	YDR327W	1	unassigned	na	108
YDR328C	SKP1	1	psiblast	1fqvB_	194
YDR329C	PEX3	1	unassigned	na	121
YDR329C	PEX3	2	msa	na	320
YDR330W	UBX5	1	unassigned	na	202
YDR330W	UBX5	2	msa	na	152
YDR330W	UBX5	3	psiblast	1jruA_	146
YDR331W	GPI8	1	pfam	PF01650	358
YDR331W	GPI8	2	unassigned	na	53
YDR332W	YDR332W	1	psiblast	1fuuA_	410
YDR332W	YDR332W	2	psiblast	1d9xA_	113
YDR332W	YDR332W	3	unassigned	na	166
YDR333C	YDR333C	1	msa	na	298
YDR333C	YDR333C	2	unassigned	na	55
YDR333C	YDR333C	3	fold_recogniti	1fchA	124
YDR333C	YDR333C	4	fold_recogniti	1fchA	131
YDR333C	YDR333C	5	unassigned	na	115
YDR334W	SWR1	1	psiblast	1i84S_	346
YDR334W	SWR1	2	unassigned	na	286
YDR334W	SWR1	3	pfam	PF00176	373
YDR334W	SWR1	4	unassigned	na	232
YDR334W	SWR1	5	pfam	PF00271	123
YDR334W	SWR1	6	msa	na	154
YDR335W	MSN5	1	psiblast	1ibrB_	253
YDR335W	MSN5	2	msa	na	140
YDR335W	MSN5	3	msa	na	88
YDR335W	MSN5	4	msa	na	166
YDR335W	MSN5	5	msa	na	484
YDR335W	MSN5	6	msa	na	93
YDR336W	YDR336W	1	msa	na	122
YDR336W	YDR336W	2	psiblast	1egaA_	192
YDR337W	MRPS28	1	unassigned	na	132
YDR337W	MRPS28	2	psiblast	1eg0F_	154
YDR338C	YDR338C	1	unassigned	na	200
YDR338C	YDR338C	2	fold_recogniti	1b3uA	143
YDR338C	YDR338C	3	fold_recogniti	1b3uA	169
YDR338C	YDR338C	4	fold_recogniti	1b3uA	183
YDR339C	YDR339C	1	unassigned	na	189
YDR340W	YDR340W	1	unassigned	na	100
YDR341C	YDR341C	1	psiblast	1bs2A_	115
YDR341C	YDR341C	2	psiblast	1bs2A_	214

YDR341C	YDR341C	3	psiblast	1bs2A_	198
YDR341C	YDR341C	4	psiblast	1bs2A_	80
YDR342C	HXT7	1	psiblast	1ja5A_	469
YDR342C	HXT7	2	psiblast	1ja5A_	101
YDR343C	HXT6	1	psiblast	1ja5A_	469
YDR343C	HXT6	2	psiblast	1ja5A_	101
YDR344C	YDR344C	1	unassigned	na	147
YDR345C	HXT3	1	psiblast	1ja5A_	466
YDR345C	HXT3	2	psiblast	1ja5A_	101
YDR346C	SVF1	1	msa	na	203
YDR346C	SVF1	2	unassigned	na	278
YDR347W	MRP1	1	psiblast	3mdsA_	90
YDR347W	MRP1	2	psiblast	3mdsA_	151
YDR347W	MRP1	3	psiblast	1lusA_	80
YDR348C	YDR348C	1	unassigned	na	77
YDR348C	YDR348C	2	msa	na	122
YDR348C	YDR348C	3	msa	na	207
YDR348C	YDR348C	4	unassigned	na	93
YDR349C	YPS7	1	psiblast	1j71A_	344
YDR349C	YPS7	2	psiblast	1j71A_	170
YDR349C	YPS7	3	unassigned	na	82
YDR350C	TCM10	1	unassigned	na	611
YDR351W	SBE2	1	unassigned	na	864
YDR352W	YDR352W	1	msa	na	135
YDR352W	YDR352W	2	msa	na	182
YDR353W	TRR1	1	psiblast	1vdc__	193
YDR353W	TRR1	2	psiblast	1vdc__	126
YDR354W	TRP4	1	psiblast	1brwA_	117
YDR354W	TRP4	2	psiblast	1brwA_	263
YDR355C	YDR355C	1	unassigned	na	100
YDR356W	SPC110	1	unassigned	na	77
YDR356W	SPC110	2	psiblast	1i84S_	332
YDR356W	SPC110	3	psiblast	2tmaA_	69
YDR356W	SPC110	4	fold_recogniti	1hciA	171
YDR356W	SPC110	5	fold_recogniti	1hciA	198
YDR356W	SPC110	6	unassigned	na	97
YDR357C	YDR357C	1	unassigned	na	122
YDR358W	GGA1	1	psiblast	1dvpA_	93
YDR358W	GGA1	2	psiblast	1dvpA_	94
YDR358W	GGA1	3	fold_recogniti	1vsgA	174
YDR358W	GGA1	4	fold_recogniti	1vsgA	48
YDR358W	GGA1	5	psiblast	1iu1A_	148
YDR359C	VID21	1	unassigned	na	259
YDR359C	VID21	2	msa	na	147
YDR359C	VID21	3	unassigned	na	173
YDR359C	VID21	4	msa	na	261
YDR359C	VID21	5	unassigned	na	119
YDR360W	YDR360W	1	unassigned	na	128

YDR361C	BCP1	1	unassigned	na	81
YDR361C	BCP1	2	msa	na	202
YDR362C	TFC6	1	unassigned	na	306
YDR362C	TFC6	2	fold_recogniti	1erjA	366
YDR363W	ESC2	1	unassigned	na	346
YDR363W	ESC2	2	psiblast	1a5r__	110
YDR363W-A	SEM1	1	fold_recogniti	1iyjA	89
YDR364C	CDC40	1	msa	na	124
YDR364C	CDC40	2	psiblast	1erjA_	331
YDR365C	ESF1	1	msa	na	206
YDR365C	ESF1	2	unassigned	na	92
YDR365C	ESF1	3	msa	na	206
YDR365C	ESF1	4	unassigned	na	124
YDR366C	YDR366C	1	unassigned	na	132
YDR367W	YDR367W	1	unassigned	na	221
YDR368W	YPR1	1	psiblast	2alr__	312
YDR369C	XRS2	1	pfam	PF00498	157
YDR369C	XRS2	2	unassigned	na	330
YDR369C	XRS2	3	msa	na	367
YDR370C	YDR370C	1	unassigned	na	442
YDR371W	CTS2	1	unassigned	na	69
YDR371W	CTS2	2	psiblast	1d2kA_	100
YDR371W	CTS2	3	psiblast	1d2kA_	342
YDR372C	VPS74	1	unassigned	na	64
YDR372C	VPS74	2	msa	na	281
YDR373W	FRQ1	1	psiblast	1fpwA_	94
YDR373W	FRQ1	2	psiblast	1fpwA_	96
YDR374C	YDR374C	1	unassigned	na	74
YDR374C	YDR374C	2	msa	na	232
YDR375C	BCS1	1	unassigned	na	166
YDR375C	BCS1	2	psiblast	1e32A_	222
YDR375C	BCS1	3	psiblast	1e32A_	68
YDR376W	ARH1	1	psiblast	1cjcA_	239
YDR376W	ARH1	2	psiblast	1cjcA_	254
YDR377W	ATP17	1	unassigned	na	101
YDR378C	LSM6	1	psiblast	1i81A_	123
YDR379C-A	YDR379C-A	1	unassigned	na	79
YDR379W	RGA2	1	psiblast	1b8tA_	70
YDR379W	RGA2	2	psiblast	1b8tA_	79
YDR379W	RGA2	3	psiblast	1bw5__	76
YDR379W	RGA2	4	msa	na	355
YDR379W	RGA2	5	unassigned	na	145
YDR379W	RGA2	6	psiblast	1rgp__	154
YDR379W	RGA2	7	psiblast	1rgp__	130
YDR380W	ARO10	1	psiblast	1qpbA_	227
YDR380W	ARO10	2	psiblast	1qpbA_	176
YDR380W	ARO10	3	psiblast	1qpbA_	232
YDR381C-A	YDR381C-A	1	unassigned	na	11

YDR381W	YRA1	1	psiblast	1l3kA_	67
YDR381W	YRA1	2	psiblast	1l3kA_	159
YDR382W	RPP2B	1	pfam	PF00428	110
YDR383C	NKP1	1	unassigned	na	252
YDR384C	ATO3	1	unassigned	na	70
YDR384C	ATO3	2	pfam	PF01184	205
YDR385W	EFT2	1	psiblast	1dar__	349
YDR385W	EFT2	2	psiblast	1dar__	167
YDR385W	EFT2	3	psiblast	1dar__	326
YDR386W	MUS81	1	msa	na	226
YDR386W	MUS81	2	unassigned	na	108
YDR386W	MUS81	3	pfam	PF02732	120
YDR386W	MUS81	4	msa	na	178
YDR387C	YDR387C	1	psiblast	1ja5A_	486
YDR387C	YDR387C	2	psiblast	1ja5A_	69
YDR388W	RVS167	1	psiblast	1i84S_	257
YDR388W	RVS167	2	fold_recogniti	1m2vB	168
YDR388W	RVS167	3	psiblast	2semA_	57
YDR389W	SAC7	1	unassigned	na	68
YDR389W	SAC7	2	psiblast	1rgp__	153
YDR389W	SAC7	3	psiblast	1rgp__	103
YDR389W	SAC7	4	fold_recogniti	1pbwA	110
YDR389W	SAC7	5	unassigned	na	220
YDR390C	UBA2	1	psiblast	1jw9B_	273
YDR390C	UBA2	2	unassigned	na	57
YDR390C	UBA2	3	pfam	PF02134	176
YDR390C	UBA2	4	msa	na	130
YDR391C	YDR391C	1	unassigned	na	109
YDR391C	YDR391C	2	psiblast	1cjwA_	123
YDR392W	SPT3	1	psiblast	1bh8A_	50
YDR392W	SPT3	2	pfam	PF02269	59
YDR392W	SPT3	3	unassigned	na	228
YDR393W	SHE9	1	psiblast	1i84S_	349
YDR393W	SHE9	2	psiblast	1c1gA_	107
YDR394W	RPT3	1	msa	na	140
YDR394W	RPT3	2	psiblast	1e32A_	203
YDR394W	RPT3	3	psiblast	1e32A_	85
YDR395W	SXM1	1	psiblast	1qgkA_	254
YDR395W	SXM1	2	psiblast	1qgkA_	230
YDR395W	SXM1	3	psiblast	1qgkA_	121
YDR395W	SXM1	4	psiblast	1qgkA_	190
YDR395W	SXM1	5	msa	na	149
YDR396W	YDR396W	1	unassigned	na	166
YDR397C	NCB2	1	psiblast	1jfiB_	78
YDR397C	NCB2	2	psiblast	1jfiB_	68
YDR398W	UTP5	1	psiblast	1erjA_	365
YDR398W	UTP5	2	msa	na	198
YDR398W	UTP5	3	msa	na	80

YDR399W	HPT1	1	psiblast	1bzyA_	221
YDR400W	URH1	1	psiblast	1ezrA_	378
YDR401W	YDR401W	1	unassigned	na	187
YDR402C	DIT2	1	psiblast	2c17A_	489
YDR403W	DIT1	1	unassigned	na	156
YDR403W	DIT1	2	msa	na	380
YDR404C	RPB7	1	psiblast	1go3E_	79
YDR404C	RPB7	2	psiblast	1go3E_	92
YDR405W	MRP20	1	unassigned	na	80
YDR405W	MRP20	2	psiblast	1lnrR_	102
YDR405W	MRP20	3	unassigned	na	81
YDR406W	PDR15	1	unassigned	na	151
YDR406W	PDR15	2	psiblast	1f3oA_	93
YDR406W	PDR15	3	psiblast	1f3oA_	201
YDR406W	PDR15	4	unassigned	na	62
YDR406W	PDR15	5	psiblast	1jsqA_	335
YDR406W	PDR15	6	psiblast	1jsqA_	292
YDR406W	PDR15	7	msa	na	303
YDR406W	PDR15	8	unassigned	na	92
YDR407C	TRS120	1	unassigned	na	204
YDR407C	TRS120	2	msa	na	788
YDR407C	TRS120	3	unassigned	na	297
YDR408C	ADE8	1	psiblast	1c3eA_	114
YDR408C	ADE8	2	psiblast	1c3eA_	100
YDR409W	SIZ1	1	pfam	PF02037	106
YDR409W	SIZ1	2	msa	na	113
YDR409W	SIZ1	3	unassigned	na	70
YDR409W	SIZ1	4	pfam	PF02891	121
YDR409W	SIZ1	5	msa	na	119
YDR409W	SIZ1	6	fold_recogniti	1i3qA	375
YDR410C	STE14	1	unassigned	na	72
YDR410C	STE14	2	msa	na	167
YDR411C	YDR411C	1	msa	na	267
YDR411C	YDR411C	2	unassigned	na	74
YDR412W	YDR412W	1	msa	na	85
YDR412W	YDR412W	2	msa	na	150
YDR413C	YDR413C	1	fold_recogniti	1brrB	116
YDR413C	YDR413C	2	unassigned	na	75
YDR414C	ERD1	1	unassigned	na	152
YDR414C	ERD1	2	pfam	PF03124	210
YDR415C	YDR415C	1	unassigned	na	79
YDR415C	YDR415C	2	psiblast	1amp__	295
YDR416W	SYF1	1	fold_recogniti	1jcqA	161
YDR416W	SYF1	2	fold_recogniti	1jcqA	93
YDR416W	SYF1	3	fold_recogniti	1jcqA	118
YDR416W	SYF1	4	psiblast	1fchA_	161
YDR416W	SYF1	5	psiblast	1fchA_	66
YDR416W	SYF1	6	psiblast	1fchA_	58

YDR416W	SYF1	7	fold_recogniti	1fchA	99
YDR416W	SYF1	8	fold_recogniti	1fchA	103
YDR417C	YDR417C	1	unassigned	na	123
YDR418W	RPL12B	1	psiblast	1k5yK_	74
YDR418W	RPL12B	2	psiblast	1k5yK_	91
YDR419W	RAD30	1	psiblast	1jihA_	211
YDR419W	RAD30	2	psiblast	1jihA_	177
YDR419W	RAD30	3	psiblast	1jihA_	142
YDR419W	RAD30	4	unassigned	na	102
YDR420W	HKR1	1	msa	na	1028
YDR420W	HKR1	2	msa	na	405
YDR420W	HKR1	3	unassigned	na	369
YDR421W	ARO80	1	fold_recogniti	1d66A	89
YDR421W	ARO80	2	unassigned	na	861
YDR422C	SIP1	1	unassigned	na	483
YDR422C	SIP1	2	msa	na	194
YDR422C	SIP1	3	msa	na	186
YDR423C	CAD1	1	psiblast	1gd2E_	133
YDR423C	CAD1	2	msa	na	127
YDR423C	CAD1	3	unassigned	na	66
YDR423C	CAD1	4	msa	na	83
YDR424C	DYN2	1	psiblast	1f3cA_	92
YDR425W	SNX41	1	psiblast	1almA_	125
YDR425W	SNX41	2	psiblast	1i84S_	341
YDR425W	SNX41	3	psiblast	1c1gA_	159
YDR426C	YDR426C	1	unassigned	na	125
YDR427W	RPN9	1	fold_recogniti	1hz4A	133
YDR427W	RPN9	2	fold_recogniti	1hz4A	118
YDR427W	RPN9	3	fold_recogniti	1hz4A	142
YDR428C	YDR428C	1	psiblast	1evqA_	261
YDR429C	TIF35	1	unassigned	na	64
YDR429C	TIF35	2	psiblast	1b7fA_	119
YDR429C	TIF35	3	psiblast	1b7fA_	91
YDR430C	CYM1	1	psiblast	1bccA_	294
YDR430C	CYM1	2	psiblast	1bccA_	238
YDR430C	CYM1	3	psiblast	1ezvB_	236
YDR430C	CYM1	4	psiblast	1ezvB_	148
YDR430C	CYM1	5	unassigned	na	73
YDR431W	YDR431W	1	unassigned	na	103
YDR432W	NPL3	1	fold_recogniti	1ldvA	124
YDR432W	NPL3	2	psiblast	1b7fA_	71
YDR432W	NPL3	3	psiblast	1b7fA_	79
YDR432W	NPL3	4	fold_recogniti	1deqA	140
YDR433W	YDR433W	1	unassigned	na	146
YDR434W	GPI17	1	msa	na	168
YDR434W	GPI17	2	unassigned	na	142
YDR434W	GPI17	3	msa	na	224
YDR435C	PPM1	1	msa	na	256

YDR435C	PPM1	2	unassigned	na	72
YDR436W	PPZ2	1	fold_recogniti	1i3qA	326
YDR436W	PPZ2	2	unassigned	na	63
YDR436W	PPZ2	3	psiblast	1it6A_	321
YDR437W	YDR437W	1	fold_recogniti	1fnuA	140
YDR438W	YDR438W	1	pfam	PF00892	199
YDR438W	YDR438W	2	msa	na	171
YDR439W	LRS4	1	unassigned	na	347
YDR440W	DOT1	1	unassigned	na	174
YDR440W	DOT1	2	msa	na	153
YDR440W	DOT1	3	unassigned	na	64
YDR440W	DOT1	4	psiblast	1m0rA_	120
YDR440W	DOT1	5	unassigned	na	71
YDR441C	APT2	1	psiblast	1g2pA_	181
YDR442W	YDR442W	1	unassigned	na	130
YDR443C	SSN2	1	unassigned	na	1420
YDR444W	YDR444W	1	unassigned	na	165
YDR444W	YDR444W	2	psiblast	1eh5A_	286
YDR444W	YDR444W	3	unassigned	na	236
YDR445C	YDR445C	1	unassigned	na	135
YDR446W	ECM11	1	unassigned	na	302
YDR447C	RPS17B	1	pfam	PF00833	136
YDR448W	ADA2	1	pfam	PF00569	40
YDR448W	ADA2	2	fold_recogniti	1h88C	69
YDR448W	ADA2	3	psiblast	1i84S_	245
YDR448W	ADA2	4	msa	na	80
YDR449C	UTP6	1	psiblast	1fchA_	64
YDR449C	UTP6	2	psiblast	1fchA_	60
YDR449C	UTP6	3	psiblast	1fchA_	97
YDR449C	UTP6	4	unassigned	na	68
YDR449C	UTP6	5	fold_recogniti	1bn7A	151
YDR450W	RPS18A	1	psiblast	1k5xM_	146
YDR451C	YHP1	1	msa	na	157
YDR451C	YHP1	2	psiblast	1fjlA_	85
YDR451C	YHP1	3	msa	na	111
YDR452W	PPN1	1	fold_recogniti	1qhwA	394
YDR452W	PPN1	2	unassigned	na	280
YDR453C	TSA2	1	psiblast	1qmvA_	196
YDR454C	GUK1	1	psiblast	1ex6A_	135
YDR454C	GUK1	2	psiblast	1ex6A_	52
YDR455C	YDR455C	1	unassigned	na	102
YDR456W	NHX1	1	pfam	PF00999	506
YDR456W	NHX1	2	unassigned	na	127
YDR457W	TOM1	1	msa	na	746
YDR457W	TOM1	2	unassigned	na	905
YDR457W	TOM1	3	msa	na	194
YDR457W	TOM1	4	msa	na	325
YDR457W	TOM1	5	unassigned	na	597

YDR457W	TOM1	6	psiblast	1i5hW_	106
YDR457W	TOM1	7	psiblast	1c4zA_	78
YDR457W	TOM1	8	psiblast	1c4zA_	141
YDR457W	TOM1	9	psiblast	1c4zA_	176
YDR458C	YDR458C	1	psiblast	1i84S_	254
YDR458C	YDR458C	2	unassigned	na	267
YDR458C	YDR458C	3	msa	na	142
YDR459C	YDR459C	1	unassigned	na	101
YDR459C	YDR459C	2	pfam	PF01529	106
YDR459C	YDR459C	3	unassigned	na	167
YDR460W	TFB3	1	psiblast	1g25A_	67
YDR460W	TFB3	2	psiblast	1i84S_	197
YDR460W	TFB3	3	unassigned	na	57
YDR461W	MFA1	1	unassigned	na	36
YDR462W	MRPL28	1	unassigned	na	147
YDR463W	STP1	1	msa	na	168
YDR463W	STP1	2	psiblast	1meyG_	102
YDR463W	STP1	3	unassigned	na	53
YDR463W	STP1	4	psiblast	1ubdC_	152
YDR463W	STP1	5	unassigned	na	102
YDR464W	SPP41	1	msa	na	619
YDR464W	SPP41	2	msa	na	591
YDR464W	SPP41	3	unassigned	na	52
YDR464W	SPP41	4	msa	na	173
YDR465C	RMT2	1	unassigned	na	67
YDR465C	RMT2	2	msa	na	120
YDR465C	RMT2	3	psiblast	1khhA_	225
YDR466W	PKH3	1	psiblast	1cdkA_	75
YDR466W	PKH3	2	psiblast	1cdkA_	193
YDR466W	PKH3	3	psiblast	1i84S_	137
YDR466W	PKH3	4	psiblast	1i84S_	239
YDR466W	PKH3	5	fold_recogniti	1i3qA	254
YDR467C	YDR467C	1	unassigned	na	108
YDR468C	TLG1	1	psiblast	1dn1B_	178
YDR468C	TLG1	2	psiblast	1dn1B_	46
YDR469W	SDC1	1	unassigned	na	175
YDR470C	UGO1	1	unassigned	na	167
YDR470C	UGO1	2	msa	na	335
YDR471W	RPL27B	1	pfam	PF00467	48
YDR471W	RPL27B	2	pfam	PF01777	88
YDR472W	TRS31	1	msa	na	148
YDR472W	TRS31	2	msa	na	135
YDR473C	PRP3	1	unassigned	na	178
YDR473C	PRP3	2	msa	na	173
YDR473C	PRP3	3	unassigned	na	118
YDR474C	1574	1	unassigned	na	253
YDR474C	1574	2	fold_recogniti	1ldvA	302
YDR475C	JIP4	1	unassigned	na	155

YDR476C	YDR476C	1	unassigned	na	224
YDR477W	SNF1	1	psiblast	1a06__	145
YDR477W	SNF1	2	psiblast	1a06__	192
YDR477W	SNF1	3	psiblast	1ahr__	83
YDR477W	SNF1	4	psiblast	1ahr__	79
YDR477W	SNF1	5	unassigned	na	134
YDR478W	SNM1	1	unassigned	na	198
YDR479C	PEX29	1	msa	na	496
YDR479C	PEX29	2	unassigned	na	58
YDR480W	DIG2	1	unassigned	na	323
YDR481C	PHO8	1	psiblast	1ew2A_	566
YDR482C	CWC21	1	fold_recogniti	1dkxA	135
YDR483W	KRE2	1	pfam	PF01793	442
YDR484W	VPS52	1	fold_recogniti	1h6oA	195
YDR484W	VPS52	2	fold_recogniti	1h6oA	132
YDR484W	VPS52	3	msa	na	314
YDR485C	VPS72	1	fold_recogniti	1l8mA	96
YDR485C	VPS72	2	fold_recogniti	1l8mA	79
YDR485C	VPS72	3	fold_recogniti	1l8mA	70
YDR485C	VPS72	4	fold_recogniti	1l8mA	76
YDR485C	VPS72	5	fold_recogniti	1ldvA	84
YDR485C	VPS72	6	fold_recogniti	1ei3A	333
YDR485C	VPS72	7	unassigned	na	72
YDR486C	VPS60	1	psiblast	1i84S_	262
YDR487C	RIB3	1	psiblast	1k49A_	208
YDR488C	PAC11	1	msa	na	140
YDR488C	PAC11	2	psiblast	1erjA_	393
YDR489W	SLD5	1	fold_recogniti	1dg3A	128
YDR489W	SLD5	2	fold_recogniti	1dg3A	105
YDR489W	SLD5	3	unassigned	na	61
YDR490C	PKH1	1	unassigned	na	72
YDR490C	PKH1	2	psiblast	1fotA_	191
YDR490C	PKH1	3	psiblast	1fotA_	220
YDR490C	PKH1	4	psiblast	1i84S_	213
YDR490C	PKH1	5	unassigned	na	70
YDR491C	YDR491C	1	unassigned	na	163
YDR492W	YDR492W	1	pfam	PF03006	316
YDR493W	FMP36	1	unassigned	na	123
YDR494W	RSM28	1	msa	na	127
YDR494W	RSM28	2	unassigned	na	161
YDR495C	VPS3	1	unassigned	na	98
YDR495C	VPS3	2	msa	na	628
YDR495C	VPS3	3	unassigned	na	285
YDR496C	PUF6	1	psiblast	1ib2A_	157
YDR496C	PUF6	2	psiblast	1ib2A_	100
YDR496C	PUF6	3	psiblast	1ib2A_	187
YDR496C	PUF6	4	msa	na	212
YDR497C	ITR1	1	unassigned	na	67

YDR497C	ITR1	2	psiblast	1ja5A_	416
YDR497C	ITR1	3	psiblast	1ja5A_	101
YDR498C	SEC20	1	unassigned	na	186
YDR498C	SEC20	2	pfam	PF03908	133
YDR498C	SEC20	3	unassigned	na	64
YDR499W	LCD1	1	unassigned	na	194
YDR499W	LCD1	2	fold_recogniti	1h6kA	90
YDR499W	LCD1	3	fold_recogniti	1h6kA	282
YDR499W	LCD1	4	fold_recogniti	1qoyA	181
YDR500C	RPL37B	1	psiblast	1k5yZ_	88
YDR501W	PLM2	1	msa	na	233
YDR501W	PLM2	2	unassigned	na	109
YDR501W	PLM2	3	msa	na	179
YDR502C	SAM2	1	psiblast	1qm4A_	170
YDR502C	SAM2	2	psiblast	1qm4A_	105
YDR502C	SAM2	3	psiblast	1qm4A_	109
YDR503C	LPP1	1	unassigned	na	64
YDR503C	LPP1	2	psiblast	1d2tA_	210
YDR504C	SPG3	1	unassigned	na	127
YDR505C	PSP1	1	unassigned	na	178
YDR505C	PSP1	2	fold_recogniti	1ruxA	210
YDR505C	PSP1	3	fold_recogniti	1ruxA	226
YDR505C	PSP1	4	fold_recogniti	1iw7D	227
YDR506C	YDR506C	1	psiblast	1hfuA_	415
YDR506C	YDR506C	2	psiblast	1hfuA_	193
YDR507C	GIN4	1	psiblast	1a06__	173
YDR507C	GIN4	2	psiblast	1a06__	198
YDR507C	GIN4	3	unassigned	na	771
YDR508C	GNP1	1	unassigned	na	121
YDR508C	GNP1	2	pfam	PF00324	542
YDR509W	YDR509W	1	unassigned	na	115
YDR510W	SMT3	1	psiblast	1l2nA_	101
YDR511W	ACN9	1	unassigned	na	133
YDR512C	EMI1	1	unassigned	na	187
YDR513W	TTR1	1	psiblast	1kte__	143
YDR514C	YDR514C	1	unassigned	na	142
YDR514C	YDR514C	2	msa	na	283
YDR514C	YDR514C	3	unassigned	na	58
YDR515W	SLF1	1	fold_recogniti	1i3qA	257
YDR515W	SLF1	2	msa	na	106
YDR515W	SLF1	3	msa	na	84
YDR516C	EMI2	1	psiblast	1ig8A_	103
YDR516C	EMI2	2	psiblast	1ig8A_	397
YDR517W	GRH1	1	msa	na	284
YDR517W	GRH1	2	msa	na	88
YDR518W	EUG1	1	psiblast	1mek__	146
YDR518W	EUG1	2	psiblast	1bjx__	109
YDR518W	EUG1	3	msa	na	75

YDR518W	EUG1	4	psiblast	1dbyA_	187
YDR519W	FPR2	1	psiblast	1yat__	135
YDR520C	YDR520C	1	psiblast	1hwtC_	151
YDR520C	YDR520C	2	unassigned	na	191
YDR520C	YDR520C	3	fold_recogniti	1qoyA	299
YDR520C	YDR520C	4	fold_recogniti	1qoyA	40
YDR520C	YDR520C	5	unassigned	na	91
YDR521W	YDR521W	1	unassigned	na	111
YDR522C	SPS2	1	unassigned	na	62
YDR522C	SPS2	2	psiblast	1h6uA_	72
YDR522C	SPS2	3	psiblast	1jl5A_	316
YDR522C	SPS2	4	unassigned	na	52
YDR523C	SPS1	1	psiblast	1f3mC_	95
YDR523C	SPS1	2	psiblast	1f3mC_	210
YDR523C	SPS1	3	psiblast	5tnc__	89
YDR523C	SPS1	4	psiblast	5tnc__	96
YDR524C	AGE1	1	psiblast	1eazA_	152
YDR524C	AGE1	2	psiblast	1dcqA_	142
YDR524C	AGE1	3	psiblast	1dcqA_	120
YDR524C	AGE1	4	unassigned	na	68
YDR524C-B	YDR524C-B	1	unassigned	na	66
YDR524W-A	YDR524W-A	1	unassigned	na	29
YDR525W	API2	1	unassigned	na	109
YDR525W-A	SNA2	1	pfam	PF01679	79
YDR526C	YDR526C	1	unassigned	na	156
YDR527W	RBA50	1	fold_recogniti	1b3uA	138
YDR527W	RBA50	2	fold_recogniti	1b3uA	151
YDR527W	RBA50	3	fold_recogniti	1b3uA	150
YDR528W	HLR1	1	msa	na	308
YDR528W	HLR1	2	unassigned	na	115
YDR529C	QCR7	1	psiblast	1kyoG_	127
YDR530C	APA2	1	fold_recogniti	1gupA	325
YDR531W	YDR531W	1	pfam	PF03630	367
YDR532C	YDR532C	1	unassigned	na	131
YDR532C	YDR532C	2	fold_recogniti	1qoyA	208
YDR532C	YDR532C	3	fold_recogniti	1qoyA	46
YDR533C	HSP31	1	psiblast	1g2iA_	237
YDR534C	FIT1	1	msa	na	259
YDR534C	FIT1	2	fold_recogniti	1m2vB	269
YDR535C	YDR535C	1	unassigned	na	166
YDR536W	STL1	1	psiblast	1ja5A_	429
YDR536W	STL1	2	psiblast	1ja5A_	140
YDR537C	YDR537C	1	unassigned	na	201
YDR538W	PAD1	1	psiblast	1e20A_	242
YDR539W	YDR539W	1	pfam	PF01977	503
YDR540C	YDR540C	1	unassigned	na	179
YDR541C	YDR541C	1	psiblast	1keuA_	161
YDR541C	YDR541C	2	psiblast	1keuA_	116

YDR541C	YDR541C	3	psiblast	1keuA_	67
YDR542W	YDR542W	1	pfam	PF00660	120
YDR543C	YDR543C	1	unassigned	na	99
YDR544C	YDR544C	1	unassigned	na	142
YDR545W	YRF1-1	1	msa	na	141
YDR545W	YRF1-1	2	fold_recogniti	1mukA_	600
YDR545W	YRF1-1	3	psiblast	1fuuA_	435
YDR545W	YRF1-1	4	fold_recogniti	1m2vB	59
YDR545W	YRF1-1	5	fold_recogniti	1bziA	250
YDR545W	YRF1-1	6	fold_recogniti	1itwA_	311
YEL001C	YEL001C	1	unassigned	na	225
YEL002C	WBP1	1	pfam	PF03345	430
YEL003W	GIM4	1	psiblast	1fxkB_	123
YEL004W	YEA4	1	msa	na	161
YEL004W	YEA4	2	msa	na	181
YEL005C	VAB2	1	fold_recogniti	1qoyA	163
YEL005C	VAB2	2	fold_recogniti	1qoyA	44
YEL005C	VAB2	3	unassigned	na	75
YEL006W	YEL006W	1	fold_recogniti	1is2A	335
YEL007W	YEL007W	1	msa	na	111
YEL007W	YEL007W	2	fold_recogniti	1l8mA	138
YEL007W	YEL007W	3	fold_recogniti	1i3qA	365
YEL007W	YEL007W	4	unassigned	na	52
YEL008W	YEL008W	1	unassigned	na	126
YEL009C	GCN4	1	msa	na	199
YEL009C	GCN4	2	psiblast	2dgcA_	82
YEL010W	YEL010W	1	unassigned	na	116
YEL011W	GLC3	1	psiblast	1eh9A_	147
YEL011W	GLC3	2	psiblast	1eh9A_	292
YEL011W	GLC3	3	psiblast	1eh9A_	189
YEL011W	GLC3	4	psiblast	1pamA_	76
YEL012W	UBC8	1	psiblast	1qcqA_	161
YEL012W	UBC8	2	unassigned	na	57
YEL013W	VAC8	1	psiblast	3bct__	86
YEL013W	VAC8	2	psiblast	3bct__	118
YEL013W	VAC8	3	psiblast	3bct__	89
YEL013W	VAC8	4	psiblast	3bct__	41
YEL013W	VAC8	5	psiblast	3bct__	107
YEL013W	VAC8	6	psiblast	1bk6A_	137
YEL014C	YEL014C	1	unassigned	na	101
YEL015W	EDC3	1	unassigned	na	265
YEL015W	EDC3	2	psiblast	1jztA_	286
YEL016C	YEL016C	1	unassigned	na	68
YEL016C	YEL016C	2	psiblast	1e1zP_	425
YEL017C-A	PMP2	1	unassigned	na	43
YEL017W	GTT3	1	unassigned	na	337
YEL018W	EAF5	1	unassigned	na	279
YEL019C	MMS21	1	unassigned	na	166

YEL019C	MMS21	2	pfam	PF02891	101
YEL020C	YEL020C	1	psiblast	1powA_	185
YEL020C	YEL020C	2	psiblast	1powA_	169
YEL020C	YEL020C	3	psiblast	1powA_	206
YEL020W-A	TIM9	1	pfam	PF02953	87
YEL021W	URA3	1	psiblast	1dqwA_	267
YEL022W	GEA2	1	msa	na	545
YEL022W	GEA2	2	psiblast	1ku1A_	81
YEL022W	GEA2	3	psiblast	1ku1A_	166
YEL022W	GEA2	4	fold_recogniti	1qgkA	322
YEL022W	GEA2	5	fold_recogniti	1qgkA	206
YEL022W	GEA2	6	fold_recogniti	1qgkA	84
YEL022W	GEA2	7	fold_recogniti	1ao6A	55
YEL023C	YEL023C	1	fold_recogniti	1cudA	174
YEL023C	YEL023C	2	unassigned	na	508
YEL024W	RIP1	1	psiblast	1ezvE_	88
YEL024W	RIP1	2	psiblast	1ezvE_	127
YEL025C	YEL025C	1	unassigned	na	1188
YEL026W	SNU13	1	psiblast	1e7kA_	126
YEL027W	CUP5	1	pfam	PF00137	81
YEL027W	CUP5	2	pfam	PF00137	79
YEL028W	YEL028W	1	unassigned	na	153
YEL029C	BUD16	1	psiblast	1jxhA_	312
YEL030W	ECM10	1	psiblast	1dkgD_	210
YEL030W	ECM10	2	psiblast	1dkgD_	118
YEL030W	ECM10	3	psiblast	1dkgD_	77
YEL030W	ECM10	4	psiblast	1dkyA_	146
YEL030W	ECM10	5	psiblast	1dkyA_	93
YEL031W	SPF1	1	unassigned	na	141
YEL031W	SPF1	2	psiblast	1eulA_	178
YEL031W	SPF1	3	psiblast	1eulA_	357
YEL031W	SPF1	4	psiblast	1eulA_	236
YEL031W	SPF1	5	psiblast	1eulA_	180
YEL031W	SPF1	6	msa	na	123
YEL032W	MCM3	1	msa	na	158
YEL032W	MCM3	2	pfam	PF00493	208
YEL032W	MCM3	3	psiblast	1g8pA_	229
YEL032W	MCM3	4	psiblast	1g8pA_	74
YEL032W	MCM3	5	fold_recogniti	1g8pA	110
YEL032W	MCM3	6	unassigned	na	192
YEL033W	YEL033W	1	unassigned	na	139
YEL034W	HYP2	1	psiblast	1fh4A_	85
YEL034W	HYP2	2	psiblast	1fh4A_	72
YEL035C	UTR5	1	unassigned	na	166
YEL036C	ANP1	1	pfam	PF03452	426
YEL036C	ANP1	2	unassigned	na	74
YEL037C	RAD23	1	psiblast	1aarA_	108
YEL037C	RAD23	2	psiblast	1ifyA_	110

YEL037C	RAD23	3	msa	na	127
YEL037C	RAD23	4	psiblast	1f4iA_	53
YEL038W	UTR4	1	psiblast	1jud__	174
YEL038W	UTR4	2	psiblast	1jud__	67
YEL039C	CYC7	1	psiblast	1yea__	113
YEL040W	UTR2	1	psiblast	1gbg__	124
YEL040W	UTR2	2	psiblast	1axkA_	183
YEL040W	UTR2	3	psiblast	1axkA_	160
YEL041W	YEL041W	1	unassigned	na	99
YEL041W	YEL041W	2	pfam	PF01513	344
YEL041W	YEL041W	3	unassigned	na	52
YEL042W	GDA1	1	fold_recogniti	1bg3A	277
YEL042W	GDA1	2	msa	na	241
YEL043W	YEL043W	1	psiblast	1i84S_	252
YEL043W	YEL043W	2	psiblast	1i84S_	267
YEL043W	YEL043W	3	psiblast	1cii__	202
YEL043W	YEL043W	4	unassigned	na	235
YEL044W	IES6	1	unassigned	na	166
YEL045C	YEL045C	1	unassigned	na	141
YEL046C	GLY1	1	psiblast	1jg8A_	138
YEL046C	GLY1	2	psiblast	1jg8A_	249
YEL047C	YEL047C	1	psiblast	1qo8A_	316
YEL047C	YEL047C	2	psiblast	1qo8A_	154
YEL048C	YEL048C	1	unassigned	na	152
YEL049W	PAU2	1	pfam	PF00660	120
YEL050C	RML2	1	unassigned	na	87
YEL050C	RML2	2	psiblast	1lnrA_	306
YEL051W	VMA8	1	fold_recogniti	1av1A	256
YEL052W	AFG1	1	psiblast	1e32A_	252
YEL052W	AFG1	2	psiblast	1e32A_	104
YEL052W	AFG1	3	msa	na	153
YEL053C	MAK10	1	msa	na	502
YEL053C	MAK10	2	unassigned	na	231
YEL055C	POL5	1	msa	na	694
YEL055C	POL5	2	msa	na	174
YEL055C	POL5	3	unassigned	na	154
YEL056W	HAT2	1	psiblast	1gg2B_	401
YEL057C	YEL057C	1	unassigned	na	233
YEL058W	PCM1	1	psiblast	1kfiA_	96
YEL058W	PCM1	2	psiblast	1k2yX_	110
YEL058W	PCM1	3	psiblast	1k2yX_	170
YEL058W	PCM1	4	psiblast	1k2yX_	96
YEL058W	PCM1	5	psiblast	1k2yX_	85
YEL059C-A	SOM1	1	unassigned	na	74
YEL059W	YEL059W	1	unassigned	na	102
YEL060C	PRB1	1	unassigned	na	111
YEL060C	PRB1	2	psiblast	1scjB_	171
YEL060C	PRB1	3	psiblast	1ic6A_	353

YEL061C	CIN8	1	unassigned	na	64
YEL061C	CIN8	2	msa	na	120
YEL061C	CIN8	3	psiblast	1ii6A_	363
YEL061C	CIN8	4	unassigned	na	188
YEL061C	CIN8	5	psiblast	1i84S_	303
YEL062W	NPR2	1	msa	na	378
YEL062W	NPR2	2	unassigned	na	246
YEL063C	CAN1	1	unassigned	na	73
YEL063C	CAN1	2	pfam	PF00324	517
YEL064C	AVT2	1	pfam	PF01490	480
YEL065W	SIT1	1	psiblast	1ja5A_	268
YEL065W	SIT1	2	unassigned	na	75
YEL065W	SIT1	3	msa	na	285
YEL066W	HPA3	1	psiblast	1qsmA_	179
YEL067C	YEL067C	1	unassigned	na	195
YEL068C	YEL068C	1	unassigned	na	110
YEL069C	HXT13	1	psiblast	1ja5A_	462
YEL069C	HXT13	2	psiblast	1ja5A_	102
YEL070W	YEL070W	1	pfam	PF01232	296
YEL070W	YEL070W	2	fold_recogniti	1n1bA	206
YEL071W	DLD3	1	psiblast	1diiA_	284
YEL071W	DLD3	2	psiblast	1diiA_	212
YEL072W	RMD6	1	unassigned	na	231
YEL073C	YEL073C	1	unassigned	na	107
YEL074W	YEL074W	1	unassigned	na	112
YEL075C	YEL075C	1	unassigned	na	122
YEL075W-A	YEL075W-A	1	unassigned	na	203
YEL076C-B	1728	1	msa	na	216
YEL077C	YEL077C	1	msa	na	112
YEL077C	YEL077C	2	psiblast	1fuuA_	499
YEL077C	YEL077C	3	unassigned	na	66
YEL077C	YEL077C	4	fold_recogniti	1ldvA	230
YEL077C	YEL077C	5	unassigned	na	370
YER001W	MNN1	1	unassigned	na	233
YER001W	MNN1	2	msa	na	529
YER002W	NOP16	1	unassigned	na	231
YER003C	PMI40	1	psiblast	1pmi__	429
YER004W	FMP52	1	psiblast	1bxkB_	231
YER005W	YND1	1	pfam	PF01150	499
YER005W	YND1	2	unassigned	na	131
YER006C-A	YER006C-A	1	unassigned	na	105
YER006W	NUG1	1	unassigned	na	83
YER006W	NUG1	2	psiblast	3ranA_	185
YER006W	NUG1	3	psiblast	1egaA_	252
YER007C-A	YER007C-A	1	psiblast	1it7A_	181
YER007W	PAC2	1	fold_recogniti	1lplA	71
YER007W	PAC2	2	psiblast	2bnh__	91
YER007W	PAC2	3	psiblast	2bnh__	52

YER007W	PAC2	4	psiblast	2bnh__	51
YER007W	PAC2	5	psiblast	2bnh__	105
YER007W	PAC2	6	psiblast	2bnh__	148
YER008C	SEC3	1	msa	na	160
YER008C	SEC3	2	fold_recogniti	1lu7A	147
YER008C	SEC3	3	fold_recogniti	1lu7A	75
YER008C	SEC3	4	psiblast	1i84S_	395
YER008C	SEC3	5	msa	na	191
YER008C	SEC3	6	unassigned	na	368
YER009W	NTF2	1	psiblast	1gy7A_	125
YER010C	YER010C	1	pfam	PF03737	234
YER011W	TIR1	1	fold_recogniti	1deqA	254
YER012W	PRE1	1	psiblast	1fntK_	198
YER013W	PRP22	1	msa	na	122
YER013W	PRP22	2	psiblast	1sro__	160
YER013W	PRP22	3	msa	na	150
YER013W	PRP22	4	psiblast	1gm5A_	229
YER013W	PRP22	5	psiblast	1gm5A_	172
YER013W	PRP22	6	msa	na	312
YER014C-A	BUD25	1	unassigned	na	153
YER014W	HEM14	1	psiblast	1h8qA_	539
YER015W	FAA2	1	unassigned	na	77
YER015W	FAA2	2	psiblast	1ba3__	322
YER015W	FAA2	3	psiblast	1ba3__	345
YER016W	BIM1	1	pfam	PF00307	122
YER016W	BIM1	2	unassigned	na	66
YER016W	BIM1	3	pfam	PF03271	156
YER017C	AFG3	1	psiblast	1e32A_	210
YER017C	AFG3	2	psiblast	1e32A_	74
YER017C	AFG3	3	psiblast	1e32A_	175
YER017C	AFG3	4	psiblast	1e32A_	113
YER017C	AFG3	5	msa	na	189
YER018C	SPC25	1	psiblast	1gk4A_	151
YER018C	SPC25	2	unassigned	na	70
YER019C-A	SBH2	1	pfam	PF03911	88
YER019W	ISC1	1	psiblast	1hd7A_	346
YER019W	ISC1	2	unassigned	na	131
YER020W	GPA2	1	unassigned	na	67
YER020W	GPA2	2	psiblast	1agrA_	259
YER020W	GPA2	3	psiblast	1agrA_	123
YER021W	RPN3	1	msa	na	109
YER021W	RPN3	2	fold_recogniti	1hz4A	206
YER021W	RPN3	3	fold_recogniti	1hz4A	132
YER021W	RPN3	4	unassigned	na	76
YER022W	SRB4	1	msa	na	342
YER022W	SRB4	2	unassigned	na	73
YER022W	SRB4	3	msa	na	272
YER023W	PRO3	1	psiblast	1f12A_	286

YER024W	YAT2	1	pfam	PF00755	780
YER024W	YAT2	2	unassigned	na	143
YER025W	GCD11	1	unassigned	na	79
YER025W	GCD11	2	psiblast	1kk3A_	226
YER025W	GCD11	3	psiblast	1kk3A_	119
YER025W	GCD11	4	psiblast	1kk3A_	103
YER026C	CHO1	1	unassigned	na	109
YER026C	CHO1	2	pfam	PF01066	167
YER027C	GAL83	1	unassigned	na	130
YER027C	GAL83	2	psiblast	1eh9A_	95
YER027C	GAL83	3	psiblast	1eh9A_	192
YER028C	MIG3	1	psiblast	1lu6A_	110
YER028C	MIG3	2	psiblast	1bbo__	69
YER028C	MIG3	3	msa	na	112
YER028C	MIG3	4	unassigned	na	103
YER029C	SMB1	1	psiblast	1d3bB_	131
YER029C	SMB1	2	unassigned	na	65
YER030W	YER030W	1	unassigned	na	160
YER031C	YPT31	1	psiblast	1g17A_	223
YER032W	FIR1	1	unassigned	na	449
YER032W	FIR1	2	fold_recogniti	1ldvA	56
YER032W	FIR1	3	fold_recogniti	1m2vB	262
YER032W	FIR1	4	unassigned	na	158
YER033C	ZRG8	1	unassigned	na	221
YER033C	ZRG8	2	msa	na	488
YER033C	ZRG8	3	unassigned	na	367
YER034W	YER034W	1	unassigned	na	185
YER035W	EDC2	1	unassigned	na	145
YER036C	YER036C	1	unassigned	na	75
YER036C	YER036C	2	psiblast	1g291_	277
YER036C	YER036C	3	unassigned	na	53
YER036C	YER036C	4	psiblast	1nbd__	205
YER037W	PHM8	1	psiblast	1fezA_	264
YER037W	PHM8	2	psiblast	1fezA_	57
YER038C	KRE29	1	unassigned	na	464
YER038W-A	FMP49	1	unassigned	na	126
YER039C	HVG1	1	msa	na	71
YER039C	HVG1	2	msa	na	178
YER039C-A	YER039C-A	1	unassigned	na	72
YER040W	GLN3	1	msa	na	197
YER040W	GLN3	2	psiblast	1gnf__	84
YER040W	GLN3	3	psiblast	4gatA_	81
YER040W	GLN3	4	msa	na	162
YER040W	GLN3	5	msa	na	206
YER041W	YEN1	1	psiblast	1b43A_	308
YER041W	YEN1	2	psiblast	1b43A_	63
YER041W	YEN1	3	unassigned	na	388
YER042W	MXR1	1	psiblast	1fvaA_	184

YER043C	SAH1	1	psiblast	1b3rA_	287
YER043C	SAH1	2	psiblast	1b3rA_	162
YER044C	ERG28	1	pfam	PF03694	148
YER044C-A	MEI4	1	unassigned	na	408
YER045C	ACA1	1	unassigned	na	121
YER045C	ACA1	2	fold_recogniti	1i3qA	263
YER045C	ACA1	3	fold_recogniti	1fosH	105
YER046W	SPO73	1	unassigned	na	143
YER046W-A	YER046W-A	1	unassigned	na	109
YER047C	SAP1	1	unassigned	na	157
YER047C	SAP1	2	msa	na	166
YER047C	SAP1	3	unassigned	na	81
YER047C	SAP1	4	psiblast	1e32A_	122
YER047C	SAP1	5	psiblast	1e32A_	75
YER047C	SAP1	6	psiblast	1e32A_	189
YER047C	SAP1	7	psiblast	1e32A_	107
YER048C	CAJ1	1	psiblast	1xbl__	144
YER048C	CAJ1	2	psiblast	1c3gA_	143
YER048C	CAJ1	3	msa	na	104
YER048W-A	YER048W-A	1	unassigned	na	94
YER049W	YER049W	1	unassigned	na	136
YER049W	YER049W	2	pfam	PF03171	170
YER049W	YER049W	3	unassigned	na	338
YER050C	RSM18	1	unassigned	na	119
YER050C	RSM18	2	psiblast	1fjgR_	83
YER051W	YER051W	1	pfam	PF00628	93
YER051W	YER051W	2	unassigned	na	191
YER051W	YER051W	3	pfam	PF02373	151
YER051W	YER051W	4	unassigned	na	57
YER052C	HOM3	1	psiblast	1gs5A_	339
YER052C	HOM3	2	msa	na	188
YER053C	YER053C	1	fold_recogniti	1is2A	300
YER054C	GIP2	1	unassigned	na	371
YER054C	GIP2	2	pfam	PF03370	177
YER055C	HIS1	1	pfam	PF01634	243
YER055C	HIS1	2	unassigned	na	54
YER056C	FCY2	1	unassigned	na	72
YER056C	FCY2	2	pfam	PF02133	461
YER056C-A	RPL34A	1	pfam	PF01199	121
YER057C	HMF1	1	psiblast	1jd1A_	129
YER058W	PET117	1	unassigned	na	107
YER059W	PCL6	1	unassigned	na	127
YER059W	PCL6	2	msa	na	181
YER059W	PCL6	3	fold_recogniti	1g3nC	112
YER060W	FCY21	1	unassigned	na	69
YER060W	FCY21	2	pfam	PF02133	459
YER060W-A	FCY22	1	unassigned	na	69
YER060W-A	FCY22	2	pfam	PF02133	461

YER061C	CEM1	1	psiblast	1b3nA_	297
YER061C	CEM1	2	psiblast	1b3nA_	145
YER062C	HOR2	1	psiblast	1qq5A_	192
YER062C	HOR2	2	psiblast	1qq5A_	58
YER063W	THO1	1	pfam	PF02037	55
YER063W	THO1	2	unassigned	na	163
YER064C	YER064C	1	unassigned	na	211
YER064C	YER064C	2	fold_recogniti	1jfzA	233
YER064C	YER064C	3	unassigned	na	61
YER065C	ICL1	1	psiblast	1dquA_	443
YER065C	ICL1	2	psiblast	1dquA_	114
YER066C-A	YER066C-A	1	unassigned	na	166
YER066W	YER066W	1	psiblast	1erjA_	185
YER067C-A	YER067C-A	1	unassigned	na	107
YER067W	YER067W	1	unassigned	na	161
YER068C-A	YER068C-A	1	unassigned	na	143
YER068W	MOT2	1	psiblast	1e4uA_	91
YER068W	MOT2	2	psiblast	3sxlA_	144
YER068W	MOT2	3	msa	na	180
YER068W	MOT2	4	unassigned	na	172
YER069W	ARG5,6	1	unassigned	na	96
YER069W	ARG5,6	2	psiblast	1gs5A_	265
YER069W	ARG5,6	3	msa	na	167
YER069W	ARG5,6	4	psiblast	1gpdG_	145
YER069W	ARG5,6	5	psiblast	1gpdG_	190
YER070W	RNR1	1	psiblast	2r1rA_	201
YER070W	RNR1	2	psiblast	2r1rA_	602
YER070W	RNR1	3	unassigned	na	85
YER071C	YER071C	1	unassigned	na	126
YER072W	VTC1	1	unassigned	na	129
YER073W	ALD5	1	psiblast	1bxsA_	331
YER073W	ALD5	2	psiblast	1bxsA_	189
YER074W	RPS24A	1	pfam	PF01282	135
YER074W-A	YER074W-A	1	unassigned	na	33
YER075C	PTP3	1	unassigned	na	80
YER075C	PTP3	2	psiblast	1hzmA_	168
YER075C	PTP3	3	msa	na	243
YER075C	PTP3	4	psiblast	1gwz__	302
YER075C	PTP3	5	fold_recogniti	1bzcA	135
YER076C	YER076C	1	unassigned	na	302
YER077C	YER077C	1	unassigned	na	135
YER077C	YER077C	2	fold_recogniti	1hz4A	145
YER077C	YER077C	3	fold_recogniti	1hz4A	102
YER077C	YER077C	4	fold_recogniti	1hz4A	153
YER077C	YER077C	5	unassigned	na	153
YER078C	YER078C	1	psiblast	1az9__	240
YER078C	YER078C	2	psiblast	1az9__	271
YER079W	YER079W	1	unassigned	na	210

YER080W	FMP29	1	unassigned	na	627
YER081W	SER3	1	psiblast	1psdA_	174
YER081W	SER3	2	psiblast	1psdA_	295
YER082C	UTP7	1	unassigned	na	92
YER082C	UTP7	2	psiblast	1gg2B_	356
YER082C	UTP7	3	unassigned	na	106
YER083C	RMD7	1	fold_recogniti	1llwA	307
YER084W	YER084W	1	unassigned	na	128
YER085C	YER085C	1	unassigned	na	173
YER086W	ILV1	1	psiblast	1tdj__	187
YER086W	ILV1	2	psiblast	1tdj__	199
YER086W	ILV1	3	psiblast	1tdj__	190
YER087C-A	YER087C-A	1	pfam	PF03911	82
YER087W	YER087W	1	psiblast	1qf6A_	187
YER087W	YER087W	2	psiblast	1evkA_	250
YER087W	YER087W	3	psiblast	1evkA_	139
YER088C	DOT6	1	psiblast	1h88C_	69
YER088C	DOT6	2	psiblast	1h88C_	51
YER088C	DOT6	3	psiblast	1h88C_	53
YER088C	DOT6	4	msa	na	197
YER088C	DOT6	5	fold_recogniti	1iu4A	300
YER089C	PTC2	1	psiblast	1a6q__	376
YER089C	PTC2	2	unassigned	na	88
YER090W	TRP2	1	psiblast	1qdlA_	507
YER091C	MET6	1	msa	na	104
YER091C	MET6	2	unassigned	na	297
YER091C	MET6	3	fold_recogniti	1j93A	133
YER091C	MET6	4	fold_recogniti	1j93A	233
YER091C-A	YER091C-A	1	unassigned	na	73
YER092W	IES5	1	unassigned	na	125
YER093C	TSC11	1	psiblast	1i84S_	52
YER093C	TSC11	2	psiblast	1cii__	49
YER093C	TSC11	3	psiblast	1cii__	110
YER093C	TSC11	4	psiblast	1cii__	256
YER093C	TSC11	5	fold_recogniti	1bkdS	121
YER093C	TSC11	6	unassigned	na	318
YER093C	TSC11	7	msa	na	376
YER093C	TSC11	8	unassigned	na	148
YER093C-A	YER093C-A	1	unassigned	na	137
YER094C	PUP3	1	psiblast	1fntJ_	205
YER095W	RAD51	1	psiblast	1b22A_	126
YER095W	RAD51	2	psiblast	1rea__	274
YER096W	SHC1	1	unassigned	na	152
YER096W	SHC1	2	psiblast	1fchA_	184
YER096W	SHC1	3	psiblast	1fchA_	111
YER096W	SHC1	4	unassigned	na	65
YER097W	YER097W	1	unassigned	na	109
YER098W	UBP9	1	unassigned	na	74

YER098W	UBP9	2	pfam	PF00442	107
YER098W	UBP9	3	unassigned	na	105
YER098W	UBP9	4	msa	na	244
YER098W	UBP9	5	unassigned	na	66
YER098W	UBP9	6	pfam	PF00443	91
YER098W	UBP9	7	unassigned	na	67
YER099C	PRS2	1	psiblast	1dkrA_	175
YER099C	PRS2	2	psiblast	1dkrA_	143
YER100W	UBC6	1	psiblast	1qcqA_	178
YER100W	UBC6	2	unassigned	na	72
YER101C	AST2	1	psiblast	1tehA_	282
YER101C	AST2	2	psiblast	1tehA_	148
YER103W	SSA4	1	psiblast	1hjoA_	191
YER103W	SSA4	2	psiblast	1hjoA_	115
YER103W	SSA4	3	psiblast	1hjoA_	75
YER103W	SSA4	4	psiblast	1ckrA_	173
YER103W	SSA4	5	fold_recogniti	1dkxA	88
YER104W	RTT105	1	unassigned	na	208
YER105C	NUP157	1	unassigned	na	65
YER105C	NUP157	2	pfam	PF03177	1326
YER106W	MAM1	1	unassigned	na	302
YER107C	GLE2	1	psiblast	1erjA_	365
YER109C	FLO8	1	unassigned	na	141
YER110C	KAP123	1	psiblast	1qgkA_	238
YER110C	KAP123	2	psiblast	1qgkA_	190
YER110C	KAP123	3	psiblast	1qgkA_	95
YER110C	KAP123	4	psiblast	1qgkA_	226
YER110C	KAP123	5	fold_recogniti	1gw5B	149
YER110C	KAP123	6	fold_recogniti	1gw5B	215
YER111C	SWI4	1	psiblast	1mb1__	165
YER111C	SWI4	2	msa	na	255
YER111C	SWI4	3	psiblast	1awcB_	90
YER111C	SWI4	4	psiblast	1sw6A_	180
YER111C	SWI4	5	msa	na	403
YER112W	LSM4	1	psiblast	1d3bA_	111
YER112W	LSM4	2	unassigned	na	76
YER113C	YER113C	1	unassigned	na	59
YER113C	YER113C	2	pfam	PF02990	647
YER114C	BOI2	1	psiblast	2ptk__	108
YER114C	BOI2	2	psiblast	2ptk__	100
YER114C	BOI2	3	psiblast	2ptk__	84
YER114C	BOI2	4	unassigned	na	89
YER114C	BOI2	5	fold_recogniti	1m2vB	294
YER114C	BOI2	6	fold_recogniti	1ei3A	90
YER114C	BOI2	7	psiblast	1fhwA_	198
YER114C	BOI2	8	unassigned	na	77
YER115C	SPR6	1	unassigned	na	191
YER116C	SLX8	1	msa	na	198

YER116C	SLX8	2	psiblast	1jm7A_	76
YER118C	SHO1	1	unassigned	na	253
YER118C	SHO1	2	psiblast	1g83A_	114
YER119C	AVT6	1	pfam	PF01490	448
YER119C-A	YER119C-A	1	unassigned	na	123
YER120W	SCS2	1	psiblast	1grwA_	162
YER120W	SCS2	2	unassigned	na	82
YER121W	YER121W	1	unassigned	na	114
YER122C	GLO3	1	psiblast	1dcqA_	163
YER122C	GLO3	2	unassigned	na	85
YER122C	GLO3	3	msa	na	181
YER122C	GLO3	4	unassigned	na	64
YER123W	YCK3	1	psiblast	1csn__	89
YER123W	YCK3	2	psiblast	1csn__	91
YER123W	YCK3	3	psiblast	1csn__	212
YER123W	YCK3	4	unassigned	na	132
YER124C	DSE1	1	unassigned	na	573
YER125W	RSP5	1	psiblast	1dqvA_	199
YER125W	RSP5	2	psiblast	1f8aB_	115
YER125W	RSP5	3	psiblast	1i5hW_	58
YER125W	RSP5	4	fold_recogniti	1i5hW	78
YER125W	RSP5	5	psiblast	1c4zA_	44
YER125W	RSP5	6	psiblast	1c4zA_	141
YER125W	RSP5	7	psiblast	1c4zA_	174
YER126C	NSA2	1	msa	na	114
YER126C	NSA2	2	unassigned	na	147
YER127W	LCP5	1	msa	na	129
YER127W	LCP5	2	unassigned	na	228
YER128W	YER128W	1	msa	na	101
YER128W	YER128W	2	unassigned	na	102
YER129W	PAK1	1	unassigned	na	96
YER129W	PAK1	2	psiblast	1a06__	156
YER129W	PAK1	3	psiblast	1a06__	244
YER129W	PAK1	4	psiblast	1iq5A_	73
YER129W	PAK1	5	psiblast	1iq5A_	75
YER129W	PAK1	6	fold_recogniti	1jl5A	152
YER129W	PAK1	7	fold_recogniti	1jl5A	346
YER130C	YER130C	1	msa	na	177
YER130C	YER130C	2	psiblast	1a1iA_	58
YER130C	YER130C	3	unassigned	na	107
YER130C	YER130C	4	psiblast	2drpA_	101
YER131W	RPS26B	1	pfam	PF01283	119
YER132C	PMD1	1	unassigned	na	129
YER132C	PMD1	2	pfam	PF01344	108
YER132C	PMD1	3	unassigned	na	1516
YER133W	GLC7	1	psiblast	1it6A_	312
YER134C	YER134C	1	psiblast	1l7mA_	130
YER134C	YER134C	2	psiblast	1l7mA_	48

YER135C	YER135C	1	unassigned	na	130
YER136W	GDI1	1	psiblast	1gnd__	451
YER137C	YER137C	1	unassigned	na	148
YER138W-A	YER138W-A	1	unassigned	na	34
YER139C	YER139C	1	msa	na	142
YER139C	YER139C	2	unassigned	na	84
YER140W	YER140W	1	unassigned	na	139
YER140W	YER140W	2	msa	na	74
YER140W	YER140W	3	msa	na	202
YER140W	YER140W	4	unassigned	na	141
YER141W	COX15	1	unassigned	na	64
YER141W	COX15	2	pfam	PF02628	422
YER142C	MAG1	1	psiblast	1dizA_	296
YER143W	DDI1	1	psiblast	1aarA_	82
YER143W	DDI1	2	unassigned	na	69
YER143W	DDI1	3	psiblast	2aidA_	153
YER143W	DDI1	4	psiblast	1rvnA_	83
YER143W	DDI1	5	psiblast	1dv0A_	41
YER144C	UBP5	1	unassigned	na	147
YER144C	UBP5	2	pfam	PF00581	130
YER144C	UBP5	3	fold_recogniti	1ldvA	211
YER144C	UBP5	4	unassigned	na	52
YER144C	UBP5	5	msa	na	130
YER144C	UBP5	6	msa	na	69
YER144C	UBP5	7	pfam	PF00443	66
YER145C	FTR1	1	pfam	PF03239	323
YER145C	FTR1	2	unassigned	na	81
YER145C-A	YER145C-A	1	unassigned	na	145
YER146W	LSM5	1	psiblast	1i81A_	93
YER147C	SCC4	1	fold_recogniti	1e8yA	218
YER147C	SCC4	2	fold_recogniti	1e8yA	154
YER147C	SCC4	3	fold_recogniti	1e8yA	252
YER148W	SPT15	1	psiblast	1tbaB_	151
YER148W	SPT15	2	psiblast	1tbaB_	89
YER148W-A	YER148W-A	1	unassigned	na	192
YER149C	PEA2	1	fold_recogniti	1e6vA	137
YER149C	PEA2	2	fold_recogniti	1e6vA	283
YER150W	SPI1	1	unassigned	na	148
YER151C	UBP3	1	fold_recogniti	1i3qA	253
YER151C	UBP3	2	fold_recogniti	1ldvA	163
YER151C	UBP3	3	pfam	PF00442	83
YER151C	UBP3	4	unassigned	na	76
YER151C	UBP3	5	msa	na	237
YER151C	UBP3	6	pfam	PF00443	100
YER152C	YER152C	1	psiblast	1iaxA_	118
YER152C	YER152C	2	psiblast	1iaxA_	325
YER153C	PET122	1	msa	na	254
YER154W	OXA1	1	unassigned	na	92

YER154W	OXA1	2	pfam	PF02096	243
YER154W	OXA1	3	unassigned	na	67
YER155C	BEM2	1	unassigned	na	575
YER155C	BEM2	2	pfam	PF00617	235
YER155C	BEM2	3	unassigned	na	294
YER155C	BEM2	4	pfam	PF00618	84
YER155C	BEM2	5	unassigned	na	426
YER155C	BEM2	6	msa	na	300
YER155C	BEM2	7	psiblast	1f7cA_	137
YER155C	BEM2	8	psiblast	1f7cA_	116
YER156C	YER156C	1	pfam	PF03690	338
YER157W	COG3	1	fold_recogniti	1qgkA	414
YER157W	COG3	2	fold_recogniti	1qgkA	119
YER157W	COG3	3	fold_recogniti	1qgkA	122
YER157W	COG3	4	fold_recogniti	1qgkA	146
YER158C	YER158C	1	fold_recogniti	1i3qA	417
YER158C	YER158C	2	unassigned	na	156
YER159C	BUR6	1	psiblast	1jfiA_	142
YER161C	SPT2	1	unassigned	na	93
YER161C	SPT2	2	msa	na	240
YER162C	RAD4	1	unassigned	na	244
YER162C	RAD4	2	pfam	PF03835	450
YER162C	RAD4	3	unassigned	na	60
YER163C	YER163C	1	msa	na	232
YER164W	CHD1	1	unassigned	na	133
YER164W	CHD1	2	pfam	PF00385	128
YER164W	CHD1	3	fold_recogniti	1gm5A	80
YER164W	CHD1	4	fold_recogniti	1gm5A	219
YER164W	CHD1	5	fold_recogniti	1hv8A	66
YER164W	CHD1	6	psiblast	1hv8A_	68
YER164W	CHD1	7	psiblast	1d9xA_	233
YER164W	CHD1	8	msa	na	178
YER164W	CHD1	9	msa	na	161
YER164W	CHD1	10	unassigned	na	95
YER164W	CHD1	11	msa	na	107
YER165C-A	YER165C-A	1	unassigned	na	118
YER165W	PAB1	1	psiblast	1cvjA_	116
YER165W	PAB1	2	psiblast	1cvjA_	99
YER165W	PAB1	3	psiblast	1l3kA_	92
YER165W	PAB1	4	psiblast	1l3kA_	125
YER165W	PAB1	5	unassigned	na	53
YER165W	PAB1	6	psiblast	1ifwA_	92
YER166W	DNF1	1	msa	na	282
YER166W	DNF1	2	psiblast	1eulA_	182
YER166W	DNF1	3	psiblast	1eulA_	389
YER166W	DNF1	4	psiblast	1eulA_	273
YER166W	DNF1	5	psiblast	1eulA_	292
YER166W	DNF1	6	msa	na	153

YER167W	BCK2	1	unassigned	na	851
YER168C	CCA1	1	unassigned	na	123
YER168C	CCA1	2	pfam	PF01743	227
YER168C	CCA1	3	unassigned	na	196
YER169W	RPH1	1	pfam	PF02375	59
YER169W	RPH1	2	msa	na	139
YER169W	RPH1	3	pfam	PF02373	180
YER169W	RPH1	4	unassigned	na	53
YER169W	RPH1	5	psiblast	1ubdC_	156
YER169W	RPH1	6	msa	na	89
YER169W	RPH1	7	psiblast	1meyG_	120
YER170W	ADK2	1	psiblast	2ak3A_	225
YER171W	RAD3	1	psiblast	1hv8A_	221
YER171W	RAD3	2	psiblast	1hv8A_	168
YER171W	RAD3	3	unassigned	na	52
YER171W	RAD3	4	fold_recogniti	1fuuA	284
YER171W	RAD3	5	unassigned	na	53
YER172C	BRR2	1	msa	na	131
YER172C	BRR2	2	unassigned	na	80
YER172C	BRR2	3	msa	na	253
YER172C	BRR2	4	psiblast	1hv8A_	234
YER172C	BRR2	5	psiblast	1hv8A_	205
YER172C	BRR2	6	psiblast	1d9xA_	134
YER172C	BRR2	7	unassigned	na	281
YER172C	BRR2	8	msa	na	466
YER172C	BRR2	9	unassigned	na	136
YER172C	BRR2	10	psiblast	1jj2X_	167
YER172C	BRR2	11	unassigned	na	76
YER172C-A	YER172C-A	1	unassigned	na	126
YER173W	RAD24	1	psiblast	1jr3A_	221
YER173W	RAD24	2	psiblast	1jr3A_	198
YER173W	RAD24	3	unassigned	na	240
YER174C	GRX4	1	psiblast	1quwA_	137
YER174C	GRX4	2	psiblast	1b4qA_	107
YER175C	TMT1	1	psiblast	1kp9A_	195
YER175C	TMT1	2	psiblast	1kp9A_	104
YER176W	ECM32	1	fold_recogniti	1i3qA	382
YER176W	ECM32	2	fold_recogniti	1l8mA	79
YER176W	ECM32	3	unassigned	na	171
YER176W	ECM32	4	psiblast	1pjr__	489
YER177W	BMH1	1	psiblast	1a37A_	115
YER177W	BMH1	2	psiblast	1a37A_	152
YER178W	PDA1	1	psiblast	1dtwA_	443
YER179W	DMC1	1	psiblast	1b22A_	54
YER179W	DMC1	2	psiblast	1rea__	280
YER180C	ISC10	1	unassigned	na	267
YER181C	YER181C	1	unassigned	na	107
YER182W	FMP10	1	unassigned	na	80

YER182W	FMP10	2	msa	na	164
YER183C	FAU1	1	pfam	PF01812	211
YER184C	YER184C	1	psiblast	1hwtD_	81
YER184C	YER184C	2	unassigned	na	104
YER184C	YER184C	3	msa	na	274
YER184C	YER184C	4	unassigned	na	335
YER185W	YER185W	1	unassigned	na	170
YER185W	YER185W	2	msa	na	133
YER186C	YER186C	1	unassigned	na	306
YER187W	YER187W	1	unassigned	na	141
YER188C-A	YER188C-A	1	unassigned	na	99
YER188W	YER188W	1	unassigned	na	239
YER189W	YER189W	1	unassigned	na	122
YER190W	YRF1-2	1	msa	na	63
YER190W	YRF1-2	2	fold_recogniti	1llwA	330
YER190W	YRF1-2	3	unassigned	na	187
YER190W	YRF1-2	4	psiblast	1fuuA_	499
YER190W	YRF1-2	5	fold_recogniti	1m2vB	256
YER190W	YRF1-2	6	fold_recogniti	1qoyA	346
YFL001W	DEG1	1	unassigned	na	84
YFL001W	DEG1	2	psiblast	1dj0A_	159
YFL001W	DEG1	3	psiblast	1dj0A_	111
YFL001W	DEG1	4	unassigned	na	88
YFL002C	SPB4	1	psiblast	1hv8A_	231
YFL002C	SPB4	2	psiblast	1hv8A_	157
YFL002C	SPB4	3	psiblast	1d9xA_	131
YFL002C	SPB4	4	unassigned	na	87
YFL003C	MSH4	1	psiblast	1e3mA_	84
YFL003C	MSH4	2	psiblast	1e3mA_	178
YFL003C	MSH4	3	psiblast	1e3mA_	319
YFL003C	MSH4	4	psiblast	1e3mA_	220
YFL003C	MSH4	5	psiblast	1e3mA_	77
YFL004W	VTC2	1	pfam	PF03105	184
YFL004W	VTC2	2	unassigned	na	54
YFL004W	VTC2	3	msa	na	590
YFL005W	SEC4	1	psiblast	1g17A_	215
YFL006W	1947	1	fold_recogniti	1b3uA	254
YFL007W	BLM3	1	unassigned	na	1804
YFL008W	SMC1	1	fold_recogniti	1ii8A	49
YFL008W	SMC1	2	psiblast	1i84S_	317
YFL008W	SMC1	3	psiblast	2tmaA_	79
YFL008W	SMC1	4	psiblast	2tmaA_	54
YFL008W	SMC1	5	psiblast	1gxlA_	77
YFL008W	SMC1	6	psiblast	1cii__	150
YFL008W	SMC1	7	psiblast	1cii__	290
YFL008W	SMC1	8	psiblast	1e69A_	209
YFL009W	CDC4	1	unassigned	na	107
YFL009W	CDC4	2	psiblast	1gxrA_	343

YFL009W	CDC4	3	psiblast	1erjA_	151
YFL009W	CDC4	4	fold_recogniti	1jjuB	178
YFL010C	WWM1	1	psiblast	1f8aB_	52
YFL010C	WWM1	2	unassigned	na	159
YFL010W-A	AUA1	1	unassigned	na	94
YFL011W	HXT10	1	psiblast	1ja5A_	453
YFL011W	HXT10	2	psiblast	1ja5A_	93
YFL012W	YFL012W	1	unassigned	na	148
YFL012W-A	YFL012W-A	1	unassigned	na	124
YFL013C	IES1	1	unassigned	na	103
YFL013C	IES1	2	msa	na	374
YFL013C	IES1	3	unassigned	na	215
YFL013W-A	YFL013W-A	1	unassigned	na	267
YFL014W	HSP12	1	unassigned	na	109
YFL015C	YFL015C	1	unassigned	na	164
YFL016C	MDJ1	1	psiblast	1xbl__	180
YFL016C	MDJ1	2	psiblast	1exkA_	116
YFL016C	MDJ1	3	psiblast	1c3gA_	215
YFL017C	GNA1	1	psiblast	1i1dA_	159
YFL017W-A	SMX2	1	psiblast	1i81A_	77
YFL018C	LPD1	1	psiblast	1jehA_	200
YFL018C	LPD1	2	psiblast	1jehA_	183
YFL018C	LPD1	3	psiblast	1jehA_	116
YFL019C	YFL019C	1	unassigned	na	117
YFL020C	PAU5	1	pfam	PF00660	122
YFL021C-A	YFL021C-A	1	unassigned	na	284
YFL021W	GAT1	1	msa	na	238
YFL021W	GAT1	2	psiblast	1gnf__	58
YFL021W	GAT1	3	psiblast	4gatA_	108
YFL021W	GAT1	4	msa	na	106
YFL022C	FRS2	1	fold_recogniti	1lnwA	114
YFL022C	FRS2	2	psiblast	1b70A_	389
YFL023W	BUD27	1	pfam	PF02996	128
YFL023W	BUD27	2	fold_recogniti	1l8mA	73
YFL023W	BUD27	3	fold_recogniti	1l8mA	79
YFL023W	BUD27	4	fold_recogniti	1l8mA	70
YFL023W	BUD27	5	fold_recogniti	1l8mA	75
YFL023W	BUD27	6	fold_recogniti	1i3qA	231
YFL023W	BUD27	7	unassigned	na	140
YFL024C	EPL1	1	unassigned	na	92
YFL024C	EPL1	2	msa	na	65
YFL024C	EPL1	3	unassigned	na	72
YFL024C	EPL1	4	psiblast	1eqfA_	130
YFL024C	EPL1	5	psiblast	1eqfA_	85
YFL024C	EPL1	6	msa	na	94
YFL024C	EPL1	7	unassigned	na	294
YFL025C	BST1	1	msa	na	396
YFL025C	BST1	2	unassigned	na	633

YFL026W	STE2	1	pfam	PF02116	431
YFL027C	GYP8	1	psiblast	1fkmA_	250
YFL027C	GYP8	2	psiblast	1fkmA_	61
YFL027C	GYP8	3	msa	na	186
YFL028C	CAF16	1	psiblast	1jsqA_	289
YFL029C	CAK1	1	psiblast	1aq1__	81
YFL029C	CAK1	2	psiblast	1aq1__	123
YFL029C	CAK1	3	psiblast	1aq1__	67
YFL029C	CAK1	4	psiblast	1aq1__	97
YFL030W	AGX1	1	psiblast	1ecxA_	115
YFL030W	AGX1	2	psiblast	1ecxA_	270
YFL031W	HAC1	1	psiblast	1i84S_	132
YFL031W	HAC1	2	unassigned	na	106
YFL032W	YFL032W	1	unassigned	na	106
YFL033C	RIM15	1	unassigned	na	403
YFL033C	RIM15	2	fold_recogniti	1i3qA	382
YFL033C	RIM15	3	psiblast	1lg3A_	195
YFL033C	RIM15	4	psiblast	1fotA_	142
YFL033C	RIM15	5	psiblast	1fotA_	218
YFL033C	RIM15	6	unassigned	na	430
YFL034C-A	RPL22B	1	pfam	PF01776	122
YFL034C-B	MOB2	1	unassigned	na	64
YFL034C-B	MOB2	2	pfam	PF03637	223
YFL034W	YFL034W	1	unassigned	na	318
YFL034W	YFL034W	2	msa	na	526
YFL034W	YFL034W	3	unassigned	na	229
YFL036W	RPO41	1	unassigned	na	379
YFL036W	RPO41	2	psiblast	1aroP_	131
YFL036W	RPO41	3	psiblast	1aroP_	316
YFL036W	RPO41	4	psiblast	1aroP_	179
YFL036W	RPO41	5	psiblast	1aroP_	254
YFL036W	RPO41	6	unassigned	na	92
YFL037W	TUB2	1	psiblast	1ffxB_	457
YFL038C	YPT1	1	psiblast	1g17A_	206
YFL039C	ACT1	1	psiblast	2btfA_	174
YFL039C	ACT1	2	psiblast	2btfA_	201
YFL040W	YFL040W	1	psiblast	1ja5A_	429
YFL040W	YFL040W	2	psiblast	1ja5A_	111
YFL041W	FET5	1	psiblast	1kyaA_	366
YFL041W	FET5	2	psiblast	1kyaA_	199
YFL041W	FET5	3	unassigned	na	57
YFL042C	YFL042C	1	msa	na	191
YFL042C	YFL042C	2	pfam	PF02893	117
YFL042C	YFL042C	3	unassigned	na	53
YFL042C	YFL042C	4	msa	na	234
YFL042C	YFL042C	5	unassigned	na	79
YFL044C	YFL044C	1	unassigned	na	94
YFL044C	YFL044C	2	pfam	PF02338	138

YFL044C	YFL044C	3	unassigned	na	69
YFL045C	SEC53	1	pfam	PF03332	254
YFL046W	FMP32	1	msa	na	134
YFL046W	FMP32	2	unassigned	na	73
YFL047W	RGD2	1	fold_recogniti	1av1A	225
YFL047W	RGD2	2	pfam	PF00610	74
YFL047W	RGD2	3	unassigned	na	67
YFL047W	RGD2	4	psiblast	1rgp__	203
YFL047W	RGD2	5	psiblast	1rgp__	145
YFL048C	EMP47	1	psiblast	1gv9A_	265
YFL048C	EMP47	2	msa	na	180
YFL049W	YFL049W	1	msa	na	240
YFL049W	YFL049W	2	unassigned	na	383
YFL050C	ALR2	1	unassigned	na	372
YFL050C	ALR2	2	msa	na	182
YFL050C	ALR2	3	fold_recogniti	1hciA	147
YFL050C	ALR2	4	msa	na	157
YFL051C	YFL051C	1	unassigned	na	160
YFL052W	YFL052W	1	psiblast	1d66A_	43
YFL052W	YFL052W	2	msa	na	307
YFL052W	YFL052W	3	unassigned	na	115
YFL053W	DAK2	1	fold_recogniti	1e1cB	591
YFL054C	YFL054C	1	msa	na	236
YFL054C	YFL054C	2	unassigned	na	90
YFL054C	YFL054C	3	psiblast	1fx8A_	134
YFL054C	YFL054C	4	psiblast	1fx8A_	186
YFL055W	AGP3	1	pfam	PF00324	558
YFL056C	AAD6	1	psiblast	1exbA_	212
YFL057C	AAD16	1	psiblast	1exbA_	152
YFL058W	THI5	1	psiblast	1hpbP_	184
YFL058W	THI5	2	psiblast	1hpbP_	102
YFL058W	THI5	3	unassigned	na	54
YFL059W	SNZ3	1	fold_recogniti	1gt8A	190
YFL059W	SNZ3	2	psiblast	2tpsA_	108
YFL060C	SNO3	1	psiblast	1gpmA_	222
YFL061W	YFL061W	1	pfam	PF01966	225
YFL062W	COS4	1	msa	na	61
YFL062W	COS4	2	pfam	PF00674	107
YFL062W	COS4	3	unassigned	na	73
YFL062W	COS4	4	pfam	PF00674	138
YFL063W	YFL063W	1	unassigned	na	151
YFL064C	YFL064C	1	unassigned	na	174
YFL065C	YFL065C	1	unassigned	na	102
YFL066C	YFL066C	1	psiblast	1fuuA_	392
YFL067W	YFL067W	1	fold_recogniti	1deqA	175
YFL068W	YFL068W	1	unassigned	na	160
YFR001W	LOC1	1	unassigned	na	204
YFR002W	NIC96	1	fold_recogniti	1bgxT	174

YFR002W	NIC96	2	fold_recogniti	1bgxT	436
YFR002W	NIC96	3	fold_recogniti	1bgxT	119
YFR002W	NIC96	4	fold_recogniti	1b8fA	110
YFR003C	YPI1	1	fold_recogniti	1kkqC	155
YFR004W	RPN11	1	pfam	PF01398	159
YFR004W	RPN11	2	unassigned	na	52
YFR004W	RPN11	3	msa	na	95
YFR005C	SAD1	1	msa	na	124
YFR005C	SAD1	2	msa	na	324
YFR006W	YFR006W	1	psiblast	1az9__	227
YFR006W	YFR006W	2	psiblast	1az9__	308
YFR007W	YFR007W	1	psiblast	1esmA_	353
YFR008W	FAR7	1	unassigned	na	221
YFR009W	GCN20	1	unassigned	na	182
YFR009W	GCN20	2	psiblast	1b0uA_	309
YFR009W	GCN20	3	psiblast	1jsqA_	261
YFR010W	UBP6	1	psiblast	1ud7A_	80
YFR010W	UBP6	2	pfam	PF00442	75
YFR010W	UBP6	3	msa	na	270
YFR010W	UBP6	4	pfam	PF00443	74
YFR011C	YFR011C	1	unassigned	na	170
YFR012W	YFR012W	1	msa	na	202
YFR012W-A	YFR012W-A	1	unassigned	na	28
YFR013W	IOC3	1	fold_recogniti	1ldvA	217
YFR013W	IOC3	2	unassigned	na	570
YFR014C	CMK1	1	psiblast	1a06__	144
YFR014C	CMK1	2	psiblast	1a06__	200
YFR014C	CMK1	3	psiblast	1iq5A_	102
YFR015C	GSY1	1	unassigned	na	68
YFR015C	GSY1	2	msa	na	291
YFR015C	GSY1	3	msa	na	297
YFR015C	GSY1	4	unassigned	na	52
YFR016C	YFR016C	1	unassigned	na	73
YFR016C	YFR016C	2	psiblast	1i84S_	352
YFR016C	YFR016C	3	unassigned	na	56
YFR016C	YFR016C	4	fold_recogniti	1i3qA	333
YFR016C	YFR016C	5	fold_recogniti	1l8mA	103
YFR016C	YFR016C	6	fold_recogniti	1l8mA	79
YFR016C	YFR016C	7	fold_recogniti	1l8mA	70
YFR016C	YFR016C	8	fold_recogniti	1l8mA	114
YFR016C	YFR016C	9	unassigned	na	53
YFR017C	YFR017C	1	unassigned	na	195
YFR018C	YFR018C	1	psiblast	1amp__	363
YFR019W	FAB1	1	msa	na	129
YFR019W	FAB1	2	unassigned	na	94
YFR019W	FAB1	3	psiblast	1hyiA_	95
YFR019W	FAB1	4	unassigned	na	303
YFR019W	FAB1	5	psiblast	1a6dA_	506

YFR019W	FAB1	6	psiblast	1a6dA_	118
YFR019W	FAB1	7	unassigned	na	311
YFR019W	FAB1	8	msa	na	350
YFR019W	FAB1	9	psiblast	1bo1A_	301
YFR019W	FAB1	10	fold_recogniti	1bo1A	71
YFR020W	YFR020W	1	unassigned	na	232
YFR021W	ATG18	1	msa	na	79
YFR021W	ATG18	2	psiblast	1gg2B_	52
YFR021W	ATG18	3	psiblast	1erjA_	369
YFR022W	YFR022W	1	msa	na	132
YFR022W	YFR022W	2	psiblast	1jsyA_	133
YFR022W	YFR022W	3	psiblast	1jsyA_	179
YFR022W	YFR022W	4	msa	na	289
YFR023W	PES4	1	msa	na	76
YFR023W	PES4	2	psiblast	1cvjA_	100
YFR023W	PES4	3	psiblast	1fjcA_	97
YFR023W	PES4	4	psiblast	1h6kX_	104
YFR023W	PES4	5	psiblast	1l3kA_	133
YFR023W	PES4	6	msa	na	101
YFR024C-A	LSB3	1	msa	na	362
YFR024C-A	LSB3	2	psiblast	1semA_	89
YFR025C	HIS2	1	unassigned	na	88
YFR025C	HIS2	2	pfam	PF02811	247
YFR026C	YFR026C	1	unassigned	na	169
YFR027W	ECO1	1	msa	na	149
YFR027W	ECO1	2	msa	na	132
YFR028C	CDC14	1	msa	na	85
YFR028C	CDC14	2	psiblast	1larA_	392
YFR028C	CDC14	3	unassigned	na	74
YFR029W	PTR3	1	unassigned	na	678
YFR030W	MET10	1	psiblast	1b0pA_	373
YFR030W	MET10	2	psiblast	1b0pA_	270
YFR030W	MET10	3	psiblast	1amoA_	97
YFR030W	MET10	4	psiblast	1amoA_	295
YFR031C	SMC2	1	psiblast	1ii8A_	213
YFR031C	SMC2	2	psiblast	2tmaA_	69
YFR031C	SMC2	3	psiblast	2tmaA_	82
YFR031C	SMC2	4	psiblast	2tmaA_	62
YFR031C	SMC2	5	psiblast	2tmaA_	61
YFR031C	SMC2	6	psiblast	1gxlA_	73
YFR031C	SMC2	7	psiblast	1gxlA_	64
YFR031C	SMC2	8	psiblast	1gxlA_	63
YFR031C	SMC2	9	psiblast	1i84S_	294
YFR031C	SMC2	10	psiblast	1e69A_	189
YFR031C-A	RPL2A	1	psiblast	1k5yB_	72
YFR031C-A	RPL2A	2	psiblast	1k5yB_	182
YFR032C	YFR032C	1	psiblast	1ha1__	106
YFR032C	YFR032C	2	psiblast	1ha1__	183

YFR032C-A	RPL29	1	pfam	PF01779	59
YFR033C	QCR6	1	unassigned	na	63
YFR033C	QCR6	2	psiblast	1ezvH_	84
YFR034C	PHO4	1	unassigned	na	247
YFR034C	PHO4	2	psiblast	1a0aA_	65
YFR035C	YFR035C	1	unassigned	na	114
YFR036W	CDC26	1	unassigned	na	124
YFR036W-A	YFR036W-A	1	unassigned	na	216
YFR037C	RSC8	1	msa	na	177
YFR037C	RSC8	2	unassigned	na	70
YFR037C	RSC8	3	pfam	PF00569	57
YFR037C	RSC8	4	psiblast	1h8aC_	54
YFR037C	RSC8	5	psiblast	1dg3A_	73
YFR037C	RSC8	6	psiblast	1dg3A_	126
YFR038W	YFR038W	1	unassigned	na	68
YFR038W	YFR038W	2	psiblast	1gm5A_	94
YFR038W	YFR038W	3	psiblast	1gm5A_	260
YFR038W	YFR038W	4	unassigned	na	56
YFR038W	YFR038W	5	psiblast	1hv8A_	300
YFR039C	YFR039C	1	unassigned	na	234
YFR039C	YFR039C	2	fold_recogniti	1jkyA	212
YFR039C	YFR039C	3	fold_recogniti	1jkyA	64
YFR040W	SAP155	1	unassigned	na	106
YFR040W	SAP155	2	msa	na	441
YFR040W	SAP155	3	unassigned	na	53
YFR040W	SAP155	4	msa	na	152
YFR040W	SAP155	5	msa	na	153
YFR041C	ERJ5	1	psiblast	1bq0__	143
YFR041C	ERJ5	2	psiblast	1exkA_	152
YFR042W	YFR042W	1	unassigned	na	200
YFR043C	YFR043C	1	unassigned	na	237
YFR044C	YFR044C	1	psiblast	1cg2A_	312
YFR044C	YFR044C	2	psiblast	1cg2A_	169
YFR045W	YFR045W	1	unassigned	na	85
YFR045W	YFR045W	2	pfam	PF00153	93
YFR046C	CNN1	1	unassigned	na	361
YFR047C	BNA6	1	psiblast	1qapA_	128
YFR047C	BNA6	2	psiblast	1qapA_	61
YFR047C	BNA6	3	psiblast	1qapA_	106
YFR048W	RMD8	1	unassigned	na	124
YFR048W	RMD8	2	msa	na	150
YFR048W	RMD8	3	unassigned	na	85
YFR048W	RMD8	4	pfam	PF02582	303
YFR049W	YMR31	1	unassigned	na	123
YFR050C	PRE4	1	psiblast	1g0uM_	266
YFR051C	RET2	1	psiblast	1gw5S_	85
YFR051C	RET2	2	psiblast	1gw5S_	92
YFR051C	RET2	3	unassigned	na	56

YFR051C	RET2	4	psiblast	1gw5M_	313
YFR052W	RPN12	1	unassigned	na	94
YFR052W	RPN12	2	msa	na	180
YFR053C	HXK1	1	psiblast	1ig8A_	107
YFR053C	HXK1	2	psiblast	1ig8A_	378
YFR054C	YFR054C	1	unassigned	na	192
YFR055W	YFR055W	1	psiblast	1cl2A_	340
YFR056C	YFR056C	1	unassigned	na	122
YFR057W	YFR057W	1	unassigned	na	151
YGL001C	ERG26	1	psiblast	1i3kA_	349
YGL002W	ERP6	1	pfam	PF01105	216
YGL003C	CDH1	1	msa	na	120
YGL003C	CDH1	2	psiblast	1gxrA_	134
YGL003C	CDH1	3	psiblast	1gg2B_	312
YGL004C	RPN14	1	psiblast	1erjA_	417
YGL005C	COG7	1	fold_recogniti	1qoyA	239
YGL005C	COG7	2	fold_recogniti	1qoyA	40
YGL006W	PMC1	1	psiblast	1eulA_	234
YGL006W	PMC1	2	psiblast	1eulA_	449
YGL006W	PMC1	3	psiblast	1eulA_	203
YGL006W	PMC1	4	psiblast	1eulA_	287
YGL006W-A	YGL006W-A	1	unassigned	na	36
YGL007W	YGL007W	1	unassigned	na	125
YGL008C	PMA1	1	psiblast	1eulA_	210
YGL008C	PMA1	2	psiblast	1eulA_	360
YGL008C	PMA1	3	psiblast	1eulA_	198
YGL008C	PMA1	4	psiblast	1eulA_	150
YGL009C	LEU1	1	psiblast	5acn__	550
YGL009C	LEU1	2	psiblast	5acn__	229
YGL010W	YGL010W	1	unassigned	na	174
YGL011C	SCL1	1	psiblast	1jd2G_	252
YGL012W	ERG4	1	pfam	PF01222	473
YGL013C	PDR1	1	psiblast	1d66A_	111
YGL013C	PDR1	2	unassigned	na	309
YGL013C	PDR1	3	msa	na	154
YGL013C	PDR1	4	unassigned	na	494
YGL014W	PUF4	1	unassigned	na	89
YGL014W	PUF4	2	psiblast	1deqA_	127
YGL014W	PUF4	3	fold_recogniti	1deqA	230
YGL014W	PUF4	4	unassigned	na	75
YGL014W	PUF4	5	psiblast	1ib2A_	152
YGL014W	PUF4	6	psiblast	1ib2A_	103
YGL014W	PUF4	7	psiblast	1ib2A_	112
YGL015C	YGL015C	1	unassigned	na	130
YGL016W	KAP122	1	unassigned	na	393
YGL016W	KAP122	2	msa	na	688
YGL017W	ATE1	1	msa	na	353
YGL017W	ATE1	2	unassigned	na	150

YGL018C	JAC1	1	psiblast	1fpoA_	71
YGL018C	JAC1	2	psiblast	1fpoA_	113
YGL019W	CKB1	1	psiblast	1jwhC_	114
YGL019W	CKB1	2	psiblast	1jwhC_	164
YGL020C	MDM39	1	unassigned	na	235
YGL021W	ALK1	1	unassigned	na	454
YGL021W	ALK1	2	psiblast	1cdkA_	95
YGL021W	ALK1	3	psiblast	1cdkA_	211
YGL022W	STT3	1	pfam	PF02516	660
YGL022W	STT3	2	unassigned	na	58
YGL023C	PIB2	1	psiblast	1i84S_	364
YGL023C	PIB2	2	unassigned	na	82
YGL023C	PIB2	3	psiblast	1vfyA_	129
YGL023C	PIB2	4	unassigned	na	60
YGL024W	YGL024W	1	unassigned	na	111
YGL025C	PGD1	1	fold_recogniti	1i3qA	431
YGL026C	TRP5	1	psiblast	1geqA_	280
YGL026C	TRP5	2	psiblast	1a5aB_	188
YGL026C	TRP5	3	psiblast	1a5aB_	239
YGL027C	CWH41	1	pfam	PF03200	833
YGL028C	SCW11	1	fold_recogniti	1m2vB	281
YGL028C	SCW11	2	psiblast	1ghsA_	85
YGL028C	SCW11	3	psiblast	1ghsA_	94
YGL028C	SCW11	4	psiblast	1ghsA_	82
YGL029W	CGR1	1	pfam	PF03879	120
YGL030W	RPL30	1	psiblast	1ck2A_	105
YGL031C	RPL24A	1	psiblast	1k5yS_	72
YGL031C	RPL24A	2	unassigned	na	83
YGL032C	AGA2	1	unassigned	na	87
YGL033W	HOP2	1	fold_recogniti	1ggqA	185
YGL034C	YGL034C	1	unassigned	na	121
YGL035C	MIG1	1	psiblast	1lu6A_	89
YGL035C	MIG1	2	psiblast	1lu6A_	104
YGL035C	MIG1	3	msa	na	61
YGL035C	MIG1	4	msa	na	179
YGL035C	MIG1	5	unassigned	na	71
YGL036W	YGL036W	1	fold_recogniti	1ej6B	600
YGL036W	YGL036W	2	unassigned	na	57
YGL036W	YGL036W	3	fold_recogniti	1i3qA	252
YGL037C	PNC1	1	psiblast	1ilwA_	216
YGL038C	OCH1	1	unassigned	na	58
YGL038C	OCH1	2	msa	na	159
YGL038C	OCH1	3	unassigned	na	263
YGL039W	YGL039W	1	psiblast	1keuA_	164
YGL039W	YGL039W	2	psiblast	1keuA_	120
YGL039W	YGL039W	3	psiblast	1keuA_	64
YGL040C	HEM2	1	psiblast	1ylvA_	342
YGL041C	YGL041C	1	unassigned	na	104

YGL042C	YGL042C	1	unassigned	na	101
YGL043W	DST1	1	psiblast	1eo0A_	103
YGL043W	DST1	2	psiblast	1enwA_	87
YGL043W	DST1	3	fold_recogniti	1enwA	68
YGL043W	DST1	4	psiblast	1tfi__	51
YGL044C	RNA15	1	psiblast	1fxlA_	96
YGL044C	RNA15	2	psiblast	1fxlA_	106
YGL044C	RNA15	3	psiblast	1g9lA_	94
YGL045W	RIM8	1	msa	na	229
YGL046W	2122	1	psiblast	1jsyA_	194
YGL046W	2122	2	psiblast	1jsyA_	68
YGL047W	YGL047W	1	psiblast	1f0kA_	202
YGL048C	RPT6	1	psiblast	1e32A_	144
YGL048C	RPT6	2	psiblast	1e32A_	174
YGL048C	RPT6	3	psiblast	1e32A_	87
YGL049C	TIF4632	1	unassigned	na	110
YGL049C	TIF4632	2	fold_recogniti	1l8mA	56
YGL049C	TIF4632	3	fold_recogniti	1jv2B	248
YGL049C	TIF4632	4	fold_recogniti	1jv2B	58
YGL049C	TIF4632	5	fold_recogniti	1i3qA	83
YGL049C	TIF4632	6	psiblast	1hu3A_	136
YGL049C	TIF4632	7	psiblast	1hu3A_	160
YGL049C	TIF4632	8	unassigned	na	63
YGL050W	YGL050W	1	pfam	PF02676	273
YGL051W	MST27	1	pfam	PF00674	234
YGL052W	YGL052W	1	unassigned	na	101
YGL053W	PRM8	1	pfam	PF00674	237
YGL054C	ERV14	1	pfam	PF03311	138
YGL055W	OLE1	1	msa	na	96
YGL055W	OLE1	2	pfam	PF00487	302
YGL055W	OLE1	3	psiblast	1iccA_	112
YGL056C	SDS23	1	psiblast	1zfjA_	324
YGL056C	SDS23	2	psiblast	1zfjA_	142
YGL056C	SDS23	3	unassigned	na	61
YGL057C	YGL057C	1	unassigned	na	287
YGL058W	RAD6	1	psiblast	1ayzA_	172
YGL059W	YGL059W	1	psiblast	1gkxA_	445
YGL060W	YBP2	1	fold_recogniti	1bgxT	57
YGL060W	YBP2	2	fold_recogniti	1bgxT	51
YGL060W	YBP2	3	fold_recogniti	1bgxT	480
YGL060W	YBP2	4	fold_recogniti	1bgxT	53
YGL061C	DUO1	1	unassigned	na	247
YGL062W	PYC1	1	psiblast	1lsrA_	128
YGL062W	PYC1	2	psiblast	1lsrA_	80
YGL062W	PYC1	3	psiblast	1lsrA_	136
YGL062W	PYC1	4	psiblast	1lsrA_	164
YGL062W	PYC1	5	fold_recogniti	1lt7A	385
YGL062W	PYC1	6	fold_recogniti	1dhpA	121

YGL062W	PYC1	7	unassigned	na	71
YGL062W	PYC1	8	psiblast	1dczA_	93
YGL063W	PUS2	1	psiblast	1dj0A_	124
YGL063W	PUS2	2	psiblast	1dj0A_	91
YGL063W	PUS2	3	pfam	PF01416	155
YGL064C	MRH4	1	unassigned	na	105
YGL064C	MRH4	2	psiblast	1hv8A_	237
YGL064C	MRH4	3	psiblast	1hv8A_	165
YGL064C	MRH4	4	fold_recogniti	1fuuA	54
YGL065C	ALG2	1	fold_recogniti	1iirA	212
YGL065C	ALG2	2	fold_recogniti	1iirA	76
YGL065C	ALG2	3	fold_recogniti	1iirA	215
YGL066W	SGF73	1	fold_recogniti	1l8mA	110
YGL066W	SGF73	2	fold_recogniti	1l8mA	79
YGL066W	SGF73	3	fold_recogniti	1l8mA	69
YGL066W	SGF73	4	fold_recogniti	1l8mA	74
YGL066W	SGF73	5	unassigned	na	325
YGL067W	NPY1	1	msa	na	190
YGL067W	NPY1	2	psiblast	1g0sA_	194
YGL068W	YGL068W	1	unassigned	na	110
YGL068W	YGL068W	2	psiblast	1ctf__	84
YGL069C	SRF3	1	unassigned	na	154
YGL070C	RPB9	1	psiblast	1k83I_	45
YGL070C	RPB9	2	psiblast	1k83I_	77
YGL071W	RCS1	1	unassigned	na	374
YGL071W	RCS1	2	fold_recogniti	1qhbA	316
YGL072C	YGL072C	1	unassigned	na	119
YGL073W	HSF1	1	unassigned	na	145
YGL073W	HSF1	2	psiblast	3htsB_	115
YGL073W	HSF1	3	fold_recogniti	3htsB	59
YGL073W	HSF1	4	fold_recogniti	1i3qA	514
YGL074C	YGL074C	1	unassigned	na	108
YGL075C	MPS2	1	unassigned	na	387
YGL076C	RPL7A	1	unassigned	na	77
YGL076C	RPL7A	2	psiblast	1k5yF_	167
YGL077C	HNM1	1	pfam	PF00324	563
YGL078C	DBP3	1	unassigned	na	78
YGL078C	DBP3	2	psiblast	1hv8A_	246
YGL078C	DBP3	3	psiblast	1hv8A_	199
YGL079W	YGL079W	1	unassigned	na	218
YGL080W	FMP37	1	pfam	PF03650	130
YGL081W	YGL081W	1	pfam	PF00498	152
YGL081W	YGL081W	2	unassigned	na	168
YGL082W	YGL082W	1	msa	na	287
YGL082W	YGL082W	2	unassigned	na	94
YGL083W	SCY1	1	psiblast	1fotA_	145
YGL083W	SCY1	2	psiblast	1fotA_	239
YGL083W	SCY1	3	psiblast	1b3uA_	189

YGL083W	SCY1	4	unassigned	na	231
YGL084C	GUP1	1	msa	na	123
YGL084C	GUP1	2	msa	na	437
YGL085W	YGL085W	1	unassigned	na	84
YGL085W	YGL085W	2	psiblast	1ey7A_	190
YGL086W	MAD1	1	psiblast	1i84S_	275
YGL086W	MAD1	2	psiblast	2tmaA_	89
YGL086W	MAD1	3	psiblast	2tmaA_	82
YGL086W	MAD1	4	psiblast	2tmaA_	62
YGL086W	MAD1	5	psiblast	2tmaA_	111
YGL086W	MAD1	6	msa	na	130
YGL087C	MMS2	1	psiblast	1jatB_	137
YGL088W	YGL088W	1	unassigned	na	121
YGL089C	MF(ALPHA)2	1	unassigned	na	120
YGL090W	LIF1	1	unassigned	na	421
YGL091C	NBP35	1	psiblast	1g3qA_	328
YGL092W	NUP145	1	psiblast	1deqA_	156
YGL092W	NUP145	2	msa	na	234
YGL092W	NUP145	3	unassigned	na	659
YGL092W	NUP145	4	msa	na	185
YGL092W	NUP145	5	unassigned	na	83
YGL093W	SPC105	1	fold_recogniti	1i3qA	371
YGL093W	SPC105	2	fold_recogniti	1ldvA	112
YGL093W	SPC105	3	psiblast	1i84S_	281
YGL093W	SPC105	4	unassigned	na	153
YGL094C	PAN2	1	psiblast	1gg2B_	395
YGL094C	PAN2	2	unassigned	na	73
YGL094C	PAN2	3	msa	na	427
YGL094C	PAN2	4	fold_recogniti	1fxxA	220
YGL095C	VPS45	1	psiblast	1fvfA_	136
YGL095C	VPS45	2	psiblast	1fvfA_	202
YGL095C	VPS45	3	psiblast	1fvfA_	112
YGL095C	VPS45	4	psiblast	1fvfA_	127
YGL096W	TOS8	1	msa	na	158
YGL096W	TOS8	2	psiblast	1b72B_	118
YGL097W	SRM1	1	psiblast	1a12A_	482
YGL098W	USE1	1	fold_recogniti	1jchA	245
YGL099W	LSG1	1	msa	na	102
YGL099W	LSG1	2	psiblast	1g7sA_	219
YGL099W	LSG1	3	psiblast	1egaA_	191
YGL099W	LSG1	4	psiblast	1egaA_	128
YGL100W	SEH1	1	psiblast	1erjA_	349
YGL101W	YGL101W	1	pfam	PF01966	215
YGL102C	YGL102C	1	unassigned	na	142
YGL103W	RPL28	1	psiblast	1k5yV_	57
YGL103W	RPL28	2	psiblast	1k5yV_	92
YGL104C	VPS73	1	psiblast	1ja5A_	440
YGL104C	VPS73	2	psiblast	1ja5A_	46

YGL105W	ARC1	1	psiblast	1jlvA_	139
YGL105W	ARC1	2	unassigned	na	59
YGL105W	ARC1	3	psiblast	1eujA_	178
YGL106W	MLC1	1	psiblast	1ggwA_	85
YGL106W	MLC1	2	psiblast	1ggwA_	64
YGL107C	RMD9	1	unassigned	na	124
YGL107C	RMD9	2	fold_recogniti	1qoyA	271
YGL107C	RMD9	3	unassigned	na	251
YGL108C	YGL108C	1	unassigned	na	140
YGL109W	YGL109W	1	unassigned	na	107
YGL110C	CUE3	1	unassigned	na	314
YGL110C	CUE3	2	pfam	PF02845	79
YGL110C	CUE3	3	unassigned	na	231
YGL111W	NSA1	1	unassigned	na	101
YGL111W	NSA1	2	fold_recogniti	1scgB	362
YGL112C	TAF6	1	psiblast	1tafB_	84
YGL112C	TAF6	2	unassigned	na	110
YGL112C	TAF6	3	fold_recogniti	1wq1G	154
YGL112C	TAF6	4	fold_recogniti	1wq1G	168
YGL113W	SLD3	1	unassigned	na	490
YGL113W	SLD3	2	msa	na	178
YGL114W	YGL114W	1	msa	na	519
YGL114W	YGL114W	2	msa	na	206
YGL115W	SNF4	1	psiblast	1jr1A_	322
YGL116W	CDC20	1	psiblast	1c5kA_	177
YGL116W	CDC20	2	psiblast	1c5kA_	260
YGL116W	CDC20	3	psiblast	1erjA_	173
YGL117W	YGL117W	1	unassigned	na	265
YGL118C	YGL118C	1	unassigned	na	145
YGL119W	ABC1	1	unassigned	na	104
YGL119W	ABC1	2	psiblast	1qcfA_	76
YGL119W	ABC1	3	psiblast	1a06__	128
YGL119W	ABC1	4	psiblast	1a06__	193
YGL120C	PRP43	1	unassigned	na	70
YGL120C	PRP43	2	psiblast	1hv8A_	203
YGL120C	PRP43	3	psiblast	1hv8A_	180
YGL120C	PRP43	4	fold_recogniti	1jr3D	70
YGL120C	PRP43	5	fold_recogniti	1jr3D	143
YGL120C	PRP43	6	msa	na	101
YGL121C	GPG1	1	unassigned	na	126
YGL122C	NAB2	1	fold_recogniti	1i3qA	237
YGL122C	NAB2	2	fold_recogniti	1jv2B	288
YGL123W	RPS2	1	unassigned	na	61
YGL123W	RPS2	2	psiblast	1k5xE_	193
YGL124C	MON1	1	unassigned	na	113
YGL124C	MON1	2	fold_recogniti	1gw5S	207
YGL124C	MON1	3	unassigned	na	92
YGL124C	MON1	4	msa	na	232

YGL125W	MET13	1	psiblast	1b5tA_	325
YGL125W	MET13	2	msa	na	274
YGL126W	SCS3	1	msa	na	127
YGL126W	SCS3	2	fold_recogniti	16vpA	139
YGL126W	SCS3	3	fold_recogniti	16vpA	114
YGL127C	SOH1	1	unassigned	na	127
YGL128C	CWC23	1	unassigned	na	65
YGL128C	CWC23	2	psiblast	1xbl__	118
YGL128C	CWC23	3	unassigned	na	164
YGL129C	RSM23	1	unassigned	na	112
YGL129C	RSM23	2	msa	na	286
YGL129C	RSM23	3	unassigned	na	90
YGL130W	CEG1	1	psiblast	1ckmA_	174
YGL130W	CEG1	2	psiblast	1ckmA_	79
YGL130W	CEG1	3	psiblast	1ckmA_	85
YGL130W	CEG1	4	fold_recogniti	1ckmA	121
YGL131C	SNT2	1	unassigned	na	102
YGL131C	SNT2	2	pfam	PF01426	170
YGL131C	SNT2	3	psiblast	1fp0A_	140
YGL131C	SNT2	4	msa	na	68
YGL131C	SNT2	5	unassigned	na	76
YGL131C	SNT2	6	pfam	PF00249	72
YGL131C	SNT2	7	unassigned	na	128
YGL131C	SNT2	8	msa	na	280
YGL131C	SNT2	9	pfam	PF00628	83
YGL131C	SNT2	10	msa	na	190
YGL131C	SNT2	11	pfam	PF00320	94
YGL132W	YGL132W	1	unassigned	na	111
YGL133W	ITC1	1	unassigned	na	119
YGL133W	ITC1	2	msa	na	294
YGL133W	ITC1	3	pfam	PF02791	93
YGL133W	ITC1	4	unassigned	na	758
YGL134W	PCL10	1	unassigned	na	206
YGL134W	PCL10	2	msa	na	227
YGL135W	RPL1B	1	psiblast	1k5yA_	127
YGL135W	RPL1B	2	psiblast	1k5yA_	90
YGL136C	MRM2	1	psiblast	1eizA_	226
YGL136C	MRM2	2	fold_recogniti	1eizA	94
YGL137W	SEC27	1	psiblast	1erjA_	311
YGL137W	SEC27	2	msa	na	66
YGL137W	SEC27	3	psiblast	1c5kA_	252
YGL137W	SEC27	4	fold_recogniti	1b89A	183
YGL137W	SEC27	5	msa	na	77
YGL138C	YGL138C	1	unassigned	na	345
YGL139W	YGL139W	1	msa	na	192
YGL139W	YGL139W	2	unassigned	na	109
YGL139W	YGL139W	3	msa	na	278
YGL139W	YGL139W	4	msa	na	170

YGL139W	YGL139W	5	unassigned	na	53
YGL140C	YGL140C	1	unassigned	na	576
YGL140C	YGL140C	2	msa	na	355
YGL140C	YGL140C	3	unassigned	na	288
YGL141W	HUL5	1	unassigned	na	323
YGL141W	HUL5	2	msa	na	135
YGL141W	HUL5	3	unassigned	na	91
YGL141W	HUL5	4	psiblast	1c4zA_	47
YGL141W	HUL5	5	psiblast	1c4zA_	142
YGL141W	HUL5	6	psiblast	1c4zA_	172
YGL142C	GPI10	1	pfam	PF03901	616
YGL143C	MRF1	1	psiblast	1gqeA_	143
YGL143C	MRF1	2	psiblast	1gqeA_	182
YGL143C	MRF1	3	psiblast	1gqeA_	88
YGL144C	ROG1	1	unassigned	na	176
YGL144C	ROG1	2	psiblast	1hqdA_	218
YGL144C	ROG1	3	unassigned	na	291
YGL145W	TIP20	1	fold_recogniti	1h6kA	221
YGL145W	TIP20	2	fold_recogniti	1h6kA	118
YGL145W	TIP20	3	fold_recogniti	1h6kA	93
YGL145W	TIP20	4	fold_recogniti	1h6kA	198
YGL145W	TIP20	5	unassigned	na	71
YGL146C	YGL146C	1	unassigned	na	311
YGL147C	RPL9A	1	psiblast	1k5yH_	84
YGL147C	RPL9A	2	psiblast	1k5yH_	107
YGL148W	ARO2	1	fold_recogniti	1fw8A	376
YGL149W	YGL149W	1	unassigned	na	101
YGL150C	INO80	1	psiblast	1i84S_	420
YGL150C	INO80	2	unassigned	na	217
YGL150C	INO80	3	fold_recogniti	1gm5A	319
YGL150C	INO80	4	fold_recogniti	1gm5A	226
YGL150C	INO80	5	psiblast	1hv8A_	232
YGL150C	INO80	6	unassigned	na	75
YGL151W	NUT1	1	unassigned	na	1132
YGL152C	YGL152C	1	unassigned	na	225
YGL153W	PEX14	1	msa	na	124
YGL153W	PEX14	2	psiblast	1i84S_	129
YGL153W	PEX14	3	unassigned	na	88
YGL154C	LYS5	1	psiblast	1qr0A_	113
YGL154C	LYS5	2	psiblast	1qr0A_	159
YGL155W	CDC43	1	psiblast	1dceB_	198
YGL155W	CDC43	2	psiblast	1dceB_	178
YGL156W	AMS1	1	unassigned	na	191
YGL156W	AMS1	2	psiblast	1hwwA_	489
YGL156W	AMS1	3	fold_recogniti	1htyA	286
YGL156W	AMS1	4	msa	na	117
YGL157W	YGL157W	1	psiblast	1keuA_	163
YGL157W	YGL157W	2	psiblast	1keuA_	116

YGL157W	YGL157W	3	psiblast	1keuA_	68
YGL158W	RCK1	1	unassigned	na	94
YGL158W	RCK1	2	psiblast	1a06__	140
YGL158W	RCK1	3	psiblast	1a06__	223
YGL158W	RCK1	4	psiblast	1lj6A_	55
YGL159W	YGL159W	1	unassigned	na	60
YGL159W	YGL159W	2	fold_recogniti	1gpjA	223
YGL159W	YGL159W	3	unassigned	na	87
YGL160W	YGL160W	1	msa	na	175
YGL160W	YGL160W	2	unassigned	na	52
YGL160W	YGL160W	3	psiblast	1cnf__	132
YGL160W	YGL160W	4	psiblast	1cnf__	211
YGL161C	YIP5	1	unassigned	na	202
YGL161C	YIP5	2	msa	na	108
YGL162W	SUT1	1	unassigned	na	299
YGL163C	RAD54	1	msa	na	223
YGL163C	RAD54	2	fold_recogniti	1gm5A	52
YGL163C	RAD54	3	fold_recogniti	1gm5A	308
YGL163C	RAD54	4	psiblast	1hv8A_	57
YGL163C	RAD54	5	psiblast	1d9xA_	258
YGL164C	YRB30	1	unassigned	na	216
YGL164C	YRB30	2	msa	na	224
YGL165C	YGL165C	1	unassigned	na	192
YGL166W	CUP2	1	psiblast	1co4A_	75
YGL166W	CUP2	2	unassigned	na	150
YGL167C	PMR1	1	psiblast	1eulA_	201
YGL167C	PMR1	2	psiblast	1eulA_	347
YGL167C	PMR1	3	psiblast	1eulA_	199
YGL167C	PMR1	4	psiblast	1eulA_	203
YGL168W	HUR1	1	unassigned	na	110
YGL169W	SUA5	1	psiblast	1jcuA_	285
YGL169W	SUA5	2	msa	na	141
YGL170C	SPO74	1	unassigned	na	95
YGL170C	SPO74	2	fold_recogniti	1iu4A	318
YGL171W	ROK1	1	unassigned	na	95
YGL171W	ROK1	2	psiblast	1hv8A_	245
YGL171W	ROK1	3	psiblast	1hv8A_	157
YGL171W	ROK1	4	unassigned	na	67
YGL172W	NUP49	1	psiblast	1deqA_	238
YGL172W	NUP49	2	fold_recogniti	1dn1B	177
YGL172W	NUP49	3	fold_recogniti	1dn1B	57
YGL173C	KEM1	1	pfam	PF03159	231
YGL173C	KEM1	2	unassigned	na	72
YGL173C	KEM1	3	msa	na	411
YGL173C	KEM1	4	unassigned	na	814
YGL174W	BUD13	1	unassigned	na	74
YGL174W	BUD13	2	msa	na	113
YGL174W	BUD13	3	unassigned	na	79

YGL175C	SAE2	1	unassigned	na	345
YGL176C	YGL176C	1	unassigned	na	554
YGL177W	YGL177W	1	unassigned	na	115
YGL178W	MPT5	1	msa	na	181
YGL178W	MPT5	2	psiblast	1ib2A_	147
YGL178W	MPT5	3	psiblast	1ib2A_	101
YGL178W	MPT5	4	psiblast	1ib2A_	199
YGL178W	MPT5	5	unassigned	na	231
YGL179C	TOS3	1	psiblast	1ctpE_	318
YGL179C	TOS3	2	psiblast	1ctpE_	121
YGL179C	TOS3	3	unassigned	na	121
YGL180W	ATG1	1	psiblast	1fotA_	91
YGL180W	ATG1	2	psiblast	1fotA_	336
YGL180W	ATG1	3	msa	na	470
YGL181W	GTS1	1	psiblast	1dcqA_	173
YGL181W	GTS1	2	pfam	PF00627	76
YGL181W	GTS1	3	unassigned	na	147
YGL182C	YGL182C	1	unassigned	na	107
YGL183C	MND1	1	unassigned	na	174
YGL184C	STR3	1	psiblast	1ibjA_	279
YGL184C	STR3	2	psiblast	1ibjA_	186
YGL185C	YGL185C	1	psiblast	1gdhA_	169
YGL185C	YGL185C	2	psiblast	1gdhA_	210
YGL186C	TPN1	1	unassigned	na	68
YGL186C	TPN1	2	pfam	PF02133	511
YGL187C	COX4	1	psiblast	1occF_	155
YGL188C	YGL188C	1	unassigned	na	57
YGL189C	RPS26A	1	pfam	PF01283	119
YGL190C	CDC55	1	psiblast	1b9xA_	419
YGL190C	CDC55	2	psiblast	1gxrA_	107
YGL191W	COX13	1	psiblast	1occG_	129
YGL192W	IME4	1	unassigned	na	295
YGL192W	IME4	2	msa	na	237
YGL192W	IME4	3	unassigned	na	68
YGL193C	YGL193C	1	unassigned	na	103
YGL194C	HOS2	1	psiblast	1c3pA_	185
YGL194C	HOS2	2	psiblast	1c3pA_	185
YGL194C	HOS2	3	unassigned	na	82
YGL195W	GCN1	1	unassigned	na	191
YGL195W	GCN1	2	msa	na	291
YGL195W	GCN1	3	unassigned	na	485
YGL195W	GCN1	4	msa	na	728
YGL195W	GCN1	5	unassigned	na	114
YGL195W	GCN1	6	msa	na	342
YGL195W	GCN1	7	msa	na	437
YGL195W	GCN1	8	unassigned	na	84
YGL196W	YGL196W	1	unassigned	na	165
YGL197W	MDS3	1	unassigned	na	88

YGL197W	MDS3	2	psiblast	1gof__	246
YGL197W	MDS3	3	unassigned	na	219
YGL197W	MDS3	4	msa	na	240
YGL197W	MDS3	5	unassigned	na	694
YGL198W	YIP4	1	msa	na	140
YGL198W	YIP4	2	msa	na	121
YGL199C	YGL199C	1	unassigned	na	156
YGL200C	EMP24	1	pfam	PF01105	203
YGL201C	MCM6	1	unassigned	na	94
YGL201C	MCM6	2	msa	na	175
YGL201C	MCM6	3	pfam	PF00493	177
YGL201C	MCM6	4	psiblast	1dbwA_	81
YGL201C	MCM6	5	psiblast	1g8pA_	232
YGL201C	MCM6	6	psiblast	1g8pA_	108
YGL201C	MCM6	7	unassigned	na	150
YGL202W	ARO8	1	psiblast	1bkgA_	348
YGL202W	ARO8	2	psiblast	1bkgA_	152
YGL203C	KEX1	1	psiblast	1ac5__	286
YGL203C	KEX1	2	psiblast	1ac5__	125
YGL203C	KEX1	3	psiblast	1ac5__	94
YGL203C	KEX1	4	fold_recogniti	1l8mA	85
YGL203C	KEX1	5	fold_recogniti	1l8mA	70
YGL203C	KEX1	6	fold_recogniti	1l8mA	69
YGL204C	YGL204C	1	unassigned	na	101
YGL205W	POX1	1	psiblast	1is2A_	465
YGL205W	POX1	2	psiblast	1is2A_	283
YGL206C	CHC1	1	psiblast	1bpoA_	176
YGL206C	CHC1	2	psiblast	1bpoA_	200
YGL206C	CHC1	3	psiblast	1bpoA_	122
YGL206C	CHC1	4	fold_recogniti	1m9iA	63
YGL206C	CHC1	5	fold_recogniti	1m9iA	245
YGL206C	CHC1	6	fold_recogniti	1m9iA	200
YGL206C	CHC1	7	fold_recogniti	1m9iA	71
YGL206C	CHC1	8	psiblast	1b89A_	473
YGL206C	CHC1	9	msa	na	103
YGL207W	SPT16	1	psiblast	1az9__	171
YGL207W	SPT16	2	psiblast	1az9__	297
YGL207W	SPT16	3	unassigned	na	55
YGL207W	SPT16	4	msa	na	250
YGL207W	SPT16	5	unassigned	na	145
YGL207W	SPT16	6	msa	na	117
YGL208W	SIP2	1	unassigned	na	97
YGL208W	SIP2	2	msa	na	318
YGL209W	MIG2	1	psiblast	2gliA_	66
YGL209W	MIG2	2	psiblast	2gliA_	51
YGL209W	MIG2	3	unassigned	na	85
YGL209W	MIG2	4	psiblast	1lu6A_	53
YGL209W	MIG2	5	psiblast	1lu6A_	127

YGL210W	YPT32	1	psiblast	1g17A_	222
YGL211W	NCS6	1	psiblast	1gpmA_	193
YGL212W	VAM7	1	psiblast	1kmdA_	121
YGL212W	VAM7	2	psiblast	1sfcC_	102
YGL212W	VAM7	3	psiblast	1l4aD_	93
YGL213C	SKI8	1	psiblast	1gg2B_	397
YGL214W	YGL214W	1	unassigned	na	162
YGL215W	CLG1	1	unassigned	na	89
YGL215W	CLG1	2	msa	na	224
YGL215W	CLG1	3	unassigned	na	139
YGL216W	KIP3	1	unassigned	na	101
YGL216W	KIP3	2	psiblast	1gojA_	365
YGL216W	KIP3	3	psiblast	1i84S_	339
YGL217C	YGL217C	1	unassigned	na	113
YGL218W	SRF1	1	unassigned	na	216
YGL219C	MDM34	1	msa	na	277
YGL219C	MDM34	2	unassigned	na	182
YGL220W	YGL220W	1	pfam	PF01722	120
YGL221C	NIF3	1	pfam	PF01784	288
YGL222C	EDC1	1	unassigned	na	175
YGL223C	COG1	1	unassigned	na	417
YGL224C	SDT1	1	psiblast	1jud__	217
YGL224C	SDT1	2	psiblast	1jud__	63
YGL225W	VRG4	1	unassigned	na	80
YGL225W	VRG4	2	msa	na	257
YGL226C-A	OST5	1	unassigned	na	86
YGL226W	YGL226W	1	fold_recogniti	1occD	123
YGL227W	VID30	1	fold_recogniti	1l8mA	127
YGL227W	VID30	2	fold_recogniti	1l8mA	73
YGL227W	VID30	3	fold_recogniti	1l8mA	79
YGL227W	VID30	4	fold_recogniti	1l8mA	70
YGL227W	VID30	5	unassigned	na	71
YGL227W	VID30	6	msa	na	252
YGL227W	VID30	7	unassigned	na	286
YGL228W	SHE10	1	unassigned	na	577
YGL229C	SAP4	1	msa	na	528
YGL229C	SAP4	2	msa	na	290
YGL230C	YGL230C	1	unassigned	na	147
YGL231C	YGL231C	1	unassigned	na	190
YGL232W	TAN1	1	unassigned	na	101
YGL232W	TAN1	2	pfam	PF02926	188
YGL233W	SEC15	1	fold_recogniti	1ddbA	76
YGL233W	SEC15	2	fold_recogniti	1av1A	215
YGL233W	SEC15	3	unassigned	na	88
YGL233W	SEC15	4	fold_recogniti	1b8fA	225
YGL233W	SEC15	5	fold_recogniti	1b8fA	171
YGL233W	SEC15	6	unassigned	na	135
YGL234W	ADE5,7	1	psiblast	1gsoA_	98

YGL234W	ADE5,7	2	psiblast	1gsoA_	154
YGL234W	ADE5,7	3	psiblast	1gsoA_	193
YGL234W	ADE5,7	4	fold_recogniti	1cliA	66
YGL234W	ADE5,7	5	psiblast	1cliA_	104
YGL234W	ADE5,7	6	psiblast	1cliA_	141
YGL234W	ADE5,7	7	psiblast	1cliA_	46
YGL235W	YGL235W	1	unassigned	na	178
YGL236C	MTO1	1	unassigned	na	72
YGL236C	MTO1	2	psiblast	1ebdA_	137
YGL236C	MTO1	3	psiblast	1ebdA_	61
YGL236C	MTO1	4	unassigned	na	61
YGL236C	MTO1	5	msa	na	106
YGL236C	MTO1	6	msa	na	242
YGL237C	HAP2	1	msa	na	152
YGL237C	HAP2	2	pfam	PF02045	113
YGL238W	CSE1	1	msa	na	220
YGL238W	CSE1	2	msa	na	591
YGL238W	CSE1	3	msa	na	149
YGL239C	YGL239C	1	unassigned	na	104
YGL240W	DOC1	1	psiblast	1gqpA_	283
YGL241W	KAP114	1	msa	na	704
YGL241W	KAP114	2	msa	na	211
YGL241W	KAP114	3	unassigned	na	89
YGL242C	YGL242C	1	psiblast	1awcB_	88
YGL242C	YGL242C	2	psiblast	1awcB_	93
YGL243W	TAD1	1	unassigned	na	69
YGL243W	TAD1	2	pfam	PF02137	331
YGL244W	RTF1	1	msa	na	230
YGL244W	RTF1	2	pfam	PF03126	120
YGL244W	RTF1	3	unassigned	na	208
YGL245W	GSN1	1	msa	na	128
YGL245W	GSN1	2	psiblast	1iq0A_	65
YGL245W	GSN1	3	psiblast	1euqA_	253
YGL245W	GSN1	4	psiblast	1euqA_	97
YGL245W	GSN1	5	psiblast	1euqA_	181
YGL246C	RAI1	1	msa	na	303
YGL246C	RAI1	2	unassigned	na	84
YGL247W	BRR6	1	unassigned	na	197
YGL248W	PDE1	1	pfam	PF02112	369
YGL249W	ZIP2	1	unassigned	na	704
YGL250W	YGL250W	1	unassigned	na	245
YGL251C	HFM1	1	psiblast	1hv8A_	190
YGL251C	HFM1	2	psiblast	1hv8A_	191
YGL251C	HFM1	3	psiblast	1d9xA_	88
YGL251C	HFM1	4	pfam	PF02889	360
YGL251C	HFM1	5	unassigned	na	217
YGL252C	RTG2	1	fold_recogniti	1jceA	134
YGL252C	RTG2	2	fold_recogniti	1jceA	217

YGL252C	RTG2	3	msa	na	173
YGL252C	RTG2	4	unassigned	na	64
YGL253W	HXK2	1	psiblast	1ig8A_	107
YGL253W	HXK2	2	psiblast	1ig8A_	379
YGL254W	FZF1	1	psiblast	1lu6A_	65
YGL254W	FZF1	2	psiblast	1lu6A_	72
YGL254W	FZF1	3	msa	na	162
YGL255W	ZRT1	1	msa	na	193
YGL255W	ZRT1	2	pfam	PF02535	183
YGL256W	ADH4	1	unassigned	na	74
YGL256W	ADH4	2	psiblast	1j5rA_	191
YGL256W	ADH4	3	psiblast	1j5rA_	78
YGL256W	ADH4	4	psiblast	1j5rA_	122
YGL257C	MNT2	1	unassigned	na	129
YGL257C	MNT2	2	fold_recogniti	1ll0A	176
YGL257C	MNT2	3	unassigned	na	253
YGL258W	VEL1	1	unassigned	na	206
YGL258W-A	YGL258W-A	1	psiblast	1eagA_	77
YGL259W	YPS5	1	psiblast	1ypsA_	165
YGL260W	YGL260W	1	unassigned	na	76
YGL262W	YGL262W	1	unassigned	na	175
YGL263W	COS12	1	msa	na	59
YGL263W	COS12	2	pfam	PF00674	112
YGL263W	COS12	3	unassigned	na	75
YGL263W	COS12	4	pfam	PF00674	134
YGR001C	YGR001C	1	unassigned	na	193
YGR002C	SWC4	1	unassigned	na	160
YGR002C	SWC4	2	pfam	PF00249	61
YGR002C	SWC4	3	unassigned	na	255
YGR003W	CUL3	1	psiblast	1ldjA_	102
YGR003W	CUL3	2	psiblast	1ldjA_	183
YGR003W	CUL3	3	psiblast	1ldjA_	192
YGR003W	CUL3	4	psiblast	1ldjA_	74
YGR003W	CUL3	5	psiblast	1ldjA_	119
YGR003W	CUL3	6	psiblast	1ldjA_	74
YGR004W	PEX31	1	unassigned	na	462
YGR005C	TFG2	1	psiblast	1f3uA_	194
YGR005C	TFG2	2	unassigned	na	75
YGR005C	TFG2	3	psiblast	1bby__	131
YGR006W	PRP18	1	unassigned	na	78
YGR006W	PRP18	2	psiblast	1dvkA_	141
YGR007W	MUQ1	1	psiblast	1cozA_	173
YGR007W	MUQ1	2	psiblast	1b6tA_	150
YGR008C	STF2	1	unassigned	na	84
YGR009C	SEC9	1	unassigned	na	204
YGR009C	SEC9	2	psiblast	1bg1A_	98
YGR009C	SEC9	3	psiblast	1i84S_	349
YGR010W	NMA2	1	unassigned	na	112

YGR010W	NMA2	2	psiblast	1kkuA_	283
YGR011W	YGR011W	1	unassigned	na	108
YGR012W	YGR012W	1	psiblast	1jbqA_	294
YGR012W	YGR012W	2	psiblast	1jbqA_	99
YGR013W	SNU71	1	psiblast	1i84S_	383
YGR013W	SNU71	2	msa	na	237
YGR014W	MSB2	1	msa	na	568
YGR014W	MSB2	2	msa	na	298
YGR014W	MSB2	3	unassigned	na	157
YGR014W	MSB2	4	msa	na	200
YGR014W	MSB2	5	unassigned	na	83
YGR015C	YGR015C	1	psiblast	1cqzA_	328
YGR016W	YGR016W	1	unassigned	na	190
YGR017W	YGR017W	1	fold_recogniti	1ci0A	152
YGR017W	YGR017W	2	unassigned	na	145
YGR018C	YGR018C	1	unassigned	na	109
YGR019W	UGA1	1	psiblast	1gtxA_	471
YGR020C	VMA7	1	pfam	PF01990	118
YGR021W	YGR021W	1	psiblast	1lfpA_	218
YGR021W	YGR021W	2	psiblast	1lfpA_	72
YGR022C	YGR022C	1	unassigned	na	109
YGR023W	MTL1	1	unassigned	na	70
YGR023W	MTL1	2	fold_recogniti	1deqA	64
YGR023W	MTL1	3	fold_recogniti	1m2vB	254
YGR023W	MTL1	4	msa	na	163
YGR024C	THG1	1	unassigned	na	85
YGR024C	THG1	2	msa	na	152
YGR025W	YGR025W	1	unassigned	na	100
YGR026W	YGR026W	1	unassigned	na	278
YGR027C	RPS25A	1	pfam	PF03297	108
YGR028W	MSP1	1	psiblast	1e32A_	87
YGR028W	MSP1	2	psiblast	1e32A_	170
YGR028W	MSP1	3	psiblast	1e32A_	105
YGR029W	ERV1	1	msa	na	72
YGR029W	ERV1	2	psiblast	1jr8A_	117
YGR030C	POP6	1	unassigned	na	158
YGR031W	YGR031W	1	psiblast	1cqzA_	342
YGR032W	GSC2	1	msa	na	233
YGR032W	GSC2	2	msa	na	307
YGR032W	GSC2	3	unassigned	na	281
YGR032W	GSC2	4	pfam	PF02364	864
YGR032W	GSC2	5	msa	na	210
YGR033C	FMP17	1	msa	na	239
YGR034W	RPL26B	1	psiblast	1k5yU_	129
YGR035C	YGR035C	1	unassigned	na	116
YGR036C	CAX4	1	psiblast	1d2tA_	239
YGR037C	ACB1	1	psiblast	2abd__	87
YGR038W	ORM1	1	msa	na	222

YGR039W	YGR039W	1	unassigned	na	103
YGR040W	KSS1	1	psiblast	1erk__	108
YGR040W	KSS1	2	psiblast	1erk__	260
YGR041W	BUD9	1	unassigned	na	547
YGR042W	YGR042W	1	unassigned	na	271
YGR043C	YGR043C	1	psiblast	1f05A_	333
YGR044C	RME1	1	psiblast	1tf6A_	172
YGR044C	RME1	2	psiblast	1meyG_	79
YGR044C	RME1	3	psiblast	1sp2__	49
YGR045C	YGR045C	1	unassigned	na	120
YGR046W	YGR046W	1	unassigned	na	103
YGR046W	YGR046W	2	msa	na	282
YGR047C	TFC4	1	psiblast	1fchA_	67
YGR047C	TFC4	2	psiblast	1b89A_	336
YGR047C	TFC4	3	psiblast	1qqeA_	138
YGR047C	TFC4	4	msa	na	103
YGR047C	TFC4	5	unassigned	na	85
YGR047C	TFC4	6	fold_recogniti	1fchA	95
YGR047C	TFC4	7	fold_recogniti	1fchA	101
YGR047C	TFC4	8	fold_recogniti	1fchA	100
YGR048W	UFD1	1	fold_recogniti	1cz4A	118
YGR048W	UFD1	2	fold_recogniti	1cz4A	92
YGR048W	UFD1	3	unassigned	na	151
YGR049W	SCM4	1	unassigned	na	187
YGR050C	YGR050C	1	unassigned	na	118
YGR051C	YGR051C	1	unassigned	na	107
YGR052W	FMP48	1	psiblast	1fotA_	68
YGR052W	FMP48	2	psiblast	1fotA_	240
YGR052W	FMP48	3	psiblast	1ggzA_	61
YGR053C	YGR053C	1	unassigned	na	283
YGR054W	YGR054W	1	psiblast	1erjA_	362
YGR054W	YGR054W	2	psiblast	1gxrA_	135
YGR054W	YGR054W	3	msa	na	145
YGR055W	MUP1	1	unassigned	na	52
YGR055W	MUP1	2	pfam	PF00324	522
YGR056W	RSC1	1	psiblast	1e6iA_	87
YGR056W	RSC1	2	psiblast	1eqfA_	143
YGR056W	RSC1	3	psiblast	1eqfA_	115
YGR056W	RSC1	4	fold_recogniti	1m4zA	193
YGR056W	RSC1	5	msa	na	159
YGR056W	RSC1	6	unassigned	na	231
YGR057C	LST7	1	unassigned	na	59
YGR057C	LST7	2	msa	na	186
YGR058W	YGR058W	1	psiblast	1juoA_	160
YGR058W	YGR058W	2	psiblast	1juoA_	85
YGR058W	YGR058W	3	psiblast	1hqvA_	90
YGR059W	SPR3	1	msa	na	81
YGR059W	SPR3	2	fold_recogniti	1h65A	103

YGR059W	SPR3	3	psiblast	1i84S_	328
YGR060W	ERG25	1	pfam	PF01598	309
YGR061C	ADE6	1	unassigned	na	251
YGR061C	ADE6	2	psiblast	1cliA_	212
YGR061C	ADE6	3	psiblast	1cliA_	150
YGR061C	ADE6	4	psiblast	1cliA_	42
YGR061C	ADE6	5	msa	na	407
YGR061C	ADE6	6	psiblast	1jvnA_	296
YGR062C	COX18	1	msa	na	119
YGR062C	COX18	2	pfam	PF02096	197
YGR063C	SPT4	1	unassigned	na	102
YGR064W	YGR064W	1	unassigned	na	122
YGR065C	VHT1	1	unassigned	na	64
YGR065C	VHT1	2	psiblast	1ja5A_	529
YGR066C	YGR066C	1	unassigned	na	63
YGR066C	YGR066C	2	msa	na	229
YGR067C	YGR067C	1	psiblast	1lu6A_	69
YGR067C	YGR067C	2	psiblast	1lu6A_	50
YGR067C	YGR067C	3	psiblast	1meyC_	46
YGR067C	YGR067C	4	psiblast	1ubdC_	172
YGR067C	YGR067C	5	unassigned	na	457
YGR068C	YGR068C	1	msa	na	126
YGR068C	YGR068C	2	unassigned	na	75
YGR068C	YGR068C	3	psiblast	1jsyA_	122
YGR068C	YGR068C	4	psiblast	1jsyA_	132
YGR068C	YGR068C	5	msa	na	131
YGR069W	YGR069W	1	unassigned	na	111
YGR070W	ROM1	1	fold_recogniti	1k83A	268
YGR070W	ROM1	2	pfam	PF00610	132
YGR070W	ROM1	3	unassigned	na	55
YGR070W	ROM1	4	psiblast	1ki1B_	199
YGR070W	ROM1	5	psiblast	1ki1B_	185
YGR070W	ROM1	6	pfam	PF00780	316
YGR071C	YGR071C	1	unassigned	na	72
YGR071C	YGR071C	2	pfam	PF02892	80
YGR071C	YGR071C	3	fold_recogniti	1h6kA	303
YGR071C	YGR071C	4	fold_recogniti	1h6kA	201
YGR071C	YGR071C	5	fold_recogniti	1e8yA	80
YGR071C	YGR071C	6	unassigned	na	124
YGR072W	UPF3	1	unassigned	na	60
YGR072W	UPF3	2	pfam	PF03467	220
YGR072W	UPF3	3	unassigned	na	107
YGR073C	YGR073C	1	unassigned	na	123
YGR074W	SMD1	1	psiblast	1b34A_	146
YGR075C	PRP38	1	pfam	PF03371	242
YGR076C	MRPL25	1	unassigned	na	157
YGR077C	PEX8	1	unassigned	na	298
YGR077C	PEX8	2	msa	na	291

YGR078C	PAC10	1	fold_recogniti	1fxkC	119
YGR078C	PAC10	2	fold_recogniti	1fxkC	80
YGR079W	YGR079W	1	unassigned	na	370
YGR080W	TWF1	1	psiblast	1f7sA_	166
YGR080W	TWF1	2	fold_recogniti	1cfyA	166
YGR081C	SLX9	1	unassigned	na	210
YGR082W	TOM20	1	pfam	PF02064	74
YGR082W	TOM20	2	psiblast	1om2A_	109
YGR083C	GCD2	1	psiblast	1i84S_	354
YGR083C	GCD2	2	fold_recogniti	1m0sA	184
YGR083C	GCD2	3	msa	na	113
YGR084C	MRP13	1	unassigned	na	339
YGR085C	RPL11B	1	psiblast	1k5yJ_	174
YGR086C	PIL1	1	fold_recogniti	1egyA	278
YGR086C	PIL1	2	unassigned	na	61
YGR087C	PDC6	1	psiblast	1qpbA_	188
YGR087C	PDC6	2	psiblast	1qpbA_	165
YGR087C	PDC6	3	psiblast	1qpbA_	210
YGR088W	CTT1	1	psiblast	1a4eA_	64
YGR088W	CTT1	2	psiblast	1a4eA_	509
YGR089W	NNF2	1	unassigned	na	208
YGR089W	NNF2	2	msa	na	381
YGR089W	NNF2	3	unassigned	na	68
YGR089W	NNF2	4	psiblast	2tmaA_	54
YGR089W	NNF2	5	psiblast	2tmaA_	78
YGR089W	NNF2	6	psiblast	2tmaA_	63
YGR089W	NNF2	7	psiblast	2tmaA_	84
YGR090W	UTP22	1	unassigned	na	164
YGR090W	UTP22	2	pfam	PF03813	1073
YGR091W	PRP31	1	fold_recogniti	1bgxT	406
YGR091W	PRP31	2	unassigned	na	88
YGR092W	DBF2	1	unassigned	na	95
YGR092W	DBF2	2	psiblast	1ctpE_	347
YGR092W	DBF2	3	psiblast	1ctpE_	130
YGR093W	YGR093W	1	msa	na	217
YGR093W	YGR093W	2	msa	na	290
YGR094W	VAS1	1	msa	na	121
YGR094W	VAS1	2	psiblast	1gaxA_	324
YGR094W	VAS1	3	psiblast	1gaxA_	512
YGR094W	VAS1	4	psiblast	1gaxA_	147
YGR095C	RRP46	1	psiblast	1e3pA_	106
YGR095C	RRP46	2	psiblast	1e3pA_	150
YGR096W	TPC1	1	fold_recogniti	1is2A	314
YGR097W	ASK10	1	msa	na	174
YGR097W	ASK10	2	unassigned	na	285
YGR097W	ASK10	3	pfam	PF00169	299
YGR097W	ASK10	4	unassigned	na	388
YGR098C	ESP1	1	unassigned	na	759

YGR098C	ESP1	2	pfam	PF03568	871
YGR099W	TEL2	1	unassigned	na	158
YGR099W	TEL2	2	fold_recogniti	1h6kA	231
YGR099W	TEL2	3	fold_recogniti	1h6kA	163
YGR099W	TEL2	4	unassigned	na	136
YGR100W	MDR1	1	pfam	PF02893	164
YGR100W	MDR1	2	psiblast	1fkmA_	236
YGR100W	MDR1	3	psiblast	1fkmA_	53
YGR100W	MDR1	4	unassigned	na	69
YGR100W	MDR1	5	psiblast	1iku__	115
YGR100W	MDR1	6	psiblast	1iku__	129
YGR100W	MDR1	7	unassigned	na	184
YGR101W	PCP1	1	unassigned	na	104
YGR101W	PCP1	2	pfam	PF01694	242
YGR102C	YGR102C	1	unassigned	na	183
YGR103W	NOP7	1	msa	na	241
YGR103W	NOP7	2	unassigned	na	63
YGR103W	NOP7	3	pfam	PF00533	153
YGR103W	NOP7	4	psiblast	1dg3A_	148
YGR104C	SRB5	1	unassigned	na	307
YGR105W	VMA21	1	unassigned	na	77
YGR106C	YGR106C	1	unassigned	na	265
YGR107W	YGR107W	1	unassigned	na	149
YGR108W	CLB1	1	msa	na	203
YGR108W	CLB1	2	psiblast	1gy3B_	146
YGR108W	CLB1	3	psiblast	1gy3B_	122
YGR109C	CLB6	1	unassigned	na	91
YGR109C	CLB6	2	psiblast	1vin__	164
YGR109C	CLB6	3	psiblast	1vin__	125
YGR110W	YGR110W	1	psiblast	1cqzA_	445
YGR111W	YGR111W	1	unassigned	na	400
YGR112W	SHY1	1	pfam	PF02104	389
YGR113W	DAM1	1	unassigned	na	343
YGR114C	YGR114C	1	unassigned	na	129
YGR115C	YGR115C	1	msa	na	197
YGR115C	YGR115C	2	unassigned	na	62
YGR116W	SPT6	1	psiblast	1i84S_	336
YGR116W	SPT6	2	msa	na	322
YGR116W	SPT6	3	unassigned	na	212
YGR116W	SPT6	4	msa	na	131
YGR116W	SPT6	5	unassigned	na	113
YGR116W	SPT6	6	psiblast	1sro__	108
YGR116W	SPT6	7	fold_recogniti	1fu5A	152
YGR116W	SPT6	8	msa	na	77
YGR117C	YGR117C	1	unassigned	na	476
YGR118W	RPS23A	1	psiblast	1k5xL_	145
YGR119C	NUP57	1	fold_recogniti	1ldvA	287
YGR119C	NUP57	2	psiblast	1avc__	140

YGR119C	NUP57	3	unassigned	na	114
YGR120C	COG2	1	fold_recogniti	1bkdS	275
YGR121C	MEP1	1	pfam	PF00909	492
YGR122C-A	YGR122C-A	1	unassigned	na	42
YGR122W	YGR122W	1	unassigned	na	402
YGR123C	PPT1	1	psiblast	1a17__	44
YGR123C	PPT1	2	psiblast	1a17__	122
YGR123C	PPT1	3	psiblast	1fjmA_	347
YGR124W	ASN2	1	psiblast	1ct9A_	192
YGR124W	ASN2	2	psiblast	1ct9A_	380
YGR125W	YGR125W	1	unassigned	na	76
YGR125W	YGR125W	2	psiblast	1hynP_	109
YGR125W	YGR125W	3	msa	na	478
YGR125W	YGR125W	4	fold_recogniti	1h4xA	161
YGR125W	YGR125W	5	unassigned	na	212
YGR126W	YGR126W	1	msa	na	230
YGR127W	YGR127W	1	msa	na	312
YGR128C	UTP8	1	unassigned	na	713
YGR129W	SYF2	1	unassigned	na	215
YGR130C	YGR130C	1	psiblast	1i84S_	191
YGR130C	YGR130C	2	psiblast	1i84S_	295
YGR130C	YGR130C	3	psiblast	1cii__	53
YGR130C	YGR130C	4	psiblast	2tmaA_	52
YGR130C	YGR130C	5	psiblast	2tmaA_	90
YGR130C	YGR130C	6	psiblast	2tmaA_	62
YGR130C	YGR130C	7	psiblast	2tmaA_	73
YGR131W	YGR131W	1	unassigned	na	174
YGR132C	PHB1	1	psiblast	1lu7A_	212
YGR132C	PHB1	2	psiblast	1lu7A_	75
YGR133W	PEX4	1	psiblast	1fxtA_	183
YGR134W	CAF130	1	unassigned	na	1122
YGR135W	PRE9	1	psiblast	1fntC_	258
YGR136W	LSB1	1	psiblast	1griA_	110
YGR136W	LSB1	2	fold_recogniti	1m2vB	131
YGR137W	YGR137W	1	unassigned	na	124
YGR138C	TPO2	1	msa	na	147
YGR138C	TPO2	2	psiblast	1ja5A_	426
YGR138C	TPO2	3	psiblast	1ja5A_	41
YGR139W	YGR139W	1	unassigned	na	112
YGR140W	CBF2	1	unassigned	na	956
YGR141W	VPS62	1	msa	na	361
YGR141W	VPS62	2	unassigned	na	106
YGR142W	BTN2	1	unassigned	na	114
YGR142W	BTN2	2	psiblast	1i84S_	296
YGR143W	SKN1	1	fold_recogniti	1l8mA	63
YGR143W	SKN1	2	fold_recogniti	1l8mA	79
YGR143W	SKN1	3	fold_recogniti	1l8mA	72
YGR143W	SKN1	4	fold_recogniti	1l8mA	51

YGR143W	SKN1	5	unassigned	na	209
YGR143W	SKN1	6	psiblast	1macA_	297
YGR144W	THI4	1	psiblast	1gt8A_	326
YGR145W	ENP2	1	psiblast	1gg2B_	367
YGR145W	ENP2	2	unassigned	na	52
YGR145W	ENP2	3	psiblast	1i84S_	288
YGR146C	YGR146C	1	unassigned	na	211
YGR147C	NAT2	1	unassigned	na	90
YGR147C	NAT2	2	msa	na	198
YGR148C	RPL24B	1	psiblast	1k5yS_	72
YGR148C	RPL24B	2	unassigned	na	83
YGR149W	YGR149W	1	unassigned	na	77
YGR149W	YGR149W	2	msa	na	355
YGR150C	YGR150C	1	fold_recogniti	1fchA	196
YGR150C	YGR150C	2	fold_recogniti	1b3uA	191
YGR150C	YGR150C	3	fold_recogniti	1b3uA	74
YGR150C	YGR150C	4	fold_recogniti	1b3uA	164
YGR150C	YGR150C	5	fold_recogniti	1b3uA	239
YGR151C	YGR151C	1	unassigned	na	111
YGR152C	RSR1	1	psiblast	1guaA_	213
YGR152C	RSR1	2	unassigned	na	59
YGR153W	YGR153W	1	unassigned	na	217
YGR154C	YGR154C	1	psiblast	1eemA_	100
YGR154C	YGR154C	2	psiblast	1eemA_	103
YGR154C	YGR154C	3	psiblast	1k0mA_	153
YGR155W	CYS4	1	psiblast	1jbqA_	275
YGR155W	CYS4	2	psiblast	1jbqA_	93
YGR155W	CYS4	3	fold_recogniti	1b3oA	139
YGR156W	PTI1	1	fold_recogniti	3sxlA	103
YGR156W	PTI1	2	fold_recogniti	3sxlA	157
YGR156W	PTI1	3	unassigned	na	165
YGR157W	CHO2	1	unassigned	na	869
YGR158C	MTR3	1	psiblast	1e3pA_	101
YGR158C	MTR3	2	psiblast	1e3pA_	149
YGR159C	NSR1	1	fold_recogniti	1ldvA	158
YGR159C	NSR1	2	psiblast	1cvjA_	88
YGR159C	NSR1	3	psiblast	1cvjA_	168
YGR160W	YGR160W	1	msa	na	203
YGR161C	RTS3	1	unassigned	na	263
YGR161W-A	YGR161W-A	1	unassigned	na	92
YGR162W	TIF4631	1	fold_recogniti	1l8mA	82
YGR162W	TIF4631	2	fold_recogniti	1jv2B	320
YGR162W	TIF4631	3	fold_recogniti	1jv2B	103
YGR162W	TIF4631	4	unassigned	na	86
YGR162W	TIF4631	5	psiblast	1hu3A_	140
YGR162W	TIF4631	6	psiblast	1hu3A_	159
YGR162W	TIF4631	7	unassigned	na	62
YGR163W	GTR2	1	psiblast	1g17A_	220

YGR163W	GTR2	2	msa	na	121
YGR164W	YGR164W	1	unassigned	na	111
YGR165W	MRPS35	1	unassigned	na	345
YGR166W	KRE11	1	unassigned	na	560
YGR167W	CLC1	1	pfam	PF01086	233
YGR168C	YGR168C	1	unassigned	na	376
YGR169C	PUS6	1	psiblast	1kskA_	112
YGR169C	PUS6	2	psiblast	1kskA_	292
YGR170W	PSD2	1	fold_recogniti	1ldvA	242
YGR170W	PSD2	2	unassigned	na	126
YGR170W	PSD2	3	psiblast	1dqvA_	114
YGR170W	PSD2	4	psiblast	1dqvA_	161
YGR170W	PSD2	5	psiblast	1bynA_	174
YGR170W	PSD2	6	msa	na	321
YGR171C	MSM1	1	psiblast	1a8h__	332
YGR171C	MSM1	2	psiblast	1a8h__	85
YGR171C	MSM1	3	psiblast	1a8h__	158
YGR172C	YIP1	1	msa	na	107
YGR172C	YIP1	2	msa	na	141
YGR173W	RBG2	1	psiblast	1egaA_	298
YGR173W	RBG2	2	unassigned	na	70
YGR174C	CBP4	1	unassigned	na	170
YGR175C	ERG1	1	psiblast	1fohA_	157
YGR175C	ERG1	2	psiblast	1fohA_	227
YGR175C	ERG1	3	psiblast	1fohA_	54
YGR175C	ERG1	4	unassigned	na	58
YGR176W	YGR176W	1	unassigned	na	115
YGR177C	ATF2	1	unassigned	na	120
YGR177C	ATF2	2	msa	na	415
YGR178C	PBP1	1	msa	na	195
YGR178C	PBP1	2	fold_recogniti	1deqA	113
YGR178C	PBP1	3	fold_recogniti	1ldvA	287
YGR178C	PBP1	4	fold_recogniti	1la5A	127
YGR179C	OKP1	1	unassigned	na	406
YGR180C	RNR4	1	psiblast	1jk0B_	198
YGR180C	RNR4	2	psiblast	1jk0B_	74
YGR180C	RNR4	3	psiblast	1jk0B_	73
YGR181W	TIM13	1	pfam	PF02953	105
YGR182C	YGR182C	1	unassigned	na	117
YGR183C	QCR9	1	psiblast	1kyoI_	66
YGR184C	UBR1	1	unassigned	na	75
YGR184C	UBR1	2	pfam	PF02207	130
YGR184C	UBR1	3	unassigned	na	479
YGR184C	UBR1	4	msa	na	573
YGR184C	UBR1	5	unassigned	na	299
YGR184C	UBR1	6	msa	na	270
YGR184C	UBR1	7	unassigned	na	124
YGR185C	TYS1	1	psiblast	1lkwA_	277

YGR185C	TYS1	2	psiblast	1lkwA_	117
YGR186W	TFG1	1	unassigned	na	127
YGR186W	TFG1	2	msa	na	219
YGR186W	TFG1	3	unassigned	na	389
YGR187C	HGH1	1	fold_recogniti	1jpwA	136
YGR187C	HGH1	2	fold_recogniti	1jpwA	147
YGR187C	HGH1	3	fold_recogniti	1jpwA	111
YGR188C	BUB1	1	unassigned	na	136
YGR188C	BUB1	2	psiblast	1cxzB_	75
YGR188C	BUB1	3	unassigned	na	241
YGR188C	BUB1	4	psiblast	1jnuA_	137
YGR188C	BUB1	5	psiblast	1a25A_	74
YGR188C	BUB1	6	psiblast	1ctpE_	289
YGR188C	BUB1	7	psiblast	1ctpE_	69
YGR189C	CRH1	1	psiblast	2ayh__	257
YGR189C	CRH1	2	psiblast	1cpm__	53
YGR189C	CRH1	3	fold_recogniti	1k83A	197
YGR190C	YGR190C	1	unassigned	na	121
YGR191W	HIP1	1	unassigned	na	74
YGR191W	HIP1	2	pfam	PF00324	529
YGR192C	TDH3	1	psiblast	3gpdR_	161
YGR192C	TDH3	2	psiblast	3gpdR_	171
YGR193C	PDX1	1	psiblast	1fyc__	147
YGR193C	PDX1	2	psiblast	1bal__	71
YGR193C	PDX1	3	psiblast	1c4tA_	192
YGR194C	XKS1	1	psiblast	1bo5O_	297
YGR194C	XKS1	2	psiblast	1bo5O_	84
YGR194C	XKS1	3	psiblast	1bo5O_	219
YGR195W	SKI6	1	psiblast	1e3pA_	87
YGR195W	SKI6	2	psiblast	1e3pA_	159
YGR196C	FYV8	1	fold_recogniti	1ldvA	216
YGR196C	FYV8	2	fold_recogniti	1l8mA	69
YGR196C	FYV8	3	fold_recogniti	1l8mA	79
YGR196C	FYV8	4	fold_recogniti	1l8mA	70
YGR196C	FYV8	5	fold_recogniti	1l8mA	94
YGR196C	FYV8	6	msa	na	289
YGR197C	SNG1	1	unassigned	na	547
YGR198W	YGR198W	1	psiblast	1elwA_	179
YGR198W	YGR198W	2	unassigned	na	78
YGR198W	YGR198W	3	psiblast	1fchA_	146
YGR198W	YGR198W	4	psiblast	1fchA_	68
YGR198W	YGR198W	5	psiblast	1fchA_	137
YGR198W	YGR198W	6	msa	na	209
YGR199W	PMT6	1	pfam	PF02366	337
YGR199W	PMT6	2	pfam	PF02815	63
YGR199W	PMT6	3	fold_recogniti	1n4kA	138
YGR199W	PMT6	4	msa	na	221
YGR200C	ELP2	1	psiblast	1k32A_	542

YGR200C	ELP2	2	psiblast	1gg2B_	92
YGR200C	ELP2	3	psiblast	1erjA_	77
YGR200C	ELP2	4	msa	na	77
YGR201C	YGR201C	1	psiblast	1gwcA_	135
YGR201C	YGR201C	2	psiblast	1gwcA_	139
YGR202C	PCT1	1	unassigned	na	100
YGR202C	PCT1	2	psiblast	1cozA_	151
YGR202C	PCT1	3	unassigned	na	173
YGR203W	YGR203W	1	psiblast	1cwtA_	148
YGR204W	ADE3	1	psiblast	1diaA_	175
YGR204W	ADE3	2	psiblast	1diaA_	138
YGR204W	ADE3	3	psiblast	1fp7A_	374
YGR204W	ADE3	4	psiblast	1fp7A_	259
YGR205W	YGR205W	1	psiblast	1esmA_	290
YGR206W	YGR206W	1	unassigned	na	101
YGR207C	YGR207C	1	psiblast	1efpB_	261
YGR208W	SER2	1	msa	na	75
YGR208W	SER2	2	psiblast	1f5sA_	167
YGR208W	SER2	3	psiblast	1f5sA_	67
YGR209C	TRX2	1	psiblast	1erv__	104
YGR210C	YGR210C	1	psiblast	1egaA_	206
YGR210C	YGR210C	2	psiblast	1egaA_	141
YGR210C	YGR210C	3	msa	na	64
YGR211W	ZPR1	1	pfam	PF03367	256
YGR211W	ZPR1	2	pfam	PF03367	230
YGR212W	YGR212W	1	msa	na	239
YGR212W	YGR212W	2	unassigned	na	229
YGR213C	RTA1	1	unassigned	na	138
YGR213C	RTA1	2	msa	na	179
YGR214W	RPS0A	1	psiblast	1k5xB_	252
YGR215W	RSM27	1	unassigned	na	110
YGR216C	GPI1	1	unassigned	na	258
YGR216C	GPI1	2	msa	na	351
YGR217W	CCH1	1	unassigned	na	232
YGR217W	CCH1	2	psiblast	1exbE_	133
YGR217W	CCH1	3	unassigned	na	61
YGR217W	CCH1	4	msa	na	185
YGR217W	CCH1	5	msa	na	499
YGR217W	CCH1	6	unassigned	na	87
YGR217W	CCH1	7	msa	na	133
YGR217W	CCH1	8	msa	na	107
YGR217W	CCH1	9	psiblast	1byyA_	91
YGR217W	CCH1	10	msa	na	233
YGR217W	CCH1	11	msa	na	130
YGR217W	CCH1	12	unassigned	na	148
YGR218W	CRM1	1	msa	na	746
YGR218W	CRM1	2	unassigned	na	102
YGR218W	CRM1	3	fold_recogniti	1c7dA	98

YGR218W	CRM1	4	fold_recogniti	1c7dA	138
YGR219W	YGR219W	1	unassigned	na	113
YGR220C	MRPL9	1	psiblast	1lnrB_	153
YGR220C	MRPL9	2	psiblast	1lnrB_	116
YGR221C	TOS2	1	unassigned	na	179
YGR221C	TOS2	2	msa	na	443
YGR222W	PET54	1	unassigned	na	293
YGR223C	YGR223C	1	psiblast	1gg2B_	379
YGR223C	YGR223C	2	unassigned	na	69
YGR224W	AZR1	1	psiblast	1lviA_	465
YGR224W	AZR1	2	unassigned	na	148
YGR225W	AMA1	1	unassigned	na	98
YGR225W	AMA1	2	psiblast	1erjA_	366
YGR225W	AMA1	3	psiblast	1gg2B_	98
YGR226C	YGR226C	1	unassigned	na	199
YGR227W	DIE2	1	msa	na	412
YGR227W	DIE2	2	unassigned	na	113
YGR228W	YGR228W	1	unassigned	na	114
YGR229C	SMI1	1	unassigned	na	73
YGR229C	SMI1	2	msa	na	230
YGR229C	SMI1	3	msa	na	202
YGR230W	BNS1	1	unassigned	na	137
YGR231C	PHB2	1	psiblast	1lu7A_	240
YGR231C	PHB2	2	psiblast	1lu7A_	75
YGR232W	NAS6	1	psiblast	1iknD_	60
YGR232W	NAS6	2	psiblast	1iknD_	74
YGR232W	NAS6	3	psiblast	1iknD_	94
YGR233C	PHO81	1	pfam	PF03105	175
YGR233C	PHO81	2	unassigned	na	68
YGR233C	PHO81	3	fold_recogniti	1qoyA	176
YGR233C	PHO81	4	psiblast	1k1aA_	132
YGR233C	PHO81	5	psiblast	1k1aA_	144
YGR233C	PHO81	6	msa	na	253
YGR233C	PHO81	7	msa	na	230
YGR234W	YHB1	1	psiblast	1cqxA_	110
YGR234W	YHB1	2	psiblast	1cqxA_	150
YGR234W	YHB1	3	psiblast	1cqxA_	139
YGR235C	YGR235C	1	unassigned	na	233
YGR236C	SPG1	1	unassigned	na	129
YGR237C	YGR237C	1	unassigned	na	331
YGR237C	YGR237C	2	msa	na	248
YGR237C	YGR237C	3	unassigned	na	206
YGR238C	KEL2	1	fold_recogniti	1k3iA	214
YGR238C	KEL2	2	psiblast	1gof__	133
YGR238C	KEL2	3	psiblast	1i84S_	327
YGR238C	KEL2	4	psiblast	1c1gA_	208
YGR239C	PEX21	1	msa	na	140
YGR239C	PEX21	2	unassigned	na	148

YGR240C	PFK1	1	unassigned	na	187
YGR240C	PFK1	2	psiblast	3pfk__	242
YGR240C	PFK1	3	psiblast	3pfk__	122
YGR240C	PFK1	4	psiblast	1kzhA_	281
YGR240C	PFK1	5	fold_recogniti	1pfkA	84
YGR240C	PFK1	6	unassigned	na	71
YGR241C	YAP1802	1	psiblast	1hf8A_	149
YGR241C	YAP1802	2	psiblast	1hf8A_	135
YGR241C	YAP1802	3	msa	na	284
YGR242W	YGR242W	1	unassigned	na	102
YGR243W	FMP43	1	pfam	PF03650	146
YGR244C	LSC2	1	psiblast	1eucB_	190
YGR244C	LSC2	2	psiblast	1eucB_	237
YGR245C	SDA1	1	unassigned	na	77
YGR245C	SDA1	2	fold_recogniti	1gw5B	119
YGR245C	SDA1	3	fold_recogniti	1gw5B	129
YGR245C	SDA1	4	fold_recogniti	1gw5B	176
YGR245C	SDA1	5	msa	na	181
YGR245C	SDA1	6	unassigned	na	85
YGR246C	BRF1	1	psiblast	1dl6A_	61
YGR246C	BRF1	2	psiblast	1d3uB_	114
YGR246C	BRF1	3	psiblast	1d3uB_	106
YGR246C	BRF1	4	msa	na	315
YGR247W	CPD1	1	unassigned	na	239
YGR248W	SOL4	1	psiblast	1d9tA_	255
YGR249W	MGA1	1	psiblast	1hks__	117
YGR249W	MGA1	2	fold_recogniti	1i3qA	339
YGR250C	YGR250C	1	unassigned	na	94
YGR250C	YGR250C	2	psiblast	1fxlA_	92
YGR250C	YGR250C	3	psiblast	1cvjA_	95
YGR250C	YGR250C	4	psiblast	1cvjA_	149
YGR250C	YGR250C	5	psiblast	2u2fA_	100
YGR250C	YGR250C	6	psiblast	1bnyA_	167
YGR250C	YGR250C	7	psiblast	1g9lA_	84
YGR251W	YGR251W	1	unassigned	na	196
YGR252W	GCN5	1	unassigned	na	70
YGR252W	GCN5	2	psiblast	1yghA_	108
YGR252W	GCN5	3	psiblast	1yghA_	56
YGR252W	GCN5	4	unassigned	na	85
YGR252W	GCN5	5	psiblast	1e6iA_	120
YGR253C	PUP2	1	psiblast	1fntE_	260
YGR254W	ENO1	1	psiblast	1ebgA_	116
YGR254W	ENO1	2	psiblast	1ebgA_	321
YGR255C	COQ6	1	psiblast	1k0iA_	301
YGR255C	COQ6	2	psiblast	1k0iA_	178
YGR256W	GND2	1	psiblast	1pgn__	182
YGR256W	GND2	2	psiblast	1pgn__	310
YGR257C	MTM1	1	pfam	PF00153	156

YGR257C	MTM1	2	pfam	PF00153	106
YGR257C	MTM1	3	pfam	PF00153	104
YGR258C	RAD2	1	fold_recogniti	1bgxT	195
YGR258C	RAD2	2	msa	na	452
YGR258C	RAD2	3	psiblast	1a76__	384
YGR259C	YGR259C	1	unassigned	na	146
YGR260W	TNA1	1	unassigned	na	73
YGR260W	TNA1	2	psiblast	1ja5A_	461
YGR261C	APL6	1	psiblast	1gw5B_	122
YGR261C	APL6	2	psiblast	1gw5B_	101
YGR261C	APL6	3	psiblast	1gw5B_	167
YGR261C	APL6	4	psiblast	1gw5B_	186
YGR261C	APL6	5	msa	na	146
YGR261C	APL6	6	psiblast	1iu1A_	87
YGR262C	BUD32	1	psiblast	1a06__	115
YGR262C	BUD32	2	psiblast	1a06__	146
YGR263C	YGR263C	1	psiblast	1k4yA_	424
YGR264C	MES1	1	unassigned	na	190
YGR264C	MES1	2	psiblast	1f4lA_	242
YGR264C	MES1	3	psiblast	1f4lA_	174
YGR264C	MES1	4	psiblast	1f4lA_	145
YGR265W	YGR265W	1	unassigned	na	136
YGR266W	YGR266W	1	unassigned	na	701
YGR267C	FOL2	1	psiblast	1is7A_	243
YGR268C	HUA1	1	unassigned	na	145
YGR268C	HUA1	2	fold_recogniti	1exkA	53
YGR269W	YGR269W	1	unassigned	na	108
YGR270W	YTA7	1	unassigned	na	154
YGR270W	YTA7	2	psiblast	1e32A_	148
YGR270W	YTA7	3	psiblast	1e32A_	107
YGR270W	YTA7	4	psiblast	1e32A_	176
YGR270W	YTA7	5	psiblast	1e32A_	145
YGR270W	YTA7	6	psiblast	1iqpA_	192
YGR270W	YTA7	7	fold_recogniti	1eqfA	216
YGR270W	YTA7	8	msa	na	147
YGR270W	YTA7	9	unassigned	na	94
YGR271C-A	YGR271C-A	1	unassigned	na	63
YGR271W	SLH1	1	msa	na	255
YGR271W	SLH1	2	psiblast	1hv8A_	237
YGR271W	SLH1	3	psiblast	1hv8A_	203
YGR271W	SLH1	4	psiblast	1d2mA_	94
YGR271W	SLH1	5	msa	na	285
YGR271W	SLH1	6	fold_recogniti	1gm5A	196
YGR271W	SLH1	7	psiblast	1d9xA_	318
YGR271W	SLH1	8	unassigned	na	188
YGR271W	SLH1	9	psiblast	1jj2X_	80
YGR271W	SLH1	10	msa	na	111
YGR272C	YGR272C	1	unassigned	na	152

YGR273C	YGR273C	1	unassigned	na	174
YGR274C	TAF1	1	unassigned	na	317
YGR274C	TAF1	2	msa	na	570
YGR274C	TAF1	3	msa	na	179
YGR275W	RTT102	1	unassigned	na	186
YGR276C	RNH70	1	msa	na	163
YGR276C	RNH70	2	psiblast	1dpi__	299
YGR276C	RNH70	3	unassigned	na	91
YGR277C	YGR277C	1	msa	na	118
YGR277C	YGR277C	2	psiblast	1cozA_	187
YGR278W	CWC22	1	psiblast	1hu3A_	226
YGR278W	CWC22	2	unassigned	na	74
YGR278W	CWC22	3	fold_recogniti	1h6kA	214
YGR278W	CWC22	4	unassigned	na	63
YGR279C	SCW4	1	unassigned	na	96
YGR279C	SCW4	2	psiblast	1ghsA_	122
YGR279C	SCW4	3	psiblast	1ghsA_	87
YGR279C	SCW4	4	psiblast	1ghsA_	81
YGR280C	PXR1	1	pfam	PF01585	136
YGR280C	PXR1	2	unassigned	na	135
YGR281W	YOR1	1	fold_recogniti	1jj7A	99
YGR281W	YOR1	2	unassigned	na	90
YGR281W	YOR1	3	fold_recogniti	1i84S	112
YGR281W	YOR1	4	fold_recogniti	1i84S	243
YGR281W	YOR1	5	psiblast	1nbd__	267
YGR281W	YOR1	6	unassigned	na	52
YGR281W	YOR1	7	psiblast	1jsqA_	310
YGR281W	YOR1	8	psiblast	1jsqA_	304
YGR282C	BGL2	1	psiblast	1ghsA_	147
YGR282C	BGL2	2	psiblast	1ghsA_	46
YGR282C	BGL2	3	psiblast	1ghsA_	120
YGR283C	YGR283C	1	psiblast	1k3rA_	234
YGR283C	YGR283C	2	psiblast	1k3rA_	107
YGR284C	ERV29	1	pfam	PF02077	310
YGR285C	ZUO1	1	unassigned	na	82
YGR285C	ZUO1	2	psiblast	1xbl__	130
YGR285C	ZUO1	3	msa	na	221
YGR286C	BIO2	1	unassigned	na	116
YGR286C	BIO2	2	psiblast	1b5tA_	146
YGR286C	BIO2	3	msa	na	113
YGR287C	YGR287C	1	psiblast	1uok__	302
YGR287C	YGR287C	2	psiblast	1uok__	287
YGR288W	MAL13	1	psiblast	1zmeC_	73
YGR288W	MAL13	2	msa	na	279
YGR288W	MAL13	3	unassigned	na	121
YGR289C	MAL11	1	unassigned	na	94
YGR289C	MAL11	2	psiblast	1ja5A_	410
YGR289C	MAL11	3	psiblast	1ja5A_	112

YGR290W	YGR290W	1	unassigned	na	147
YGR291C	YGR291C	1	unassigned	na	73
YGR292W	MAL12	1	psiblast	1uok__	299
YGR292W	MAL12	2	psiblast	1uok__	285
YGR293C	YGR293C	1	unassigned	na	153
YGR294W	YGR294W	1	pfam	PF00660	120
YGR295C	COS6	1	msa	na	61
YGR295C	COS6	2	pfam	PF00674	108
YGR295C	COS6	3	unassigned	na	72
YGR295C	COS6	4	pfam	PF00674	140
YGR296W	YRF1-3	1	msa	na	273
YGR296W	YRF1-3	2	msa	na	298
YGR296W	YRF1-3	3	unassigned	na	180
YGR296W	YRF1-3	4	psiblast	1fuuA_	517
YGR296W	YRF1-3	5	msa	na	334
YGR296W	YRF1-3	6	unassigned	na	257
YHL001W	RPL14B	1	pfam	PF00467	50
YHL001W	RPL14B	2	pfam	PF01929	88
YHL002C-A	YHL002C-A	1	unassigned	na	162
YHL002W	HSE1	1	psiblast	1elkA_	78
YHL002W	HSE1	2	psiblast	1elkA_	68
YHL002W	HSE1	3	unassigned	na	61
YHL002W	HSE1	4	psiblast	1griA_	96
YHL002W	HSE1	5	psiblast	1griA_	149
YHL003C	LAG1	1	msa	na	164
YHL003C	LAG1	2	pfam	PF03798	247
YHL004W	MRP4	1	unassigned	na	128
YHL004W	MRP4	2	psiblast	1fjgB_	266
YHL005C	YHL005C	1	unassigned	na	130
YHL006C	SHU1	1	unassigned	na	159
YHL006W-A	YHL006W-A	1	unassigned	na	117
YHL007C	STE20	1	unassigned	na	69
YHL007C	STE20	2	fold_recogniti	1deqA	225
YHL007C	STE20	3	psiblast	1f3mA_	117
YHL007C	STE20	4	fold_recogniti	1m2vB	187
YHL007C	STE20	5	psiblast	1f3mC_	98
YHL007C	STE20	6	psiblast	1f3mC_	243
YHL008C	YHL008C	1	psiblast	1fqyA_	150
YHL008C	YHL008C	2	psiblast	1fqyA_	167
YHL008C	YHL008C	3	unassigned	na	310
YHL009C	YAP3	1	unassigned	na	138
YHL009C	YAP3	2	psiblast	1gd2E_	107
YHL009C	YAP3	3	unassigned	na	85
YHL010C	YHL010C	1	unassigned	na	209
YHL010C	YHL010C	2	psiblast	1i84S_	376
YHL011C	PRS3	1	psiblast	1dkrA_	174
YHL011C	PRS3	2	psiblast	1dkrA_	146
YHL012W	YHL012W	1	unassigned	na	61

YHL012W	YHL012W	2	fold_recogniti	1hv9A	432
YHL013C	YHL013C	1	msa	na	171
YHL013C	YHL013C	2	pfam	PF02338	136
YHL014C	YLF2	1	psiblast	1egaA_	202
YHL014C	YLF2	2	psiblast	1egaA_	118
YHL014C	YLF2	3	msa	na	85
YHL015W	RPS20	1	psiblast	1k5xJ_	121
YHL016C	DUR3	1	pfam	PF00474	486
YHL016C	DUR3	2	msa	na	91
YHL016C	DUR3	3	msa	na	71
YHL016C	DUR3	4	unassigned	na	87
YHL017W	YHL017W	1	unassigned	na	108
YHL017W	YHL017W	2	msa	na	355
YHL017W	YHL017W	3	unassigned	na	69
YHL018W	YHL018W	1	psiblast	1dchA_	120
YHL019C	APM2	1	psiblast	1gw5M_	143
YHL019C	APM2	2	psiblast	1gw5M_	401
YHL019C	APM2	3	fold_recogniti	1gw5M	61
YHL020C	OPI1	1	unassigned	na	404
YHL021C	FMP12	1	unassigned	na	126
YHL021C	FMP12	2	psiblast	1gy9A_	217
YHL021C	FMP12	3	psiblast	1gy9A_	122
YHL022C	SPO11	1	msa	na	95
YHL022C	SPO11	2	psiblast	1d3yA_	75
YHL022C	SPO11	3	psiblast	1d3yA_	137
YHL022C	SPO11	4	psiblast	1d3yA_	91
YHL023C	RMD11	1	unassigned	na	307
YHL023C	RMD11	2	pfam	PF03666	839
YHL024W	RIM4	1	msa	na	73
YHL024W	RIM4	2	psiblast	1cvjA_	156
YHL024W	RIM4	3	psiblast	2up1A_	102
YHL024W	RIM4	4	psiblast	2up1A_	97
YHL024W	RIM4	5	psiblast	1deqA_	146
YHL024W	RIM4	6	msa	na	139
YHL025W	SNF6	1	unassigned	na	332
YHL026C	YHL026C	1	unassigned	na	116
YHL026C	YHL026C	2	msa	na	152
YHL027W	RIM101	1	msa	na	167
YHL027W	RIM101	2	psiblast	1a1hA_	96
YHL027W	RIM101	3	fold_recogniti	1l2qA	362
YHL028W	WSC4	1	pfam	PF01822	114
YHL028W	WSC4	2	msa	na	167
YHL028W	WSC4	3	fold_recogniti	1ei3A	324
YHL029C	YHL029C	1	unassigned	na	679
YHL030W	ECM29	1	msa	na	151
YHL030W	ECM29	2	unassigned	na	374
YHL030W	ECM29	3	msa	na	353
YHL030W	ECM29	4	psiblast	1f59A_	144

YHL030W	ECM29	5	unassigned	na	131
YHL030W	ECM29	6	msa	na	476
YHL030W	ECM29	7	unassigned	na	239
YHL030W-A	YHL030W-A	1	unassigned	na	153
YHL031C	GOS1	1	psiblast	1i84S_	135
YHL031C	GOS1	2	psiblast	1gl2C_	88
YHL032C	GUT1	1	unassigned	na	103
YHL032C	GUT1	2	psiblast	1bo5O_	281
YHL032C	GUT1	3	psiblast	1bo5O_	108
YHL032C	GUT1	4	psiblast	1bo5O_	217
YHL033C	RPL8A	1	msa	na	82
YHL033C	RPL8A	2	psiblast	1k5yG_	174
YHL034C	SBP1	1	psiblast	1cvjA_	150
YHL034C	SBP1	2	psiblast	1cvjA_	144
YHL035C	VMR1	1	msa	na	294
YHL035C	VMR1	2	psiblast	1jsqA_	333
YHL035C	VMR1	3	psiblast	1jsqA_	315
YHL035C	VMR1	4	msa	na	334
YHL035C	VMR1	5	msa	na	316
YHL036W	MUP3	1	msa	na	380
YHL036W	MUP3	2	msa	na	166
YHL037C	YHL037C	1	unassigned	na	159
YHL038C	CBP2	1	fold_recogniti	1mukA	630
YHL039W	YHL039W	1	msa	na	298
YHL039W	YHL039W	2	unassigned	na	287
YHL040C	ARN1	1	unassigned	na	69
YHL040C	ARN1	2	psiblast	1lviA_	191
YHL040C	ARN1	3	unassigned	na	60
YHL040C	ARN1	4	msa	na	224
YHL040C	ARN1	5	unassigned	na	83
YHL041W	YHL041W	1	unassigned	na	149
YHL042W	YHL042W	1	pfam	PF00674	150
YHL043W	ECM34	1	msa	na	68
YHL043W	ECM34	2	pfam	PF00674	102
YHL044W	YHL044W	1	pfam	PF00674	235
YHL045W	YHL045W	1	unassigned	na	115
YHL046C	YHL046C	1	pfam	PF00660	120
YHL046W-A	YHL046W-A	1	unassigned	na	108
YHL047C	ARN2	1	unassigned	na	53
YHL047C	ARN2	2	psiblast	1lviA_	217
YHL047C	ARN2	3	unassigned	na	59
YHL047C	ARN2	4	msa	na	308
YHL048W	COS8	1	msa	na	61
YHL048W	COS8	2	pfam	PF00674	107
YHL048W	COS8	3	unassigned	na	71
YHL048W	COS8	4	pfam	PF00674	142
YHL049C	YHL049C	1	msa	na	209
YHL049C	YHL049C	2	msa	na	62

YHL050C	YHL050C	1	msa	na	53
YHL050C	YHL050C	2	msa	na	349
YHL050C	YHL050C	3	unassigned	na	295
YHR001W	OSH7	1	pfam	PF01237	437
YHR001W-A	QCR10	1	unassigned	na	77
YHR002W	LEU5	1	pfam	PF00153	129
YHR002W	LEU5	2	pfam	PF00153	121
YHR002W	LEU5	3	pfam	PF00153	107
YHR003C	YHR003C	1	psiblast	1jw9B_	361
YHR003C	YHR003C	2	unassigned	na	68
YHR004C	NEM1	1	unassigned	na	178
YHR004C	NEM1	2	pfam	PF03031	268
YHR005C	GPA1	1	pfam	PF00503	146
YHR005C	GPA1	2	psiblast	1scgA_	326
YHR005C-A	MRS11	1	pfam	PF02953	93
YHR006W	STP2	1	msa	na	143
YHR006W	STP2	2	psiblast	1lu6A_	82
YHR006W	STP2	3	psiblast	1lu6A_	52
YHR006W	STP2	4	psiblast	1tf6A_	193
YHR006W	STP2	5	unassigned	na	71
YHR007C	ERG11	1	psiblast	1ea1A_	295
YHR007C	ERG11	2	psiblast	1ea1A_	235
YHR008C	SOD2	1	psiblast	1kkcA_	233
YHR009C	YHR009C	1	psiblast	1f8rA_	264
YHR009C	YHR009C	2	psiblast	1f8rA_	99
YHR009C	YHR009C	3	psiblast	1el5A_	160
YHR010W	RPL27A	1	pfam	PF00467	49
YHR010W	RPL27A	2	pfam	PF01777	87
YHR011W	DIA4	1	psiblast	1serA_	446
YHR012W	VPS29	1	psiblast	1ii7A_	222
YHR012W	VPS29	2	unassigned	na	60
YHR013C	ARD1	1	psiblast	1i1dA_	238
YHR014W	SPO13	1	unassigned	na	291
YHR015W	MIP6	1	unassigned	na	96
YHR015W	MIP6	2	psiblast	1cvjA_	93
YHR015W	MIP6	3	psiblast	1cvjA_	105
YHR015W	MIP6	4	psiblast	1b7fA_	95
YHR015W	MIP6	5	psiblast	1b7fA_	154
YHR015W	MIP6	6	unassigned	na	116
YHR016C	YSC84	1	msa	na	80
YHR016C	YSC84	2	fold_recogniti	1k83A	333
YHR016C	YSC84	3	psiblast	1semA_	55
YHR017W	YSC83	1	unassigned	na	385
YHR018C	ARG4	1	psiblast	1aosA_	194
YHR018C	ARG4	2	psiblast	1aosA_	205
YHR018C	ARG4	3	psiblast	1aosA_	64
YHR019C	DED81	1	unassigned	na	104
YHR019C	DED81	2	psiblast	1b8aA_	122

YHR019C	DED81	3	psiblast	1b8aA_	328
YHR020W	YHR020W	1	psiblast	1qf6A_	586
YHR020W	YHR020W	2	psiblast	1h4qA_	102
YHR021C	RPS27B	1	pfam	PF01667	82
YHR021W-A	ECM12	1	unassigned	na	151
YHR022C	YHR022C	1	psiblast	3rabA_	256
YHR023W	MYO1	1	psiblast	1i84S_	1171
YHR023W	MYO1	2	msa	na	224
YHR023W	MYO1	3	psiblast	1cii__	448
YHR023W	MYO1	4	psiblast	1c1gA_	85
YHR024C	MAS2	1	psiblast	1hr6A_	238
YHR024C	MAS2	2	psiblast	1hr6A_	244
YHR025W	THR1	1	psiblast	1fwkA_	222
YHR025W	THR1	2	psiblast	1fwkA_	135
YHR026W	PPA1	1	pfam	PF00137	133
YHR026W	PPA1	2	msa	na	80
YHR027C	RPN1	1	msa	na	177
YHR027C	RPN1	2	unassigned	na	80
YHR027C	RPN1	3	fold_recogniti	1b89A	493
YHR027C	RPN1	4	msa	na	243
YHR028C	DAP2	1	psiblast	1erjA_	257
YHR028C	DAP2	2	psiblast	1qfsA_	287
YHR028C	DAP2	3	psiblast	1qfsA_	274
YHR029C	YHR029C	1	psiblast	1bwzA_	137
YHR029C	YHR029C	2	psiblast	1bwzA_	157
YHR030C	SLT2	1	psiblast	1erk__	172
YHR030C	SLT2	2	psiblast	1erk__	239
YHR030C	SLT2	3	unassigned	na	73
YHR031C	RRM3	1	msa	na	84
YHR031C	RRM3	2	unassigned	na	61
YHR031C	RRM3	3	psiblast	1fts__	81
YHR031C	RRM3	4	psiblast	1pjr__	497
YHR032W	YHR032W	1	unassigned	na	144
YHR032W	YHR032W	2	fold_recogniti	1b3uA	176
YHR032W	YHR032W	3	fold_recogniti	1b3uA	261
YHR033W	YHR033W	1	psiblast	1b7bA_	267
YHR033W	YHR033W	2	pfam	PF01472	156
YHR034C	PIH1	1	msa	na	204
YHR034C	PIH1	2	unassigned	na	140
YHR035W	YHR035W	1	msa	na	56
YHR035W	YHR035W	2	unassigned	na	83
YHR035W	YHR035W	3	msa	na	342
YHR035W	YHR035W	4	psiblast	1svy__	149
YHR036W	YHR036W	1	unassigned	na	182
YHR036W	YHR036W	2	msa	na	289
YHR037W	PUT2	1	psiblast	1bxsA_	369
YHR037W	PUT2	2	psiblast	1bxsA_	206
YHR038W	RRF1	1	psiblast	1eh1A_	154

YHR038W	RRF1	2	psiblast	1eh1A_	76
YHR039C	MSC7	1	unassigned	na	67
YHR039C	MSC7	2	psiblast	1bxsA_	379
YHR039C	MSC7	3	psiblast	1bxsA_	198
YHR039C-A	VMA10	1	pfam	PF03179	114
YHR040W	BCD1	1	msa	na	72
YHR040W	BCD1	2	msa	na	294
YHR041C	SRB2	1	unassigned	na	210
YHR042W	NCP1	1	psiblast	1amoA_	223
YHR042W	NCP1	2	psiblast	1amoA_	166
YHR042W	NCP1	3	psiblast	1amoA_	135
YHR042W	NCP1	4	psiblast	1amoA_	167
YHR043C	DOG2	1	psiblast	1jud__	188
YHR043C	DOG2	2	psiblast	1jud__	58
YHR044C	DOG1	1	psiblast	1jud__	188
YHR044C	DOG1	2	psiblast	1jud__	58
YHR045W	YHR045W	1	unassigned	na	560
YHR046C	INM1	1	psiblast	1imaA_	149
YHR046C	INM1	2	psiblast	1imaA_	146
YHR047C	AAP1'	1	psiblast	1hs6A_	205
YHR047C	AAP1'	2	psiblast	1hs6A_	93
YHR047C	AAP1'	3	psiblast	1hs6A_	177
YHR047C	AAP1'	4	fold_recogniti	1gw5B	123
YHR047C	AAP1'	5	fold_recogniti	1gw5B	98
YHR047C	AAP1'	6	fold_recogniti	1gw5B	160
YHR048W	YHR048W	1	psiblast	1ja5A_	514
YHR049C-A	YHR049C-A	1	unassigned	na	103
YHR049W	FSH1	1	fold_recogniti	1fj2A	243
YHR050W	SMF2	1	unassigned	na	75
YHR050W	SMF2	2	pfam	PF01566	420
YHR050W	SMF2	3	unassigned	na	54
YHR051W	COX6	1	psiblast	1occE_	148
YHR052W	CIC1	1	psiblast	1eg0N_	223
YHR052W	CIC1	2	psiblast	1eg0N_	88
YHR052W	CIC1	3	unassigned	na	65
YHR053C	CUP1-1	1	psiblast	1aqs__	61
YHR054C	YHR054C	1	unassigned	na	354
YHR056C	RSC30	1	unassigned	na	477
YHR056C	RSC30	2	msa	na	355
YHR056W-A	YHR056W-A	1	unassigned	na	143
YHR057C	CPR2	1	psiblast	1cynA_	205
YHR058C	MED6	1	msa	na	232
YHR058C	MED6	2	unassigned	na	63
YHR059W	FYV4	1	unassigned	na	130
YHR060W	VMA22	1	unassigned	na	181
YHR061C	GIC1	1	unassigned	na	99
YHR061C	GIC1	2	pfam	PF00786	142
YHR061C	GIC1	3	unassigned	na	73

YHR062C	RPP1	1	unassigned	na	61
YHR062C	RPP1	2	pfam	PF01876	232
YHR063C	PAN5	1	psiblast	1ks9A_	220
YHR063C	PAN5	2	psiblast	1ks9A_	159
YHR063W-A	YHR063W-A	1	unassigned	na	111
YHR064C	SSZ1	1	psiblast	1hpm__	229
YHR064C	SSZ1	2	psiblast	1hpm__	124
YHR064C	SSZ1	3	psiblast	1hpm__	75
YHR064C	SSZ1	4	psiblast	1ckrA_	144
YHR065C	RRP3	1	unassigned	na	114
YHR065C	RRP3	2	psiblast	1hv8A_	219
YHR065C	RRP3	3	psiblast	1hv8A_	210
YHR066W	SSF1	1	pfam	PF03877	294
YHR066W	SSF1	2	unassigned	na	78
YHR066W	SSF1	3	msa	na	81
YHR067W	RMD12	1	unassigned	na	280
YHR068W	DYS1	1	psiblast	1dhs__	387
YHR069C	RRP4	1	psiblast	1e3pA_	189
YHR069C	RRP4	2	msa	na	98
YHR069C	RRP4	3	unassigned	na	72
YHR070C-A	YHR070C-A	1	unassigned	na	136
YHR070W	TRM5	1	unassigned	na	106
YHR070W	TRM5	2	psiblast	1g60A_	221
YHR070W	TRM5	3	psiblast	1dusA_	52
YHR070W	TRM5	4	unassigned	na	120
YHR071W	PCL5	1	psiblast	1vin__	229
YHR072W	ERG7	1	psiblast	1sqc__	248
YHR072W	ERG7	2	psiblast	1sqc__	179
YHR072W	ERG7	3	psiblast	1sqc__	138
YHR072W	ERG7	4	psiblast	1sqc__	166
YHR072W-A	NOP10	1	unassigned	na	58
YHR073W	OSH3	1	psiblast	1pls__	109
YHR073W	OSH3	2	unassigned	na	90
YHR073W	OSH3	3	psiblast	1qqgA_	142
YHR073W	OSH3	4	psiblast	1qqgA_	129
YHR073W	OSH3	5	unassigned	na	133
YHR073W	OSH3	6	msa	na	393
YHR074W	QNS1	1	psiblast	1f89A_	313
YHR074W	QNS1	2	psiblast	1ee1A_	345
YHR074W	QNS1	3	unassigned	na	56
YHR075C	PPE1	1	unassigned	na	89
YHR075C	PPE1	2	psiblast	1cqzA_	311
YHR076W	PTC7	1	unassigned	na	82
YHR076W	PTC7	2	psiblast	1a6q__	292
YHR077C	NMD2	1	pfam	PF02854	272
YHR077C	NMD2	2	unassigned	na	64
YHR077C	NMD2	3	fold_recogniti	1h2vC	253
YHR077C	NMD2	4	psiblast	1i84S_	355

YHR077C	NMD2	5	psiblast	2tmaA_	145
YHR078W	YHR078W	1	unassigned	na	241
YHR078W	YHR078W	2	fold_recogniti	1qoyA	311
YHR079C	IRE1	1	fold_recogniti	1h4iA	471
YHR079C	IRE1	2	unassigned	na	166
YHR079C	IRE1	3	psiblast	1gy3A_	93
YHR079C	IRE1	4	psiblast	1gy3A_	330
YHR079C	IRE1	5	unassigned	na	55
YHR079C-A	SAE3	1	unassigned	na	50
YHR080C	YHR080C	1	msa	na	546
YHR080C	YHR080C	2	pfam	PF02893	118
YHR080C	YHR080C	3	msa	na	293
YHR080C	YHR080C	4	unassigned	na	81
YHR080C	YHR080C	5	msa	na	307
YHR081W	LRP1	1	unassigned	na	184
YHR082C	KSP1	1	psiblast	1gxcA_	81
YHR082C	KSP1	2	psiblast	1b6cB_	227
YHR082C	KSP1	3	psiblast	1a06__	83
YHR082C	KSP1	4	psiblast	1iq5A_	72
YHR082C	KSP1	5	psiblast	1iq5A_	93
YHR082C	KSP1	6	unassigned	na	473
YHR083W	SAM35	1	unassigned	na	111
YHR083W	SAM35	2	msa	na	218
YHR084W	STE12	1	pfam	PF02200	200
YHR084W	STE12	2	unassigned	na	488
YHR085W	IPI1	1	msa	na	230
YHR085W	IPI1	2	unassigned	na	104
YHR086W	NAM8	1	psiblast	1cvjA_	164
YHR086W	NAM8	2	psiblast	1qm9A_	139
YHR086W	NAM8	3	psiblast	1qm9A_	93
YHR086W	NAM8	4	msa	na	127
YHR087W	YHR087W	1	unassigned	na	111
YHR088W	RPF1	1	unassigned	na	93
YHR088W	RPF1	2	pfam	PF01945	202
YHR089C	GAR1	1	msa	na	130
YHR089C	GAR1	2	fold_recogniti	1la5A	75
YHR090C	YNG2	1	fold_recogniti	2vsgA	161
YHR090C	YNG2	2	fold_recogniti	2vsgA	45
YHR090C	YNG2	3	psiblast	1f62A_	76
YHR091C	MSR1	1	psiblast	1bs2A_	151
YHR091C	MSR1	2	psiblast	1bs2A_	214
YHR091C	MSR1	3	psiblast	1bs2A_	198
YHR091C	MSR1	4	psiblast	1bs2A_	80
YHR092C	HXT4	1	psiblast	1ja5A_	478
YHR092C	HXT4	2	psiblast	1ja5A_	82
YHR093W	AHT1	1	unassigned	na	182
YHR094C	HXT1	1	psiblast	1ja5A_	469
YHR094C	HXT1	2	psiblast	1ja5A_	101

YHR095W	YHR095W	1	unassigned	na	144
YHR096C	HXT5	1	unassigned	na	58
YHR096C	HXT5	2	psiblast	1ja5A_	435
YHR096C	HXT5	3	psiblast	1ja5A_	99
YHR097C	YHR097C	1	unassigned	na	122
YHR097C	YHR097C	2	msa	na	164
YHR097C	YHR097C	3	unassigned	na	80
YHR098C	SFB3	1	psiblast	1deqA_	175
YHR098C	SFB3	2	unassigned	na	157
YHR098C	SFB3	3	msa	na	318
YHR098C	SFB3	4	unassigned	na	57
YHR098C	SFB3	5	msa	na	222
YHR099W	TRA1	1	msa	na	522
YHR099W	TRA1	2	unassigned	na	825
YHR099W	TRA1	3	msa	na	762
YHR099W	TRA1	4	unassigned	na	302
YHR099W	TRA1	5	msa	na	186
YHR099W	TRA1	6	pfam	PF02259	596
YHR099W	TRA1	7	psiblast	1he8A_	122
YHR099W	TRA1	8	psiblast	1he8A_	121
YHR099W	TRA1	9	psiblast	1he8A_	272
YHR099W	TRA1	10	pfam	PF02260	36
YHR100C	YHR100C	1	psiblast	1zrm__	185
YHR101C	BIG1	1	unassigned	na	335
YHR102W	KIC1	1	psiblast	1f3mC_	101
YHR102W	KIC1	2	psiblast	1f3mC_	206
YHR102W	KIC1	3	psiblast	1cdmA_	83
YHR102W	KIC1	4	psiblast	1cdmA_	80
YHR102W	KIC1	5	fold_recogniti	1ei3A	195
YHR102W	KIC1	6	fold_recogniti	1l8mA	83
YHR102W	KIC1	7	fold_recogniti	1l8mA	73
YHR102W	KIC1	8	fold_recogniti	1l8mA	79
YHR102W	KIC1	9	fold_recogniti	1l8mA	72
YHR102W	KIC1	10	unassigned	na	108
YHR103W	SBE22	1	fold_recogniti	1k83A	356
YHR103W	SBE22	2	unassigned	na	63
YHR103W	SBE22	3	msa	na	189
YHR103W	SBE22	4	unassigned	na	244
YHR104W	GRE3	1	psiblast	1jezA_	327
YHR105W	YPT35	1	fold_recogniti	1h6hA	214
YHR106W	TRR2	1	psiblast	1vdc__	216
YHR106W	TRR2	2	psiblast	1vdc__	126
YHR107C	CDC12	1	psiblast	1egaA_	274
YHR107C	CDC12	2	msa	na	133
YHR108W	GGA2	1	psiblast	1elkA_	96
YHR108W	GGA2	2	psiblast	1elkA_	100
YHR108W	GGA2	3	pfam	PF03127	141
YHR108W	GGA2	4	unassigned	na	108

YHR108W	GGA2	5	psiblast	1iu1A_	140
YHR109W	CTM1	1	unassigned	na	585
YHR110W	ERP5	1	pfam	PF01105	212
YHR111W	UBA4	1	psiblast	1jw9B_	290
YHR111W	UBA4	2	psiblast	1h4kX_	150
YHR112C	YHR112C	1	psiblast	1ibjA_	262
YHR112C	YHR112C	2	psiblast	1ibjA_	116
YHR113W	YHR113W	1	pfam	PF02127	490
YHR114W	BZZ1	1	psiblast	1i84S_	420
YHR114W	BZZ1	2	unassigned	na	75
YHR114W	BZZ1	3	psiblast	1pnj__	70
YHR114W	BZZ1	4	psiblast	1gcqA_	68
YHR115C	YHR115C	1	unassigned	na	118
YHR115C	YHR115C	2	psiblast	1fbvA_	245
YHR115C	YHR115C	3	psiblast	1fbvA_	53
YHR116W	YHR116W	1	unassigned	na	151
YHR117W	TOM71	1	unassigned	na	121
YHR117W	TOM71	2	psiblast	1a17__	66
YHR117W	TOM71	3	fold_recogniti	1jcqA	153
YHR117W	TOM71	4	psiblast	1elrA_	103
YHR117W	TOM71	5	msa	na	196
YHR118C	ORC6	1	unassigned	na	435
YHR119W	SET1	1	unassigned	na	286
YHR119W	SET1	2	msa	na	521
YHR119W	SET1	3	unassigned	na	94
YHR119W	SET1	4	pfam	PF00856	179
YHR120W	MSH1	1	unassigned	na	68
YHR120W	MSH1	2	psiblast	1e3mA_	117
YHR120W	MSH1	3	psiblast	1e3mA_	175
YHR120W	MSH1	4	psiblast	1e3mA_	269
YHR120W	MSH1	5	psiblast	1e3mA_	239
YHR120W	MSH1	6	psiblast	1e3mA_	91
YHR121W	YHR121W	1	unassigned	na	187
YHR122W	YHR122W	1	unassigned	na	100
YHR122W	YHR122W	2	pfam	PF01883	131
YHR123W	EPT1	1	unassigned	na	75
YHR123W	EPT1	2	pfam	PF01066	182
YHR123W	EPT1	3	msa	na	134
YHR124W	NDT80	1	msa	na	434
YHR124W	NDT80	2	unassigned	na	193
YHR125W	YHR125W	1	unassigned	na	101
YHR126C	YHR126C	1	unassigned	na	159
YHR127W	YHR127W	1	unassigned	na	243
YHR128W	FUR1	1	psiblast	1bd3D_	251
YHR129C	ARP1	1	psiblast	1d4xA_	181
YHR129C	ARP1	2	psiblast	1d4xA_	203
YHR130C	YHR130C	1	unassigned	na	111
YHR131C	YHR131C	1	unassigned	na	181

YHR131C	YHR131C	2	pfam	PF00169	146
YHR131C	YHR131C	3	msa	na	147
YHR131C	YHR131C	4	unassigned	na	265
YHR131C	YHR131C	5	msa	na	101
YHR131W-A	YHR131W-A	1	unassigned	na	115
YHR132C	ECM14	1	psiblast	1aye__	89
YHR132C	ECM14	2	psiblast	1aye__	341
YHR132W-A	YHR132W-A	1	unassigned	na	131
YHR133C	NSG1	1	unassigned	na	291
YHR134W	WSS1	1	msa	na	186
YHR134W	WSS1	2	unassigned	na	83
YHR135C	YCK1	1	psiblast	1csn__	127
YHR135C	YCK1	2	psiblast	1csn__	75
YHR135C	YCK1	3	psiblast	1csn__	214
YHR135C	YCK1	4	unassigned	na	122
YHR136C	SPL2	1	unassigned	na	148
YHR137W	ARO9	1	psiblast	1bkgA_	376
YHR137W	ARO9	2	psiblast	1bkgA_	137
YHR138C	YHR138C	1	unassigned	na	114
YHR139C	SPS100	1	psiblast	1agx__	211
YHR139C	SPS100	2	psiblast	1agx__	115
YHR139C-A	YHR139C-A	1	unassigned	na	103
YHR140W	YHR140W	1	msa	na	239
YHR141C	RPL42B	1	psiblast	1k5y1_	106
YHR142W	CHS7	1	unassigned	na	201
YHR142W	CHS7	2	msa	na	115
YHR143W	DSE2	1	psiblast	1acc__	208
YHR143W	DSE2	2	unassigned	na	117
YHR143W-A	RPC10	1	psiblast	1k83L_	70
YHR144C	DCD1	1	unassigned	na	111
YHR144C	DCD1	2	fold_recogniti	1jtkA	201
YHR145C	YHR145C	1	unassigned	na	118
YHR146W	CRP1	1	msa	na	86
YHR146W	CRP1	2	fold_recogniti	1deqA	62
YHR146W	CRP1	3	fold_recogniti	1m2vB	317
YHR147C	MRPL6	1	psiblast	1c04B_	117
YHR147C	MRPL6	2	psiblast	1c04B_	97
YHR148W	IMP3	1	psiblast	1k5xD_	67
YHR148W	IMP3	2	psiblast	1k5xD_	116
YHR149C	SKG6	1	unassigned	na	135
YHR149C	SKG6	2	msa	na	401
YHR149C	SKG6	3	unassigned	na	198
YHR150W	PEX28	1	unassigned	na	159
YHR150W	PEX28	2	msa	na	177
YHR150W	PEX28	3	msa	na	243
YHR151C	YHR151C	1	unassigned	na	526
YHR152W	SPO12	1	unassigned	na	173
YHR153C	SPO16	1	unassigned	na	198

YHR154W	RTT107	1	fold_recogniti	1l0bA	119
YHR154W	RTT107	2	fold_recogniti	1l0bA	141
YHR154W	RTT107	3	unassigned	na	99
YHR154W	RTT107	4	fold_recogniti	1dgsA	93
YHR154W	RTT107	5	psiblast	1i84S_	295
YHR154W	RTT107	6	psiblast	2tmaA_	87
YHR154W	RTT107	7	msa	na	236
YHR155W	YHR155W	1	msa	na	113
YHR155W	YHR155W	2	psiblast	1pbv__	76
YHR155W	YHR155W	3	psiblast	1pbv__	107
YHR155W	YHR155W	4	psiblast	1eazA_	171
YHR155W	YHR155W	5	psiblast	1dcqA_	104
YHR155W	YHR155W	6	unassigned	na	657
YHR156C	LIN1	1	msa	na	280
YHR156C	LIN1	2	psiblast	1gyfA_	60
YHR157W	REC104	1	unassigned	na	182
YHR158C	KEL1	1	unassigned	na	75
YHR158C	KEL1	2	msa	na	101
YHR158C	KEL1	3	pfam	PF01344	102
YHR158C	KEL1	4	pfam	PF01344	63
YHR158C	KEL1	5	psiblast	1dg3A_	240
YHR158C	KEL1	6	psiblast	1cii__	145
YHR158C	KEL1	7	psiblast	1cii__	242
YHR158C	KEL1	8	psiblast	1i84S_	196
YHR159W	YHR159W	1	unassigned	na	172
YHR159W	YHR159W	2	msa	na	332
YHR160C	PEX18	1	unassigned	na	283
YHR161C	YAP1801	1	psiblast	1hf8A_	148
YHR161C	YAP1801	2	psiblast	1hf8A_	123
YHR161C	YAP1801	3	unassigned	na	67
YHR161C	YAP1801	4	msa	na	299
YHR162W	YHR162W	1	pfam	PF03650	129
YHR163W	SOL3	1	psiblast	1d9tA_	280
YHR164C	DNA2	1	unassigned	na	435
YHR164C	DNA2	2	msa	na	319
YHR164C	DNA2	3	psiblast	1gm5A_	182
YHR164C	DNA2	4	psiblast	1gm5A_	295
YHR164C	DNA2	5	psiblast	1pjr__	192
YHR164C	DNA2	6	psiblast	1qhhD_	99
YHR165C	PRP8	1	msa	na	237
YHR165C	PRP8	2	unassigned	na	566
YHR165C	PRP8	3	msa	na	219
YHR165C	PRP8	4	unassigned	na	260
YHR165C	PRP8	5	msa	na	786
YHR165C	PRP8	6	unassigned	na	83
YHR165C	PRP8	7	msa	na	171
YHR165C	PRP8	8	unassigned	na	91
YHR166C	CDC23	1	unassigned	na	112

YHR166C	CDC23	2	psiblast	1a17__	91
YHR166C	CDC23	3	psiblast	1fchA_	140
YHR166C	CDC23	4	psiblast	1fchA_	68
YHR166C	CDC23	5	psiblast	1fchA_	86
YHR166C	CDC23	6	psiblast	1jcqA_	129
YHR167W	THP2	1	unassigned	na	261
YHR168W	YHR168W	1	pfam	PF01018	324
YHR168W	YHR168W	2	psiblast	1ksgA_	175
YHR169W	DBP8	1	psiblast	1hv8A_	222
YHR169W	DBP8	2	psiblast	1hv8A_	209
YHR170W	NMD3	1	msa	na	268
YHR170W	NMD3	2	unassigned	na	164
YHR170W	NMD3	3	msa	na	86
YHR171W	ATG7	1	unassigned	na	293
YHR171W	ATG7	2	psiblast	1jw9B_	337
YHR172W	SPC97	1	unassigned	na	53
YHR172W	SPC97	2	msa	na	377
YHR172W	SPC97	3	unassigned	na	114
YHR172W	SPC97	4	fold_recogniti	1ldjA	279
YHR173C	YHR173C	1	unassigned	na	112
YHR174W	ENO2	1	psiblast	1els__	127
YHR174W	ENO2	2	psiblast	1els__	310
YHR175W	CTR2	1	unassigned	na	189
YHR176W	FMO1	1	psiblast	1ebdA_	183
YHR176W	FMO1	2	psiblast	1ebdA_	190
YHR177W	GON3	1	msa	na	210
YHR177W	GON3	2	unassigned	na	243
YHR178W	STB5	1	psiblast	1cld__	111
YHR178W	STB5	2	unassigned	na	64
YHR178W	STB5	3	msa	na	568
YHR179W	OYE2	1	psiblast	1oya__	400
YHR180W	YHR180W	1	unassigned	na	163
YHR181W	SVP26	1	msa	na	228
YHR182W	YHR182W	1	unassigned	na	785
YHR183W	GND1	1	psiblast	1pgn__	179
YHR183W	GND1	2	psiblast	1pgn__	310
YHR184W	SSP1	1	unassigned	na	163
YHR184W	SSP1	2	fold_recogniti	1jkyA	229
YHR184W	SSP1	3	fold_recogniti	1jkyA	179
YHR185C	PFS1	1	unassigned	na	237
YHR186C	KOG1	1	fold_recogniti	1bk6A	792
YHR186C	KOG1	2	msa	na	225
YHR186C	KOG1	3	psiblast	1k8kC_	367
YHR186C	KOG1	4	psiblast	1gg2B_	173
YHR187W	IKI1	1	unassigned	na	309
YHR188C	GPI16	1	unassigned	na	114
YHR188C	GPI16	2	msa	na	153
YHR188C	GPI16	3	unassigned	na	343

YHR189W	PTH1	1	psiblast	2pth__	129
YHR189W	PTH1	2	psiblast	2pth__	61
YHR190W	ERG9	1	psiblast	1ezfA_	184
YHR190W	ERG9	2	psiblast	1ezfA_	260
YHR191C	CTF8	1	unassigned	na	133
YHR192W	YHR192W	1	unassigned	na	278
YHR193C	EGD2	1	pfam	PF01849	114
YHR193C	EGD2	2	pfam	PF00627	60
YHR194W	MDM31	1	unassigned	na	579
YHR195W	NVJ1	1	unassigned	na	321
YHR196W	UTP9	1	fold_recogniti	1tbgA	369
YHR196W	UTP9	2	fold_recogniti	1erjC	74
YHR196W	UTP9	3	unassigned	na	132
YHR197W	RIX1	1	unassigned	na	763
YHR198C	FMP22	1	unassigned	na	86
YHR198C	FMP22	2	msa	na	235
YHR199C	FMP34	1	unassigned	na	93
YHR199C	FMP34	2	msa	na	217
YHR200W	RPN10	1	fold_recogniti	1dgqA	197
YHR200W	RPN10	2	unassigned	na	71
YHR201C	PPX1	1	psiblast	1k20A_	251
YHR201C	PPX1	2	psiblast	1k20A_	146
YHR202W	YHR202W	1	psiblast	1ush__	362
YHR202W	YHR202W	2	psiblast	1ush__	240
YHR203C	RPS4B	1	pfam	PF01479	93
YHR203C	RPS4B	2	pfam	PF00900	79
YHR203C	RPS4B	3	pfam	PF00467	89
YHR204W	MNL1	1	psiblast	1fmiA_	417
YHR204W	MNL1	2	psiblast	1fmiA_	154
YHR204W	MNL1	3	unassigned	na	225
YHR205W	SCH9	1	unassigned	na	140
YHR205W	SCH9	2	psiblast	1tbn__	79
YHR205W	SCH9	3	psiblast	1dsyA_	133
YHR205W	SCH9	4	psiblast	1cdkA_	270
YHR205W	SCH9	5	psiblast	1cdkA_	128
YHR205W	SCH9	6	unassigned	na	74
YHR206W	SKN7	1	unassigned	na	67
YHR206W	SKN7	2	psiblast	1hks__	134
YHR206W	SKN7	3	msa	na	168
YHR206W	SKN7	4	psiblast	1kgsA_	131
YHR206W	SKN7	5	psiblast	1kgsA_	122
YHR207C	SET5	1	unassigned	na	89
YHR207C	SET5	2	msa	na	123
YHR207C	SET5	3	unassigned	na	70
YHR207C	SET5	4	fold_recogniti	1mvhA	164
YHR207C	SET5	5	unassigned	na	80
YHR208W	BAT1	1	psiblast	1ekfA_	212
YHR208W	BAT1	2	psiblast	1ekfA_	181

YHR209W	YHR209W	1	psiblast	1kp9A_	198
YHR209W	YHR209W	2	psiblast	1kp9A_	98
YHR210C	YHR210C	1	psiblast	1l7jA_	341
YHR211W	FLO5	1	msa	na	250
YHR211W	FLO5	2	pfam	PF00624	66
YHR211W	FLO5	3	pfam	PF00624	45
YHR211W	FLO5	4	pfam	PF00624	45
YHR211W	FLO5	5	pfam	PF00624	45
YHR211W	FLO5	6	pfam	PF00624	45
YHR211W	FLO5	7	pfam	PF00624	45
YHR211W	FLO5	8	pfam	PF00624	45
YHR211W	FLO5	9	pfam	PF00624	60
YHR211W	FLO5	10	msa	na	429
YHR214W-A	YHR214W-A	1	unassigned	na	161
YHR215W	PHO12	1	psiblast	1qfxA_	169
YHR215W	PHO12	2	psiblast	1qfxA_	298
YHR216W	IMD2	1	psiblast	1b3oA_	523
YHR217C	YHR217C	1	fold_recogniti	1k83A	153
YHR218W	YHR218W	1	msa	na	173
YHR218W	YHR218W	2	unassigned	na	107
YHR218W	YHR218W	3	pfam	PF00270	271
YHR218W	YHR218W	4	unassigned	na	52
YHR219W	YHR219W	1	fold_recogniti	1m2vB	375
YHR219W	YHR219W	2	fold_recogniti	1itwA	249
YIL001W	YIL001W	1	psiblast	1myo__	94
YIL001W	YIL001W	2	msa	na	123
YIL001W	YIL001W	3	psiblast	1buoA_	174
YIL001W	YIL001W	4	msa	na	122
YIL002C	INP51	1	pfam	PF02383	408
YIL002C	INP51	2	unassigned	na	88
YIL002C	INP51	3	psiblast	1i9yA_	383
YIL002C	INP51	4	unassigned	na	67
YIL003W	CFD1	1	psiblast	1g3qA_	293
YIL004C	BET1	1	psiblast	1dn1B_	59
YIL004C	BET1	2	psiblast	1dn1B_	83
YIL005W	EPS1	1	psiblast	1mek__	165
YIL005W	EPS1	2	psiblast	1a8y__	235
YIL005W	EPS1	3	psiblast	1jfuA_	197
YIL005W	EPS1	4	unassigned	na	104
YIL006W	YIL006W	1	unassigned	na	72
YIL006W	YIL006W	2	fold_recogniti	1is2A	301
YIL007C	NAS2	1	psiblast	1ky9A_	220
YIL008W	URM1	1	unassigned	na	99
YIL009C-A	EST3	1	unassigned	na	181
YIL009W	FAA3	1	psiblast	1ba3__	360
YIL009W	FAA3	2	psiblast	1ba3__	334
YIL010W	DOT5	1	psiblast	1qq2A_	215
YIL011W	TIR3	1	pfam	PF00660	110

YIL011W	TIR3	2	psiblast	1deqA_	159
YIL012W	YIL012W	1	unassigned	na	130
YIL013C	PDR11	1	psiblast	1l2tA_	294
YIL013C	PDR11	2	unassigned	na	66
YIL013C	PDR11	3	msa	na	224
YIL013C	PDR11	4	unassigned	na	144
YIL013C	PDR11	5	psiblast	1g291_	327
YIL013C	PDR11	6	msa	na	261
YIL013C	PDR11	7	unassigned	na	95
YIL014C-A	YIL014C-A	1	unassigned	na	104
YIL014W	MNT3	1	unassigned	na	220
YIL014W	MNT3	2	fold_recogniti	1ga8A	164
YIL014W	MNT3	3	unassigned	na	246
YIL015W	BAR1	1	psiblast	1ypsA_	205
YIL015W	BAR1	2	psiblast	1ypsA_	125
YIL015W	BAR1	3	psiblast	1ypsA_	135
YIL015W	BAR1	4	unassigned	na	122
YIL016W	SNL1	1	unassigned	na	66
YIL016W	SNL1	2	pfam	PF02179	93
YIL017C	VID28	1	unassigned	na	340
YIL017C	VID28	2	psiblast	1ibrB_	163
YIL017C	VID28	3	psiblast	1ejlI_	112
YIL017C	VID28	4	psiblast	1ejlI_	53
YIL017C	VID28	5	psiblast	1ejlI_	157
YIL017C	VID28	6	unassigned	na	96
YIL019W	FAF1	1	unassigned	na	346
YIL020C	HIS6	1	psiblast	1gpwA_	261
YIL021W	RPB3	1	psiblast	1k83C_	274
YIL021W	RPB3	2	psiblast	1k83C_	44
YIL022W	TIM44	1	unassigned	na	246
YIL022W	TIM44	2	msa	na	185
YIL023C	YIL023C	1	msa	na	169
YIL023C	YIL023C	2	pfam	PF02535	177
YIL024C	YIL024C	1	unassigned	na	189
YIL025C	YIL025C	1	unassigned	na	124
YIL026C	IRR1	1	msa	na	162
YIL026C	IRR1	2	pfam	PF03365	988
YIL027C	KRE27	1	unassigned	na	141
YIL028W	YIL028W	1	unassigned	na	132
YIL029C	YIL029C	1	unassigned	na	142
YIL030C	SSM4	1	msa	na	164
YIL030C	SSM4	2	unassigned	na	441
YIL030C	SSM4	3	msa	na	232
YIL030C	SSM4	4	unassigned	na	482
YIL031W	ULP2	1	unassigned	na	401
YIL031W	ULP2	2	psiblast	1euvA_	329
YIL031W	ULP2	3	unassigned	na	304
YIL032C	YIL032C	1	unassigned	na	118

YIL033C	BCY1	1	psiblast	2apk__	416
YIL034C	CAP2	1	pfam	PF01115	287
YIL035C	CKA1	1	psiblast	1jwhA_	164
YIL035C	CKA1	2	psiblast	1jwhA_	147
YIL035C	CKA1	3	psiblast	1jwhA_	61
YIL036W	CST6	1	msa	na	300
YIL036W	CST6	2	unassigned	na	79
YIL036W	CST6	3	psiblast	1io4A_	125
YIL036W	CST6	4	unassigned	na	83
YIL037C	PRM2	1	unassigned	na	99
YIL037C	PRM2	2	fold_recogniti	1l9uD	481
YIL037C	PRM2	3	unassigned	na	76
YIL038C	NOT3	1	psiblast	1gk4A_	107
YIL038C	NOT3	2	unassigned	na	151
YIL038C	NOT3	3	fold_recogniti	1deqA	114
YIL038C	NOT3	4	fold_recogniti	1ei3A	315
YIL038C	NOT3	5	msa	na	149
YIL039W	YIL039W	1	unassigned	na	175
YIL039W	YIL039W	2	psiblast	1qfcA_	298
YIL040W	APQ12	1	unassigned	na	138
YIL041W	GVP36	1	unassigned	na	113
YIL041W	GVP36	2	fold_recogniti	1i49A	213
YIL042C	YIL042C	1	psiblast	1gkxA_	210
YIL042C	YIL042C	2	psiblast	1gkxA_	184
YIL043C	CBR1	1	psiblast	2cjn__	72
YIL043C	CBR1	2	psiblast	1cnf__	102
YIL043C	CBR1	3	psiblast	1cnf__	148
YIL044C	AGE2	1	psiblast	1dcqA_	130
YIL044C	AGE2	2	msa	na	168
YIL045W	PIG2	1	unassigned	na	354
YIL045W	PIG2	2	pfam	PF03370	184
YIL046W	MET30	1	unassigned	na	103
YIL046W	MET30	2	psiblast	1erjA_	390
YIL046W	MET30	3	psiblast	1gxrA_	147
YIL047C	SYG1	1	psiblast	1i84S_	434
YIL047C	SYG1	2	msa	na	400
YIL047C	SYG1	3	unassigned	na	68
YIL048W	NEO1	1	unassigned	na	114
YIL048W	NEO1	2	psiblast	1eulA_	166
YIL048W	NEO1	3	psiblast	1eulA_	399
YIL048W	NEO1	4	psiblast	1eulA_	231
YIL048W	NEO1	5	psiblast	1eulA_	241
YIL049W	DFG10	1	msa	na	63
YIL049W	DFG10	2	pfam	PF02544	190
YIL050W	PCL7	1	msa	na	285
YIL051C	MMF1	1	psiblast	1jd1A_	145
YIL052C	RPL34B	1	pfam	PF01199	121
YIL053W	RHR2	1	psiblast	1f5sA_	205

YIL053W	RHR2	2	psiblast	1f5sA_	66
YIL054W	YIL054W	1	unassigned	na	105
YIL055C	YIL055C	1	psiblast	1f6wA_	155
YIL055C	YIL055C	2	fold_recogniti	1deqA	248
YIL055C	YIL055C	3	unassigned	na	224
YIL056W	YIL056W	1	unassigned	na	227
YIL056W	YIL056W	2	fold_recogniti	1jfzA	172
YIL056W	YIL056W	3	unassigned	na	241
YIL057C	YIL057C	1	unassigned	na	164
YIL058W	YIL058W	1	unassigned	na	94
YIL059C	YIL059C	1	unassigned	na	121
YIL060W	YIL060W	1	unassigned	na	144
YIL061C	SNP1	1	psiblast	2up1A_	90
YIL061C	SNP1	2	psiblast	2up1A_	97
YIL061C	SNP1	3	psiblast	1fxlA_	113
YIL062C	ARC15	1	psiblast	1k8kG_	80
YIL062C	ARC15	2	psiblast	1k8kG_	74
YIL063C	YRB2	1	fold_recogniti	1l8mA	113
YIL063C	YRB2	2	fold_recogniti	1l8mA	76
YIL063C	YRB2	3	psiblast	1rrpB_	138
YIL064W	YIL064W	1	psiblast	1kpiA_	257
YIL065C	FIS1	1	unassigned	na	155
YIL066C	RNR3	1	psiblast	2r1rA_	217
YIL066C	RNR3	2	psiblast	2r1rA_	604
YIL066C	RNR3	3	unassigned	na	64
YIL066W-A	YIL066W-A	1	unassigned	na	147
YIL067C	YIL067C	1	unassigned	na	173
YIL067C	YIL067C	2	fold_recogniti	1jbiA	131
YIL067C	YIL067C	3	unassigned	na	374
YIL068C	SEC6	1	fold_recogniti	1qgkA	285
YIL068C	SEC6	2	fold_recogniti	1qgkA	140
YIL068C	SEC6	3	fold_recogniti	1qgkA	121
YIL068C	SEC6	4	fold_recogniti	1qgkA	259
YIL068W-A	YIL068W-A	1	unassigned	na	127
YIL070C	MAM33	1	psiblast	1p32A_	266
YIL071C	PCI8	1	unassigned	na	298
YIL071C	PCI8	2	pfam	PF01399	146
YIL071W-A	YIL071W-A	1	unassigned	na	158
YIL072W	HOP1	1	psiblast	1klqA_	297
YIL072W	HOP1	2	unassigned	na	65
YIL072W	HOP1	3	unassigned	na	243
YIL073C	SPO22	1	unassigned	na	127
YIL073C	SPO22	2	fold_recogniti	1n1bA	253
YIL073C	SPO22	3	fold_recogniti	1n1bA	62
YIL073C	SPO22	4	fold_recogniti	1n1bA	124
YIL073C	SPO22	5	fold_recogniti	1hz4A	244
YIL073C	SPO22	6	unassigned	na	76
YIL074C	SER33	1	psiblast	1psdA_	174

YIL074C	SER33	2	psiblast	1psdA_	295
YIL075C	RPN2	1	fold_recogniti	1b3uA	148
YIL075C	RPN2	2	fold_recogniti	1b3uA	87
YIL075C	RPN2	3	fold_recogniti	1b3uA	125
YIL075C	RPN2	4	psiblast	1b3uA_	200
YIL075C	RPN2	5	psiblast	1b3uA_	161
YIL075C	RPN2	6	psiblast	1jmmA_	80
YIL075C	RPN2	7	msa	na	144
YIL076W	SEC28	1	unassigned	na	114
YIL076W	SEC28	2	fold_recogniti	1hh8A	133
YIL076W	SEC28	3	fold_recogniti	1hh8A	48
YIL076W	SEC28	4	fold_recogniti	1hh8A	64
YIL077C	YIL077C	1	unassigned	na	320
YIL078W	THS1	1	msa	na	68
YIL078W	THS1	2	psiblast	1qf6A_	71
YIL078W	THS1	3	psiblast	1qf6A_	183
YIL078W	THS1	4	psiblast	1qf6A_	298
YIL078W	THS1	5	psiblast	1qf6A_	114
YIL079C	AIR1	1	psiblast	1baj__	99
YIL079C	AIR1	2	psiblast	1aaf__	69
YIL079C	AIR1	3	unassigned	na	65
YIL079C	AIR1	4	msa	na	127
YIL083C	YIL083C	1	psiblast	1e20A_	80
YIL083C	YIL083C	2	msa	na	285
YIL084C	SDS3	1	psiblast	1i84S_	197
YIL084C	SDS3	2	unassigned	na	130
YIL085C	KTR7	1	pfam	PF01793	432
YIL085C	KTR7	2	unassigned	na	85
YIL086C	YIL086C	1	unassigned	na	102
YIL087C	YIL087C	1	unassigned	na	157
YIL088C	AVT7	1	pfam	PF01490	490
YIL089W	YIL089W	1	unassigned	na	205
YIL090W	YIL090W	1	unassigned	na	491
YIL091C	YIL091C	1	psiblast	1i84S_	219
YIL091C	YIL091C	2	fold_recogniti	1hv8A	502
YIL092W	YIL092W	1	unassigned	na	633
YIL093C	RSM25	1	unassigned	na	264
YIL094C	LYS12	1	psiblast	1osjA_	371
YIL095W	PRK1	1	psiblast	2phkA_	91
YIL095W	PRK1	2	psiblast	2phkA_	260
YIL095W	PRK1	3	unassigned	na	459
YIL096C	YIL096C	1	unassigned	na	63
YIL096C	YIL096C	2	msa	na	273
YIL097W	FYV10	1	unassigned	na	116
YIL097W	FYV10	2	msa	na	256
YIL097W	FYV10	3	unassigned	na	55
YIL097W	FYV10	4	msa	na	89
YIL098C	FMC1	1	unassigned	na	155

YIL099W	SGA1	1	unassigned	na	71
YIL099W	SGA1	2	psiblast	1ayx__	257
YIL099W	SGA1	3	psiblast	1ayx__	221
YIL100C-A	YIL100C-A	1	unassigned	na	112
YIL100W	YIL100W	1	unassigned	na	117
YIL101C	XBP1	1	fold_recogniti	1ruxA	305
YIL101C	XBP1	2	fold_recogniti	1ruxA	342
YIL102C	YIL102C	1	unassigned	na	101
YIL103W	YIL103W	1	unassigned	na	162
YIL103W	YIL103W	2	fold_recogniti	1jzxK	208
YIL103W	YIL103W	3	fold_recogniti	1jzxK	55
YIL104C	SHQ1	1	unassigned	na	273
YIL104C	SHQ1	2	msa	na	234
YIL105C	LIT2	1	unassigned	na	76
YIL105C	LIT2	2	fold_recogniti	1itwA	178
YIL105C	LIT2	3	fold_recogniti	1itwA	155
YIL105C	LIT2	4	fold_recogniti	1i5pA	58
YIL105C	LIT2	5	psiblast	1fhwA_	125
YIL105C	LIT2	6	msa	na	94
YIL106W	MOB1	1	unassigned	na	91
YIL106W	MOB1	2	pfam	PF03637	223
YIL107C	PFK26	1	msa	na	91
YIL107C	PFK26	2	psiblast	1bif__	303
YIL107C	PFK26	3	psiblast	1bif__	267
YIL107C	PFK26	4	unassigned	na	166
YIL108W	YIL108W	1	unassigned	na	204
YIL108W	YIL108W	2	msa	na	492
YIL109C	SEC24	1	fold_recogniti	1ldvA	279
YIL109C	SEC24	2	unassigned	na	60
YIL109C	SEC24	3	msa	na	352
YIL109C	SEC24	4	unassigned	na	87
YIL109C	SEC24	5	psiblast	1svq__	66
YIL109C	SEC24	6	fold_recogniti	1m2vB	82
YIL110W	YIL110W	1	unassigned	na	120
YIL110W	YIL110W	2	psiblast	1f3lA_	112
YIL110W	YIL110W	3	unassigned	na	145
YIL111W	COX5B	1	psiblast	1occD_	151
YIL112W	HOS4	1	msa	na	202
YIL112W	HOS4	2	psiblast	1ihbA_	99
YIL112W	HOS4	3	psiblast	1ihbA_	87
YIL112W	HOS4	4	psiblast	1awcB_	93
YIL112W	HOS4	5	msa	na	76
YIL112W	HOS4	6	psiblast	1myo__	68
YIL112W	HOS4	7	psiblast	1myo__	53
YIL112W	HOS4	8	fold_recogniti	1ldvA	261
YIL112W	HOS4	9	unassigned	na	144
YIL113W	SDP1	1	psiblast	1mkp__	209
YIL114C	POR2	1	pfam	PF01459	281

YIL115C	NUP159	1	unassigned	na	132
YIL115C	NUP159	2	msa	na	477
YIL115C	NUP159	3	msa	na	326
YIL115C	NUP159	4	msa	na	441
YIL115C	NUP159	5	unassigned	na	84
YIL116W	HIS5	1	psiblast	1gewA_	286
YIL116W	HIS5	2	psiblast	1gewA_	99
YIL117C	PRM5	1	unassigned	na	318
YIL118W	RHO3	1	psiblast	1lb1B_	231
YIL119C	RPI1	1	unassigned	na	407
YIL120W	QDR1	1	msa	na	68
YIL120W	QDR1	2	psiblast	1lviA_	410
YIL120W	QDR1	3	unassigned	na	85
YIL121W	QDR2	1	psiblast	1lviA_	469
YIL121W	QDR2	2	unassigned	na	73
YIL122W	POG1	1	unassigned	na	351
YIL123W	SIM1	1	fold_recogniti	1m2vB	199
YIL123W	SIM1	2	psiblast	1deqA_	140
YIL123W	SIM1	3	pfam	PF03856	136
YIL124W	AYR1	1	psiblast	1ae1A_	297
YIL125W	KGD1	1	msa	na	148
YIL125W	KGD1	2	msa	na	127
YIL125W	KGD1	3	psiblast	1gpuA_	267
YIL125W	KGD1	4	psiblast	1gpuA_	56
YIL125W	KGD1	5	psiblast	1gpuA_	104
YIL125W	KGD1	6	psiblast	1ik6A_	179
YIL125W	KGD1	7	psiblast	1ik6A_	133
YIL126W	STH1	1	unassigned	na	233
YIL126W	STH1	2	psiblast	1gm5A_	109
YIL126W	STH1	3	psiblast	1gm5A_	307
YIL126W	STH1	4	unassigned	na	58
YIL126W	STH1	5	psiblast	1hv8A_	212
YIL126W	STH1	6	msa	na	227
YIL126W	STH1	7	unassigned	na	81
YIL126W	STH1	8	psiblast	1e6iA_	132
YIL127C	YIL127C	1	unassigned	na	206
YIL128W	MET18	1	msa	na	207
YIL128W	MET18	2	unassigned	na	251
YIL128W	MET18	3	msa	na	391
YIL128W	MET18	4	unassigned	na	183
YIL129C	TAO3	1	unassigned	na	202
YIL129C	TAO3	2	msa	na	133
YIL129C	TAO3	3	msa	na	818
YIL129C	TAO3	4	unassigned	na	304
YIL129C	TAO3	5	msa	na	538
YIL129C	TAO3	6	msa	na	228
YIL129C	TAO3	7	unassigned	na	153
YIL130W	YIL130W	1	psiblast	1d66A_	95

YIL130W	YIL130W	2	unassigned	na	97
YIL130W	YIL130W	3	msa	na	243
YIL130W	YIL130W	4	unassigned	na	347
YIL130W	YIL130W	5	msa	na	182
YIL131C	FKH1	1	unassigned	na	74
YIL131C	FKH1	2	pfam	PF00498	115
YIL131C	FKH1	3	msa	na	105
YIL131C	FKH1	4	psiblast	1d5vA_	120
YIL131C	FKH1	5	unassigned	na	70
YIL132C	CSM2	1	unassigned	na	213
YIL133C	RPL16A	1	psiblast	1k5yM_	199
YIL134W	FLX1	1	pfam	PF00153	115
YIL134W	FLX1	2	pfam	PF00153	105
YIL134W	FLX1	3	pfam	PF00153	91
YIL135C	VHS2	1	msa	na	281
YIL135C	VHS2	2	unassigned	na	155
YIL136W	OM45	1	unassigned	na	393
YIL137C	YIL137C	1	psiblast	1hs6A_	227
YIL137C	YIL137C	2	psiblast	1hs6A_	101
YIL137C	YIL137C	3	psiblast	1hs6A_	194
YIL137C	YIL137C	4	unassigned	na	59
YIL137C	YIL137C	5	msa	na	258
YIL137C	YIL137C	6	unassigned	na	107
YIL138C	TPM2	1	psiblast	2tmaA_	91
YIL138C	TPM2	2	psiblast	2tmaA_	70
YIL139C	REV7	1	pfam	PF02301	245
YIL140W	AXL2	1	psiblast	1cwvA_	148
YIL140W	AXL2	2	psiblast	1f00I_	94
YIL140W	AXL2	3	psiblast	1f00I_	84
YIL140W	AXL2	4	psiblast	1f00I_	116
YIL140W	AXL2	5	unassigned	na	381
YIL141W	YIL141W	1	unassigned	na	129
YIL142W	CCT2	1	psiblast	1a6dA_	411
YIL142W	CCT2	2	psiblast	1a6dA_	116
YIL143C	SSL2	1	unassigned	na	297
YIL143C	SSL2	2	psiblast	1hv8A_	273
YIL143C	SSL2	3	psiblast	1hv8A_	147
YIL143C	SSL2	4	psiblast	1d2mA_	126
YIL144W	TID3	1	pfam	PF03801	181
YIL144W	TID3	2	psiblast	1i84S_	317
YIL144W	TID3	3	psiblast	2tmaA_	87
YIL144W	TID3	4	msa	na	106
YIL145C	PAN6	1	psiblast	1ihoA_	222
YIL145C	PAN6	2	psiblast	1ihoA_	123
YIL146C	ECM37	1	fold_recogniti	1llwA	361
YIL146C	ECM37	2	unassigned	na	168
YIL147C	SLN1	1	msa	na	161
YIL147C	SLN1	2	unassigned	na	103

YIL147C	SLN1	3	msa	na	289
YIL147C	SLN1	4	fold_recogniti	1joyA	117
YIL147C	SLN1	5	msa	na	115
YIL147C	SLN1	6	psiblast	1bxdA_	154
YIL147C	SLN1	7	psiblast	1qkkA_	146
YIL147C	SLN1	8	psiblast	1udrA_	135
YIL148W	RPL40A	1	psiblast	1aarA_	76
YIL148W	RPL40A	2	pfam	PF01020	52
YIL149C	MLP2	1	msa	na	175
YIL149C	MLP2	2	psiblast	1c1gA_	93
YIL149C	MLP2	3	psiblast	1i84S_	424
YIL149C	MLP2	4	unassigned	na	53
YIL149C	MLP2	5	msa	na	347
YIL149C	MLP2	6	unassigned	na	77
YIL149C	MLP2	7	msa	na	434
YIL149C	MLP2	8	unassigned	na	76
YIL150C	MCM10	1	msa	na	364
YIL150C	MCM10	2	unassigned	na	95
YIL150C	MCM10	3	msa	na	112
YIL151C	YIL151C	1	unassigned	na	943
YIL151C	YIL151C	2	msa	na	175
YIL152W	YIL152W	1	unassigned	na	235
YIL153W	RRD1	1	pfam	PF03095	337
YIL153W	RRD1	2	unassigned	na	56
YIL154C	IMP2'	1	unassigned	na	181
YIL154C	IMP2'	2	fold_recogniti	1jmvA	165
YIL155C	GUT2	1	psiblast	1el5A_	497
YIL155C	GUT2	2	unassigned	na	73
YIL155C	GUT2	3	msa	na	79
YIL156W	UBP7	1	msa	na	222
YIL156W	UBP7	2	unassigned	na	77
YIL156W	UBP7	3	pfam	PF00581	179
YIL156W	UBP7	4	unassigned	na	75
YIL156W	UBP7	5	pfam	PF00442	95
YIL156W	UBP7	6	msa	na	333
YIL156W	UBP7	7	pfam	PF00443	90
YIL156W-A	YIL156W-A	1	unassigned	na	133
YIL157C	FMP35	1	unassigned	na	197
YIL158W	YIL158W	1	msa	na	106
YIL158W	YIL158W	2	unassigned	na	98
YIL159W	BNR1	1	msa	na	162
YIL159W	BNR1	2	fold_recogniti	1jpwA	129
YIL159W	BNR1	3	fold_recogniti	1jpwA	102
YIL159W	BNR1	4	fold_recogniti	1jpwA	129
YIL159W	BNR1	5	fold_recogniti	1jpwA	81
YIL159W	BNR1	6	psiblast	1dynA_	62
YIL159W	BNR1	7	fold_recogniti	1deqA	202
YIL159W	BNR1	8	unassigned	na	84

YIL159W	BNR1	9	fold_recogniti	1l9zH	102
YIL159W	BNR1	10	fold_recogniti	1l9zH	147
YIL159W	BNR1	11	fold_recogniti	1l9zH	123
YIL159W	BNR1	12	unassigned	na	52
YIL160C	POT1	1	psiblast	1afwA_	417
YIL161W	YIL161W	1	unassigned	na	235
YIL162W	SUC2	1	unassigned	na	153
YIL162W	SUC2	2	fold_recogniti	1gyhA	338
YIL162W	SUC2	3	msa	na	169
YIL163C	YIL163C	1	unassigned	na	117
YIL164C	NIT1	1	psiblast	1f89A_	199
YIL165C	YIL165C	1	psiblast	1emsA_	119
YIL166C	YIL166C	1	unassigned	na	62
YIL166C	YIL166C	2	psiblast	1ja5A_	405
YIL166C	YIL166C	3	psiblast	1ja5A_	75
YIL167W	YIL167W	1	psiblast	1tdj__	210
YIL168W	SDL1	1	psiblast	1tdj__	127
YIL169C	YIL169C	1	unassigned	na	114
YIL169C	YIL169C	2	fold_recogniti	1qu7A	164
YIL169C	YIL169C	3	unassigned	na	66
YIL169C	YIL169C	4	fold_recogniti	1deqA	121
YIL169C	YIL169C	5	fold_recogniti	1ldvA	272
YIL169C	YIL169C	6	msa	na	258
YIL170W	HXT12	1	psiblast	1ja5A_	360
YIL170W	HXT12	2	psiblast	1ja5A_	97
YIL171W	HXT12	1	unassigned	na	109
YIL171W-A	YIL171W-A	1	unassigned	na	150
YIL172C	YIL172C	1	psiblast	1uok__	302
YIL172C	YIL172C	2	psiblast	1uok__	287
YIL173W	VTH1	1	msa	na	182
YIL173W	VTH1	2	unassigned	na	354
YIL173W	VTH1	3	msa	na	186
YIL173W	VTH1	4	msa	na	676
YIL173W	VTH1	5	unassigned	na	151
YIL174W	YIL174W	1	unassigned	na	75
YIL176C	YIL176C	1	pfam	PF00660	120
YIL177C	YIL177C	1	msa	na	238
YIL177C	YIL177C	2	msa	na	211
YIL177C	YIL177C	3	unassigned	na	108
YIL177C	YIL177C	4	psiblast	1fuuA_	546
YIL177C	YIL177C	5	fold_recogniti	1m2vB	361
YIL177C	YIL177C	6	unassigned	na	294
YIR001C	SGN1	1	psiblast	1ha1__	142
YIR001C	SGN1	2	psiblast	1ha1__	108
YIR002C	MPH1	1	psiblast	1qdeA_	286
YIR002C	MPH1	2	psiblast	1hv8A_	200
YIR002C	MPH1	3	psiblast	1hv8A_	148
YIR002C	MPH1	4	psiblast	1d9xA_	103

YIR002C	MPH1	5	unassigned	na	256
YIR003W	YIR003W	1	fold_recogniti	1l8mA	96
YIR003W	YIR003W	2	fold_recogniti	1l8mA	73
YIR003W	YIR003W	3	fold_recogniti	1l8mA	79
YIR003W	YIR003W	4	fold_recogniti	1l8mA	72
YIR003W	YIR003W	5	fold_recogniti	1jhnA	183
YIR003W	YIR003W	6	fold_recogniti	1m2vB	88
YIR003W	YIR003W	7	unassigned	na	88
YIR004W	DJP1	1	psiblast	1xbl__	117
YIR004W	DJP1	2	psiblast	1exkA_	80
YIR004W	DJP1	3	psiblast	1c3gA_	128
YIR004W	DJP1	4	msa	na	107
YIR005W	IST3	1	psiblast	1ha1__	148
YIR006C	PAN1	1	fold_recogniti	1deqA	235
YIR006C	PAN1	2	psiblast	1fi6A_	119
YIR006C	PAN1	3	fold_recogniti	1k83A	238
YIR006C	PAN1	4	psiblast	1c07A_	100
YIR006C	PAN1	5	fold_recogniti	1jv2B	326
YIR006C	PAN1	6	fold_recogniti	1jv2B	151
YIR006C	PAN1	7	fold_recogniti	1l8mA	85
YIR006C	PAN1	8	fold_recogniti	1l8mA	73
YIR006C	PAN1	9	fold_recogniti	1l8mA	80
YIR006C	PAN1	10	fold_recogniti	1l8mA	73
YIR007W	YIR007W	1	unassigned	na	69
YIR007W	YIR007W	2	psiblast	1edg__	98
YIR007W	YIR007W	3	unassigned	na	597
YIR008C	PRI1	1	psiblast	1g71A_	98
YIR008C	PRI1	2	psiblast	1g71A_	197
YIR008C	PRI1	3	psiblast	1g71A_	114
YIR009W	MSL1	1	psiblast	1nrcA_	111
YIR010W	DSN1	1	unassigned	na	77
YIR010W	DSN1	2	psiblast	1mmg__	95
YIR010W	DSN1	3	psiblast	1i84S_	178
YIR010W	DSN1	4	psiblast	1i84S_	226
YIR011C	STS1	1	unassigned	na	319
YIR012W	SQT1	1	psiblast	1gxrA_	97
YIR012W	SQT1	2	psiblast	1gg2B_	334
YIR013C	GAT4	1	psiblast	2gatA_	121
YIR014W	YIR014W	1	unassigned	na	276
YIR015W	RPR2	1	unassigned	na	144
YIR016W	YIR016W	1	unassigned	na	265
YIR017C	MET28	1	msa	na	93
YIR017C	MET28	2	psiblast	1hjbA_	94
YIR017W-A	YIR017W-A	1	unassigned	na	199
YIR018W	YAP5	1	psiblast	1hjbA_	193
YIR018W	YAP5	2	unassigned	na	52
YIR019C	MUC1	1	msa	na	407
YIR019C	MUC1	2	msa	na	648

YIR019C	MUC1	3	msa	na	312
YIR020C	YIR020C	1	unassigned	na	100
YIR020C-B	YIR020C-B	1	unassigned	na	244
YIR020W-A	YIR020W-A	1	unassigned	na	80
YIR021W	MRS1	1	unassigned	na	69
YIR021W	MRS1	2	psiblast	1kcfA_	149
YIR021W	MRS1	3	unassigned	na	145
YIR022W	SEC11	1	psiblast	1b12A_	115
YIR022W	SEC11	2	unassigned	na	52
YIR023C-A	YIR023C-A	1	unassigned	na	107
YIR023W	DAL81	1	msa	na	123
YIR023W	DAL81	2	psiblast	1pyiA_	172
YIR023W	DAL81	3	msa	na	193
YIR023W	DAL81	4	unassigned	na	256
YIR023W	DAL81	5	fold_recogniti	1ldvA	226
YIR024C	YIR024C	1	unassigned	na	216
YIR025W	MND2	1	unassigned	na	368
YIR026C	YVH1	1	psiblast	1mkp__	70
YIR026C	YVH1	2	psiblast	1larA_	180
YIR026C	YVH1	3	unassigned	na	114
YIR027C	DAL1	1	psiblast	1gkrA_	460
YIR028W	DAL4	1	unassigned	na	97
YIR028W	DAL4	2	pfam	PF02133	484
YIR028W	DAL4	3	unassigned	na	54
YIR029W	DAL2	1	pfam	PF03561	185
YIR029W	DAL2	2	pfam	PF03561	158
YIR030C	DCG1	1	psiblast	1b73A_	112
YIR030C	DCG1	2	psiblast	1b73A_	132
YIR031C	DAL7	1	fold_recogniti	1d8cA	104
YIR031C	DAL7	2	psiblast	1d8cA_	317
YIR031C	DAL7	3	psiblast	1d8cA_	133
YIR032C	DAL3	1	unassigned	na	195
YIR033W	MGA2	1	unassigned	na	346
YIR033W	MGA2	2	msa	na	178
YIR033W	MGA2	3	psiblast	1k1aA_	158
YIR033W	MGA2	4	psiblast	1k1aA_	119
YIR033W	MGA2	5	psiblast	1lnnA_	68
YIR033W	MGA2	6	unassigned	na	111
YIR033W	MGA2	7	msa	na	79
YIR033W	MGA2	8	unassigned	na	54
YIR034C	LYS1	1	psiblast	1pjbA_	185
YIR034C	LYS1	2	psiblast	1pjbA_	188
YIR035C	YIR035C	1	psiblast	1ae1A_	254
YIR036C	YIR036C	1	psiblast	2ae1__	263
YIR037W	HYR1	1	psiblast	1gp1A_	163
YIR038C	GTT1	1	psiblast	1byeA_	89
YIR038C	GTT1	2	psiblast	1byeA_	145
YIR039C	YPS6	1	psiblast	1ypsA_	146

YIR039C	YPS6	2	psiblast	1ypsA_	305
YIR039C	YPS6	3	unassigned	na	86
YIR040C	YIR040C	1	unassigned	na	110
YIR041W	YIR041W	1	pfam	PF00660	124
YIR042C	YIR042C	1	psiblast	1i12A_	236
YIR043C	YIR043C	1	unassigned	na	64
YIR043C	YIR043C	2	pfam	PF00674	166
YIR044C	YIR044C	1	unassigned	na	61
YJL001W	PRE3	1	psiblast	1rypH_	215
YJL002C	OST1	1	msa	na	109
YJL002C	OST1	2	unassigned	na	184
YJL002C	OST1	3	msa	na	183
YJL003W	COX16	1	unassigned	na	118
YJL004C	SYS1	1	msa	na	203
YJL005W	CYR1	1	fold_recogniti	1k83A	409
YJL005W	CYR1	2	fold_recogniti	1ldvA	144
YJL005W	CYR1	3	psiblast	2bnh__	106
YJL005W	CYR1	4	psiblast	2bnh__	60
YJL005W	CYR1	5	psiblast	2bnh__	58
YJL005W	CYR1	6	psiblast	2bnh__	170
YJL005W	CYR1	7	psiblast	1k5dC_	83
YJL005W	CYR1	8	psiblast	1jl5A_	93
YJL005W	CYR1	9	unassigned	na	62
YJL005W	CYR1	10	psiblast	1kooA_	115
YJL005W	CYR1	11	unassigned	na	52
YJL005W	CYR1	12	psiblast	1a6q__	297
YJL005W	CYR1	13	psiblast	1fx4A_	253
YJL005W	CYR1	14	unassigned	na	124
YJL006C	CTK2	1	psiblast	1vin__	194
YJL006C	CTK2	2	psiblast	1vin__	129
YJL007C	YJL007C	1	unassigned	na	104
YJL008C	CCT8	1	psiblast	1a6dA_	442
YJL008C	CCT8	2	psiblast	1a6dA_	126
YJL009W	YJL009W	1	unassigned	na	108
YJL010C	YJL010C	1	unassigned	na	77
YJL010C	YJL010C	2	pfam	PF00806	49
YJL010C	YJL010C	3	pfam	PF00806	43
YJL010C	YJL010C	4	psiblast	1ib2A_	166
YJL010C	YJL010C	5	psiblast	1ib2A_	102
YJL010C	YJL010C	6	psiblast	1ib2A_	156
YJL010C	YJL010C	7	unassigned	na	73
YJL011C	RPC17	1	unassigned	na	161
YJL012C	VTC4	1	pfam	PF03105	194
YJL012C	VTC4	2	msa	na	304
YJL012C	VTC4	3	unassigned	na	150
YJL012C-A	3171	1	unassigned	na	65
YJL013C	MAD3	1	fold_recogniti	1fchA	265
YJL013C	MAD3	2	unassigned	na	124

YJL013C	MAD3	3	msa	na	126
YJL014W	CCT3	1	psiblast	1a6dB_	534
YJL015C	YJL015C	1	unassigned	na	124
YJL016W	YJL016W	1	unassigned	na	171
YJL017W	3176	1	unassigned	na	325
YJL018W	3177	1	unassigned	na	104
YJL019W	MPS3	1	unassigned	na	110
YJL019W	MPS3	2	fold_recogniti	1nf1A	315
YJL019W	MPS3	3	unassigned	na	195
YJL020C	BBC1	1	fold_recogniti	1k83A	369
YJL020C	BBC1	2	psiblast	1i84S_	299
YJL020C	BBC1	3	fold_recogniti	1ldvA	103
YJL021C	3180	1	unassigned	na	365
YJL022W	YJL022W	1	unassigned	na	102
YJL023C	PET130	1	unassigned	na	347
YJL024C	APS3	1	psiblast	1gw5S_	103
YJL024C	APS3	2	psiblast	1gw5S_	91
YJL025W	RRN7	1	unassigned	na	514
YJL026W	RNR2	1	psiblast	1jk0A_	399
YJL027C	YJL027C	1	unassigned	na	138
YJL028W	YJL028W	1	unassigned	na	111
YJL029C	VPS53	1	fold_recogniti	1n1bA	332
YJL029C	VPS53	2	fold_recogniti	1n1bA	58
YJL029C	VPS53	3	fold_recogniti	1n1bA	130
YJL029C	VPS53	4	unassigned	na	302
YJL030W	MAD2	1	psiblast	1klqA_	196
YJL031C	BET4	1	psiblast	1dceA_	125
YJL031C	BET4	2	psiblast	1dceA_	74
YJL031C	BET4	3	psiblast	1ihgA_	91
YJL032W	YJL032W	1	unassigned	na	104
YJL033W	HCA4	1	psiblast	1hv8A_	261
YJL033W	HCA4	2	psiblast	1hv8A_	150
YJL033W	HCA4	3	psiblast	1d2mA_	118
YJL033W	HCA4	4	msa	na	241
YJL034W	KAR2	1	psiblast	1hpm__	237
YJL034W	KAR2	2	psiblast	1hpm__	116
YJL034W	KAR2	3	psiblast	1hpm__	75
YJL034W	KAR2	4	psiblast	1ckrA_	158
YJL034W	KAR2	5	psiblast	1i84S_	96
YJL035C	TAD2	1	fold_recogniti	1jtkA	142
YJL035C	TAD2	2	msa	na	108
YJL036W	SNX4	1	psiblast	1h6hA_	68
YJL036W	SNX4	2	psiblast	1i84S_	355
YJL037W	YJL037W	1	unassigned	na	224
YJL038C	YJL038C	1	unassigned	na	219
YJL039C	NUP192	1	unassigned	na	686
YJL039C	NUP192	2	msa	na	518
YJL039C	NUP192	3	unassigned	na	479

YJL041W	NSP1	1	fold_recogniti	1deqA	68
YJL041W	NSP1	2	fold_recogniti	1jv2B	293
YJL041W	NSP1	3	fold_recogniti	1jv2B	164
YJL041W	NSP1	4	fold_recogniti	1jv2B	103
YJL041W	NSP1	5	msa	na	195
YJL042W	MHP1	1	msa	na	677
YJL042W	MHP1	2	msa	na	321
YJL042W	MHP1	3	unassigned	na	400
YJL043W	YJL043W	1	msa	na	139
YJL043W	YJL043W	2	unassigned	na	118
YJL044C	GYP6	1	unassigned	na	82
YJL044C	GYP6	2	psiblast	1fkmA_	198
YJL044C	GYP6	3	psiblast	1fkmA_	68
YJL044C	GYP6	4	unassigned	na	110
YJL045W	YJL045W	1	psiblast	1kf6A_	349
YJL045W	YJL045W	2	psiblast	1kf6A_	285
YJL046W	YJL046W	1	unassigned	na	110
YJL046W	YJL046W	2	msa	na	118
YJL046W	YJL046W	3	fold_recogniti	1hxdA	223
YJL047C	RTT101	1	psiblast	1ldjA_	383
YJL047C	RTT101	2	psiblast	1ldjA_	168
YJL047C	RTT101	3	psiblast	1ldjA_	98
YJL047C	RTT101	4	psiblast	1ldjA_	124
YJL047C	RTT101	5	psiblast	1ldjA_	69
YJL048C	UBX6	1	unassigned	na	67
YJL048C	UBX6	2	msa	na	117
YJL048C	UBX6	3	psiblast	1h8cA_	118
YJL048C	UBX6	4	unassigned	na	94
YJL049W	YJL049W	1	unassigned	na	84
YJL049W	YJL049W	2	psiblast	1i84S_	366
YJL050W	MTR4	1	unassigned	na	109
YJL050W	MTR4	2	psiblast	1hv8A_	212
YJL050W	MTR4	3	psiblast	1hv8A_	134
YJL050W	MTR4	4	psiblast	1fukA_	181
YJL050W	MTR4	5	unassigned	na	116
YJL050W	MTR4	6	fold_recogniti	1n1bA	228
YJL050W	MTR4	7	fold_recogniti	1n1bA	93
YJL051W	YJL051W	1	unassigned	na	154
YJL051W	YJL051W	2	msa	na	537
YJL051W	YJL051W	3	msa	na	131
YJL052C-A	YJL052C-A	1	unassigned	na	39
YJL052W	TDH1	1	psiblast	3gpdR_	161
YJL052W	TDH1	2	psiblast	3gpdR_	171
YJL053W	PEP8	1	fold_recogniti	1cf1A	188
YJL053W	PEP8	2	fold_recogniti	1cf1A	191
YJL054W	TIM54	1	msa	na	166
YJL054W	TIM54	2	unassigned	na	173
YJL054W	TIM54	3	msa	na	139

YJL055W	YJL055W	1	pfam	PF03641	245
YJL056C	ZAP1	1	msa	na	118
YJL056C	ZAP1	2	msa	na	168
YJL056C	ZAP1	3	unassigned	na	122
YJL056C	ZAP1	4	msa	na	151
YJL056C	ZAP1	5	msa	na	131
YJL056C	ZAP1	6	psiblast	2gliA_	97
YJL056C	ZAP1	7	psiblast	2gliA_	93
YJL057C	YJL057C	1	unassigned	na	140
YJL057C	YJL057C	2	psiblast	1a06__	139
YJL057C	YJL057C	3	psiblast	1a06__	291
YJL057C	YJL057C	4	unassigned	na	97
YJL058C	BIT61	1	unassigned	na	543
YJL059W	YHC3	1	pfam	PF02487	408
YJL060W	BNA3	1	psiblast	1gd9A_	309
YJL060W	BNA3	2	psiblast	1gd9A_	135
YJL061W	NUP82	1	msa	na	200
YJL061W	NUP82	2	unassigned	na	274
YJL061W	NUP82	3	msa	na	239
YJL062W	LAS21	1	psiblast	1e3cP_	378
YJL062W	LAS21	2	msa	na	130
YJL062W	LAS21	3	unassigned	na	196
YJL062W	LAS21	4	msa	na	126
YJL062W-A	YJL062W-A	1	unassigned	na	85
YJL063C	MRPL8	1	psiblast	1gd8A_	161
YJL063C	MRPL8	2	unassigned	na	77
YJL064W	YJL064W	1	unassigned	na	131
YJL065C	DLS1	1	psiblast	1jfiA_	110
YJL065C	DLS1	2	unassigned	na	57
YJL066C	MPM1	1	unassigned	na	252
YJL067W	YJL067W	1	unassigned	na	116
YJL068C	YJL068C	1	psiblast	1f0nA_	299
YJL069C	UTP18	1	unassigned	na	90
YJL069C	UTP18	2	msa	na	141
YJL069C	UTP18	3	psiblast	1gg2B_	363
YJL070C	YJL070C	1	unassigned	na	426
YJL070C	YJL070C	2	fold_recogniti	1m7mA	462
YJL071W	ARG2	1	unassigned	na	400
YJL071W	ARG2	2	msa	na	174
YJL072C	PSF2	1	unassigned	na	115
YJL072C	PSF2	2	msa	na	98
YJL073W	JEM1	1	unassigned	na	111
YJL073W	JEM1	2	psiblast	1a17__	199
YJL073W	JEM1	3	unassigned	na	59
YJL073W	JEM1	4	msa	na	162
YJL073W	JEM1	5	psiblast	1xbl__	114
YJL074C	SMC3	1	psiblast	1br2A_	50
YJL074C	SMC3	2	psiblast	1i84S_	344

YJL074C	SMC3	3	unassigned	na	57
YJL074C	SMC3	4	psiblast	1gxlA_	122
YJL074C	SMC3	5	psiblast	1gxlA_	46
YJL074C	SMC3	6	psiblast	1gxlA_	44
YJL074C	SMC3	7	psiblast	1c1gA_	67
YJL074C	SMC3	8	psiblast	1c1gA_	262
YJL074C	SMC3	9	unassigned	na	53
YJL074C	SMC3	10	psiblast	1e69A_	185
YJL075C	APQ13	1	unassigned	na	138
YJL076W	NET1	1	msa	na	226
YJL076W	NET1	2	unassigned	na	70
YJL076W	NET1	3	psiblast	1i84S_	361
YJL076W	NET1	4	fold_recogniti	1l8mA	106
YJL076W	NET1	5	fold_recogniti	1l8mA	73
YJL076W	NET1	6	fold_recogniti	1l8mA	79
YJL076W	NET1	7	fold_recogniti	1l8mA	70
YJL076W	NET1	8	fold_recogniti	1deqA	204
YJL077C	ICS3	1	unassigned	na	131
YJL078C	PRY3	1	psiblast	1cfe__	168
YJL078C	PRY3	2	fold_recogniti	1m2vB	129
YJL078C	PRY3	3	fold_recogniti	1jv2B	323
YJL078C	PRY3	4	fold_recogniti	1jv2B	103
YJL078C	PRY3	5	fold_recogniti	1ei3A	74
YJL078C	PRY3	6	unassigned	na	84
YJL079C	PRY1	1	fold_recogniti	1ldvA	159
YJL079C	PRY1	2	psiblast	1cfe__	140
YJL080C	SCP160	1	msa	na	171
YJL080C	SCP160	2	psiblast	1k1gA_	95
YJL080C	SCP160	3	unassigned	na	73
YJL080C	SCP160	4	psiblast	1hh2P_	78
YJL080C	SCP160	5	psiblast	1e3pA_	66
YJL080C	SCP160	6	psiblast	1e3pA_	84
YJL080C	SCP160	7	psiblast	1e3pA_	282
YJL080C	SCP160	8	fold_recogniti	1khmA	92
YJL080C	SCP160	9	psiblast	1vig__	69
YJL080C	SCP160	10	psiblast	1sro__	72
YJL080C	SCP160	11	psiblast	1j4wA_	66
YJL080C	SCP160	12	psiblast	1j4wA_	74
YJL081C	ARP4	1	psiblast	1dgaA_	180
YJL081C	ARP4	2	psiblast	1dgaA_	309
YJL082W	IML2	1	msa	na	215
YJL082W	IML2	2	unassigned	na	171
YJL082W	IML2	3	fold_recogniti	1fchA	157
YJL082W	IML2	4	fold_recogniti	1fchA	92
YJL082W	IML2	5	fold_recogniti	1fchA	96
YJL083W	YJL083W	1	unassigned	na	433
YJL083W	YJL083W	2	psiblast	1c07A_	171
YJL084C	YJL084C	1	msa	na	241

YJL084C	YJL084C	2	psiblast	1jsyA_	192
YJL084C	YJL084C	3	msa	na	613
YJL085W	EXO70	1	fold_recogniti	1hciA	216
YJL085W	EXO70	2	msa	na	317
YJL085W	EXO70	3	unassigned	na	90
YJL086C	YJL086C	1	unassigned	na	122
YJL087C	TRL1	1	msa	na	307
YJL087C	TRL1	2	unassigned	na	520
YJL088W	ARG3	1	unassigned	na	103
YJL088W	ARG3	2	psiblast	1c9yA_	174
YJL088W	ARG3	3	psiblast	1c9yA_	163
YJL089W	SIP4	1	psiblast	1pyiA_	176
YJL089W	SIP4	2	unassigned	na	653
YJL090C	DPB11	1	fold_recogniti	1l0bA	124
YJL090C	DPB11	2	fold_recogniti	1l0bA	139
YJL090C	DPB11	3	unassigned	na	57
YJL090C	DPB11	4	pfam	PF00533	109
YJL090C	DPB11	5	unassigned	na	335
YJL091C	GWT1	1	msa	na	350
YJL091C	GWT1	2	unassigned	na	148
YJL092W	HPR5	1	psiblast	1pjr__	313
YJL092W	HPR5	2	psiblast	1pjr__	526
YJL092W	HPR5	3	fold_recogniti	1ei3A	335
YJL093C	TOK1	1	msa	na	117
YJL093C	TOK1	2	msa	na	77
YJL093C	TOK1	3	fold_recogniti	1f6gA	156
YJL093C	TOK1	4	psiblast	1lnqA_	108
YJL093C	TOK1	5	psiblast	1lnqA_	180
YJL093C	TOK1	6	unassigned	na	53
YJL094C	KHA1	1	pfam	PF00999	451
YJL094C	KHA1	2	unassigned	na	100
YJL094C	KHA1	3	msa	na	105
YJL094C	KHA1	4	unassigned	na	217
YJL095W	BCK1	1	unassigned	na	653
YJL095W	BCK1	2	fold_recogniti	1ldvA	296
YJL095W	BCK1	3	psiblast	1f3mA_	83
YJL095W	BCK1	4	unassigned	na	97
YJL095W	BCK1	5	psiblast	1f3mC_	130
YJL095W	BCK1	6	psiblast	1f3mC_	219
YJL096W	MRPL49	1	unassigned	na	113
YJL096W	MRPL49	2	psiblast	1lnrP_	111
YJL097W	YJL097W	1	msa	na	129
YJL097W	YJL097W	2	unassigned	na	88
YJL098W	SAP185	1	unassigned	na	124
YJL098W	SAP185	2	msa	na	572
YJL098W	SAP185	3	unassigned	na	53
YJL098W	SAP185	4	msa	na	309
YJL099W	CHS6	1	unassigned	na	199

YJL099W	CHS6	2	msa	na	288
YJL099W	CHS6	3	msa	na	259
YJL100W	LSB6	1	unassigned	na	107
YJL100W	LSB6	2	msa	na	294
YJL100W	LSB6	3	msa	na	154
YJL100W	LSB6	4	unassigned	na	52
YJL101C	GSH1	1	msa	na	237
YJL101C	GSH1	2	pfam	PF03074	441
YJL102W	MEF2	1	psiblast	1dar__	330
YJL102W	MEF2	2	psiblast	1dar__	171
YJL102W	MEF2	3	psiblast	1dar__	273
YJL102W	MEF2	4	fold_recogniti	1efgA	45
YJL103C	YJL103C	1	fold_recogniti	1pyiA	110
YJL103C	YJL103C	2	unassigned	na	53
YJL103C	YJL103C	3	msa	na	213
YJL103C	YJL103C	4	msa	na	242
YJL104W	PAM16	1	fold_recogniti	1gh6A	149
YJL105W	SET4	1	msa	na	95
YJL105W	SET4	2	psiblast	1fp0A_	150
YJL105W	SET4	3	msa	na	315
YJL106W	IME2	1	psiblast	1aq1__	148
YJL106W	IME2	2	psiblast	1aq1__	141
YJL106W	IME2	3	psiblast	1aq1__	51
YJL106W	IME2	4	psiblast	1aq1__	91
YJL106W	IME2	5	unassigned	na	214
YJL107C	YJL107C	1	unassigned	na	109
YJL107C	YJL107C	2	msa	na	193
YJL107C	YJL107C	3	msa	na	85
YJL108C	PRM10	1	msa	na	212
YJL108C	PRM10	2	unassigned	na	171
YJL109C	UTP10	1	fold_recogniti	1b3uA	186
YJL109C	UTP10	2	fold_recogniti	1b3uA	67
YJL109C	UTP10	3	fold_recogniti	1b3uA	148
YJL109C	UTP10	4	fold_recogniti	1b3uA	205
YJL109C	UTP10	5	unassigned	na	57
YJL109C	UTP10	6	msa	na	155
YJL109C	UTP10	7	msa	na	951
YJL110C	GZF3	1	unassigned	na	123
YJL110C	GZF3	2	psiblast	4gatA_	69
YJL110C	GZF3	3	msa	na	286
YJL110C	GZF3	4	msa	na	73
YJL111W	CCT7	1	psiblast	1a6dA_	435
YJL111W	CCT7	2	psiblast	1a6dA_	115
YJL112W	MDV1	1	psiblast	1k32A_	516
YJL112W	MDV1	2	psiblast	1gg2B_	198
YJL115W	ASF1	1	unassigned	na	83
YJL115W	ASF1	2	fold_recogniti	1l8mA	98
YJL115W	ASF1	3	fold_recogniti	1l8mA	98

YJL116C	NCA3	1	pfam	PF03856	337
YJL117W	PHO86	1	unassigned	na	311
YJL118W	YJL118W	1	unassigned	na	219
YJL119C	YJL119C	1	unassigned	na	107
YJL120W	YJL120W	1	unassigned	na	107
YJL121C	RPE1	1	psiblast	1rpxA_	238
YJL122W	YJL122W	1	unassigned	na	175
YJL123C	YJL123C	1	fold_recogniti	1ldvA	267
YJL123C	YJL123C	2	unassigned	na	211
YJL124C	LSM1	1	psiblast	1i8fA_	172
YJL125C	GCD14	1	psiblast	1i9gA_	78
YJL125C	GCD14	2	psiblast	1i9gA_	234
YJL125C	GCD14	3	unassigned	na	71
YJL126W	NIT2	1	psiblast	1emsA_	307
YJL127C	SPT10	1	unassigned	na	88
YJL127C	SPT10	2	psiblast	1b87A_	221
YJL127C	SPT10	3	fold_recogniti	1k6yA	88
YJL127C	SPT10	4	fold_recogniti	1k6yA	243
YJL127W-A	YJL127W-A	1	unassigned	na	38
YJL128C	PBS2	1	unassigned	na	138
YJL128C	PBS2	2	fold_recogniti	1ldvA	189
YJL128C	PBS2	3	psiblast	1f3mC_	110
YJL128C	PBS2	4	psiblast	1f3mC_	231
YJL129C	TRK1	1	unassigned	na	137
YJL129C	TRK1	2	msa	na	488
YJL129C	TRK1	3	pfam	PF02386	610
YJL130C	URA2	1	psiblast	1bxrB_	190
YJL130C	URA2	2	psiblast	1bxrB_	232
YJL130C	URA2	3	psiblast	1a9xA_	294
YJL130C	URA2	4	psiblast	1a9xA_	174
YJL130C	URA2	5	psiblast	1a9xA_	74
YJL130C	URA2	6	psiblast	1a9xA_	122
YJL130C	URA2	7	psiblast	1a9xA_	212
YJL130C	URA2	8	psiblast	1a9xA_	192
YJL130C	URA2	9	psiblast	1gkrA_	347
YJL130C	URA2	10	unassigned	na	60
YJL130C	URA2	11	psiblast	1ekxA_	82
YJL130C	URA2	12	psiblast	1ekxA_	235
YJL131C	YJL131C	1	unassigned	na	356
YJL132W	YJL132W	1	msa	na	301
YJL132W	YJL132W	2	unassigned	na	449
YJL133W	MRS3	1	fold_recogniti	1is2A	314
YJL134W	LCB3	1	unassigned	na	112
YJL134W	LCB3	2	psiblast	1d2tA_	156
YJL134W	LCB3	3	unassigned	na	141
YJL135W	YJL135W	1	unassigned	na	105
YJL136C	RPS21B	1	pfam	PF01249	87
YJL136W-A	YJL136W-A	1	unassigned	na	27

YJL137C	GLG2	1	psiblast	1ll0A_	298
YJL137C	GLG2	2	unassigned	na	82
YJL138C	TIF2	1	psiblast	1fuuA_	395
YJL139C	YUR1	1	pfam	PF01793	428
YJL140W	RPB4	1	pfam	PF03874	122
YJL140W	RPB4	2	fold_recogniti	1go3F	99
YJL141C	YAK1	1	unassigned	na	339
YJL141C	YAK1	2	psiblast	1aq1__	112
YJL141C	YAK1	3	psiblast	1aq1__	87
YJL141C	YAK1	4	psiblast	1aq1__	50
YJL141C	YAK1	5	psiblast	1aq1__	73
YJL141C	YAK1	6	fold_recogniti	1i09A	146
YJL142C	YJL142C	1	unassigned	na	130
YJL143W	TIM17	1	pfam	PF02466	158
YJL144W	YJL144W	1	unassigned	na	104
YJL145W	SFH5	1	psiblast	1aua__	232
YJL145W	SFH5	2	psiblast	1aua__	62
YJL146W	IDS2	1	unassigned	na	469
YJL147C	YJL147C	1	unassigned	na	382
YJL148W	RPA34	1	unassigned	na	233
YJL149W	YJL149W	1	pfam	PF00646	103
YJL149W	YJL149W	2	unassigned	na	560
YJL150W	YJL150W	1	unassigned	na	100
YJL151C	SNA3	1	pfam	PF01679	133
YJL152W	YJL152W	1	unassigned	na	119
YJL153C	INO1	1	psiblast	1jkfA_	555
YJL154C	VPS35	1	unassigned	na	273
YJL154C	VPS35	2	msa	na	619
YJL154C	VPS35	3	unassigned	na	52
YJL155C	FBP26	1	psiblast	1bif__	222
YJL155C	FBP26	2	psiblast	1bif__	230
YJL156C	SSY5	1	unassigned	na	687
YJL156W-A	YJL156W-A	1	unassigned	na	73
YJL157C	FAR1	1	unassigned	na	174
YJL157C	FAR1	2	msa	na	347
YJL157C	FAR1	3	unassigned	na	309
YJL158C	CIS3	1	msa	na	163
YJL158C	CIS3	2	msa	na	64
YJL159W	HSP150	1	fold_recogniti	1qqeA	150
YJL159W	HSP150	2	fold_recogniti	1qqeA	160
YJL160C	YJL160C	1	unassigned	na	180
YJL161W	FMP33	1	unassigned	na	180
YJL162C	JJJ2	1	psiblast	1xbl__	124
YJL162C	JJJ2	2	psiblast	1exkA_	106
YJL162C	JJJ2	3	msa	na	74
YJL162C	JJJ2	4	unassigned	na	178
YJL163C	YJL163C	1	unassigned	na	70
YJL163C	YJL163C	2	psiblast	1ja5A_	427

YJL163C	YJL163C	3	psiblast	1ja5A_	58
YJL164C	TPK1	1	unassigned	na	60
YJL164C	TPK1	2	psiblast	1fotA_	129
YJL164C	TPK1	3	psiblast	1fotA_	208
YJL165C	HAL5	1	unassigned	na	464
YJL165C	HAL5	2	psiblast	1a06__	161
YJL165C	HAL5	3	psiblast	1a06__	230
YJL166W	QCR8	1	psiblast	1ezvG_	94
YJL167W	ERG20	1	psiblast	1fps__	188
YJL167W	ERG20	2	psiblast	1fps__	100
YJL167W	ERG20	3	psiblast	1fps__	64
YJL168C	SET2	1	unassigned	na	89
YJL168C	SET2	2	pfam	PF00856	190
YJL168C	SET2	3	unassigned	na	454
YJL169W	YJL169W	1	unassigned	na	122
YJL170C	ASG7	1	unassigned	na	183
YJL171C	YJL171C	1	unassigned	na	73
YJL171C	YJL171C	2	msa	na	323
YJL172W	CPS1	1	unassigned	na	68
YJL172W	CPS1	2	psiblast	1cg2A_	302
YJL172W	CPS1	3	psiblast	1cg2A_	109
YJL172W	CPS1	4	fold_recogniti	1lfwA	97
YJL173C	RFA3	1	unassigned	na	122
YJL174W	KRE9	1	unassigned	na	276
YJL175W	YJL175W	1	unassigned	na	170
YJL176C	SWI3	1	fold_recogniti	1l8mA	86
YJL176C	SWI3	2	fold_recogniti	1l8mA	73
YJL176C	SWI3	3	fold_recogniti	1l8mA	79
YJL176C	SWI3	4	fold_recogniti	1l8mA	70
YJL176C	SWI3	5	unassigned	na	101
YJL176C	SWI3	6	psiblast	1h8qA_	76
YJL176C	SWI3	7	fold_recogniti	1h88C	111
YJL176C	SWI3	8	msa	na	229
YJL177W	RPL17B	1	psiblast	1k5yN_	184
YJL178C	ATG27	1	unassigned	na	196
YJL179W	PFD1	1	unassigned	na	109
YJL180C	ATP12	1	msa	na	134
YJL180C	ATP12	2	unassigned	na	79
YJL180C	ATP12	3	msa	na	112
YJL181W	YJL181W	1	msa	na	399
YJL181W	YJL181W	2	unassigned	na	212
YJL182C	YJL182C	1	unassigned	na	105
YJL183W	MNN11	1	unassigned	na	87
YJL183W	MNN11	2	msa	na	335
YJL184W	GON7	1	unassigned	na	123
YJL185C	YJL185C	1	unassigned	na	293
YJL186W	MNN5	1	unassigned	na	136
YJL186W	MNN5	2	fold_recogniti	1ga8A	166

YJL186W	MNN5	3	unassigned	na	284
YJL187C	SWE1	1	unassigned	na	400
YJL187C	SWE1	2	psiblast	1fotA_	116
YJL187C	SWE1	3	psiblast	1fotA_	232
YJL187C	SWE1	4	unassigned	na	71
YJL188C	BUD19	1	unassigned	na	102
YJL189W	RPL39	1	pfam	PF00832	51
YJL190C	RPS22A	1	psiblast	1k5xH_	79
YJL190C	RPS22A	2	psiblast	1k5xH_	51
YJL191W	RPS14B	1	psiblast	1k5xK_	138
YJL192C	SOP4	1	unassigned	na	234
YJL193W	YJL193W	1	msa	na	249
YJL193W	YJL193W	2	msa	na	153
YJL194W	CDC6	1	psiblast	1e32A_	290
YJL194W	CDC6	2	psiblast	1e32A_	68
YJL194W	CDC6	3	fold_recogniti	1fnnA	155
YJL195C	YJL195C	1	unassigned	na	233
YJL196C	ELO1	1	pfam	PF01151	310
YJL197W	UBP12	1	unassigned	na	90
YJL197W	UBP12	2	msa	na	244
YJL197W	UBP12	3	pfam	PF00442	71
YJL197W	UBP12	4	msa	na	313
YJL197W	UBP12	5	unassigned	na	193
YJL197W	UBP12	6	msa	na	124
YJL197W	UBP12	7	pfam	PF00443	136
YJL197W	UBP12	8	unassigned	na	83
YJL198W	PHO90	1	pfam	PF03105	294
YJL198W	PHO90	2	unassigned	na	95
YJL198W	PHO90	3	pfam	PF00939	492
YJL199C	MBB1	1	unassigned	na	108
YJL200C	YJL200C	1	psiblast	5acn__	584
YJL200C	YJL200C	2	psiblast	5acn__	205
YJL201W	ECM25	1	msa	na	133
YJL201W	ECM25	2	psiblast	1rgp__	126
YJL201W	ECM25	3	psiblast	1rgp__	105
YJL201W	ECM25	4	fold_recogniti	1m2vB	235
YJL202C	YJL202C	1	unassigned	na	115
YJL203W	PRP21	1	pfam	PF01805	75
YJL203W	PRP21	2	pfam	PF01805	133
YJL203W	PRP21	3	unassigned	na	72
YJL204C	RCY1	1	pfam	PF00646	88
YJL204C	RCY1	2	unassigned	na	378
YJL204C	RCY1	3	fold_recogniti	1n1bA	216
YJL204C	RCY1	4	fold_recogniti	1n1bA	158
YJL205C-A	3366	1	unassigned	na	53
YJL206C	YJL206C	1	psiblast	1ajyA_	116
YJL206C	YJL206C	2	msa	na	268
YJL206C	YJL206C	3	msa	na	285

YJL206C	YJL206C	4	unassigned	na	89
YJL207C	YJL207C	1	unassigned	na	1286
YJL207C	YJL207C	2	msa	na	187
YJL207C	YJL207C	3	msa	na	365
YJL207C	YJL207C	4	unassigned	na	176
YJL208C	NUC1	1	psiblast	1qaeA_	329
YJL209W	CBP1	1	unassigned	na	557
YJL209W	CBP1	2	msa	na	97
YJL210W	PEX2	1	unassigned	na	165
YJL210W	PEX2	2	psiblast	1chc__	106
YJL211C	YJL211C	1	unassigned	na	147
YJL212C	OPT1	1	pfam	PF03169	799
YJL213W	YJL213W	1	psiblast	1e9yB_	331
YJL214W	HXT8	1	psiblast	1ja5A_	471
YJL214W	HXT8	2	psiblast	1ja5A_	98
YJL215C	YJL215C	1	unassigned	na	119
YJL216C	YJL216C	1	psiblast	1uok__	297
YJL216C	YJL216C	2	psiblast	1uok__	284
YJL217W	YJL217W	1	unassigned	na	198
YJL218W	YJL218W	1	psiblast	1krrA_	196
YJL219W	HXT9	1	psiblast	1ja5A_	470
YJL219W	HXT9	2	psiblast	1ja5A_	97
YJL222W	VTH2	1	msa	na	182
YJL222W	VTH2	2	unassigned	na	354
YJL222W	VTH2	3	msa	na	186
YJL222W	VTH2	4	msa	na	676
YJL222W	VTH2	5	unassigned	na	151
YJL225C	YJL225C	1	msa	na	238
YJL225C	YJL225C	2	msa	na	211
YJL225C	YJL225C	3	unassigned	na	107
YJL225C	YJL225C	4	psiblast	1fuuA_	538
YJL225C	YJL225C	5	fold_recogniti	1m2vB	345
YJL225C	YJL225C	6	unassigned	na	64
YJL225C	YJL225C	7	fold_recogniti	1itwA	255
YJR001W	AVT1	1	unassigned	na	179
YJR001W	AVT1	2	pfam	PF01490	423
YJR002W	MPP10	1	fold_recogniti	1l8mA	78
YJR002W	MPP10	2	fold_recogniti	1l8mA	79
YJR002W	MPP10	3	fold_recogniti	1l8mA	67
YJR002W	MPP10	4	fold_recogniti	1l8mA	78
YJR002W	MPP10	5	msa	na	199
YJR002W	MPP10	6	unassigned	na	92
YJR003C	YJR003C	1	fold_recogniti	1n1bA	307
YJR003C	YJR003C	2	fold_recogniti	1n1bA	140
YJR003C	YJR003C	3	unassigned	na	92
YJR004C	SAG1	1	fold_recogniti	1jv2B	369
YJR004C	SAG1	2	fold_recogniti	1jv2B	92
YJR004C	SAG1	3	fold_recogniti	1m2vB	189

YJR005W	APL1	1	psiblast	1gw5B_	97
YJR005W	APL1	2	psiblast	1gw5B_	108
YJR005W	APL1	3	psiblast	1gw5B_	178
YJR005W	APL1	4	psiblast	1gw5B_	93
YJR005W	APL1	5	psiblast	1gw5B_	135
YJR005W	APL1	6	unassigned	na	89
YJR006W	HYS2	1	unassigned	na	141
YJR006W	HYS2	2	msa	na	346
YJR007W	SUI2	1	psiblast	1kl9A_	104
YJR007W	SUI2	2	psiblast	1kl9A_	118
YJR007W	SUI2	3	unassigned	na	82
YJR008W	YJR008W	1	fold_recogniti	1b4uB	213
YJR008W	YJR008W	2	unassigned	na	125
YJR009C	TDH2	1	psiblast	1crwG_	119
YJR009C	TDH2	2	psiblast	1crwG_	44
YJR009C	TDH2	3	psiblast	1crwG_	169
YJR010C-A	SPC1	1	unassigned	na	94
YJR010W	MET3	1	psiblast	1g8fA_	166
YJR010W	MET3	2	psiblast	1g8fA_	164
YJR010W	MET3	3	psiblast	1g8fA_	59
YJR010W	MET3	4	psiblast	1g8fA_	122
YJR011C	YJR011C	1	unassigned	na	261
YJR012C	YJR012C	1	unassigned	na	207
YJR013W	PMH1	1	msa	na	160
YJR013W	PMH1	2	unassigned	na	145
YJR014W	YJR014W	1	unassigned	na	62
YJR014W	YJR014W	2	psiblast	1d1rA_	136
YJR015W	YJR015W	1	unassigned	na	510
YJR016C	ILV3	1	fold_recogniti	1b8fA	100
YJR016C	ILV3	2	fold_recogniti	1o0uA	220
YJR016C	ILV3	3	fold_recogniti	1o0uA	265
YJR017C	ESS1	1	psiblast	1f8aB_	190
YJR018W	YJR018W	1	unassigned	na	120
YJR019C	TES1	1	psiblast	1c8uA_	349
YJR020W	YJR020W	1	unassigned	na	110
YJR021C	REC107	1	unassigned	na	191
YJR021C	REC107	2	msa	na	123
YJR022W	LSM8	1	psiblast	1d3bB_	128
YJR023C	YJR023C	1	unassigned	na	133
YJR024C	YJR024C	1	psiblast	1jdiA_	244
YJR025C	BNA1	1	unassigned	na	177
YJR030C	YJR030C	1	msa	na	420
YJR030C	YJR030C	2	unassigned	na	325
YJR031C	GEA1	1	msa	na	521
YJR031C	GEA1	2	psiblast	1ku1A_	87
YJR031C	GEA1	3	psiblast	1ku1A_	150
YJR031C	GEA1	4	psiblast	1fhwA_	61
YJR031C	GEA1	5	fold_recogniti	1b3uA	229

YJR031C	GEA1	6	fold_recogniti	1b3uA	68
YJR031C	GEA1	7	fold_recogniti	1b3uA	292
YJR032W	CPR7	1	psiblast	1ihgA_	209
YJR032W	CPR7	2	psiblast	1ihgA_	96
YJR032W	CPR7	3	psiblast	1ihgA_	88
YJR033C	RAV1	1	psiblast	1gg2B_	98
YJR033C	RAV1	2	psiblast	1erjA_	400
YJR033C	RAV1	3	unassigned	na	329
YJR033C	RAV1	4	fold_recogniti	1n1bA	151
YJR033C	RAV1	5	fold_recogniti	1n1bA	135
YJR033C	RAV1	6	unassigned	na	244
YJR034W	PET191	1	unassigned	na	108
YJR035W	RAD26	1	msa	na	285
YJR035W	RAD26	2	pfam	PF00176	315
YJR035W	RAD26	3	psiblast	1hv8A_	170
YJR035W	RAD26	4	msa	na	188
YJR035W	RAD26	5	msa	na	127
YJR036C	HUL4	1	unassigned	na	448
YJR036C	HUL4	2	psiblast	1c4zA_	111
YJR036C	HUL4	3	psiblast	1c4zA_	147
YJR036C	HUL4	4	psiblast	1c4zA_	186
YJR037W	YJR037W	1	unassigned	na	127
YJR038C	YJR038C	1	unassigned	na	120
YJR039W	YJR039W	1	unassigned	na	1121
YJR040W	GEF1	1	psiblast	1kplA_	212
YJR040W	GEF1	2	psiblast	1kplA_	359
YJR040W	GEF1	3	psiblast	1b3oA_	208
YJR041C	URB2	1	fold_recogniti	1b3uA	267
YJR041C	URB2	2	fold_recogniti	1b3uA	69
YJR041C	URB2	3	fold_recogniti	1b3uA	173
YJR041C	URB2	4	fold_recogniti	1b3uA	239
YJR041C	URB2	5	unassigned	na	255
YJR041C	URB2	6	msa	na	171
YJR042W	NUP85	1	unassigned	na	134
YJR042W	NUP85	2	msa	na	544
YJR042W	NUP85	3	unassigned	na	66
YJR043C	POL32	1	unassigned	na	74
YJR043C	POL32	2	msa	na	276
YJR044C	VPS55	1	unassigned	na	140
YJR045C	SSC1	1	psiblast	1dkgD_	213
YJR045C	SSC1	2	psiblast	1dkgD_	118
YJR045C	SSC1	3	psiblast	1dkgD_	77
YJR045C	SSC1	4	psiblast	1dkyA_	146
YJR045C	SSC1	5	psiblast	1dkyA_	100
YJR046W	TAH11	1	unassigned	na	604
YJR047C	ANB1	1	psiblast	1fh4A_	85
YJR047C	ANB1	2	psiblast	1fh4A_	72
YJR048W	CYC1	1	psiblast	2pccB_	109

YJR049C	UTR1	1	unassigned	na	101
YJR049C	UTR1	2	pfam	PF01513	353
YJR049C	UTR1	3	unassigned	na	76
YJR050W	ISY1	1	fold_recogniti	1cunA	104
YJR050W	ISY1	2	fold_recogniti	1cunA	131
YJR051W	OSM1	1	psiblast	1qo8A_	348
YJR051W	OSM1	2	psiblast	1qo8A_	153
YJR052W	RAD7	1	unassigned	na	55
YJR052W	RAD7	2	psiblast	2bnh__	161
YJR052W	RAD7	3	psiblast	2bnh__	55
YJR052W	RAD7	4	psiblast	2bnh__	53
YJR052W	RAD7	5	psiblast	2bnh__	126
YJR052W	RAD7	6	psiblast	2bnh__	115
YJR053W	BFA1	1	unassigned	na	325
YJR053W	BFA1	2	msa	na	115
YJR053W	BFA1	3	unassigned	na	134
YJR054W	YJR054W	1	msa	na	247
YJR054W	YJR054W	2	unassigned	na	250
YJR055W	HIT1	1	unassigned	na	164
YJR056C	YJR056C	1	unassigned	na	236
YJR057W	CDC8	1	psiblast	1tmkA_	216
YJR058C	APS2	1	psiblast	1gw5S_	90
YJR058C	APS2	2	psiblast	1gw5S_	57
YJR059W	PTK2	1	unassigned	na	225
YJR059W	PTK2	2	psiblast	1aq1__	118
YJR059W	PTK2	3	psiblast	1aq1__	102
YJR059W	PTK2	4	psiblast	1aq1__	69
YJR059W	PTK2	5	psiblast	1aq1__	70
YJR059W	PTK2	6	psiblast	1a29__	87
YJR059W	PTK2	7	psiblast	1a29__	89
YJR059W	PTK2	8	unassigned	na	58
YJR060W	CBF1	1	unassigned	na	168
YJR060W	CBF1	2	psiblast	1hloA_	183
YJR061W	YJR061W	1	msa	na	157
YJR061W	YJR061W	2	unassigned	na	287
YJR061W	YJR061W	3	msa	na	168
YJR061W	YJR061W	4	msa	na	323
YJR062C	NTA1	1	psiblast	1f89A_	342
YJR062C	NTA1	2	unassigned	na	115
YJR063W	RPA12	1	psiblast	1meyG_	125
YJR064W	CCT5	1	psiblast	1a6dA_	446
YJR064W	CCT5	2	psiblast	1a6dA_	116
YJR065C	ARP3	1	psiblast	1yagA_	223
YJR065C	ARP3	2	psiblast	1yagA_	226
YJR066W	TOR1	1	unassigned	na	85
YJR066W	TOR1	2	psiblast	1qgkA_	237
YJR066W	TOR1	3	psiblast	1qgkA_	181
YJR066W	TOR1	4	psiblast	1qgkA_	122

YJR066W	TOR1	5	psiblast	1qgkA_	135
YJR066W	TOR1	6	psiblast	1qgkA_	228
YJR066W	TOR1	7	psiblast	1b3uA_	238
YJR066W	TOR1	8	unassigned	na	97
YJR066W	TOR1	9	pfam	PF02259	596
YJR066W	TOR1	10	psiblast	1aueA_	165
YJR066W	TOR1	11	pfam	PF00454	352
YJR066W	TOR1	12	pfam	PF02260	34
YJR067C	YAE1	1	unassigned	na	141
YJR068W	RFC2	1	psiblast	1iqpA_	100
YJR068W	RFC2	2	psiblast	1iqpA_	253
YJR069C	HAM1	1	psiblast	1b78A_	110
YJR069C	HAM1	2	psiblast	1b78A_	87
YJR070C	LIA1	1	psiblast	1b3uA_	142
YJR070C	LIA1	2	psiblast	1b3uA_	183
YJR071W	YJR071W	1	unassigned	na	122
YJR072C	YJR072C	1	psiblast	2ffhA_	270
YJR072C	YJR072C	2	psiblast	2ffhA_	115
YJR073C	OPI3	1	msa	na	132
YJR073C	OPI3	2	msa	na	74
YJR074W	MOG1	1	psiblast	1eq6A_	218
YJR075W	HOC1	1	msa	na	328
YJR075W	HOC1	2	unassigned	na	68
YJR076C	CDC11	1	psiblast	1egaA_	175
YJR076C	CDC11	2	psiblast	1i84S_	240
YJR077C	MIR1	1	fold_recogniti	1is2A	311
YJR078W	BNA2	1	pfam	PF01231	453
YJR079W	YJR079W	1	unassigned	na	109
YJR080C	FMP26	1	unassigned	na	394
YJR082C	EAF6	1	unassigned	na	113
YJR083C	ACF4	1	unassigned	na	309
YJR084W	CSN12	1	unassigned	na	103
YJR084W	CSN12	2	msa	na	320
YJR085C	YJR085C	1	pfam	PF03647	105
YJR086W	STE18	1	psiblast	1scgG_	110
YJR087W	YJR087W	1	unassigned	na	116
YJR088C	YJR088C	1	psiblast	1fchA_	100
YJR088C	YJR088C	2	psiblast	1fchA_	77
YJR088C	YJR088C	3	psiblast	1fchA_	115
YJR089W	BIR1	1	pfam	PF00653	128
YJR089W	BIR1	2	fold_recogniti	1e31A	152
YJR089W	BIR1	3	msa	na	355
YJR089W	BIR1	4	unassigned	na	102
YJR089W	BIR1	5	msa	na	217
YJR090C	GRR1	1	unassigned	na	96
YJR090C	GRR1	2	psiblast	1k15A_	173
YJR090C	GRR1	3	unassigned	na	63
YJR090C	GRR1	4	psiblast	2bnh__	92

YJR090C	GRR1	5	psiblast	2bnh__	53
YJR090C	GRR1	6	psiblast	2bnh__	52
YJR090C	GRR1	7	psiblast	2bnh__	119
YJR090C	GRR1	8	psiblast	2bnh__	106
YJR090C	GRR1	9	psiblast	1jl5A_	291
YJR090C	GRR1	10	unassigned	na	106
YJR091C	JSN1	1	msa	na	299
YJR091C	JSN1	2	fold_recogniti	1h2tZ	149
YJR091C	JSN1	3	unassigned	na	118
YJR091C	JSN1	4	psiblast	1ib2A_	125
YJR091C	JSN1	5	psiblast	1ib2A_	101
YJR091C	JSN1	6	psiblast	1ib2A_	150
YJR091C	JSN1	7	unassigned	na	149
YJR092W	BUD4	1	msa	na	164
YJR092W	BUD4	2	unassigned	na	596
YJR092W	BUD4	3	msa	na	119
YJR092W	BUD4	4	unassigned	na	382
YJR092W	BUD4	5	pfam	PF00169	187
YJR093C	FIP1	1	msa	na	258
YJR093C	FIP1	2	unassigned	na	69
YJR094C	IME1	1	unassigned	na	360
YJR094W-A	RPL43B	1	psiblast	1k5y2_	92
YJR095W	SFC1	1	pfam	PF00153	107
YJR095W	SFC1	2	pfam	PF00153	104
YJR095W	SFC1	3	pfam	PF00153	111
YJR096W	YJR096W	1	psiblast	1ihiA_	220
YJR096W	YJR096W	2	psiblast	1ihiA_	62
YJR097W	JJJ3	1	psiblast	1xbl__	172
YJR098C	YJR098C	1	unassigned	na	301
YJR098C	YJR098C	2	msa	na	206
YJR098C	YJR098C	3	unassigned	na	148
YJR099W	YUH1	1	psiblast	1cmxA_	236
YJR100C	YJR100C	1	pfam	PF03803	327
YJR101W	RSM26	1	psiblast	1kkcA_	266
YJR102C	VPS25	1	unassigned	na	115
YJR102C	VPS25	2	msa	na	87
YJR103W	URA8	1	fold_recogniti	1cp2A	208
YJR103W	URA8	2	psiblast	1bxrB_	356
YJR104C	SOD1	1	psiblast	1b4lA_	154
YJR105W	ADO1	1	psiblast	1lnjA_	340
YJR106W	ECM27	1	fold_recogniti	1l9uD	130
YJR106W	ECM27	2	fold_recogniti	1itwA	292
YJR106W	ECM27	3	fold_recogniti	1itwA	174
YJR106W	ECM27	4	msa	na	129
YJR107W	YJR107W	1	psiblast	1dt3A_	328
YJR108W	ABM1	1	unassigned	na	123
YJR109C	CPA2	1	psiblast	1bxrA_	682
YJR109C	CPA2	2	psiblast	1bxrA_	322

YJR109C	CPA2	3	psiblast	1bxrA_	114
YJR110W	YJR110W	1	msa	na	170
YJR110W	YJR110W	2	msa	na	518
YJR111C	YJR111C	1	unassigned	na	137
YJR111C	YJR111C	2	msa	na	146
YJR112W	NNF1	1	msa	na	201
YJR113C	RSM7	1	unassigned	na	89
YJR113C	RSM7	2	psiblast	1rss__	62
YJR113C	RSM7	3	psiblast	1rss__	96
YJR114W	SRF2	1	unassigned	na	130
YJR115W	YJR115W	1	unassigned	na	169
YJR116W	YJR116W	1	msa	na	279
YJR117W	STE24	1	unassigned	na	226
YJR117W	STE24	2	pfam	PF01435	227
YJR118C	ILM1	1	msa	na	203
YJR119C	YJR119C	1	pfam	PF02375	70
YJR119C	YJR119C	2	unassigned	na	161
YJR119C	YJR119C	3	psiblast	1f62A_	87
YJR119C	YJR119C	4	unassigned	na	59
YJR119C	YJR119C	5	fold_recogniti	1mzeA	177
YJR119C	YJR119C	6	msa	na	174
YJR120W	YJR120W	1	unassigned	na	116
YJR121W	ATP2	1	psiblast	1bmfD_	115
YJR121W	ATP2	2	psiblast	1bmfD_	255
YJR121W	ATP2	3	psiblast	1bmfD_	141
YJR122W	CAF17	1	unassigned	na	132
YJR122W	CAF17	2	msa	na	365
YJR123W	RPS5	1	fold_recogniti	1iqvA	75
YJR123W	RPS5	2	psiblast	1k5xG_	150
YJR124C	YJR124C	1	psiblast	1ja5A_	448
YJR125C	ENT3	1	psiblast	1inzA_	167
YJR125C	ENT3	2	msa	na	241
YJR126C	VPS70	1	unassigned	na	124
YJR126C	VPS70	2	psiblast	1de4C_	352
YJR126C	VPS70	3	psiblast	1de4C_	335
YJR127C	ZMS1	1	psiblast	2gliA_	92
YJR127C	ZMS1	2	unassigned	na	110
YJR127C	ZMS1	3	psiblast	2drpA_	45
YJR127C	ZMS1	4	msa	na	76
YJR127C	ZMS1	5	unassigned	na	239
YJR127C	ZMS1	6	msa	na	818
YJR128W	YJR128W	1	unassigned	na	119
YJR129C	YJR129C	1	msa	na	121
YJR129C	YJR129C	2	psiblast	1g6q1_	218
YJR130C	STR2	1	psiblast	1h7dA_	107
YJR130C	STR2	2	unassigned	na	73
YJR130C	STR2	3	psiblast	1gc0A_	321
YJR130C	STR2	4	psiblast	1gc0A_	138

YJR131W	MNS1	1	psiblast	1dl2A_	357
YJR131W	MNS1	2	psiblast	1dl2A_	192
YJR132W	NMD5	1	msa	na	726
YJR132W	NMD5	2	msa	na	140
YJR132W	NMD5	3	msa	na	182
YJR133W	XPT1	1	psiblast	1bzyA_	209
YJR134C	SGM1	1	psiblast	1i84S_	244
YJR134C	SGM1	2	unassigned	na	66
YJR134C	SGM1	3	psiblast	2tmaA_	104
YJR134C	SGM1	4	msa	na	293
YJR135C	MCM22	1	unassigned	na	239
YJR135W-A	TIM8	1	pfam	PF02953	87
YJR136C	YJR136C	1	unassigned	na	421
YJR137C	ECM17	1	fold_recogniti	1ik6A	192
YJR137C	ECM17	2	fold_recogniti	1ik6A	127
YJR137C	ECM17	3	psiblast	1b0pA_	360
YJR137C	ECM17	4	psiblast	1b1cA_	178
YJR137C	ECM17	5	psiblast	1aop__	332
YJR137C	ECM17	6	psiblast	1aop__	253
YJR138W	IML1	1	unassigned	na	113
YJR138W	IML1	2	msa	na	460
YJR138W	IML1	3	unassigned	na	279
YJR138W	IML1	4	msa	na	101
YJR138W	IML1	5	unassigned	na	217
YJR138W	IML1	6	psiblast	1fshA_	134
YJR138W	IML1	7	unassigned	na	280
YJR139C	HOM6	1	psiblast	1ebfA_	163
YJR139C	HOM6	2	psiblast	1ebfA_	196
YJR140C	HIR3	1	unassigned	na	521
YJR140C	HIR3	2	msa	na	1127
YJR141W	YJR141W	1	unassigned	na	347
YJR142W	YJR142W	1	unassigned	na	121
YJR142W	YJR142W	2	psiblast	1hztA_	221
YJR143C	PMT4	1	pfam	PF02366	322
YJR143C	PMT4	2	pfam	PF02815	69
YJR143C	PMT4	3	fold_recogniti	1n4kA	147
YJR143C	PMT4	4	unassigned	na	73
YJR143C	PMT4	5	msa	na	151
YJR144W	MGM101	1	unassigned	na	77
YJR144W	MGM101	2	msa	na	192
YJR146W	YJR146W	1	unassigned	na	117
YJR147W	HMS2	1	psiblast	3htsB_	113
YJR147W	HMS2	2	msa	na	140
YJR147W	HMS2	3	unassigned	na	105
YJR148W	BAT2	1	psiblast	1ekfA_	195
YJR148W	BAT2	2	psiblast	1ekfA_	181
YJR149W	YJR149W	1	psiblast	1jr1A_	404
YJR150C	DAN1	1	fold_recogniti	1m2vB	298

YJR151C	DAN4	1	pfam	PF00660	125
YJR151C	DAN4	2	unassigned	na	110
YJR151C	DAN4	3	msa	na	388
YJR151C	DAN4	4	msa	na	239
YJR151C	DAN4	5	msa	na	299
YJR152W	DAL5	1	unassigned	na	73
YJR152W	DAL5	2	psiblast	1ja5A_	470
YJR153W	PGU1	1	psiblast	1czfA_	361
YJR154W	YJR154W	1	msa	na	346
YJR155W	AAD10	1	psiblast	1gveB_	288
YJR156C	THI11	1	psiblast	1gggA_	188
YJR156C	THI11	2	psiblast	1gggA_	98
YJR156C	THI11	3	unassigned	na	54
YJR157W	YJR157W	1	unassigned	na	120
YJR158W	HXT16	1	psiblast	1ja5A_	465
YJR158W	HXT16	2	psiblast	1ja5A_	102
YJR159W	SOR1	1	psiblast	1e3jA_	188
YJR159W	SOR1	2	psiblast	1e3jA_	169
YJR160C	MPH3	1	unassigned	na	68
YJR160C	MPH3	2	psiblast	1ja5A_	429
YJR160C	MPH3	3	psiblast	1ja5A_	105
YJR161C	COS5	1	msa	na	61
YJR161C	COS5	2	pfam	PF00674	108
YJR161C	COS5	3	unassigned	na	53
YJR161C	COS5	4	pfam	PF00674	161
YJR162C	YJR162C	1	unassigned	na	116
YKL001C	MET14	1	psiblast	1d6jA_	202
YKL002W	DID4	1	pfam	PF03357	151
YKL002W-A	3548	1	unassigned	na	39
YKL003C	MRP17	1	psiblast	1fjgF_	131
YKL004W	AUR1	1	unassigned	na	220
YKL004W	AUR1	2	fold_recogniti	1d2tA	125
YKL004W	AUR1	3	unassigned	na	56
YKL005C	BYE1	1	fold_recogniti	1fp0A	193
YKL005C	BYE1	2	unassigned	na	61
YKL005C	BYE1	3	psiblast	1enwA_	97
YKL005C	BYE1	4	msa	na	71
YKL005C	BYE1	5	unassigned	na	172
YKL006C-A	SFT1	1	psiblast	1sfcD_	97
YKL006W	RPL14A	1	pfam	PF00467	50
YKL006W	RPL14A	2	pfam	PF01929	88
YKL007W	CAP1	1	pfam	PF01267	268
YKL008C	LAC1	1	msa	na	164
YKL008C	LAC1	2	pfam	PF03798	254
YKL009W	MRT4	1	psiblast	1jj2G_	236
YKL010C	UFD4	1	unassigned	na	72
YKL010C	UFD4	2	psiblast	1qgkB_	86
YKL010C	UFD4	3	psiblast	1ejlI_	136

YKL010C	UFD4	4	psiblast	1ejlI_	49
YKL010C	UFD4	5	psiblast	1ejlI_	114
YKL010C	UFD4	6	psiblast	1ejlI_	111
YKL010C	UFD4	7	psiblast	1ejlI_	102
YKL010C	UFD4	8	unassigned	na	303
YKL010C	UFD4	9	psiblast	1i5hW_	119
YKL010C	UFD4	10	psiblast	1c4zA_	46
YKL010C	UFD4	11	psiblast	1c4zA_	168
YKL010C	UFD4	12	psiblast	1c4zA_	177
YKL011C	CCE1	1	psiblast	1kcfA_	353
YKL012W	PRP40	1	psiblast	1e0lA_	33
YKL012W	PRP40	2	psiblast	1i5hW_	49
YKL012W	PRP40	3	unassigned	na	71
YKL012W	PRP40	4	psiblast	1i84S_	341
YKL012W	PRP40	5	pfam	PF01846	89
YKL013C	ARC19	1	psiblast	1k8kF_	171
YKL014C	URB1	1	fold_recogniti	1b3uA	230
YKL014C	URB1	2	fold_recogniti	1b3uA	74
YKL014C	URB1	3	fold_recogniti	1b3uA	152
YKL014C	URB1	4	fold_recogniti	1b3uA	237
YKL014C	URB1	5	unassigned	na	1071
YKL015W	PUT3	1	psiblast	1ajyA_	132
YKL015W	PUT3	2	msa	na	681
YKL015W	PUT3	3	unassigned	na	166
YKL016C	ATP7	1	fold_recogniti	2tmaB	101
YKL016C	ATP7	2	fold_recogniti	2tmaB	73
YKL017C	HCS1	1	msa	na	186
YKL017C	HCS1	2	psiblast	1pjr__	497
YKL018C-A	YKL018C-A	1	unassigned	na	99
YKL018W	SWD2	1	psiblast	1gg2B_	329
YKL019W	RAM2	1	psiblast	1jcqA_	124
YKL019W	RAM2	2	psiblast	1jcqA_	84
YKL019W	RAM2	3	psiblast	1jcqA_	108
YKL020C	SPT23	1	unassigned	na	181
YKL020C	SPT23	2	msa	na	319
YKL020C	SPT23	3	pfam	PF01833	85
YKL020C	SPT23	4	psiblast	1iknD_	75
YKL020C	SPT23	5	psiblast	1iknD_	77
YKL020C	SPT23	6	psiblast	1iknD_	62
YKL020C	SPT23	7	msa	na	228
YKL020C	SPT23	8	unassigned	na	55
YKL021C	MAK11	1	psiblast	1gg2B_	104
YKL021C	MAK11	2	psiblast	1erjA_	364
YKL022C	CDC16	1	psiblast	1elwA_	92
YKL022C	CDC16	2	unassigned	na	114
YKL022C	CDC16	3	psiblast	1hz4A_	96
YKL022C	CDC16	4	psiblast	1hz4A_	116
YKL022C	CDC16	5	psiblast	1fchA_	161

YKL022C	CDC16	6	psiblast	1fchA_	68
YKL022C	CDC16	7	psiblast	1fchA_	141
YKL022C	CDC16	8	unassigned	na	52
YKL023W	YKL023W	1	psiblast	1i84S_	277
YKL024C	URA6	1	psiblast	1uky__	204
YKL025C	PAN3	1	unassigned	na	224
YKL025C	PAN3	2	psiblast	1a06__	177
YKL025C	PAN3	3	psiblast	1a06__	184
YKL025C	PAN3	4	psiblast	1dtlA_	94
YKL026C	GPX1	1	psiblast	1gp1A_	167
YKL027W	YKL027W	1	psiblast	1jw9B_	372
YKL027W	YKL027W	2	unassigned	na	75
YKL028W	TFA1	1	pfam	PF02002	219
YKL028W	TFA1	2	msa	na	263
YKL029C	MAE1	1	msa	na	97
YKL029C	MAE1	2	psiblast	1gq2A_	278
YKL029C	MAE1	3	psiblast	1gq2A_	294
YKL030W	YKL030W	1	unassigned	na	201
YKL031W	YKL031W	1	unassigned	na	137
YKL032C	IXR1	1	msa	na	121
YKL032C	IXR1	2	unassigned	na	52
YKL032C	IXR1	3	psiblast	1k99A_	163
YKL032C	IXR1	4	psiblast	1i11A_	91
YKL032C	IXR1	5	psiblast	1cg7A_	84
YKL032C	IXR1	6	msa	na	86
YKL033W	FMP47	1	fold_recogniti	1i7wC	173
YKL033W	FMP47	2	fold_recogniti	1i7wC	109
YKL033W	FMP47	3	fold_recogniti	1i7wC	107
YKL033W	FMP47	4	fold_recogniti	1i7wC	42
YKL033W	FMP47	5	fold_recogniti	1i7wC	123
YKL033W	FMP47	6	msa	na	420
YKL033W	FMP47	7	unassigned	na	64
YKL033W-A	YKL033W-A	1	fold_recogniti	1lvhA	60
YKL034W	TUL1	1	unassigned	na	360
YKL034W	TUL1	2	msa	na	237
YKL034W	TUL1	3	unassigned	na	96
YKL034W	TUL1	4	psiblast	1chc__	65
YKL035W	UGP1	1	unassigned	na	96
YKL035W	UGP1	2	fold_recogniti	1hv9A	403
YKL036C	YKL036C	1	unassigned	na	130
YKL037W	YKL037W	1	unassigned	na	118
YKL038W	RGT1	1	psiblast	1hwtC_	120
YKL038W	RGT1	2	msa	na	350
YKL038W	RGT1	3	unassigned	na	700
YKL039W	PTM1	1	unassigned	na	70
YKL039W	PTM1	2	msa	na	461
YKL040C	NFU1	1	msa	na	145
YKL040C	NFU1	2	pfam	PF01106	111

YKL041W	VPS24	1	fold_recogniti	1av1A	224
YKL042W	SPC42	1	unassigned	na	363
YKL043W	PHD1	1	msa	na	194
YKL043W	PHD1	2	psiblast	1bm8__	95
YKL043W	PHD1	3	unassigned	na	77
YKL044W	YKL044W	1	unassigned	na	106
YKL045W	PRI2	1	msa	na	139
YKL045W	PRI2	2	unassigned	na	81
YKL045W	PRI2	3	msa	na	248
YKL045W	PRI2	4	unassigned	na	60
YKL046C	DCW1	1	fold_recogniti	1fo2A	258
YKL046C	DCW1	2	fold_recogniti	1fo2A	191
YKL047W	YKL047W	1	unassigned	na	516
YKL048C	ELM1	1	psiblast	1a06__	225
YKL048C	ELM1	2	psiblast	1a06__	218
YKL048C	ELM1	3	psiblast	1ahr__	71
YKL048C	ELM1	4	psiblast	1ahr__	126
YKL049C	CSE4	1	psiblast	1eqzC_	137
YKL050C	YKL050C	1	unassigned	na	72
YKL050C	YKL050C	2	psiblast	1i84S_	323
YKL050C	YKL050C	3	psiblast	1c1gA_	224
YKL050C	YKL050C	4	msa	na	249
YKL050C	YKL050C	5	unassigned	na	54
YKL051W	SFK1	1	unassigned	na	353
YKL052C	ASK1	1	unassigned	na	96
YKL052C	ASK1	2	msa	na	137
YKL052C	ASK1	3	unassigned	na	59
YKL053C-A	MDM35	1	unassigned	na	86
YKL053W	YKL053W	1	unassigned	na	124
YKL054C	DEF1	1	unassigned	na	176
YKL054C	DEF1	2	fold_recogniti	1l8mA	82
YKL054C	DEF1	3	fold_recogniti	1l8mA	73
YKL054C	DEF1	4	fold_recogniti	1l8mA	83
YKL054C	DEF1	5	fold_recogniti	1l8mA	70
YKL054C	DEF1	6	fold_recogniti	1deqA	129
YKL054C	DEF1	7	unassigned	na	125
YKL055C	OAR1	1	psiblast	1edoA_	278
YKL056C	YKL056C	1	psiblast	1h7yA_	167
YKL057C	NUP120	1	msa	na	479
YKL057C	NUP120	2	msa	na	558
YKL058W	TOA2	1	psiblast	1ytfD_	57
YKL058W	TOA2	2	psiblast	1ytfD_	65
YKL059C	MPE1	1	msa	na	69
YKL059C	MPE1	2	msa	na	199
YKL059C	MPE1	3	psiblast	1rmd__	173
YKL060C	FBA1	1	psiblast	1dosA_	359
YKL061W	YKL061W	1	unassigned	na	113
YKL062W	MSN4	1	msa	na	212

YKL062W	MSN4	2	msa	na	137
YKL062W	MSN4	3	psiblast	1tf3A_	111
YKL062W	MSN4	4	psiblast	1zaaC_	97
YKL062W	MSN4	5	psiblast	1a1fA_	73
YKL063C	YKL063C	1	unassigned	na	167
YKL064W	MNR2	1	unassigned	na	467
YKL064W	MNR2	2	pfam	PF01544	502
YKL065C	YET1	1	msa	na	206
YKL066W	YKL066W	1	unassigned	na	147
YKL067W	YNK1	1	psiblast	1nueA_	153
YKL068W	NUP100	1	msa	na	752
YKL068W	NUP100	2	msa	na	207
YKL069W	YKL069W	1	psiblast	1f5mA_	61
YKL069W	YKL069W	2	psiblast	1f5mA_	119
YKL070W	YKL070W	1	unassigned	na	169
YKL071W	YKL071W	1	psiblast	1i01A_	256
YKL072W	STB6	1	unassigned	na	469
YKL072W	STB6	2	msa	na	297
YKL073W	LHS1	1	psiblast	1ba0__	228
YKL073W	LHS1	2	psiblast	1ba0__	131
YKL073W	LHS1	3	psiblast	1ba0__	79
YKL073W	LHS1	4	psiblast	1dkxA_	164
YKL073W	LHS1	5	psiblast	1dkxA_	107
YKL073W	LHS1	6	unassigned	na	172
YKL074C	MUD2	1	msa	na	392
YKL074C	MUD2	2	psiblast	1jmtA_	135
YKL075C	YKL075C	1	unassigned	na	450
YKL076C	PSY1	1	unassigned	na	127
YKL077W	YKL077W	1	unassigned	na	392
YKL078W	DHR2	1	psiblast	1fuuA_	487
YKL078W	DHR2	2	unassigned	na	57
YKL078W	DHR2	3	msa	na	191
YKL079W	SMY1	1	psiblast	1gojA_	366
YKL079W	SMY1	2	psiblast	1i84S_	290
YKL080W	VMA5	1	pfam	PF03223	392
YKL081W	TEF4	1	psiblast	1bx9A_	80
YKL081W	TEF4	2	psiblast	1bx9A_	167
YKL081W	TEF4	3	pfam	PF00647	165
YKL082C	RRP14	1	msa	na	219
YKL082C	RRP14	2	msa	na	215
YKL083W	YKL083W	1	unassigned	na	204
YKL084W	YKL084W	1	unassigned	na	116
YKL085W	MDH1	1	psiblast	1mldA_	334
YKL086W	SRX1	1	pfam	PF02195	127
YKL087C	CYT2	1	pfam	PF01265	224
YKL088W	YKL088W	1	fold_recogniti	1c1gB	275
YKL088W	YKL088W	2	psiblast	1e20A_	296
YKL089W	MIF2	1	fold_recogniti	1l8mA	91

YKL089W	MIF2	2	fold_recogniti	1l8mA	74
YKL089W	MIF2	3	fold_recogniti	1l8mA	79
YKL089W	MIF2	4	fold_recogniti	1l8mA	70
YKL089W	MIF2	5	unassigned	na	156
YKL089W	MIF2	6	msa	na	79
YKL090W	CUE2	1	pfam	PF02845	52
YKL090W	CUE2	2	pfam	PF02845	51
YKL090W	CUE2	3	unassigned	na	228
YKL090W	CUE2	4	msa	na	112
YKL091C	YKL091C	1	psiblast	1aua__	249
YKL091C	YKL091C	2	psiblast	1aua__	61
YKL092C	BUD2	1	unassigned	na	194
YKL092C	BUD2	2	psiblast	1dqvA_	122
YKL092C	BUD2	3	psiblast	1dqvA_	150
YKL092C	BUD2	4	psiblast	1wer__	138
YKL092C	BUD2	5	psiblast	1wer__	223
YKL092C	BUD2	6	unassigned	na	277
YKL093W	MBR1	1	unassigned	na	107
YKL093W	MBR1	2	msa	na	232
YKL094W	YJU3	1	psiblast	1cqzA_	313
YKL095W	YJU2	1	msa	na	212
YKL095W	YJU2	2	unassigned	na	66
YKL096W	CWP1	1	unassigned	na	239
YKL096W-A	CWP2	1	unassigned	na	92
YKL097C	YKL097C	1	unassigned	na	136
YKL098W	YKL098W	1	psiblast	1g8pA_	218
YKL098W	YKL098W	2	psiblast	1g8pA_	139
YKL099C	UTP11	1	fold_recogniti	1c1gB	256
YKL100C	YKL100C	1	unassigned	na	259
YKL100C	YKL100C	2	msa	na	328
YKL101W	HSL1	1	psiblast	1a06__	215
YKL101W	HSL1	2	psiblast	1a06__	193
YKL101W	HSL1	3	psiblast	1ahr__	92
YKL101W	HSL1	4	psiblast	1ahr__	77
YKL101W	HSL1	5	unassigned	na	94
YKL101W	HSL1	6	fold_recogniti	1k83A	150
YKL101W	HSL1	7	fold_recogniti	1ivoA	176
YKL101W	HSL1	8	fold_recogniti	1ivoA	358
YKL101W	HSL1	9	unassigned	na	163
YKL102C	YKL102C	1	unassigned	na	101
YKL103C	LAP4	1	pfam	PF02127	514
YKL104C	GFA1	1	psiblast	1jxaA_	302
YKL104C	GFA1	2	psiblast	1jxaA_	260
YKL104C	GFA1	3	psiblast	1jxaA_	155
YKL105C	YKL105C	1	unassigned	na	142
YKL105C	YKL105C	2	psiblast	1i84S_	359
YKL105C	YKL105C	3	fold_recogniti	1l8mA	72
YKL105C	YKL105C	4	fold_recogniti	1l8mA	83

YKL105C	YKL105C	5	fold_recogniti	1l8mA	70
YKL105C	YKL105C	6	fold_recogniti	1l8mA	85
YKL105C	YKL105C	7	unassigned	na	88
YKL105C	YKL105C	8	msa	na	233
YKL106C-A	YKL106C-A	1	unassigned	na	39
YKL106W	AAT1	1	psiblast	1yaaA_	145
YKL106W	AAT1	2	psiblast	1yaaA_	306
YKL107W	YKL107W	1	psiblast	1ae1A_	309
YKL108W	SLD2	1	fold_recogniti	1ldvA	315
YKL108W	SLD2	2	unassigned	na	138
YKL109W	HAP4	1	unassigned	na	216
YKL109W	HAP4	2	fold_recogniti	1iu4A	338
YKL110C	KTI12	1	psiblast	1ak2__	217
YKL110C	KTI12	2	psiblast	1jhdA_	96
YKL111C	YKL111C	1	unassigned	na	111
YKL112W	ABF1	1	unassigned	na	78
YKL112W	ABF1	2	fold_recogniti	1l8mA	98
YKL112W	ABF1	3	fold_recogniti	1l8mA	69
YKL112W	ABF1	4	fold_recogniti	1l8mA	75
YKL112W	ABF1	5	fold_recogniti	1itwA	204
YKL112W	ABF1	6	fold_recogniti	1itwA	207
YKL113C	RAD27	1	psiblast	1b43A_	296
YKL113C	RAD27	2	psiblast	1b43A_	86
YKL114C	APN1	1	psiblast	1qtwA_	367
YKL115C	YKL115C	1	unassigned	na	130
YKL116C	PRR1	1	unassigned	na	128
YKL116C	PRR1	2	psiblast	1kmwC_	128
YKL116C	PRR1	3	psiblast	1kmwC_	262
YKL117W	SBA1	1	psiblast	1ejfA_	216
YKL118W	YKL118W	1	unassigned	na	103
YKL119C	VPH2	1	msa	na	215
YKL120W	OAC1	1	fold_recogniti	1is2A	324
YKL121W	YKL121W	1	unassigned	na	107
YKL121W	YKL121W	2	psiblast	1erjA_	167
YKL121W	YKL121W	3	psiblast	1scgB_	337
YKL121W	YKL121W	4	psiblast	1c5kA_	241
YKL122C	SRP21	1	unassigned	na	167
YKL123W	YKL123W	1	unassigned	na	126
YKL124W	SSH4	1	unassigned	na	184
YKL124W	SSH4	2	pfam	PF00622	237
YKL124W	SSH4	3	unassigned	na	158
YKL125W	RRN3	1	fold_recogniti	1b3uA	272
YKL125W	RRN3	2	unassigned	na	232
YKL125W	RRN3	3	msa	na	123
YKL126W	YPK1	1	unassigned	na	152
YKL126W	YPK1	2	psiblast	1a25A_	153
YKL126W	YPK1	3	psiblast	1atpE_	256
YKL126W	YPK1	4	psiblast	1atpE_	119

YKL127W	PGM1	1	psiblast	1c47A_	192
YKL127W	PGM1	2	psiblast	1c47A_	149
YKL127W	PGM1	3	psiblast	1c47A_	102
YKL127W	PGM1	4	psiblast	1c47A_	127
YKL128C	PMU1	1	psiblast	1bif__	295
YKL129C	MYO3	1	psiblast	1lkxA_	641
YKL129C	MYO3	2	psiblast	1lkxA_	72
YKL129C	MYO3	3	psiblast	1wdcA_	63
YKL129C	MYO3	4	psiblast	1deqA_	321
YKL129C	MYO3	5	psiblast	2semA_	92
YKL129C	MYO3	6	unassigned	na	82
YKL130C	SHE2	1	unassigned	na	246
YKL131W	YKL131W	1	unassigned	na	173
YKL132C	RMA1	1	psiblast	1fgs__	276
YKL132C	RMA1	2	psiblast	1fgs__	154
YKL133C	YKL133C	1	unassigned	na	463
YKL134C	1-Oct	1	unassigned	na	84
YKL134C	1-Oct	2	psiblast	1i1iP_	399
YKL134C	1-Oct	3	psiblast	1i1iP_	287
YKL135C	APL2	1	psiblast	1gw5B_	103
YKL135C	APL2	2	psiblast	1gw5B_	107
YKL135C	APL2	3	psiblast	1gw5B_	192
YKL135C	APL2	4	psiblast	1gw5B_	95
YKL135C	APL2	5	psiblast	1gw5B_	136
YKL135C	APL2	6	unassigned	na	93
YKL136W	YKL136W	1	unassigned	na	132
YKL137W	YKL137W	1	unassigned	na	103
YKL138C	MRPL31	1	unassigned	na	131
YKL139W	CTK1	1	unassigned	na	144
YKL139W	CTK1	2	psiblast	1lfnA_	97
YKL139W	CTK1	3	psiblast	1lfnA_	287
YKL140W	TGL1	1	psiblast	1k8qA_	463
YKL140W	TGL1	2	unassigned	na	85
YKL141W	SDH3	1	unassigned	na	53
YKL141W	SDH3	2	pfam	PF01127	145
YKL142W	MRP8	1	unassigned	na	219
YKL143W	LTV1	1	msa	na	239
YKL143W	LTV1	2	unassigned	na	224
YKL144C	RPC25	1	psiblast	1go3E_	77
YKL144C	RPC25	2	psiblast	1go3E_	135
YKL145W	RPT1	1	psiblast	1e32A_	121
YKL145W	RPT1	2	psiblast	1e32A_	84
YKL145W	RPT1	3	psiblast	1e32A_	174
YKL145W	RPT1	4	psiblast	1e32A_	88
YKL146W	AVT3	1	msa	na	282
YKL146W	AVT3	2	pfam	PF01490	410
YKL147C	YKL147C	1	unassigned	na	205
YKL148C	SDH1	1	psiblast	1kf6A_	355

YKL148C	SDH1	2	psiblast	1kf6A_	285
YKL149C	DBR1	1	fold_recogniti	1kbpA	260
YKL149C	DBR1	2	msa	na	145
YKL150W	MCR1	1	psiblast	1ib0A_	148
YKL150W	MCR1	2	psiblast	1ib0A_	154
YKL151C	YKL151C	1	psiblast	1lljA_	337
YKL152C	GPM1	1	psiblast	1bq3D_	247
YKL153W	YKL153W	1	unassigned	na	169
YKL154W	SRP102	1	psiblast	1fzqA_	244
YKL155C	RSM22	1	msa	na	491
YKL155C	RSM22	2	unassigned	na	137
YKL156W	RPS27A	1	pfam	PF01667	82
YKL157W	APE2	1	psiblast	1hs6A_	210
YKL157W	APE2	2	psiblast	1hs6A_	93
YKL157W	APE2	3	psiblast	1hs6A_	202
YKL157W	APE2	4	fold_recogniti	1gw5B	183
YKL157W	APE2	5	fold_recogniti	1gw5B	156
YKL158W	3710	1	unassigned	na	100
YKL159C	RCN1	1	unassigned	na	211
YKL160W	ELF1	1	unassigned	na	145
YKL161C	YKL161C	1	psiblast	1gol__	191
YKL161C	YKL161C	2	psiblast	1gol__	242
YKL162C	YKL162C	1	unassigned	na	402
YKL162C-A	YKL162C-A	1	unassigned	na	50
YKL163W	PIR3	1	msa	na	207
YKL163W	PIR3	2	msa	na	118
YKL164C	PIR1	1	fold_recogniti	1kapP	128
YKL164C	PIR1	2	fold_recogniti	1kapP	77
YKL164C	PIR1	3	fold_recogniti	1kapP	136
YKL165C	MCD4	1	psiblast	1e1zP_	383
YKL165C	MCD4	2	msa	na	152
YKL165C	MCD4	3	unassigned	na	384
YKL165C-A	YKL165C-A	1	unassigned	na	4
YKL166C	TPK3	1	unassigned	na	78
YKL166C	TPK3	2	psiblast	1fotA_	112
YKL166C	TPK3	3	psiblast	1fotA_	208
YKL167C	MRP49	1	unassigned	na	137
YKL168C	KKQ8	1	fold_recogniti	1ldvA	223
YKL168C	KKQ8	2	unassigned	na	146
YKL168C	KKQ8	3	psiblast	1a06__	159
YKL168C	KKQ8	4	psiblast	1a06__	206
YKL169C	YKL169C	1	unassigned	na	127
YKL170W	MRPL38	1	psiblast	487dM_	138
YKL171W	YKL171W	1	unassigned	na	401
YKL171W	YKL171W	2	psiblast	1a06__	172
YKL171W	YKL171W	3	psiblast	1a06__	208
YKL171W	YKL171W	4	msa	na	147
YKL172W	EBP2	1	fold_recogniti	1l8mA	125

YKL172W	EBP2	2	fold_recogniti	1l8mA	73
YKL172W	EBP2	3	fold_recogniti	1l8mA	79
YKL172W	EBP2	4	fold_recogniti	1l8mA	70
YKL172W	EBP2	5	unassigned	na	80
YKL173W	SNU114	1	unassigned	na	113
YKL173W	SNU114	2	psiblast	1efgA_	330
YKL173W	SNU114	3	psiblast	1efgA_	146
YKL173W	SNU114	4	psiblast	1efgA_	220
YKL173W	SNU114	5	psiblast	1efgA_	199
YKL174C	YKL174C	1	pfam	PF00324	535
YKL174C	YKL174C	2	unassigned	na	83
YKL175W	ZRT3	1	fold_recogniti	1k83A	312
YKL175W	ZRT3	2	pfam	PF02535	191
YKL176C	LST4	1	msa	na	322
YKL176C	LST4	2	unassigned	na	191
YKL176C	LST4	3	fold_recogniti	1f1kA	315
YKL177W	YKL177W	1	unassigned	na	112
YKL178C	STE3	1	pfam	PF02076	332
YKL178C	STE3	2	unassigned	na	138
YKL179C	COY1	1	psiblast	1i84S_	359
YKL179C	COY1	2	msa	na	320
YKL180W	RPL17A	1	psiblast	1k5yN_	184
YKL181W	PRS1	1	psiblast	1dkrA_	167
YKL181W	PRS1	2	psiblast	1dkrA_	147
YKL181W	PRS1	3	psiblast	1ao0A_	113
YKL182W	FAS1	1	unassigned	na	138
YKL182W	FAS1	2	msa	na	414
YKL182W	FAS1	3	unassigned	na	93
YKL182W	FAS1	4	psiblast	1eepA_	194
YKL182W	FAS1	5	unassigned	na	437
YKL182W	FAS1	6	psiblast	1b3nA_	370
YKL182W	FAS1	7	unassigned	na	67
YKL182W	FAS1	8	psiblast	1mla__	338
YKL183W	LOT5	1	unassigned	na	306
YKL184W	SPE1	1	psiblast	7odcA_	104
YKL184W	SPE1	2	psiblast	7odcA_	232
YKL184W	SPE1	3	psiblast	7odcA_	130
YKL185W	ASH1	1	unassigned	na	236
YKL185W	ASH1	2	msa	na	239
YKL185W	ASH1	3	psiblast	4gatA_	113
YKL186C	MTR2	1	unassigned	na	184
YKL187C	YKL187C	1	unassigned	na	178
YKL187C	YKL187C	2	msa	na	189
YKL187C	YKL187C	3	unassigned	na	74
YKL187C	YKL187C	4	msa	na	248
YKL187C	YKL187C	5	unassigned	na	61
YKL188C	PXA2	1	unassigned	na	85
YKL188C	PXA2	2	psiblast	1jsqA_	342

YKL188C	PXA2	3	psiblast	1jsqA_	373
YKL188C	PXA2	4	unassigned	na	53
YKL189W	HYM1	1	fold_recogniti	1ee5A	122
YKL189W	HYM1	2	fold_recogniti	1ee5A	122
YKL189W	HYM1	3	fold_recogniti	1ee5A	155
YKL190W	CNB1	1	psiblast	1auiB_	86
YKL190W	CNB1	2	psiblast	1auiB_	89
YKL191W	DPH2	1	pfam	PF01866	423
YKL191W	DPH2	2	unassigned	na	111
YKL192C	ACP1	1	psiblast	1acp__	125
YKL193C	SDS22	1	psiblast	1k5dC_	111
YKL193C	SDS22	2	psiblast	1h6uA_	227
YKL194C	MST1	1	psiblast	1qf6A_	340
YKL194C	MST1	2	psiblast	1qf6A_	122
YKL195W	FMP15	1	msa	na	427
YKL196C	YKT6	1	psiblast	1h8mA_	137
YKL196C	YKT6	2	psiblast	1gl2A_	63
YKL197C	PEX1	1	fold_recogniti	1cz4A	199
YKL197C	PEX1	2	psiblast	1e32A_	118
YKL197C	PEX1	3	psiblast	1e32A_	97
YKL197C	PEX1	4	psiblast	1e32A_	188
YKL197C	PEX1	5	psiblast	1e32A_	87
YKL197C	PEX1	6	psiblast	1g4aE_	132
YKL197C	PEX1	7	fold_recogniti	1hqcA	149
YKL197C	PEX1	8	unassigned	na	73
YKL198C	PTK1	1	unassigned	na	159
YKL198C	PTK1	2	psiblast	1giiA_	112
YKL198C	PTK1	3	psiblast	1giiA_	128
YKL199C	3753	1	psiblast	1a06__	159
YKL199C	3753	2	msa	na	120
YKL201C	MNN4	1	msa	na	164
YKL201C	MNN4	2	unassigned	na	93
YKL201C	MNN4	3	msa	na	432
YKL201C	MNN4	4	psiblast	1i84S_	433
YKL201C	MNN4	5	unassigned	na	56
YKL202W	YKL202W	1	unassigned	na	193
YKL203C	TOR2	1	unassigned	na	75
YKL203C	TOR2	2	fold_recogniti	1b3uA	334
YKL203C	TOR2	3	unassigned	na	154
YKL203C	TOR2	4	msa	na	451
YKL203C	TOR2	5	unassigned	na	80
YKL203C	TOR2	6	msa	na	136
YKL203C	TOR2	7	unassigned	na	79
YKL203C	TOR2	8	msa	na	292
YKL203C	TOR2	9	msa	na	339
YKL203C	TOR2	10	psiblast	1aueA_	143
YKL203C	TOR2	11	msa	na	390
YKL204W	EAP1	1	unassigned	na	178

YKL204W	EAP1	2	fold_recogniti	1ei3A	314
YKL204W	EAP1	3	fold_recogniti	1ldvA	140
YKL205W	LOS1	1	msa	na	434
YKL205W	LOS1	2	unassigned	na	82
YKL205W	LOS1	3	msa	na	584
YKL206C	YKL206C	1	unassigned	na	267
YKL207W	YKL207W	1	unassigned	na	132
YKL207W	YKL207W	2	msa	na	128
YKL208W	CBT1	1	unassigned	na	271
YKL209C	STE6	1	fold_recogniti	1i84S	120
YKL209C	STE6	2	fold_recogniti	1i84S	223
YKL209C	STE6	3	psiblast	1l2tA_	296
YKL209C	STE6	4	unassigned	na	56
YKL209C	STE6	5	psiblast	1jsqA_	305
YKL209C	STE6	6	psiblast	1jsqA_	290
YKL210W	UBA1	1	psiblast	1jw9B_	306
YKL210W	UBA1	2	unassigned	na	102
YKL210W	UBA1	3	fold_recogniti	1eq2A	212
YKL210W	UBA1	4	msa	na	130
YKL210W	UBA1	5	unassigned	na	63
YKL210W	UBA1	6	msa	na	211
YKL211C	TRP3	1	msa	na	252
YKL211C	TRP3	2	psiblast	1igs__	232
YKL212W	SAC1	1	pfam	PF02383	375
YKL212W	SAC1	2	msa	na	248
YKL213C	DOA1	1	psiblast	1erjA_	89
YKL213C	DOA1	2	psiblast	1gxrA_	304
YKL213C	DOA1	3	psiblast	1k8kC_	98
YKL213C	DOA1	4	unassigned	na	224
YKL214C	YRA2	1	psiblast	1cvjA_	203
YKL215C	YKL215C	1	pfam	PF01968	664
YKL215C	YKL215C	2	unassigned	na	77
YKL215C	YKL215C	3	pfam	PF02538	545
YKL216W	URA1	1	psiblast	1dorA_	314
YKL217W	JEN1	1	unassigned	na	86
YKL217W	JEN1	2	psiblast	1ja5A_	430
YKL217W	JEN1	3	psiblast	1ja5A_	100
YKL218C	YKL218C	1	psiblast	1tdj__	131
YKL218C	YKL218C	2	psiblast	1tdj__	195
YKL219W	COS9	1	msa	na	89
YKL219W	COS9	2	pfam	PF00674	110
YKL219W	COS9	3	unassigned	na	73
YKL219W	COS9	4	pfam	PF00674	135
YKL220C	FRE2	1	msa	na	218
YKL220C	FRE2	2	msa	na	126
YKL220C	FRE2	3	unassigned	na	61
YKL220C	FRE2	4	psiblast	1cnf__	123
YKL220C	FRE2	5	psiblast	1cnf__	183

YKL221W	MCH2	1	unassigned	na	76
YKL221W	MCH2	2	psiblast	1ja5A_	397
YKL222C	YKL222C	1	psiblast	1hwtD_	95
YKL222C	YKL222C	2	unassigned	na	113
YKL222C	YKL222C	3	msa	na	244
YKL222C	YKL222C	4	unassigned	na	66
YKL222C	YKL222C	5	msa	na	187
YKL223W	YKL223W	1	unassigned	na	110
YKL224C	YKL224C	1	pfam	PF00660	123
YKL225W	YKL225W	1	unassigned	na	115
YKR001C	VPS1	1	psiblast	1jwyB_	356
YKR001C	VPS1	2	pfam	PF01031	227
YKR001C	VPS1	3	pfam	PF02212	121
YKR002W	PAP1	1	psiblast	1fa0A_	201
YKR002W	PAP1	2	psiblast	1fa0A_	367
YKR003W	OSH6	1	pfam	PF01237	448
YKR004C	ECM9	1	unassigned	na	292
YKR005C	YKR005C	1	fold_recogniti	1l2qA	334
YKR005C	YKR005C	2	unassigned	na	113
YKR006C	MRPL13	1	unassigned	na	275
YKR007W	MEH1	1	unassigned	na	184
YKR008W	RSC4	1	pfam	PF00439	163
YKR008W	RSC4	2	psiblast	1e6iA_	175
YKR008W	RSC4	3	msa	na	120
YKR008W	RSC4	4	unassigned	na	167
YKR009C	FOX2	1	psiblast	1cwuA_	258
YKR009C	FOX2	2	unassigned	na	56
YKR009C	FOX2	3	psiblast	1edoA_	282
YKR009C	FOX2	4	msa	na	103
YKR009C	FOX2	5	unassigned	na	58
YKR009C	FOX2	6	msa	na	143
YKR010C	TOF2	1	msa	na	241
YKR010C	TOF2	2	unassigned	na	530
YKR011C	YKR011C	1	unassigned	na	131
YKR011C	YKR011C	2	msa	na	222
YKR012C	YKR012C	1	unassigned	na	125
YKR013W	PRY2	1	fold_recogniti	1k83A	189
YKR013W	PRY2	2	psiblast	1cfe__	140
YKR014C	YPT52	1	psiblast	1huqA_	234
YKR015C	YKR015C	1	msa	na	111
YKR015C	YKR015C	2	unassigned	na	163
YKR015C	YKR015C	3	msa	na	192
YKR015C	YKR015C	4	unassigned	na	102
YKR016W	FMP13	1	psiblast	1i84S_	377
YKR016W	FMP13	2	msa	na	163
YKR017C	YKR017C	1	unassigned	na	154
YKR017C	YKR017C	2	psiblast	1rmd__	139
YKR017C	YKR017C	3	unassigned	na	93

YKR017C	YKR017C	4	msa	na	165
YKR018C	YKR018C	1	msa	na	183
YKR018C	YKR018C	2	unassigned	na	180
YKR018C	YKR018C	3	fold_recogniti	1hz4A	93
YKR018C	YKR018C	4	fold_recogniti	1hz4A	120
YKR018C	YKR018C	5	fold_recogniti	1hz4A	149
YKR019C	IRS4	1	unassigned	na	474
YKR019C	IRS4	2	psiblast	1c07A_	141
YKR020W	VPS51	1	unassigned	na	164
YKR021W	YKR021W	1	unassigned	na	146
YKR021W	YKR021W	2	psiblast	1jsyA_	232
YKR021W	YKR021W	3	psiblast	1jsyA_	194
YKR021W	YKR021W	4	msa	na	157
YKR021W	YKR021W	5	unassigned	na	186
YKR022C	YKR022C	1	unassigned	na	322
YKR023W	YKR023W	1	msa	na	118
YKR023W	YKR023W	2	msa	na	227
YKR023W	YKR023W	3	unassigned	na	185
YKR024C	DBP7	1	unassigned	na	101
YKR024C	DBP7	2	psiblast	1hv8A_	284
YKR024C	DBP7	3	psiblast	1hv8A_	196
YKR024C	DBP7	4	psiblast	1d2mA_	161
YKR025W	RPC37	1	msa	na	282
YKR026C	GCN3	1	fold_recogniti	2ffhA	234
YKR026C	GCN3	2	fold_recogniti	1m0sA	71
YKR027W	FMP50	1	msa	na	419
YKR027W	FMP50	2	unassigned	na	93
YKR027W	FMP50	3	msa	na	253
YKR028W	SAP190	1	unassigned	na	88
YKR028W	SAP190	2	msa	na	489
YKR028W	SAP190	3	msa	na	193
YKR028W	SAP190	4	msa	na	233
YKR028W	SAP190	5	unassigned	na	95
YKR029C	SET3	1	unassigned	na	110
YKR029C	SET3	2	psiblast	1fp0A_	105
YKR029C	SET3	3	msa	na	283
YKR029C	SET3	4	unassigned	na	253
YKR030W	GMH1	1	unassigned	na	273
YKR031C	SPO14	1	unassigned	na	178
YKR031C	SPO14	2	msa	na	208
YKR031C	SPO14	3	unassigned	na	286
YKR031C	SPO14	4	psiblast	1f0iA_	532
YKR031C	SPO14	5	unassigned	na	479
YKR032W	YKR032W	1	unassigned	na	104
YKR033C	YKR033C	1	unassigned	na	141
YKR034W	DAL80	1	psiblast	4gatA_	95
YKR034W	DAL80	2	unassigned	na	174
YKR035W-A	DID2	1	fold_recogniti	1cunA	80

YKR035W-A	DID2	2	fold_recogniti	1cunA	124
YKR036C	CAF4	1	unassigned	na	268
YKR036C	CAF4	2	psiblast	1gg2B_	391
YKR037C	SPC34	1	unassigned	na	295
YKR038C	KAE1	1	fold_recogniti	1huxA	211
YKR038C	KAE1	2	fold_recogniti	1huxA	210
YKR039W	GAP1	1	unassigned	na	70
YKR039W	GAP1	2	pfam	PF00324	532
YKR040C	YKR040C	1	unassigned	na	167
YKR041W	YKR041W	1	msa	na	167
YKR041W	YKR041W	2	unassigned	na	83
YKR042W	UTH1	1	unassigned	na	75
YKR042W	UTH1	2	fold_recogniti	1jx5A	375
YKR043C	YKR043C	1	psiblast	1bq3D_	271
YKR044W	UIP5	1	psiblast	1gv9A_	367
YKR044W	UIP5	2	unassigned	na	76
YKR045C	YKR045C	1	unassigned	na	191
YKR046C	PET10	1	unassigned	na	283
YKR047W	YKR047W	1	unassigned	na	101
YKR048C	NAP1	1	unassigned	na	81
YKR048C	NAP1	2	pfam	PF00956	336
YKR049C	FMP46	1	unassigned	na	133
YKR050W	TRK2	1	unassigned	na	95
YKR050W	TRK2	2	msa	na	259
YKR050W	TRK2	3	pfam	PF02386	535
YKR051W	YKR051W	1	pfam	PF03619	317
YKR051W	YKR051W	2	unassigned	na	101
YKR052C	MRS4	1	fold_recogniti	1is2A	304
YKR053C	YSR3	1	unassigned	na	89
YKR053C	YSR3	2	psiblast	1d2tA_	177
YKR053C	YSR3	3	unassigned	na	138
YKR055W	RHO4	1	unassigned	na	62
YKR055W	RHO4	2	psiblast	1lb1B_	229
YKR056W	TRM2	1	unassigned	na	156
YKR056W	TRM2	2	pfam	PF01938	84
YKR056W	TRM2	3	msa	na	117
YKR056W	TRM2	4	unassigned	na	74
YKR056W	TRM2	5	psiblast	1g6q1_	186
YKR057W	RPS21A	1	pfam	PF01249	87
YKR058W	GLG1	1	psiblast	1ll0A_	205
YKR058W	GLG1	2	fold_recogniti	1l8mA	71
YKR058W	GLG1	3	fold_recogniti	1l8mA	78
YKR058W	GLG1	4	fold_recogniti	1l8mA	69
YKR058W	GLG1	5	fold_recogniti	1l8mA	57
YKR060W	UTP30	1	psiblast	1eg0N_	179
YKR060W	UTP30	2	psiblast	1eg0N_	95
YKR061W	KTR2	1	pfam	PF01793	425
YKR062W	TFA2	1	unassigned	na	96

YKR062W	TFA2	2	psiblast	1d8jA_	91
YKR062W	TFA2	3	unassigned	na	141
YKR063C	LAS1	1	fold_recogniti	1n1bA	219
YKR063C	LAS1	2	fold_recogniti	1n1bA	142
YKR063C	LAS1	3	unassigned	na	141
YKR064W	YKR064W	1	psiblast	1qp9A_	118
YKR064W	YKR064W	2	unassigned	na	122
YKR064W	YKR064W	3	msa	na	267
YKR064W	YKR064W	4	unassigned	na	356
YKR065C	FMP18	1	unassigned	na	197
YKR066C	CCP1	1	psiblast	2cyp__	243
YKR066C	CCP1	2	psiblast	2cyp__	118
YKR067W	GPT2	1	fold_recogniti	1k30A	398
YKR067W	GPT2	2	unassigned	na	231
YKR067W	GPT2	3	msa	na	114
YKR068C	BET3	1	unassigned	na	193
YKR069W	MET1	1	psiblast	1kyqA_	216
YKR069W	MET1	2	unassigned	na	105
YKR069W	MET1	3	psiblast	2cbf__	120
YKR069W	MET1	4	psiblast	2cbf__	152
YKR070W	YKR070W	1	fold_recogniti	1mhsA	181
YKR070W	YKR070W	2	psiblast	1qq6A_	171
YKR071C	DRE2	1	unassigned	na	183
YKR071C	DRE2	2	msa	na	165
YKR072C	SIS2	1	msa	na	283
YKR072C	SIS2	2	psiblast	1e20A_	279
YKR073C	YKR073C	1	unassigned	na	106
YKR074W	YKR074W	1	unassigned	na	155
YKR075C	YKR075C	1	msa	na	197
YKR075C	YKR075C	2	unassigned	na	110
YKR076W	ECM4	1	fold_recogniti	1eemA	198
YKR076W	ECM4	2	fold_recogniti	1eemA	172
YKR077W	YKR077W	1	unassigned	na	363
YKR078W	YKR078W	1	fold_recogniti	1h6hA	253
YKR078W	YKR078W	2	msa	na	102
YKR078W	YKR078W	3	fold_recogniti	1i84S	230
YKR079C	YKR079C	1	fold_recogniti	1qh5A	204
YKR079C	YKR079C	2	fold_recogniti	1qh5A	104
YKR079C	YKR079C	3	unassigned	na	148
YKR079C	YKR079C	4	psiblast	1e5dA_	183
YKR079C	YKR079C	5	msa	na	199
YKR080W	MTD1	1	psiblast	1edzA_	190
YKR080W	MTD1	2	psiblast	1edzA_	130
YKR081C	RPF2	1	pfam	PF03679	262
YKR081C	RPF2	2	unassigned	na	82
YKR082W	NUP133	1	unassigned	na	637
YKR082W	NUP133	2	msa	na	437
YKR082W	NUP133	3	unassigned	na	83

YKR083C	DAD2	1	unassigned	na	133
YKR084C	HBS1	1	msa	na	152
YKR084C	HBS1	2	psiblast	1jnyA_	241
YKR084C	HBS1	3	psiblast	1jnyA_	107
YKR084C	HBS1	4	psiblast	1jnyA_	111
YKR085C	MRPL20	1	unassigned	na	195
YKR086W	PRP16	1	msa	na	300
YKR086W	PRP16	2	psiblast	1fuuA_	447
YKR086W	PRP16	3	unassigned	na	73
YKR086W	PRP16	4	msa	na	251
YKR087C	OMA1	1	fold_recogniti	1qu7A	314
YKR088C	TVP38	1	fold_recogniti	1hu3A	148
YKR088C	TVP38	2	msa	na	189
YKR089C	STC1	1	unassigned	na	112
YKR089C	STC1	2	msa	na	147
YKR089C	STC1	3	pfam	PF01734	246
YKR089C	STC1	4	msa	na	267
YKR089C	STC1	5	unassigned	na	138
YKR090W	PXL1	1	fold_recogniti	1m2vB	191
YKR090W	PXL1	2	msa	na	228
YKR090W	PXL1	3	fold_recogniti	1k83A	132
YKR090W	PXL1	4	psiblast	1b8tA_	66
YKR090W	PXL1	5	psiblast	1b8tA_	89
YKR091W	SRL3	1	unassigned	na	152
YKR092C	SRP40	1	psiblast	1deqA_	198
YKR092C	SRP40	2	fold_recogniti	1ldvA	208
YKR093W	PTR2	1	psiblast	1ja5A_	516
YKR093W	PTR2	2	psiblast	1ja5A_	85
YKR095W	MLP1	1	psiblast	1c1gA_	103
YKR095W	MLP1	2	psiblast	1c1gA_	224
YKR095W	MLP1	3	msa	na	123
YKR095W	MLP1	4	psiblast	1i84S_	152
YKR095W	MLP1	5	psiblast	1i84S_	299
YKR095W	MLP1	6	msa	na	520
YKR095W	MLP1	7	unassigned	na	86
YKR095W	MLP1	8	fold_recogniti	1k83A	368
YKR096W	YKR096W	1	unassigned	na	1019
YKR096W	YKR096W	2	msa	na	176
YKR097W	PCK1	1	psiblast	1ii2A_	283
YKR097W	PCK1	2	psiblast	1ii2A_	126
YKR097W	PCK1	3	psiblast	1ii2A_	140
YKR098C	UBP11	1	unassigned	na	54
YKR098C	UBP11	2	msa	na	192
YKR098C	UBP11	3	pfam	PF00442	136
YKR098C	UBP11	4	msa	na	140
YKR098C	UBP11	5	unassigned	na	100
YKR098C	UBP11	6	pfam	PF00443	95
YKR099W	BAS1	1	psiblast	1h88C_	113

YKR099W	BAS1	2	psiblast	1h88C_	51
YKR099W	BAS1	3	psiblast	1h88C_	62
YKR099W	BAS1	4	msa	na	233
YKR099W	BAS1	5	unassigned	na	352
YKR100C	SKG1	1	msa	na	182
YKR100C	SKG1	2	unassigned	na	59
YKR101W	SIR1	1	unassigned	na	678
YKR102W	FLO10	1	msa	na	345
YKR102W	FLO10	2	unassigned	na	217
YKR102W	FLO10	3	msa	na	274
YKR102W	FLO10	4	msa	na	193
YKR102W	FLO10	5	msa	na	140
YKR103W	NFT1	1	msa	na	268
YKR103W	NFT1	2	psiblast	1jsqA_	334
YKR103W	NFT1	3	psiblast	1jsqA_	307
YKR103W	NFT1	4	unassigned	na	88
YKR103W	NFT1	5	fold_recogniti	1qu7A	221
YKR104W	NFT1	1	psiblast	1jsqA_	306
YKR105C	YKR105C	1	psiblast	1lviA_	242
YKR105C	YKR105C	2	unassigned	na	72
YKR105C	YKR105C	3	msa	na	212
YKR105C	YKR105C	4	unassigned	na	56
YKR106W	YKR106W	1	psiblast	1ja5A_	406
YKR106W	YKR106W	2	msa	na	209
YLL001W	DNM1	1	psiblast	1jwyB_	353
YLL001W	DNM1	2	pfam	PF01031	190
YLL001W	DNM1	3	psiblast	1dynA_	117
YLL001W	DNM1	4	pfam	PF02212	97
YLL002W	RTT109	1	unassigned	na	168
YLL002W	RTT109	2	msa	na	268
YLL003W	SFI1	1	unassigned	na	96
YLL003W	SFI1	2	fold_recogniti	1qgkA	164
YLL003W	SFI1	3	fold_recogniti	1qgkA	194
YLL003W	SFI1	4	fold_recogniti	1qgkA	92
YLL003W	SFI1	5	fold_recogniti	1qgkA	109
YLL003W	SFI1	6	fold_recogniti	1qgkA	158
YLL003W	SFI1	7	unassigned	na	133
YLL004W	ORC3	1	unassigned	na	122
YLL004W	ORC3	2	fold_recogniti	1n1bA	156
YLL004W	ORC3	3	fold_recogniti	1n1bA	228
YLL004W	ORC3	4	unassigned	na	110
YLL005C	SPO75	1	msa	na	235
YLL005C	SPO75	2	unassigned	na	162
YLL005C	SPO75	3	pfam	PF02714	471
YLL006W	MMM1	1	unassigned	na	84
YLL006W	MMM1	2	msa	na	342
YLL007C	YLL007C	1	unassigned	na	115
YLL007C	YLL007C	2	fold_recogniti	1qoyA	304

YLL007C	YLL007C	3	fold_recogniti	1qoyA	52
YLL007C	YLL007C	4	unassigned	na	194
YLL008W	DRS1	1	fold_recogniti	1l8mA	68
YLL008W	DRS1	2	fold_recogniti	1l8mA	79
YLL008W	DRS1	3	fold_recogniti	1l8mA	84
YLL008W	DRS1	4	psiblast	1hv8A_	213
YLL008W	DRS1	5	psiblast	1hv8A_	154
YLL008W	DRS1	6	psiblast	1d2mA_	154
YLL009C	COX17	1	unassigned	na	69
YLL010C	PSR1	1	msa	na	225
YLL010C	PSR1	2	pfam	PF03031	202
YLL011W	SOF1	1	fold_recogniti	1scgB	108
YLL011W	SOF1	2	psiblast	1erjA_	381
YLL012W	YLL012W	1	unassigned	na	138
YLL012W	YLL012W	2	psiblast	1hlgA_	435
YLL013C	PUF3	1	msa	na	147
YLL013C	PUF3	2	fold_recogniti	1k83A	366
YLL013C	PUF3	3	psiblast	1ib2A_	134
YLL013C	PUF3	4	psiblast	1ib2A_	99
YLL013C	PUF3	5	psiblast	1ib2A_	133
YLL014W	YLL014W	1	unassigned	na	108
YLL015W	BPT1	1	msa	na	241
YLL015W	BPT1	2	fold_recogniti	1i84S	384
YLL015W	BPT1	3	psiblast	1nbd__	267
YLL015W	BPT1	4	unassigned	na	63
YLL015W	BPT1	5	psiblast	1jsqA_	305
YLL015W	BPT1	6	psiblast	1jsqA_	299
YLL016W	SDC25	1	msa	na	158
YLL016W	SDC25	2	unassigned	na	368
YLL016W	SDC25	3	psiblast	1bkdS_	220
YLL016W	SDC25	4	psiblast	1bkdS_	302
YLL017W	SDC25	1	psiblast	1g83A_	103
YLL018C	DPS1	1	psiblast	1asyA_	210
YLL018C	DPS1	2	psiblast	1asyA_	347
YLL018C-A	COX19	1	unassigned	na	98
YLL019C	KNS1	1	msa	na	156
YLL019C	KNS1	2	unassigned	na	57
YLL019C	KNS1	3	psiblast	1bmkA_	210
YLL019C	KNS1	4	psiblast	1bmkA_	254
YLL019C	KNS1	5	fold_recogniti	1i09A	60
YLL020C	YLL020C	1	unassigned	na	101
YLL021W	SPA2	1	msa	na	284
YLL021W	SPA2	2	unassigned	na	89
YLL021W	SPA2	3	msa	na	586
YLL021W	SPA2	4	msa	na	393
YLL021W	SPA2	5	unassigned	na	114
YLL022C	HIF1	1	fold_recogniti	1gt8A	385
YLL023C	YLL023C	1	unassigned	na	279

YLL024C	SSA2	1	psiblast	1hpm__	191
YLL024C	SSA2	2	psiblast	1hpm__	116
YLL024C	SSA2	3	psiblast	1hpm__	75
YLL024C	SSA2	4	psiblast	1ckrA_	158
YLL024C	SSA2	5	psiblast	1i84S_	99
YLL025W	YLL025W	1	pfam	PF00660	124
YLL026W	HSP104	1	msa	na	85
YLL026W	HSP104	2	pfam	PF02861	76
YLL026W	HSP104	3	psiblast	1jbkA_	192
YLL026W	HSP104	4	fold_recogniti	1ksfX	83
YLL026W	HSP104	5	psiblast	1g4aE_	414
YLL026W	HSP104	6	unassigned	na	58
YLL027W	ISA1	1	unassigned	na	110
YLL027W	ISA1	2	pfam	PF01521	140
YLL028W	TPO1	1	unassigned	na	119
YLL028W	TPO1	2	psiblast	1ja5A_	426
YLL028W	TPO1	3	psiblast	1ja5A_	41
YLL029W	YLL029W	1	unassigned	na	83
YLL029W	YLL029W	2	fold_recogniti	1chmA	194
YLL029W	YLL029W	3	psiblast	1jaw__	174
YLL029W	YLL029W	4	psiblast	1jaw__	298
YLL030C	YLL030C	1	unassigned	na	113
YLL031C	GPI13	1	unassigned	na	75
YLL031C	GPI13	2	psiblast	1ejjA_	338
YLL031C	GPI13	3	msa	na	143
YLL031C	GPI13	4	unassigned	na	64
YLL031C	GPI13	5	msa	na	397
YLL032C	YLL032C	1	unassigned	na	141
YLL032C	YLL032C	2	msa	na	217
YLL032C	YLL032C	3	psiblast	1e3pA_	196
YLL032C	YLL032C	4	psiblast	1j4wA_	93
YLL032C	YLL032C	5	msa	na	98
YLL032C	YLL032C	6	unassigned	na	80
YLL033W	GON2	1	unassigned	na	230
YLL034C	RIX7	1	psiblast	1e32A_	105
YLL034C	RIX7	2	psiblast	1e32A_	96
YLL034C	RIX7	3	psiblast	1e32A_	176
YLL034C	RIX7	4	psiblast	1e32A_	97
YLL034C	RIX7	5	unassigned	na	90
YLL034C	RIX7	6	pfam	PF00004	218
YLL034C	RIX7	7	unassigned	na	55
YLL035W	GRC3	1	unassigned	na	196
YLL035W	GRC3	2	fold_recogniti	1jsqA	205
YLL035W	GRC3	3	msa	na	231
YLL036C	PRP19	1	psiblast	1bf5A_	124
YLL036C	PRP19	2	psiblast	1gg2B_	379
YLL037W	YLL037W	1	unassigned	na	126
YLL038C	ENT4	1	psiblast	1eyhA_	162

YLL038C	ENT4	2	unassigned	na	85
YLL039C	UBI4	1	psiblast	1nddA_	76
YLL039C	UBI4	2	fold_recogniti	1nddA	74
YLL039C	UBI4	3	psiblast	1gjzA_	65
YLL039C	UBI4	4	psiblast	1aarA_	89
YLL039C	UBI4	5	fold_recogniti	1j8cA	77
YLL040C	VPS13	1	msa	na	394
YLL040C	VPS13	2	unassigned	na	79
YLL040C	VPS13	3	msa	na	390
YLL040C	VPS13	4	unassigned	na	364
YLL040C	VPS13	5	msa	na	126
YLL040C	VPS13	6	unassigned	na	386
YLL040C	VPS13	7	msa	na	125
YLL040C	VPS13	8	unassigned	na	305
YLL040C	VPS13	9	msa	na	109
YLL040C	VPS13	10	msa	na	263
YLL040C	VPS13	11	unassigned	na	78
YLL040C	VPS13	12	msa	na	402
YLL040C	VPS13	13	unassigned	na	123
YLL041C	SDH2	1	psiblast	1kf6B_	136
YLL041C	SDH2	2	psiblast	1kf6B_	130
YLL042C	ATG10	1	unassigned	na	167
YLL043W	FPS1	1	unassigned	na	228
YLL043W	FPS1	2	psiblast	1fx8A_	176
YLL043W	FPS1	3	psiblast	1fx8A_	153
YLL043W	FPS1	4	unassigned	na	112
YLL045C	RPL8B	1	msa	na	96
YLL045C	RPL8B	2	psiblast	1k5yG_	160
YLL046C	RNP1	1	psiblast	1l3kA_	119
YLL046C	RNP1	2	psiblast	1l3kA_	130
YLL048C	YBT1	1	msa	na	306
YLL048C	YBT1	2	pfam	PF00664	372
YLL048C	YBT1	3	psiblast	1nbd__	278
YLL048C	YBT1	4	unassigned	na	78
YLL048C	YBT1	5	psiblast	1jsqA_	306
YLL048C	YBT1	6	psiblast	1jsqA_	321
YLL049W	YLL049W	1	unassigned	na	179
YLL050C	COF1	1	psiblast	1cfyA_	143
YLL051C	FRE6	1	unassigned	na	272
YLL051C	FRE6	2	msa	na	150
YLL051C	FRE6	3	psiblast	1cnf__	118
YLL051C	FRE6	4	psiblast	1cnf__	172
YLL052C	AQY2	1	unassigned	na	149
YLL053C	YLL053C	1	psiblast	1j4nA_	152
YLL054C	YLL054C	1	psiblast	1pyiA_	150
YLL054C	YLL054C	2	unassigned	na	60
YLL054C	YLL054C	3	fold_recogniti	1n1bA	59
YLL054C	YLL054C	4	fold_recogniti	1n1bA	436

YLL054C	YLL054C	5	unassigned	na	64
YLL055W	YLL055W	1	psiblast	1ja5A_	531
YLL056C	YLL056C	1	psiblast	1bxkA_	227
YLL056C	YLL056C	2	psiblast	1bxkA_	71
YLL057C	JLP1	1	unassigned	na	91
YLL057C	JLP1	2	psiblast	1gy9A_	203
YLL057C	JLP1	3	psiblast	1gy9A_	118
YLL058W	YLL058W	1	unassigned	na	95
YLL058W	YLL058W	2	psiblast	1gc0A_	338
YLL058W	YLL058W	3	psiblast	1gc0A_	142
YLL059C	YLL059C	1	unassigned	na	168
YLL060C	GTT2	1	psiblast	1byeA_	99
YLL060C	GTT2	2	psiblast	1byeA_	134
YLL061W	MMP1	1	pfam	PF00324	583
YLL062C	MHT1	1	fold_recogniti	1dhpA	324
YLL063C	AYT1	1	fold_recogniti	1l5aA	272
YLL063C	AYT1	2	fold_recogniti	1l5aA	202
YLL064C	YLL064C	1	pfam	PF00660	120
YLL065W	YLL065W	1	unassigned	na	116
YLL066C	YLL066C	1	msa	na	144
YLL066C	YLL066C	2	psiblast	1fuuA_	499
YLL066C	YLL066C	3	unassigned	na	69
YLL066C	YLL066C	4	psiblast	1ezgA_	107
YLL066C	YLL066C	5	unassigned	na	386
YLL067C	YLL067C	1	msa	na	111
YLL067C	YLL067C	2	psiblast	1fuuA_	513
YLL067C	YLL067C	3	fold_recogniti	1m2vB	404
YLL067C	YLL067C	4	unassigned	na	177
YLR001C	YLR001C	1	pfam	PF02469	197
YLR001C	YLR001C	2	unassigned	na	85
YLR001C	YLR001C	3	msa	na	379
YLR001C	YLR001C	4	unassigned	na	140
YLR001C	YLR001C	5	msa	na	61
YLR002C	NOC3	1	unassigned	na	111
YLR002C	NOC3	2	fold_recogniti	1b3uA	152
YLR002C	NOC3	3	fold_recogniti	1b3uA	66
YLR002C	NOC3	4	fold_recogniti	1b3uA	141
YLR002C	NOC3	5	fold_recogniti	1b3uA	193
YLR003C	YLR003C	1	unassigned	na	291
YLR004C	YLR004C	1	psiblast	1ja5A_	523
YLR005W	SSL1	1	unassigned	na	116
YLR005W	SSL1	2	psiblast	1a8x__	229
YLR005W	SSL1	3	psiblast	1e53A_	116
YLR006C	SSK1	1	unassigned	na	183
YLR006C	SSK1	2	msa	na	131
YLR006C	SSK1	3	psiblast	1bxdA_	183
YLR006C	SSK1	4	psiblast	1kgsA_	215
YLR007W	NSE1	1	unassigned	na	107

YLR007W	NSE1	2	msa	na	97
YLR007W	NSE1	3	unassigned	na	132
YLR008C	PAM18	1	unassigned	na	92
YLR008C	PAM18	2	psiblast	1hdj__	76
YLR009W	RLP24	1	psiblast	1k5yS_	61
YLR009W	RLP24	2	msa	na	138
YLR010C	TEN1	1	unassigned	na	160
YLR011W	LOT6	1	psiblast	1qrdA_	191
YLR012C	YLR012C	1	unassigned	na	122
YLR013W	GAT3	1	psiblast	4gatA_	141
YLR014C	PPR1	1	psiblast	1pyiA_	133
YLR014C	PPR1	2	unassigned	na	139
YLR014C	PPR1	3	msa	na	346
YLR014C	PPR1	4	unassigned	na	286
YLR015W	BRE2	1	msa	na	275
YLR015W	BRE2	2	unassigned	na	230
YLR016C	YLR016C	1	unassigned	na	72
YLR016C	YLR016C	2	psiblast	1lgqA_	132
YLR017W	MEU1	1	psiblast	1cb0A_	337
YLR018C	POM34	1	msa	na	179
YLR018C	POM34	2	unassigned	na	120
YLR019W	PSR2	1	msa	na	188
YLR019W	PSR2	2	pfam	PF03031	209
YLR020C	YLR020C	1	unassigned	na	121
YLR020C	YLR020C	2	psiblast	1k8qA_	417
YLR021W	YLR021W	1	unassigned	na	179
YLR022C	YLR022C	1	pfam	PF01172	250
YLR023C	YLR023C	1	unassigned	na	174
YLR023C	YLR023C	2	pfam	PF03006	369
YLR024C	UBR2	1	unassigned	na	81
YLR024C	UBR2	2	pfam	PF02207	92
YLR024C	UBR2	3	unassigned	na	540
YLR024C	UBR2	4	msa	na	557
YLR024C	UBR2	5	unassigned	na	300
YLR024C	UBR2	6	msa	na	302
YLR025W	SNF7	1	psiblast	1i84S_	240
YLR026C	SED5	1	fold_recogniti	1mqsB	45
YLR026C	SED5	2	psiblast	1dn1B_	242
YLR026C	SED5	3	psiblast	1dn1B_	53
YLR027C	AAT2	1	psiblast	1yaaA_	152
YLR027C	AAT2	2	psiblast	1yaaA_	280
YLR028C	ADE16	1	psiblast	1g8mA_	197
YLR028C	ADE16	2	psiblast	1g8mA_	181
YLR028C	ADE16	3	psiblast	1g8mA_	213
YLR029C	RPL15A	1	psiblast	1k5yL_	204
YLR030W	YLR030W	1	unassigned	na	263
YLR031W	YLR031W	1	unassigned	na	186
YLR032W	RAD5	1	msa	na	378

YLR032W	RAD5	2	fold_recogniti	1gm5A	98
YLR032W	RAD5	3	fold_recogniti	1gm5A	343
YLR032W	RAD5	4	msa	na	102
YLR032W	RAD5	5	unassigned	na	54
YLR032W	RAD5	6	psiblast	1d9xA_	194
YLR033W	RSC58	1	unassigned	na	502
YLR034C	SMF3	1	pfam	PF01566	473
YLR035C	MLH2	1	psiblast	1ea6A_	221
YLR035C	MLH2	2	psiblast	1ea6A_	173
YLR035C	MLH2	3	msa	na	118
YLR035C	MLH2	4	unassigned	na	183
YLR036C	YLR036C	1	unassigned	na	203
YLR037C	DAN2	1	pfam	PF00660	124
YLR038C	COX12	1	psiblast	1occH_	83
YLR039C	RIC1	1	unassigned	na	1056
YLR040C	YLR040C	1	unassigned	na	224
YLR041W	YLR041W	1	unassigned	na	106
YLR042C	YLR042C	1	unassigned	na	161
YLR043C	TRX1	1	psiblast	1erv__	103
YLR044C	PDC1	1	psiblast	1qpbA_	188
YLR044C	PDC1	2	psiblast	1qpbA_	165
YLR044C	PDC1	3	psiblast	1qpbA_	210
YLR045C	STU2	1	psiblast	1b3uA_	155
YLR045C	STU2	2	psiblast	1b3uA_	175
YLR045C	STU2	3	psiblast	1b3uA_	231
YLR045C	STU2	4	msa	na	194
YLR045C	STU2	5	unassigned	na	133
YLR046C	YLR046C	1	msa	na	270
YLR047C	YLR047C	1	unassigned	na	142
YLR047C	YLR047C	2	msa	na	122
YLR047C	YLR047C	3	psiblast	1ib0A_	129
YLR047C	YLR047C	4	psiblast	1ib0A_	178
YLR047C	YLR047C	5	unassigned	na	115
YLR048W	RPS0B	1	psiblast	1k5xB_	252
YLR049C	YLR049C	1	unassigned	na	428
YLR050C	YLR050C	1	unassigned	na	161
YLR051C	YLR051C	1	msa	na	217
YLR052W	IES3	1	unassigned	na	250
YLR053C	YLR053C	1	unassigned	na	108
YLR054C	OSW2	1	unassigned	na	512
YLR055C	SPT8	1	fold_recogniti	1l8mA	66
YLR055C	SPT8	2	fold_recogniti	1l8mA	58
YLR055C	SPT8	3	psiblast	1gg2B_	367
YLR055C	SPT8	4	psiblast	1kb0A_	111
YLR056W	ERG3	1	unassigned	na	84
YLR056W	ERG3	2	pfam	PF01598	281
YLR057W	YLR057W	1	unassigned	na	157
YLR057W	YLR057W	2	psiblast	1fmiA_	291

YLR057W	YLR057W	3	unassigned	na	229
YLR057W	YLR057W	4	msa	na	172
YLR058C	SHM2	1	psiblast	1cj0A_	196
YLR058C	SHM2	2	psiblast	1cj0A_	273
YLR059C	REX2	1	psiblast	1fxxA_	269
YLR060W	FRS1	1	psiblast	1b70B_	293
YLR060W	FRS1	2	psiblast	1b70B_	82
YLR060W	FRS1	3	psiblast	1b70B_	220
YLR061W	RPL22A	1	pfam	PF01776	121
YLR062C	BUD28	1	unassigned	na	125
YLR063W	YLR063W	1	unassigned	na	365
YLR064W	YLR064W	1	unassigned	na	273
YLR065C	YLR065C	1	unassigned	na	181
YLR066W	SPC3	1	unassigned	na	184
YLR067C	PET309	1	msa	na	692
YLR067C	PET309	2	msa	na	132
YLR067C	PET309	3	msa	na	141
YLR068W	FYV7	1	unassigned	na	151
YLR069C	MEF1	1	psiblast	1dar__	352
YLR069C	MEF1	2	psiblast	1dar__	146
YLR069C	MEF1	3	psiblast	1dar__	263
YLR070C	XYL2	1	psiblast	1e3jA_	189
YLR070C	XYL2	2	psiblast	1e3jA_	167
YLR071C	RGR1	1	unassigned	na	224
YLR071C	RGR1	2	msa	na	219
YLR071C	RGR1	3	unassigned	na	639
YLR072W	YLR072W	1	unassigned	na	112
YLR072W	YLR072W	2	pfam	PF02893	163
YLR072W	YLR072W	3	unassigned	na	78
YLR072W	YLR072W	4	msa	na	235
YLR072W	YLR072W	5	unassigned	na	105
YLR073C	YLR073C	1	unassigned	na	200
YLR074C	BUD20	1	unassigned	na	166
YLR075W	RPL10	1	psiblast	1k5yI_	221
YLR076C	YLR076C	1	unassigned	na	140
YLR077W	FMP25	1	unassigned	na	158
YLR077W	FMP25	2	psiblast	1a12A_	425
YLR078C	BOS1	1	psiblast	1i84S_	244
YLR079W	SIC1	1	unassigned	na	284
YLR080W	EMP46	1	psiblast	1gv9A_	277
YLR080W	EMP46	2	psiblast	1i84S_	167
YLR081W	GAL2	1	psiblast	1ja5A_	475
YLR081W	GAL2	2	psiblast	1ja5A_	99
YLR082C	SRL2	1	unassigned	na	392
YLR083C	EMP70	1	pfam	PF02990	667
YLR084C	RAX2	1	unassigned	na	1220
YLR085C	ARP6	1	psiblast	1yagA_	176
YLR085C	ARP6	2	psiblast	1yagA_	262

YLR086W	SMC4	1	unassigned	na	135
YLR086W	SMC4	2	psiblast	1ii8A_	78
YLR086W	SMC4	3	psiblast	1i84S_	298
YLR086W	SMC4	4	psiblast	1cii__	144
YLR086W	SMC4	5	psiblast	1gxlA_	69
YLR086W	SMC4	6	psiblast	1gxlA_	72
YLR086W	SMC4	7	psiblast	1gxlA_	58
YLR086W	SMC4	8	psiblast	1c1gA_	64
YLR086W	SMC4	9	psiblast	1c1gA_	213
YLR086W	SMC4	10	psiblast	1jchA_	88
YLR086W	SMC4	11	psiblast	1e69A_	199
YLR087C	CSF1	1	unassigned	na	416
YLR087C	CSF1	2	msa	na	322
YLR087C	CSF1	3	unassigned	na	2220
YLR088W	GAA1	1	unassigned	na	309
YLR088W	GAA1	2	msa	na	193
YLR088W	GAA1	3	unassigned	na	112
YLR089C	ALT1	1	msa	na	162
YLR089C	ALT1	2	psiblast	1iaxA_	129
YLR089C	ALT1	3	psiblast	1iaxA_	301
YLR090W	XDJ1	1	psiblast	1bq0__	120
YLR090W	XDJ1	2	psiblast	1exkA_	118
YLR090W	XDJ1	3	psiblast	1c3gA_	161
YLR090W	XDJ1	4	unassigned	na	60
YLR091W	YLR091W	1	unassigned	na	293
YLR092W	SUL2	1	psiblast	1hynP_	105
YLR092W	SUL2	2	msa	na	126
YLR092W	SUL2	3	pfam	PF00916	353
YLR092W	SUL2	4	msa	na	309
YLR093C	NYV1	1	psiblast	1ifqA_	145
YLR093C	NYV1	2	psiblast	1sfcA_	106
YLR094C	GIS3	1	msa	na	275
YLR094C	GIS3	2	unassigned	na	227
YLR095C	IOC2	1	unassigned	na	285
YLR095C	IOC2	2	fold_recogniti	1iu4A	340
YLR095C	IOC2	3	unassigned	na	187
YLR096W	KIN2	1	unassigned	na	66
YLR096W	KIN2	2	psiblast	1a06__	167
YLR096W	KIN2	3	psiblast	1a06__	194
YLR096W	KIN2	4	psiblast	1ahr__	78
YLR096W	KIN2	5	psiblast	1ahr__	64
YLR096W	KIN2	6	fold_recogniti	1k83A	361
YLR096W	KIN2	7	fold_recogniti	1m2vB	53
YLR096W	KIN2	8	unassigned	na	164
YLR097C	HRT3	1	unassigned	na	73
YLR097C	HRT3	2	pfam	PF00646	116
YLR097C	HRT3	3	unassigned	na	155
YLR098C	CHA4	1	psiblast	1cld__	118

YLR098C	CHA4	2	unassigned	na	55
YLR098C	CHA4	3	fold_recogniti	1n1bA	304
YLR098C	CHA4	4	fold_recogniti	1n1bA	57
YLR098C	CHA4	5	fold_recogniti	1n1bA	114
YLR099C	ICT1	1	psiblast	1cqzA_	394
YLR099W-A	YLR099W-A	1	unassigned	na	87
YLR100W	ERG27	1	psiblast	1gcoA_	347
YLR101C	YLR101C	1	unassigned	na	131
YLR102C	APC9	1	unassigned	na	265
YLR103C	CDC45	1	pfam	PF02724	650
YLR104W	YLR104W	1	unassigned	na	131
YLR105C	SEN2	1	msa	na	155
YLR105C	SEN2	2	psiblast	1a79A_	105
YLR105C	SEN2	3	psiblast	1a79A_	117
YLR107W	REX3	1	unassigned	na	189
YLR107W	REX3	2	psiblast	1dpi__	215
YLR108C	YLR108C	1	pfam	PF02214	165
YLR108C	YLR108C	2	unassigned	na	320
YLR109W	AHP1	1	psiblast	1h4oA_	176
YLR110C	CCW12	1	unassigned	na	133
YLR111W	YLR111W	1	unassigned	na	110
YLR112W	YLR112W	1	unassigned	na	139
YLR113W	HOG1	1	psiblast	1bmkA_	166
YLR113W	HOG1	2	psiblast	1bmkA_	216
YLR113W	HOG1	3	unassigned	na	53
YLR114C	YLR114C	1	msa	na	169
YLR114C	YLR114C	2	unassigned	na	118
YLR114C	YLR114C	3	msa	na	477
YLR115W	CFT2	1	psiblast	1e5dA_	273
YLR115W	CFT2	2	msa	na	208
YLR115W	CFT2	3	msa	na	378
YLR116W	MSL5	1	msa	na	95
YLR116W	MSL5	2	psiblast	1k1gA_	178
YLR116W	MSL5	3	fold_recogniti	1k83A	203
YLR117C	CLF1	1	psiblast	1a17__	154
YLR117C	CLF1	2	fold_recogniti	1ihgA	103
YLR117C	CLF1	3	fold_recogniti	1ihgA	92
YLR117C	CLF1	4	psiblast	1fchA_	102
YLR117C	CLF1	5	psiblast	1fchA_	74
YLR117C	CLF1	6	psiblast	1fchA_	100
YLR117C	CLF1	7	unassigned	na	62
YLR118C	YLR118C	1	psiblast	1fj2A_	227
YLR119W	SRN2	1	unassigned	na	213
YLR120C	YPS1	1	fold_recogniti	1qdmA	139
YLR120C	YPS1	2	psiblast	1ypsA_	83
YLR120C	YPS1	3	psiblast	1ypsA_	270
YLR120C	YPS1	4	unassigned	na	77
YLR121C	YPS3	1	psiblast	1ypsA_	156

YLR121C	YPS3	2	psiblast	1ypsA_	277
YLR121C	YPS3	3	unassigned	na	75
YLR122C	YLR122C	1	unassigned	na	125
YLR123C	YLR123C	1	unassigned	na	109
YLR124W	YLR124W	1	unassigned	na	114
YLR125W	YLR125W	1	unassigned	na	136
YLR126C	YLR126C	1	msa	na	251
YLR127C	APC2	1	unassigned	na	93
YLR127C	APC2	2	msa	na	93
YLR127C	APC2	3	msa	na	220
YLR127C	APC2	4	unassigned	na	352
YLR127C	APC2	5	psiblast	1lddA_	95
YLR128W	YLR128W	1	unassigned	na	124
YLR128W	YLR128W	2	pfam	PF03556	145
YLR129W	DIP2	1	unassigned	na	66
YLR129W	DIP2	2	pfam	PF00400	85
YLR129W	DIP2	3	pfam	PF00400	40
YLR129W	DIP2	4	pfam	PF00400	51
YLR129W	DIP2	5	unassigned	na	67
YLR129W	DIP2	6	psiblast	1c5kA_	80
YLR129W	DIP2	7	psiblast	1erjA_	340
YLR129W	DIP2	8	msa	na	214
YLR130C	ZRT2	1	msa	na	205
YLR130C	ZRT2	2	pfam	PF02535	217
YLR131C	ACE2	1	msa	na	101
YLR131C	ACE2	2	unassigned	na	69
YLR131C	ACE2	3	msa	na	168
YLR131C	ACE2	4	psiblast	1lu6A_	59
YLR131C	ACE2	5	psiblast	1lu6A_	62
YLR131C	ACE2	6	unassigned	na	85
YLR131C	ACE2	7	psiblast	1ubdC_	92
YLR131C	ACE2	8	msa	na	82
YLR131C	ACE2	9	psiblast	2adr__	52
YLR132C	YLR132C	1	unassigned	na	290
YLR133W	CKI1	1	unassigned	na	143
YLR133W	CKI1	2	fold_recogniti	1j7iA	62
YLR133W	CKI1	3	fold_recogniti	1j7iA	158
YLR133W	CKI1	4	msa	na	156
YLR133W	CKI1	5	unassigned	na	63
YLR134W	PDC5	1	psiblast	1qpbA_	188
YLR134W	PDC5	2	psiblast	1qpbA_	165
YLR134W	PDC5	3	psiblast	1qpbA_	210
YLR135W	SLX4	1	unassigned	na	748
YLR136C	TIS11	1	msa	na	119
YLR136C	TIS11	2	msa	na	166
YLR137W	YLR137W	1	msa	na	225
YLR137W	YLR137W	2	unassigned	na	142
YLR138W	NHA1	1	psiblast	1fftA_	321

YLR138W	NHA1	2	unassigned	na	83
YLR138W	NHA1	3	msa	na	581
YLR139C	SLS1	1	unassigned	na	643
YLR140W	YLR140W	1	unassigned	na	108
YLR141W	RRN5	1	unassigned	na	363
YLR142W	PUT1	1	unassigned	na	106
YLR142W	PUT1	2	pfam	PF01619	370
YLR143W	YLR143W	1	fold_recogniti	1kh1A	159
YLR143W	YLR143W	2	pfam	PF01902	120
YLR143W	YLR143W	3	psiblast	1qd9A_	147
YLR143W	YLR143W	4	msa	na	161
YLR143W	YLR143W	5	unassigned	na	98
YLR144C	ACF2	1	fold_recogniti	1ldvA	208
YLR144C	ACF2	2	pfam	PF03639	571
YLR145W	YLR145W	1	unassigned	na	201
YLR146C	SPE4	1	psiblast	1inlA_	65
YLR146C	SPE4	2	psiblast	1inlA_	235
YLR147C	SMD3	1	psiblast	1d3bA_	101
YLR148W	PEP3	1	unassigned	na	304
YLR148W	PEP3	2	psiblast	1b89A_	519
YLR148W	PEP3	3	unassigned	na	95
YLR149C	YLR149C	1	psiblast	1erjA_	374
YLR149C	YLR149C	2	psiblast	1gg2B_	159
YLR149C	YLR149C	3	unassigned	na	197
YLR149C-A	YLR149C-A	1	unassigned	na	28
YLR150W	STM1	1	unassigned	na	273
YLR151C	PCD1	1	psiblast	1f3yA_	244
YLR151C	PCD1	2	unassigned	na	96
YLR152C	YLR152C	1	msa	na	199
YLR152C	YLR152C	2	unassigned	na	132
YLR152C	YLR152C	3	msa	na	245
YLR153C	ACS2	1	psiblast	1amuA_	266
YLR153C	ACS2	2	psiblast	1amuA_	265
YLR153C	ACS2	3	psiblast	1amuA_	152
YLR154C	RNH203	1	unassigned	na	110
YLR155C	ASP3-1	1	psiblast	1hfjA_	245
YLR155C	ASP3-1	2	psiblast	1hfjA_	117
YLR156W	YLR156W	1	unassigned	na	114
YLR162W	YLR162W	1	unassigned	na	118
YLR163C	MAS1	1	psiblast	1hr6B_	254
YLR163C	MAS1	2	psiblast	1hr6B_	208
YLR164W	YLR164W	1	unassigned	na	168
YLR165C	PUS5	1	psiblast	1kskA_	254
YLR166C	SEC10	1	fold_recogniti	1n1bA	53
YLR166C	SEC10	2	fold_recogniti	1qgkA	340
YLR166C	SEC10	3	fold_recogniti	1qgkA	74
YLR166C	SEC10	4	fold_recogniti	1qgkA	143
YLR166C	SEC10	5	fold_recogniti	1qgkA	261

YLR167W	RPS31	1	psiblast	1aarA_	89
YLR167W	RPS31	2	pfam	PF01599	63
YLR168C	YLR168C	1	msa	na	230
YLR169W	YLR169W	1	unassigned	na	117
YLR170C	APS1	1	psiblast	1gw5S_	88
YLR170C	APS1	2	psiblast	1gw5S_	68
YLR171W	YLR171W	1	unassigned	na	129
YLR172C	DPH5	1	psiblast	2cbf__	113
YLR172C	DPH5	2	psiblast	2cbf__	187
YLR173W	YLR173W	1	unassigned	na	608
YLR174W	IDP2	1	psiblast	1cnzA_	412
YLR175W	CBF5	1	psiblast	1k8wA_	264
YLR175W	CBF5	2	psiblast	1k8wA_	80
YLR175W	CBF5	3	fold_recogniti	1c1gA	139
YLR176C	RFX1	1	fold_recogniti	1deqA	230
YLR176C	RFX1	2	unassigned	na	54
YLR176C	RFX1	3	psiblast	1dp7P_	76
YLR176C	RFX1	4	fold_recogniti	1k83A	197
YLR176C	RFX1	5	unassigned	na	109
YLR176C	RFX1	6	msa	na	145
YLR177W	YLR177W	1	msa	na	52
YLR177W	YLR177W	2	unassigned	na	283
YLR177W	YLR177W	3	msa	na	293
YLR178C	TFS1	1	psiblast	1bd9A_	219
YLR179C	YLR179C	1	psiblast	1kn3A_	201
YLR180W	SAM1	1	psiblast	1qm4A_	168
YLR180W	SAM1	2	psiblast	1qm4A_	105
YLR180W	SAM1	3	psiblast	1qm4A_	109
YLR181C	VTA1	1	unassigned	na	84
YLR181C	VTA1	2	fold_recogniti	1ldvA	246
YLR182W	SWI6	1	msa	na	201
YLR182W	SWI6	2	psiblast	1sw6A_	310
YLR182W	SWI6	3	psiblast	1k1aA_	89
YLR182W	SWI6	4	fold_recogniti	1f6fA	203
YLR183C	TOS4	1	fold_recogniti	1g3gA	183
YLR183C	TOS4	2	unassigned	na	83
YLR183C	TOS4	3	msa	na	223
YLR184W	YLR184W	1	unassigned	na	115
YLR185W	RPL37A	1	psiblast	1k5yZ_	88
YLR186W	EMG1	1	unassigned	na	61
YLR186W	EMG1	2	pfam	PF03587	191
YLR187W	SKG3	1	unassigned	na	72
YLR187W	SKG3	2	pfam	PF00169	206
YLR187W	SKG3	3	unassigned	na	72
YLR187W	SKG3	4	msa	na	101
YLR187W	SKG3	5	msa	na	575
YLR188W	MDL1	1	unassigned	na	78
YLR188W	MDL1	2	psiblast	1jsqA_	315

YLR188W	MDL1	3	psiblast	1jsqA_	302
YLR189C	ATG26	1	unassigned	na	161
YLR189C	ATG26	2	fold_recogniti	1eazA	213
YLR189C	ATG26	3	unassigned	na	69
YLR189C	ATG26	4	msa	na	269
YLR189C	ATG26	5	psiblast	1iirA_	205
YLR189C	ATG26	6	psiblast	1iirA_	113
YLR189C	ATG26	7	psiblast	1iirA_	168
YLR190W	MMR1	1	fold_recogniti	1iu4A	302
YLR190W	MMR1	2	msa	na	189
YLR191W	PEX13	1	msa	na	109
YLR191W	PEX13	2	unassigned	na	89
YLR191W	PEX13	3	psiblast	1bmbA_	104
YLR191W	PEX13	4	psiblast	1aww__	84
YLR192C	HCR1	1	unassigned	na	265
YLR193C	YLR193C	1	unassigned	na	175
YLR194C	YLR194C	1	psiblast	1f00I_	152
YLR194C	YLR194C	2	psiblast	1f00I_	102
YLR195C	NMT1	1	psiblast	1iicA_	214
YLR195C	NMT1	2	psiblast	1iicA_	241
YLR196W	PWP1	1	unassigned	na	115
YLR196W	PWP1	2	psiblast	1gg2B_	339
YLR196W	PWP1	3	fold_recogniti	1jjuB	122
YLR197W	SIK1	1	msa	na	89
YLR197W	SIK1	2	unassigned	na	52
YLR197W	SIK1	3	msa	na	363
YLR198C	YLR198C	1	unassigned	na	119
YLR199C	YLR199C	1	unassigned	na	220
YLR200W	YKE2	1	psiblast	1fxkA_	114
YLR201C	FMP53	1	fold_recogniti	1jtxB	127
YLR201C	FMP53	2	fold_recogniti	1jtxB	133
YLR202C	YLR202C	1	unassigned	na	108
YLR203C	MSS51	1	unassigned	na	436
YLR204W	QRI5	1	unassigned	na	111
YLR205C	HMX1	1	psiblast	1qq8A_	273
YLR206W	ENT2	1	psiblast	1inzA_	159
YLR206W	ENT2	2	psiblast	1i84S_	362
YLR206W	ENT2	3	unassigned	na	92
YLR207W	HRD3	1	fold_recogniti	1fchA	169
YLR207W	HRD3	2	fold_recogniti	1fchA	71
YLR207W	HRD3	3	fold_recogniti	1fchA	112
YLR207W	HRD3	4	psiblast	1fchA_	144
YLR207W	HRD3	5	psiblast	1fchA_	82
YLR207W	HRD3	6	psiblast	1fchA_	128
YLR207W	HRD3	7	unassigned	na	127
YLR208W	SEC13	1	psiblast	1erjA_	297
YLR209C	PNP1	1	psiblast	1vfn__	311
YLR210W	CLB4	1	msa	na	201

YLR210W	CLB4	2	psiblast	1vin__	137
YLR210W	CLB4	3	psiblast	1vin__	122
YLR211C	YLR211C	1	unassigned	na	140
YLR212C	TUB4	1	psiblast	1ffxB_	473
YLR213C	CRR1	1	unassigned	na	136
YLR213C	CRR1	2	psiblast	1gbg__	286
YLR214W	FRE1	1	unassigned	na	144
YLR214W	FRE1	2	msa	na	187
YLR214W	FRE1	3	unassigned	na	55
YLR214W	FRE1	4	psiblast	1cnf__	130
YLR214W	FRE1	5	psiblast	1cnf__	170
YLR215C	CDC123	1	unassigned	na	174
YLR215C	CDC123	2	msa	na	186
YLR216C	CPR6	1	psiblast	1ihgA_	188
YLR216C	CPR6	2	psiblast	1ihgA_	95
YLR216C	CPR6	3	psiblast	1ihgA_	88
YLR217W	YLR217W	1	unassigned	na	107
YLR218C	YLR218C	1	unassigned	na	150
YLR219W	MSC3	1	fold_recogniti	1deqA	166
YLR219W	MSC3	2	fold_recogniti	1jv2B	359
YLR219W	MSC3	3	fold_recogniti	1jv2B	140
YLR219W	MSC3	4	unassigned	na	63
YLR220W	CCC1	1	unassigned	na	78
YLR220W	CCC1	2	pfam	PF01988	244
YLR221C	RSA3	1	msa	na	220
YLR222C	UTP13	1	fold_recogniti	1erjA	131
YLR222C	UTP13	2	pfam	PF00400	47
YLR222C	UTP13	3	pfam	PF00400	47
YLR222C	UTP13	4	unassigned	na	90
YLR222C	UTP13	5	psiblast	1gg2B_	330
YLR222C	UTP13	6	psiblast	1gxrA_	119
YLR222C	UTP13	7	unassigned	na	53
YLR223C	IFH1	1	psiblast	1i84S_	406
YLR223C	IFH1	2	msa	na	350
YLR223C	IFH1	3	unassigned	na	329
YLR224W	YLR224W	1	unassigned	na	369
YLR225C	YLR225C	1	unassigned	na	407
YLR226W	BUR2	1	unassigned	na	395
YLR227C	ADY4	1	unassigned	na	493
YLR228C	ECM22	1	psiblast	1pyiA_	161
YLR228C	ECM22	2	fold_recogniti	1k83A	386
YLR228C	ECM22	3	msa	na	109
YLR228C	ECM22	4	unassigned	na	158
YLR229C	CDC42	1	psiblast	1grnA_	191
YLR230W	YLR230W	1	unassigned	na	101
YLR231C	BNA5	1	psiblast	1elqA_	341
YLR231C	BNA5	2	psiblast	1elqA_	112
YLR232W	YLR232W	1	unassigned	na	115

YLR233C	EST1	1	fold_recogniti	1b89A	471
YLR233C	EST1	2	unassigned	na	228
YLR234W	TOP3	1	psiblast	1ecl__	119
YLR234W	TOP3	2	psiblast	1ecl__	265
YLR234W	TOP3	3	psiblast	1ecl__	105
YLR234W	TOP3	4	psiblast	1ecl__	167
YLR235C	YLR235C	1	unassigned	na	132
YLR236C	YLR236C	1	unassigned	na	107
YLR237W	THI7	1	pfam	PF02133	546
YLR237W	THI7	2	unassigned	na	52
YLR238W	FAR10	1	unassigned	na	112
YLR238W	FAR10	2	psiblast	1i84S_	366
YLR239C	LIP2	1	msa	na	109
YLR239C	LIP2	2	pfam	PF03099	158
YLR239C	LIP2	3	msa	na	61
YLR240W	VPS34	1	psiblast	1e7uA_	707
YLR240W	VPS34	2	psiblast	1e7uA_	168
YLR241W	YLR241W	1	msa	na	326
YLR241W	YLR241W	2	pfam	PF02714	456
YLR242C	ARV1	1	msa	na	135
YLR242C	ARV1	2	unassigned	na	186
YLR243W	YLR243W	1	psiblast	2ffhA_	272
YLR244C	MAP1	1	unassigned	na	115
YLR244C	MAP1	2	psiblast	4matA_	272
YLR245C	CDD1	1	psiblast	1jtkA_	142
YLR246W	ERF2	1	unassigned	na	163
YLR246W	ERF2	2	pfam	PF01529	134
YLR246W	ERF2	3	unassigned	na	62
YLR247C	YLR247C	1	msa	na	282
YLR247C	YLR247C	2	pfam	PF00176	436
YLR247C	YLR247C	3	msa	na	160
YLR247C	YLR247C	4	unassigned	na	358
YLR247C	YLR247C	5	pfam	PF00097	40
YLR247C	YLR247C	6	psiblast	1hv8A_	200
YLR247C	YLR247C	7	unassigned	na	80
YLR248W	RCK2	1	unassigned	na	120
YLR248W	RCK2	2	psiblast	1a06__	152
YLR248W	RCK2	3	psiblast	1a06__	260
YLR248W	RCK2	4	unassigned	na	78
YLR249W	YEF3	1	fold_recogniti	1lshA	71
YLR249W	YEF3	2	fold_recogniti	1lshA	108
YLR249W	YEF3	3	fold_recogniti	1lshA	145
YLR249W	YEF3	4	unassigned	na	100
YLR249W	YEF3	5	psiblast	1g291_	238
YLR249W	YEF3	6	psiblast	1e69A_	124
YLR249W	YEF3	7	psiblast	1e69A_	189
YLR249W	YEF3	8	unassigned	na	69
YLR250W	SSP120	1	psiblast	1bjfA_	144

YLR250W	SSP120	2	unassigned	na	90
YLR251W	SYM1	1	unassigned	na	197
YLR252W	YLR252W	1	unassigned	na	101
YLR253W	YLR253W	1	unassigned	na	153
YLR253W	YLR253W	2	psiblast	1f3mC_	147
YLR253W	YLR253W	3	psiblast	1f3mC_	194
YLR253W	YLR253W	4	unassigned	na	75
YLR254C	YLR254C	1	psiblast	1jchA_	189
YLR255C	YLR255C	1	unassigned	na	117
YLR256W	HAP1	1	psiblast	1hwtD_	168
YLR256W	HAP1	2	unassigned	na	1334
YLR257W	YLR257W	1	unassigned	na	321
YLR258W	GSY2	1	unassigned	na	87
YLR258W	GSY2	2	msa	na	273
YLR258W	GSY2	3	msa	na	90
YLR258W	GSY2	4	fold_recogniti	1f6dA	203
YLR258W	GSY2	5	unassigned	na	52
YLR259C	HSP60	1	psiblast	1iokA_	302
YLR259C	HSP60	2	psiblast	1iokA_	270
YLR260W	LCB5	1	unassigned	na	201
YLR260W	LCB5	2	pfam	PF00781	211
YLR260W	LCB5	3	msa	na	179
YLR260W	LCB5	4	unassigned	na	96
YLR261C	VPS63	1	unassigned	na	108
YLR262C	YPT6	1	psiblast	1d5cA_	215
YLR262C-A	YLR262C-A	1	unassigned	na	64
YLR263W	RED1	1	fold_recogniti	1be3C_	320
YLR263W	RED1	2	unassigned	na	89
YLR263W	RED1	3	unassigned	na	165
YLR263W	RED1	4	msa	na	163
YLR263W	RED1	5	unassigned	na	90
YLR264C-A	YLR264C-A	1	unassigned	na	38
YLR264W	RPS28B	1	pfam	PF01200	67
YLR265C	NEJ1	1	unassigned	na	342
YLR266C	PDR8	1	psiblast	1hwtD_	101
YLR266C	PDR8	2	unassigned	na	68
YLR266C	PDR8	3	msa	na	277
YLR266C	PDR8	4	unassigned	na	61
YLR266C	PDR8	5	msa	na	194
YLR267W	BOP2	1	unassigned	na	570
YLR268W	SEC22	1	psiblast	1ifqA_	125
YLR268W	SEC22	2	psiblast	1gl2A_	89
YLR269C	YLR269C	1	unassigned	na	116
YLR270W	DCS1	1	unassigned	na	157
YLR270W	DCS1	2	fold_recogniti	1av5A	193
YLR271W	YLR271W	1	pfam	PF01585	139
YLR271W	YLR271W	2	msa	na	135
YLR272C	YCS4	1	fold_recogniti	1ejlI	129

YLR272C	YCS4	2	fold_recogniti	1ejlI	62
YLR272C	YCS4	3	fold_recogniti	1ejlI	62
YLR272C	YCS4	4	fold_recogniti	1jpwA	146
YLR272C	YCS4	5	fold_recogniti	1jpwA	127
YLR272C	YCS4	6	fold_recogniti	1jpwA	123
YLR272C	YCS4	7	fold_recogniti	1jpwA	54
YLR272C	YCS4	8	fold_recogniti	1jpwA	184
YLR272C	YCS4	9	unassigned	na	58
YLR272C	YCS4	10	psiblast	1gw5B_	231
YLR273C	PIG1	1	unassigned	na	140
YLR273C	PIG1	2	pfam	PF03370	227
YLR273C	PIG1	3	unassigned	na	281
YLR274W	CDC46	1	msa	na	122
YLR274W	CDC46	2	pfam	PF00493	251
YLR274W	CDC46	3	psiblast	1g8pA_	233
YLR274W	CDC46	4	psiblast	1g8pA_	169
YLR275W	SMD2	1	psiblast	1b34B_	110
YLR276C	DBP9	1	psiblast	1hv8A_	242
YLR276C	DBP9	2	psiblast	1hv8A_	148
YLR276C	DBP9	3	psiblast	1d2mA_	96
YLR276C	DBP9	4	msa	na	108
YLR277C	YSH1	1	psiblast	1smlA_	170
YLR277C	YSH1	2	msa	na	261
YLR277C	YSH1	3	msa	na	98
YLR277C	YSH1	4	msa	na	250
YLR278C	YLR278C	1	psiblast	1pyiA_	185
YLR278C	YLR278C	2	unassigned	na	79
YLR278C	YLR278C	3	msa	na	417
YLR278C	YLR278C	4	msa	na	660
YLR279W	YLR279W	1	unassigned	na	129
YLR280C	YLR280C	1	unassigned	na	116
YLR281C	YLR281C	1	psiblast	1gqeA_	155
YLR282C	YLR282C	1	unassigned	na	113
YLR283W	YLR283W	1	msa	na	314
YLR284C	ECI1	1	psiblast	1hnoA_	280
YLR285W	NNT1	1	psiblast	1f3lA_	261
YLR286C	CTS1	1	psiblast	1hvq__	303
YLR286C	CTS1	2	fold_recogniti	1m2vB	259
YLR287C	YLR287C	1	fold_recogniti	1dofA	98
YLR287C	YLR287C	2	fold_recogniti	1dofA	257
YLR287C-A	RPS30A	1	unassigned	na	63
YLR288C	MEC3	1	unassigned	na	474
YLR289W	GUF1	1	psiblast	1dar__	304
YLR289W	GUF1	2	psiblast	1dar__	265
YLR289W	GUF1	3	unassigned	na	76
YLR290C	YLR290C	1	psiblast	1lrkA_	229
YLR290C	YLR290C	2	psiblast	1lrkA_	48
YLR291C	GCD7	1	msa	na	137

YLR291C	GCD7	2	fold_recogniti	1m0sA	180
YLR291C	GCD7	3	fold_recogniti	1m0sA	64
YLR292C	SEC72	1	unassigned	na	139
YLR292C	SEC72	2	pfam	PF00515	54
YLR293C	GSP1	1	psiblast	1a2kC_	219
YLR294C	YLR294C	1	unassigned	na	109
YLR295C	ATP14	1	unassigned	na	124
YLR296W	YLR296W	1	unassigned	na	108
YLR297W	YLR297W	1	unassigned	na	129
YLR298C	YHC1	1	msa	na	69
YLR298C	YHC1	2	unassigned	na	162
YLR299W	ECM38	1	fold_recogniti	1itwA	274
YLR299W	ECM38	2	fold_recogniti	1itwA	166
YLR299W	ECM38	3	fold_recogniti	9gaaA	220
YLR300W	EXG1	1	psiblast	1cz1A_	328
YLR300W	EXG1	2	psiblast	1cz1A_	120
YLR301W	YLR301W	1	unassigned	na	244
YLR302C	YLR302C	1	unassigned	na	120
YLR303W	MET17	1	psiblast	1gc0A_	296
YLR303W	MET17	2	psiblast	1gc0A_	148
YLR304C	ACO1	1	psiblast	5acn__	577
YLR304C	ACO1	2	psiblast	5acn__	201
YLR305C	STT4	1	unassigned	na	505
YLR305C	STT4	2	msa	na	312
YLR305C	STT4	3	unassigned	na	463
YLR305C	STT4	4	psiblast	1he8A_	264
YLR305C	STT4	5	psiblast	1he8A_	131
YLR305C	STT4	6	psiblast	1he8A_	225
YLR306W	UBC12	1	psiblast	2aak__	188
YLR307W	CDA1	1	unassigned	na	106
YLR307W	CDA1	2	pfam	PF01522	195
YLR308W	CDA2	1	unassigned	na	114
YLR308W	CDA2	2	pfam	PF01522	198
YLR309C	IMH1	1	psiblast	1i84S_	336
YLR309C	IMH1	2	msa	na	216
YLR309C	IMH1	3	psiblast	2tmaA_	84
YLR309C	IMH1	4	psiblast	2tmaA_	83
YLR309C	IMH1	5	psiblast	2tmaA_	62
YLR309C	IMH1	6	psiblast	2tmaA_	130
YLR310C	CDC25	1	psiblast	2ptk__	129
YLR310C	CDC25	2	psiblast	2ptk__	127
YLR310C	CDC25	3	unassigned	na	719
YLR310C	CDC25	4	psiblast	1awe__	104
YLR310C	CDC25	5	psiblast	1bkdS_	212
YLR310C	CDC25	6	psiblast	1bkdS_	298
YLR311C	YLR311C	1	unassigned	na	115
YLR312C	YLR312C	1	unassigned	na	398
YLR312W-A	MRPL15	1	unassigned	na	253

YLR313C	SPH1	1	fold_recogniti	1itwA	115
YLR313C	SPH1	2	fold_recogniti	1itwA	135
YLR313C	SPH1	3	fold_recogniti	1itwA	280
YLR314C	CDC3	1	msa	na	90
YLR314C	CDC3	2	psiblast	1jnyA_	148
YLR314C	CDC3	3	unassigned	na	52
YLR314C	CDC3	4	psiblast	1e5wA_	148
YLR314C	CDC3	5	psiblast	1dg3A_	82
YLR315W	NKP2	1	fold_recogniti	1b68A	153
YLR316C	TAD3	1	fold_recogniti	1jtkA	163
YLR317W	YLR317W	1	unassigned	na	144
YLR318W	EST2	1	unassigned	na	301
YLR318W	EST2	2	msa	na	283
YLR318W	EST2	3	psiblast	1d0eA_	189
YLR318W	EST2	4	unassigned	na	111
YLR319C	BUD6	1	unassigned	na	79
YLR319C	BUD6	2	psiblast	1jchA_	200
YLR319C	BUD6	3	psiblast	1i84S_	339
YLR319C	BUD6	4	psiblast	2tmaA_	170
YLR320W	MMS22	1	unassigned	na	1454
YLR321C	SFH1	1	unassigned	na	137
YLR321C	SFH1	2	msa	na	289
YLR322W	VPS65	1	unassigned	na	104
YLR323C	CWC24	1	msa	na	168
YLR323C	CWC24	2	psiblast	1rmd__	91
YLR324W	PEX30	1	unassigned	na	282
YLR324W	PEX30	2	msa	na	154
YLR324W	PEX30	3	unassigned	na	87
YLR325C	RPL38	1	pfam	PF01781	78
YLR326W	YLR326W	1	unassigned	na	240
YLR327C	YLR327C	1	unassigned	na	86
YLR328W	NMA1	1	unassigned	na	111
YLR328W	NMA1	2	psiblast	1kkuA_	290
YLR329W	REC102	1	unassigned	na	200
YLR330W	CHS5	1	unassigned	na	80
YLR330W	CHS5	2	fold_recogniti	1jv2B	281
YLR330W	CHS5	3	fold_recogniti	1jv2B	140
YLR330W	CHS5	4	fold_recogniti	1jv2B	87
YLR330W	CHS5	5	fold_recogniti	1l8mA	83
YLR331C	JIP3	1	unassigned	na	125
YLR332W	MID2	1	fold_recogniti	1m2vB	324
YLR332W	MID2	2	unassigned	na	52
YLR333C	RPS25B	1	pfam	PF03297	108
YLR334C	YLR334C	1	unassigned	na	126
YLR335W	NUP2	1	unassigned	na	149
YLR335W	NUP2	2	fold_recogniti	1m2vB	124
YLR335W	NUP2	3	fold_recogniti	1l8mA	87
YLR335W	NUP2	4	fold_recogniti	1l8mA	73

YLR335W	NUP2	5	fold_recogniti	1l8mA	82
YLR335W	NUP2	6	fold_recogniti	1l8mA	70
YLR335W	NUP2	7	psiblast	1rrpB_	135
YLR336C	SGD1	1	msa	na	309
YLR336C	SGD1	2	psiblast	1hu3A_	133
YLR336C	SGD1	3	psiblast	1hu3A_	153
YLR336C	SGD1	4	msa	na	242
YLR336C	SGD1	5	unassigned	na	62
YLR337C	VRP1	1	msa	na	601
YLR337C	VRP1	2	unassigned	na	99
YLR337C	VRP1	3	msa	na	117
YLR338W	YLR338W	1	fold_recogniti	1la5A	188
YLR338W	YLR338W	2	unassigned	na	97
YLR339C	YLR339C	1	unassigned	na	183
YLR340W	RPP0	1	psiblast	1jj2G_	312
YLR341W	SPO77	1	unassigned	na	477
YLR342W	FKS1	1	msa	na	153
YLR342W	FKS1	2	msa	na	235
YLR342W	FKS1	3	unassigned	na	414
YLR342W	FKS1	4	pfam	PF02364	864
YLR342W	FKS1	5	msa	na	210
YLR343W	YLR343W	1	fold_recogniti	1lf1A	283
YLR343W	YLR343W	2	unassigned	na	63
YLR343W	YLR343W	3	msa	na	209
YLR344W	RPL26A	1	psiblast	1k5yU_	127
YLR345W	YLR345W	1	psiblast	1bif__	289
YLR345W	YLR345W	2	psiblast	1bif__	220
YLR346C	YLR346C	1	unassigned	na	101
YLR347C	KAP95	1	psiblast	1qgkA_	248
YLR347C	KAP95	2	psiblast	1qgkA_	194
YLR347C	KAP95	3	psiblast	1qgkA_	99
YLR347C	KAP95	4	psiblast	1qgkA_	107
YLR347C	KAP95	5	psiblast	1qgkA_	213
YLR348C	DIC1	1	fold_recogniti	1is2A	298
YLR349W	YLR349W	1	unassigned	na	168
YLR350W	ORM2	1	msa	na	216
YLR351C	NIT3	1	psiblast	1f89A_	291
YLR352W	YLR352W	1	msa	na	606
YLR352W	YLR352W	2	unassigned	na	201
YLR353W	BUD8	1	unassigned	na	64
YLR353W	BUD8	2	fold_recogniti	1jv2B	354
YLR353W	BUD8	3	fold_recogniti	1jv2B	122
YLR353W	BUD8	4	unassigned	na	63
YLR354C	TAL1	1	psiblast	1f05A_	335
YLR355C	ILV5	1	psiblast	1qmgA_	395
YLR356W	YLR356W	1	unassigned	na	197
YLR357W	RSC2	1	psiblast	1e6iA_	189
YLR357W	RSC2	2	unassigned	na	86

YLR357W	RSC2	3	pfam	PF00439	118
YLR357W	RSC2	4	pfam	PF01426	171
YLR357W	RSC2	5	msa	na	118
YLR357W	RSC2	6	unassigned	na	207
YLR358C	YLR358C	1	unassigned	na	187
YLR359W	ADE13	1	psiblast	1f1oA_	166
YLR359W	ADE13	2	psiblast	1f1oA_	97
YLR359W	ADE13	3	psiblast	1f1oA_	219
YLR360W	VPS38	1	unassigned	na	128
YLR360W	VPS38	2	psiblast	1i84S_	186
YLR360W	VPS38	3	unassigned	na	125
YLR361C	DCR2	1	unassigned	na	122
YLR361C	DCR2	2	psiblast	1kbpA_	203
YLR361C	DCR2	3	psiblast	1kbpA_	253
YLR362W	STE11	1	fold_recogniti	1b0xA	136
YLR362W	STE11	2	psiblast	1f3mA_	96
YLR362W	STE11	3	unassigned	na	137
YLR362W	STE11	4	psiblast	1f3mC_	187
YLR362W	STE11	5	psiblast	1f3mC_	182
YLR363C	NMD4	1	unassigned	na	218
YLR363W-A	YLR363W-A	1	unassigned	na	85
YLR364W	YLR364W	1	psiblast	1jhb__	109
YLR365W	YLR365W	1	unassigned	na	110
YLR366W	YLR366W	1	unassigned	na	101
YLR367W	RPS22B	1	psiblast	1k5xH_	79
YLR367W	RPS22B	2	psiblast	1k5xH_	51
YLR368W	MDM30	1	pfam	PF00646	75
YLR368W	MDM30	2	unassigned	na	523
YLR369W	SSQ1	1	psiblast	1hpm__	226
YLR369W	SSQ1	2	psiblast	1hpm__	115
YLR369W	SSQ1	3	psiblast	1hpm__	79
YLR369W	SSQ1	4	psiblast	1bpr__	237
YLR370C	ARC18	1	psiblast	1k8kE_	71
YLR370C	ARC18	2	psiblast	1k8kE_	107
YLR371W	ROM2	1	fold_recogniti	1ldvA	137
YLR371W	ROM2	2	fold_recogniti	1m2vB	394
YLR371W	ROM2	3	unassigned	na	110
YLR371W	ROM2	4	psiblast	1ki1B_	208
YLR371W	ROM2	5	psiblast	1ki1B_	183
YLR371W	ROM2	6	pfam	PF00780	324
YLR372W	SUR4	1	pfam	PF01151	345
YLR373C	VID22	1	msa	na	672
YLR373C	VID22	2	unassigned	na	229
YLR374C	YLR374C	1	unassigned	na	129
YLR375W	STP3	1	psiblast	1tf3A_	84
YLR375W	STP3	2	psiblast	1a1fA_	53
YLR375W	STP3	3	psiblast	1f2iG_	60
YLR375W	STP3	4	msa	na	146

YLR376C	PSY3	1	unassigned	na	242
YLR377C	FBP1	1	psiblast	1bk4A_	209
YLR377C	FBP1	2	psiblast	1bk4A_	139
YLR378C	SEC61	1	msa	na	72
YLR378C	SEC61	2	pfam	PF00344	408
YLR379W	YLR379W	1	unassigned	na	124
YLR380W	CSR1	1	unassigned	na	52
YLR380W	CSR1	2	psiblast	1aua__	82
YLR380W	CSR1	3	psiblast	1aua__	274
YLR381W	CTF3	1	fold_recogniti	1b3uA	178
YLR381W	CTF3	2	fold_recogniti	1b3uA	73
YLR381W	CTF3	3	fold_recogniti	1b3uA	129
YLR381W	CTF3	4	fold_recogniti	1b3uA	218
YLR381W	CTF3	5	unassigned	na	135
YLR382C	NAM2	1	psiblast	1ffyA_	152
YLR382C	NAM2	2	psiblast	1ffyA_	238
YLR382C	NAM2	3	psiblast	1ffyA_	261
YLR382C	NAM2	4	psiblast	1ffyA_	243
YLR383W	RHC18	1	unassigned	na	69
YLR383W	RHC18	2	psiblast	1f2tA_	102
YLR383W	RHC18	3	psiblast	1i84S_	335
YLR383W	RHC18	4	fold_recogniti	1gxlA	65
YLR383W	RHC18	5	fold_recogniti	1gxlA	87
YLR383W	RHC18	6	fold_recogniti	1gxlA	58
YLR383W	RHC18	7	psiblast	2tmaA_	56
YLR383W	RHC18	8	psiblast	2tmaA_	82
YLR383W	RHC18	9	psiblast	2tmaA_	63
YLR383W	RHC18	10	psiblast	2tmaA_	92
YLR383W	RHC18	11	psiblast	1e69A_	105
YLR384C	IKI3	1	psiblast	1gxrA_	111
YLR384C	IKI3	2	psiblast	1gg2B_	314
YLR384C	IKI3	3	psiblast	1erjA_	202
YLR384C	IKI3	4	unassigned	na	209
YLR384C	IKI3	5	fold_recogniti	1qqeA	198
YLR384C	IKI3	6	fold_recogniti	1qqeA	154
YLR384C	IKI3	7	msa	na	161
YLR385C	SWC7	1	unassigned	na	132
YLR386W	VAC14	1	unassigned	na	199
YLR386W	VAC14	2	fold_recogniti	1b89A	454
YLR386W	VAC14	3	msa	na	227
YLR387C	YLR387C	1	psiblast	1meyG_	96
YLR387C	YLR387C	2	msa	na	193
YLR387C	YLR387C	3	unassigned	na	143
YLR388W	RPS29A	1	pfam	PF00253	56
YLR389C	STE23	1	unassigned	na	69
YLR389C	STE23	2	psiblast	1hr6B_	241
YLR389C	STE23	3	psiblast	1hr6B_	244
YLR389C	STE23	4	msa	na	434

YLR390W	ECM19	1	unassigned	na	112
YLR390W-A	CCW14	1	fold_recogniti	1m2vB	238
YLR392C	YLR392C	1	msa	na	236
YLR392C	YLR392C	2	unassigned	na	282
YLR393W	ATP10	1	unassigned	na	279
YLR394W	CST9	1	fold_recogniti	1jm7B	188
YLR394W	CST9	2	unassigned	na	294
YLR395C	COX8	1	unassigned	na	78
YLR396C	VPS33	1	psiblast	1fvfA_	240
YLR396C	VPS33	2	psiblast	1fvfA_	156
YLR396C	VPS33	3	psiblast	1fvfA_	213
YLR396C	VPS33	4	msa	na	82
YLR397C	AFG2	1	psiblast	1e32A_	120
YLR397C	AFG2	2	psiblast	1e32A_	121
YLR397C	AFG2	3	psiblast	1e32A_	172
YLR397C	AFG2	4	psiblast	1e32A_	78
YLR397C	AFG2	5	psiblast	1d2nA_	176
YLR397C	AFG2	6	fold_recogniti	1iy2A	113
YLR398C	SKI2	1	msa	na	170
YLR398C	SKI2	2	unassigned	na	104
YLR398C	SKI2	3	psiblast	1fuuA_	393
YLR398C	SKI2	4	psiblast	1c4oA_	224
YLR398C	SKI2	5	unassigned	na	73
YLR398C	SKI2	6	fold_recogniti	1n1bA	323
YLR399C	BDF1	1	fold_recogniti	1ldvA	151
YLR399C	BDF1	2	pfam	PF00439	118
YLR399C	BDF1	3	psiblast	1e6iA_	203
YLR399C	BDF1	4	unassigned	na	55
YLR399C	BDF1	5	msa	na	159
YLR400W	YLR400W	1	unassigned	na	157
YLR401C	DUS3	1	msa	na	257
YLR401C	DUS3	2	psiblast	1ep1A_	352
YLR402W	YLR402W	1	unassigned	na	171
YLR403W	SFP1	1	unassigned	na	113
YLR403W	SFP1	2	msa	na	210
YLR403W	SFP1	3	psiblast	1meyG_	111
YLR403W	SFP1	4	msa	na	77
YLR403W	SFP1	5	psiblast	2gliA_	78
YLR403W	SFP1	6	psiblast	2gliA_	94
YLR404W	YLR404W	1	unassigned	na	285
YLR405W	DUS4	1	psiblast	1ep1A_	279
YLR405W	DUS4	2	psiblast	1h5yA_	88
YLR406C	RPL31B	1	psiblast	1k5yW_	113
YLR407W	YLR407W	1	unassigned	na	229
YLR408C	YLR408C	1	unassigned	na	122
YLR409C	UTP21	1	psiblast	1gg2B_	315
YLR409C	UTP21	2	psiblast	1gxrA_	342
YLR409C	UTP21	3	psiblast	1erjA_	89

YLR409C	UTP21	4	msa	na	193
YLR410W	VIP1	1	unassigned	na	234
YLR410W	VIP1	2	psiblast	2dln__	286
YLR410W	VIP1	3	fold_recogniti	1qfxA	124
YLR410W	VIP1	4	fold_recogniti	1qfxA	104
YLR410W	VIP1	5	unassigned	na	398
YLR411W	CTR3	1	msa	na	75
YLR411W	CTR3	2	msa	na	166
YLR412W	YLR412W	1	msa	na	194
YLR412W	YLR412W	2	unassigned	na	80
YLR413W	YLR413W	1	fold_recogniti	1ei3A	73
YLR413W	YLR413W	2	fold_recogniti	1k83A	401
YLR413W	YLR413W	3	unassigned	na	201
YLR414C	YLR414C	1	unassigned	na	263
YLR415C	YLR415C	1	unassigned	na	112
YLR416C	YLR416C	1	unassigned	na	132
YLR417W	VPS36	1	unassigned	na	147
YLR417W	VPS36	2	msa	na	419
YLR418C	CDC73	1	unassigned	na	133
YLR418C	CDC73	2	msa	na	260
YLR419W	YLR419W	1	unassigned	na	122
YLR419W	YLR419W	2	msa	na	127
YLR419W	YLR419W	3	unassigned	na	105
YLR419W	YLR419W	4	pfam	PF00627	57
YLR419W	YLR419W	5	msa	na	156
YLR419W	YLR419W	6	psiblast	1hv8A_	251
YLR419W	YLR419W	7	psiblast	1hv8A_	181
YLR419W	YLR419W	8	msa	na	310
YLR419W	YLR419W	9	msa	na	126
YLR420W	URA4	1	psiblast	1j79A_	364
YLR421C	RPN13	1	unassigned	na	156
YLR422W	YLR422W	1	unassigned	na	1253
YLR422W	YLR422W	2	msa	na	603
YLR422W	YLR422W	3	unassigned	na	76
YLR423C	ATG17	1	psiblast	1gojA_	255
YLR423C	ATG17	2	fold_recogniti	1i84S	162
YLR424W	YLR424W	1	pfam	PF01585	111
YLR424W	YLR424W	2	unassigned	na	82
YLR424W	YLR424W	3	msa	na	515
YLR425W	TUS1	1	fold_recogniti	1m2vB	304
YLR425W	TUS1	2	unassigned	na	138
YLR425W	TUS1	3	psiblast	1ki1B_	218
YLR425W	TUS1	4	psiblast	1ki1B_	167
YLR425W	TUS1	5	msa	na	480
YLR426W	YLR426W	1	psiblast	1i01A_	326
YLR427W	MAG2	1	msa	na	148
YLR427W	MAG2	2	psiblast	1jm7B_	128
YLR427W	MAG2	3	psiblast	1fre__	38

YLR427W	MAG2	4	msa	na	179
YLR427W	MAG2	5	unassigned	na	177
YLR428C	YLR428C	1	unassigned	na	114
YLR429W	CRN1	1	unassigned	na	70
YLR429W	CRN1	2	psiblast	1erjA_	320
YLR429W	CRN1	3	psiblast	1gxrA_	76
YLR429W	CRN1	4	fold_recogniti	1k83A	185
YLR430W	SEN1	1	unassigned	na	1122
YLR430W	SEN1	2	msa	na	198
YLR430W	SEN1	3	psiblast	1pjr__	335
YLR430W	SEN1	4	psiblast	1pjr__	378
YLR430W	SEN1	5	unassigned	na	198
YLR431C	ATG23	1	psiblast	1i84S_	122
YLR431C	ATG23	2	msa	na	139
YLR431C	ATG23	3	psiblast	1gk4A_	192
YLR432W	IMD3	1	psiblast	1jr1A_	523
YLR433C	CNA1	1	psiblast	1auiA_	553
YLR434C	YLR434C	1	unassigned	na	127
YLR435W	TSR2	1	msa	na	249
YLR436C	ECM30	1	msa	na	406
YLR436C	ECM30	2	unassigned	na	868
YLR437C	YLR437C	1	unassigned	na	133
YLR438C-A	LSM3	1	psiblast	1i81A_	89
YLR438W	CAR2	1	psiblast	2canA_	424
YLR439W	MRPL4	1	unassigned	na	85
YLR439W	MRPL4	2	fold_recogniti	1ffkS	106
YLR439W	MRPL4	3	unassigned	na	128
YLR440C	SEC39	1	fold_recogniti	1qgkA	285
YLR440C	SEC39	2	fold_recogniti	1qgkA	104
YLR440C	SEC39	3	fold_recogniti	1qgkA	109
YLR440C	SEC39	4	fold_recogniti	1qgkA	211
YLR441C	RPS1A	1	pfam	PF01015	255
YLR442C	SIR3	1	psiblast	1i84S_	339
YLR442C	SIR3	2	unassigned	na	181
YLR442C	SIR3	3	psiblast	1fnnA_	143
YLR442C	SIR3	4	psiblast	1fnnA_	315
YLR443W	ECM7	1	unassigned	na	448
YLR444C	YLR444C	1	unassigned	na	100
YLR445W	YLR445W	1	unassigned	na	134
YLR446W	YLR446W	1	psiblast	1bg3_B:1	433
YLR447C	VMA6	1	fold_recogniti	1b89A	345
YLR448W	RPL6B	1	pfam	PF01159	176
YLR449W	FPR4	1	fold_recogniti	1l8mA	71
YLR449W	FPR4	2	fold_recogniti	1l8mA	79
YLR449W	FPR4	3	fold_recogniti	1l8mA	70
YLR449W	FPR4	4	fold_recogniti	1l8mA	63
YLR449W	FPR4	5	psiblast	1yat__	109
YLR450W	HMG2	1	unassigned	na	66

YLR450W	HMG2	2	msa	na	467
YLR450W	HMG2	3	unassigned	na	52
YLR450W	HMG2	4	psiblast	1dq8A_	100
YLR450W	HMG2	5	psiblast	1dq8A_	258
YLR450W	HMG2	6	psiblast	1dq8A_	102
YLR451W	LEU3	1	psiblast	1hwtD_	106
YLR451W	LEU3	2	msa	na	385
YLR451W	LEU3	3	unassigned	na	395
YLR452C	SST2	1	msa	na	217
YLR452C	SST2	2	unassigned	na	60
YLR452C	SST2	3	pfam	PF00610	79
YLR452C	SST2	4	psiblast	1agrE_	254
YLR452C	SST2	5	unassigned	na	88
YLR453C	RIF2	1	unassigned	na	395
YLR454W	FMP27	1	unassigned	na	1357
YLR454W	FMP27	2	msa	na	386
YLR454W	FMP27	3	unassigned	na	432
YLR454W	FMP27	4	msa	na	453
YLR455W	YLR455W	1	psiblast	1khcA_	83
YLR455W	YLR455W	2	psiblast	1khcA_	62
YLR455W	YLR455W	3	msa	na	97
YLR455W	YLR455W	4	unassigned	na	62
YLR456W	YLR456W	1	fold_recogniti	1jnwA	156
YLR456W	YLR456W	2	fold_recogniti	1jnwA	48
YLR457C	NBP1	1	unassigned	na	161
YLR457C	NBP1	2	msa	na	158
YLR458W	YLR458W	1	unassigned	na	126
YLR459W	CDC91	1	msa	na	127
YLR459W	CDC91	2	unassigned	na	61
YLR459W	CDC91	3	msa	na	131
YLR459W	CDC91	4	unassigned	na	75
YLR460C	YLR460C	1	psiblast	1tehA_	232
YLR460C	YLR460C	2	psiblast	1tehA_	144
YLR461W	PAU4	1	pfam	PF00660	120
YLR462W	YLR462W	1	msa	na	118
YLR462W	YLR462W	2	msa	na	84
YLR463C	YLR463C	1	unassigned	na	183
YLR465C	BSC3	1	unassigned	na	102
YLR466W	YRF1-4	1	msa	na	93
YLR466W	YRF1-4	2	unassigned	na	80
YLR466W	YRF1-4	3	msa	na	141
YLR466W	YRF1-4	4	psiblast	1fuuA_	464
YLR466W	YRF1-4	5	msa	na	229
YLR466W	YRF1-4	6	unassigned	na	375
YML001W	YPT7	1	psiblast	1ky2A_	208
YML002W	YML002W	1	psiblast	1ihbA_	96
YML002W	YML002W	2	psiblast	1ihbA_	70
YML002W	YML002W	3	psiblast	1k1aA_	116

YML002W	YML002W	4	psiblast	1k1aA_	165
YML002W	YML002W	5	unassigned	na	290
YML003W	YML003W	1	unassigned	na	290
YML004C	GLO1	1	psiblast	1froA_	111
YML004C	GLO1	2	psiblast	1froA_	65
YML004C	GLO1	3	psiblast	1jc5A_	150
YML005W	TRM12	1	unassigned	na	226
YML005W	TRM12	2	psiblast	1g6q1_	236
YML006C	GIS4	1	unassigned	na	774
YML007C-A	YML007C-A	1	unassigned	na	36
YML007W	YAP1	1	psiblast	1gd2E_	187
YML007W	YAP1	2	unassigned	na	138
YML007W	YAP1	3	msa	na	325
YML008C	ERG6	1	psiblast	1kp9A_	142
YML008C	ERG6	2	psiblast	1kp9A_	241
YML009C	MRPL39	1	unassigned	na	70
YML009C-A	YML009C-A	1	unassigned	na	108
YML009W-B	YML009W-B	1	unassigned	na	158
YML010W	SPT5	1	msa	na	232
YML010W	SPT5	2	pfam	PF03439	82
YML010W	SPT5	3	pfam	PF03439	67
YML010W	SPT5	4	pfam	PF00467	59
YML010W	SPT5	5	unassigned	na	91
YML010W	SPT5	6	pfam	PF00467	39
YML010W	SPT5	7	pfam	PF00467	47
YML010W	SPT5	8	unassigned	na	177
YML010W	SPT5	9	pfam	PF00467	48
YML010W	SPT5	10	unassigned	na	66
YML010W	SPT5	11	psiblast	1deqA_	155
YML011C	YML011C	1	unassigned	na	177
YML012C-A	YML012C-A	1	unassigned	na	125
YML012W	ERV25	1	pfam	PF01105	211
YML013W	SEL1	1	msa	na	95
YML013W	SEL1	2	unassigned	na	87
YML013W	SEL1	3	msa	na	169
YML013W	SEL1	4	unassigned	na	75
YML013W	SEL1	5	psiblast	1h8cA_	92
YML013W	SEL1	6	unassigned	na	66
YML014W	TRM9	1	psiblast	1kp9A_	113
YML014W	TRM9	2	psiblast	1kp9A_	166
YML015C	TAF11	1	unassigned	na	113
YML015C	TAF11	2	psiblast	1bh8B_	125
YML015C	TAF11	3	unassigned	na	108
YML016C	PPZ1	1	fold_recogniti	1m2vB	354
YML016C	PPZ1	2	psiblast	1it6A_	338
YML017W	PSP2	1	unassigned	na	135
YML017W	PSP2	2	fold_recogniti	1ldvA	130
YML017W	PSP2	3	fold_recogniti	1l8mA	92

YML017W	PSP2	4	fold_recogniti	1l8mA	73
YML017W	PSP2	5	fold_recogniti	1l8mA	78
YML017W	PSP2	6	fold_recogniti	1l8mA	70
YML018C	YML018C	1	msa	na	87
YML018C	YML018C	2	msa	na	306
YML019W	OST6	1	msa	na	74
YML019W	OST6	2	msa	na	258
YML020W	YML020W	1	fold_recogniti	1ldvA	275
YML020W	YML020W	2	unassigned	na	76
YML020W	YML020W	3	msa	na	213
YML020W	YML020W	4	unassigned	na	100
YML021C	UNG1	1	unassigned	na	86
YML021C	UNG1	2	psiblast	1akz__	273
YML022W	APT1	1	psiblast	1g2pA_	187
YML023C	YML023C	1	unassigned	na	556
YML024W	RPS17A	1	pfam	PF00833	136
YML025C	YML6	1	psiblast	1dmgA_	286
YML027W	YOX1	1	msa	na	134
YML027W	YOX1	2	psiblast	1b8iA_	112
YML027W	YOX1	3	msa	na	139
YML028W	TSA1	1	psiblast	1qmvA_	196
YML029W	USA1	1	unassigned	na	838
YML030W	YML030W	1	unassigned	na	159
YML031C-A	YML031C-A	1	unassigned	na	111
YML031W	NDC1	1	fold_recogniti	1ej6B	655
YML032C	RAD52	1	msa	na	145
YML032C	RAD52	2	fold_recogniti	1k83A	359
YML034C-A	YML034C-A	1	unassigned	na	132
YML034W	SRC1	1	msa	na	149
YML034W	SRC1	2	fold_recogniti	1iu4A	287
YML034W	SRC1	3	unassigned	na	242
YML034W	SRC1	4	msa	na	156
YML035C	AMD1	1	msa	na	240
YML035C	AMD1	2	unassigned	na	53
YML035C	AMD1	3	pfam	PF00962	269
YML035C	AMD1	4	psiblast	1m7mA_	248
YML036W	YML036W	1	unassigned	na	189
YML037C	YML037C	1	unassigned	na	340
YML038C	YMD8	1	pfam	PF00892	157
YML038C	YMD8	2	unassigned	na	119
YML038C	YMD8	3	msa	na	111
YML038C	YMD8	4	unassigned	na	55
YML041C	VPS71	1	unassigned	na	182
YML041C	VPS71	2	msa	na	98
YML042W	CAT2	1	unassigned	na	62
YML042W	CAT2	2	pfam	PF00755	203
YML042W	CAT2	3	fold_recogniti	1cb7B	133
YML042W	CAT2	4	fold_recogniti	1cb7B	149

YML042W	CAT2	5	fold_recogniti	1cb7B	123
YML043C	RRN11	1	unassigned	na	120
YML043C	RRN11	2	msa	na	172
YML043C	RRN11	3	unassigned	na	215
YML046W	PRP39	1	unassigned	na	64
YML046W	PRP39	2	psiblast	1fchA_	145
YML046W	PRP39	3	psiblast	1fchA_	61
YML046W	PRP39	4	psiblast	1fchA_	83
YML046W	PRP39	5	psiblast	1ihgA_	58
YML046W	PRP39	6	psiblast	1ihgA_	135
YML046W	PRP39	7	unassigned	na	83
YML047C	PRM6	1	msa	na	352
YML047W-A	YML047W-A	1	unassigned	na	121
YML048W	GSF2	1	unassigned	na	403
YML049C	RSE1	1	msa	na	858
YML049C	RSE1	2	unassigned	na	74
YML049C	RSE1	3	pfam	PF03178	429
YML050W	YML050W	1	unassigned	na	130
YML050W	YML050W	2	msa	na	181
YML051W	GAL80	1	psiblast	1ofgA_	175
YML051W	GAL80	2	psiblast	1ofgA_	260
YML052W	SUR7	1	msa	na	232
YML052W	SUR7	2	unassigned	na	70
YML053C	YML053C	1	unassigned	na	212
YML054C	CYB2	1	unassigned	na	79
YML054C	CYB2	2	psiblast	1fcbA_	97
YML054C	CYB2	3	psiblast	1fcbA_	415
YML055W	SPC2	1	unassigned	na	178
YML056C	IMD4	1	psiblast	1b3oA_	524
YML057C-A	YML057C-A	1	unassigned	na	129
YML057W	CMP2	1	psiblast	1auiA_	604
YML058W	SML1	1	unassigned	na	104
YML058W-A	HUG1	1	unassigned	na	68
YML059C	YML059C	1	unassigned	na	299
YML059C	YML059C	2	msa	na	82
YML059C	YML059C	3	unassigned	na	270
YML059C	YML059C	4	psiblast	1apk__	420
YML059C	YML059C	5	psiblast	2gapA_	92
YML059C	YML059C	6	msa	na	120
YML059C	YML059C	7	unassigned	na	66
YML059C	YML059C	8	msa	na	330
YML060W	OGG1	1	psiblast	1ko9A_	74
YML060W	OGG1	2	psiblast	1ko9A_	111
YML060W	OGG1	3	psiblast	1ko9A_	191
YML061C	PIF1	1	unassigned	na	133
YML061C	PIF1	2	psiblast	1fts__	103
YML061C	PIF1	3	psiblast	1uaaA_	279
YML061C	PIF1	4	psiblast	1uaaA_	119

YML061C	PIF1	5	msa	na	144
YML061C	PIF1	6	unassigned	na	81
YML062C	MFT1	1	msa	na	72
YML062C	MFT1	2	fold_recogniti	1l8mA	130
YML062C	MFT1	3	fold_recogniti	1l8mA	79
YML062C	MFT1	4	fold_recogniti	1l8mA	111
YML063W	RPS1B	1	pfam	PF01015	255
YML064C	TEM1	1	psiblast	1g17A_	245
YML065W	ORC1	1	pfam	PF01426	198
YML065W	ORC1	2	msa	na	127
YML065W	ORC1	3	unassigned	na	84
YML065W	ORC1	4	psiblast	1e32A_	217
YML065W	ORC1	5	psiblast	1e32A_	132
YML065W	ORC1	6	unassigned	na	156
YML066C	SMA2	1	unassigned	na	369
YML067C	ERV41	1	unassigned	na	99
YML067C	ERV41	2	msa	na	253
YML068W	ITT1	1	msa	na	77
YML068W	ITT1	2	unassigned	na	76
YML068W	ITT1	3	msa	na	98
YML068W	ITT1	4	pfam	PF01485	87
YML068W	ITT1	5	pfam	PF01485	126
YML069W	POB3	1	unassigned	na	208
YML069W	POB3	2	pfam	PF03531	267
YML069W	POB3	3	unassigned	na	77
YML070W	DAK1	1	fold_recogniti	1itwA	352
YML070W	DAK1	2	fold_recogniti	1itwA	232
YML071C	COG8	1	psiblast	1i84S_	306
YML071C	COG8	2	fold_recogniti	1k83A	301
YML072C	TCB3	1	unassigned	na	360
YML072C	TCB3	2	msa	na	121
YML072C	TCB3	3	psiblast	1rsy__	152
YML072C	TCB3	4	msa	na	136
YML072C	TCB3	5	psiblast	1dsyA_	146
YML072C	TCB3	6	unassigned	na	64
YML072C	TCB3	7	psiblast	1dqvA_	137
YML072C	TCB3	8	psiblast	1dqvA_	170
YML072C	TCB3	9	unassigned	na	116
YML072C	TCB3	10	msa	na	143
YML073C	RPL6A	1	msa	na	66
YML073C	RPL6A	2	pfam	PF01159	110
YML074C	FPR3	1	fold_recogniti	1l8mA	89
YML074C	FPR3	2	fold_recogniti	1l8mA	79
YML074C	FPR3	3	fold_recogniti	1l8mA	70
YML074C	FPR3	4	fold_recogniti	1l8mA	69
YML074C	FPR3	5	psiblast	1yat__	104
YML075C	HMG1	1	msa	na	524
YML075C	HMG1	2	unassigned	na	53

YML075C	HMG1	3	psiblast	1dq8A_	112
YML075C	HMG1	4	psiblast	1dq8A_	263
YML075C	HMG1	5	psiblast	1dq8A_	102
YML076C	WAR1	1	unassigned	na	944
YML077W	BET5	1	unassigned	na	159
YML078W	CPR3	1	psiblast	1ak4A_	182
YML079W	YML079W	1	msa	na	132
YML079W	YML079W	2	msa	na	69
YML080W	DUS1	1	psiblast	1gox__	365
YML080W	DUS1	2	unassigned	na	58
YML081C-A	ATP18	1	unassigned	na	59
YML081W	YML081W	1	psiblast	2adr__	155
YML081W	YML081W	2	psiblast	2drpA_	62
YML081W	YML081W	3	msa	na	58
YML081W	YML081W	4	msa	na	129
YML081W	YML081W	5	unassigned	na	64
YML081W	YML081W	6	msa	na	783
YML082W	YML082W	1	msa	na	184
YML082W	YML082W	2	psiblast	1qgnA_	342
YML082W	YML082W	3	psiblast	1qgnA_	123
YML083C	YML083C	1	unassigned	na	418
YML084W	YML084W	1	unassigned	na	102
YML085C	TUB1	1	psiblast	1ffxA_	447
YML086C	ALO1	1	psiblast	1i19A_	284
YML086C	ALO1	2	psiblast	1i19A_	242
YML087C	YML087C	1	psiblast	1vhbA_	61
YML087C	YML087C	2	psiblast	1cnf__	106
YML087C	YML087C	3	psiblast	1cnf__	145
YML088W	UFO1	1	psiblast	1fs2A_	102
YML088W	UFO1	2	psiblast	1erjA_	247
YML088W	UFO1	3	unassigned	na	319
YML089C	YML089C	1	unassigned	na	122
YML090W	YML090W	1	unassigned	na	128
YML091C	RPM2	1	unassigned	na	1202
YML092C	PRE8	1	psiblast	1fntP_	250
YML093W	UTP14	1	psiblast	1i84S_	320
YML093W	UTP14	2	msa	na	496
YML093W	UTP14	3	unassigned	na	83
YML094C-A	YML094C-A	1	unassigned	na	133
YML094W	GIM5	1	psiblast	1fxkC_	83
YML094W	GIM5	2	psiblast	1fxkC_	80
YML095C	RAD10	1	unassigned	na	73
YML095C	RAD10	2	pfam	PF03834	137
YML096W	YML096W	1	psiblast	1ct9A_	203
YML096W	YML096W	2	psiblast	1ct9A_	322
YML097C	VPS9	1	fold_recogniti	1n1bA	451
YML098W	TAF13	1	psiblast	1bh8A_	54
YML098W	TAF13	2	pfam	PF02269	113

YML099C	ARG81	1	psiblast	1pyiA_	69
YML099C	ARG81	2	unassigned	na	811
YML099W-A	YML099W-A	1	unassigned	na	109
YML100W	TSL1	1	msa	na	251
YML100W	TSL1	2	pfam	PF00982	558
YML100W	TSL1	3	pfam	PF02358	289
YML101C	CUE4	1	unassigned	na	53
YML101C	CUE4	2	pfam	PF02845	64
YML101C-A	YML101C-A	1	unassigned	na	105
YML102W	CAC2	1	pfam	PF00400	48
YML102W	CAC2	2	psiblast	1erjA_	420
YML103C	NUP188	1	unassigned	na	1655
YML104C	MDM1	1	unassigned	na	80
YML104C	MDM1	2	pfam	PF02194	214
YML104C	MDM1	3	unassigned	na	94
YML104C	MDM1	4	fold_recogniti	1agrE	219
YML104C	MDM1	5	unassigned	na	89
YML104C	MDM1	6	psiblast	1h6hA_	224
YML104C	MDM1	7	msa	na	207
YML105C	SEC65	1	unassigned	na	81
YML105C	SEC65	2	psiblast	1jidA_	119
YML105C	SEC65	3	unassigned	na	73
YML106W	URA5	1	psiblast	1lh0A_	226
YML107C	YML107C	1	unassigned	na	386
YML108W	YML108W	1	unassigned	na	105
YML109W	ZDS2	1	unassigned	na	131
YML109W	ZDS2	2	fold_recogniti	1jv2B	385
YML109W	ZDS2	3	fold_recogniti	1jv2B	84
YML109W	ZDS2	4	fold_recogniti	1m2vB	219
YML109W	ZDS2	5	unassigned	na	123
YML110C	COQ5	1	psiblast	1kpiA_	307
YML111W	BUL2	1	unassigned	na	97
YML111W	BUL2	2	msa	na	459
YML111W	BUL2	3	unassigned	na	85
YML111W	BUL2	4	msa	na	279
YML112W	CTK3	1	unassigned	na	296
YML113W	DAT1	1	unassigned	na	248
YML114C	TAF8	1	msa	na	264
YML114C	TAF8	2	unassigned	na	246
YML115C	VAN1	1	unassigned	na	121
YML115C	VAN1	2	pfam	PF03452	414
YML116W	ATR1	1	psiblast	1lviA_	490
YML116W	ATR1	2	unassigned	na	52
YML116W-A	YML116W-A	1	unassigned	na	100
YML117W	NAB6	1	fold_recogniti	1ruxA	204
YML117W	NAB6	2	fold_recogniti	1ruxA	169
YML117W	NAB6	3	fold_recogniti	1ruxA	179
YML117W	NAB6	4	fold_recogniti	1k28D	84

YML117W	NAB6	5	psiblast	1fxlA_	90
YML117W	NAB6	6	psiblast	1fxlA_	120
YML117W	NAB6	7	unassigned	na	63
YML117W	NAB6	8	msa	na	168
YML117W	NAB6	9	unassigned	na	57
YML118W	NGL3	1	psiblast	1hd7A_	407
YML118W	NGL3	2	unassigned	na	98
YML119W	YML119W	1	unassigned	na	357
YML120C	NDI1	1	psiblast	1lvl__	352
YML120C	NDI1	2	psiblast	1lvl__	161
YML121W	GTR1	1	psiblast	1g17A_	234
YML121W	GTR1	2	msa	na	76
YML122C	YML122C	1	unassigned	na	126
YML123C	PHO84	1	psiblast	1ja5A_	496
YML123C	PHO84	2	psiblast	1ja5A_	91
YML124C	TUB3	1	psiblast	1ffxA_	445
YML125C	YML125C	1	psiblast	1vhbA_	61
YML125C	YML125C	2	psiblast	1ib0A_	106
YML125C	YML125C	3	psiblast	1ib0A_	145
YML126C	ERG13	1	psiblast	1cgkA_	374
YML126C	ERG13	2	pfam	PF01154	117
YML127W	RSC9	1	unassigned	na	54
YML127W	RSC9	2	msa	na	446
YML127W	RSC9	3	unassigned	na	81
YML128C	MSC1	1	msa	na	395
YML128C	MSC1	2	unassigned	na	118
YML129C	COX14	1	unassigned	na	70
YML130C	ERO1	1	unassigned	na	173
YML130C	ERO1	2	fold_recogniti	1n1bA	390
YML131W	YML131W	1	psiblast	1qorA_	183
YML131W	YML131W	2	psiblast	1qorA_	182
YML133C	YML133C	1	msa	na	209
YML133C	YML133C	2	msa	na	93
YML133C	YML133C	3	psiblast	1fuuA_	487
YML133C	YML133C	4	unassigned	na	88
YML133C	YML133C	5	psiblast	1ezgA_	125
YML133C	YML133C	6	unassigned	na	372
YMR001C	CDC5	1	psiblast	1ctpE_	301
YMR001C	CDC5	2	psiblast	1ctpE_	81
YMR001C	CDC5	3	psiblast	1ahr__	73
YMR001C	CDC5	4	psiblast	1ahr__	64
YMR001C	CDC5	5	fold_recogniti	1mbyA	83
YMR001C	CDC5	6	unassigned	na	103
YMR002W	YMR002W	1	unassigned	na	156
YMR003W	YMR003W	1	psiblast	1jeqA_	106
YMR003W	YMR003W	2	unassigned	na	92
YMR004W	MVP1	1	unassigned	na	84
YMR004W	MVP1	2	psiblast	1h6hA_	159

YMR004W	MVP1	3	psiblast	1dg3A_	145
YMR004W	MVP1	4	psiblast	1dg3A_	123
YMR005W	TAF4	1	unassigned	na	96
YMR005W	TAF4	2	psiblast	1i84S_	222
YMR005W	TAF4	3	unassigned	na	70
YMR006C	PLB2	1	psiblast	1cjyA_	465
YMR006C	PLB2	2	unassigned	na	241
YMR007W	YMR007W	1	unassigned	na	126
YMR008C	PLB1	1	psiblast	1cjyA_	364
YMR008C	PLB1	2	fold_recogniti	1cjyA	228
YMR008C	PLB1	3	unassigned	na	72
YMR009W	YMR009W	1	unassigned	na	70
YMR009W	YMR009W	2	fold_recogniti	1l3jA	109
YMR010W	YMR010W	1	msa	na	167
YMR010W	YMR010W	2	unassigned	na	76
YMR010W	YMR010W	3	msa	na	162
YMR011W	HXT2	1	psiblast	1ja5A_	463
YMR011W	HXT2	2	psiblast	1ja5A_	78
YMR012W	CLU1	1	msa	na	716
YMR012W	CLU1	2	unassigned	na	86
YMR012W	CLU1	3	psiblast	1fchA_	189
YMR012W	CLU1	4	psiblast	1fchA_	85
YMR012W	CLU1	5	psiblast	1fchA_	78
YMR012W	CLU1	6	psiblast	1fchA_	123
YMR013C	SEC59	1	fold_recogniti	1k04A	59
YMR013C	SEC59	2	fold_recogniti	1n1bA	125
YMR013C	SEC59	3	fold_recogniti	1n1bA	199
YMR013C	SEC59	4	pfam	PF01879	136
YMR013W-A	YMR013W-A	1	unassigned	na	26
YMR014W	BUD22	1	unassigned	na	110
YMR014W	BUD22	2	msa	na	126
YMR014W	BUD22	3	psiblast	1i84S_	283
YMR015C	ERG5	1	unassigned	na	80
YMR015C	ERG5	2	psiblast	1jo9A_	143
YMR015C	ERG5	3	psiblast	1jo9A_	166
YMR015C	ERG5	4	psiblast	1jo9A_	149
YMR016C	SOK2	1	fold_recogniti	1deqA	125
YMR016C	SOK2	2	fold_recogniti	1ei3A	289
YMR016C	SOK2	3	psiblast	1bm8__	95
YMR016C	SOK2	4	fold_recogniti	1ldvA	276
YMR017W	SPO20	1	unassigned	na	139
YMR017W	SPO20	2	psiblast	1jthA_	111
YMR017W	SPO20	3	psiblast	1sfcD_	147
YMR018W	YMR018W	1	msa	na	78
YMR018W	YMR018W	2	unassigned	na	75
YMR018W	YMR018W	3	psiblast	1fchA_	148
YMR018W	YMR018W	4	psiblast	1fchA_	73
YMR018W	YMR018W	5	psiblast	1fchA_	60

YMR018W	YMR018W	6	psiblast	1fchA_	80
YMR019W	STB4	1	unassigned	na	67
YMR019W	STB4	2	psiblast	1pyiA_	155
YMR019W	STB4	3	unassigned	na	727
YMR020W	FMS1	1	psiblast	1b37A_	353
YMR020W	FMS1	2	psiblast	1b37A_	155
YMR021C	MAC1	1	psiblast	1co4A_	61
YMR021C	MAC1	2	unassigned	na	356
YMR022W	QRI8	1	psiblast	2ucz__	165
YMR023C	MSS1	1	msa	na	87
YMR023C	MSS1	2	msa	na	177
YMR023C	MSS1	3	psiblast	1egaA_	262
YMR024W	MRPL3	1	msa	na	106
YMR024W	MRPL3	2	psiblast	1jfzA_	193
YMR024W	MRPL3	3	psiblast	1di2A_	91
YMR025W	CSI1	1	unassigned	na	295
YMR026C	PEX12	1	unassigned	na	65
YMR026C	PEX12	2	psiblast	1fbvA_	106
YMR026C	PEX12	3	psiblast	1fbvA_	162
YMR026C	PEX12	4	psiblast	1fbvA_	66
YMR027W	YMR027W	1	msa	na	130
YMR027W	YMR027W	2	unassigned	na	69
YMR027W	YMR027W	3	msa	na	271
YMR028W	TAP42	1	msa	na	90
YMR028W	TAP42	2	unassigned	na	276
YMR029C	FAR8	1	msa	na	120
YMR029C	FAR8	2	unassigned	na	403
YMR030W	RSF1	1	unassigned	na	376
YMR031C	YMR031C	1	msa	na	381
YMR031C	YMR031C	2	unassigned	na	64
YMR031C	YMR031C	3	psiblast	1i84S_	398
YMR031W-A	YMR031W-A	1	unassigned	na	108
YMR032W	HOF1	1	psiblast	1i84S_	241
YMR032W	HOF1	2	msa	na	257
YMR032W	HOF1	3	msa	na	96
YMR032W	HOF1	4	psiblast	1ark__	75
YMR033W	ARP9	1	psiblast	1yagA_	242
YMR033W	ARP9	2	psiblast	1yagA_	225
YMR034C	YMR034C	1	msa	na	352
YMR034C	YMR034C	2	unassigned	na	82
YMR035W	IMP2	1	psiblast	1kn9A_	177
YMR036C	MIH1	1	unassigned	na	66
YMR036C	MIH1	2	msa	na	110
YMR036C	MIH1	3	psiblast	1cwrA_	280
YMR036C	MIH1	4	unassigned	na	98
YMR037C	MSN2	1	unassigned	na	162
YMR037C	MSN2	2	msa	na	189
YMR037C	MSN2	3	psiblast	1tf6A_	192

YMR037C	MSN2	4	psiblast	1meyG_	87
YMR037C	MSN2	5	psiblast	1jk1A_	74
YMR038C	LYS7	1	psiblast	1jk9B_	104
YMR038C	LYS7	2	psiblast	1jk9B_	145
YMR039C	SUB1	1	psiblast	1pcfA_	116
YMR039C	SUB1	2	msa	na	176
YMR040W	YET2	1	unassigned	na	160
YMR041C	YMR041C	1	psiblast	1exbA_	271
YMR041C	YMR041C	2	psiblast	1exbA_	64
YMR042W	ARG80	1	psiblast	1mnmA_	177
YMR043W	MCM1	1	psiblast	1mnmA_	100
YMR043W	MCM1	2	msa	na	186
YMR044W	IOC4	1	pfam	PF00855	103
YMR044W	IOC4	2	msa	na	315
YMR044W	IOC4	3	unassigned	na	57
YMR046W-A	YMR046W-A	1	unassigned	na	42
YMR047C	NUP116	1	msa	na	510
YMR047C	NUP116	2	unassigned	na	113
YMR047C	NUP116	3	msa	na	383
YMR047C	NUP116	4	unassigned	na	107
YMR048W	CSM3	1	msa	na	174
YMR048W	CSM3	2	unassigned	na	143
YMR049C	ERB1	1	msa	na	308
YMR049C	ERB1	2	unassigned	na	60
YMR049C	ERB1	3	psiblast	1gg2B_	383
YMR049C	ERB1	4	psiblast	1erjA_	56
YMR052C-A	YMR052C-A	1	unassigned	na	121
YMR052W	FAR3	1	unassigned	na	204
YMR053C	STB2	1	unassigned	na	600
YMR053C	STB2	2	msa	na	250
YMR054W	STV1	1	unassigned	na	80
YMR054W	STV1	2	psiblast	1i84S_	328
YMR054W	STV1	3	msa	na	482
YMR055C	BUB2	1	psiblast	1fkmA_	181
YMR055C	BUB2	2	psiblast	1fkmA_	53
YMR055C	BUB2	3	unassigned	na	72
YMR056C	AAC1	1	fold_recogniti	1is2A	309
YMR057C	YMR057C	1	unassigned	na	123
YMR058W	FET3	1	psiblast	1kyaA_	352
YMR058W	FET3	2	psiblast	1kyaA_	193
YMR058W	FET3	3	unassigned	na	91
YMR059W	SEN15	1	unassigned	na	148
YMR060C	TOM37	1	unassigned	na	327
YMR061W	RNA14	1	psiblast	1hz4A_	126
YMR061W	RNA14	2	psiblast	1hz4A_	100
YMR061W	RNA14	3	psiblast	1fchA_	137
YMR061W	RNA14	4	psiblast	1fchA_	100
YMR061W	RNA14	5	psiblast	1fchA_	128

YMR061W	RNA14	6	unassigned	na	86
YMR062C	ECM40	1	fold_recogniti	1b65A	358
YMR062C	ECM40	2	unassigned	na	83
YMR063W	RIM9	1	msa	na	116
YMR063W	RIM9	2	unassigned	na	123
YMR064W	AEP1	1	unassigned	na	518
YMR065W	KAR5	1	unassigned	na	108
YMR065W	KAR5	2	fold_recogniti	3btaA	291
YMR065W	KAR5	3	fold_recogniti	3btaA	105
YMR066W	SOV1	1	unassigned	na	898
YMR067C	UBX4	1	unassigned	na	126
YMR067C	UBX4	2	msa	na	99
YMR067C	UBX4	3	psiblast	1jruA_	191
YMR068W	AVO2	1	psiblast	1k1aA_	103
YMR068W	AVO2	2	psiblast	1k1aA_	126
YMR068W	AVO2	3	psiblast	1sw6A_	67
YMR068W	AVO2	4	msa	na	130
YMR069W	NAT4	1	unassigned	na	128
YMR069W	NAT4	2	psiblast	1bo4A_	157
YMR070W	MOT3	1	unassigned	na	164
YMR070W	MOT3	2	msa	na	118
YMR070W	MOT3	3	psiblast	1lu6A_	85
YMR070W	MOT3	4	psiblast	1lu6A_	123
YMR071C	TVP18	1	unassigned	na	167
YMR072W	ABF2	1	psiblast	1hme__	113
YMR072W	ABF2	2	fold_recogniti	1j46A	70
YMR073C	YMR073C	1	unassigned	na	112
YMR073C	YMR073C	2	psiblast	1b5m__	89
YMR074C	YMR074C	1	psiblast	1eijA_	145
YMR075C-A	YMR075C-A	1	unassigned	na	121
YMR075W	RCO1	1	msa	na	215
YMR075W	RCO1	2	psiblast	1fp0A_	96
YMR075W	RCO1	3	unassigned	na	111
YMR075W	RCO1	4	psiblast	1f62A_	62
YMR075W	RCO1	5	unassigned	na	200
YMR076C	PDS5	1	msa	na	698
YMR076C	PDS5	2	unassigned	na	142
YMR076C	PDS5	3	msa	na	356
YMR076C	PDS5	4	unassigned	na	81
YMR077C	VPS20	1	psiblast	1i84S_	221
YMR078C	CTF18	1	psiblast	1e32A_	122
YMR078C	CTF18	2	psiblast	1e32A_	197
YMR078C	CTF18	3	psiblast	1e32A_	122
YMR078C	CTF18	4	msa	na	213
YMR078C	CTF18	5	unassigned	na	87
YMR079W	SEC14	1	psiblast	1aua__	243
YMR079W	SEC14	2	psiblast	1aua__	61
YMR080C	NAM7	1	unassigned	na	224

YMR080C	NAM7	2	msa	na	165
YMR080C	NAM7	3	psiblast	1pjr__	249
YMR080C	NAM7	4	psiblast	1pjr__	333
YMR081C	ISF1	1	unassigned	na	338
YMR082C	YMR082C	1	unassigned	na	118
YMR083W	ADH3	1	psiblast	1e3jA_	214
YMR083W	ADH3	2	psiblast	1e3jA_	161
YMR084W	YMR084W	1	psiblast	1jxaA_	262
YMR085W	YMR085W	1	psiblast	1jxaA_	277
YMR085W	YMR085W	2	psiblast	1jxaA_	155
YMR086C-A	YMR086C-A	1	unassigned	na	112
YMR086W	YMR086W	1	fold_recogniti	1l8mA	70
YMR086W	YMR086W	2	fold_recogniti	1jl5A	346
YMR086W	YMR086W	3	unassigned	na	58
YMR086W	YMR086W	4	msa	na	277
YMR086W	YMR086W	5	msa	na	209
YMR087W	YMR087W	1	unassigned	na	284
YMR088C	YMR088C	1	psiblast	1ja5A_	360
YMR088C	YMR088C	2	psiblast	1ja5A_	63
YMR088C	YMR088C	3	msa	na	139
YMR089C	YTA12	1	unassigned	na	162
YMR089C	YTA12	2	psiblast	1e32A_	102
YMR089C	YTA12	3	psiblast	1e32A_	80
YMR089C	YTA12	4	psiblast	1e32A_	174
YMR089C	YTA12	5	psiblast	1e32A_	84
YMR089C	YTA12	6	msa	na	119
YMR089C	YTA12	7	msa	na	104
YMR090W	YMR090W	1	psiblast	1bxkB_	227
YMR091C	NPL6	1	msa	na	261
YMR091C	NPL6	2	unassigned	na	174
YMR092C	AIP1	1	psiblast	1erjA_	247
YMR092C	AIP1	2	fold_recogniti	1jjuB	119
YMR092C	AIP1	3	psiblast	1gxrA_	67
YMR092C	AIP1	4	psiblast	1c5kA_	75
YMR092C	AIP1	5	fold_recogniti	1k8kC	107
YMR093W	UTP15	1	psiblast	1gg2B_	334
YMR093W	UTP15	2	msa	na	179
YMR094W	CTF13	1	msa	na	292
YMR094W	CTF13	2	unassigned	na	186
YMR095C	SNO1	1	psiblast	1gpmA_	224
YMR096W	SNZ1	1	fold_recogniti	1pymA	194
YMR096W	SNZ1	2	psiblast	2tpsA_	103
YMR097C	MTG1	1	psiblast	1egaA_	137
YMR097C	MTG1	2	fold_recogniti	1dg3A	230
YMR098C	YMR098C	1	unassigned	na	612
YMR099C	YMR099C	1	psiblast	1l7jA_	199
YMR099C	YMR099C	2	unassigned	na	98
YMR100W	MUB1	1	msa	na	215

YMR100W	MUB1	2	unassigned	na	251
YMR100W	MUB1	3	pfam	PF01753	154
YMR101C	SRT1	1	psiblast	1jp3A_	343
YMR102C	YMR102C	1	psiblast	1k8kC_	163
YMR102C	YMR102C	2	psiblast	1erjA_	343
YMR102C	YMR102C	3	psiblast	1crzA_	118
YMR102C	YMR102C	4	msa	na	210
YMR103C	YMR103C	1	unassigned	na	120
YMR104C	YPK2	1	unassigned	na	151
YMR104C	YPK2	2	psiblast	1jnuA_	129
YMR104C	YPK2	3	psiblast	1atpE_	278
YMR104C	YPK2	4	psiblast	1atpE_	119
YMR105C	PGM2	1	psiblast	1c47A_	191
YMR105C	PGM2	2	psiblast	1c47A_	149
YMR105C	PGM2	3	psiblast	1c47A_	102
YMR105C	PGM2	4	psiblast	1c47A_	127
YMR106C	YKU80	1	psiblast	1jeqA_	297
YMR106C	YKU80	2	psiblast	1jeqA_	181
YMR106C	YKU80	3	psiblast	1jeqA_	151
YMR107W	SPG4	1	unassigned	na	115
YMR108W	ILV2	1	psiblast	1jscA_	274
YMR108W	ILV2	2	psiblast	1jscA_	199
YMR108W	ILV2	3	psiblast	1jscA_	214
YMR109W	MYO5	1	psiblast	1lkxA_	642
YMR109W	MYO5	2	psiblast	1lkxA_	72
YMR109W	MYO5	3	psiblast	1wdcA_	54
YMR109W	MYO5	4	psiblast	2tmaA_	74
YMR109W	MYO5	5	psiblast	2tmaA_	63
YMR109W	MYO5	6	psiblast	2tmaA_	52
YMR109W	MYO5	7	fold_recogniti	1ldvA	113
YMR109W	MYO5	8	psiblast	1awj__	97
YMR109W	MYO5	9	unassigned	na	52
YMR110C	YMR110C	1	psiblast	1bi9A_	330
YMR110C	YMR110C	2	psiblast	1bi9A_	202
YMR111C	YMR111C	1	unassigned	na	201
YMR111C	YMR111C	2	msa	na	261
YMR112C	MED11	1	unassigned	na	131
YMR113W	FOL3	1	psiblast	1fgs__	272
YMR113W	FOL3	2	psiblast	1fgs__	155
YMR114C	YMR114C	1	pfam	PF02586	254
YMR114C	YMR114C	2	unassigned	na	114
YMR115W	FMP24	1	unassigned	na	103
YMR115W	FMP24	2	fold_recogniti	1hz4A	107
YMR115W	FMP24	3	fold_recogniti	1hz4A	141
YMR115W	FMP24	4	fold_recogniti	1hz4A	150
YMR116C	ASC1	1	psiblast	1erjA_	319
YMR117C	SPC24	1	unassigned	na	213
YMR118C	YMR118C	1	pfam	PF01127	196

YMR119W	ASI1	1	unassigned	na	296
YMR119W	ASI1	2	psiblast	1i84S_	269
YMR119W	ASI1	3	psiblast	1fbvA_	59
YMR119W-A	YMR119W-A	1	unassigned	na	124
YMR120C	ADE17	1	psiblast	1g8mA_	197
YMR120C	ADE17	2	psiblast	1g8mA_	181
YMR120C	ADE17	3	psiblast	1g8mA_	214
YMR121C	RPL15B	1	psiblast	1k5yL_	204
YMR122C	YMR122C	1	unassigned	na	124
YMR122W-A	YMR122W-A	1	unassigned	na	84
YMR123W	PKR1	1	unassigned	na	122
YMR124W	YMR124W	1	unassigned	na	171
YMR124W	YMR124W	2	fold_recogniti	1ldvA	110
YMR124W	YMR124W	3	fold_recogniti	1ivoA	174
YMR124W	YMR124W	4	fold_recogniti	1ivoA	400
YMR124W	YMR124W	5	unassigned	na	88
YMR125W	STO1	1	psiblast	1h6kA_	293
YMR125W	STO1	2	psiblast	1h6kA_	169
YMR125W	STO1	3	psiblast	1h6kA_	93
YMR125W	STO1	4	psiblast	1h6kA_	306
YMR126C	DLT1	1	msa	na	117
YMR126C	DLT1	2	unassigned	na	225
YMR127C	SAS2	1	psiblast	1fy7A_	123
YMR127C	SAS2	2	psiblast	1fy7A_	113
YMR127C	SAS2	3	psiblast	1fy7A_	102
YMR128W	ECM16	1	psiblast	1i84S_	291
YMR128W	ECM16	2	unassigned	na	117
YMR128W	ECM16	3	psiblast	1hv8A_	176
YMR128W	ECM16	4	psiblast	1hv8A_	111
YMR128W	ECM16	5	fold_recogniti	1fuuA	158
YMR128W	ECM16	6	msa	na	414
YMR129W	POM152	1	unassigned	na	396
YMR129W	POM152	2	msa	na	247
YMR129W	POM152	3	unassigned	na	694
YMR130W	YMR130W	1	psiblast	1jud__	220
YMR130W	YMR130W	2	psiblast	1jud__	82
YMR131C	RRB1	1	msa	na	181
YMR131C	RRB1	2	psiblast	1gg2B_	330
YMR132C	JLP2	1	msa	na	145
YMR132C	JLP2	2	unassigned	na	63
YMR133W	REC114	1	pfam	PF03525	351
YMR133W	REC114	2	unassigned	na	77
YMR134W	YMR134W	1	unassigned	na	237
YMR135C	GID8	1	msa	na	350
YMR135C	GID8	2	unassigned	na	105
YMR135W-A	YMR135W-A	1	unassigned	na	177
YMR136W	GAT2	1	unassigned	na	86
YMR136W	GAT2	2	msa	na	360

YMR136W	GAT2	3	psiblast	1gnf__	114
YMR137C	PSO2	1	unassigned	na	183
YMR137C	PSO2	2	psiblast	1ddkA_	113
YMR137C	PSO2	3	psiblast	1ddkA_	110
YMR137C	PSO2	4	unassigned	na	60
YMR137C	PSO2	5	msa	na	195
YMR138W	CIN4	1	psiblast	1kshA_	191
YMR139W	RIM11	1	psiblast	1i09A_	116
YMR139W	RIM11	2	psiblast	1i09A_	254
YMR140W	SIP5	1	unassigned	na	315
YMR140W	SIP5	2	pfam	PF00462	174
YMR141C	YMR141C	1	unassigned	na	102
YMR142C	RPL13B	1	pfam	PF01294	199
YMR144W	YMR144W	1	unassigned	na	342
YMR145C	NDE1	1	psiblast	1fcdA_	102
YMR145C	NDE1	2	psiblast	1ebdA_	161
YMR145C	NDE1	3	psiblast	1ebdA_	203
YMR145C	NDE1	4	psiblast	1ebdA_	94
YMR146C	TIF34	1	psiblast	1gg2B_	347
YMR147W	YMR147W	1	unassigned	na	223
YMR148W	YMR148W	1	unassigned	na	148
YMR149W	SWP1	1	unassigned	na	286
YMR150C	IMP1	1	psiblast	1b12A_	104
YMR150C	IMP1	2	fold_recogniti	1kn9A	86
YMR151W	YIM2	1	unassigned	na	145
YMR152W	YIM1	1	psiblast	1hdyA_	131
YMR152W	YIM1	2	psiblast	1hdyA_	84
YMR152W	YIM1	3	psiblast	1hdyA_	150
YMR153C-A	YMR153C-A	1	unassigned	na	111
YMR153W	NUP53	1	fold_recogniti	1k83A	379
YMR153W	NUP53	2	unassigned	na	96
YMR154C	RIM13	1	psiblast	1kfuL_	219
YMR154C	RIM13	2	psiblast	1kfuL_	105
YMR154C	RIM13	3	psiblast	1kfuL_	131
YMR154C	RIM13	4	psiblast	1kfuL_	70
YMR154C	RIM13	5	psiblast	1df0_A:2	70
YMR154C	RIM13	6	unassigned	na	132
YMR155W	YMR155W	1	psiblast	1lviA_	271
YMR155W	YMR155W	2	unassigned	na	64
YMR155W	YMR155W	3	msa	na	212
YMR156C	TPP1	1	msa	na	238
YMR157C	FMP39	1	unassigned	na	255
YMR158C-A	YMR158C-A	1	unassigned	na	45
YMR158W	MRPS8	1	psiblast	1seiA_	90
YMR158W	MRPS8	2	psiblast	1seiA_	65
YMR158W-B	YMR158W-B	1	unassigned	na	106
YMR159C	ATG16	1	psiblast	1i84S_	150
YMR160W	YMR160W	1	unassigned	na	816

YMR161W	HLJ1	1	psiblast	1xbl__	148
YMR161W	HLJ1	2	psiblast	1exkA_	76
YMR162C	DNF3	1	unassigned	na	85
YMR162C	DNF3	2	msa	na	145
YMR162C	DNF3	3	unassigned	na	59
YMR162C	DNF3	4	msa	na	103
YMR162C	DNF3	5	psiblast	1eulA_	205
YMR162C	DNF3	6	psiblast	1eulA_	420
YMR162C	DNF3	7	psiblast	1eulA_	243
YMR162C	DNF3	8	psiblast	1eulA_	256
YMR162C	DNF3	9	msa	na	140
YMR163C	YMR163C	1	unassigned	na	160
YMR163C	YMR163C	2	fold_recogniti	1qoyA	287
YMR163C	YMR163C	3	unassigned	na	258
YMR164C	MSS11	1	unassigned	na	237
YMR164C	MSS11	2	msa	na	212
YMR164C	MSS11	3	msa	na	250
YMR164C	MSS11	4	unassigned	na	59
YMR165C	SMP2	1	unassigned	na	156
YMR165C	SMP2	2	msa	na	498
YMR165C	SMP2	3	unassigned	na	208
YMR166C	YMR166C	1	fold_recogniti	1is2A	368
YMR167W	MLH1	1	psiblast	1bknA_	212
YMR167W	MLH1	2	psiblast	1bknA_	182
YMR167W	MLH1	3	msa	na	375
YMR168C	CEP3	1	psiblast	2hapC_	81
YMR168C	CEP3	2	unassigned	na	527
YMR169C	ALD3	1	psiblast	1bi9A_	302
YMR169C	ALD3	2	psiblast	1bi9A_	204
YMR170C	ALD2	1	psiblast	1bi9A_	302
YMR170C	ALD2	2	psiblast	1bi9A_	204
YMR171C	YMR171C	1	unassigned	na	168
YMR171C	YMR171C	2	pfam	PF00622	256
YMR171C	YMR171C	3	unassigned	na	126
YMR172C-A	YMR172C-A	1	unassigned	na	127
YMR172W	HOT1	1	unassigned	na	154
YMR172W	HOT1	2	fold_recogniti	1k83A	434
YMR172W	HOT1	3	unassigned	na	131
YMR173W	DDR48	1	psiblast	1kapP_	203
YMR173W	DDR48	2	psiblast	1kapP_	227
YMR173W-A	YMR173W-A	1	fold_recogniti	1ldvA	66
YMR173W-A	YMR173W-A	2	fold_recogniti	1m2vB	328
YMR174C	PAI3	1	psiblast	1dp5B_	68
YMR175W	SIP18	1	unassigned	na	79
YMR176W	ECM5	1	unassigned	na	114
YMR176W	ECM5	2	psiblast	1kqqA_	97
YMR176W	ECM5	3	psiblast	1kqqA_	106
YMR176W	ECM5	4	unassigned	na	77

YMR176W	ECM5	5	msa	na	345
YMR176W	ECM5	6	unassigned	na	85
YMR176W	ECM5	7	psiblast	1dg3A_	62
YMR176W	ECM5	8	psiblast	1i84S_	333
YMR176W	ECM5	9	psiblast	1fp0A_	80
YMR176W	ECM5	10	unassigned	na	112
YMR177W	MMT1	1	unassigned	na	145
YMR177W	MMT1	2	pfam	PF01545	365
YMR178W	YMR178W	1	psiblast	1g8lA_	139
YMR178W	YMR178W	2	msa	na	135
YMR179W	SPT21	1	unassigned	na	758
YMR180C	CTL1	1	unassigned	na	57
YMR180C	CTL1	2	psiblast	1d8hA_	263
YMR181C	YMR181C	1	unassigned	na	154
YMR182C	RGM1	1	psiblast	1lu6A_	71
YMR182C	RGM1	2	psiblast	1lu6A_	66
YMR182C	RGM1	3	msa	na	74
YMR183C	SSO2	1	psiblast	1fioA_	240
YMR183C	SSO2	2	psiblast	1fioA_	55
YMR184W	YMR184W	1	unassigned	na	198
YMR185W	YMR185W	1	unassigned	na	152
YMR185W	YMR185W	2	fold_recogniti	1qgkA	216
YMR185W	YMR185W	3	fold_recogniti	1qgkA	195
YMR185W	YMR185W	4	fold_recogniti	1qgkA	96
YMR185W	YMR185W	5	fold_recogniti	1qgkA	118
YMR185W	YMR185W	6	fold_recogniti	1qgkA	204
YMR186W	HSC82	1	psiblast	1a4h__	220
YMR186W	HSC82	2	pfam	PF00183	485
YMR187C	YMR187C	1	unassigned	na	431
YMR188C	MRPS17	1	psiblast	1qd7I_	112
YMR188C	MRPS17	2	unassigned	na	125
YMR189W	GCV2	1	unassigned	na	111
YMR189W	GCV2	2	psiblast	1jf9A_	149
YMR189W	GCV2	3	psiblast	1jf9A_	279
YMR189W	GCV2	4	psiblast	1qgnA_	324
YMR189W	GCV2	5	psiblast	1qgnA_	171
YMR190C	SGS1	1	unassigned	na	134
YMR190C	SGS1	2	msa	na	520
YMR190C	SGS1	3	psiblast	1hv8A_	213
YMR190C	SGS1	4	psiblast	1hv8A_	150
YMR190C	SGS1	5	psiblast	1d2mA_	139
YMR190C	SGS1	6	unassigned	na	113
YMR190C	SGS1	7	psiblast	1d8bA_	104
YMR190C	SGS1	8	unassigned	na	74
YMR191W	SPG5	1	msa	na	255
YMR191W	SPG5	2	unassigned	na	203
YMR192W	APP2	1	fold_recogniti	1llwA	247
YMR192W	APP2	2	psiblast	1fkmA_	110

YMR192W	APP2	3	psiblast	1i84S_	363
YMR193C-A	YMR193C-A	1	unassigned	na	128
YMR193W	MRPL24	1	pfam	PF00830	169
YMR193W	MRPL24	2	unassigned	na	89
YMR194C-A	YMR194C-A	1	unassigned	na	74
YMR194W	RPL36A	1	pfam	PF01158	100
YMR195W	ICY1	1	unassigned	na	127
YMR196W	YMR196W	1	unassigned	na	751
YMR196W	YMR196W	2	msa	na	254
YMR196W	YMR196W	3	unassigned	na	83
YMR197C	VTI1	1	psiblast	1i84S_	121
YMR197C	VTI1	2	psiblast	1gl2C_	96
YMR198W	CIK1	1	psiblast	1i84S_	362
YMR198W	CIK1	2	psiblast	2tmaA_	44
YMR198W	CIK1	3	psiblast	2tmaA_	117
YMR198W	CIK1	4	unassigned	na	71
YMR199W	CLN1	1	psiblast	1vin__	238
YMR199W	CLN1	2	psiblast	1vin__	148
YMR199W	CLN1	3	unassigned	na	160
YMR200W	ROT1	1	unassigned	na	256
YMR201C	RAD14	1	pfam	PF01286	186
YMR201C	RAD14	2	psiblast	1xpa__	185
YMR202W	ERG2	1	msa	na	222
YMR203W	TOM40	1	msa	na	158
YMR203W	TOM40	2	unassigned	na	69
YMR203W	TOM40	3	msa	na	160
YMR204C	YMR204C	1	unassigned	na	420
YMR205C	PFK2	1	unassigned	na	185
YMR205C	PFK2	2	psiblast	6pfkA_	235
YMR205C	PFK2	3	psiblast	6pfkA_	128
YMR205C	PFK2	4	psiblast	1kzhA_	279
YMR205C	PFK2	5	fold_recogniti	1pfkA	132
YMR206W	YMR206W	1	unassigned	na	313
YMR207C	HFA1	1	psiblast	1bncA_	100
YMR207C	HFA1	2	psiblast	1bncA_	166
YMR207C	HFA1	3	psiblast	1bncA_	262
YMR207C	HFA1	4	unassigned	na	79
YMR207C	HFA1	5	psiblast	1dczA_	104
YMR207C	HFA1	6	unassigned	na	159
YMR207C	HFA1	7	msa	na	177
YMR207C	HFA1	8	msa	na	180
YMR207C	HFA1	9	msa	na	196
YMR207C	HFA1	10	msa	na	623
YMR207C	HFA1	11	unassigned	na	77
YMR208W	ERG12	1	psiblast	1kvkA_	239
YMR208W	ERG12	2	psiblast	1kvkA_	204
YMR209C	YMR209C	1	unassigned	na	201
YMR209C	YMR209C	2	msa	na	256

YMR210W	YMR210W	1	unassigned	na	119
YMR210W	YMR210W	2	psiblast	1a8s__	330
YMR211W	DML1	1	msa	na	79
YMR211W	DML1	2	psiblast	1ffxB_	396
YMR212C	EFR3	1	msa	na	416
YMR212C	EFR3	2	unassigned	na	366
YMR213W	CEF1	1	psiblast	1mseC_	61
YMR213W	CEF1	2	psiblast	1mseC_	56
YMR213W	CEF1	3	fold_recogniti	1cii_	473
YMR214W	SCJ1	1	psiblast	1xbl__	168
YMR214W	SCJ1	2	psiblast	1exkA_	78
YMR214W	SCJ1	3	psiblast	1c3gA_	62
YMR214W	SCJ1	4	psiblast	1c3gA_	96
YMR215W	GAS3	1	fold_recogniti	1g01A	291
YMR215W	GAS3	2	unassigned	na	77
YMR215W	GAS3	3	msa	na	156
YMR216C	SKY1	1	unassigned	na	129
YMR216C	SKY1	2	psiblast	1ckiA_	243
YMR216C	SKY1	3	unassigned	na	52
YMR216C	SKY1	4	psiblast	1howA_	61
YMR216C	SKY1	5	psiblast	1howA_	75
YMR216C	SKY1	6	psiblast	1howA_	182
YMR217W	GUA1	1	psiblast	1gpmA_	203
YMR217W	GUA1	2	psiblast	1gpmA_	200
YMR217W	GUA1	3	psiblast	1gpmA_	122
YMR218C	TRS130	1	msa	na	515
YMR218C	TRS130	2	unassigned	na	587
YMR219W	ESC1	1	unassigned	na	72
YMR219W	ESC1	2	psiblast	1c1gA_	83
YMR219W	ESC1	3	psiblast	1i84S_	343
YMR219W	ESC1	4	unassigned	na	73
YMR219W	ESC1	5	psiblast	2tmaA_	58
YMR219W	ESC1	6	psiblast	2tmaA_	82
YMR219W	ESC1	7	psiblast	2tmaA_	62
YMR219W	ESC1	8	psiblast	2tmaA_	83
YMR219W	ESC1	9	msa	na	341
YMR219W	ESC1	10	unassigned	na	76
YMR219W	ESC1	11	fold_recogniti	1l8mA	69
YMR219W	ESC1	12	fold_recogniti	1m2vB	316
YMR220W	ERG8	1	psiblast	1kvkA_	209
YMR220W	ERG8	2	psiblast	1kvkA_	242
YMR221C	FMP42	1	msa	na	211
YMR221C	FMP42	2	unassigned	na	71
YMR221C	FMP42	3	msa	na	222
YMR222C	FSH2	1	psiblast	1fj2A_	223
YMR223W	UBP8	1	pfam	PF02148	130
YMR223W	UBP8	2	pfam	PF00442	49
YMR223W	UBP8	3	msa	na	219

YMR223W	UBP8	4	pfam	PF00443	73
YMR224C	MRE11	1	psiblast	1ii7A_	294
YMR224C	MRE11	2	psiblast	1ii7A_	128
YMR224C	MRE11	3	unassigned	na	270
YMR225C	MRPL44	1	unassigned	na	98
YMR226C	YMR226C	1	psiblast	1i01A_	267
YMR227C	TAF7	1	psiblast	1i84S_	152
YMR227C	TAF7	2	psiblast	1i84S_	284
YMR227C	TAF7	3	psiblast	1jchA_	154
YMR228W	MTF1	1	psiblast	1i4wA_	254
YMR228W	MTF1	2	psiblast	1i4wA_	87
YMR229C	RRP5	1	unassigned	na	69
YMR229C	RRP5	2	msa	na	162
YMR229C	RRP5	3	unassigned	na	64
YMR229C	RRP5	4	msa	na	159
YMR229C	RRP5	5	unassigned	na	96
YMR229C	RRP5	6	psiblast	1sro__	139
YMR229C	RRP5	7	psiblast	1e3pA_	119
YMR229C	RRP5	8	psiblast	1e3pA_	135
YMR229C	RRP5	9	psiblast	1e3pA_	102
YMR229C	RRP5	10	psiblast	1e3pA_	324
YMR229C	RRP5	11	psiblast	1fchA_	159
YMR229C	RRP5	12	psiblast	1fchA_	68
YMR229C	RRP5	13	psiblast	1fchA_	133
YMR230W	RPS10B	1	pfam	PF03501	105
YMR231W	PEP5	1	unassigned	na	377
YMR231W	PEP5	2	pfam	PF00637	258
YMR231W	PEP5	3	unassigned	na	230
YMR231W	PEP5	4	psiblast	1jm7B_	164
YMR232W	FUS2	1	unassigned	na	66
YMR232W	FUS2	2	psiblast	1lb1A_	255
YMR232W	FUS2	3	psiblast	1lb1A_	156
YMR232W	FUS2	4	unassigned	na	200
YMR233W	YMR233W	1	msa	na	84
YMR233W	YMR233W	2	pfam	PF02201	142
YMR234W	RNH1	1	psiblast	1qhkA_	47
YMR234W	RNH1	2	psiblast	1rt3A_	128
YMR234W	RNH1	3	psiblast	1rt3A_	173
YMR235C	RNA1	1	psiblast	1k5dC_	195
YMR235C	RNA1	2	psiblast	1k5dC_	145
YMR235C	RNA1	3	psiblast	1k14A_	67
YMR236W	TAF9	1	psiblast	1tafA_	157
YMR237W	YMR237W	1	unassigned	na	415
YMR237W	YMR237W	2	msa	na	309
YMR238W	DFG5	1	pfam	PF03663	458
YMR239C	RNT1	1	msa	na	112
YMR239C	RNT1	2	unassigned	na	68
YMR239C	RNT1	3	psiblast	1jfzA_	179

YMR239C	RNT1	4	psiblast	1ekzA_	112
YMR240C	CUS1	1	unassigned	na	279
YMR240C	CUS1	2	msa	na	157
YMR241W	YHM2	1	pfam	PF00153	107
YMR241W	YHM2	2	pfam	PF00153	99
YMR241W	YHM2	3	pfam	PF00153	108
YMR242C	RPL20A	1	pfam	PF01775	180
YMR243C	ZRC1	1	pfam	PF01545	442
YMR244C-A	YMR244C-A	1	unassigned	na	104
YMR244W	YMR244W	1	pfam	PF03856	355
YMR245W	YMR245W	1	unassigned	na	206
YMR246W	FAA4	1	psiblast	1ba3__	360
YMR246W	FAA4	2	psiblast	1ba3__	334
YMR247C	YMR247C	1	msa	na	350
YMR247C	YMR247C	2	unassigned	na	1099
YMR247C	YMR247C	3	psiblast	1e4uA_	113
YMR250W	GAD1	1	psiblast	1js3A_	85
YMR250W	GAD1	2	psiblast	1js3A_	500
YMR251W	YMR251W	1	psiblast	1eemA_	190
YMR251W	YMR251W	2	unassigned	na	176
YMR251W-A	HOR7	1	unassigned	na	59
YMR252C	YMR252C	1	unassigned	na	134
YMR253C	YMR253C	1	unassigned	na	53
YMR253C	YMR253C	2	pfam	PF00892	176
YMR253C	YMR253C	3	pfam	PF00892	185
YMR254C	YMR254C	1	unassigned	na	102
YMR255W	GFD1	1	unassigned	na	188
YMR256C	COX7	1	unassigned	na	60
YMR257C	PET111	1	unassigned	na	511
YMR257C	PET111	2	msa	na	289
YMR258C	YMR258C	1	unassigned	na	553
YMR259C	YMR259C	1	msa	na	162
YMR259C	YMR259C	2	unassigned	na	142
YMR259C	YMR259C	3	msa	na	585
YMR259C	YMR259C	4	msa	na	142
YMR259C	YMR259C	5	unassigned	na	389
YMR260C	TIF11	1	psiblast	1d7qA_	153
YMR261C	TPS3	1	msa	na	250
YMR261C	TPS3	2	pfam	PF00982	525
YMR261C	TPS3	3	pfam	PF02358	279
YMR262W	YMR262W	1	pfam	PF01026	313
YMR263W	SAP30	1	unassigned	na	201
YMR264W	CUE1	1	pfam	PF02845	109
YMR264W	CUE1	2	unassigned	na	94
YMR265C	YMR265C	1	msa	na	258
YMR265C	YMR265C	2	unassigned	na	203
YMR266W	RSN1	1	msa	na	206
YMR266W	RSN1	2	unassigned	na	87

YMR266W	RSN1	3	pfam	PF02714	484
YMR266W	RSN1	4	unassigned	na	176
YMR267W	PPA2	1	psiblast	1e6aA_	310
YMR268C	PRP24	1	psiblast	1cvjA_	112
YMR268C	PRP24	2	psiblast	1l3kA_	89
YMR268C	PRP24	3	psiblast	1l3kA_	99
YMR268C	PRP24	4	psiblast	1qm9A_	144
YMR269W	YMR269W	1	unassigned	na	142
YMR270C	RRN9	1	unassigned	na	365
YMR271C	URA10	1	psiblast	1lh0A_	227
YMR272C	SCS7	1	psiblast	1bfx__	112
YMR272C	SCS7	2	psiblast	1ndh__	66
YMR272C	SCS7	3	msa	na	206
YMR273C	ZDS1	1	unassigned	na	247
YMR273C	ZDS1	2	msa	na	604
YMR273C	ZDS1	3	unassigned	na	64
YMR274C	RCE1	1	unassigned	na	89
YMR274C	RCE1	2	pfam	PF02517	226
YMR275C	BUL1	1	msa	na	98
YMR275C	BUL1	2	msa	na	470
YMR275C	BUL1	3	unassigned	na	84
YMR275C	BUL1	4	msa	na	324
YMR276W	DSK2	1	psiblast	1bt0A_	107
YMR276W	DSK2	2	msa	na	152
YMR276W	DSK2	3	unassigned	na	70
YMR276W	DSK2	4	psiblast	1f4iA_	44
YMR277W	FCP1	1	unassigned	na	138
YMR277W	FCP1	2	pfam	PF03031	277
YMR277W	FCP1	3	unassigned	na	74
YMR277W	FCP1	4	psiblast	1jnxX_	152
YMR277W	FCP1	5	msa	na	91
YMR278W	YMR278W	1	psiblast	1c47A_	224
YMR278W	YMR278W	2	psiblast	1c47A_	153
YMR278W	YMR278W	3	psiblast	1c47A_	114
YMR278W	YMR278W	4	psiblast	1c47A_	131
YMR279C	YMR279C	1	psiblast	1ja5A_	473
YMR279C	YMR279C	2	psiblast	1ja5A_	67
YMR280C	CAT8	1	psiblast	1d66A_	192
YMR280C	CAT8	2	msa	na	140
YMR280C	CAT8	3	msa	na	521
YMR280C	CAT8	4	unassigned	na	580
YMR281W	GPI12	1	msa	na	250
YMR281W	GPI12	2	unassigned	na	54
YMR282C	AEP2	1	msa	na	128
YMR282C	AEP2	2	unassigned	na	452
YMR283C	RIT1	1	msa	na	324
YMR283C	RIT1	2	unassigned	na	189
YMR284W	YKU70	1	psiblast	1jeqA_	412

YMR284W	YKU70	2	psiblast	1jeqA_	190
YMR285C	NGL2	1	psiblast	1hd7A_	358
YMR285C	NGL2	2	msa	na	157
YMR286W	MRPL33	1	psiblast	1bxyA_	86
YMR287C	MSU1	1	unassigned	na	434
YMR287C	MSU1	2	pfam	PF00773	418
YMR287C	MSU1	3	unassigned	na	117
YMR288W	HSH155	1	unassigned	na	71
YMR288W	HSH155	2	msa	na	102
YMR288W	HSH155	3	unassigned	na	80
YMR288W	HSH155	4	psiblast	1b3uA_	110
YMR288W	HSH155	5	psiblast	1qgkA_	384
YMR288W	HSH155	6	psiblast	1qgkA_	224
YMR289W	ABZ2	1	psiblast	1i1kA_	215
YMR289W	ABZ2	2	psiblast	1i1kA_	159
YMR290C	HAS1	1	psiblast	1hv8A_	259
YMR290C	HAS1	2	psiblast	1hv8A_	151
YMR290C	HAS1	3	unassigned	na	95
YMR290W-A	YMR290W-A	1	unassigned	na	115
YMR291W	YMR291W	1	psiblast	1a06__	152
YMR291W	YMR291W	2	psiblast	1a06__	237
YMR291W	YMR291W	3	psiblast	1ew7C_	95
YMR291W	YMR291W	4	unassigned	na	102
YMR292W	GOT1	1	unassigned	na	138
YMR293C	YMR293C	1	psiblast	1gr8A_	464
YMR294W	JNM1	1	unassigned	na	373
YMR294W-A	YMR294W-A	1	unassigned	na	119
YMR295C	YMR295C	1	unassigned	na	197
YMR296C	LCB1	1	unassigned	na	85
YMR296C	LCB1	2	psiblast	1fc4A_	215
YMR296C	LCB1	3	psiblast	1fc4A_	258
YMR297W	PRC1	1	unassigned	na	78
YMR297W	PRC1	2	psiblast	1ysc__	245
YMR297W	PRC1	3	psiblast	1ysc__	209
YMR298W	YMR298W	1	unassigned	na	150
YMR299C	DYN3	1	unassigned	na	137
YMR299C	DYN3	2	msa	na	175
YMR300C	ADE4	1	psiblast	1ecbA_	323
YMR300C	ADE4	2	psiblast	1ecbA_	187
YMR301C	ATM1	1	unassigned	na	67
YMR301C	ATM1	2	psiblast	1jsqA_	329
YMR301C	ATM1	3	psiblast	1jsqA_	294
YMR302C	PRP12	1	msa	na	178
YMR302C	PRP12	2	pfam	PF00076	158
YMR302C	PRP12	3	unassigned	na	514
YMR303C	ADH2	1	psiblast	1e3jA_	187
YMR303C	ADH2	2	psiblast	1e3jA_	161
YMR304C-A	YMR304C-A	1	unassigned	na	116

YMR304W	UBP15	1	pfam	PF00917	195
YMR304W	UBP15	2	pfam	PF00442	46
YMR304W	UBP15	3	msa	na	196
YMR304W	UBP15	4	pfam	PF00443	104
YMR304W	UBP15	5	msa	na	344
YMR304W	UBP15	6	unassigned	na	345
YMR305C	SCW10	1	msa	na	101
YMR305C	SCW10	2	psiblast	1aq0A_	288
YMR306C-A	YMR306C-A	1	unassigned	na	129
YMR306W	FKS3	1	unassigned	na	142
YMR306W	FKS3	2	msa	na	528
YMR306W	FKS3	3	pfam	PF02364	907
YMR306W	FKS3	4	msa	na	208
YMR307W	GAS1	1	pfam	PF03198	342
YMR307W	GAS1	2	msa	na	217
YMR308C	PSE1	1	psiblast	1qgkA_	246
YMR308C	PSE1	2	psiblast	1qgkA_	193
YMR308C	PSE1	3	psiblast	1qgkA_	95
YMR308C	PSE1	4	psiblast	1qgkA_	98
YMR308C	PSE1	5	psiblast	1qgkA_	278
YMR308C	PSE1	6	msa	na	179
YMR309C	NIP1	1	msa	na	646
YMR309C	NIP1	2	unassigned	na	60
YMR309C	NIP1	3	pfam	PF01399	106
YMR310C	YMR310C	1	psiblast	1k3rA_	198
YMR310C	YMR310C	2	psiblast	1k3rA_	119
YMR311C	GLC8	1	unassigned	na	88
YMR311C	GLC8	2	msa	na	141
YMR312W	ELP6	1	unassigned	na	273
YMR313C	TGL3	1	msa	na	185
YMR313C	TGL3	2	pfam	PF01734	227
YMR313C	TGL3	3	unassigned	na	230
YMR314W	PRE5	1	psiblast	1fntF_	234
YMR315W	YMR315W	1	psiblast	1evjA_	154
YMR315W	YMR315W	2	psiblast	1evjA_	195
YMR316C-A	YMR316C-A	1	unassigned	na	103
YMR316C-B	YMR316C-B	1	unassigned	na	102
YMR316W	DIA1	1	unassigned	na	336
YMR317W	YMR317W	1	msa	na	362
YMR317W	YMR317W	2	unassigned	na	86
YMR317W	YMR317W	3	msa	na	606
YMR317W	YMR317W	4	unassigned	na	86
YMR318C	ADH6	1	psiblast	1e3jA_	200
YMR318C	ADH6	2	psiblast	1e3jA_	160
YMR319C	FET4	1	fold_recogniti	1itwA	189
YMR319C	FET4	2	fold_recogniti	1itwA	140
YMR319C	FET4	3	unassigned	na	223
YMR320W	YMR320W	1	unassigned	na	101

YMR321C	YMR321C	1	unassigned	na	105
YMR322C	SNO4	1	psiblast	1g2iA_	237
YMR323W	YMR323W	1	psiblast	1els__	127
YMR323W	YMR323W	2	psiblast	1els__	310
YMR324C	YMR324C	1	unassigned	na	80
YMR325W	YMR325W	1	pfam	PF00660	124
YMR326C	YMR326C	1	unassigned	na	102
YNL001W	DOM34	1	psiblast	1dt9A_	135
YNL001W	DOM34	2	psiblast	1dt9A_	140
YNL001W	DOM34	3	psiblast	1dt9A_	111
YNL002C	RLP7	1	unassigned	na	136
YNL002C	RLP7	2	psiblast	1k5yF_	186
YNL003C	PET8	1	fold_recogniti	1is2A	284
YNL004W	HRB1	1	psiblast	1cvjA_	123
YNL004W	HRB1	2	psiblast	1l3kA_	99
YNL004W	HRB1	3	psiblast	1l3kA_	115
YNL004W	HRB1	4	psiblast	2mssA_	92
YNL005C	MRP7	1	psiblast	1lnrU_	120
YNL005C	MRP7	2	unassigned	na	251
YNL006W	LST8	1	psiblast	1erjA_	303
YNL007C	SIS1	1	psiblast	1bqz__	79
YNL007C	SIS1	2	fold_recogniti	1la5A	101
YNL007C	SIS1	3	psiblast	1c3gA_	77
YNL007C	SIS1	4	psiblast	1c3gA_	95
YNL008C	ASI3	1	msa	na	417
YNL008C	ASI3	2	unassigned	na	67
YNL008C	ASI3	3	fold_recogniti	1fbvA	132
YNL008C	ASI3	4	fold_recogniti	1fbvA	53
YNL009W	IDP3	1	psiblast	1cnzA_	420
YNL010W	YNL010W	1	psiblast	1jud__	60
YNL010W	YNL010W	2	psiblast	1eulA_	181
YNL011C	YNL011C	1	msa	na	283
YNL011C	YNL011C	2	unassigned	na	161
YNL012W	SPO1	1	unassigned	na	142
YNL012W	SPO1	2	psiblast	1cjyA_	159
YNL012W	SPO1	3	fold_recogniti	1cjyA	235
YNL013C	YNL013C	1	unassigned	na	125
YNL014W	HEF3	1	msa	na	321
YNL014W	HEF3	2	unassigned	na	97
YNL014W	HEF3	3	psiblast	1b0uA_	227
YNL014W	HEF3	4	msa	na	399
YNL015W	PBI2	1	unassigned	na	75
YNL016W	PUB1	1	psiblast	1cvjA_	152
YNL016W	PUB1	2	psiblast	1cvjA_	123
YNL016W	PUB1	3	unassigned	na	56
YNL016W	PUB1	4	psiblast	1bnyA_	122
YNL017C	YNL017C	1	unassigned	na	112
YNL018C	YNL018C	1	unassigned	na	612

YNL019C	YNL019C	1	unassigned	na	284
YNL020C	ARK1	1	psiblast	2phkA_	92
YNL020C	ARK1	2	psiblast	2phkA_	243
YNL020C	ARK1	3	psiblast	1cdlA_	79
YNL020C	ARK1	4	psiblast	1cdlA_	107
YNL020C	ARK1	5	unassigned	na	117
YNL021W	HDA1	1	psiblast	1c3pA_	236
YNL021W	HDA1	2	psiblast	1c3pA_	200
YNL021W	HDA1	3	unassigned	na	270
YNL022C	YNL022C	1	msa	na	96
YNL022C	YNL022C	2	unassigned	na	56
YNL022C	YNL022C	3	psiblast	1jg1A_	71
YNL022C	YNL022C	4	psiblast	1j4fA_	267
YNL023C	FAP1	1	fold_recogniti	1jv2B	284
YNL023C	FAP1	2	psiblast	1klo__	127
YNL023C	FAP1	3	psiblast	1jv2B_	221
YNL023C	FAP1	4	psiblast	1dcxB_	79
YNL023C	FAP1	5	fold_recogniti	1mszA	133
YNL023C	FAP1	6	unassigned	na	121
YNL024C	YNL024C	1	psiblast	1g6q1_	246
YNL025C	SSN8	1	psiblast	1jkw__	222
YNL025C	SSN8	2	psiblast	1jkw__	101
YNL026W	SAM50	1	unassigned	na	100
YNL026W	SAM50	2	msa	na	384
YNL027W	CRZ1	1	msa	na	148
YNL027W	CRZ1	2	msa	na	98
YNL027W	CRZ1	3	psiblast	1tf6A_	196
YNL027W	CRZ1	4	msa	na	117
YNL027W	CRZ1	5	psiblast	1meyG_	119
YNL028W	YNL028W	1	unassigned	na	105
YNL029C	KTR5	1	pfam	PF01793	443
YNL029C	KTR5	2	unassigned	na	79
YNL032W	SIW14	1	unassigned	na	84
YNL032W	SIW14	2	psiblast	1d5rA_	117
YNL032W	SIW14	3	psiblast	1d5rA_	80
YNL033W	YNL033W	1	unassigned	na	284
YNL034W	YNL034W	1	unassigned	na	612
YNL035C	YNL035C	1	psiblast	1gg2B_	389
YNL036W	NCE103	1	psiblast	1ddzA_	47
YNL036W	NCE103	2	psiblast	1ddzA_	174
YNL037C	IDH1	1	psiblast	1xac__	360
YNL038W	GPI15	1	unassigned	na	91
YNL038W	GPI15	2	msa	na	121
YNL039W	BDP1	1	msa	na	384
YNL039W	BDP1	2	pfam	PF00249	94
YNL039W	BDP1	3	msa	na	116
YNL040W	YNL040W	1	unassigned	na	59
YNL040W	YNL040W	2	psiblast	1qf6A_	316

YNL040W	YNL040W	3	msa	na	81
YNL041C	COG6	1	unassigned	na	119
YNL041C	COG6	2	msa	na	392
YNL041C	COG6	3	unassigned	na	95
YNL041C	COG6	4	fold_recogniti	1n1bA	233
YNL042W	BOP3	1	unassigned	na	125
YNL042W	BOP3	2	msa	na	126
YNL042W	BOP3	3	unassigned	na	145
YNL043C	YNL043C	1	unassigned	na	106
YNL044W	YIP3	1	pfam	PF03208	176
YNL045W	YNL045W	1	psiblast	1hs6A_	253
YNL045W	YNL045W	2	psiblast	1hs6A_	85
YNL045W	YNL045W	3	psiblast	1hs6A_	170
YNL045W	YNL045W	4	psiblast	1hs6A_	163
YNL046W	YNL046W	1	unassigned	na	172
YNL047C	LIT1	1	unassigned	na	121
YNL047C	LIT1	2	fold_recogniti	1i84S	168
YNL047C	LIT1	3	fold_recogniti	1i84S	115
YNL047C	LIT1	4	psiblast	1fhwA_	191
YNL047C	LIT1	5	unassigned	na	61
YNL048W	ALG11	1	unassigned	na	69
YNL048W	ALG11	2	fold_recogniti	1iirA	206
YNL048W	ALG11	3	fold_recogniti	1iirA	83
YNL048W	ALG11	4	fold_recogniti	1iirA	190
YNL049C	SFB2	1	fold_recogniti	1k83A	215
YNL049C	SFB2	2	fold_recogniti	1fe8A	328
YNL049C	SFB2	3	unassigned	na	185
YNL049C	SFB2	4	psiblast	1svq__	65
YNL049C	SFB2	5	fold_recogniti	1m2vB	83
YNL050C	YNL050C	1	unassigned	na	270
YNL051W	COG5	1	msa	na	179
YNL051W	COG5	2	msa	na	224
YNL052W	COX5A	1	psiblast	1occD_	103
YNL052W	COX5A	2	psiblast	1occD_	50
YNL053W	MSG5	1	msa	na	216
YNL053W	MSG5	2	psiblast	1mkp__	187
YNL053W	MSG5	3	msa	na	86
YNL054W	VAC7	1	unassigned	na	260
YNL054W	VAC7	2	msa	na	460
YNL054W	VAC7	3	unassigned	na	445
YNL055C	POR1	1	pfam	PF01459	283
YNL056W	YNL056W	1	psiblast	1mkp__	197
YNL057W	YNL057W	1	unassigned	na	110
YNL058C	YNL058C	1	unassigned	na	316
YNL059C	ARP5	1	psiblast	1yagA_	279
YNL059C	ARP5	2	psiblast	1i84S_	314
YNL059C	ARP5	3	msa	na	162
YNL061W	NOP2	1	msa	na	215

YNL061W	NOP2	2	msa	na	96
YNL061W	NOP2	3	psiblast	1j4fA_	307
YNL062C	GCD10	1	msa	na	324
YNL062C	GCD10	2	unassigned	na	154
YNL063W	YNL063W	1	unassigned	na	69
YNL063W	YNL063W	2	psiblast	1dusA_	245
YNL064C	YDJ1	1	psiblast	1hdj__	112
YNL064C	YDJ1	2	psiblast	1exkA_	84
YNL064C	YDJ1	3	psiblast	1c3gA_	59
YNL064C	YDJ1	4	psiblast	1c3gA_	154
YNL065W	AQR1	1	unassigned	na	83
YNL065W	AQR1	2	psiblast	1ja5A_	420
YNL065W	AQR1	3	psiblast	1ja5A_	83
YNL066W	SUN4	1	fold_recogniti	1kapP	175
YNL066W	SUN4	2	fold_recogniti	1kapP	68
YNL066W	SUN4	3	fold_recogniti	1kapP	80
YNL066W	SUN4	4	fold_recogniti	1kapP	97
YNL067W	RPL9B	1	psiblast	1k5yH_	84
YNL067W	RPL9B	2	psiblast	1k5yH_	107
YNL067W-A	YNL067W-A	1	unassigned	na	48
YNL068C	FKH2	1	fold_recogniti	1g3gA	214
YNL068C	FKH2	2	unassigned	na	123
YNL068C	FKH2	3	psiblast	1d5vA_	93
YNL068C	FKH2	4	fold_recogniti	1k83A	432
YNL069C	RPL16B	1	psiblast	1k5yM_	198
YNL070W	TOM7	1	unassigned	na	60
YNL071W	LAT1	1	psiblast	1fyc__	161
YNL071W	LAT1	2	psiblast	1ebdC_	72
YNL071W	LAT1	3	psiblast	1c4tA_	249
YNL072W	RNH201	1	psiblast	1io2A_	205
YNL072W	RNH201	2	psiblast	1io2A_	102
YNL073W	MSK1	1	psiblast	1e1oA_	204
YNL073W	MSK1	2	psiblast	1e1oA_	372
YNL074C	MLF3	1	msa	na	254
YNL074C	MLF3	2	unassigned	na	198
YNL075W	IMP4	1	unassigned	na	87
YNL075W	IMP4	2	pfam	PF01945	203
YNL076W	MKS1	1	unassigned	na	584
YNL077W	APJ1	1	psiblast	1bq0__	129
YNL077W	APJ1	2	psiblast	1exkA_	130
YNL077W	APJ1	3	psiblast	1c3gA_	198
YNL077W	APJ1	4	unassigned	na	71
YNL078W	NIS1	1	unassigned	na	407
YNL079C	TPM1	1	psiblast	2tmaA_	91
YNL079C	TPM1	2	psiblast	2tmaA_	108
YNL080C	YNL080C	1	fold_recogniti	1e6vB	275
YNL080C	YNL080C	2	fold_recogniti	1e6vB	91
YNL081C	SWS2	1	psiblast	1fjgM_	74

YNL081C	SWS2	2	psiblast	1fjgM_	69
YNL082W	PMS1	1	psiblast	1ea6A_	249
YNL082W	PMS1	2	psiblast	1ea6A_	162
YNL082W	PMS1	3	psiblast	1lwzA_	204
YNL082W	PMS1	4	unassigned	na	83
YNL082W	PMS1	5	msa	na	206
YNL083W	YNL083W	1	psiblast	1jfjA_	93
YNL083W	YNL083W	2	unassigned	na	63
YNL083W	YNL083W	3	msa	na	64
YNL083W	YNL083W	4	pfam	PF00153	119
YNL083W	YNL083W	5	pfam	PF00153	155
YNL084C	END3	1	psiblast	1eh2__	98
YNL084C	END3	2	unassigned	na	82
YNL084C	END3	3	psiblast	1iq5A_	94
YNL084C	END3	4	msa	na	75
YNL085W	MKT1	1	psiblast	1b43A_	303
YNL085W	MKT1	2	psiblast	1b43A_	69
YNL085W	MKT1	3	psiblast	1xwl__	67
YNL085W	MKT1	4	unassigned	na	123
YNL085W	MKT1	5	msa	na	268
YNL086W	YNL086W	1	unassigned	na	102
YNL087W	TCB2	1	unassigned	na	206
YNL087W	TCB2	2	psiblast	1dqvA_	156
YNL087W	TCB2	3	psiblast	1dqvA_	126
YNL087W	TCB2	4	fold_recogniti	1bynA	134
YNL087W	TCB2	5	psiblast	1k5wA_	159
YNL087W	TCB2	6	unassigned	na	141
YNL087W	TCB2	7	psiblast	1rsy__	197
YNL087W	TCB2	8	msa	na	59
YNL088W	TOP2	1	psiblast	1ei1A_	248
YNL088W	TOP2	2	psiblast	1ei1A_	157
YNL088W	TOP2	3	psiblast	1bgw__	209
YNL088W	TOP2	4	psiblast	1bgw__	225
YNL088W	TOP2	5	psiblast	1bgw__	368
YNL088W	TOP2	6	msa	na	221
YNL089C	YNL089C	1	unassigned	na	158
YNL090W	RHO2	1	psiblast	1ftn__	192
YNL091W	NST1	1	psiblast	1c1gA_	135
YNL091W	NST1	2	psiblast	1i84S_	361
YNL091W	NST1	3	msa	na	280
YNL091W	NST1	4	fold_recogniti	1ldvA	317
YNL091W	NST1	5	unassigned	na	147
YNL092W	YNL092W	1	msa	na	319
YNL092W	YNL092W	2	unassigned	na	81
YNL093W	YPT53	1	psiblast	1ek0A_	220
YNL094W	APP1	1	unassigned	na	159
YNL094W	APP1	2	msa	na	315
YNL094W	APP1	3	unassigned	na	113

YNL095C	YNL095C	1	msa	na	181
YNL095C	YNL095C	2	unassigned	na	461
YNL096C	RPS7B	1	pfam	PF01251	190
YNL097C	PHO23	1	fold_recogniti	2vsgA	162
YNL097C	PHO23	2	fold_recogniti	2vsgA	45
YNL097C	PHO23	3	unassigned	na	66
YNL097C	PHO23	4	psiblast	1f62A_	57
YNL098C	RAS2	1	psiblast	821p__	185
YNL098C	RAS2	2	msa	na	137
YNL099C	OCA1	1	psiblast	1vhrA_	238
YNL100W	YNL100W	1	unassigned	na	234
YNL101W	AVT4	1	unassigned	na	234
YNL101W	AVT4	2	pfam	PF01490	479
YNL102W	POL1	1	msa	na	338
YNL102W	POL1	2	psiblast	1qhtA_	387
YNL102W	POL1	3	psiblast	1qhtA_	230
YNL102W	POL1	4	psiblast	1qhtA_	114
YNL102W	POL1	5	psiblast	1qhtA_	230
YNL102W	POL1	6	unassigned	na	169
YNL103W	MET4	1	unassigned	na	174
YNL103W	MET4	2	fold_recogniti	1k83A	377
YNL103W	MET4	3	fold_recogniti	1m2vB	121
YNL104C	LEU4	1	fold_recogniti	1e1cA	476
YNL104C	LEU4	2	msa	na	143
YNL105W	YNL105W	1	unassigned	na	142
YNL106C	INP52	1	pfam	PF02383	475
YNL106C	INP52	2	unassigned	na	84
YNL106C	INP52	3	psiblast	1i9yA_	379
YNL106C	INP52	4	fold_recogniti	1deqA	245
YNL107W	YAF9	1	pfam	PF03366	123
YNL107W	YAF9	2	unassigned	na	103
YNL108C	YNL108C	1	psiblast	1bif__	270
YNL109W	YNL109W	1	unassigned	na	181
YNL110C	NOP15	1	psiblast	1fxlA_	86
YNL110C	NOP15	2	psiblast	1fxlA_	134
YNL111C	CYB5	1	psiblast	1eueA_	120
YNL112W	DBP2	1	unassigned	na	80
YNL112W	DBP2	2	psiblast	1hv8A_	247
YNL112W	DBP2	3	psiblast	1hv8A_	219
YNL113W	RPC19	1	psiblast	1k83K_	142
YNL114C	YNL114C	1	unassigned	na	123
YNL115C	YNL115C	1	unassigned	na	58
YNL115C	YNL115C	2	msa	na	156
YNL115C	YNL115C	3	unassigned	na	94
YNL115C	YNL115C	4	psiblast	1a88A_	282
YNL115C	YNL115C	5	unassigned	na	54
YNL116W	YNL116W	1	fold_recogniti	1ldvA	234
YNL116W	YNL116W	2	unassigned	na	71

YNL116W	YNL116W	3	fold_recogniti	1g3gA	103
YNL116W	YNL116W	4	psiblast	1chc__	114
YNL117W	MLS1	1	fold_recogniti	1d8cA	104
YNL117W	MLS1	2	psiblast	1d8cA_	317
YNL117W	MLS1	3	psiblast	1d8cA_	133
YNL118C	DCP2	1	unassigned	na	64
YNL118C	DCP2	2	psiblast	1mut__	203
YNL118C	DCP2	3	fold_recogniti	1deqA	241
YNL118C	DCP2	4	unassigned	na	79
YNL118C	DCP2	5	fold_recogniti	1l8mA	90
YNL118C	DCP2	6	fold_recogniti	1l8mA	73
YNL118C	DCP2	7	fold_recogniti	1l8mA	83
YNL118C	DCP2	8	fold_recogniti	1l8mA	70
YNL118C	DCP2	9	unassigned	na	67
YNL119W	NCS2	1	fold_recogniti	1kh1A	304
YNL119W	NCS2	2	unassigned	na	189
YNL120C	YNL120C	1	unassigned	na	161
YNL121C	TOM70	1	unassigned	na	90
YNL121C	TOM70	2	psiblast	1a17__	104
YNL121C	TOM70	3	unassigned	na	124
YNL121C	TOM70	4	psiblast	1elrA_	111
YNL121C	TOM70	5	fold_recogniti	1hxiA	69
YNL121C	TOM70	6	psiblast	1ihgA_	119
YNL122C	YNL122C	1	unassigned	na	115
YNL123W	YNL123W	1	psiblast	1lcyA_	295
YNL123W	YNL123W	2	psiblast	1lcyA_	93
YNL123W	YNL123W	3	psiblast	1fc7A_	118
YNL123W	YNL123W	4	psiblast	1ky9A_	250
YNL123W	YNL123W	5	psiblast	1ky9A_	241
YNL124W	NAF1	1	unassigned	na	126
YNL124W	NAF1	2	psiblast	1k83A_	165
YNL124W	NAF1	3	msa	na	201
YNL125C	ESBP6	1	unassigned	na	179
YNL125C	ESBP6	2	psiblast	1ja5A_	494
YNL126W	SPC98	1	unassigned	na	158
YNL126W	SPC98	2	fold_recogniti	1b89A	549
YNL126W	SPC98	3	unassigned	na	139
YNL127W	FAR11	1	unassigned	na	191
YNL127W	FAR11	2	msa	na	762
YNL128W	TEP1	1	psiblast	1d5rA_	180
YNL128W	TEP1	2	psiblast	1d5rA_	144
YNL128W	TEP1	3	unassigned	na	110
YNL129W	YNL129W	1	psiblast	1g291_	240
YNL130C	CPT1	1	unassigned	na	68
YNL130C	CPT1	2	pfam	PF01066	178
YNL130C	CPT1	3	msa	na	140
YNL131W	TOM22	1	unassigned	na	152
YNL132W	KRE33	1	unassigned	na	101

YNL132W	KRE33	2	msa	na	619
YNL132W	KRE33	3	msa	na	94
YNL132W	KRE33	4	unassigned	na	242
YNL133C	FYV6	1	unassigned	na	173
YNL134C	YNL134C	1	psiblast	1tehA_	227
YNL134C	YNL134C	2	psiblast	1tehA_	149
YNL135C	FPR1	1	psiblast	1yat__	114
YNL136W	EAF7	1	unassigned	na	121
YNL136W	EAF7	2	fold_recogniti	1ldvA	304
YNL137C	NAM9	1	psiblast	1fkaD_	148
YNL137C	NAM9	2	psiblast	1fkaD_	111
YNL137C	NAM9	3	unassigned	na	227
YNL138W	SRV2	1	fold_recogniti	1k7lA	199
YNL138W	SRV2	2	fold_recogniti	1k7lA	48
YNL138W	SRV2	3	msa	na	106
YNL138W	SRV2	4	psiblast	1k4zA_	173
YNL139C	RLR1	1	unassigned	na	1111
YNL139C	RLR1	2	psiblast	1i84S_	378
YNL139C	RLR1	3	msa	na	108
YNL140C	YNL140C	1	unassigned	na	189
YNL141W	AAH1	1	psiblast	1m7mA_	347
YNL142W	MEP2	1	pfam	PF00909	499
YNL143C	YNL143C	1	unassigned	na	130
YNL144C	YNL144C	1	unassigned	na	363
YNL144C	YNL144C	2	fold_recogniti	1l8mA	74
YNL144C	YNL144C	3	fold_recogniti	1l8mA	78
YNL144C	YNL144C	4	fold_recogniti	1l8mA	70
YNL144C	YNL144C	5	fold_recogniti	1l8mA	94
YNL144C	YNL144C	6	unassigned	na	61
YNL145W	MFA2	1	unassigned	na	38
YNL146W	YNL146W	1	unassigned	na	100
YNL147W	LSM7	1	psiblast	1d3bB_	107
YNL148C	ALF1	1	msa	na	142
YNL148C	ALF1	2	psiblast	1lplA_	112
YNL149C	YNL149C	1	fold_recogniti	1jgsA	129
YNL150W	YNL150W	1	unassigned	na	135
YNL151C	RPC31	1	unassigned	na	251
YNL152W	YNL152W	1	psiblast	1dqvA_	160
YNL152W	YNL152W	2	unassigned	na	124
YNL152W	YNL152W	3	psiblast	1i5hW_	69
YNL152W	YNL152W	4	unassigned	na	56
YNL153C	GIM3	1	unassigned	na	129
YNL154C	YCK2	1	psiblast	1csn__	134
YNL154C	YCK2	2	psiblast	1csn__	75
YNL154C	YCK2	3	psiblast	1csn__	175
YNL154C	YCK2	4	unassigned	na	162
YNL155W	YNL155W	1	pfam	PF01428	64
YNL155W	YNL155W	2	unassigned	na	210

YNL156C	NSG2	1	unassigned	na	63
YNL156C	NSG2	2	fold_recogniti	1jfaA	236
YNL157W	YNL157W	1	unassigned	na	168
YNL158W	YNL158W	1	unassigned	na	198
YNL159C	ASI2	1	unassigned	na	289
YNL160W	YGP1	1	psiblast	1agx__	250
YNL160W	YGP1	2	psiblast	1agx__	104
YNL161W	CBK1	1	unassigned	na	323
YNL161W	CBK1	2	psiblast	1lhxA_	180
YNL161W	CBK1	3	unassigned	na	133
YNL161W	CBK1	4	psiblast	1ctpE_	120
YNL162W-A	YNL162W-A	1	unassigned	na	72
YNL163C	RIA1	1	psiblast	1dar__	372
YNL163C	RIA1	2	psiblast	1dar__	156
YNL163C	RIA1	3	psiblast	1dar__	198
YNL163C	RIA1	4	fold_recogniti	1efgA	248
YNL163C	RIA1	5	psiblast	1jqsC_	73
YNL163C	RIA1	6	msa	na	63
YNL164C	IBD2	1	unassigned	na	351
YNL165W	YNL165W	1	unassigned	na	406
YNL166C	BNI5	1	fold_recogniti	1l8mA	113
YNL166C	BNI5	2	fold_recogniti	1l8mA	77
YNL166C	BNI5	3	fold_recogniti	1l8mA	76
YNL166C	BNI5	4	unassigned	na	182
YNL167C	SKO1	1	fold_recogniti	1ei3A	129
YNL167C	SKO1	2	fold_recogniti	1l8mA	79
YNL167C	SKO1	3	fold_recogniti	1l8mA	73
YNL167C	SKO1	4	fold_recogniti	1l8mA	79
YNL167C	SKO1	5	fold_recogniti	1l8mA	70
YNL167C	SKO1	6	psiblast	1dh3A_	123
YNL167C	SKO1	7	unassigned	na	94
YNL168C	FMP41	1	psiblast	1gttA_	259
YNL169C	PSD1	1	msa	na	175
YNL169C	PSD1	2	pfam	PF02666	325
YNL170W	YNL170W	1	unassigned	na	131
YNL171C	YNL171C	1	unassigned	na	122
YNL172W	APC1	1	unassigned	na	591
YNL172W	APC1	2	msa	na	854
YNL172W	APC1	3	unassigned	na	117
YNL172W	APC1	4	msa	na	83
YNL172W	APC1	5	unassigned	na	103
YNL173C	MDG1	1	fold_recogniti	1m2vB	366
YNL174W	YNL174W	1	unassigned	na	190
YNL175C	NOP13	1	psiblast	1cvjA_	112
YNL175C	NOP13	2	psiblast	1l3kA_	115
YNL175C	NOP13	3	psiblast	1l3kA_	101
YNL175C	NOP13	4	psiblast	1b7fA_	75
YNL176C	YNL176C	1	unassigned	na	76

YNL176C	YNL176C	2	fold_recogniti	1mvmA	151
YNL176C	YNL176C	3	fold_recogniti	1mvmA	173
YNL176C	YNL176C	4	fold_recogniti	1ei3A	135
YNL176C	YNL176C	5	unassigned	na	101
YNL177C	MRPL22	1	unassigned	na	124
YNL177C	MRPL22	2	psiblast	1lnrQ_	185
YNL178W	RPS3	1	psiblast	1k5xC_	91
YNL178W	RPS3	2	psiblast	1k5xC_	149
YNL179C	SRF6	1	unassigned	na	145
YNL180C	RHO5	1	psiblast	1i4dD_	279
YNL180C	RHO5	2	unassigned	na	52
YNL181W	YNL181W	1	psiblast	1gcoA_	407
YNL182C	IPI3	1	psiblast	1erjA_	167
YNL182C	IPI3	2	psiblast	1gg2B_	388
YNL183C	NPR1	1	unassigned	na	393
YNL183C	NPR1	2	psiblast	1aq1__	126
YNL183C	NPR1	3	psiblast	1aq1__	129
YNL183C	NPR1	4	psiblast	1aq1__	51
YNL183C	NPR1	5	psiblast	1aq1__	91
YNL184C	YNL184C	1	unassigned	na	108
YNL185C	MRPL19	1	psiblast	1giyL_	74
YNL185C	MRPL19	2	psiblast	1giyL_	84
YNL186W	UBP10	1	fold_recogniti	1ldvA	260
YNL186W	UBP10	2	unassigned	na	63
YNL186W	UBP10	3	pfam	PF00442	103
YNL186W	UBP10	4	msa	na	134
YNL186W	UBP10	5	unassigned	na	89
YNL186W	UBP10	6	pfam	PF00443	143
YNL187W	YNL187W	1	unassigned	na	357
YNL188W	KAR1	1	unassigned	na	96
YNL188W	KAR1	2	unassigned	na	122
YNL188W	KAR1	3	unassigned	na	111
YNL188W	KAR1	4	unassigned	na	104
YNL189W	SRP1	1	psiblast	1qgkB_	62
YNL189W	SRP1	2	psiblast	1bk5A_	143
YNL189W	SRP1	3	psiblast	1bk5A_	167
YNL189W	SRP1	4	psiblast	1bk5A_	170
YNL190W	YNL190W	1	unassigned	na	204
YNL191W	YNL191W	1	psiblast	1jxaA_	357
YNL192W	CHS1	1	psiblast	1deqA_	142
YNL192W	CHS1	2	fold_recogniti	1k83A	267
YNL192W	CHS1	3	unassigned	na	79
YNL192W	CHS1	4	fold_recogniti	1dioA	237
YNL192W	CHS1	5	fold_recogniti	1dioA	130
YNL192W	CHS1	6	unassigned	na	53
YNL192W	CHS1	7	msa	na	223
YNL193W	YNL193W	1	unassigned	na	558
YNL194C	YNL194C	1	unassigned	na	301

YNL195C	YNL195C	1	msa	na	148
YNL195C	YNL195C	2	unassigned	na	95
YNL196C	YNL196C	1	unassigned	na	298
YNL197C	WHI3	1	unassigned	na	99
YNL197C	WHI3	2	fold_recogniti	1k83A	405
YNL197C	WHI3	3	psiblast	1cvjA_	157
YNL198C	YNL198C	1	unassigned	na	100
YNL199C	GCR2	1	msa	na	157
YNL199C	GCR2	2	unassigned	na	80
YNL199C	GCR2	3	msa	na	81
YNL199C	GCR2	4	unassigned	na	216
YNL200C	YNL200C	1	psiblast	1jztA_	246
YNL201C	PSY2	1	fold_recogniti	1i2hA	162
YNL201C	PSY2	2	unassigned	na	79
YNL201C	PSY2	3	msa	na	293
YNL201C	PSY2	4	unassigned	na	65
YNL201C	PSY2	5	msa	na	119
YNL201C	PSY2	6	unassigned	na	140
YNL202W	SPS19	1	psiblast	1gcoA_	295
YNL203C	YNL203C	1	unassigned	na	203
YNL204C	SPS18	1	psiblast	1dcqA_	164
YNL204C	SPS18	2	unassigned	na	65
YNL204C	SPS18	3	msa	na	71
YNL205C	YNL205C	1	unassigned	na	140
YNL206C	RTT106	1	unassigned	na	215
YNL206C	RTT106	2	fold_recogniti	1l8mA	113
YNL206C	RTT106	3	fold_recogniti	1l8mA	79
YNL206C	RTT106	4	fold_recogniti	1l8mA	48
YNL207W	RIO2	1	msa	na	67
YNL207W	RIO2	2	psiblast	1jnk__	358
YNL208W	YNL208W	1	unassigned	na	204
YNL209W	SSB2	1	psiblast	1hx1A_	196
YNL209W	SSB2	2	psiblast	1hx1A_	116
YNL209W	SSB2	3	psiblast	1hx1A_	75
YNL209W	SSB2	4	psiblast	1ckrA_	160
YNL209W	SSB2	5	psiblast	1i84S_	66
YNL210W	MER1	1	unassigned	na	180
YNL210W	MER1	2	pfam	PF00013	90
YNL211C	YNL211C	1	unassigned	na	86
YNL212W	VID27	1	unassigned	na	364
YNL212W	VID27	2	fold_recogniti	1a0rB	318
YNL212W	VID27	3	fold_recogniti	1jjuB	100
YNL213C	YNL213C	1	msa	na	148
YNL213C	YNL213C	2	unassigned	na	66
YNL214W	PEX17	1	unassigned	na	199
YNL215W	IES2	1	fold_recogniti	1l8mA	93
YNL215W	IES2	2	fold_recogniti	1l8mA	73
YNL215W	IES2	3	fold_recogniti	1l8mA	80

YNL215W	IES2	4	fold_recogniti	1l8mA	74
YNL216W	RAP1	1	unassigned	na	103
YNL216W	RAP1	2	fold_recogniti	1imoA	166
YNL216W	RAP1	3	unassigned	na	71
YNL216W	RAP1	4	psiblast	1ignA_	133
YNL216W	RAP1	5	psiblast	1ignA_	143
YNL216W	RAP1	6	unassigned	na	211
YNL217W	YNL217W	1	psiblast	1auiA_	326
YNL218W	MGS1	1	unassigned	na	110
YNL218W	MGS1	2	psiblast	1iqpA_	99
YNL218W	MGS1	3	psiblast	1iqpA_	299
YNL218W	MGS1	4	msa	na	79
YNL219C	ALG9	1	msa	na	412
YNL219C	ALG9	2	unassigned	na	143
YNL220W	ADE12	1	psiblast	1j4bA_	141
YNL220W	ADE12	2	psiblast	1j4bA_	217
YNL220W	ADE12	3	psiblast	1j4bA_	75
YNL221C	POP1	1	msa	na	125
YNL221C	POP1	2	unassigned	na	228
YNL221C	POP1	3	msa	na	447
YNL221C	POP1	4	unassigned	na	75
YNL222W	SSU72	1	msa	na	206
YNL223W	ATG4	1	unassigned	na	122
YNL223W	ATG4	2	pfam	PF03416	323
YNL223W	ATG4	3	unassigned	na	61
YNL224C	YNL224C	1	msa	na	307
YNL224C	YNL224C	2	unassigned	na	64
YNL224C	YNL224C	3	msa	na	347
YNL224C	YNL224C	4	pfam	PF01585	49
YNL225C	CNM67	1	psiblast	1i84S_	357
YNL225C	CNM67	2	psiblast	1c1gA_	107
YNL225C	CNM67	3	msa	na	117
YNL226W	YNL226W	1	unassigned	na	136
YNL227C	JJJ1	1	psiblast	1xbl__	119
YNL227C	JJJ1	2	psiblast	1exkA_	137
YNL227C	JJJ1	3	psiblast	1i84S_	334
YNL228W	YNL228W	1	unassigned	na	258
YNL229C	URE2	1	msa	na	91
YNL229C	URE2	2	psiblast	1g6wA_	116
YNL229C	URE2	3	psiblast	1g6wA_	147
YNL230C	ELA1	1	msa	na	177
YNL230C	ELA1	2	unassigned	na	202
YNL231C	PDR16	1	psiblast	1aua__	296
YNL231C	PDR16	2	psiblast	1aua__	55
YNL232W	CSL4	1	fold_recogniti	1e3hA	292
YNL233W	BNI4	1	unassigned	na	892
YNL234W	YNL234W	1	unassigned	na	155
YNL234W	YNL234W	2	psiblast	1abwA_	210

YNL234W	YNL234W	3	unassigned	na	61
YNL235C	YNL235C	1	unassigned	na	143
YNL236W	SIN4	1	unassigned	na	457
YNL236W	SIN4	2	fold_recogniti	1qoyA	269
YNL236W	SIN4	3	fold_recogniti	1qoyA	47
YNL236W	SIN4	4	unassigned	na	201
YNL237W	YTP1	1	unassigned	na	118
YNL237W	YTP1	2	msa	na	341
YNL238W	KEX2	1	psiblast	1kn6A_	126
YNL238W	KEX2	2	psiblast	1meeA_	339
YNL238W	KEX2	3	msa	na	146
YNL238W	KEX2	4	unassigned	na	203
YNL239W	LAP3	1	psiblast	1gcb__	248
YNL239W	LAP3	2	psiblast	1gcb__	206
YNL240C	NAR1	1	psiblast	1c4aA_	491
YNL241C	ZWF1	1	psiblast	1qkiA_	202
YNL241C	ZWF1	2	psiblast	1qkiA_	303
YNL242W	ATG2	1	unassigned	na	1209
YNL242W	ATG2	2	msa	na	383
YNL243W	SLA2	1	psiblast	1i84S_	311
YNL243W	SLA2	2	psiblast	1c1gA_	69
YNL243W	SLA2	3	psiblast	1c1gA_	202
YNL243W	SLA2	4	fold_recogniti	1hciA	149
YNL243W	SLA2	5	fold_recogniti	1hciA	113
YNL243W	SLA2	6	fold_recogniti	1hciA	124
YNL244C	SUI1	1	psiblast	2if1__	108
YNL245C	CWC25	1	unassigned	na	179
YNL246W	VPS75	1	pfam	PF00956	264
YNL247W	YNL247W	1	psiblast	1lleA_	153
YNL247W	YNL247W	2	psiblast	1li5A_	266
YNL247W	YNL247W	3	psiblast	1li5A_	55
YNL247W	YNL247W	4	psiblast	1li5A_	190
YNL247W	YNL247W	5	unassigned	na	103
YNL248C	RPA49	1	unassigned	na	415
YNL249C	MPA43	1	psiblast	1bo5O_	264
YNL249C	MPA43	2	psiblast	1bo5O_	80
YNL249C	MPA43	3	psiblast	1bo5O_	198
YNL250W	RAD50	1	psiblast	1f2tA_	56
YNL250W	RAD50	2	psiblast	1i84S_	348
YNL250W	RAD50	3	unassigned	na	97
YNL250W	RAD50	4	psiblast	2tmaA_	109
YNL250W	RAD50	5	psiblast	2tmaA_	80
YNL250W	RAD50	6	psiblast	2tmaA_	62
YNL250W	RAD50	7	psiblast	2tmaA_	76
YNL250W	RAD50	8	msa	na	307
YNL250W	RAD50	9	psiblast	1f2tB_	94
YNL250W	RAD50	10	psiblast	1f2tB_	83
YNL251C	NRD1	1	msa	na	200

YNL251C	NRD1	2	psiblast	2up1A_	101
YNL251C	NRD1	3	psiblast	2up1A_	112
YNL251C	NRD1	4	psiblast	1fxlA_	95
YNL251C	NRD1	5	unassigned	na	67
YNL252C	MRPL17	1	unassigned	na	143
YNL252C	MRPL17	2	fold_recogniti	1kt9A	138
YNL253W	TEX1	1	psiblast	1gg2B_	323
YNL253W	TEX1	2	psiblast	1k8kC_	99
YNL254C	YNL254C	1	unassigned	na	401
YNL255C	GIS2	1	psiblast	1baj__	52
YNL255C	GIS2	2	psiblast	1aaf__	101
YNL256W	FOL1	1	psiblast	1dhn__	220
YNL256W	FOL1	2	unassigned	na	98
YNL256W	FOL1	3	psiblast	1dy3A_	196
YNL256W	FOL1	4	psiblast	1aj0__	179
YNL256W	FOL1	5	psiblast	1aj0__	171
YNL257C	SIP3	1	fold_recogniti	1hciA	162
YNL257C	SIP3	2	fold_recogniti	1hciA	133
YNL257C	SIP3	3	psiblast	1fhwA_	119
YNL257C	SIP3	4	psiblast	1f7cA_	130
YNL257C	SIP3	5	unassigned	na	685
YNL258C	DSL1	1	unassigned	na	754
YNL259C	ATX1	1	psiblast	1cc7A_	73
YNL260C	YNL260C	1	unassigned	na	198
YNL261W	ORC5	1	psiblast	1e32A_	191
YNL261W	ORC5	2	psiblast	1e32A_	163
YNL261W	ORC5	3	unassigned	na	125
YNL262W	POL2	1	unassigned	na	100
YNL262W	POL2	2	psiblast	1tgoA_	325
YNL262W	POL2	3	psiblast	1tgoA_	234
YNL262W	POL2	4	psiblast	1tgoA_	198
YNL262W	POL2	5	psiblast	1b1fR_	241
YNL262W	POL2	6	msa	na	134
YNL262W	POL2	7	unassigned	na	692
YNL262W	POL2	8	msa	na	298
YNL263C	YIF1	1	pfam	PF03878	314
YNL264C	PDR17	1	psiblast	1aua__	117
YNL264C	PDR17	2	psiblast	1aua__	233
YNL265C	IST1	1	pfam	PF03398	151
YNL265C	IST1	2	msa	na	81
YNL265C	IST1	3	unassigned	na	66
YNL266W	YNL266W	1	unassigned	na	139
YNL267W	PIK1	1	msa	na	121
YNL267W	PIK1	2	msa	na	313
YNL267W	PIK1	3	psiblast	1e8yA_	258
YNL267W	PIK1	4	psiblast	1e8yA_	185
YNL267W	PIK1	5	psiblast	1e8yA_	189
YNL268W	LYP1	1	unassigned	na	84

YNL268W	LYP1	2	pfam	PF00324	527
YNL269W	BSC4	1	unassigned	na	131
YNL270C	ALP1	1	pfam	PF00324	573
YNL271C	BNI1	1	psiblast	1i84S_	444
YNL271C	BNI1	2	psiblast	1almA_	67
YNL271C	BNI1	3	psiblast	1cii__	162
YNL271C	BNI1	4	psiblast	1cii__	275
YNL271C	BNI1	5	unassigned	na	63
YNL271C	BNI1	6	msa	na	196
YNL271C	BNI1	7	msa	na	618
YNL271C	BNI1	8	unassigned	na	128
YNL272C	SEC2	1	psiblast	1i84S_	302
YNL272C	SEC2	2	unassigned	na	154
YNL272C	SEC2	3	msa	na	303
YNL273W	TOF1	1	msa	na	639
YNL273W	TOF1	2	msa	na	223
YNL273W	TOF1	3	unassigned	na	376
YNL274C	YNL274C	1	psiblast	1gdhA_	159
YNL274C	YNL274C	2	psiblast	1gdhA_	191
YNL275W	YNL275W	1	psiblast	1bzkA_	163
YNL275W	YNL275W	2	msa	na	413
YNL276C	YNL276C	1	unassigned	na	131
YNL277W	MET2	1	psiblast	1azwA_	357
YNL277W	MET2	2	fold_recogniti	1iunA	129
YNL278W	CAF120	1	unassigned	na	75
YNL278W	CAF120	2	pfam	PF00169	157
YNL278W	CAF120	3	unassigned	na	99
YNL278W	CAF120	4	msa	na	729
YNL279W	PRM1	1	unassigned	na	661
YNL280C	ERG24	1	pfam	PF01222	438
YNL281W	HCH1	1	unassigned	na	153
YNL282W	POP3	1	unassigned	na	195
YNL283C	WSC2	1	pfam	PF01822	108
YNL283C	WSC2	2	unassigned	na	80
YNL283C	WSC2	3	fold_recogniti	1ei3A	315
YNL284C	MRPL10	1	psiblast	1lnrJ_	117
YNL284C	MRPL10	2	psiblast	1lnrJ_	117
YNL284C	MRPL10	3	unassigned	na	88
YNL285W	YNL285W	1	unassigned	na	123
YNL286W	CUS2	1	psiblast	1cvjA_	130
YNL286W	CUS2	2	psiblast	1cvjA_	155
YNL287W	SEC21	1	psiblast	1gw5B_	105
YNL287W	SEC21	2	psiblast	1gw5B_	108
YNL287W	SEC21	3	psiblast	1gw5B_	216
YNL287W	SEC21	4	psiblast	1gw5B_	94
YNL287W	SEC21	5	psiblast	1gw5B_	127
YNL287W	SEC21	6	msa	na	99
YNL287W	SEC21	7	msa	na	186

YNL288W	CAF40	1	unassigned	na	76
YNL288W	CAF40	2	fold_recogniti	1ee5A	203
YNL288W	CAF40	3	fold_recogniti	1ee5A	94
YNL289W	PCL1	1	psiblast	1vin__	150
YNL289W	PCL1	2	psiblast	1vin__	129
YNL290W	RFC3	1	psiblast	1lfsA_	89
YNL290W	RFC3	2	psiblast	1lfsA_	251
YNL291C	MID1	1	msa	na	245
YNL291C	MID1	2	unassigned	na	303
YNL292W	PUS4	1	psiblast	1k8wA_	282
YNL292W	PUS4	2	psiblast	1k8wA_	121
YNL293W	MSB3	1	unassigned	na	131
YNL293W	MSB3	2	psiblast	1fkmA_	244
YNL293W	MSB3	3	psiblast	1fkmA_	83
YNL293W	MSB3	4	unassigned	na	67
YNL293W	MSB3	5	msa	na	108
YNL294C	RIM21	1	unassigned	na	64
YNL294C	RIM21	2	msa	na	312
YNL294C	RIM21	3	unassigned	na	157
YNL295W	YNL295W	1	unassigned	na	524
YNL296W	YNL296W	1	unassigned	na	104
YNL297C	MON2	1	msa	na	220
YNL297C	MON2	2	unassigned	na	106
YNL297C	MON2	3	msa	na	1067
YNL297C	MON2	4	unassigned	na	243
YNL298W	CLA4	1	fold_recogniti	1faoA	177
YNL298W	CLA4	2	psiblast	1f3mA_	81
YNL298W	CLA4	3	fold_recogniti	1deqA	203
YNL298W	CLA4	4	psiblast	1f3mC_	181
YNL298W	CLA4	5	psiblast	1f3mC_	200
YNL299W	TRF5	1	unassigned	na	122
YNL299W	TRF5	2	psiblast	1f5aA_	269
YNL299W	TRF5	3	psiblast	1f5aA_	111
YNL299W	TRF5	4	unassigned	na	123
YNL300W	YNL300W	1	unassigned	na	102
YNL301C	RPL18B	1	psiblast	1k5yO_	186
YNL302C	RPS19B	1	pfam	PF01090	144
YNL303W	YNL303W	1	unassigned	na	115
YNL304W	YPT11	1	unassigned	na	97
YNL304W	YPT11	2	psiblast	1d5cA_	258
YNL305C	YNL305C	1	pfam	PF01027	297
YNL306W	MRPS18	1	unassigned	na	93
YNL306W	MRPS18	2	psiblast	1l1uK_	124
YNL307C	MCK1	1	psiblast	1i09A_	117
YNL307C	MCK1	2	psiblast	1i09A_	258
YNL308C	KRI1	1	unassigned	na	121
YNL308C	KRI1	2	msa	na	470
YNL309W	STB1	1	fold_recogniti	1l8mA	95

YNL309W	STB1	2	fold_recogniti	1k83A	364
YNL310C	FMP28	1	unassigned	na	55
YNL310C	FMP28	2	msa	na	150
YNL311C	YNL311C	1	pfam	PF00646	128
YNL311C	YNL311C	2	unassigned	na	635
YNL312W	RFA2	1	psiblast	1quqA_	178
YNL312W	RFA2	2	psiblast	1dpuA_	95
YNL313C	YNL313C	1	unassigned	na	105
YNL313C	YNL313C	2	msa	na	380
YNL313C	YNL313C	3	psiblast	1b89A_	78
YNL313C	YNL313C	4	psiblast	1fchA_	122
YNL313C	YNL313C	5	psiblast	1fchA_	75
YNL313C	YNL313C	6	psiblast	1fchA_	72
YNL313C	YNL313C	7	psiblast	1fchA_	72
YNL314W	DAL82	1	unassigned	na	255
YNL315C	ATP11	1	unassigned	na	87
YNL315C	ATP11	2	msa	na	231
YNL316C	PHA2	1	psiblast	1ecmA_	45
YNL316C	PHA2	2	pfam	PF00800	215
YNL316C	PHA2	3	psiblast	1in9A_	108
YNL317W	PFS2	1	psiblast	1gg2B_	333
YNL317W	PFS2	2	fold_recogniti	1scgB	132
YNL318C	HXT14	1	psiblast	1ja5A_	481
YNL318C	HXT14	2	psiblast	1ja5A_	59
YNL319W	YNL319W	1	unassigned	na	146
YNL320W	YNL320W	1	psiblast	1qfsA_	284
YNL321W	YNL321W	1	unassigned	na	178
YNL321W	YNL321W	2	pfam	PF03733	148
YNL321W	YNL321W	3	unassigned	na	138
YNL321W	YNL321W	4	msa	na	444
YNL322C	KRE1	1	fold_recogniti	1ei3A	313
YNL323W	LEM3	1	unassigned	na	78
YNL323W	LEM3	2	pfam	PF03381	336
YNL324W	YNL324W	1	unassigned	na	131
YNL325C	FIG4	1	unassigned	na	95
YNL325C	FIG4	2	pfam	PF02383	335
YNL325C	FIG4	3	msa	na	163
YNL325C	FIG4	4	unassigned	na	286
YNL326C	YNL326C	1	unassigned	na	82
YNL326C	YNL326C	2	pfam	PF01529	96
YNL326C	YNL326C	3	msa	na	158
YNL327W	EGT2	1	msa	na	412
YNL327W	EGT2	2	msa	na	629
YNL328C	MDJ2	1	unassigned	na	82
YNL328C	MDJ2	2	psiblast	1hdj__	64
YNL329C	PEX6	1	unassigned	na	190
YNL329C	PEX6	2	psiblast	1e32A_	115
YNL329C	PEX6	3	psiblast	1e32A_	132

YNL329C	PEX6	4	psiblast	1e32A_	181
YNL329C	PEX6	5	psiblast	1e32A_	89
YNL329C	PEX6	6	psiblast	1iqpA_	58
YNL329C	PEX6	7	psiblast	1d2nA_	118
YNL329C	PEX6	8	fold_recogniti	1lv7A	147
YNL330C	RPD3	1	psiblast	1c3rA_	177
YNL330C	RPD3	2	psiblast	1c3rA_	170
YNL330C	RPD3	3	unassigned	na	86
YNL331C	AAD14	1	psiblast	1exbA_	285
YNL331C	AAD14	2	psiblast	1exbA_	91
YNL332W	THI12	1	psiblast	1gggA_	188
YNL332W	THI12	2	psiblast	1gggA_	98
YNL332W	THI12	3	unassigned	na	54
YNL333W	SNZ2	1	fold_recogniti	1gt8A	190
YNL333W	SNZ2	2	psiblast	2tpsA_	108
YNL334C	SNO2	1	psiblast	1gpmA_	222
YNL336W	COS1	1	msa	na	61
YNL336W	COS1	2	pfam	PF00674	110
YNL336W	COS1	3	unassigned	na	70
YNL336W	COS1	4	pfam	PF00674	140
YNL337W	YNL337W	1	unassigned	na	84
YNL338W	YNL338W	1	unassigned	na	52
YNL339C	YRF1-6	1	msa	na	206
YNL339C	YRF1-6	2	fold_recogniti	1mukA	594
YNL339C	YRF1-6	3	psiblast	1fuuA_	456
YNL339C	YRF1-6	4	unassigned	na	55
YNL339C	YRF1-6	5	psiblast	1dnnB_	187
YNL339C	YRF1-6	6	unassigned	na	361
YNR001C	CIT1	1	psiblast	1cts__	375
YNR001C	CIT1	2	psiblast	1cts__	104
YNR001W-A	YNR001W-A	1	unassigned	na	72
YNR002C	FUN34	1	pfam	PF01184	282
YNR003C	RPC34	1	msa	na	193
YNR003C	RPC34	2	unassigned	na	124
YNR004W	YNR004W	1	unassigned	na	146
YNR005C	YNR005C	1	unassigned	na	134
YNR006W	VPS27	1	fold_recogniti	1hf8A	146
YNR006W	VPS27	2	psiblast	1vfyA_	97
YNR006W	VPS27	3	unassigned	na	113
YNR006W	VPS27	4	fold_recogniti	1ldvA	266
YNR007C	ATG3	1	unassigned	na	141
YNR007C	ATG3	2	msa	na	169
YNR008W	LRO1	1	unassigned	na	125
YNR008W	LRO1	2	msa	na	96
YNR008W	LRO1	3	fold_recogniti	1ex9A	171
YNR008W	LRO1	4	msa	na	210
YNR008W	LRO1	5	unassigned	na	59
YNR009W	YNR009W	1	unassigned	na	249

YNR010W	CSE2	1	unassigned	na	149
YNR011C	PRP2	1	msa	na	165
YNR011C	PRP2	2	psiblast	1fuuA_	435
YNR011C	PRP2	3	msa	na	97
YNR011C	PRP2	4	msa	na	179
YNR012W	URK1	1	psiblast	1esmA_	279
YNR012W	URK1	2	psiblast	1bd3D_	222
YNR013C	PHO91	1	pfam	PF03105	312
YNR013C	PHO91	2	unassigned	na	90
YNR013C	PHO91	3	pfam	PF00939	492
YNR014W	YNR014W	1	unassigned	na	212
YNR015W	SMM1	1	psiblast	1gt8A_	305
YNR015W	SMM1	2	psiblast	1gt8A_	79
YNR016C	ACC1	1	psiblast	1bncA_	174
YNR016C	ACC1	2	psiblast	1bncA_	174
YNR016C	ACC1	3	psiblast	1bncA_	255
YNR016C	ACC1	4	unassigned	na	92
YNR016C	ACC1	5	psiblast	1dczA_	86
YNR016C	ACC1	6	unassigned	na	208
YNR016C	ACC1	7	msa	na	102
YNR016C	ACC1	8	unassigned	na	63
YNR016C	ACC1	9	msa	na	174
YNR016C	ACC1	10	msa	na	199
YNR016C	ACC1	11	msa	na	619
YNR016C	ACC1	12	unassigned	na	87
YNR017W	MAS6	1	msa	na	222
YNR018W	YNR018W	1	msa	na	224
YNR019W	ARE2	1	unassigned	na	133
YNR019W	ARE2	2	msa	na	185
YNR019W	ARE2	3	unassigned	na	79
YNR019W	ARE2	4	pfam	PF01800	245
YNR020C	YNR020C	1	unassigned	na	66
YNR020C	YNR020C	2	msa	na	204
YNR021W	YNR021W	1	msa	na	322
YNR021W	YNR021W	2	msa	na	82
YNR022C	MRPL50	1	unassigned	na	139
YNR023W	SNF12	1	msa	na	204
YNR023W	SNF12	2	unassigned	na	59
YNR023W	SNF12	3	msa	na	303
YNR024W	YNR024W	1	unassigned	na	186
YNR025C	YNR025C	1	unassigned	na	119
YNR026C	SEC12	1	psiblast	1gg2B_	337
YNR026C	SEC12	2	psiblast	1gxrA_	134
YNR027W	BUD17	1	psiblast	1lljA_	317
YNR028W	CPR8	1	psiblast	1cynA_	253
YNR028W	CPR8	2	unassigned	na	55
YNR029C	YNR029C	1	unassigned	na	65
YNR029C	YNR029C	2	psiblast	2ffhA_	250

YNR029C	YNR029C	3	msa	na	114
YNR030W	ECM39	1	msa	na	407
YNR030W	ECM39	2	unassigned	na	144
YNR031C	SSK2	1	unassigned	na	560
YNR031C	SSK2	2	msa	na	326
YNR031C	SSK2	3	unassigned	na	237
YNR031C	SSK2	4	psiblast	1gxcA_	133
YNR031C	SSK2	5	psiblast	1f3mC_	90
YNR031C	SSK2	6	psiblast	1f3mC_	233
YNR032C-A	HUB1	1	psiblast	1ud7A_	73
YNR032W	PPG1	1	psiblast	1fjmA_	368
YNR033W	ABZ1	1	psiblast	1qdlB_	249
YNR033W	ABZ1	2	psiblast	1k0eA_	538
YNR034W	SOL1	1	psiblast	1d9tA_	321
YNR034W-A	YNR034W-A	1	unassigned	na	98
YNR035C	ARC35	1	psiblast	1k8kD_	128
YNR035C	ARC35	2	psiblast	1k8kD_	214
YNR036C	YNR036C	1	psiblast	1l1uL_	153
YNR037C	RSM19	1	psiblast	1fjgS_	91
YNR038W	DBP6	1	msa	na	169
YNR038W	DBP6	2	psiblast	1hv8A_	241
YNR038W	DBP6	3	psiblast	1hv8A_	219
YNR039C	ZRG17	1	unassigned	na	137
YNR039C	ZRG17	2	msa	na	219
YNR039C	ZRG17	3	unassigned	na	249
YNR040W	YNR040W	1	unassigned	na	256
YNR041C	COQ2	1	unassigned	na	94
YNR041C	COQ2	2	pfam	PF01040	278
YNR042W	YNR042W	1	unassigned	na	142
YNR043W	MVD1	1	psiblast	1fi4A_	396
YNR044W	AGA1	1	msa	na	169
YNR044W	AGA1	2	fold_recogniti	1m2vB	74
YNR044W	AGA1	3	unassigned	na	76
YNR044W	AGA1	4	msa	na	134
YNR044W	AGA1	5	fold_recogniti	1deqA	149
YNR044W	AGA1	6	msa	na	123
YNR045W	PET494	1	unassigned	na	489
YNR046W	YNR046W	1	pfam	PF03966	135
YNR047W	YNR047W	1	fold_recogniti	1k83A	307
YNR047W	YNR047W	2	unassigned	na	119
YNR047W	YNR047W	3	psiblast	1fotA_	155
YNR047W	YNR047W	4	psiblast	1fotA_	233
YNR047W	YNR047W	5	unassigned	na	79
YNR048W	YNR048W	1	pfam	PF03381	393
YNR049C	MSO1	1	unassigned	na	210
YNR050C	LYS9	1	psiblast	1e5lA_	149
YNR050C	LYS9	2	psiblast	1e5lA_	206
YNR050C	LYS9	3	psiblast	1e5lA_	91

YNR051C	BRE5	1	psiblast	1gy7A_	149
YNR051C	BRE5	2	fold_recogniti	1m2vB	366
YNR052C	POP2	1	unassigned	na	154
YNR052C	POP2	2	fold_recogniti	1fxxA	279
YNR053C	NOG2	1	msa	na	112
YNR053C	NOG2	2	psiblast	1jah__	200
YNR053C	NOG2	3	psiblast	1egaA_	174
YNR054C	YNR054C	1	psiblast	1up1__	98
YNR054C	YNR054C	2	psiblast	1up1__	99
YNR054C	YNR054C	3	psiblast	1qm9A_	68
YNR054C	YNR054C	4	psiblast	1g9lA_	51
YNR055C	HOL1	1	msa	na	63
YNR055C	HOL1	2	psiblast	1lviA_	442
YNR055C	HOL1	3	unassigned	na	81
YNR056C	BIO5	1	msa	na	154
YNR056C	BIO5	2	unassigned	na	125
YNR056C	BIO5	3	msa	na	282
YNR057C	BIO4	1	psiblast	1m3tA_	237
YNR058W	BIO3	1	psiblast	1qj3A_	480
YNR059W	MNT4	1	unassigned	na	181
YNR059W	MNT4	2	fold_recogniti	1ga8A	180
YNR059W	MNT4	3	unassigned	na	219
YNR060W	FRE4	1	msa	na	212
YNR060W	FRE4	2	msa	na	124
YNR060W	FRE4	3	unassigned	na	60
YNR060W	FRE4	4	psiblast	1cnf__	125
YNR060W	FRE4	5	psiblast	1cnf__	125
YNR060W	FRE4	6	unassigned	na	73
YNR061C	YNR061C	1	msa	na	145
YNR061C	YNR061C	2	unassigned	na	74
YNR062C	YNR062C	1	unassigned	na	327
YNR063W	YNR063W	1	psiblast	1qp9A_	75
YNR063W	YNR063W	2	msa	na	434
YNR063W	YNR063W	3	unassigned	na	98
YNR064C	YNR064C	1	psiblast	1bn6A_	290
YNR065C	YSN1	1	unassigned	na	229
YNR065C	YSN1	2	msa	na	737
YNR065C	YSN1	3	unassigned	na	150
YNR066C	YNR066C	1	fold_recogniti	1mz5A	436
YNR067C	DSE4	1	msa	na	407
YNR067C	DSE4	2	pfam	PF03639	710
YNR068C	YNR068C	1	msa	na	120
YNR068C	YNR068C	2	unassigned	na	152
YNR069C	BSC5	1	unassigned	na	489
YNR070W	YNR070W	1	psiblast	1jj7A_	308
YNR070W	YNR070W	2	unassigned	na	59
YNR070W	YNR070W	3	psiblast	1jsqA_	302
YNR070W	YNR070W	4	psiblast	1jsqA_	308

YNR070W	YNR070W	5	msa	na	296
YNR070W	YNR070W	6	unassigned	na	60
YNR071C	YNR071C	1	psiblast	1l7jA_	342
YNR072W	HXT17	1	psiblast	1ja5A_	462
YNR072W	HXT17	2	psiblast	1ja5A_	102
YNR074C	YNR074C	1	psiblast	1ndaA_	233
YNR074C	YNR074C	2	psiblast	1ndaA_	145
YNR075W	COS10	1	msa	na	54
YNR075W	COS10	2	pfam	PF00674	108
YNR075W	COS10	3	unassigned	na	75
YNR075W	COS10	4	pfam	PF00674	137
YNR077C	YNR077C	1	unassigned	na	84
YOL001W	PHO80	1	psiblast	1vin__	209
YOL001W	PHO80	2	msa	na	84
YOL002C	YOL002C	1	unassigned	na	72
YOL002C	YOL002C	2	pfam	PF03006	255
YOL003C	YOL003C	1	pfam	PF01529	154
YOL003C	YOL003C	2	unassigned	na	224
YOL004W	SIN3	1	fold_recogniti	1k83A	237
YOL004W	SIN3	2	pfam	PF02671	84
YOL004W	SIN3	3	unassigned	na	102
YOL004W	SIN3	4	pfam	PF02671	66
YOL004W	SIN3	5	psiblast	1g1eB_	117
YOL004W	SIN3	6	fold_recogniti	1g1eB	126
YOL004W	SIN3	7	msa	na	292
YOL004W	SIN3	8	msa	na	344
YOL004W	SIN3	9	msa	na	168
YOL005C	RPB11	1	psiblast	1k83K_	120
YOL006C	TOP1	1	msa	na	123
YOL006C	TOP1	2	psiblast	1ois__	133
YOL006C	TOP1	3	psiblast	1ois__	110
YOL006C	TOP1	4	psiblast	1a41__	114
YOL006C	TOP1	5	psiblast	2tmaA_	54
YOL006C	TOP1	6	psiblast	2tmaA_	81
YOL006C	TOP1	7	psiblast	2tmaA_	104
YOL006C	TOP1	8	fold_recogniti	1a36A	50
YOL007C	YOL007C	1	unassigned	na	341
YOL008W	YOL008W	1	pfam	PF03654	207
YOL009C	MDM12	1	msa	na	181
YOL009C	MDM12	2	unassigned	na	90
YOL010W	RCL1	1	psiblast	1qmhA_	81
YOL010W	RCL1	2	psiblast	1qmhA_	96
YOL010W	RCL1	3	psiblast	1qmhA_	190
YOL011W	PLB3	1	psiblast	1cjyA_	368
YOL011W	PLB3	2	fold_recogniti	1cjyA	245
YOL011W	PLB3	3	unassigned	na	73
YOL012C	HTZ1	1	psiblast	1f66C_	66
YOL012C	HTZ1	2	psiblast	1f66C_	68

YOL013C	HRD1	1	msa	na	221
YOL013C	HRD1	2	unassigned	na	123
YOL013C	HRD1	3	psiblast	1chc__	88
YOL013C	HRD1	4	unassigned	na	119
YOL013W	5345	1	unassigned	na	63
YOL013W-A	YOL013W-A	1	unassigned	na	96
YOL014W	YOL014W	1	unassigned	na	124
YOL015W	YOL015W	1	msa	na	109
YOL015W	YOL015W	2	unassigned	na	477
YOL016C	CMK2	1	psiblast	1a06__	145
YOL016C	CMK2	2	psiblast	1a06__	200
YOL016C	CMK2	3	psiblast	1exrA_	102
YOL017W	ESC8	1	msa	na	599
YOL017W	ESC8	2	unassigned	na	115
YOL018C	TLG2	1	psiblast	1ez3A_	131
YOL018C	TLG2	2	psiblast	1ez3A_	72
YOL018C	TLG2	3	psiblast	1jthB_	142
YOL018C	TLG2	4	unassigned	na	52
YOL019W	YOL019W	1	msa	na	230
YOL019W	YOL019W	2	fold_recogniti	1m2vB	321
YOL020W	TAT2	1	unassigned	na	53
YOL020W	TAT2	2	pfam	PF00324	539
YOL021C	DIS3	1	msa	na	325
YOL021C	DIS3	2	msa	na	90
YOL021C	DIS3	3	pfam	PF00773	408
YOL021C	DIS3	4	psiblast	1go3E_	178
YOL022C	YOL022C	1	msa	na	282
YOL022C	YOL022C	2	msa	na	126
YOL023W	IFM1	1	msa	na	119
YOL023W	IFM1	2	psiblast	1g7sA_	203
YOL023W	IFM1	3	psiblast	1g7sA_	128
YOL023W	IFM1	4	psiblast	1g7sA_	117
YOL023W	IFM1	5	psiblast	1g7sA_	109
YOL024W	YOL024W	1	unassigned	na	172
YOL025W	LAG2	1	unassigned	na	83
YOL025W	LAG2	2	fold_recogniti	1e6vA	348
YOL025W	LAG2	3	fold_recogniti	1e6vA	119
YOL025W	LAG2	4	fold_recogniti	1l9uD	110
YOL026C	MIM1	1	unassigned	na	113
YOL027C	MDM38	1	msa	na	229
YOL027C	MDM38	2	psiblast	1i84S_	157
YOL027C	MDM38	3	psiblast	1i84S_	187
YOL028C	YAP7	1	msa	na	112
YOL028C	YAP7	2	psiblast	1hjbA_	133
YOL029C	YOL029C	1	unassigned	na	201
YOL030W	GAS5	1	psiblast	1bglA_	196
YOL030W	GAS5	2	psiblast	1bglA_	132
YOL030W	GAS5	3	fold_recogniti	1ldvA	156

YOL031C	SIL1	1	unassigned	na	125
YOL031C	SIL1	2	fold_recogniti	1ejlI	106
YOL031C	SIL1	3	fold_recogniti	1ejlI	93
YOL031C	SIL1	4	fold_recogniti	1ejlI	97
YOL032W	YOL032W	1	msa	na	246
YOL033W	MSE1	1	psiblast	1gln__	282
YOL033W	MSE1	2	psiblast	1gln__	86
YOL033W	MSE1	3	psiblast	1gln__	61
YOL033W	MSE1	4	psiblast	1gln__	107
YOL034W	SMC5	1	psiblast	1ii8A_	126
YOL034W	SMC5	2	psiblast	1i84S_	157
YOL034W	SMC5	3	psiblast	1cii__	50
YOL034W	SMC5	4	psiblast	1cii__	102
YOL034W	SMC5	5	psiblast	1cii__	278
YOL034W	SMC5	6	psiblast	1c1gA_	190
YOL034W	SMC5	7	psiblast	1e69A_	190
YOL035C	YOL035C	1	unassigned	na	100
YOL036W	YOL036W	1	unassigned	na	761
YOL037C	YOL037C	1	unassigned	na	117
YOL038C-A	YOL038C-A	1	unassigned	na	31
YOL038W	PRE6	1	psiblast	1g0uC_	254
YOL039W	RPP2A	1	pfam	PF00428	106
YOL040C	RPS15	1	psiblast	1k5xS_	142
YOL041C	NOP12	1	psiblast	1fj7A_	90
YOL041C	NOP12	2	unassigned	na	58
YOL041C	NOP12	3	psiblast	1l3kA_	122
YOL041C	NOP12	4	psiblast	1l3kA_	102
YOL041C	NOP12	5	msa	na	87
YOL042W	NGL1	1	fold_recogniti	1hd7A	363
YOL043C	NTG2	1	unassigned	na	97
YOL043C	NTG2	2	psiblast	2abk__	174
YOL043C	NTG2	3	psiblast	2abk__	109
YOL044W	PEX15	1	unassigned	na	383
YOL045W	PSK2	1	msa	na	208
YOL045W	PSK2	2	unassigned	na	79
YOL045W	PSK2	3	fold_recogniti	1ll8A	146
YOL045W	PSK2	4	unassigned	na	194
YOL045W	PSK2	5	psiblast	1dsyA_	186
YOL045W	PSK2	6	psiblast	1fotA_	103
YOL045W	PSK2	7	psiblast	1fotA_	185
YOL046C	YOL046C	1	unassigned	na	224
YOL047C	YOL047C	1	msa	na	234
YOL048C	YOL048C	1	unassigned	na	106
YOL049W	GSH2	1	psiblast	2hgsA_	432
YOL049W	GSH2	2	psiblast	2hgsA_	59
YOL050C	YOL050C	1	unassigned	na	106
YOL051W	GAL11	1	psiblast	1i84S_	296
YOL051W	GAL11	2	fold_recogniti	1m2vB	211

YOL051W	GAL11	3	unassigned	na	94
YOL051W	GAL11	4	msa	na	80
YOL051W	GAL11	5	fold_recogniti	1k83A	235
YOL051W	GAL11	6	unassigned	na	165
YOL052C	SPE2	1	psiblast	1jl0A_	199
YOL052C	SPE2	2	psiblast	1jl0A_	197
YOL052C-A	DDR2	1	unassigned	na	61
YOL053W	YOL053W	1	msa	na	133
YOL053W	YOL053W	2	unassigned	na	73
YOL053W	YOL053W	3	msa	na	189
YOL054W	PSH1	1	psiblast	1fbvA_	107
YOL054W	PSH1	2	psiblast	1fre__	74
YOL054W	PSH1	3	msa	na	150
YOL054W	PSH1	4	psiblast	1flkA_	75
YOL055C	THI20	1	psiblast	1lljA_	316
YOL055C	THI20	2	fold_recogniti	1dveA	235
YOL056W	GPM3	1	psiblast	1bq3D_	303
YOL057W	YOL057W	1	msa	na	130
YOL057W	YOL057W	2	pfam	PF03571	581
YOL058W	ARG1	1	psiblast	1kh1A_	146
YOL058W	ARG1	2	psiblast	1kh1A_	118
YOL058W	ARG1	3	psiblast	1kh1A_	156
YOL059W	GPD2	1	unassigned	na	71
YOL059W	GPD2	2	psiblast	1evyA_	232
YOL059W	GPD2	3	psiblast	1evyA_	137
YOL060C	MAM3	1	pfam	PF01595	195
YOL060C	MAM3	2	psiblast	1zfjA_	326
YOL060C	MAM3	3	unassigned	na	185
YOL061W	PRS5	1	psiblast	1dkrA_	162
YOL061W	PRS5	2	psiblast	1dkrA_	154
YOL061W	PRS5	3	psiblast	1ao0A_	180
YOL062C	APM4	1	psiblast	1gw5M_	179
YOL062C	APM4	2	psiblast	1gw5M_	312
YOL063C	MOR1	1	msa	na	363
YOL063C	MOR1	2	unassigned	na	594
YOL064C	MET22	1	psiblast	1ka0A_	220
YOL064C	MET22	2	psiblast	1ka0A_	137
YOL065C	INP54	1	psiblast	1i9yA_	384
YOL066C	RIB2	1	psiblast	1fjgD_	100
YOL066C	RIB2	2	psiblast	1kskA_	66
YOL066C	RIB2	3	psiblast	1kskA_	202
YOL066C	RIB2	4	unassigned	na	58
YOL066C	RIB2	5	fold_recogniti	1jtkA	165
YOL067C	RTG1	1	fold_recogniti	1am9A	177
YOL068C	HST1	1	unassigned	na	145
YOL068C	HST1	2	psiblast	1j8fA_	237
YOL068C	HST1	3	psiblast	1j8fA_	121
YOL069W	NUF2	1	psiblast	1i84S_	323

YOL069W	NUF2	2	psiblast	2tmaA_	128
YOL070C	YOL070C	1	unassigned	na	501
YOL071W	EMI5	1	pfam	PF03937	162
YOL072W	THP1	1	fold_recogniti	1hz4A	107
YOL072W	THP1	2	fold_recogniti	1hz4A	117
YOL072W	THP1	3	fold_recogniti	1hz4A	156
YOL072W	THP1	4	msa	na	75
YOL073C	YOL073C	1	msa	na	212
YOL073C	YOL073C	2	fold_recogniti	1n6cA_	110
YOL075C	YOL075C	1	psiblast	1l2tA_	281
YOL075C	YOL075C	2	unassigned	na	71
YOL075C	YOL075C	3	psiblast	1jsqA_	302
YOL075C	YOL075C	4	psiblast	1jsqA_	288
YOL075C	YOL075C	5	msa	na	296
YOL075C	YOL075C	6	unassigned	na	56
YOL076W	MDM20	1	msa	na	587
YOL076W	MDM20	2	unassigned	na	209
YOL077C	BRX1	1	msa	na	230
YOL077C	BRX1	2	unassigned	na	61
YOL077W-A	ATP19	1	unassigned	na	68
YOL078W	AVO1	1	unassigned	na	615
YOL078W	AVO1	2	msa	na	176
YOL078W	AVO1	3	unassigned	na	224
YOL078W	AVO1	4	msa	na	161
YOL079W	YOL079W	1	unassigned	na	132
YOL080C	REX4	1	unassigned	na	108
YOL080C	REX4	2	psiblast	1fxxA_	181
YOL081W	IRA2	1	unassigned	na	1260
YOL081W	IRA2	2	msa	na	299
YOL081W	IRA2	3	unassigned	na	106
YOL081W	IRA2	4	psiblast	1nf1A_	121
YOL081W	IRA2	5	psiblast	1nf1A_	215
YOL081W	IRA2	6	unassigned	na	529
YOL081W	IRA2	7	msa	na	347
YOL081W	IRA2	8	unassigned	na	202
YOL082W	ATG19	1	unassigned	na	415
YOL083W	YOL083W	1	unassigned	na	412
YOL084W	PHM7	1	msa	na	193
YOL084W	PHM7	2	unassigned	na	99
YOL084W	PHM7	3	pfam	PF02714	488
YOL084W	PHM7	4	unassigned	na	211
YOL085C	YOL085C	1	unassigned	na	113
YOL086C	ADH1	1	psiblast	1hdyA_	140
YOL086C	ADH1	2	psiblast	1hdyA_	70
YOL086C	ADH1	3	psiblast	1hdyA_	138
YOL086W-A	YOL086W-A	1	unassigned	na	90
YOL087C	YOL087C	1	psiblast	1gg2B_	338
YOL087C	YOL087C	2	psiblast	1erjA_	131

YOL087C	YOL087C	3	fold_recogniti	1k83A	368
YOL087C	YOL087C	4	fold_recogniti	1m2vB	111
YOL087C	YOL087C	5	msa	na	168
YOL088C	MPD2	1	psiblast	1quwA_	144
YOL088C	MPD2	2	psiblast	1bjx__	133
YOL089C	HAL9	1	msa	na	111
YOL089C	HAL9	2	psiblast	2alcA_	82
YOL089C	HAL9	3	unassigned	na	91
YOL089C	HAL9	4	msa	na	297
YOL089C	HAL9	5	msa	na	225
YOL089C	HAL9	6	unassigned	na	224
YOL090W	MSH2	1	psiblast	1e3mA_	118
YOL090W	MSH2	2	psiblast	1e3mA_	174
YOL090W	MSH2	3	psiblast	1e3mA_	297
YOL090W	MSH2	4	psiblast	1e3mA_	215
YOL090W	MSH2	5	psiblast	1e3mA_	79
YOL090W	MSH2	6	unassigned	na	81
YOL091W	SPO21	1	psiblast	1i84S_	392
YOL091W	SPO21	2	msa	na	217
YOL092W	YOL092W	1	msa	na	308
YOL093W	TRM10	1	unassigned	na	85
YOL093W	TRM10	2	msa	na	208
YOL094C	RFC4	1	psiblast	1iqpA_	78
YOL094C	RFC4	2	psiblast	1iqpA_	245
YOL095C	HMI1	1	psiblast	1pjr__	274
YOL095C	HMI1	2	psiblast	1pjr__	432
YOL096C	COQ3	1	psiblast	1fp1D_	68
YOL096C	COQ3	2	psiblast	1kp9A_	248
YOL097C	WRS1	1	psiblast	1jiiA_	287
YOL097C	WRS1	2	psiblast	1jiiA_	145
YOL098C	YOL098C	1	psiblast	1bccA_	273
YOL098C	YOL098C	2	psiblast	1bccA_	225
YOL098C	YOL098C	3	unassigned	na	52
YOL098C	YOL098C	4	fold_recogniti	1kb9A	251
YOL098C	YOL098C	5	fold_recogniti	1kb9A	236
YOL099C	YOL099C	1	unassigned	na	163
YOL100W	PKH2	1	psiblast	1a25A_	132
YOL100W	PKH2	2	psiblast	1fotA_	149
YOL100W	PKH2	3	psiblast	1fotA_	218
YOL100W	PKH2	4	fold_recogniti	1k83A	316
YOL100W	PKH2	5	unassigned	na	266
YOL101C	YOL101C	1	pfam	PF03006	312
YOL102C	TPT1	1	pfam	PF01885	230
YOL103W	ITR2	1	unassigned	na	93
YOL103W	ITR2	2	psiblast	1ja5A_	421
YOL103W	ITR2	3	psiblast	1ja5A_	98
YOL104C	NDJ1	1	unassigned	na	352
YOL105C	WSC3	1	pfam	PF01822	112

YOL105C	WSC3	2	fold_recogniti	1deqA	61
YOL105C	WSC3	3	fold_recogniti	1ei3A	302
YOL105C	WSC3	4	unassigned	na	81
YOL106W	YOL106W	1	unassigned	na	117
YOL107W	YOL107W	1	msa	na	242
YOL107W	YOL107W	2	msa	na	100
YOL108C	INO4	1	fold_recogniti	1hloA	151
YOL109W	ZEO1	1	psiblast	1av1A_	113
YOL110W	SHR5	1	unassigned	na	237
YOL111C	YOL111C	1	unassigned	na	72
YOL111C	YOL111C	2	psiblast	1aarA_	140
YOL112W	MSB4	1	unassigned	na	91
YOL112W	MSB4	2	psiblast	1fkmA_	264
YOL112W	MSB4	3	psiblast	1fkmA_	76
YOL112W	MSB4	4	msa	na	61
YOL113W	SKM1	1	fold_recogniti	1fhwA	117
YOL113W	SKM1	2	psiblast	1f3mA_	89
YOL113W	SKM1	3	unassigned	na	99
YOL113W	SKM1	4	psiblast	1f3mC_	149
YOL113W	SKM1	5	psiblast	1f3mC_	201
YOL114C	YOL114C	1	psiblast	1gqeA_	202
YOL115W	TRF4	1	unassigned	na	116
YOL115W	TRF4	2	psiblast	1f5aA_	357
YOL115W	TRF4	3	psiblast	1f5aA_	111
YOL116W	MSN1	1	unassigned	na	87
YOL116W	MSN1	2	msa	na	151
YOL116W	MSN1	3	unassigned	na	144
YOL117W	RRI2	1	unassigned	na	275
YOL117W	RRI2	2	fold_recogniti	1qoyA	285
YOL117W	RRI2	3	unassigned	na	85
YOL118C	YOL118C	1	unassigned	na	102
YOL119C	MCH4	1	msa	na	87
YOL119C	MCH4	2	psiblast	1lviA_	414
YOL121C	RPS19A	1	pfam	PF01090	144
YOL122C	SMF1	1	unassigned	na	94
YOL122C	SMF1	2	pfam	PF01566	412
YOL122C	SMF1	3	unassigned	na	69
YOL123W	HRP1	1	unassigned	na	58
YOL123W	HRP1	2	psiblast	1cvjA_	94
YOL123W	HRP1	3	psiblast	2up1A_	92
YOL123W	HRP1	4	psiblast	1qm9A_	103
YOL123W	HRP1	5	psiblast	1qm9A_	82
YOL123W	HRP1	6	unassigned	na	105
YOL124C	YOL124C	1	unassigned	na	112
YOL124C	YOL124C	2	psiblast	1g60A_	79
YOL124C	YOL124C	3	psiblast	2admA_	242
YOL125W	YOL125W	1	msa	na	228
YOL125W	YOL125W	2	unassigned	na	248

YOL126C	MDH2	1	psiblast	1mldA_	423
YOL127W	RPL25	1	pfam	PF03939	54
YOL127W	RPL25	2	psiblast	1k5yT_	88
YOL128C	YGK3	1	psiblast	1i09A_	126
YOL128C	YGK3	2	psiblast	1i09A_	249
YOL129W	VPS68	1	unassigned	na	184
YOL130W	ALR1	1	unassigned	na	365
YOL130W	ALR1	2	msa	na	189
YOL130W	ALR1	3	fold_recogniti	1hciA	123
YOL130W	ALR1	4	fold_recogniti	1hciA	182
YOL131W	YOL131W	1	unassigned	na	108
YOL132W	GAS4	1	pfam	PF03198	343
YOL132W	GAS4	2	unassigned	na	128
YOL133W	HRT1	1	psiblast	1ldjB_	121
YOL134C	YOL134C	1	unassigned	na	129
YOL135C	MED7	1	msa	na	222
YOL136C	PFK27	1	psiblast	2bifA_	327
YOL136C	PFK27	2	unassigned	na	70
YOL137W	BSC6	1	unassigned	na	59
YOL137W	BSC6	2	psiblast	1lviA_	438
YOL138C	YOL138C	1	psiblast	1c5kA_	110
YOL138C	YOL138C	2	psiblast	1erjA_	328
YOL138C	YOL138C	3	psiblast	1gg2B_	114
YOL138C	YOL138C	4	msa	na	163
YOL138C	YOL138C	5	unassigned	na	414
YOL138C	YOL138C	6	msa	na	212
YOL139C	CDC33	1	psiblast	1ap8__	213
YOL140W	ARG8	1	psiblast	1lx9A_	423
YOL141W	PPM2	1	msa	na	363
YOL141W	PPM2	2	psiblast	1gof__	332
YOL142W	RRP40	1	fold_recogniti	1e3hA	146
YOL142W	RRP40	2	msa	na	94
YOL143C	RIB4	1	psiblast	1ejbA_	169
YOL144W	NOP8	1	psiblast	1b7fA_	155
YOL144W	NOP8	2	unassigned	na	329
YOL145C	CTR9	1	psiblast	1elrA_	128
YOL145C	CTR9	2	psiblast	1fchA_	138
YOL145C	CTR9	3	psiblast	1fchA_	86
YOL145C	CTR9	4	psiblast	1fchA_	139
YOL145C	CTR9	5	unassigned	na	52
YOL145C	CTR9	6	psiblast	1jcqA_	86
YOL145C	CTR9	7	psiblast	1jcqA_	59
YOL145C	CTR9	8	psiblast	1i84S_	122
YOL145C	CTR9	9	psiblast	1i84S_	267
YOL146W	PSF3	1	fold_recogniti	1ek8A	122
YOL146W	PSF3	2	fold_recogniti	1ek8A	114
YOL147C	PEX11	1	unassigned	na	115
YOL147C	PEX11	2	msa	na	121

YOL148C	SPT20	1	unassigned	na	604
YOL149W	DCP1	1	unassigned	na	231
YOL150C	YOL150C	1	unassigned	na	103
YOL151W	GRE2	1	psiblast	1keuA_	159
YOL151W	GRE2	2	psiblast	1keuA_	116
YOL151W	GRE2	3	psiblast	1keuA_	67
YOL152W	FRE7	1	unassigned	na	78
YOL152W	FRE7	2	msa	na	236
YOL152W	FRE7	3	psiblast	1cnf__	99
YOL152W	FRE7	4	psiblast	1cnf__	140
YOL152W	FRE7	5	unassigned	na	76
YOL153C	YOL153C	1	unassigned	na	130
YOL154W	ZPS1	1	fold_recogniti	1ge5A	249
YOL155C	YOL155C	1	msa	na	428
YOL155C	YOL155C	2	unassigned	na	63
YOL155C	YOL155C	3	msa	na	476
YOL156W	HXT11	1	psiblast	1ja5A_	470
YOL156W	HXT11	2	psiblast	1ja5A_	97
YOL157C	YOL157C	1	psiblast	1uok__	302
YOL157C	YOL157C	2	psiblast	1uok__	287
YOL158C	ENB1	1	psiblast	1lviA_	256
YOL158C	ENB1	2	unassigned	na	69
YOL158C	ENB1	3	msa	na	208
YOL158C	ENB1	4	unassigned	na	73
YOL159C	YOL159C	1	unassigned	na	171
YOL159C-A	YOL159C-A	1	unassigned	na	90
YOL160W	YOL160W	1	unassigned	na	113
YOL161C	YOL161C	1	pfam	PF00660	120
YOL162W	YOL162W	1	msa	na	145
YOL162W	YOL162W	2	unassigned	na	70
YOL163W	YOL163W	1	psiblast	1ja5A_	169
YOL164W	YOL164W	1	unassigned	na	80
YOL164W	YOL164W	2	psiblast	1bc2A_	174
YOL164W	YOL164W	3	psiblast	1bc2A_	118
YOL164W	YOL164W	4	unassigned	na	55
YOL164W	YOL164W	5	fold_recogniti	1hxiA	89
YOL164W	YOL164W	6	psiblast	1iktA_	130
YOL165C	AAD15	1	psiblast	1exbA_	143
YOL166C	YOL166C	1	unassigned	na	112
YOR001W	RRP6	1	psiblast	1j5fA_	187
YOR001W	RRP6	2	psiblast	1m0yA_	233
YOR001W	RRP6	3	msa	na	107
YOR001W	RRP6	4	msa	na	206
YOR002W	ALG6	1	pfam	PF03155	544
YOR003W	YSP3	1	unassigned	na	68
YOR003W	YSP3	2	psiblast	1kn6A_	104
YOR003W	YSP3	3	psiblast	1ic6A_	306
YOR004W	YOR004W	1	msa	na	198

YOR004W	YOR004W	2	unassigned	na	56
YOR005C	DNL4	1	msa	na	242
YOR005C	DNL4	2	fold_recogniti	1fviA	67
YOR005C	DNL4	3	psiblast	1fviA_	112
YOR005C	DNL4	4	psiblast	1fviA_	47
YOR005C	DNL4	5	psiblast	1fviA_	111
YOR005C	DNL4	6	unassigned	na	154
YOR005C	DNL4	7	psiblast	1ik9C_	83
YOR005C	DNL4	8	unassigned	na	128
YOR006C	YOR006C	1	msa	na	238
YOR006C	YOR006C	2	unassigned	na	75
YOR007C	SGT2	1	unassigned	na	61
YOR007C	SGT2	2	psiblast	1a17__	230
YOR007C	SGT2	3	unassigned	na	55
YOR008C	SLG1	1	fold_recogniti	1ei3A	64
YOR008C	SLG1	2	fold_recogniti	1m2vB	314
YOR008C-A	YOR008C-A	1	unassigned	na	74
YOR008W-B	YOR008W-B	1	unassigned	na	33
YOR009W	TIR4	1	pfam	PF00660	123
YOR009W	TIR4	2	fold_recogniti	1ei3A	300
YOR009W	TIR4	3	fold_recogniti	1deqA	64
YOR010C	TIR2	1	pfam	PF00660	119
YOR010C	TIR2	2	msa	na	132
YOR011W	AUS1	1	psiblast	1g291_	360
YOR011W	AUS1	2	msa	na	224
YOR011W	AUS1	3	unassigned	na	153
YOR011W	AUS1	4	pfam	PF00005	247
YOR011W	AUS1	5	msa	na	305
YOR011W	AUS1	6	unassigned	na	105
YOR012W	YOR012W	1	unassigned	na	137
YOR013W	YOR013W	1	unassigned	na	156
YOR014W	RTS1	1	fold_recogniti	1ei3A	282
YOR014W	RTS1	2	msa	na	92
YOR014W	RTS1	3	fold_recogniti	1b3uA	95
YOR014W	RTS1	4	fold_recogniti	1b3uA	288
YOR015W	YOR015W	1	unassigned	na	119
YOR016C	ERP4	1	pfam	PF01105	207
YOR017W	PET127	1	unassigned	na	147
YOR017W	PET127	2	msa	na	495
YOR017W	PET127	3	unassigned	na	158
YOR018W	ROD1	1	msa	na	122
YOR018W	ROD1	2	psiblast	1jsyA_	139
YOR018W	ROD1	3	psiblast	1jsyA_	155
YOR018W	ROD1	4	fold_recogniti	1k28D	301
YOR018W	ROD1	5	fold_recogniti	1k28D	120
YOR019W	YOR019W	1	msa	na	134
YOR019W	YOR019W	2	unassigned	na	83
YOR019W	YOR019W	3	fold_recogniti	1jmvA	214

YOR019W	YOR019W	4	unassigned	na	299
YOR020C	HSP10	1	psiblast	1hx5A_	106
YOR021C	YOR021C	1	unassigned	na	78
YOR021C	YOR021C	2	msa	na	135
YOR022C	YOR022C	1	unassigned	na	261
YOR022C	YOR022C	2	msa	na	166
YOR022C	YOR022C	3	unassigned	na	64
YOR022C	YOR022C	4	pfam	PF02862	224
YOR023C	AHC1	1	unassigned	na	261
YOR023C	AHC1	2	fold_recogniti	1ldvA	305
YOR024W	YOR024W	1	unassigned	na	107
YOR025W	HST3	1	psiblast	1j8fA_	275
YOR025W	HST3	2	psiblast	1j8fA_	117
YOR025W	HST3	3	unassigned	na	55
YOR026W	BUB3	1	psiblast	1b9xA_	341
YOR027W	STI1	1	psiblast	1elwA_	147
YOR027W	STI1	2	unassigned	na	77
YOR027W	STI1	3	psiblast	1elrA_	117
YOR027W	STI1	4	psiblast	1elrA_	50
YOR027W	STI1	5	psiblast	1a17__	198
YOR028C	CIN5	1	msa	na	229
YOR028C	CIN5	2	psiblast	1gd2E_	66
YOR029W	YOR029W	1	unassigned	na	111
YOR030W	DFG16	1	unassigned	na	157
YOR030W	DFG16	2	msa	na	321
YOR030W	DFG16	3	unassigned	na	141
YOR031W	CRS5	1	unassigned	na	8
YOR032C	HMS1	1	msa	na	263
YOR032C	HMS1	2	psiblast	1am9A_	171
YOR033C	EXO1	1	psiblast	1a76__	361
YOR033C	EXO1	2	psiblast	1d9dA_	66
YOR033C	EXO1	3	unassigned	na	275
YOR034C	AKR2	1	unassigned	na	80
YOR034C	AKR2	2	psiblast	1k1aA_	104
YOR034C	AKR2	3	psiblast	1k1aA_	153
YOR034C	AKR2	4	msa	na	300
YOR034C	AKR2	5	msa	na	112
YOR035C	SHE4	1	unassigned	na	659
YOR035C	SHE4	2	fold_recogniti	1b3uA	130
YOR036W	PEP12	1	psiblast	1dn1B_	229
YOR036W	PEP12	2	psiblast	1dn1B_	59
YOR037W	CYC2	1	unassigned	na	90
YOR037W	CYC2	2	psiblast	1ndh__	134
YOR037W	CYC2	3	psiblast	1ndh__	180
YOR038C	HIR2	1	msa	na	85
YOR038C	HIR2	2	psiblast	1gg2B_	388
YOR038C	HIR2	3	msa	na	225
YOR038C	HIR2	4	unassigned	na	54

YOR038C	HIR2	5	fold_recogniti	1bgxT	123
YOR039W	CKB2	1	psiblast	1jwhC_	97
YOR039W	CKB2	2	psiblast	1jwhC_	161
YOR040W	GLO4	1	psiblast	1qh3A_	130
YOR040W	GLO4	2	psiblast	1qh3A_	70
YOR040W	GLO4	3	psiblast	1qh3A_	85
YOR041C	SRF5	1	unassigned	na	143
YOR042W	CUE5	1	unassigned	na	88
YOR042W	CUE5	2	pfam	PF02845	71
YOR042W	CUE5	3	unassigned	na	252
YOR043W	WHI2	1	msa	na	309
YOR043W	WHI2	2	unassigned	na	177
YOR044W	YOR044W	1	unassigned	na	157
YOR045W	TOM6	1	unassigned	na	61
YOR046C	DBP5	1	psiblast	1fuuA_	482
YOR047C	STD1	1	unassigned	na	444
YOR048C	RAT1	1	pfam	PF03159	271
YOR048C	RAT1	2	unassigned	na	407
YOR048C	RAT1	3	msa	na	111
YOR048C	RAT1	4	unassigned	na	91
YOR048C	RAT1	5	msa	na	126
YOR049C	RSB1	1	unassigned	na	183
YOR049C	RSB1	2	msa	na	171
YOR050C	YOR050C	1	unassigned	na	115
YOR051C	YOR051C	1	psiblast	1i84S_	251
YOR051C	YOR051C	2	fold_recogniti	1llwA	161
YOR052C	YOR052C	1	msa	na	88
YOR052C	YOR052C	2	pfam	PF01428	62
YOR053W	YOR053W	1	unassigned	na	113
YOR054C	VHS3	1	unassigned	na	281
YOR054C	VHS3	2	fold_recogniti	1mvlA	94
YOR054C	VHS3	3	psiblast	1e20A_	217
YOR054C	VHS3	4	unassigned	na	82
YOR055W	YOR055W	1	unassigned	na	144
YOR056C	NOB1	1	msa	na	177
YOR056C	NOB1	2	msa	na	282
YOR057W	SGT1	1	psiblast	1a17__	54
YOR057W	SGT1	2	psiblast	1d8dA_	178
YOR057W	SGT1	3	psiblast	1lg0A_	84
YOR057W	SGT1	4	unassigned	na	79
YOR058C	ASE1	1	msa	na	298
YOR058C	ASE1	2	psiblast	1i84S_	448
YOR058C	ASE1	3	unassigned	na	139
YOR059C	YOR059C	1	psiblast	1eh5A_	256
YOR059C	YOR059C	2	unassigned	na	194
YOR060C	YOR060C	1	unassigned	na	257
YOR061W	CKA2	1	psiblast	1ds5A_	167
YOR061W	CKA2	2	psiblast	1ds5A_	111

YOR061W	CKA2	3	psiblast	1ds5A_	61
YOR062C	YOR062C	1	msa	na	127
YOR062C	YOR062C	2	unassigned	na	141
YOR063W	RPL3	1	psiblast	1k5yC_	64
YOR063W	RPL3	2	psiblast	1k5yC_	191
YOR063W	RPL3	3	psiblast	1k5yC_	132
YOR064C	YNG1	1	msa	na	121
YOR064C	YNG1	2	psiblast	1f62A_	98
YOR065W	CYT1	1	psiblast	1kyoD_	309
YOR066W	YOR066W	1	fold_recogniti	1ivoA	547
YOR066W	YOR066W	2	fold_recogniti	1m6bA	82
YOR067C	ALG8	1	pfam	PF03155	577
YOR068C	VAM10	1	unassigned	na	114
YOR069W	VPS5	1	psiblast	1bg2__	198
YOR069W	VPS5	2	psiblast	1i84S_	234
YOR069W	VPS5	3	psiblast	1i84S_	243
YOR070C	GYP1	1	msa	na	238
YOR070C	GYP1	2	psiblast	1fkmA_	248
YOR070C	GYP1	3	psiblast	1fkmA_	151
YOR071C	YOR071C	1	pfam	PF02133	544
YOR071C	YOR071C	2	unassigned	na	54
YOR072W	YOR072W	1	unassigned	na	104
YOR073W	SGO1	1	msa	na	410
YOR073W	SGO1	2	unassigned	na	180
YOR074C	CDC21	1	psiblast	1ci7A_	220
YOR074C	CDC21	2	psiblast	1ci7A_	84
YOR075W	UFE1	1	psiblast	1dn1B_	285
YOR075W	UFE1	2	psiblast	1dn1B_	61
YOR076C	SKI7	1	unassigned	na	70
YOR076C	SKI7	2	msa	na	190
YOR076C	SKI7	3	psiblast	1f60A_	213
YOR076C	SKI7	4	psiblast	1f60A_	165
YOR076C	SKI7	5	psiblast	1f60A_	109
YOR077W	RTS2	1	msa	na	232
YOR078W	BUD21	1	unassigned	na	214
YOR079C	ATX2	1	msa	na	128
YOR079C	ATX2	2	pfam	PF02535	185
YOR080W	DIA2	1	fold_recogniti	1qqeA	215
YOR080W	DIA2	2	pfam	PF00646	51
YOR080W	DIA2	3	unassigned	na	344
YOR080W	DIA2	4	msa	na	136
YOR081C	STC2	1	fold_recogniti	1itwA	314
YOR081C	STC2	2	msa	na	103
YOR081C	STC2	3	msa	na	332
YOR082C	YOR082C	1	unassigned	na	113
YOR083W	WHI5	1	unassigned	na	295
YOR084W	YOR084W	1	psiblast	1cqzA_	387
YOR085W	OST3	1	msa	na	95

YOR085W	OST3	2	msa	na	255
YOR086C	TCB1	1	unassigned	na	80
YOR086C	TCB1	2	msa	na	290
YOR086C	TCB1	3	psiblast	1gmiA_	136
YOR086C	TCB1	4	psiblast	1dqvA_	134
YOR086C	TCB1	5	psiblast	1dqvA_	151
YOR086C	TCB1	6	unassigned	na	139
YOR086C	TCB1	7	psiblast	1dsyA_	200
YOR086C	TCB1	8	unassigned	na	56
YOR087W	YVC1	1	unassigned	na	247
YOR088W	5593	1	unassigned	na	72
YOR088W	5593	2	pfam	PF00520	193
YOR088W	5593	3	msa	na	115
YOR088W	5593	4	msa	na	102
YOR089C	VPS21	1	psiblast	1ek0A_	210
YOR090C	PTC5	1	unassigned	na	106
YOR090C	PTC5	2	psiblast	1a6q__	466
YOR091W	YOR091W	1	msa	na	401
YOR092W	ECM3	1	msa	na	174
YOR092W	ECM3	2	unassigned	na	196
YOR092W	ECM3	3	msa	na	243
YOR093C	YOR093C	1	msa	na	61
YOR093C	YOR093C	2	unassigned	na	78
YOR093C	YOR093C	3	psiblast	1amuA_	292
YOR093C	YOR093C	4	psiblast	1amuA_	261
YOR093C	YOR093C	5	psiblast	1amuA_	140
YOR093C	YOR093C	6	psiblast	1dnyA_	78
YOR093C	YOR093C	7	unassigned	na	63
YOR093C	YOR093C	8	psiblast	1ba3__	189
YOR093C	YOR093C	9	psiblast	1ba3__	348
YOR093C	YOR093C	10	psiblast	1ba3__	138
YOR094W	ARF3	1	psiblast	1e0sA_	183
YOR095C	RKI1	1	psiblast	1lk5A_	169
YOR095C	RKI1	2	psiblast	1lk5A_	89
YOR096W	RPS7A	1	pfam	PF01251	190
YOR097C	YOR097C	1	unassigned	na	175
YOR098C	NUP1	1	msa	na	276
YOR098C	NUP1	2	msa	na	800
YOR099W	KTR1	1	pfam	PF01793	393
YOR100C	CRC1	1	fold_recogniti	1is2A	327
YOR101W	RAS1	1	psiblast	821p__	185
YOR101W	RAS1	2	unassigned	na	59
YOR101W	RAS1	3	msa	na	65
YOR102W	YOR102W	1	unassigned	na	116
YOR103C	OST2	1	pfam	PF02109	133
YOR104W	PIN2	1	unassigned	na	282
YOR105W	YOR105W	1	unassigned	na	108
YOR106W	VAM3	1	psiblast	1hs7A_	121

YOR106W	VAM3	2	unassigned	na	64
YOR106W	VAM3	3	fold_recogniti	1gl2B	98
YOR107W	RGS2	1	psiblast	1cmzA_	195
YOR107W	RGS2	2	unassigned	na	114
YOR108W	LEU9	1	unassigned	na	52
YOR108W	LEU9	2	fold_recogniti	1euaA	166
YOR108W	LEU9	3	fold_recogniti	1f5zA	272
YOR108W	LEU9	4	msa	na	114
YOR109W	INP53	1	pfam	PF02383	403
YOR109W	INP53	2	unassigned	na	92
YOR109W	INP53	3	psiblast	1i9yA_	394
YOR109W	INP53	4	fold_recogniti	1ldvA	218
YOR110W	TFC7	1	psiblast	1bq3D_	233
YOR110W	TFC7	2	msa	na	202
YOR111W	YOR111W	1	psiblast	1ex2A_	170
YOR111W	YOR111W	2	psiblast	1ex2A_	62
YOR112W	YOR112W	1	psiblast	1bmkA_	130
YOR112W	YOR112W	2	psiblast	1bmkA_	194
YOR112W	YOR112W	3	psiblast	1b3uA_	329
YOR112W	YOR112W	4	unassigned	na	108
YOR113W	AZF1	1	unassigned	na	114
YOR113W	AZF1	2	msa	na	148
YOR113W	AZF1	3	msa	na	219
YOR113W	AZF1	4	unassigned	na	52
YOR113W	AZF1	5	psiblast	1lu6A_	147
YOR113W	AZF1	6	msa	na	182
YOR113W	AZF1	7	unassigned	na	52
YOR114W	YOR114W	1	unassigned	na	294
YOR115C	TRS33	1	msa	na	268
YOR116C	RPO31	1	psiblast	1k83A_	1460
YOR117W	RPT5	1	msa	na	158
YOR117W	RPT5	2	psiblast	1e32A_	193
YOR117W	RPT5	3	psiblast	1e32A_	83
YOR118W	YOR118W	1	unassigned	na	254
YOR118W	YOR118W	2	msa	na	313
YOR119C	RIO1	1	psiblast	2ptk__	103
YOR119C	RIO1	2	psiblast	1a06__	97
YOR119C	RIO1	3	psiblast	1a06__	217
YOR119C	RIO1	4	unassigned	na	67
YOR120W	GCY1	1	psiblast	2alr__	312
YOR121C	YOR121C	1	unassigned	na	101
YOR122C	PFY1	1	psiblast	1yprA_	126
YOR123C	LEO1	1	msa	na	181
YOR123C	LEO1	2	unassigned	na	283
YOR124C	UBP2	1	unassigned	na	616
YOR124C	UBP2	2	msa	na	106
YOR124C	UBP2	3	pfam	PF00442	64
YOR124C	UBP2	4	unassigned	na	52

YOR124C	UBP2	5	msa	na	346
YOR124C	UBP2	6	pfam	PF00443	88
YOR125C	CAT5	1	unassigned	na	72
YOR125C	CAT5	2	fold_recogniti	1jgcA	200
YOR126C	IAH1	1	psiblast	1es9A_	238
YOR127W	RGA1	1	psiblast	1b8tA_	70
YOR127W	RGA1	2	psiblast	1b8tA_	60
YOR127W	RGA1	3	fold_recogniti	1ldvA	108
YOR127W	RGA1	4	fold_recogniti	1k83A	378
YOR127W	RGA1	5	unassigned	na	123
YOR127W	RGA1	6	psiblast	1am4A_	139
YOR127W	RGA1	7	psiblast	1am4A_	129
YOR128C	ADE2	1	psiblast	1b6rA_	122
YOR128C	ADE2	2	psiblast	1b6rA_	63
YOR128C	ADE2	3	psiblast	1b6rA_	208
YOR128C	ADE2	4	psiblast	1qczA_	178
YOR129C	YOR129C	1	msa	na	105
YOR129C	YOR129C	2	unassigned	na	550
YOR129C	YOR129C	3	fold_recogniti	1l9uD	238
YOR130C	ORT1	1	fold_recogniti	1is2A	292
YOR131C	YOR131C	1	psiblast	1fezA_	218
YOR132W	VPS17	1	unassigned	na	71
YOR132W	VPS17	2	psiblast	1h6hA_	166
YOR132W	VPS17	3	psiblast	1i84S_	246
YOR132W	VPS17	4	unassigned	na	68
YOR134W	BAG7	1	psiblast	1grnB_	145
YOR134W	BAG7	2	psiblast	1grnB_	141
YOR134W	BAG7	3	unassigned	na	123
YOR135C	YOR135C	1	unassigned	na	113
YOR136W	IDH2	1	psiblast	1xac__	369
YOR137C	SIA1	1	unassigned	na	281
YOR137C	SIA1	2	psiblast	1qhwA_	341
YOR138C	RUP1	1	pfam	PF00627	67
YOR138C	RUP1	2	unassigned	na	604
YOR139C	YOR139C	1	unassigned	na	130
YOR140W	SFL1	1	psiblast	1hks__	176
YOR140W	SFL1	2	msa	na	118
YOR140W	SFL1	3	unassigned	na	76
YOR140W	SFL1	4	fold_recogniti	1ldvA	168
YOR140W	SFL1	5	unassigned	na	69
YOR140W	SFL1	6	fold_recogniti	1deqA	159
YOR141C	ARP8	1	msa	na	108
YOR141C	ARP8	2	unassigned	na	201
YOR141C	ARP8	3	psiblast	2btfA_	191
YOR141C	ARP8	4	psiblast	2btfA_	205
YOR141C	ARP8	5	fold_recogniti	1k8kA	176
YOR142W	LSC1	1	psiblast	1eucA_	160
YOR142W	LSC1	2	psiblast	1eucA_	169

YOR143C	THI80	1	psiblast	1ig0A_	107
YOR143C	THI80	2	psiblast	1ig0A_	212
YOR144C	ELG1	1	psiblast	1jbkA_	162
YOR144C	ELG1	2	unassigned	na	76
YOR144C	ELG1	3	psiblast	1iqpA_	130
YOR144C	ELG1	4	psiblast	1iqpA_	324
YOR144C	ELG1	5	unassigned	na	99
YOR145C	PNO1	1	msa	na	86
YOR145C	PNO1	2	psiblast	1j4wA_	95
YOR145C	PNO1	3	psiblast	1j4wA_	93
YOR146W	YOR146W	1	unassigned	na	101
YOR147W	MDM32	1	unassigned	na	105
YOR147W	MDM32	2	msa	na	437
YOR147W	MDM32	3	unassigned	na	111
YOR148C	SPP2	1	unassigned	na	185
YOR149C	SMP3	1	pfam	PF03901	516
YOR150W	MRPL23	1	psiblast	1lnrH_	163
YOR151C	RPB2	1	psiblast	1k83B_	527
YOR151C	RPB2	2	psiblast	1k83B_	601
YOR151C	RPB2	3	psiblast	1k83B_	96
YOR152C	YOR152C	1	unassigned	na	256
YOR153W	PDR5	1	unassigned	na	97
YOR153W	PDR5	2	psiblast	1l2tA_	331
YOR153W	PDR5	3	unassigned	na	65
YOR153W	PDR5	4	psiblast	1jsqA_	336
YOR153W	PDR5	5	psiblast	1jsqA_	290
YOR153W	PDR5	6	msa	na	301
YOR153W	PDR5	7	unassigned	na	91
YOR154W	YOR154W	1	unassigned	na	110
YOR154W	YOR154W	2	msa	na	407
YOR154W	YOR154W	3	unassigned	na	70
YOR155C	ISN1	1	unassigned	na	450
YOR156C	NFI1	1	pfam	PF02037	96
YOR156C	NFI1	2	unassigned	na	229
YOR156C	NFI1	3	pfam	PF02891	62
YOR156C	NFI1	4	fold_recogniti	1k83A	339
YOR157C	PUP1	1	psiblast	1fntI_	261
YOR158W	PET123	1	unassigned	na	66
YOR158W	PET123	2	msa	na	158
YOR158W	PET123	3	unassigned	na	94
YOR159C	SME1	1	psiblast	1jriA_	94
YOR160W	MTR10	1	msa	na	642
YOR160W	MTR10	2	msa	na	330
YOR161C	PNS1	1	unassigned	na	257
YOR161C	PNS1	2	msa	na	111
YOR161C	PNS1	3	msa	na	171
YOR162C	YRR1	1	psiblast	1hwtD_	141
YOR162C	YRR1	2	unassigned	na	108

YOR162C	YRR1	3	msa	na	332
YOR162C	YRR1	4	msa	na	229
YOR163W	DDP1	1	psiblast	1f3yA_	188
YOR164C	YOR164C	1	fold_recogniti	1qqeA	131
YOR164C	YOR164C	2	fold_recogniti	1qqeA	181
YOR165W	SEY1	1	psiblast	1dg3A_	148
YOR165W	SEY1	2	fold_recogniti	1n1bA	135
YOR165W	SEY1	3	fold_recogniti	1n1bA	350
YOR165W	SEY1	4	unassigned	na	143
YOR166C	YOR166C	1	unassigned	na	112
YOR166C	YOR166C	2	msa	na	205
YOR166C	YOR166C	3	unassigned	na	141
YOR167C	RPS28A	1	pfam	PF01200	67
YOR168W	GLN4	1	unassigned	na	198
YOR168W	GLN4	2	psiblast	1qrtA_	290
YOR168W	GLN4	3	psiblast	1qrtA_	94
YOR168W	GLN4	4	psiblast	1qrtA_	227
YOR169C	YOR169C	1	unassigned	na	154
YOR170W	YOR170W	1	unassigned	na	101
YOR171C	LCB4	1	unassigned	na	160
YOR171C	LCB4	2	fold_recogniti	1kq3A	207
YOR171C	LCB4	3	unassigned	na	61
YOR171C	LCB4	4	msa	na	196
YOR172W	YRM1	1	psiblast	1hwtD_	100
YOR172W	YRM1	2	msa	na	456
YOR172W	YRM1	3	msa	na	230
YOR173W	DCS2	1	unassigned	na	397
YOR174W	MED4	1	unassigned	na	64
YOR174W	MED4	2	msa	na	220
YOR175C	YOR175C	1	msa	na	143
YOR175C	YOR175C	2	pfam	PF03062	369
YOR175C	YOR175C	3	unassigned	na	107
YOR176W	HEM15	1	psiblast	1hrkA_	201
YOR176W	HEM15	2	psiblast	1hrkA_	192
YOR177C	MPC54	1	psiblast	1i84S_	407
YOR177C	MPC54	2	unassigned	na	57
YOR178C	GAC1	1	unassigned	na	171
YOR178C	GAC1	2	pfam	PF03370	221
YOR178C	GAC1	3	unassigned	na	401
YOR179C	SYC1	1	unassigned	na	188
YOR180C	DCI1	1	psiblast	1hnoA_	271
YOR181W	LAS17	1	pfam	PF00568	154
YOR181W	LAS17	2	msa	na	272
YOR181W	LAS17	3	fold_recogniti	1deqA	207
YOR183W	FYV12	1	unassigned	na	129
YOR184W	SER1	1	psiblast	1bjoA_	112
YOR184W	SER1	2	psiblast	1bjoA_	283
YOR185C	GSP2	1	psiblast	1a2kC_	220

YOR186W	YOR186W	1	unassigned	na	144
YOR187W	TUF1	1	psiblast	1b23P_	239
YOR187W	TUF1	2	psiblast	1b23P_	198
YOR188W	MSB1	1	unassigned	na	1137
YOR189W	IES4	1	unassigned	na	116
YOR190W	SPR1	1	psiblast	1cz1A_	329
YOR190W	SPR1	2	psiblast	1cz1A_	116
YOR191W	RIS1	1	unassigned	na	91
YOR191W	RIS1	2	psiblast	1i84S_	347
YOR191W	RIS1	3	msa	na	194
YOR191W	RIS1	4	unassigned	na	125
YOR191W	RIS1	5	msa	na	670
YOR191W	RIS1	6	msa	na	192
YOR192C	YOR192C	1	pfam	PF02133	547
YOR192C	YOR192C	2	unassigned	na	52
YOR193W	PEX27	1	unassigned	na	75
YOR193W	PEX27	2	msa	na	301
YOR194C	TOA1	1	psiblast	1ytfB_	88
YOR194C	TOA1	2	unassigned	na	121
YOR194C	TOA1	3	psiblast	1ytfC_	77
YOR195W	SLK19	1	psiblast	1i84S_	458
YOR195W	SLK19	2	psiblast	2tmaA_	94
YOR195W	SLK19	3	psiblast	2tmaA_	103
YOR195W	SLK19	4	msa	na	166
YOR196C	LIP5	1	msa	na	177
YOR196C	LIP5	2	fold_recogniti	1o0yA	237
YOR197W	MCA1	1	fold_recogniti	1m2vB	100
YOR197W	MCA1	2	fold_recogniti	1cp3A	353
YOR198C	BFR1	1	psiblast	1i84S_	323
YOR198C	BFR1	2	msa	na	147
YOR199W	YOR199W	1	unassigned	na	109
YOR200W	YOR200W	1	unassigned	na	132
YOR201C	PET56	1	msa	na	85
YOR201C	PET56	2	psiblast	1ipaA_	145
YOR201C	PET56	3	psiblast	1ipaA_	182
YOR202W	HIS3	1	pfam	PF00475	220
YOR203W	YOR203W	1	unassigned	na	117
YOR204W	DED1	1	fold_recogniti	1deqA	142
YOR204W	DED1	2	psiblast	1hv8A_	227
YOR204W	DED1	3	psiblast	1hv8A_	175
YOR204W	DED1	4	unassigned	na	60
YOR205C	FMP38	1	psiblast	1egaA_	224
YOR205C	FMP38	2	msa	na	332
YOR206W	NOC2	1	msa	na	251
YOR206W	NOC2	2	unassigned	na	175
YOR206W	NOC2	3	msa	na	284
YOR207C	RET1	1	psiblast	1k83B_	470
YOR207C	RET1	2	psiblast	1k83B_	590

YOR207C	RET1	3	psiblast	1k83B_	89
YOR208W	PTP2	1	unassigned	na	200
YOR208W	PTP2	2	psiblast	1larA_	550
YOR209C	NPT1	1	msa	na	183
YOR209C	NPT1	2	msa	na	246
YOR210W	RPB10	1	psiblast	1k83J_	70
YOR211C	MGM1	1	msa	na	184
YOR211C	MGM1	2	psiblast	1jwyB_	326
YOR211C	MGM1	3	fold_recogniti	1b89A	392
YOR212W	STE4	1	psiblast	1scgB_	423
YOR213C	SAS5	1	pfam	PF03366	179
YOR213C	SAS5	2	unassigned	na	69
YOR214C	YOR214C	1	unassigned	na	236
YOR215C	YOR215C	1	fold_recogniti	1egyA	185
YOR216C	RUD3	1	psiblast	1i84S_	217
YOR216C	RUD3	2	psiblast	2tmaA_	52
YOR216C	RUD3	3	psiblast	2tmaA_	82
YOR216C	RUD3	4	psiblast	2tmaA_	62
YOR216C	RUD3	5	psiblast	2tmaA_	71
YOR217W	RFC1	1	psiblast	1e32A_	196
YOR217W	RFC1	2	psiblast	1e32A_	84
YOR217W	RFC1	3	psiblast	1iqpA_	85
YOR217W	RFC1	4	psiblast	1iqpA_	298
YOR217W	RFC1	5	unassigned	na	198
YOR218C	YOR218C	1	unassigned	na	139
YOR219C	STE13	1	msa	na	114
YOR219C	STE13	2	msa	na	101
YOR219C	STE13	3	psiblast	1c5kA_	92
YOR219C	STE13	4	psiblast	1qfsA_	320
YOR219C	STE13	5	psiblast	1qfsA_	304
YOR220W	YOR220W	1	unassigned	na	265
YOR221C	MCT1	1	psiblast	1mla__	384
YOR222W	ODC2	1	fold_recogniti	1is2A	307
YOR223W	YOR223W	1	unassigned	na	292
YOR224C	RPB8	1	psiblast	1a1d__	146
YOR225W	YOR225W	1	unassigned	na	109
YOR226C	ISU2	1	pfam	PF01592	156
YOR227W	YOR227W	1	fold_recogniti	1deqA	223
YOR227W	YOR227W	2	unassigned	na	69
YOR227W	YOR227W	3	msa	na	221
YOR227W	YOR227W	4	psiblast	2bnh__	84
YOR227W	YOR227W	5	psiblast	2bnh__	42
YOR227W	YOR227W	6	psiblast	2bnh__	230
YOR227W	YOR227W	7	psiblast	1jl5A_	97
YOR227W	YOR227W	8	fold_recogniti	1l2qA	280
YOR228C	YOR228C	1	unassigned	na	302
YOR229W	WTM2	1	unassigned	na	112
YOR229W	WTM2	2	psiblast	1gg2B_	355

YOR230W	WTM1	1	unassigned	na	74
YOR230W	WTM1	2	psiblast	1gg2B_	363
YOR231W	MKK1	1	psiblast	1f3mA_	83
YOR231W	MKK1	2	unassigned	na	69
YOR231W	MKK1	3	psiblast	1f3mC_	149
YOR231W	MKK1	4	psiblast	1f3mC_	207
YOR232W	MGE1	1	psiblast	1dkgA_	170
YOR232W	MGE1	2	psiblast	1dkgA_	58
YOR233W	KIN4	1	psiblast	1fotA_	191
YOR233W	KIN4	2	psiblast	1fotA_	225
YOR233W	KIN4	3	unassigned	na	384
YOR234C	RPL33B	1	pfam	PF01247	107
YOR235W	YOR235W	1	unassigned	na	104
YOR236W	DFR1	1	psiblast	1ai9A_	211
YOR237W	HES1	1	pfam	PF01237	434
YOR238W	YOR238W	1	unassigned	na	306
YOR239W	ABP140	1	fold_recogniti	1l8mA	72
YOR239W	ABP140	2	fold_recogniti	1l8mA	79
YOR239W	ABP140	3	fold_recogniti	1l8mA	70
YOR239W	ABP140	4	fold_recogniti	1l8mA	58
YOR239W-A	5745	1	psiblast	2tmaA_	60
YOR239W-A	5745	2	psiblast	1i84S_	357
YOR239W-A	5745	3	psiblast	1im8A_	128
YOR239W-A	5745	4	psiblast	1hnnA_	83
YOR240W	5746	1	psiblast	1amuA_	71
YOR240W	5746	2	psiblast	1kp9A_	291
YOR241W	MET7	1	unassigned	na	97
YOR241W	MET7	2	psiblast	1fgs__	282
YOR241W	MET7	3	psiblast	1fgs__	169
YOR242C	SSP2	1	unassigned	na	154
YOR242C	SSP2	2	psiblast	1cvjA_	98
YOR242C	SSP2	3	psiblast	1cvjA_	119
YOR243C	PUS7	1	unassigned	na	117
YOR243C	PUS7	2	pfam	PF01142	490
YOR243C	PUS7	3	unassigned	na	69
YOR244W	ESA1	1	fold_recogniti	1ap0_	141
YOR244W	ESA1	2	psiblast	1fy7A_	77
YOR244W	ESA1	3	psiblast	1fy7A_	96
YOR244W	ESA1	4	psiblast	1fy7A_	61
YOR244W	ESA1	5	psiblast	1fy7A_	70
YOR245C	DGA1	1	unassigned	na	144
YOR245C	DGA1	2	msa	na	274
YOR246C	YOR246C	1	psiblast	1cydA_	330
YOR247W	SRL1	1	unassigned	na	210
YOR248W	YOR248W	1	unassigned	na	100
YOR249C	APC5	1	msa	na	223
YOR249C	APC5	2	unassigned	na	59
YOR249C	APC5	3	fold_recogniti	1hz4A	103

YOR249C	APC5	4	fold_recogniti	1hz4A	127
YOR249C	APC5	5	fold_recogniti	1hz4A	173
YOR250C	CLP1	1	psiblast	1jpjA_	106
YOR250C	CLP1	2	psiblast	1jpjA_	256
YOR250C	CLP1	3	msa	na	83
YOR251C	YOR251C	1	psiblast	1rhd__	158
YOR251C	YOR251C	2	psiblast	1rhd__	146
YOR252W	YOR252W	1	unassigned	na	141
YOR253W	NAT5	1	psiblast	1bo4A_	176
YOR254C	SEC63	1	unassigned	na	116
YOR254C	SEC63	2	psiblast	1xbl__	125
YOR254C	SEC63	3	msa	na	422
YOR255W	OSW1	1	unassigned	na	278
YOR256C	YOR256C	1	unassigned	na	151
YOR256C	YOR256C	2	psiblast	1de4C_	318
YOR256C	YOR256C	3	psiblast	1de4C_	340
YOR257W	CDC31	1	psiblast	1iq5A_	89
YOR257W	CDC31	2	psiblast	1iq5A_	72
YOR258W	HNT3	1	fold_recogniti	1av5A	161
YOR258W	HNT3	2	unassigned	na	56
YOR259C	RPT4	1	psiblast	1e32A_	94
YOR259C	RPT4	2	psiblast	1e32A_	83
YOR259C	RPT4	3	psiblast	1e32A_	174
YOR259C	RPT4	4	psiblast	1e32A_	86
YOR260W	GCD1	1	psiblast	1hv9A_	345
YOR260W	GCD1	2	psiblast	1hv9A_	233
YOR261C	RPN8	1	pfam	PF01398	125
YOR261C	RPN8	2	msa	na	213
YOR262W	YOR262W	1	psiblast	1fts__	200
YOR262W	YOR262W	2	unassigned	na	147
YOR263C	YOR263C	1	unassigned	na	135
YOR264W	DSE3	1	unassigned	na	430
YOR265W	RBL2	1	psiblast	1qsdA_	106
YOR266W	PNT1	1	unassigned	na	423
YOR267C	HRK1	1	unassigned	na	181
YOR267C	HRK1	2	psiblast	1aq1__	117
YOR267C	HRK1	3	psiblast	1aq1__	124
YOR267C	HRK1	4	psiblast	1aq1__	52
YOR267C	HRK1	5	psiblast	1aq1__	63
YOR267C	HRK1	6	unassigned	na	222
YOR268C	YOR268C	1	unassigned	na	132
YOR269W	PAC1	1	psiblast	1gxrA_	153
YOR269W	PAC1	2	psiblast	1erjA_	341
YOR270C	VPH1	1	psiblast	1i84S_	371
YOR270C	VPH1	2	msa	na	469
YOR271C	YOR271C	1	pfam	PF03820	327
YOR272W	YTM1	1	psiblast	1gg2B_	404
YOR272W	YTM1	2	psiblast	1erjA_	56

YOR273C	TPO4	1	unassigned	na	67
YOR273C	TPO4	2	psiblast	1ja5A_	412
YOR273C	TPO4	3	psiblast	1ja5A_	128
YOR273C	TPO4	4	unassigned	na	52
YOR274W	MOD5	1	fold_recogniti	1iqpA	128
YOR274W	MOD5	2	fold_recogniti	1iqpA	67
YOR274W	MOD5	3	fold_recogniti	1iqpA	110
YOR274W	MOD5	4	unassigned	na	123
YOR275C	RIM20	1	pfam	PF03097	148
YOR275C	RIM20	2	psiblast	1i84S_	320
YOR275C	RIM20	3	psiblast	1c1gA_	193
YOR276W	CAF20	1	unassigned	na	161
YOR277C	YOR277C	1	unassigned	na	102
YOR278W	HEM4	1	psiblast	1jr2A_	136
YOR278W	HEM4	2	psiblast	1jr2A_	139
YOR279C	RFM1	1	unassigned	na	68
YOR279C	RFM1	2	fold_recogniti	1ej6B	242
YOR280C	FSH3	1	psiblast	1fj2A_	266
YOR281C	PLP2	1	psiblast	1a0rP_	237
YOR281C	PLP2	2	psiblast	1bjx__	49
YOR282W	YOR282W	1	unassigned	na	106
YOR283W	YOR283W	1	psiblast	1ljdA_	230
YOR284W	HUA2	1	unassigned	na	243
YOR285W	YOR285W	1	psiblast	1h4kX_	139
YOR286W	FMP31	1	psiblast	1orb__	149
YOR287C	YOR287C	1	fold_recogniti	1c1gB	300
YOR288C	MPD1	1	psiblast	1mek__	156
YOR288C	MPD1	2	psiblast	1bjx__	162
YOR289W	YOR289W	1	pfam	PF01871	251
YOR290C	SNF2	1	msa	na	404
YOR290C	SNF2	2	unassigned	na	304
YOR290C	SNF2	3	psiblast	1gm5A_	254
YOR290C	SNF2	4	unassigned	na	52
YOR290C	SNF2	5	psiblast	1hv8A_	72
YOR290C	SNF2	6	psiblast	1d9xA_	261
YOR290C	SNF2	7	unassigned	na	163
YOR290C	SNF2	8	psiblast	1e6iA_	193
YOR291W	YOR291W	1	unassigned	na	414
YOR291W	YOR291W	2	psiblast	1eulA_	198
YOR291W	YOR291W	3	psiblast	1eulA_	364
YOR291W	YOR291W	4	psiblast	1eulA_	254
YOR291W	YOR291W	5	psiblast	1eulA_	242
YOR292C	YOR292C	1	unassigned	na	163
YOR292C	YOR292C	2	msa	na	146
YOR293W	RPS10A	1	pfam	PF03501	105
YOR294W	RRS1	1	msa	na	135
YOR294W	RRS1	2	unassigned	na	68
YOR295W	UAF30	1	msa	na	99

YOR295W	UAF30	2	pfam	PF02201	129
YOR296W	YOR296W	1	unassigned	na	224
YOR296W	YOR296W	2	fold_recogniti	1n1bA	296
YOR296W	YOR296W	3	fold_recogniti	1n1bA	81
YOR296W	YOR296W	4	fold_recogniti	1n1bA	119
YOR296W	YOR296W	5	psiblast	1dqvA_	118
YOR296W	YOR296W	6	psiblast	1dqvA_	194
YOR296W	YOR296W	7	unassigned	na	257
YOR297C	TIM18	1	unassigned	na	192
YOR298C-A	MBF1	1	fold_recogniti	1b0nA	58
YOR298W	YOR298W	1	unassigned	na	479
YOR299W	BUD7	1	unassigned	na	363
YOR299W	BUD7	2	psiblast	1na3A_	109
YOR299W	BUD7	3	msa	na	274
YOR300W	YOR300W	1	unassigned	na	102
YOR302W	YOR302W	1	unassigned	na	25
YOR303W	CPA1	1	psiblast	1bxrB_	158
YOR303W	CPA1	2	psiblast	1bxrB_	253
YOR304C-A	YOR304C-A	1	unassigned	na	76
YOR304W	ISW2	1	msa	na	170
YOR304W	ISW2	2	pfam	PF00176	262
YOR304W	ISW2	3	psiblast	1hv8A_	62
YOR304W	ISW2	4	psiblast	1d9xA_	228
YOR304W	ISW2	5	msa	na	167
YOR304W	ISW2	6	msa	na	231
YOR305W	YOR305W	1	unassigned	na	242
YOR306C	MCH5	1	unassigned	na	82
YOR306C	MCH5	2	pfam	PF00083	439
YOR307C	SLY41	1	unassigned	na	108
YOR307C	SLY41	2	msa	na	345
YOR308C	SNU66	1	psiblast	1i84S_	411
YOR308C	SNU66	2	msa	na	176
YOR309C	YOR309C	1	unassigned	na	126
YOR310C	NOP58	1	fold_recogniti	1n1bA	383
YOR310C	NOP58	2	psiblast	1i84S_	128
YOR311C	HSD1	1	unassigned	na	75
YOR311C	HSD1	2	pfam	PF01879	215
YOR312C	RPL20B	1	pfam	PF01775	174
YOR313C	SPS4	1	unassigned	na	338
YOR314W	YOR314W	1	unassigned	na	109
YOR314W-A	YOR314W-A	1	unassigned	na	36
YOR315W	YOR315W	1	unassigned	na	346
YOR316C	COT1	1	pfam	PF01545	439
YOR317W	FAA1	1	unassigned	na	79
YOR317W	FAA1	2	psiblast	1ba3__	174
YOR317W	FAA1	3	psiblast	1ba3__	301
YOR317W	FAA1	4	psiblast	1ba3__	146
YOR318C	YOR318C	1	unassigned	na	101

YOR319W	HSH49	1	psiblast	1cvjA_	88
YOR319W	HSH49	2	psiblast	1cvjA_	125
YOR320C	GNT1	1	unassigned	na	82
YOR320C	GNT1	2	psiblast	1ll0A_	258
YOR320C	GNT1	3	unassigned	na	151
YOR322C	YOR322C	1	unassigned	na	79
YOR322C	YOR322C	2	msa	na	308
YOR322C	YOR322C	3	unassigned	na	431
YOR323C	PRO2	1	psiblast	1o20A_	292
YOR323C	PRO2	2	psiblast	1o20A_	164
YOR324C	FRT1	1	unassigned	na	154
YOR324C	FRT1	2	psiblast	1i84S_	448
YOR325W	YOR325W	1	unassigned	na	157
YOR326W	MYO2	1	psiblast	1jwyA_	63
YOR326W	MYO2	2	psiblast	1jwyA_	360
YOR326W	MYO2	3	psiblast	1jwyA_	264
YOR326W	MYO2	4	psiblast	1jwyA_	96
YOR326W	MYO2	5	psiblast	1wdcA_	61
YOR326W	MYO2	6	psiblast	1cii__	165
YOR326W	MYO2	7	psiblast	1cii__	318
YOR326W	MYO2	8	unassigned	na	53
YOR326W	MYO2	9	pfam	PF01843	129
YOR326W	MYO2	10	unassigned	na	65
YOR327C	SNC2	1	psiblast	1kilA_	115
YOR328W	PDR10	1	unassigned	na	111
YOR328W	PDR10	2	psiblast	1mt0A_	124
YOR328W	PDR10	3	psiblast	1mt0A_	215
YOR328W	PDR10	4	msa	na	261
YOR328W	PDR10	5	unassigned	na	173
YOR328W	PDR10	6	psiblast	1l2tA_	289
YOR328W	PDR10	7	msa	na	302
YOR328W	PDR10	8	unassigned	na	89
YOR329C	SCD5	1	unassigned	na	205
YOR329C	SCD5	2	msa	na	330
YOR329C	SCD5	3	psiblast	1m2vB_	177
YOR329C	SCD5	4	unassigned	na	160
YOR330C	MIP1	1	msa	na	241
YOR330C	MIP1	2	unassigned	na	152
YOR330C	MIP1	3	psiblast	1d8yA_	289
YOR330C	MIP1	4	psiblast	1d8yA_	257
YOR330C	MIP1	5	psiblast	1d8yA_	72
YOR330C	MIP1	6	msa	na	269
YOR331C	YOR331C	1	unassigned	na	185
YOR332W	VMA4	1	pfam	PF01991	233
YOR333C	YOR333C	1	unassigned	na	138
YOR335C	ALA1	1	pfam	PF01411	570
YOR335C	ALA1	2	psiblast	1qf6A_	319
YOR335C	ALA1	3	msa	na	69

YOR336W	KRE5	1	msa	na	284
YOR336W	KRE5	2	unassigned	na	477
YOR336W	KRE5	3	msa	na	266
YOR336W	KRE5	4	unassigned	na	61
YOR336W	KRE5	5	psiblast	1g9rA_	277
YOR337W	TEA1	1	psiblast	1pyiA_	176
YOR337W	TEA1	2	unassigned	na	65
YOR337W	TEA1	3	msa	na	518
YOR338W	YOR338W	1	msa	na	96
YOR338W	YOR338W	2	msa	na	267
YOR339C	UBC11	1	psiblast	2e2c__	156
YOR340C	RPA43	1	msa	na	183
YOR340C	RPA43	2	unassigned	na	143
YOR341W	RPA190	1	msa	na	103
YOR341W	RPA190	2	psiblast	1i3qA_	1561
YOR342C	YOR342C	1	unassigned	na	319
YOR343C	YOR343C	1	unassigned	na	108
YOR344C	TYE7	1	msa	na	177
YOR344C	TYE7	2	psiblast	1am9A_	114
YOR345C	YOR345C	1	unassigned	na	116
YOR346W	REV1	1	unassigned	na	103
YOR346W	REV1	2	psiblast	1n5oX_	148
YOR346W	REV1	3	unassigned	na	91
YOR346W	REV1	4	psiblast	1jxlA_	122
YOR346W	REV1	5	psiblast	1jxlA_	155
YOR346W	REV1	6	psiblast	1jxlA_	188
YOR346W	REV1	7	unassigned	na	178
YOR347C	PYK2	1	psiblast	1a3wA_	266
YOR347C	PYK2	2	psiblast	1a3wA_	240
YOR349W	CIN1	1	unassigned	na	180
YOR349W	CIN1	2	msa	na	272
YOR349W	CIN1	3	unassigned	na	293
YOR349W	CIN1	4	msa	na	269
YOR350C	MNE1	1	msa	na	240
YOR350C	MNE1	2	unassigned	na	423
YOR351C	MEK1	1	psiblast	1gxcA_	138
YOR351C	MEK1	2	psiblast	1a06__	133
YOR351C	MEK1	3	psiblast	1a06__	226
YOR352W	YOR352W	1	unassigned	na	343
YOR353C	SOG2	1	psiblast	1k14A_	199
YOR353C	SOG2	2	psiblast	1dceA_	117
YOR353C	SOG2	3	unassigned	na	475
YOR354C	MSC6	1	unassigned	na	692
YOR355W	GDS1	1	unassigned	na	129
YOR355W	GDS1	2	msa	na	393
YOR356W	YOR356W	1	psiblast	1cc4A_	298
YOR356W	YOR356W	2	psiblast	1cc4A_	201
YOR356W	YOR356W	3	msa	na	132

YOR357C	GRD19	1	psiblast	1h6hA_	162
YOR358W	HAP5	1	msa	na	142
YOR358W	HAP5	2	psiblast	1n1jB_	100
YOR359W	VTS1	1	msa	na	416
YOR359W	VTS1	2	msa	na	107
YOR360C	PDE2	1	unassigned	na	133
YOR360C	PDE2	2	psiblast	1oymA_	237
YOR360C	PDE2	3	psiblast	1oymA_	156
YOR362C	PRE10	1	psiblast	1fntG_	288
YOR363C	PIP2	1	psiblast	1hwtD_	111
YOR363C	PIP2	2	unassigned	na	139
YOR363C	PIP2	3	msa	na	325
YOR363C	PIP2	4	unassigned	na	421
YOR364W	YOR364W	1	unassigned	na	122
YOR365C	YOR365C	1	msa	na	182
YOR365C	YOR365C	2	unassigned	na	147
YOR365C	YOR365C	3	msa	na	311
YOR365C	YOR365C	4	unassigned	na	63
YOR366W	YOR366W	1	unassigned	na	117
YOR367W	SCP1	1	psiblast	1h67A_	200
YOR368W	RAD17	1	psiblast	1ge8A_	148
YOR368W	RAD17	2	psiblast	1ge8A_	186
YOR368W	RAD17	3	unassigned	na	67
YOR369C	RPS12	1	psiblast	1e7kA_	143
YOR370C	MRS6	1	psiblast	1gnd__	494
YOR370C	MRS6	2	unassigned	na	109
YOR371C	GPB1	1	unassigned	na	645
YOR371C	GPB1	2	msa	na	149
YOR371C	GPB1	3	unassigned	na	103
YOR372C	NDD1	1	unassigned	na	554
YOR373W	NUD1	1	unassigned	na	90
YOR373W	NUD1	2	psiblast	1ds9A_	58
YOR373W	NUD1	3	psiblast	1m9sA_	252
YOR373W	NUD1	4	unassigned	na	64
YOR373W	NUD1	5	psiblast	1o6sA_	387
YOR374W	ALD4	1	psiblast	1cw3A_	330
YOR374W	ALD4	2	psiblast	1cw3A_	189
YOR375C	GDH1	1	psiblast	1bgvA_	223
YOR375C	GDH1	2	psiblast	1bgvA_	231
YOR376W	YOR376W	1	unassigned	na	122
YOR377W	ATF1	1	unassigned	na	195
YOR377W	ATF1	2	msa	na	330
YOR378W	YOR378W	1	msa	na	268
YOR378W	YOR378W	2	msa	na	247
YOR379C	YOR379C	1	unassigned	na	112
YOR380W	RDR1	1	psiblast	1zmeC_	99
YOR380W	RDR1	2	msa	na	447
YOR381W	FRE3	1	psiblast	1g8iA_	188

YOR381W	FRE3	2	msa	na	168
YOR381W	FRE3	3	psiblast	1ndh__	173
YOR381W	FRE3	4	psiblast	1ndh__	182
YOR382W	FIT2	1	unassigned	na	153
YOR383C	FIT3	1	msa	na	204
YOR384W	FRE5	1	psiblast	5tnc__	185
YOR384W	FRE5	2	msa	na	160
YOR384W	FRE5	3	psiblast	1cqxA_	179
YOR384W	FRE5	4	psiblast	1cqxA_	170
YOR385W	YOR385W	1	unassigned	na	290
YOR386W	PHR1	1	unassigned	na	71
YOR386W	PHR1	2	psiblast	1dnpA_	178
YOR386W	PHR1	3	psiblast	1dnpA_	151
YOR386W	PHR1	4	psiblast	1dnpA_	165
YOR387C	YOR387C	1	unassigned	na	206
YOR388C	FDH1	1	psiblast	2nacA_	173
YOR388C	FDH1	2	psiblast	2nacA_	203
YOR389W	YOR389W	1	msa	na	489
YOR389W	YOR389W	2	msa	na	135
YOR390W	YOR390W	1	unassigned	na	171
YOR390W	YOR390W	2	pfam	PF02537	204
YOR391C	HSP33	1	psiblast	1n57A_	237
YOR392W	YOR392W	1	unassigned	na	147
YOR393W	ERR1	1	psiblast	1els__	127
YOR393W	ERR1	2	psiblast	1els__	310
YOR394W	YOR394W	1	pfam	PF00660	164
YPL001W	HAT1	1	psiblast	1bob__	112
YPL001W	HAT1	2	psiblast	1bob__	156
YPL001W	HAT1	3	psiblast	1bob__	106
YPL002C	SNF8	1	msa	na	233
YPL004C	LSP1	1	msa	na	282
YPL004C	LSP1	2	unassigned	na	59
YPL005W	AEP3	1	msa	na	138
YPL005W	AEP3	2	msa	na	207
YPL005W	AEP3	3	psiblast	1na0A_	118
YPL005W	AEP3	4	msa	na	143
YPL006W	NCR1	1	msa	na	284
YPL006W	NCR1	2	psiblast	1oy6A_	274
YPL006W	NCR1	3	psiblast	1oy6A_	328
YPL006W	NCR1	4	unassigned	na	91
YPL006W	NCR1	5	msa	na	193
YPL007C	TFC8	1	unassigned	na	588
YPL008W	CHL1	1	msa	na	75
YPL008W	CHL1	2	unassigned	na	73
YPL008W	CHL1	3	psiblast	1hv8A_	115
YPL008W	CHL1	4	unassigned	na	147
YPL008W	CHL1	5	msa	na	451
YPL009C	YPL009C	1	psiblast	1i84S_	254

YPL009C	YPL009C	2	psiblast	1i84S_	315
YPL009C	YPL009C	3	msa	na	131
YPL009C	YPL009C	4	msa	na	228
YPL009C	YPL009C	5	unassigned	na	110
YPL010W	RET3	1	psiblast	1gw5M_	189
YPL011C	TAF3	1	msa	na	152
YPL011C	TAF3	2	unassigned	na	201
YPL013C	MRPS16	1	psiblast	1emwA_	121
YPL014W	YPL014W	1	unassigned	na	381
YPL015C	HST2	1	psiblast	1j8fA_	262
YPL015C	HST2	2	psiblast	1j8fA_	95
YPL016W	SWI1	1	psiblast	1epwA_	405
YPL016W	SWI1	2	psiblast	1kkxA_	143
YPL016W	SWI1	3	unassigned	na	360
YPL016W	SWI1	4	msa	na	158
YPL016W	SWI1	5	unassigned	na	248
YPL017C	YPL017C	1	psiblast	1jehA_	188
YPL017C	YPL017C	2	psiblast	1jehA_	183
YPL017C	YPL017C	3	psiblast	1jehA_	128
YPL018W	CTF19	1	unassigned	na	369
YPL019C	VTC3	1	pfam	PF03105	179
YPL019C	VTC3	2	unassigned	na	75
YPL019C	VTC3	3	msa	na	298
YPL019C	VTC3	4	unassigned	na	107
YPL019C	VTC3	5	msa	na	176
YPL020C	ULP1	1	unassigned	na	153
YPL020C	ULP1	2	msa	na	110
YPL020C	ULP1	3	unassigned	na	128
YPL020C	ULP1	4	psiblast	1euvA_	230
YPL021W	ECM23	1	unassigned	na	116
YPL021W	ECM23	2	psiblast	4gatA_	71
YPL022W	RAD1	1	psiblast	1qdeA_	299
YPL022W	RAD1	2	psiblast	1gm5A_	286
YPL022W	RAD1	3	psiblast	1gm5A_	157
YPL022W	RAD1	4	psiblast	1d9xA_	77
YPL022W	RAD1	5	psiblast	1j22A_	170
YPL022W	RAD1	6	msa	na	111
YPL023C	MET12	1	psiblast	1b5tA_	324
YPL023C	MET12	2	unassigned	na	69
YPL023C	MET12	3	msa	na	264
YPL024W	NCE4	1	unassigned	na	241
YPL025C	YPL025C	1	unassigned	na	185
YPL026C	SKS1	1	psiblast	1ql6A_	126
YPL026C	SKS1	2	psiblast	1ql6A_	65
YPL026C	SKS1	3	psiblast	1ql6A_	165
YPL026C	SKS1	4	psiblast	1niwA_	146
YPL027W	SMA1	1	unassigned	na	245
YPL028W	ERG10	1	psiblast	1dluA_	221

YPL028W	ERG10	2	psiblast	1dluA_	177
YPL029W	SUV3	1	unassigned	na	135
YPL029W	SUV3	2	psiblast	1fuuA_	407
YPL029W	SUV3	3	psiblast	1d2mA_	113
YPL029W	SUV3	4	unassigned	na	82
YPL030W	YPL030W	1	unassigned	na	165
YPL030W	YPL030W	2	msa	na	402
YPL031C	PHO85	1	psiblast	1aq1__	84
YPL031C	PHO85	2	psiblast	1aq1__	92
YPL031C	PHO85	3	psiblast	1aq1__	51
YPL031C	PHO85	4	psiblast	1aq1__	78
YPL032C	SVL3	1	psiblast	1ks9A_	213
YPL032C	SVL3	2	psiblast	1ks9A_	132
YPL032C	SVL3	3	psiblast	1i84S_	205
YPL032C	SVL3	4	msa	na	275
YPL033C	YPL033C	1	psiblast	1i01A_	281
YPL034W	YPL034W	1	unassigned	na	165
YPL035C	YPL035C	1	unassigned	na	115
YPL036W	PMA2	1	psiblast	1mhsA_	183
YPL036W	PMA2	2	psiblast	1mhsA_	213
YPL036W	PMA2	3	psiblast	1mhsA_	316
YPL036W	PMA2	4	psiblast	1mhsA_	235
YPL037C	EGD1	1	pfam	PF01849	157
YPL038W	MET31	1	psiblast	1meyG_	177
YPL039W	YPL039W	1	unassigned	na	316
YPL040C	ISM1	1	psiblast	1ffyA_	113
YPL040C	ISM1	2	psiblast	1ffyA_	362
YPL040C	ISM1	3	psiblast	1ffyA_	274
YPL040C	ISM1	4	psiblast	1ffyA_	253
YPL041C	YPL041C	1	unassigned	na	207
YPL042C	SSN3	1	psiblast	1opkA_	416
YPL042C	SSN3	2	psiblast	1b38A_	56
YPL042C	SSN3	3	unassigned	na	83
YPL043W	NOP4	1	psiblast	1l3kA_	123
YPL043W	NOP4	2	psiblast	1l3kA_	132
YPL043W	NOP4	3	psiblast	1cvjA_	128
YPL043W	NOP4	4	psiblast	1cvjA_	117
YPL043W	NOP4	5	psiblast	2up1A_	133
YPL043W	NOP4	6	unassigned	na	52
YPL044C	YPL044C	1	unassigned	na	182
YPL045W	VPS16	1	msa	na	432
YPL045W	VPS16	2	unassigned	na	187
YPL045W	VPS16	3	msa	na	179
YPL046C	ELC1	1	psiblast	1hv2A_	47
YPL046C	ELC1	2	psiblast	1hv2A_	52
YPL047W	SGF11	1	unassigned	na	99
YPL049C	DIG1	1	unassigned	na	452
YPL050C	MNN9	1	pfam	PF03452	395

YPL051W	ARL3	1	psiblast	1ksgA_	198
YPL052W	YPL052W	1	unassigned	na	201
YPL053C	KTR6	1	pfam	PF01793	446
YPL054W	LEE1	1	psiblast	1m9oA_	118
YPL054W	LEE1	2	msa	na	183
YPL055C	LGE1	1	unassigned	na	332
YPL056C	YPL056C	1	unassigned	na	101
YPL058C	PDR12	1	unassigned	na	85
YPL058C	PDR12	2	psiblast	1jj7A_	336
YPL058C	PDR12	3	unassigned	na	417
YPL058C	PDR12	4	psiblast	1mt0A_	57
YPL058C	PDR12	5	psiblast	1mt0A_	196
YPL058C	PDR12	6	msa	na	298
YPL058C	PDR12	7	unassigned	na	122
YPL059W	GRX5	1	psiblast	1kte__	150
YPL060W	LPE10	1	msa	na	117
YPL060W	LPE10	2	msa	na	296
YPL061W	ALD6	1	psiblast	1nzxA_	310
YPL061W	ALD6	2	psiblast	1nzxA_	190
YPL062W	YPL062W	1	unassigned	na	134
YPL063W	TIM50	1	msa	na	163
YPL063W	TIM50	2	pfam	PF03031	219
YPL063W	TIM50	3	unassigned	na	94
YPL064C	CWC27	1	psiblast	1ak4A_	197
YPL064C	CWC27	2	unassigned	na	104
YPL065W	VPS28	1	msa	na	242
YPL066W	YPL066W	1	unassigned	na	479
YPL067C	YPL067C	1	unassigned	na	198
YPL068C	YPL068C	1	unassigned	na	293
YPL069C	BTS1	1	psiblast	1ubx__	168
YPL069C	BTS1	2	psiblast	1ubx__	167
YPL070W	MUK1	1	msa	na	159
YPL070W	MUK1	2	unassigned	na	109
YPL070W	MUK1	3	pfam	PF02204	193
YPL070W	MUK1	4	unassigned	na	151
YPL071C	YPL071C	1	unassigned	na	156
YPL072W	UBP16	1	psiblast	1nbfA_	447
YPL072W	UBP16	2	unassigned	na	52
YPL073C	YPL073C	1	unassigned	na	161
YPL074W	YTA6	1	msa	na	215
YPL074W	YTA6	2	psiblast	1ksfX_	159
YPL074W	YTA6	3	psiblast	1ksfX_	144
YPL074W	YTA6	4	psiblast	1e32A_	129
YPL074W	YTA6	5	psiblast	1e32A_	107
YPL076W	GPI2	1	msa	na	184
YPL076W	GPI2	2	unassigned	na	96
YPL077C	YPL077C	1	unassigned	na	100
YPL077C	YPL077C	2	msa	na	140

YPL078C	ATP4	1	msa	na	89
YPL078C	ATP4	2	msa	na	155
YPL079W	RPL21B	1	psiblast	1ffkN_	160
YPL080C	YPL080C	1	unassigned	na	108
YPL081W	RPS9A	1	psiblast	1c05A_	105
YPL081W	RPS9A	2	psiblast	1c05A_	92
YPL082C	MOT1	1	msa	na	266
YPL082C	MOT1	2	unassigned	na	194
YPL082C	MOT1	3	msa	na	229
YPL082C	MOT1	4	unassigned	na	329
YPL082C	MOT1	5	msa	na	180
YPL082C	MOT1	6	unassigned	na	71
YPL082C	MOT1	7	pfam	PF00176	314
YPL082C	MOT1	8	unassigned	na	89
YPL082C	MOT1	9	pfam	PF00271	74
YPL082C	MOT1	10	msa	na	121
YPL083C	SEN54	1	msa	na	381
YPL083C	SEN54	2	unassigned	na	86
YPL084W	BRO1	1	pfam	PF03097	163
YPL084W	BRO1	2	unassigned	na	128
YPL084W	BRO1	3	psiblast	1c1gA_	93
YPL084W	BRO1	4	psiblast	1i84S_	368
YPL084W	BRO1	5	unassigned	na	92
YPL086C	ELP3	1	msa	na	423
YPL086C	ELP3	2	pfam	PF00583	134
YPL087W	YDC1	1	msa	na	317
YPL088W	YPL088W	1	psiblast	1exbA_	279
YPL088W	YPL088W	2	psiblast	1exbA_	63
YPL089C	RLM1	1	psiblast	1egwA_	80
YPL089C	RLM1	2	msa	na	180
YPL089C	RLM1	3	msa	na	189
YPL089C	RLM1	4	unassigned	na	82
YPL089C	RLM1	5	msa	na	145
YPL091W	GLR1	1	psiblast	1grt__	192
YPL091W	GLR1	2	psiblast	1grt__	164
YPL091W	GLR1	3	psiblast	1grt__	127
YPL092W	SSU1	1	msa	na	339
YPL092W	SSU1	2	unassigned	na	119
YPL093W	NOG1	1	psiblast	1mkyA_	161
YPL093W	NOG1	2	psiblast	1mkyA_	183
YPL093W	NOG1	3	psiblast	1mkyA_	96
YPL093W	NOG1	4	msa	na	207
YPL094C	SEC62	1	pfam	PF03839	283
YPL095C	YPL095C	1	msa	na	130
YPL095C	YPL095C	2	psiblast	1a8s__	326
YPL096W	PNG1	1	msa	na	133
YPL096W	PNG1	2	pfam	PF01841	144
YPL096W	PNG1	3	msa	na	86

YPL097W	MSY1	1	psiblast	1jiiA_	297
YPL097W	MSY1	2	psiblast	1jiiA_	195
YPL098C	YPL098C	1	unassigned	na	113
YPL099C	FMP14	1	unassigned	na	182
YPL100W	ATG21	1	psiblast	1nexB_	366
YPL100W	ATG21	2	psiblast	1k8kC_	57
YPL100W	ATG21	3	psiblast	1erjA_	73
YPL101W	ELP4	1	msa	na	302
YPL101W	ELP4	2	msa	na	154
YPL102C	YPL102C	1	unassigned	na	100
YPL103C	FMP30	1	unassigned	na	174
YPL103C	FMP30	2	psiblast	1smlA_	164
YPL103C	FMP30	3	psiblast	1smlA_	130
YPL104W	MSD1	1	psiblast	1eqrA_	139
YPL104W	MSD1	2	psiblast	1eqrA_	519
YPL105C	YPL105C	1	unassigned	na	143
YPL105C	YPL105C	2	pfam	PF02213	87
YPL105C	YPL105C	3	psiblast	1i84S_	360
YPL105C	YPL105C	4	unassigned	na	259
YPL106C	SSE1	1	psiblast	1hjoA_	192
YPL106C	SSE1	2	psiblast	1hjoA_	119
YPL106C	SSE1	3	psiblast	1hjoA_	75
YPL106C	SSE1	4	psiblast	1dkyA_	180
YPL106C	SSE1	5	psiblast	1dkyA_	127
YPL107W	YPL107W	1	msa	na	192
YPL107W	YPL107W	2	unassigned	na	56
YPL108W	YPL108W	1	unassigned	na	168
YPL109C	YPL109C	1	msa	na	155
YPL109C	YPL109C	2	psiblast	1mruA_	126
YPL109C	YPL109C	3	psiblast	1mruA_	97
YPL109C	YPL109C	4	psiblast	1mruA_	165
YPL110C	YPL110C	1	pfam	PF03105	202
YPL110C	YPL110C	2	unassigned	na	60
YPL110C	YPL110C	3	psiblast	1n11A_	104
YPL110C	YPL110C	4	psiblast	1n11A_	145
YPL110C	YPL110C	5	psiblast	1n11A_	169
YPL110C	YPL110C	6	psiblast	1sw6A_	68
YPL110C	YPL110C	7	unassigned	na	109
YPL110C	YPL110C	8	psiblast	1o1zA_	182
YPL110C	YPL110C	9	pfam	PF03009	184
YPL111W	CAR1	1	psiblast	1hqxA_	333
YPL112C	PEX25	1	unassigned	na	84
YPL112C	PEX25	2	msa	na	310
YPL113C	YPL113C	1	psiblast	1gdhA_	153
YPL113C	YPL113C	2	psiblast	1gdhA_	243
YPL114W	YPL114W	1	unassigned	na	139
YPL115C	BEM3	1	unassigned	na	155
YPL115C	BEM3	2	msa	na	356

YPL115C	BEM3	3	pfam	PF00787	118
YPL115C	BEM3	4	pfam	PF00169	119
YPL115C	BEM3	5	unassigned	na	124
YPL115C	BEM3	6	psiblast	1am4A_	137
YPL115C	BEM3	7	psiblast	1am4A_	119
YPL116W	HOS3	1	psiblast	1c3pA_	224
YPL116W	HOS3	2	psiblast	1c3pA_	241
YPL116W	HOS3	3	msa	na	232
YPL117C	IDI1	1	psiblast	1nfsA_	288
YPL118W	MRP51	1	msa	na	101
YPL118W	MRP51	2	unassigned	na	243
YPL119C	DBP1	1	msa	na	146
YPL119C	DBP1	2	psiblast	1hv8A_	235
YPL119C	DBP1	3	psiblast	1hv8A_	176
YPL119C	DBP1	4	unassigned	na	60
YPL120W	VPS30	1	psiblast	1i84S_	339
YPL120W	VPS30	2	msa	na	125
YPL120W	VPS30	3	unassigned	na	93
YPL121C	MEI5	1	unassigned	na	222
YPL122C	TFB2	1	pfam	PF03849	414
YPL122C	TFB2	2	unassigned	na	99
YPL123C	RNY1	1	psiblast	1bolA_	299
YPL123C	RNY1	2	unassigned	na	135
YPL124W	SPC29	1	unassigned	na	253
YPL125W	KAP120	1	msa	na	702
YPL125W	KAP120	2	msa	na	214
YPL125W	KAP120	3	msa	na	116
YPL126W	NAN1	1	psiblast	1nexB_	172
YPL126W	NAN1	2	psiblast	1erjA_	364
YPL126W	NAN1	3	psiblast	1gg2B_	96
YPL126W	NAN1	4	psiblast	1l0qA_	264
YPL127C	HHO1	1	psiblast	1hstA_	142
YPL127C	HHO1	2	msa	na	116
YPL128C	TBF1	1	unassigned	na	112
YPL128C	TBF1	2	msa	na	272
YPL128C	TBF1	3	pfam	PF00249	113
YPL128C	TBF1	4	unassigned	na	65
YPL129W	TAF14	1	pfam	PF03366	154
YPL129W	TAF14	2	unassigned	na	90
YPL130W	SPO19	1	unassigned	na	223
YPL131W	RPL5	1	psiblast	1ffkK_	297
YPL132W	COX11	1	msa	na	300
YPL133C	RDS2	1	psiblast	1pyc__	55
YPL133C	RDS2	2	msa	na	142
YPL133C	RDS2	3	unassigned	na	249
YPL134C	ODC1	1	pfam	PF00153	117
YPL134C	ODC1	2	pfam	PF00153	95
YPL134C	ODC1	3	pfam	PF00153	98

YPL135W	ISU1	1	pfam	PF01592	165
YPL136W	YPL136W	1	unassigned	na	122
YPL137C	YPL137C	1	msa	na	498
YPL137C	YPL137C	2	psiblast	2bnh__	94
YPL137C	YPL137C	3	psiblast	2bnh__	52
YPL137C	YPL137C	4	psiblast	2bnh__	57
YPL137C	YPL137C	5	psiblast	2bnh__	216
YPL137C	YPL137C	6	msa	na	202
YPL137C	YPL137C	7	unassigned	na	157
YPL138C	SPP1	1	psiblast	1fp0A_	109
YPL138C	SPP1	2	psiblast	1e6iA_	192
YPL138C	SPP1	3	unassigned	na	52
YPL139C	UME1	1	unassigned	na	64
YPL139C	UME1	2	psiblast	1gg2B_	396
YPL140C	MKK2	1	psiblast	1f3mA_	77
YPL140C	MKK2	2	unassigned	na	93
YPL140C	MKK2	3	psiblast	1f3mC_	124
YPL140C	MKK2	4	psiblast	1f3mC_	212
YPL141C	YPL141C	1	psiblast	1ia8A_	114
YPL141C	YPL141C	2	psiblast	1ia8A_	76
YPL141C	YPL141C	3	psiblast	1ia8A_	180
YPL141C	YPL141C	4	unassigned	na	495
YPL142C	YPL142C	1	unassigned	na	105
YPL143W	RPL33A	1	pfam	PF01247	107
YPL144W	YPL144W	1	unassigned	na	148
YPL145C	KES1	1	pfam	PF01237	434
YPL146C	YPL146C	1	psiblast	1i84S_	385
YPL146C	YPL146C	2	unassigned	na	70
YPL147W	PXA1	1	unassigned	na	302
YPL147W	PXA1	2	psiblast	1jsqA_	268
YPL147W	PXA1	3	psiblast	1jsqA_	300
YPL148C	PPT2	1	pfam	PF01648	177
YPL149W	ATG5	1	msa	na	196
YPL149W	ATG5	2	msa	na	98
YPL150W	YPL150W	1	psiblast	1gznA_	94
YPL150W	YPL150W	2	psiblast	1gznA_	328
YPL150W	YPL150W	3	unassigned	na	479
YPL151C	PRP46	1	psiblast	1nexB_	367
YPL151C	PRP46	2	psiblast	1erjA_	84
YPL152W	RRD2	1	pfam	PF03095	358
YPL153C	RAD53	1	psiblast	1g3gA_	165
YPL153C	RAD53	2	psiblast	1a06__	157
YPL153C	RAD53	3	psiblast	1a06__	212
YPL153C	RAD53	4	psiblast	1qu5A_	219
YPL153C	RAD53	5	unassigned	na	68
YPL154C	PEP4	1	unassigned	na	74
YPL154C	PEP4	2	psiblast	1dp5A_	198
YPL154C	PEP4	3	psiblast	1dp5A_	133

YPL155C	KIP2	1	unassigned	na	71
YPL155C	KIP2	2	psiblast	1gojA_	436
YPL155C	KIP2	3	psiblast	1i84S_	199
YPL156C	PRM4	1	unassigned	na	118
YPL156C	PRM4	2	psiblast	1jhb__	166
YPL157W	TGS1	1	unassigned	na	96
YPL157W	TGS1	2	psiblast	1f38A_	167
YPL157W	TGS1	3	unassigned	na	52
YPL158C	YPL158C	1	unassigned	na	129
YPL158C	YPL158C	2	psiblast	1epwA_	629
YPL159C	PET20	1	msa	na	253
YPL160W	CDC60	1	psiblast	1ffyA_	71
YPL160W	CDC60	2	psiblast	1ffyA_	311
YPL160W	CDC60	3	psiblast	1ffyA_	279
YPL160W	CDC60	4	psiblast	1ffyA_	338
YPL160W	CDC60	5	msa	na	91
YPL161C	BEM4	1	unassigned	na	633
YPL162C	YPL162C	1	unassigned	na	88
YPL162C	YPL162C	2	msa	na	185
YPL163C	SVS1	1	unassigned	na	260
YPL165C	SET6	1	msa	na	84
YPL165C	SET6	2	psiblast	1ml9A_	201
YPL165C	SET6	3	psiblast	1ml9A_	88
YPL166W	YPL166W	1	unassigned	na	213
YPL167C	REV3	1	msa	na	496
YPL167C	REV3	2	psiblast	1tgoA_	358
YPL167C	REV3	3	psiblast	1tgoA_	242
YPL167C	REV3	4	psiblast	1tgoA_	118
YPL167C	REV3	5	psiblast	1tgoA_	180
YPL167C	REV3	6	msa	na	110
YPL168W	YPL168W	1	unassigned	na	430
YPL169C	MEX67	1	psiblast	1ft8A_	98
YPL169C	MEX67	2	psiblast	1ft8A_	162
YPL169C	MEX67	3	psiblast	1p8tA_	115
YPL169C	MEX67	4	unassigned	na	70
YPL169C	MEX67	5	psiblast	1jkgB_	154
YPL170W	DAP1	1	psiblast	1eueA_	152
YPL171C	OYE3	1	psiblast	1oya__	400
YPL172C	COX10	1	msa	na	83
YPL172C	COX10	2	unassigned	na	71
YPL172C	COX10	3	pfam	PF01040	308
YPL173W	MRPL40	1	pfam	PF00467	142
YPL173W	MRPL40	2	unassigned	na	155
YPL174C	NIP100	1	psiblast	1i84S_	342
YPL174C	NIP100	2	unassigned	na	103
YPL174C	NIP100	3	psiblast	1c1gA_	117
YPL174C	NIP100	4	psiblast	1c1gA_	228
YPL174C	NIP100	5	unassigned	na	78

YPL175W	SPT14	1	psiblast	1f0kA_	185
YPL175W	SPT14	2	psiblast	1f0kA_	128
YPL175W	SPT14	3	msa	na	148
YPL176C	YPL176C	1	unassigned	na	136
YPL176C	YPL176C	2	psiblast	1de4C_	322
YPL176C	YPL176C	3	psiblast	1de4C_	325
YPL177C	CUP9	1	msa	na	127
YPL177C	CUP9	2	psiblast	1b72B_	179
YPL178W	CBC2	1	psiblast	1h2tZ_	208
YPL179W	PPQ1	1	unassigned	na	72
YPL179W	PPQ1	2	psiblast	1a17__	159
YPL179W	PPQ1	3	psiblast	1fjmA_	318
YPL180W	TCO89	1	msa	na	568
YPL180W	TCO89	2	unassigned	na	231
YPL181W	CTI6	1	unassigned	na	68
YPL181W	CTI6	2	pfam	PF00628	59
YPL181W	CTI6	3	msa	na	379
YPL182C	YPL182C	1	unassigned	na	127
YPL183C	YPL183C	1	psiblast	1gg2B_	116
YPL183C	YPL183C	2	psiblast	1erjA_	303
YPL183C	YPL183C	3	psiblast	1l0qA_	79
YPL183C	YPL183C	4	msa	na	313
YPL183C	YPL183C	5	msa	na	202
YPL183W-A	GON5	1	psiblast	1dfeA_	93
YPL184C	YPL184C	1	msa	na	167
YPL184C	YPL184C	2	psiblast	1cvjA_	126
YPL184C	YPL184C	3	psiblast	1qm9A_	109
YPL184C	YPL184C	4	psiblast	1qm9A_	128
YPL184C	YPL184C	5	psiblast	1o0pA_	82
YPL185W	YPL185W	1	unassigned	na	131
YPL186C	UIP4	1	msa	na	208
YPL186C	UIP4	2	unassigned	na	96
YPL187W	MF(ALPHA)1	1	unassigned	na	165
YPL188W	POS5	1	unassigned	na	97
YPL188W	POS5	2	pfam	PF01513	317
YPL189W	GUP2	1	msa	na	156
YPL189W	GUP2	2	msa	na	453
YPL190C	NAB3	1	unassigned	na	116
YPL190C	NAB3	2	psiblast	1epwA_	284
YPL190C	NAB3	3	unassigned	na	158
YPL190C	NAB3	4	msa	na	244
YPL191C	YPL191C	1	msa	na	204
YPL191C	YPL191C	2	unassigned	na	156
YPL192C	PRM3	1	unassigned	na	133
YPL193W	RSA1	1	unassigned	na	381
YPL194W	DDC1	1	unassigned	na	612
YPL195W	APL5	1	psiblast	1gw5A_	94
YPL195W	APL5	2	psiblast	1b3uA_	172

YPL195W	APL5	3	psiblast	1b3uA_	64
YPL195W	APL5	4	psiblast	1b3uA_	172
YPL195W	APL5	5	psiblast	1b3uA_	151
YPL195W	APL5	6	unassigned	na	59
YPL195W	APL5	7	msa	na	220
YPL196W	OXR1	1	msa	na	95
YPL196W	OXR1	2	msa	na	178
YPL197C	YPL197C	1	unassigned	na	137
YPL198W	RPL7B	1	unassigned	na	78
YPL198W	RPL7B	2	psiblast	1ffkT_	166
YPL199C	YPL199C	1	unassigned	na	92
YPL199C	YPL199C	2	pfam	PF01713	148
YPL200W	CSM4	1	unassigned	na	156
YPL201C	YPL201C	1	unassigned	na	461
YPL202C	AFT2	1	unassigned	na	227
YPL202C	AFT2	2	msa	na	76
YPL202C	AFT2	3	unassigned	na	113
YPL203W	TPK2	1	psiblast	1fotA_	172
YPL203W	TPK2	2	psiblast	1fotA_	208
YPL204W	HRR25	1	psiblast	1ckiA_	69
YPL204W	HRR25	2	psiblast	1ckiA_	278
YPL204W	HRR25	3	unassigned	na	147
YPL205C	YPL205C	1	unassigned	na	114
YPL206C	YPL206C	1	psiblast	1o1zA_	321
YPL207W	YPL207W	1	unassigned	na	123
YPL207W	YPL207W	2	psiblast	4nosA_	50
YPL207W	YPL207W	3	psiblast	1j9zA_	215
YPL207W	YPL207W	4	psiblast	1j9zA_	57
YPL207W	YPL207W	5	msa	na	283
YPL207W	YPL207W	6	msa	na	82
YPL208W	YPL208W	1	psiblast	1mlvA_	246
YPL208W	YPL208W	2	psiblast	1mlvA_	95
YPL208W	YPL208W	3	psiblast	1mlvA_	171
YPL208W	YPL208W	4	unassigned	na	71
YPL209C	IPL1	1	unassigned	na	75
YPL209C	IPL1	2	psiblast	1fotA_	95
YPL209C	IPL1	3	psiblast	1fotA_	197
YPL210C	SRP72	1	psiblast	1fchA_	139
YPL210C	SRP72	2	psiblast	1fchA_	110
YPL210C	SRP72	3	psiblast	1kt1A_	129
YPL210C	SRP72	4	msa	na	202
YPL210C	SRP72	5	msa	na	66
YPL211W	NIP7	1	pfam	PF03657	181
YPL212C	PUS1	1	unassigned	na	64
YPL212C	PUS1	2	psiblast	1dj0A_	137
YPL212C	PUS1	3	psiblast	1dj0A_	105
YPL212C	PUS1	4	msa	na	238
YPL213W	LEA1	1	psiblast	1a9nA_	238

YPL214C	THI6	1	psiblast	1g4tA_	233
YPL214C	THI6	2	psiblast	1c3qA_	307
YPL215W	CBP3	1	unassigned	na	65
YPL215W	CBP3	2	msa	na	270
YPL216W	YPL216W	1	msa	na	433
YPL216W	YPL216W	2	unassigned	na	669
YPL217C	BMS1	1	msa	na	174
YPL217C	BMS1	2	unassigned	na	155
YPL217C	BMS1	3	psiblast	1i84S_	364
YPL217C	BMS1	4	msa	na	289
YPL217C	BMS1	5	msa	na	114
YPL217C	BMS1	6	unassigned	na	87
YPL218W	SAR1	1	psiblast	1m2oB_	190
YPL219W	PCL8	1	unassigned	na	290
YPL219W	PCL8	2	msa	na	202
YPL221W	BOP1	1	msa	na	216
YPL221W	BOP1	2	unassigned	na	78
YPL221W	BOP1	3	msa	na	307
YPL221W	BOP1	4	msa	na	192
YPL222W	FMP40	1	pfam	PF02696	688
YPL223C	GRE1	1	unassigned	na	168
YPL224C	MMT2	1	unassigned	na	116
YPL224C	MMT2	2	pfam	PF01545	333
YPL225W	YPL225W	1	unassigned	na	146
YPL226W	NEW1	1	msa	na	172
YPL226W	NEW1	2	unassigned	na	108
YPL226W	NEW1	3	psiblast	1ii8B_	147
YPL226W	NEW1	4	psiblast	1ii8B_	71
YPL226W	NEW1	5	unassigned	na	66
YPL226W	NEW1	6	psiblast	1oxsC_	203
YPL226W	NEW1	7	psiblast	1jsqA_	266
YPL226W	NEW1	8	msa	na	163
YPL227C	ALG5	1	unassigned	na	67
YPL227C	ALG5	2	psiblast	1h7lA_	150
YPL227C	ALG5	3	msa	na	117
YPL228W	CET1	1	unassigned	na	219
YPL228W	CET1	2	psiblast	1d8hA_	330
YPL229W	YPL229W	1	unassigned	na	100
YPL229W	YPL229W	2	msa	na	106
YPL230W	YPL230W	1	psiblast	1a1iA_	115
YPL230W	YPL230W	2	msa	na	56
YPL230W	YPL230W	3	msa	na	76
YPL230W	YPL230W	4	msa	na	61
YPL230W	YPL230W	5	psiblast	2adr__	83
YPL231W	FAS2	1	msa	na	217
YPL231W	FAS2	2	unassigned	na	417
YPL231W	FAS2	3	psiblast	1gz6A_	262
YPL231W	FAS2	4	msa	na	230

YPL231W	FAS2	5	psiblast	1e5mA_	306
YPL231W	FAS2	6	psiblast	1e5mA_	151
YPL231W	FAS2	7	msa	na	78
YPL231W	FAS2	8	unassigned	na	90
YPL231W	FAS2	9	psiblast	1f80A_	136
YPL232W	SSO1	1	psiblast	1fioA_	235
YPL232W	SSO1	2	psiblast	1fioA_	55
YPL233W	NSL1	1	fold_recogniti	1cunA	132
YPL233W	NSL1	2	fold_recogniti	1cunA	84
YPL234C	TFP3	1	pfam	PF00137	85
YPL234C	TFP3	2	pfam	PF00137	79
YPL235W	RVB2	1	psiblast	1e32A_	224
YPL235W	RVB2	2	psiblast	1e32A_	58
YPL235W	RVB2	3	psiblast	1jr3A_	189
YPL236C	YPL236C	1	psiblast	1muoA_	121
YPL236C	YPL236C	2	psiblast	1muoA_	243
YPL237W	SUI3	1	msa	na	124
YPL237W	SUI3	2	psiblast	1k8bA_	93
YPL237W	SUI3	3	psiblast	1k81A_	68
YPL238C	YPL238C	1	unassigned	na	129
YPL239W	YAR1	1	psiblast	1n0rA_	121
YPL239W	YAR1	2	psiblast	1n0rA_	79
YPL240C	HSP82	1	psiblast	1a4h__	219
YPL240C	HSP82	2	pfam	PF00183	490
YPL241C	CIN2	1	unassigned	na	268
YPL242C	IQG1	1	unassigned	na	86
YPL242C	IQG1	2	psiblast	1i84S_	589
YPL242C	IQG1	3	psiblast	1i84S_	298
YPL242C	IQG1	4	psiblast	1nf1A_	195
YPL242C	IQG1	5	unassigned	na	81
YPL242C	IQG1	6	msa	na	246
YPL243W	SRP68	1	msa	na	96
YPL243W	SRP68	2	unassigned	na	131
YPL243W	SRP68	3	msa	na	372
YPL244C	HUT1	1	unassigned	na	73
YPL244C	HUT1	2	msa	na	266
YPL245W	YPL245W	1	msa	na	214
YPL245W	YPL245W	2	unassigned	na	65
YPL245W	YPL245W	3	msa	na	123
YPL245W	YPL245W	4	unassigned	na	52
YPL246C	RBD2	1	pfam	PF01694	262
YPL247C	YPL247C	1	psiblast	1gxrA_	122
YPL247C	YPL247C	2	psiblast	1gg2B_	401
YPL248C	GAL4	1	psiblast	1d66A_	65
YPL248C	GAL4	2	pfam	PF03902	60
YPL248C	GAL4	3	msa	na	366
YPL248C	GAL4	4	unassigned	na	106
YPL248C	GAL4	5	msa	na	284

YPL249C	GYP5	1	msa	na	412
YPL249C	GYP5	2	psiblast	1fkmA_	225
YPL249C	GYP5	3	psiblast	1i84S_	176
YPL249C	GYP5	4	unassigned	na	81
YPL249C-A	RPL36B	1	pfam	PF01158	100
YPL250C	ICY2	1	unassigned	na	136
YPL251W	YPL251W	1	unassigned	na	100
YPL252C	YAH1	1	psiblast	1cqxA_	51
YPL252C	YAH1	2	psiblast	1l6uA_	121
YPL253C	VIK1	1	psiblast	1i84S_	345
YPL253C	VIK1	2	psiblast	2tmaA_	61
YPL253C	VIK1	3	psiblast	2tmaA_	60
YPL253C	VIK1	4	psiblast	2tmaA_	98
YPL253C	VIK1	5	unassigned	na	83
YPL254W	HFI1	1	msa	na	322
YPL254W	HFI1	2	unassigned	na	166
YPL255W	BBP1	1	unassigned	na	232
YPL255W	BBP1	2	msa	na	153
YPL256C	CLN2	1	psiblast	1vin__	168
YPL256C	CLN2	2	psiblast	1vin__	148
YPL256C	CLN2	3	unassigned	na	229
YPL257W	YPL257W	1	unassigned	na	193
YPL258C	THI21	1	psiblast	1jxhA_	319
YPL258C	THI21	2	pfam	PF03070	232
YPL259C	APM1	1	psiblast	1gw5M_	143
YPL259C	APM1	2	psiblast	1gw5M_	332
YPL260W	YPL260W	1	msa	na	159
YPL260W	YPL260W	2	unassigned	na	104
YPL260W	YPL260W	3	msa	na	288
YPL261C	YPL261C	1	unassigned	na	102
YPL262W	FUM1	1	psiblast	1yfm__	326
YPL262W	FUM1	2	psiblast	1yfm__	102
YPL262W	FUM1	3	psiblast	1yfm__	60
YPL263C	KEL3	1	psiblast	1gof__	420
YPL263C	KEL3	2	msa	na	148
YPL263C	KEL3	3	unassigned	na	83
YPL264C	YPL264C	1	pfam	PF00892	174
YPL264C	YPL264C	2	pfam	PF00892	179
YPL265W	DIP5	1	unassigned	na	81
YPL265W	DIP5	2	pfam	PF00324	527
YPL266W	DIM1	1	psiblast	1yub__	215
YPL266W	DIM1	2	psiblast	1yub__	103
YPL267W	YPL267W	1	unassigned	na	209
YPL268W	PLC1	1	unassigned	na	86
YPL268W	PLC1	2	psiblast	1mai__	138
YPL268W	PLC1	3	psiblast	1djgA_	145
YPL268W	PLC1	4	psiblast	1djgA_	357
YPL268W	PLC1	5	psiblast	1djgA_	143

YPL269W	KAR9	1	unassigned	na	341
YPL269W	KAR9	2	msa	na	303
YPL270W	MDL2	1	unassigned	na	134
YPL270W	MDL2	2	psiblast	1jsqA_	321
YPL270W	MDL2	3	psiblast	1jsqA_	297
YPL270W	MDL2	4	unassigned	na	68
YPL271W	ATP15	1	psiblast	1e79I_	62
YPL272C	YPL272C	1	unassigned	na	517
YPL273W	SAM4	1	psiblast	1lt8A_	325
YPL274W	SAM3	1	unassigned	na	56
YPL274W	SAM3	2	pfam	PF00324	531
YPL275W	FDH2	1	psiblast	2nacA_	186
YPL275W	FDH2	2	psiblast	2nacA_	50
YPL276W	FDH2	1	psiblast	2nacA_	145
YPL277C	YPL277C	1	unassigned	na	487
YPL278C	YPL278C	1	unassigned	na	100
YPL279C	YPL279C	1	unassigned	na	171
YPL279C	YPL279C	2	pfam	PF02537	204
YPL280W	HSP32	1	psiblast	1n57A_	237
YPR001W	CIT3	1	psiblast	1cts__	379
YPR001W	CIT3	2	psiblast	1cts__	107
YPR002C-A	YPR002C-A	1	unassigned	na	65
YPR002W	PDH1	1	pfam	PF03972	516
YPR003C	YPR003C	1	unassigned	na	90
YPR003C	YPR003C	2	msa	na	105
YPR003C	YPR003C	3	pfam	PF00916	359
YPR003C	YPR003C	4	unassigned	na	59
YPR003C	YPR003C	5	psiblast	1auz__	141
YPR004C	YPR004C	1	psiblast	1efvA_	212
YPR004C	YPR004C	2	psiblast	1efvA_	132
YPR005C	HAL1	1	unassigned	na	294
YPR006C	ICL2	1	psiblast	1dquA_	460
YPR006C	ICL2	2	psiblast	1dquA_	115
YPR007C	REC8	1	unassigned	na	680
YPR008W	HAA1	1	psiblast	1co4A_	84
YPR008W	HAA1	2	unassigned	na	610
YPR009W	SUT2	1	unassigned	na	268
YPR010C	RPA135	1	msa	na	895
YPR010C	RPA135	2	unassigned	na	82
YPR010C	RPA135	3	msa	na	94
YPR010C	RPA135	4	msa	na	132
YPR011C	YPR011C	1	pfam	PF00153	117
YPR011C	YPR011C	2	pfam	PF00153	111
YPR011C	YPR011C	3	pfam	PF00153	98
YPR012W	YPR012W	1	unassigned	na	84
YPR013C	YPR013C	1	msa	na	191
YPR013C	YPR013C	2	psiblast	1ubdC_	126
YPR014C	YPR014C	1	unassigned	na	109

YPR015C	YPR015C	1	msa	na	132
YPR015C	YPR015C	2	psiblast	1ubdC_	115
YPR016C	TIF6	1	psiblast	1g62A_	245
YPR016W-A	YPR016W-A	1	unassigned	na	86
YPR017C	DSS4	1	unassigned	na	143
YPR018W	RLF2	1	psiblast	1i84S_	264
YPR018W	RLF2	2	msa	na	219
YPR018W	RLF2	3	unassigned	na	123
YPR019W	CDC54	1	msa	na	181
YPR019W	CDC54	2	psiblast	1ltlA_	125
YPR019W	CDC54	3	psiblast	1ltlA_	168
YPR019W	CDC54	4	psiblast	1ltlA_	52
YPR019W	CDC54	5	psiblast	1g8pA_	229
YPR019W	CDC54	6	psiblast	1g8pA_	87
YPR019W	CDC54	7	unassigned	na	91
YPR020W	ATP20	1	unassigned	na	115
YPR021C	AGC1	1	msa	na	150
YPR021C	AGC1	2	psiblast	1vrkA_	93
YPR021C	AGC1	3	psiblast	1vrkA_	100
YPR021C	AGC1	4	unassigned	na	131
YPR021C	AGC1	5	msa	na	370
YPR021C	AGC1	6	unassigned	na	58
YPR022C	YPR022C	1	psiblast	2adr__	95
YPR022C	YPR022C	2	msa	na	275
YPR022C	YPR022C	3	unassigned	na	101
YPR022C	YPR022C	4	msa	na	524
YPR022C	YPR022C	5	unassigned	na	138
YPR023C	EAF3	1	unassigned	na	94
YPR023C	EAF3	2	msa	na	307
YPR024W	YME1	1	msa	na	182
YPR024W	YME1	2	unassigned	na	72
YPR024W	YME1	3	psiblast	1lv7A_	193
YPR024W	YME1	4	psiblast	1lv7A_	105
YPR024W	YME1	5	msa	na	80
YPR024W	YME1	6	msa	na	115
YPR025C	CCL1	1	psiblast	1jkw__	259
YPR025C	CCL1	2	psiblast	1jkw__	134
YPR026W	ATH1	1	unassigned	na	103
YPR026W	ATH1	2	psiblast	1h54B_	336
YPR026W	ATH1	3	psiblast	1h54B_	479
YPR026W	ATH1	4	unassigned	na	293
YPR027C	YPR027C	1	unassigned	na	277
YPR028W	YOP1	1	pfam	PF03134	180
YPR029C	APL4	1	psiblast	1gw5A_	104
YPR029C	APL4	2	psiblast	1gw5A_	74
YPR029C	APL4	3	psiblast	1gw5A_	159
YPR029C	APL4	4	psiblast	1gw5A_	70
YPR029C	APL4	5	psiblast	1gw5A_	127

YPR029C	APL4	6	msa	na	154
YPR029C	APL4	7	psiblast	1iu1A_	144
YPR030W	CSR2	1	unassigned	na	268
YPR030W	CSR2	2	msa	na	281
YPR030W	CSR2	3	psiblast	1g4mA_	242
YPR030W	CSR2	4	unassigned	na	330
YPR031W	NTO1	1	msa	na	257
YPR031W	NTO1	2	psiblast	1f62A_	59
YPR031W	NTO1	3	msa	na	66
YPR031W	NTO1	4	msa	na	219
YPR031W	NTO1	5	psiblast	1jm4B_	147
YPR032W	SRO7	1	psiblast	1erjA_	338
YPR032W	SRO7	2	psiblast	1c5kA_	113
YPR032W	SRO7	3	msa	na	172
YPR032W	SRO7	4	msa	na	91
YPR032W	SRO7	5	msa	na	319
YPR033C	HTS1	1	psiblast	1kmmA_	421
YPR033C	HTS1	2	psiblast	1kmmA_	125
YPR034W	ARP7	1	psiblast	1yagA_	183
YPR034W	ARP7	2	psiblast	1yagA_	294
YPR035W	GLN1	1	psiblast	1f1hA_	106
YPR035W	GLN1	2	psiblast	1f1hA_	152
YPR035W	GLN1	3	psiblast	1f1hA_	112
YPR036W	VMA13	1	psiblast	1ho8A_	158
YPR036W	VMA13	2	psiblast	1ho8A_	81
YPR036W	VMA13	3	psiblast	1ho8A_	114
YPR036W	VMA13	4	psiblast	1ho8A_	125
YPR037C	ERV2	1	psiblast	1jr8A_	196
YPR038W	YPR038W	1	unassigned	na	119
YPR039W	YPR039W	1	unassigned	na	111
YPR040W	TIP41	1	unassigned	na	54
YPR040W	TIP41	2	msa	na	245
YPR040W	TIP41	3	unassigned	na	57
YPR041W	TIF5	1	psiblast	1k8bA_	95
YPR041W	TIF5	2	psiblast	1k81A_	38
YPR041W	TIF5	3	msa	na	148
YPR041W	TIF5	4	pfam	PF02020	124
YPR042C	PUF2	1	msa	na	279
YPR042C	PUF2	2	pfam	PF00076	175
YPR042C	PUF2	3	unassigned	na	57
YPR042C	PUF2	4	psiblast	1ib2A_	139
YPR042C	PUF2	5	psiblast	1ib2A_	101
YPR042C	PUF2	6	psiblast	1ib2A_	148
YPR042C	PUF2	7	unassigned	na	176
YPR044C	YPR044C	1	unassigned	na	117
YPR045C	YPR045C	1	unassigned	na	192
YPR045C	YPR045C	2	pfam	PF03399	278
YPR046W	MCM16	1	fold_recogniti	1bgd_	181

YPR047W	MSF1	1	psiblast	1b70A_	283
YPR047W	MSF1	2	pfam	PF01409	83
YPR047W	MSF1	3	pfam	PF03147	108
YPR048W	TAH18	1	psiblast	1amoA_	187
YPR048W	TAH18	2	psiblast	1amoA_	177
YPR048W	TAH18	3	psiblast	1amoA_	103
YPR048W	TAH18	4	psiblast	1amoA_	156
YPR049C	ATG11	1	psiblast	1i84S_	376
YPR049C	ATG11	2	unassigned	na	106
YPR049C	ATG11	3	psiblast	2tmaA_	71
YPR049C	ATG11	4	psiblast	2tmaA_	83
YPR049C	ATG11	5	psiblast	2tmaA_	62
YPR049C	ATG11	6	psiblast	2tmaA_	70
YPR049C	ATG11	7	psiblast	1jchA_	157
YPR049C	ATG11	8	msa	na	253
YPR050C	YPR050C	1	unassigned	na	137
YPR051W	MAK3	1	psiblast	1mk4A_	176
YPR052C	NHP6A	1	psiblast	1cg7A_	93
YPR053C	YPR053C	1	unassigned	na	151
YPR054W	SMK1	1	psiblast	1erk__	142
YPR054W	SMK1	2	psiblast	1erk__	246
YPR055W	SEC8	1	psiblast	1i84S_	320
YPR055W	SEC8	2	msa	na	227
YPR055W	SEC8	3	msa	na	130
YPR055W	SEC8	4	unassigned	na	67
YPR055W	SEC8	5	msa	na	269
YPR055W	SEC8	6	unassigned	na	52
YPR056W	TFB4	1	pfam	PF03850	338
YPR057W	BRR1	1	unassigned	na	341
YPR058W	YMC1	1	pfam	PF00153	115
YPR058W	YMC1	2	pfam	PF00153	96
YPR058W	YMC1	3	pfam	PF00153	96
YPR059C	YPR059C	1	unassigned	na	128
YPR060C	ARO7	1	psiblast	2csmA_	256
YPR061C	JID1	1	psiblast	1xbl__	198
YPR061C	JID1	2	unassigned	na	103
YPR062W	FCY1	1	psiblast	1uaqA_	158
YPR063C	YPR063C	1	unassigned	na	140
YPR064W	YPR064W	1	unassigned	na	139
YPR065W	ROX1	1	psiblast	1gt0D_	92
YPR065W	ROX1	2	msa	na	276
YPR066W	UBA3	1	psiblast	1ngvB_	171
YPR066W	UBA3	2	psiblast	1ngvB_	56
YPR066W	UBA3	3	psiblast	1ngvB_	72
YPR067W	ISA2	1	psiblast	1nwbA_	185
YPR068C	HOS1	1	unassigned	na	74
YPR068C	HOS1	2	psiblast	1c3pA_	164
YPR068C	HOS1	3	psiblast	1c3pA_	232

YPR069C	SPE3	1	psiblast	1inlA_	65
YPR069C	SPE3	2	psiblast	1inlA_	228
YPR070W	MED1	1	unassigned	na	566
YPR071W	YPR071W	1	msa	na	211
YPR072W	NOT5	1	psiblast	1i84S_	260
YPR072W	NOT5	2	msa	na	300
YPR073C	LTP1	1	psiblast	1d1pA_	161
YPR074C	TKL1	1	psiblast	1gpuA_	291
YPR074C	TKL1	2	psiblast	1gpuA_	49
YPR074C	TKL1	3	psiblast	1gpuA_	242
YPR074C	TKL1	4	psiblast	1gpuA_	98
YPR074W-A	YPR074W-A	1	unassigned	na	56
YPR075C	OPY2	1	msa	na	247
YPR075C	OPY2	2	unassigned	na	113
YPR076W	YPR076W	1	unassigned	na	124
YPR077C	YPR077C	1	unassigned	na	123
YPR078C	YPR078C	1	unassigned	na	372
YPR079W	MRL1	1	unassigned	na	59
YPR079W	MRL1	2	psiblast	1c39A_	170
YPR079W	MRL1	3	psiblast	1e6fA_	152
YPR081C	GRS2	1	psiblast	1atiA_	427
YPR081C	GRS2	2	psiblast	1atiA_	122
YPR081C	GRS2	3	psiblast	1nj8A_	69
YPR082C	DIB1	1	psiblast	1qgvA_	143
YPR083W	MDM36	1	unassigned	na	378
YPR083W	MDM36	2	msa	na	201
YPR084W	YPR084W	1	unassigned	na	456
YPR085C	YPR085C	1	fold_recogniti	1erjA	448
YPR086W	SUA7	1	psiblast	1dl6A_	72
YPR086W	SUA7	2	psiblast	1c9bA_	160
YPR086W	SUA7	3	psiblast	1c9bA_	113
YPR087W	VPS69	1	unassigned	na	106
YPR088C	SRP54	1	psiblast	1j8mF_	89
YPR088C	SRP54	2	psiblast	1j8mF_	230
YPR088C	SRP54	3	psiblast	1mfqC_	89
YPR088C	SRP54	4	psiblast	1m2vB_	133
YPR089W	YPR089W	1	unassigned	na	155
YPR090W	6281	1	msa	na	334
YPR090W	6281	2	unassigned	na	402
YPR091C	YPR091C	1	unassigned	na	109
YPR091C	YPR091C	2	pfam	PF00169	158
YPR091C	YPR091C	3	msa	na	324
YPR091C	YPR091C	4	unassigned	na	179
YPR092W	YPR092W	1	unassigned	na	101
YPR093C	YPR093C	1	psiblast	1jm7A_	111
YPR093C	YPR093C	2	unassigned	na	59
YPR093C	YPR093C	3	msa	na	140
YPR094W	RDS3	1	pfam	PF03660	107

YPR095C	SYT1	1	msa	na	408
YPR095C	SYT1	2	psiblast	1bc9__	81
YPR095C	SYT1	3	psiblast	1bc9__	199
YPR095C	SYT1	4	unassigned	na	538
YPR096C	YPR096C	1	unassigned	na	100
YPR097W	YPR097W	1	unassigned	na	232
YPR097W	YPR097W	2	pfam	PF00787	272
YPR097W	YPR097W	3	msa	na	413
YPR097W	YPR097W	4	unassigned	na	156
YPR098C	YPR098C	1	unassigned	na	108
YPR099C	YPR099C	1	unassigned	na	118
YPR100W	MRPL51	1	unassigned	na	140
YPR101W	SNT309	1	unassigned	na	175
YPR102C	RPL11A	1	psiblast	1ffkD_	174
YPR103W	PRE2	1	unassigned	na	65
YPR103W	PRE2	2	psiblast	1g0uK_	222
YPR104C	FHL1	1	msa	na	235
YPR104C	FHL1	2	fold_recogniti	1gxcA	187
YPR104C	FHL1	3	psiblast	1d5vA_	159
YPR104C	FHL1	4	unassigned	na	56
YPR104C	FHL1	5	fold_recogniti	1l8mA	76
YPR104C	FHL1	6	fold_recogniti	1l8mA	78
YPR104C	FHL1	7	fold_recogniti	1l8mA	70
YPR104C	FHL1	8	fold_recogniti	1l8mA	75
YPR105C	COG4	1	psiblast	1i84S_	228
YPR105C	COG4	2	unassigned	na	330
YPR105C	COG4	3	msa	na	303
YPR106W	ISR1	1	unassigned	na	76
YPR106W	ISR1	2	psiblast	1f3mC_	156
YPR106W	ISR1	3	psiblast	1f3mC_	211
YPR107C	YTH1	1	psiblast	1m9oA_	120
YPR107C	YTH1	2	msa	na	88
YPR108W	RPN7	1	msa	na	90
YPR108W	RPN7	2	psiblast	1na0A_	181
YPR108W	RPN7	3	msa	na	158
YPR109W	YPR109W	1	unassigned	na	294
YPR110C	RPC40	1	psiblast	1i3qC_	270
YPR110C	RPC40	2	psiblast	1i3qC_	65
YPR111W	DBF20	1	psiblast	1ctpE_	284
YPR111W	DBF20	2	psiblast	1ctpE_	140
YPR112C	MRD1	1	psiblast	1fj7A_	113
YPR112C	MRD1	2	msa	na	203
YPR112C	MRD1	3	psiblast	1cvjA_	107
YPR112C	MRD1	4	psiblast	1cvjA_	105
YPR112C	MRD1	5	psiblast	1fjeB_	99
YPR112C	MRD1	6	psiblast	1l3kA_	127
YPR112C	MRD1	7	psiblast	1l3kA_	133
YPR113W	PIS1	1	pfam	PF01066	220

YPR114W	YPR114W	1	unassigned	na	77
YPR114W	YPR114W	2	msa	na	238
YPR115W	YPR115W	1	unassigned	na	475
YPR115W	YPR115W	2	pfam	PF00169	272
YPR115W	YPR115W	3	unassigned	na	336
YPR116W	YPR116W	1	unassigned	na	277
YPR117W	YPR117W	1	unassigned	na	739
YPR117W	YPR117W	2	msa	na	736
YPR117W	YPR117W	3	unassigned	na	225
YPR117W	YPR117W	4	msa	na	235
YPR117W	YPR117W	5	msa	na	502
YPR117W	YPR117W	6	unassigned	na	52
YPR118W	YPR118W	1	pfam	PF01008	411
YPR119W	CLB2	1	msa	na	223
YPR119W	CLB2	2	psiblast	1vin__	147
YPR119W	CLB2	3	psiblast	1vin__	121
YPR120C	CLB5	1	msa	na	137
YPR120C	CLB5	2	psiblast	1vin__	173
YPR120C	CLB5	3	psiblast	1vin__	125
YPR121W	THI22	1	psiblast	1jxhA_	320
YPR121W	THI22	2	msa	na	252
YPR122W	AXL1	1	psiblast	1hr6B_	273
YPR122W	AXL1	2	psiblast	1hr6B_	198
YPR122W	AXL1	3	unassigned	na	97
YPR122W	AXL1	4	msa	na	443
YPR122W	AXL1	5	msa	na	110
YPR122W	AXL1	6	unassigned	na	87
YPR123C	YPR123C	1	unassigned	na	144
YPR124W	CTR1	1	msa	na	185
YPR124W	CTR1	2	unassigned	na	221
YPR125W	YPR125W	1	msa	na	354
YPR125W	YPR125W	2	unassigned	na	100
YPR126C	YPR126C	1	unassigned	na	102
YPR127W	YPR127W	1	psiblast	1exbA_	270
YPR127W	YPR127W	2	psiblast	1exbA_	75
YPR128C	ANT1	1	pfam	PF00153	112
YPR128C	ANT1	2	pfam	PF00153	103
YPR128C	ANT1	3	pfam	PF00153	113
YPR129W	SCD6	1	msa	na	189
YPR129W	SCD6	2	msa	na	160
YPR130C	YPR130C	1	unassigned	na	135
YPR131C	NAT3	1	psiblast	1i1dA_	251
YPR133C	IWS1	1	msa	na	227
YPR133C	IWS1	2	unassigned	na	53
YPR133C	IWS1	3	msa	na	130
YPR133W-A	TOM5	1	unassigned	na	50
YPR134W	MSS18	1	unassigned	na	268
YPR135W	CTF4	1	psiblast	1erjA_	348

YPR135W	CTF4	2	psiblast	1l0qA_	120
YPR135W	CTF4	3	unassigned	na	70
YPR135W	CTF4	4	msa	na	256
YPR135W	CTF4	5	unassigned	na	133
YPR136C	YPR136C	1	unassigned	na	170
YPR137W	RRP9	1	unassigned	na	59
YPR137W	RRP9	2	psiblast	1nexB_	81
YPR137W	RRP9	3	psiblast	1nexB_	294
YPR137W	RRP9	4	psiblast	1erjA_	68
YPR137W	RRP9	5	unassigned	na	71
YPR138C	MEP3	1	pfam	PF00909	489
YPR139C	VPS66	1	msa	na	205
YPR139C	VPS66	2	unassigned	na	95
YPR140W	TAZ1	1	psiblast	1k30A_	329
YPR140W	TAZ1	2	unassigned	na	52
YPR141C	KAR3	1	psiblast	1i84S_	371
YPR141C	KAR3	2	psiblast	1f9tA_	358
YPR142C	YPR142C	1	unassigned	na	187
YPR143W	YPR143W	1	fold_recogniti	1c1gA	59
YPR143W	YPR143W	2	fold_recogniti	1c1gA	191
YPR144C	NOC4	1	fold_recogniti	1b3uA_	425
YPR144C	NOC4	2	unassigned	na	127
YPR145W	ASN1	1	psiblast	1ct9A_	192
YPR145W	ASN1	2	psiblast	1ct9A_	380
YPR146C	YPR146C	1	unassigned	na	109
YPR147C	YPR147C	1	msa	na	118
YPR147C	YPR147C	2	msa	na	186
YPR148C	YPR148C	1	msa	na	294
YPR148C	YPR148C	2	unassigned	na	141
YPR149W	NCE102	1	unassigned	na	173
YPR150W	YPR150W	1	unassigned	na	173
YPR151C	SUE1	1	msa	na	206
YPR152C	YPR152C	1	psiblast	1o6wA_	96
YPR152C	YPR152C	2	unassigned	na	78
YPR152C	YPR152C	3	psiblast	1h40A_	138
YPR152C	YPR152C	4	unassigned	na	153
YPR153W	YPR153W	1	unassigned	na	144
YPR154W	PIN3	1	psiblast	1griA_	122
YPR154W	PIN3	2	msa	na	93
YPR155C	NCA2	1	unassigned	na	212
YPR155C	NCA2	2	msa	na	404
YPR156C	TPO3	1	msa	na	163
YPR156C	TPO3	2	pfam	PF00083	459
YPR157W	YPR157W	1	unassigned	na	100
YPR157W	YPR157W	2	msa	na	367
YPR158W	YPR158W	1	msa	na	252
YPR159W	KRE6	1	unassigned	na	206
YPR159W	KRE6	2	pfam	PF03935	71

YPR159W	KRE6	3	psiblast	1dypA_	274
YPR159W	KRE6	4	psiblast	1macA_	169
YPR160W	GPH1	1	psiblast	1a8i__	556
YPR160W	GPH1	2	psiblast	1a8i__	346
YPR161C	SGV1	1	psiblast	1aq1__	151
YPR161C	SGV1	2	psiblast	1aq1__	153
YPR161C	SGV1	3	psiblast	1aq1__	51
YPR161C	SGV1	4	psiblast	1aq1__	73
YPR161C	SGV1	5	unassigned	na	229
YPR162C	ORC4	1	psiblast	1lv7A_	278
YPR162C	ORC4	2	psiblast	1lv7A_	93
YPR162C	ORC4	3	msa	na	158
YPR163C	TIF3	1	psiblast	2up1A_	92
YPR163C	TIF3	2	psiblast	1cvjA_	125
YPR163C	TIF3	3	psiblast	1cvjA_	119
YPR163C	TIF3	4	unassigned	na	100
YPR164W	MMS1	1	unassigned	na	1407
YPR165W	RHO1	1	psiblast	1lb1B_	209
YPR166C	MRP2	1	psiblast	1p87N_	115
YPR167C	MET16	1	psiblast	1sur__	261
YPR168W	NUT2	1	unassigned	na	157
YPR169W	JIP5	1	psiblast	1k8kC_	380
YPR169W	JIP5	2	psiblast	1gg2B_	134
YPR170C	YPR170C	1	unassigned	na	111
YPR170W-A	YPR170W-A	1	unassigned	na	61
YPR171W	BSP1	1	unassigned	na	97
YPR171W	BSP1	2	msa	na	367
YPR171W	BSP1	3	msa	na	112
YPR172W	YPR172W	1	unassigned	na	200
YPR173C	VPS4	1	msa	na	96
YPR173C	VPS4	2	psiblast	1e32A_	202
YPR173C	VPS4	3	psiblast	1e32A_	75
YPR173C	VPS4	4	unassigned	na	64
YPR174C	YPR174C	1	unassigned	na	83
YPR174C	YPR174C	2	msa	na	138
YPR175W	DPB2	1	unassigned	na	498
YPR175W	DPB2	2	msa	na	194
YPR176C	BET2	1	psiblast	1dceB_	178
YPR176C	BET2	2	psiblast	1dceB_	147
YPR177C	YPR177C	1	unassigned	na	123
YPR178W	PRP4	1	psiblast	1l0qA_	126
YPR178W	PRP4	2	psiblast	1gg2B_	339
YPR179C	HDA3	1	psiblast	2mysA_	241
YPR179C	HDA3	2	unassigned	na	121
YPR179C	HDA3	3	psiblast	1c1gA_	64
YPR179C	HDA3	4	psiblast	1c1gA_	229
YPR180W	AOS1	1	psiblast	1ngvA_	170
YPR180W	AOS1	2	psiblast	1ngvA_	177

YPR181C	SEC23	1	psiblast	1m2oA_	646
YPR181C	SEC23	2	psiblast	1m2oA_	122
YPR182W	SMX3	1	psiblast	1i81A_	86
YPR183W	DPM1	1	psiblast	1h7lA_	125
YPR183W	DPM1	2	msa	na	142
YPR184W	GDB1	1	psiblast	1j0hA_	110
YPR184W	GDB1	2	psiblast	1j0hA_	334
YPR184W	GDB1	3	unassigned	na	95
YPR184W	GDB1	4	msa	na	273
YPR184W	GDB1	5	unassigned	na	115
YPR184W	GDB1	6	msa	na	609
YPR185W	ATG13	1	unassigned	na	90
YPR185W	ATG13	2	msa	na	154
YPR185W	ATG13	3	msa	na	356
YPR185W	ATG13	4	unassigned	na	138
YPR186C	PZF1	1	psiblast	1ubdC_	188
YPR186C	PZF1	2	msa	na	162
YPR186C	PZF1	3	unassigned	na	79
YPR187W	RPO26	1	psiblast	1i3qF_	155
YPR188C	MLC2	1	psiblast	1iq5A_	88
YPR188C	MLC2	2	psiblast	1iq5A_	75
YPR189W	SKI3	1	msa	na	179
YPR189W	SKI3	2	msa	na	207
YPR189W	SKI3	3	psiblast	1fchA_	346
YPR189W	SKI3	4	psiblast	1na0A_	117
YPR189W	SKI3	5	unassigned	na	86
YPR189W	SKI3	6	psiblast	1d8dA_	214
YPR189W	SKI3	7	psiblast	1elrA_	72
YPR189W	SKI3	8	msa	na	211
YPR190C	RPC82	1	msa	na	144
YPR190C	RPC82	2	unassigned	na	128
YPR190C	RPC82	3	msa	na	382
YPR191W	QCR2	1	psiblast	1ezvB_	193
YPR191W	QCR2	2	psiblast	1ezvB_	175
YPR192W	AQY1	1	psiblast	1fqyA_	165
YPR192W	AQY1	2	psiblast	1fqyA_	140
YPR193C	HPA2	1	psiblast	1qsmA_	156
YPR195C	YPR195C	1	unassigned	na	109
YPR196W	YPR196W	1	psiblast	1d66A_	56
YPR196W	YPR196W	2	msa	na	234
YPR196W	YPR196W	3	unassigned	na	180
YPR197C	YPR197C	1	unassigned	na	187
YPR198W	SGE1	1	pfam	PF00083	428
YPR198W	SGE1	2	msa	na	115
YPR199C	ARR1	1	psiblast	1gd2E_	136
YPR199C	ARR1	2	unassigned	na	158
YPR200C	ARR2	1	psiblast	1cwtA_	130
YPR201W	ARR3	1	unassigned	na	66

YPR201W	ARR3	2	pfam	PF01758	211
YPR201W	ARR3	3	msa	na	127
YPR202W	YPR202W	1	msa	na	238
YPR203W	YPR203W	1	unassigned	na	102
YPR204W	YPR204W	1	psiblast	1fuuA_	392
YPR204W	YPR204W	2	msa	na	346
YPR204W	YPR204W	3	unassigned	na	294
