# Supplementary material for: Superfamily Assignments for the Yeast Proteome through Integration of Structure Prediction with the Gene Ontology
Source: PLoS Biol. 2007 Mar 20;5(4):e76. doi: 10.1371/journal.pbio.0050076 (PMC1828141; doi:10.1371/journal.pbio.0050076)
Supplement: Supplementary file 2 [file pbio.0050076.st002.pdf]

ac	gene_name	domain_nr	MCM score	sccs description
YMR225C	MRPL44	1	0.8500161	SH2 domain
YCL056C	YCL056C	1	0.901412	t-snare proteins
YCR020C-A	MAK31	1	0.8052541	L30e-like
YBR253W	SRB6	1	0.8380764	t-snare proteins
YLR204W	QRI5	1	0.9001616	Succinate dehydrogenase/fumarate reductase flavoprotein C-terminal domain
YFL010W-A	AUA1	1	0.8709216	HSC20 (HSCB), C-terminal oligomerisation domain
YAR030C	YAR030C	1	0.8047973	Cytochromes
YAR053W	YAR053W	1	0.9471299	Translation initiation factor IF3, C-terminal domain
YKL224C	YKL224C	1	0.8935874	Acyl-CoA dehydrogenase C-terminal domain-like
YKL123W	YKL123W	1	0.8383844	Fertilization protein
YKL137W	YKL137W	1	0.9478462	Hemerythrin
YBL077W	YBL077W	1	0.8216679	SGNH hydrolase
YIL176C	YIL176C	1	0.9538127	Glutathione S-transferase (GST), C-terminal domain
YJL067W	YJL067W	1	0.8415872	C-terminal effector domain of the bipartite response regulators
YBR301W	DAN3	1	0.8022961	Acyl-CoA dehydrogenase C-terminal domain-like
YJL199C	MBB1	1	0.8364534	Quinohemoprotein amine dehydrogenase A chain, domain 3
YIL141W	YIL141W	1	0.8364395	t-snare proteins
YIL012W	YIL012W	1	0.8565117	Neurotransmitter-gated ion-channel transmembrane pore
YFL020C	PAU5	1	0.8256845	alpha-catenin/vinculin
YML089C	YML089C	1	0.8014963	Neurotransmitter-gated ion-channel transmembrane pore
YAL064W	YAL064W	1	0.8301918	Group V grass pollen allergen
YAL045C	YAL045C	1	0.9116643	Hemerythrin
YEL008W	YEL008W	1	0.8104104	Cyclin-like
YHL046C	YHL046C	1	0.82386	Acyl-CoA dehydrogenase C-terminal domain-like
YLR325C	RPL38	1	0.8578486	RNA 3'-terminal phosphate cyclase, RPTC, insert domain
YDR134C	YDR134C	1	0.8450396	Alpha-lytic protease prodomain
YMR007W	YMR007W	1	0.8019607	t-snare proteins
YMR325W	YMR325W	1	0.919294	Ferritin-like
YGL239C	YGL239C	1	0.8759231	Ribosomal protein S20
YDR016C	DAD1	1	0.9345842	Neurotransmitter-gated ion-channel transmembrane pore
YDL170W	UGA3	3	0.887551	Cyclin-like
YPR020W	ATP20	1	0.9237924	Globin-like
YNL086W	YNL086W	1	0.813412	F1F0 ATP synthase subunit C
YDR511W	ACN9	1	0.873999	Ribosomal protein S5 domain 2-like

YHR039C-A	VMA10	1	0.9407527	t-snare proteins
YJL188C	BUD19	1	0.9145571	Cyclin-like
YJR067C	YAE1	1	0.8588077	Apolipophorin-III
YJR071W	YJR071W	1	0.8922787	Enzyme IIa from lactose specific PTS, IIa-lac
YJR079W	YJR079W	1	0.8073631	Spectrin repeat
YDL158C	YDL158C	1	0.9395859	Enzyme IIa from lactose specific PTS, IIa-lac
YMR321C	YMR321C	1	0.8286087	PreATP-grasp domain
YHL046W-A	YHL046W-A	1	0.8477858	Mechanosensitive channel protein MscS (YggB), transmembrane region
YHL006W-A	YHL006W-A	1	0.801192	Globin-like
YAL068C	YAL068C	1	0.8397204	Ferritin-like
YDR542W	YDR542W	1	0.830162	Ferritin-like
YLR112W	YLR112W	1	0.8534201	Globin-like
YLL025W	YLL025W	1	0.8464268	Acyl-CoA dehydrogenase C-terminal domain-like
YGL121C	GPG1	1	0.934683	TPR-like
YGR011W	YGR011W	1	0.9032881	FAT domain of focal adhesion kinase
YGR050C	YGR050C	1	0.9255073	Cyclin-like
YGR151C	YGR151C	1	0.8087466	Sigma2 domain of RNA polymerase sigma factors
YGR182C	YGR182C	1	0.8673217	Ribosomal protein S20
YGR294W	YGR294W	1	0.857793	Acyl-CoA dehydrogenase C-terminal domain-like
YDR286C	YDR286C	1	0.8651624	Thioredoxin-like
YLR012C	YLR012C	1	0.8107106	HSC20 (HSCB), C-terminal oligomerisation domain
YFL034C-A	RPL22B	1	0.8548544	Acylphosphatase-like
YPL144W	YPL144W	1	0.8182168	Flavoproteins
YOL118C	YOL118C	1	0.9642878	Subdomain of clathrin and coatomer appendage domain
YOL134C	YOL134C	1	0.9434323	Voltage-gated potassium channels
YOR072W	YOR072W	1	0.9163313	Hemerythrin
YOR200W	YOR200W	1	0.8008774	ARM repeat
YOR300W	YOR300W	1	0.8921667	Enzyme IIa from lactose specific PTS, IIa-lac
YOR366W	YOR366W	1	0.93792	Cytochromes
YLR202C	YLR202C	1	0.831457	DNA polymerase III clamp loader subunits, C-terminal domain
YLR255C	YLR255C	1	0.8582043	FKBP12-rapamycin-binding domain of FKBP-rapamycin-associated protein (FRAP)
YLR317W	YLR317W	1	0.8079231	Pili subunits
YPL035C	YPL035C	1	0.8522011	Nucleotidyltransferase substrate binding subunit/domain
YPL102C	YPL102C	1	0.9616348	Cytochromes
YPR096C	YPR096C	1	0.8722385	tRNA-intron endonuclease catalytic domain-like

YPR146C	YPR146C	1	0.8451808	Enzyme IIa from lactose specific PTS, IIa-lac
YLR235C	YLR235C	1	0.9248184	F1F0 ATP synthase subunit C
YDL045W-A	MRP10	1	0.9161435	Domain of the SRP/SRP receptor G-proteins
YEL020W-A	TIM9	1	0.8811445	DEATH domain
YDR322C-A	TIM11	1	0.8580783	t-snare proteins
YER048W-A	YER048W-A	1	0.8590212	Saposin
YHR072W-A	NOP10	1	0.8286719	
YDR079C-A	YDR079C-A	1	0.9157771	CheY-binding domain of CheA
YDR379C-A	YDR379C-A	1	0.8581252	Ribosomal protein S20
YOL086W-A	YOL086W-A	1	0.9272228	Chemotaxis receptor methyltransferase CheR, N-terminal domain
YBL071W-A	KTI11	1	0.8784145	N-utilization substance G protein NusG, insert domain
YDL185C-A	YDL185C-A	1	0.8902787	Anticodon-binding domain of a subclass of class I aminoacyl-tRNA synthetases
YNR001W-A	YNR001W-A	1	0.9639813	ACT-like
YML019W	OST6	1	0.8310058	Saposin
YDL240W	LRG1	3	0.959994	Succinate dehydrogenase/fumarate reductase flavoprotein C-terminal domain
YDL240W	LRG1	4	0.9507163	t-snare proteins
YOL051W	GAL11	3	0.8937189	Enzyme IIa from lactose specific PTS, IIa-lac
YHR070W	TRM5	4	0.9036459	Aminoacid dehydrogenase-like, N-terminal domain
YML095C	RAD10	1	0.8689633	
YDL059C	RAD59	2	0.8245387	dsRNA-binding domain-like
YBR079C	RPG1	4	0.8724772	HMA, heavy metal-associated domain
YHR077C	NMD2	2	0.8714656	C-terminal domain of RNA polymerase alpha subunit
YER015W	FAA2	1	0.8546869	S13-like H2TH domain
YDR449C	UTP6	4	0.8282438	DNA polymerase beta-like, second domain
YBR286W	APE3	1	0.8161709	
YLR425W	TUS1	2	0.8616455	Origin of replication-binding domain, RBD-like
YBR058C	UBP14	4	0.892164	TPR-like
YNL053W	MSG5	3	0.8511909	Domain of the SRP/SRP receptor G-proteins
YDR379W	RGA2	5	0.8983544	FAT domain of focal adhesion kinase
YLR151C	PCD1	2	0.9046461	F-box domain
YBR276C	PPS1	2	0.8808284	TPR-like
YGR277C	YGR277C	1	0.8888104	Anticodon-binding domain of Class II aaRS
YBR229C	ROT2	1	0.9326923	ClpS-like
YHR117W	TOM71	5	0.8741559	TPR-like
YGL035C	MIG1	3	0.8536502	HMA, heavy metal-associated domain

YGL206C	CHC1	9	0.8434813	Ribosomal protein L22
YJR066W	TOR1	8	0.9068531	Cytochrome c oxidase subunit E
YDR335W	MSN5	3	0.8965996	Methionine synthase domain
YDR335W	MSN5	4	0.8620366	BAG domain
YLR291C	GCD7	1	0.9395466	TPR-like
YAL013W	DEP1	2	0.9551276	DBL homology domain (DH-domain)
YAR062W	YAR062W	2	0.8131105	Carboxypeptidase regulatory domain
YBL023C	MCM2	3	0.8167756	Cytochrome c oxidase subunit E
YBL042C	FUI1	1	0.8473574	Anticodon-binding domain of a subclass of class I aminoacyl-tRNA synthetases
YBL105C	PKC1	1	0.8795691	Small-conductance potassium channel
YBR102C	EXO84	2	0.8184288	Spectrin repeat
YBR111C	YSA1	1	0.8906018	Amine oxidase N-terminal region
YBR192W	RIM2	3	0.9565296	HSC20 (HSCB), C-terminal oligomerisation domain
YBR208C	DUR1,2	8	0.8095037	Aminoacid dehydrogenase-like, N-terminal domain
YBR245C	ISW1	1	0.8205161	Nucleotidyltransferase substrate binding subunit/domain
YBR291C	CTP1	2	0.8480396	Group V grass pollen allergen
YBR295W	PCA1	3	0.8012953	GlnB-like
YCL031C	RRP7	2	0.9027887	Ferritin-like
YCR037C	PHO87	2	0.8191776	Saposin
YDL037C	BSC1	1	0.8849834	SH3-domain
YDL070W	BDF2	5	0.8073258	Bacterial immunoglobulin/albumin-binding domains
YDL093W	PMT5	2	0.8772479	Complement control module/SCR domain
YDL095W	PMT1	2	0.8680828	Complement control module/SCR domain
YDL119C	YDL119C	1	0.8560971	
YDR036C	EHD3	4	0.8289086	p8-MTCP1
YDR068W	DOS2	2	0.8609879	6-phosphogluconate dehydrogenase C-terminal domain-like
YDR081C	PDC2	5	0.8877515	Enzyme IIa from lactose specific PTS, IIa-lac
YDR087C	RRP1	1	0.8426341	ARM repeat
YDR122W	KIN1	4	0.8840306	alpha-2-Macroglobulin receptor associated protein (RAP) domain 1
YDR124W	YDR124W	1	0.9636502	Probable ACP-binding domain of malonyl-CoA ACP transacylase
YDR265W	PEX10	1	0.8876452	Glutathione S-transferase (GST), C-terminal domain
YDR283C	GCN2	2	0.8443807	Ribosomal protein L20
YDR289C	RTT103	1	0.8708721	Methionine synthase domain
YGL076C	RPL7A	1	0.9196403	Enzyme IIa from lactose specific PTS, IIa-lac
YGL159W	YGL159W	1	0.8276263	Winged helix DNA-binding domain

YGL171W	ROK1	4	0.9497807	Conserved domain common to transcription factors TFIIS, elongin A, CRSP70
YIR010W	DSN1	1	0.8573101	ACT-like
YHR012W	VPS29	2	0.9412826	PUA domain-like
YBL022C	PIM1	7	0.8650376	Porphobilinogen deaminase (hydroxymethylbilane synthase), C-terminal domain
YBL047C	EDE1	8	0.8618831	F1F0 ATP synthase subunit C
YBR203W	COS111	2	0.8432728	Hemerythrin
YBR263W	SHM1	1	0.8718294	Enzyme IIa from lactose specific PTS, IIa-lac
YCL008C	STP22	2	0.9554952	Anticodon-binding domain of a subclass of class I aminoacyl-tRNA synthetases
YCL030C	HIS4	2	0.9437147	C-terminal domain of ProRS
YCL030C	HIS4	4	0.9070026	Group V grass pollen allergen
YCL048W	YCL048W	3	0.8081712	DNA helicase RuvA subunit, C-terminal domain
YCL067C	HMLALPHA2	1	0.9941406	t-snare proteins
YCR059C	YIH1	2	0.8646185	Cytidine deaminase-like
YDL028C	MPS1	3	0.8592224	TrpR-like
YDL038C	YDL038C	2	0.8345791	SirA-like
YDL040C	NAT1	4	0.9452862	TPR-like
YDL077C	VAM6	2	0.8045683	Polynucleotide phosphorylase/guanosine pentaphosphate synthase (PNPase/GPSI)
YDL077C	VAM6	4	0.8527022	Hemerythrin
YDL104C	QRI7	1	0.8864191	RNA-binding domain, RBD
YDL113C	ATG20	3	0.820895	Hemerythrin
YDL234C	GYP7	5	0.8355689	HMG-box
YDR006C	SOK1	4	0.9284228	DNA polymerase III clamp loader subunits, C-terminal domain
YDR049W	YDR049W	5	0.8814846	HSC20 (HSCB), C-terminal oligomerisation domain
YDR090C	YDR090C	2	0.8091055	LEM domain
YDR125C	ECM18	1	0.9094613	
YDR213W	UPC2	4	0.931642	TPR-like
YDR258C	HSP78	1	0.8516331	Winged helix DNA-binding domain
YDR458C	YDR458C	3	0.8091979	Bcl-2 inhibitors of programmed cell death
YDR465C	RMT2	2	0.8727244	Anticodon-binding domain of Class II aaRS
YDR498C	SEC20	2	0.9726961	tRNA-binding arm
YEL061C	CIN8	1	0.9554122	Rudiment single hybrid motif
YER021W	RPN3	1	0.8234546	TPR-like
YER048C	CAJ1	3	0.8664393	Cyclin-like
YER060W	FCY21	1	0.9270249	FF domain
YER128W	YER128W	1	0.8502465	Group V grass pollen allergen

YER128W	YER128W	2	0.8256895	HSC20 (HSCB), C-terminal oligomerisation domain
YER143W	DDI1	2	0.8490885	DEATH domain
YER184C	YER184C	2	0.8970864	HSC20 (HSCB), C-terminal oligomerisation domain
YFL001W	DEG1	4	0.9216408	Mechanosensitive channel protein MscS (YggB), C-terminal domain
YFL042C	YFL042C	2	0.8025051	Histone H3 K4-specific methyltransferase SET7/9 N-terminal domain
YGL141W	HUL5	2	0.8718905	Outer surface protein C (OspC)
YGL227W	VID30	5	0.8864474	Zinc beta-ribbon
YGR047C	TFC4	5	0.84476	6-phosphogluconate dehydrogenase C-terminal domain-like
YGR270W	YTA7	9	0.8972044	Group V grass pollen allergen
YHL024W	RIM4	1	0.9155148	Hemolysin expression modulating protein HHA
YHR066W	SSF1	2	0.8195461	dsRNA-binding domain-like
YHR146W	CRP1	1	0.9692786	Invasin/intimin cell-adhesion fragments
YHR203C	RPS4B	2	0.8632227	Quinohemoprotein amine dehydrogenase A chain, domain 3
YIL011W	TIR3	1	0.9246745	Succinate dehydrogenase/fumarate reductase flavoprotein C-terminal domain
YIL016W	SNL1	2	0.9592668	HSC20 (HSCB), C-terminal oligomerisation domain
YIL097W	FYV10	1	0.9787787	Spectrin repeat
YIL110W	YIL110W	1	0.8111381	Phospholipase A2, PLA2
YIL144W	TID3	4	0.8512339	DNA polymerase III clamp loader subunits, C-terminal domain
YJL002C	OST1	1	0.8663242	Neurotransmitter-gated ion-channel transmembrane pore
YJL065C	DLS1	2	0.8216577	
YJL149W	YJL149W	1	0.8571997	FliG
YJL180C	ATP12	2	0.8179357	Voltage-gated potassium channels
YJL198W	PHO90	2	0.8738329	Fatty acid responsive transcription factor FadR, C-terminal domain
YJR003C	YJR003C	3	0.9144265	
YJR041C	URB2	6	0.8246184	TPR-like
YKL045W	PRI2	1	0.9457604	Gametocyte protein Pfg27
YLR129W	DIP2	3	0.8883723	Scorpion toxin-like
YLR129W	DIP2	4	0.8893714	Zinc beta-ribbon
YLR129W	DIP2	5	0.8669213	Signal recognition particle alu RNA binding heterodimer, SRP9/14
YLR222C	UTP13	2	0.9432172	Zinc beta-ribbon
YOR262W	YOR262W	2	0.9188342	Phosphoglucomutase, first 3 domains
YKR001C	VPS1	3	0.9149118	Neurotransmitter-gated ion-channel transmembrane pore
YKR041W	YKR041W	2	0.9754845	t-snare proteins
YKR095W	MLP1	3	0.9751936	t-snare proteins
YKR095W	MLP1	7	0.8635437	DEATH domain

YLL001W	DNM1	4	0.9619956	tRNA-binding arm
YLL026W	HSP104	1	0.9712926	Cyclin-like
YLR137W	YLR137W	2	0.8160349	Macro domain-like
YLR176C	RFX1	5	0.9828888	HSC20 (HSCB), C-terminal oligomerisation domain
YLR228C	ECM22	3	0.955797	Anticodon-binding domain of a subclass of class I aminoacyl-tRNA synthetases
YLR357W	RSC2	3	0.9842254	Bromodomain
YLR403W	SFP1	1	0.9418915	Pterin-4a-carbinolamine dehydratase (PCD)/dimerization cofactor of HNF1 (DCoH)
YML034W	SRC1	4	0.8004288	Spectrin repeat
YML068W	ITT1	2	0.840586	Anticodon-binding domain of a subclass of class I aminoacyl-tRNA synthetases
YML102W	CAC2	1	0.9367181	Myosin S1 fragment, N-terminal domain
YML104C	MDM1	3	0.9060428	Ribosomal protein S20
YMR023C	MSS1	1	0.9174477	GlnB-like
YMR027W	YMR027W	1	0.8593547	Glutathione S-transferase (GST), C-terminal domain
YMR027W	YMR027W	2	0.9319933	BAG domain
YMR069W	NAT4	1	0.8348828	Globin-like
YMR203W	TOM40	2	0.8856672	Immunoglobulin-binding domains
YNL014W	HEF3	2	0.9416687	Anticodon-binding domain of a subclass of class I aminoacyl-tRNA synthetases
YNL041C	COG6	3	0.8756553	Ribosomal protein S20
YNL051W	COG5	1	0.9071597	STAT
YHR107C	CDC12	2	0.9269455	Smac/diablo
YNL022C	YNL022C	1	0.970272	DNA polymerase III clamp loader subunits, C-terminal domain
YNL083W	YNL083W	3	0.8633557	
YNL201C	PSY2	2	0.8685044	Anaphylotoxins (complement system)
YNL201C	PSY2	5	0.8420155	DNA polymerase III clamp loader subunits, C-terminal domain
YNR060W	FRE4	6	0.8327289	SirA-like
YNR070W	YNR070W	2	0.812975	
YOL015W	YOL015W	1	0.841856	Enzyme IIa from lactose specific PTS, IIa-lac
YOL089C	HAL9	3	0.8295168	Nucleoside phosphorylase/phosphoribosyltransferase N-terminal domain
YOR001W	RRP6	3	0.949215	Cyclin-like
YDR146C	SWI5	1	0.857895	Glucose permease domain IIB
YDR174W	HMO1	1	0.9322783	Tubulin chaperone cofactor A
YNL234W	YNL234W	3	0.8162249	Anaphylotoxins (complement system)
YOL147C	PEX11	2	0.8874784	t-snare proteins
YOR206W	NOC2	2	0.8297047	ARM repeat
YOR230W	WTM1	1	0.8586761	Carboxypeptidase regulatory domain

YOR295W	UAF30	1	0.8548579	Nucleoside phosphorylase/phosphoribosyltransferase N-terminal domain
YDR152W	GIR2	2	0.9008706	Ferritin-like
YDR198C	YDR198C	2	0.938088	alpha-ketoacid dehydrogenase kinase, N-terminal domain
YDR200C	VPS64	2	0.9214186	Immunoglobulin-binding domains
YDR532C	YDR532C	1	0.820245	Glutathione S-transferase (GST), C-terminal domain
YEL007W	YEL007W	4	0.8623422	Interleukin 8-like chemokines
YER060W-A	FCY22	1	0.8291569	FF domain
YER112W	LSM4	2	0.8189611	RuvA domain 2-like
YER140W	YER140W	2	0.8157977	F1F0 ATP synthase subunit C
YER144C	UBP5	1	0.9770273	alpha-catenin/vinculin
YER144C	UBP5	2	0.823573	P-loop containing nucleoside triphosphate hydrolases
YER144C	UBP5	6	0.8728158	Rudiment single hybrid motif
YER144C	UBP5	7	0.9088517	Zinc beta-ribbon
YER164W	CHD1	11	0.9315958	Enzyme IIa from lactose specific PTS, IIa-lac
YFR004W	RPN11	3	0.9291744	HR1 repeat
YFR021W	ATG18	1	0.912833	Invasin/intimin cell-adhesion fragments
YFR023W	PES4	1	0.8061702	PGBD-like
YFR023W	PES4	6	0.908654	Aminoacid dehydrogenase-like, N-terminal domain
YFR052W	RPN12	1	0.8649605	alpha-catenin/vinculin
YGL062W	PYC1	7	0.8465603	Translation proteins SH3-like domain
YGL086W	MAD1	6	0.836577	Ferritin-like
YGL246C	RAI1	2	0.8012532	RNA-binding domain, RBD
YGL256W	ADH4	1	0.8441637	F1F0 ATP synthase subunit C
YGR062C	COX18	1	0.8693236	Transducin (alpha subunit), insertion domain
YGR103W	NOP7	2	0.8094338	SAM/Pointed domain
YGR119C	NUP57	3	0.9165855	Ferritin-like
YGR199W	PMT6	2	0.8909677	Complement control module/SCR domain
YGR200C	ELP2	4	0.8971821	Immunoglobulin
YGR208W	SER2	1	0.8469606	EF-G C-terminal domain-like
YGR210C	YGR210C	3	0.8570886	CBS-domain
YHR069C	RRP4	3	0.830134	Polynucleotide phosphorylase/guanosine pentaphosphate synthase (PNPase/GPSI)
YHR188C	GPI16	1	0.8309189	Prokaryotic type KH domain (KH-domain type II)
YIL073C	SPO22	6	0.8765613	Prokaryotic type KH domain (KH-domain type II)
YIL147C	SLN1	5	0.8058374	PTS system, Lactose/Cellobiose specific IIB subunit (Pfam 02302)
YIL155C	GUT2	3	0.82938	Bifunctional inhibitor/lipid-transfer protein/seed storage 2S albumin

YIL177C	YIL177C	3	0.8449964	Globin-like
YIR004W	DJP1	4	0.9784976	Group V grass pollen allergen
YJL044C	GYP6	4	0.8043367	Anticodon-binding domain of a subclass of class I aminoacyl-tRNA synthetases
YJL090C	DPB11	4	0.8415452	ACT-like
YJL110C	GZF3	4	0.8066248	Nucleoside phosphorylase/phosphoribosyltransferase N-terminal domain
YJL140W	RPB4	1	0.8927559	t-snare proteins
YJL225C	YJL225C	3	0.9467272	Fatty acid responsive transcription factor FadR, C-terminal domain
YJL225C	YJL225C	6	0.8292633	SAM/Pointed domain
YJR073C	OPI3	1	0.812691	Enzyme IIa from lactose specific PTS, IIa-lac
YJR144W	MGM101	1	0.8235488	SirA-like
YKL020C	SPT23	3	0.8904563	Fibronectin type III
YKL035W	UGP1	1	0.8513054	Calponin-homology domain, CH-domain
YKL040C	NFU1	2	0.9788899	Multidrug efflux transporter AcrB pore domain; PN1, PN2, PC1 and PC2 subdomai
YKL052C	ASK1	1	0.8216234	lambda integrase-like, N-terminal domain
YKL187C	YKL187C	3	0.8463651	Domain of the SRP/SRP receptor G-proteins
YKL203C	TOR2	6	0.8981829	ENTH/VHS domain
YKR086W	PRP16	3	0.8683447	
YKR098C	UBP11	1	0.8394411	PABC (PABP) domain
YLL005C	SPO75	2	0.8710116	t-snare proteins
YLR067C	PET309	2	0.8742474	TPR-like
YLR181C	VTA1	1	0.9551035	Winged helix DNA-binding domain
YLR191W	PEX13	2	0.9102088	GAT-like domain
YLR292C	SEC72	1	0.9499311	Globin-like
YLR292C	SEC72	2	0.8399399	C-terminal domain of DFF45/ICAD (DFF-C domain)
YLR318W	EST2	4	0.9551976	Group V grass pollen allergen
YLR330W	CHS5	1	0.9422031	Ribosomal protein L6
YLR396C	VPS33	4	0.8320249	tRNA-intron endonuclease catalytic domain-like
YLR398C	SKI2	5	0.8008504	FAD-linked reductases, C-terminal domain
YLR431C	ATG23	2	0.9391253	t-snare proteins
YML010W	SPT5	7	0.8960837	Fibronectin type I module
YML010W	SPT5	9	0.9061005	Zinc beta-ribbon
YML020W	YML020W	2	0.9352144	Elongation factor Ts (EF-Ts), dimerisation domain
YML035C	AMD1	2	0.9265023	Zinc beta-ribbon
YML133C	YML133C	2	0.9009496	DNA polymerase III clamp loader subunits, C-terminal domain
YMR054W	STV1	1	0.8137335	EF-hand

YNL084C	END3	4	0.9238798	tRNA-binding arm
YNR011C	PRP2	3	0.889332	ARM repeat
YNR063W	YNR063W	3	0.8160029	Group V grass pollen allergen
YOL136C	PFK27	2	0.8813473	
YOL142W	RRP40	2	0.8447015	Acylphosphatase-like
YOR010C	TIR2	1	0.8342172	Anticodon-binding domain of a subclass of class I aminoacyl-tRNA synthetases
YOR021C	YOR021C	1	0.8802431	SirA-like
YOR062C	YOR062C	1	0.8195407	Neurotransmitter-gated ion-channel transmembrane pore
YOR093C	YOR093C	7	0.8372342	Annexin
YOR140W	SFL1	3	0.8613835	tRNA-binding arm
YOR144C	ELG1	5	0.8400926	Bifunctional inhibitor/lipid-transfer protein/seed storage 2S albumin
YOR156C	NFI1	3	0.8420273	Immunoglobulin-binding domains
YOR261C	RPN8	1	0.8553643	YjgF-like
YBR136W	MEC1	4	0.8339585	Ferritin-like
YBR140C	IRA1	4	0.829292	Translational machinery components
YER132C	PMD1	1	0.8584122	Enolase N-terminal domain-like
YER172C	BRR2	2	0.8313507	Saposin
YGL131C	SNT2	6	0.8274532	Ribosomal protein L20
YGL195W	GCN1	5	0.9847852	BAG domain
YGR184C	UBR1	1	0.8616297	
YHL049C	YHL049C	2	0.8038659	Winged helix DNA-binding domain
YIL115C	NUP159	5	0.8471193	Annexin
YIL130W	YIL130W	2	0.8627901	lambda integrase-like, N-terminal domain
YIL134W	FLX1	3	0.8941778	DEATH domain
YIL137C	YIL137C	4	0.8213756	Probable ACP-binding domain of malonyl-CoA ACP transacylase
YIL137C	YIL137C	6	0.8991087	alpha-ketoacid dehydrogenase kinase, N-terminal domain
YIL149C	MLP2	6	0.9232631	DNA polymerase beta, N-terminal domain-like
YJR095W	SFC1	3	0.8478431	Spectrin repeat
YJR111C	YJR111C	2	0.9241612	Cyclin-like
YKL205W	LOS1	2	0.9174189	Polynucleotide phosphorylase/guanosine pentaphosphate synthase (PNPase/GPSI)
YKL215C	YKL215C	2	0.9184647	D-aminopeptidase, middle and C-terminal domains
YKR028W	SAP190	5	0.8680031	Eukaryotic DNA topoisomerase I, dispensable insert domain
YLL021W	SPA2	5	0.954572	Computational models partly based on NMR data
YLL040C	VPS13	11	0.8120934	Signal recognition particle alu RNA binding heterodimer, SRP9/14
YLL040C	VPS13	13	0.8411587	PH domain-like

YLR024C	UBR2	1	0.8717696	Saposin
YML059C	YML059C	6	0.8390749	Anti-sigma factor antagonist SpoIIaa
YML072C	TCB3	2	0.8157936	Avidin/streptavidin
YMR014W	BUD22	1	0.8391174	Bromodomain
YMR028W	TAP42	1	0.9426104	Enzyme IIa from lactose specific PTS, IIa-lac
YMR031C	YMR031C	2	0.807123	Anticodon-binding domain of a subclass of class I aminoacyl-tRNA synthetases
YMR162C	DNF3	3	0.823674	Winged helix DNA-binding domain
YMR164C	MSS11	4	0.8419077	
YMR207C	HFA1	11	0.8553813	Replisome organizer (g39p helicase loader/inhibitor protein)
YMR229C	RRP5	3	0.816214	SH3-domain
YMR239C	RNT1	2	0.8230939	Annexin
YMR288W	HSH155	3	0.9643049	Domain of the SRP/SRP receptor G-proteins
YMR299C	DYN3	1	0.8899595	CheY-like
YMR306W	FKS3	1	0.8312362	Cyclin-like
YMR309C	NIP1	3	0.823603	Winged helix DNA-binding domain
YMR317W	YMR317W	4	0.8620721	Microbial ribonucleases
YNL297C	MON2	2	0.854508	Anticodon-binding domain of a subclass of class I aminoacyl-tRNA synthetases
YNR016C	ACC1	4	0.9586209	beta-Barrel protease inhibitors
YNR016C	ACC1	7	0.8665298	Domain of the SRP/SRP receptor G-proteins
YOR336W	KRE5	4	0.8852698	C-terminal domain of arginine repressor
YOR356W	YOR356W	3	0.8130475	Pili subunits
YOR389W	YOR389W	2	0.8697974	t-snare proteins
YPL020C	ULP1	3	0.9048112	Neurotransmitter-gated ion-channel transmembrane pore
YPL072W	UBP16	2	0.805102	SAM/Pointed domain
YPL078C	ATP4	1	0.900074	BAG domain
YPL082C	MOT1	9	0.8229364	CheY-like
YPL084W	BRO1	2	0.96721	Fertilization protein
YPL128C	TBF1	1	0.8522214	Globin-like
YPL134C	ODC1	2	0.9385137	HSC20 (HSCB), C-terminal oligomerisation domain
YPL154C	PEP4	1	0.8873521	Mechanosensitive channel protein MscS (YggB), transmembrane region
YPL174C	NIP100	2	0.8009328	lambda integrase-like, N-terminal domain
YPL198W	RPL7B	1	0.9406932	GreA transcript cleavage protein, N-terminal domain
YPL199C	YPL199C	1	0.9708753	F1F0 ATP synthase subunit C
YPL226W	NEW1	2	0.9652017	ENTH/VHS domain
YPL243W	SRP68	2	0.9431132	alpha-catenin/vinculin

YPL248C	GAL4	4	0.8745111	F1F0 ATP synthase subunit A
YPR011C	YPR011C	3	0.8032248	Cyclin-like
YPR019W	CDC54	7	0.8054829	SirA-like
YPR022C	YPR022C	5	0.9008338	ENTH/VHS domain
YPR032W	SRO7	4	0.8768658	Immunoglobulin
YPR041W	TIF5	4	0.8892932	Globin-like
YPR049C	ATG11	2	0.9612743	t-snare proteins
YPR055W	SEC8	3	0.9181923	FAT domain of focal adhesion kinase
YPR058W	YMC1	1	0.8303617	Succinate dehydrogenase/fumarate reductase flavoprotein C-terminal domain
YPR061C	JID1	2	0.8238815	DNA polymerase III clamp loader subunits, C-terminal domain
YPR093C	YPR093C	3	0.8922738	Cytochromes
YOR124C	UBP2	2	0.8933772	ENTH/VHS domain
YPR128C	ANT1	3	0.8448585	F1F0 ATP synthase subunit C
YPR135W	CTF4	3	0.8027644	Translation proteins SH3-like domain
YPR135W	CTF4	5	0.8819868	Acyl-CoA dehydrogenase C-terminal domain-like
YPR139C	VPS66	2	0.8720207	CheY-like
YPR173C	VPS4	1	0.9750281	t-snare proteins
YPR190C	RPC82	1	0.8423688	ARID-like
YLR263W	RED1	5	0.9655801	DNA polymerase III clamp loader subunits, C-terminal domain
YIL072W	HOP1	2	0.8276	HMA, heavy metal-associated domain
