# Supplementary material for: Superfamily Assignments for the Yeast Proteome through Integration of Structure Prediction with the Gene Ontology
Source: PLoS Biol. 2007 Mar 20;5(4):e76. doi: 10.1371/journal.pbio.0050076 (PMC1828141; doi:10.1371/journal.pbio.0050076)
Supplement: Supplementary file 3 [file pbio.0050076.st003.pdf]

ac	gene name	domain nr	GI score	sccs description	go description
YDL181W	INH1	1	0.8184115	ARM repeat	ATP synthesis coupled proton transport
YIL148W	RPL40A	2	0.9634657	UBA-like	cytosolic large ribosomal subunit (sensu Eukaryota)
YBR254C	TRS20	1	0.9781324	SNARE-like	ER to Golgi vesicle-mediated transport
YBL001C	ECM15	1	0.8705166	MTH1187/YkoF-like	cell wall organization and biogenesis
YKL160W	ELF1	1	0.9289918	Winged helix DNA-binding domain	RNA elongation from RNA polymerase II promoter
YKL084W	YKL084W	1	0.9634324	Cytochrome c	mitochondrial intermembrane space
YKR068C	BET3	1	0.9033663	P-loop containing nucleoside triphosphate hydrolas	ER to Golgi vesicle-mediated transport
YGR020C	VMA7	1	0.8287817	P-loop containing nucleoside triphosphate hydrolas	hydrogen-transporting ATPase activity, rotational me
YBR268W	MRPL37	1	0.8584643	Ribosomal protein L6	structural constituent of ribosome
YGL189C	RPS26A	1	0.8013301	P-loop containing nucleoside triphosphate hydrolas	cytosolic small ribosomal subunit (sensu Eukaryota)
YBR188C	NTC20	1	0.8882758	L30e-like	nuclear mRNA splicing, via spliceosome
YER074W	RPS24A	1	0.8862791	Immunoglobulin	cytosolic small ribosomal subunit (sensu Eukaryota)
YDR185C	YDR185C	1	0.8269767	P-loop containing nucleoside triphosphate hydrolas	mitochondrion
YNR022C	MRPL50	1	0.9042176	S13-like H2TH domain	structural constituent of ribosome
YLL042C	ATG10	1	0.9787164	P-loop containing nucleoside triphosphate hydrolas	protein targeting to vacuole
YML058W-A	HUG1	1	0.8286195	Winged helix DNA-binding domain	cell cycle arrest
YCR071C	IMG2	1	0.8551944	P-loop containing nucleoside triphosphate hydrolas	structural constituent of ribosome
YCL063W	VAC17	1	0.8715908	t-snare proteins	vacuolar membrane
YIL075C	RPN2	7	0.9152665	P-loop containing nucleoside triphosphate hydrolas	ubiquitin-dependent protein catabolism
YHR040W	BCD1	1	0.8365779	Homeodomain-like	nucleus
YFR027W	ECO1	2	0.8666423	Tudor/PWWP/MBT	nuclear chromatin
YOR174W	MED4	1	0.9775938	Homeodomain-like	transcription from RNA polymerase II promoter
YDR147W	EKI1	1	0.8694301	PH domain-like	cytoplasm
YMR216C	SKY1	1	0.9539021	P-loop containing nucleoside triphosphate hydrolas	positive regulation of protein import into nucleus
YER169W	RPH1	4	0.968531	Homeodomain-like	specific transcriptional repressor activity
YDL131W	LYS21	2	0.837121	Cyclin-like	nucleus
YML121W	GTR1	2	0.8687055	RNA-binding domain, RBD	telomere maintenance
YBR017C	KAP104	3	0.9901366	ARM repeat	nuclear localization sequence binding
YIR023W	DAL81	1	0.8631204	Winged helix DNA-binding domain	specific RNA polymerase II transcription factor activi
YJL088W	ARG3	1	0.8337213	Immunoglobulin	ornithine metabolism
YGL035C	MIG1	5	0.9332434	Winged helix DNA-binding domain	specific RNA polymerase II transcription factor activi
YOR025W	HST3	3	0.956238	Homeodomain-like	chromatin silencing at telomere
YLR032W	RAD5	5	0.9202435	Homeodomain-like	nuclear chromatin

YGR024C	THG1	2	0.9835023	Nucleic acid-binding proteins	tRNA modification
YAL041W	CDC24	6	0.8260112	Immunoglobulin	pheromone-dependent signal transduction during co
YBL093C	ROX3	1	0.8300491	Homeodomain-like	transcription from RNA polymerase II promoter
YBR025C	YBR025C	3	0.8464367	Thioredoxin-like	cytoplasm
YBR150C	TBS1	1	0.8533888	Homeodomain-like	nucleus
YDL048C	STP4	2	0.8319085	Cytochromes	mitochondrion
YDL070W	BDF2	2	0.8843345	Bromodomain	nucleus
YDL070W	BDF2	3	0.8877531	Homeodomain-like	nucleus
YDL201W	TRM8	1	0.8732086	Homeodomain-like	nucleus
YDR027C	VPS54	6	0.9626964	P-loop containing nucleoside triphosphate hydrolas	Golgi to vacuole transport
YDR285W	ZIP1	3	0.9385488	Homeodomain-like	chromatin binding
YDR356W	SPC110	6	0.9609295	EF-hand	central plaque of spindle pole body
YHR006W	STP2	5	0.941218	Winged helix DNA-binding domain	specific RNA polymerase II transcription factor activi
YGR280C	PXR1	1	0.8974607	Nucleic acid-binding proteins	nucleolus
YHR161C	YAP1801	3	0.9209755	EF-hand	clathrin binding
YBL037W	APL3	6	0.9442186	ENTH/VHS domain	vesicle-mediated transport
YBR273C	UBX7	4	0.8416931	EF-hand	nuclear envelope
YCL030C	HIS4	3	0.9504301	Immunoglobulin	histidinol dehydrogenase activity
YCR008W	SAT4	2	0.8518975	Skp1 dimerisation domain-like	G1/S transition of mitotic cell cycle
YCR044C	PER1	1	0.9695922	Winged helix DNA-binding domain	manganese ion homeostasis
YJR160C	MPH3	1	0.889758	Winged helix DNA-binding domain	carbohydrate transport
YDR043C	NRG1	1	0.8698169	Immunoglobulin	invasive growth (sensu Saccharomyces)
YDR045C	RPC11	1	0.9187443	Zinc beta-ribbon	DNA-directed RNA polymerase III complex
YDR236C	FMN1	2	0.9747125	Riboflavin kinase-like	riboflavin kinase activity
YDR311W	TFB1	3	0.808548	ARID-like	general RNA polymerase II transcription factor activi
YDR359C	VID21	5	0.9657795	Histone-fold	histone acetyltransferase complex
YDR429C	TIF35	1	0.9160146	Translation proteins SH3-like domain	translation initiation factor activity
YDR460W	TFB3	3	0.8214381	C-terminal UvrC-binding domain of UvrB	nucleotide-excision repair
YDR518W	EUG1	3	0.9787021	Thioredoxin-like	protein disulfide isomerase activity
YDR520C	YDR520C	5	0.856605	Winged helix DNA-binding domain	nucleus
YER093C	TSC11	8	0.9718512	4-helical cytokines	regulation of cell growth
YFL047W	RGD2	2	0.8073621	CAD & PB1 domains	bud neck
YGL156W	AMS1	4	0.8215096	Immunoglobulin	alpha-mannosidase activity
YGR071C	YGR071C	2	0.8505617	Ubiquitin-like	nucleus

YGR218W	CRM1	2	0.8710138	ARM repeat	protein carrier activity
YGR250C	YGR250C	1	0.9459291	RNA-binding domain, RBD	RNA binding
YHL009C	YAP3	3	0.9263121	Winged helix DNA-binding domain	regulation of transcription from RNA polymerase II p
YHL021C	FMP12	1	0.8163017	NAD(P)-binding Rossmann-fold domains	mitochondrion
YHR061C	GIC1	1	0.8497072	EF-hand	bud tip
YHR108W	GGA2	3	0.9322657	t-snare proteins	Golgi to vacuole transport
YHR154W	RTT107	3	0.8686939	EF-hand	double-strand break repair
YIL126W	STH1	4	0.9014696	Winged helix DNA-binding domain	DNA helicase activity
YJR119C	YJR119C	4	0.9318595	Homeodomain-like	nucleus
YJR147W	HMS2	3	0.8396565	Thioredoxin-like	pseudohyphal growth
YKL043W	PHD1	3	0.8579841	Homeodomain-like	specific RNA polymerase II transcription factor activi
YKL045W	PRI2	2	0.9495231	Winged helix DNA-binding domain	DNA replication initiation
YLR007W	NSE1	2	0.9326322	Nucleic acid-binding proteins	DNA repair
YKR078W	YKR078W	2	0.8464829	GAT-like domain	protein transporter activity
YLL054C	YLL054C	5	0.8019782	Winged helix DNA-binding domain	transcriptional activator activity
YLR016C	YLR016C	1	0.8848402	RNA-binding domain, RBD	mRNA export from nucleus
YLR127C	APC2	1	0.9671614	Winged helix DNA-binding domain	mitotic spindle elongation
YLR127C	APC2	2	0.9326083	Winged helix DNA-binding domain	mitotic spindle elongation
YLR143W	YLR143W	2	0.8916268	P-loop containing nucleoside triphosphate hydrolas	cytoplasm
YLR143W	YLR143W	5	0.9085045	P-loop containing nucleoside triphosphate hydrolas	cytoplasm
YLR220W	CCC1	1	0.9817049	Winged helix DNA-binding domain	manganese ion homeostasis
YLR386W	VAC14	1	0.864634	ARM repeat	vacuolar membrane (sensu Fungi)
YLR399C	BDF1	2	0.937793	Bromodomain	chromatin remodeling
YLR452C	SST2	2	0.8001403	Ankyrin repeat	GTPase activator activity
YLR455W	YLR455W	4	0.9662133	Homeodomain-like	nucleus
YML007W	YAP1	2	0.9155976	Thioredoxin-like	response to arsenic
YML052W	SUR7	2	0.8371186	Immunoglobulin	lipid raft
YML104C	MDM1	5	0.8820834	EF-hand	mitochondrion inheritance
YML127W	RSC9	1	0.9592669	Homeodomain-like	chromatin binding
YMR176W	ECM5	10	0.9517149	PreATP-grasp domain	cell wall organization and biogenesis
YMR190C	SGS1	6	0.830691	Immunoglobulin	meiotic chromosome segregation
YMR193W	MRPL24	2	0.9467004	Nucleic acid-binding proteins	structural constituent of ribosome
YMR219W	ESC1	1	0.9769498	Homeodomain-like	chromatin silencing at telomere
YMR219W	ESC1	4	0.9621029	Homeodomain-like	chromatin silencing at telomere

YNL023C	FAP1	6	0.9725824	Winged helix DNA-binding domain	transcription factor activity
YNL047C	LIT1	1	0.8867635	PH domain-like	phosphoinositide binding
YDR334W	SWR1	5	0.9302427	P-loop containing nucleoside triphosphate hydrolas	SWR1 complex
YNL022C	YNL022C	2	0.911681	Winged helix DNA-binding domain	nucleus
YNL102W	POL1	6	0.9559371	Winged helix DNA-binding domain	DNA replication initiation
YNL283C	WSC2	2	0.8625901	E set domains	Rho protein signal transduction
YOL105C	WSC3	4	0.9364641	Winged helix DNA-binding domain	response to heat
YOL113W	SKM1	3	0.8616128	Cyclin-like	protein serine/threonine kinase activity
YOR090C	PTC5	1	0.8538654	Immunoglobulin	protein phosphatase type 2C activity
YOR147W	MDM32	3	0.9002483	Bromodomain	mitochondrion organization and biogenesis
YCL024W	KCC4	5	0.9779102	Profilin (actin-binding protein)	axial bud site selection
YDR143C	SAN1	2	0.8149581	POZ domain	ubiquitin-protein ligase activity
YDR166C	SEC5	1	0.8983689	P-loop containing nucleoside triphosphate hydrolas	bipolar bud site selection
YDR488C	PAC11	1	0.9720205	P-loop containing nucleoside triphosphate hydrolas	microtubule motor activity
YDR497C	ITR1	1	0.9592453	Immunoglobulin	plasma membrane
YEL005C	VAB2	3	0.8334301	Fibronectin type III	protein binding
YEL077C	YEL077C	3	0.8969756	Winged helix DNA-binding domain	helicase activity
YER169W	RPH1	1	0.9768461	Homeodomain-like	specific transcriptional repressor activity
YGL236C	MTO1	5	0.8410343	P-loop containing nucleoside triphosphate hydrolas	protein biosynthesis
YGR170W	PSD2	2	0.9546257	ARM repeat	vacuolar membrane (sensu Fungi)
YGR202C	PCT1	3	0.8817312	TPR-like	Golgi apparatus
YGR229C	SMI1	1	0.8927604	Homeodomain-like	telomere maintenance
YHR073W	OSH3	2	0.8636877	Fibronectin type III	steroid biosynthesis
YIL017C	VID28	6	0.8510541	Winged helix DNA-binding domain	nucleus
YIL036W	CST6	2	0.9412392	Homeodomain-like	specific RNA polymerase II transcription factor activi
YJL187C	SWE1	4	0.9251892	Cyclin-like	regulation of cyclin dependent protein kinase activity
YKL040C	NFU1	1	0.8920596	LuxS/MPP-like metallohydrolase	mitochondrial matrix
YKL203C	TOR2	5	0.9193685	Cyclin-like	G1 phase of mitotic cell cycle
YKL203C	TOR2	7	0.9208379	Cyclin-like	G1 phase of mitotic cell cycle
YKR098C	UBP11	6	0.807372	Immunoglobulin	ubiquitin-specific protease activity
YLL067C	YLL067C	1	0.8021117	Winged helix DNA-binding domain	helicase activity
YLR086W	SMC4	1	0.8115097	P-loop containing nucleoside triphosphate hydrolas	mitotic chromosome condensation
YLR097C	HRT3	1	0.9871193	Winged helix DNA-binding domain	nuclear ubiquitin ligase complex
YLR219W	MSC3	4	0.8977331	dsRNA-binding domain-like	meiotic recombination

YLR239C	LIP2	3	0.8203151	Winged helix DNA-binding domain	ligase activity
YLR418C	CDC73	1	0.8287418	NAD(P)-binding Rossmann-fold domains	histone methylation
YML013W	SEL1	1	0.8490795	Ubiquitin-like	mitochondrial outer membrane
YML095C	RAD10	2	0.9479021	P-loop containing nucleoside triphosphate hydrolas	mitotic recombination
YNL339C	YRF1-6	4	0.9162954	DNA helicase RuvA subunit, C-terminal domain	DNA helicase activity
YOL004W	SIN3	4	0.8806806	Homeodomain-like	chromatin silencing at telomere
YBR023C	CHS3	3	0.9158667	CAD & PB1 domains	incipient bud site
YDR464W	SPP41	3	0.9072147	Histone-fold	negative regulation of transcription from RNA polyme
YER172C	BRR2	11	0.9867798	Sm-like ribonucleoproteins	pre-mRNA splicing factor activity
YHR002W	LEU5	1	0.9608735	Cytochrome c	mitochondrial inner membrane
YHR165C	PRP8	6	0.8857398	Thioredoxin-like	pre-mRNA splicing factor activity
YIL131C	FKH1	2	0.8194697	Immunoglobulin	chromatin silencing at silent mating-type cassette
YJR059W	PTK2	8	0.9196492	Homeodomain-like	G1/S transition of mitotic cell cycle
YJR138W	IML1	1	0.9577941	PX domain	vacuolar membrane (sensu Fungi)
YKR028W	SAP190	1	0.9216831	Cyclin-like	G1/S transition of mitotic cell cycle
YLL040C	VPS13	9	0.8950427	Immunoglobulin	endosome
YLR187W	SKG3	4	0.9641249	EF-hand	bud
YML069W	POB3	3	0.8540438	Winged helix DNA-binding domain	transcription initiation from RNA polymerase II prom
YML072C	TCB3	4	0.8142791	Profilin (actin-binding protein)	bud
YML072C	TCB3	10	0.8056627	Profilin (actin-binding protein)	bud
YMR036C	MIH1	1	0.9430463	Cyclin-like	regulation of cyclin dependent protein kinase activity
YMR036C	MIH1	2	0.9473278	Cyclin-like	regulation of cyclin dependent protein kinase activity
YMR282C	AEP2	1	0.8369749	P-loop containing nucleoside triphosphate hydrolas	protein biosynthesis
YMR301C	ATM1	1	0.8996612	HMA, heavy metal-associated domain	ATPase activity, coupled to transmembrane moveme
YOR326W	MYO2	10	0.9446453	EF-hand	microfilament motor activity
YOR335C	ALA1	3	0.848941	Cytochrome c	mitochondrion
YOR337W	TEA1	2	0.8663328	lambda repressor-like DNA-binding domains	transcription
YPL008W	CHL1	1	0.8439332	Winged helix DNA-binding domain	DNA helicase activity
YPL115C	BEM3	4	0.8855558	PH domain-like	Rho GTPase activator activity
YPL128C	TBF1	4	0.9084612	Homeodomain-like	nuclear chromosome, telomeric region
YPL133C	RDS2	2	0.9482051	Globin-like	response to xenobiotic stimulus
YPL138C	SPP1	3	0.8363453	Homeodomain-like	chromatin silencing at telomere
YPL172C	COX10	2	0.8494145	Cytochrome c	mitochondrial inner membrane
YPL181W	CTI6	1	0.8559488	Winged helix DNA-binding domain	positive regulation of transcription

YPL245W	YPL245W	2	0.9650208	Winged helix DNA-binding domain	nucleus
YPL245W	YPL245W	4	0.9412066	Homeodomain-like	nucleus
YPR011C	YPR011C	1	0.8395214	a domain/subunit of cytochrome bc1 complex (Ubi	mitochondrial inner membrane
YOR113W	AZF1	4	0.8218997	Homeodomain-like	transcriptional activator activity
YPL209C	IPL1	1	0.8446282	Winged helix DNA-binding domain	chromosome segregation
YPR137W	RRP9	5	0.8585231	RNA-binding domain, RBD	small nuclear ribonucleoprotein complex
YPR147C	YPR147C	1	0.8247942	P-loop containing nucleoside triphosphate hydrolas	cytoplasm
YOR373W	NUD1	4	0.919052	EF-hand	spindle pole body
