# Supplementary material for: Superfamily Assignments for the Yeast Proteome through Integration of Structure Prediction with the Gene Ontology
Source: PLoS Biol. 2007 Mar 20;5(4):e76. doi: 10.1371/journal.pbio.0050076 (PMC1828141; doi:10.1371/journal.pbio.0050076)
Supplement: Supplementary file 4 [file pbio.0050076.st004.pdf]

pdb	native sccs	length	convergence	lowest rms in	lowest rms in	MCM score	MCM sccs	score
1dlwa	a.1.1.1	116	4.7	3.87	4.32	0.99890784	a.1.1.1	5
1kr7a	a.1.1.4	110	3.1	3.51	4.61	0.99442452	a.1.1.2	3
1nekb	a.1.2.1	132	9.54	7.02	9.58	0.54485293	a.74.1.1	1
1qlab	a.1.2.1	133	9.61	8.49	8.56	0.59667794	a.1.2.1	5
1hd6a	a.10.1.1	37	2.68	3.1	4.45	0.34815738	a.10.1.1	5
1erp_	a.10.1.1	38	1.8	3.92	7.58	0.30010057	a.140.2.1	1
1f0ya	a.100.1.3	99	5.79	5.37	8.8	0.77579808	a.100.1.3	5
1dlja	a.100.1.4	98	3.96	6.64	9.7	0.37713609	a.100.1.4	5
1CCD	a.101.1.1	77	3.11	5.32	7.62	0.94292041	a.24.13.1	1
1utg_	a.101.1.1	70	2.27	4.28	6.18	0.90859478	a.101.1.1	5
5eau_	a.102.4.1	200	9.85	10.81	12.2	0.78142281	a.102.4.1	5
1ntca	a.105.1.1	91	3.99	11.57	12.52	0.96153607	a.4.9.1	1
d1etxa_	a.105.1.1	89	3.54	15.99	18.56	0.8516648	a.24.3.2	1
1dd3a	a.108.1.1	57	2.63	3.72	6.75	0.71712582	a.108.1.1	5
1iiea	a.109.1.1	75	4.25	10.24	14.38	0.56643423	a.46.2.1	1
1hb6a	a.11.1.1	86	4.37	3.88	5.15	0.94506086	a.11.1.1	5
1hbka	a.11.1.1	89	4.62	5.2	8.12	0.86514221	a.24.13.1	1
1gg3a	a.11.2.1	106	5.17	6.69	12.7	0.61999128	a.121.1.1	1
1h4ra	a.11.2.1	111	6.67	7.05	10.16	0.51403397	a.142.1.1	1
1KU3A	a.112.1.1	73	2.07	7.21	8	0.90077846	a.112.1.1	4
1pbwa	a.116.1.1	184	11.06	11.57	15.82	0.50117126	a.116.1.1	4
d1f7ca_	a.116.1.1	191	11.6	14.18	17.81	0.68828245	a.116.1.1	4
1kx8A	a.118.1.12	100	2.49	5.18	6.19	0.87112816	a.74.1.2	1
1K19A	a.118.1.12	112	3.57	5.74	8.12	0.79282955	a.1.1.2	1
1ocre	a.118.11.1	109	4.94	3.96	4.93	0.99366934	a.118.11.1	5
1kpsb	a.118.12.1	156	9	7.7	10.4	0.94834165	a.118.12.1	5
d1k8kg_	a.118.13.1	143	8.12	18.42	20.17	0.9258585	a.118.13.1	4
1qc7a	a.118.14.1	101	3.06	5.48	8.24	0.98591533	a.118.14.1	5
1l5ja	a.118.15.1	160	9.14	9	12.59	0.85032797	a.118.15.1	5
1klxa	a.118.18.1	133	4.85	7.54	11.37	0.96888648	a.118.18.1	5
1BC9	a.118.3.1	200	12.25	10.62	10.85	0.65233462	a.118.3.1	4
1pbv_	a.118.3.1	195	11.64	10.41	15.73	0.66294739	a.118.3.1	5
1iyga	a.118.8.1	133	4.71	5.21	6.5	0.9997454	a.118.8.1	5

1hxiA	a.118.8.1	121	5.98	14.13	17.38	0.90098008	a.118.8.1	4
1eyha	a.118.9.1	144	7.67	7.56	10.6	0.98969278	a.118.9.1	5
1kdxa	a.12.1.1	81	3.17	3.91	5.02	0.95429733	a.12.1.1	5
d2tct_2	a.121.1.1	141	8.76	14.55	18.78	0.7483137	a.26.1.1	1
1jt6a	a.121.1.1	115	8.18	6.97	8.29	0.73294619	a.24.3.2	1
1kxpd	a.126.1.1	69	2.13	3.28	5.88	0.86357023	a.126.1.1	5
1kxpd	a.126.1.1	190	10.97	11.62	13.95	0.95592817	a.126.1.1	5
1lre_	a.13.1.1	81	4.91	3.97	5.63	0.95850797	a.13.1.1	5
1ecma	a.130.1.1	91	3.98	6.04	7.09	0.97641716	a.130.1.1	5
1pprm	a.131.1.1	156	9.54	11.02	15.03	0.54114289	a.131.1.1	5
d1j77a_	a.132.1.2	200	13.13	12.88	15.02	0.79697643	a.132.1.2	4
1FE5A	a.133.1.2	118	9.27	8.83	10.38	0.5155915	a.133.1.2	4
1DPYA	a.133.1.2	118	8.8	11.49	14.85	0.43816265	a.133.1.2	4
1BEO	a.134.1.1	98	7.15	6.25	9.9	0.71198748	a.134.1.1	4
1lria	a.134.1.1	98	7.88	6.27	10.5	0.78439199	a.24.17.1	1
1g8qa	a.135.1.1	90	5.8	6.55	9.09	0.79939049	a.23.4.1	1
1dvoa	a.136.1.1	152	10.88	15.59	18.02	0.85659227	a.52.1.2	1
1g72b	a.137.2.1	57	5.25	11.67	14.1	0.42256774	a.159.2.1	1
1tbge	a.137.3.1	68	4.29	15.1	18.39	0.54273259	a.46.2.1	1
1gg2g	a.137.3.1	54	3.37	14.81	18.48	0.59721466	d.47.1.1	0
1hfes	a.137.4.1	88	6.69	14.01	16.77	0.69962679	d.58.17.1	0
d1ef1c_	a.137.5.1	90	6.66	12.59	15.37	0.528333	a.71.2.1	1
d2prgc_	a.137.6.1	76	6.19	25.04	30.63	0.5124204	a.4.8.1	1
1e79i	a.137.8.1	47	2.07	8.67	10	0.25448629	d.45.1.1	0
1L3OA	a.138.1.1	68	5.26	5.54	5.83	0.4679941	a.52.1.1	1
1hh5a	a.138.1.1	68	6.68	5.93	6.45	0.28341545	d.58.17.1	0
1dava	a.139.1.1	71	4.49	7.9	10.31	0.49467813	a.85.1.1	1
1qqvA	a.14.1.1	67	3.27	6.37	11.11	0.65379492	a.164.1.1	1
1vii_	a.14.1.1	36	0.97	2.28	2.54	0.4195326	a.14.1.1	5
1jeia	a.140.1.1	53	3.4	5.16	8.07	0.54302496	a.140.1.1	5
1h9eA	a.140.1.1	56	3.11	5.06	8.16	0.57607309	a.140.4.1	2
1JJRA	a.140.2.1	151	10.44	13.34	15.53	0.18544543	a.118.13.1	1
1h1jS	a.140.2.1	51	3.19	8.21	11.65	0.4559823	a.140.1.1	2
d1a62_1	a.140.3.1	47	1.08	1.33	2.86	0.6860198	a.140.4.1	2

1e7la	a.140.4.1	54	2.11	2.44	4.43	0.80574968	a.140.4.1	5
1ijya	a.141.1.1	122	8.41	7.84	12.32	0.33668163	a.133.1.3	1
1h99a	a.142.1.1	115	4.05	4.15	7.21	0.99531199	a.142.1.1	5
d1h99a2	a.142.1.1	107	4.69	5.14	5.5	0.9973688	a.142.1.1	4
1iw7e	a.143.1.1	95	7.65	7.42	11.16	0.47148217	a.118.9.2	1
1ifwa	a.144.1.1	92	3.76	6.56	7.9	0.96767589	a.144.1.1	5
1i2ta	a.144.1.1	61	1.8	2.5	3.39	0.94961566	a.144.1.1	5
1gyza	a.144.2.1	60	1.82	2.49	3.32	0.96372375	a.144.2.1	5
1g8ea	a.145.1.1	98	6.65	8.5	10.25	0.81423113	a.145.1.1	5
d1k1fa_	a.147.1.1	67	3.76	11.46	14.36	0.78909978	a.74.1.1	1
1k8ke	a.148.1.1	174	11.27	11.78	14.82	0.78559112	f.13.1.1	0
1o0wa	a.149.1.1	169	9.28	9.62	12.61	0.9853512	a.149.1.1	4
1tbaa	a.15.1.1	67	5.37	6.37	9.7	0.38954488	a.60.3.1	1
d1gpja1	a.151.1.1	102	2.78	17.1	19.78	0.970604	a.23.1.1	1
1knca	a.152.1.1	174	9.55	8.29	9.53	0.79587716	a.152.1.1	5
1JJSA	a.153.1.1	50	1.49	5.11	6.89	0.7149052	a.4.10.1	1
1hns_	a.155.1.1	47	2.16	4.14	6.17	0.45776417	g.7.1.3	0
1l1za	a.156.1.2	88	3.79	4.92	10.48	0.59692462	a.156.1.2	4
1mu5a	a.156.1.3	78	3.03	3.51	6.08	0.98487821	a.156.1.3	5
1fs1b	a.157.1.1	55	1.94	3.83	5.69	0.8367471	a.46.2.1	1
1nexb	a.158.1.1	100	5.56	5.78	7.56	0.76599951	a.158.1.1	5
1fs1a	a.158.1.1	41	1.68	2.65	3.76	0.59244214	a.158.1.1	5
d1a6q_1	a.159.1.1	72	2.85	17.23	18.26	0.92671867	a.112.1.1	1
1H40A	a.159.2.1	71	2.67	3.96	5.5	0.94031187	a.159.2.1	4
1r1bA	a.16.1.3	56	2.04	3.53	4.75	0.62906323	a.16.1.3	4
1fyja	a.16.1.3	57	1.61	2	5.53	0.93828117	a.16.1.3	5
d1f5aa3	a.160.1.1	150	10.58	9.57	11.81	0.38302315	a.74.1.3	1
d1m1eb_	a.161.1.1	70	4.12	16.18	18.72	0.71366486	a.4.1.1	1
d1m6na1	a.162.1.1	122	5.42	5.39	10.76	0.9823432	a.162.1.1	4
1j0ta	a.163.1.1	78	3.97	4.07	4.97	0.98903091	a.163.1.1	5
1IYRA	a.164.1.1	111	8.72	10.25	13.38	0.30088368	f.14.1.1	0
1koya	a.164.1.1	62	3.2	5.91	9.62	0.66560505	a.144.1.1	1
1K5OA	a.165.1.1	86	5.85	9.5	9.94	0.82461504	a.52.1.1	1
1p0ya	a.166.1.1	176	11.73	14.14	17.98	0.41351338	c.54.1.1	0

1gzsb	a.168.1.1	165	10.41	9.52	15.59	0.49082622	c.23.4.1	0
2hp8_	a.17.1.1	68	3.09	4.7	5.7	0.81344488	a.17.1.1	5
d1iyjb2	a.171.1.1	137	8.38	27.38	29.54	0.63571748	a.4.12.1	1
1k6ka	a.174.1.1	142	7.09	6.71	10.7	0.82437456	a.174.1.1	5
1m98a	a.175.1.1	174	10.39	13.72	17.29	0.13726901	c.37.1.19	0
d1k87a1	a.176.1.1	175	9.69	24.66	27.78	0.5340396	c.108.1.1	0
2end_	a.18.1.1	137	10.14	9.16	13.39	0.38913977	d.43.1.1	0
1enk_	a.18.1.1	137	10.06	9.81	12.58	0.71807384	a.18.1.1	4
2lisa	a.19.1.1	131	8.46	9.81	12.72	0.99289689	a.19.1.1	5
1aqt_	a.2.10.1	50	1.81	1.72	3.02	0.88507751	a.2.10.1	5
1e79h	a.2.10.1	45	2.86	4.84	5.9	0.43444178	a.2.10.1	5
d1qnna1	a.2.11.1	84	4.64	6.35	8.78	0.74597538	a.2.11.1	4
1coja	a.2.11.1	89	5.21	6.6	9.35	0.92819748	a.23.1.1	1
1jj2u	a.2.2.1	65	1.23	2.33	3.39	0.87589657	a.2.2.1	5
1hdj_	a.2.3.1	77	2.42	3.16	3.99	0.96763473	a.2.3.1	5
1BQZ	a.2.3.1	77	3.33	4.89	5.76	0.95965157	a.2.3.1	4
1du2a	a.2.4.1	76	5.12	10.59	13.52	0.47175478	c.47.1.1	0
d1fxka_	a.2.5.1	107	5.56	3.62	4.47	0.97263516	a.2.5.1	4
1fxkc	a.2.5.1	133	5.21	5.48	6.68	0.99607571	a.2.5.1	5
1cxzb	a.2.6.1	86	3.41	6.81	12.59	0.72616741	a.44.1.1	1
1ivsa	a.2.7.3	66	3.38	5.72	6.98	0.85809495	a.2.7.3	5
1lrza	a.2.7.4	65	3.38	7.91	8.46	0.56011242	a.46.2.1	1
1k4ta	a.2.8.1	72	4.68	5.66	8.11	0.89548689	a.2.8.1	5
1e52a	a.2.9.1	56	1.28	6.76	7.84	0.68935072	a.2.9.1	5
d1l6ja1	a.20.1.2	77	3.83	22.92	24.39	0.6479286	a.20.1.1	3
d1slm_1	a.20.1.2	65	3.62	39.45	40.97	0.50676784	a.28.1.1	1
1HME	a.21.1.1	77	3.42	5.88	8.49	0.89283254	a.21.1.1	4
1qrva	a.21.1.1	73	3.32	5.83	8.35	0.90690269	a.159.1.1	1
1a7w_	a.22.1.2	68	1.97	6.16	9.73	0.79823074	a.39.1.1	1
1hta_	a.22.1.2	68	2.54	5.65	9.21	0.89361483	a.179.1.1	1
1fpoa	a.23.1.1	95	1.98	2.97	5.4	0.99667196	a.23.1.1	5
1eexg	a.23.2.1	137	8.31	10.41	12.39	0.70252754	a.23.2.1	5
1om2a	a.23.4.1	95	3.37	7.9	9.04	0.95839323	a.23.4.1	5
1jw2a	a.23.5.1	72	2.93	5.75	8.71	0.67932888	a.23.5.1	5

1LE2	a.24.1.1	144	5.86	7.48	10.83	0.93600205	a.2.5.1	1
1LE4	a.24.1.1	144	7.81	15.19	15.19	0.95161071	a.24.1.1	4
1a0b_	a.24.10.1	117	4.82	3.6	13.41	0.99948628	a.24.10.1	4
2a0b_	a.24.10.1	118	4.66	3.83	13.19	0.99742645	a.24.10.1	5
1he9A	a.24.11.1	134	8.76	7.22	10.51	0.75266328	f.14.1.1	0
1G5ZA	a.24.12.1	164	10.01	11	13.97	0.7800039	a.24.1.1	2
1j8mf	a.24.13.1	84	2.53	2.67	3.18	0.99326731	a.24.13.1	5
1fts_	a.24.13.1	84	2.25	4.02	5.05	0.94374772	a.24.13.1	4
1k04a	a.24.14.1	142	9.73	12.4	14.14	0.97537323	f.13.1.1	0
1K40A	a.24.14.1	126	7.76	3.93	5.16	0.94854501	f.36.1.1	0
1jr8a	a.24.15.1	105	5.19	5.88	10.09	0.82319237	a.118.8.1	1
1knya	a.24.16.1	128	8.42	7.5	9.82	0.72550804	a.24.16.1	5
d1joga_	a.24.16.2	135	8.02	3.88	5.44	0.82690645	a.19.1.1	1
1nlxa	a.24.17.1	104	4.97	4.97	8.02	0.9868818	a.24.17.1	5
2ASR	a.24.2.1	142	9.27	5.91	5.91	0.90408208	a.24.3.2	2
1WAS	a.24.2.1	146	7.86	6.9	9.64	0.98911356	a.24.12.1	2
256ba	a.24.3.1	106	2.61	3.12	4.64	0.99098845	a.24.3.2	3
1M6TA	a.24.3.1	106	2.96	2.63	4.68	0.99115395	a.24.3.1	4
1HRB	a.24.4.1	113	4.7	5.6	10.49	0.97632869	a.24.4.1	4
2mhr_	a.24.4.1	118	4.03	6.73	8.67	0.9968477	a.24.4.1	5
1dova	a.24.9.1	181	7.89	8.48	22.46	0.9993633	a.24.9.1	5
1krqA	a.25.1.1	165	6.06	4.5	6.39	0.99846872	a.25.1.1	4
1rce_	a.25.1.1	171	9.01	7.75	9.16	0.97830265	a.25.1.1	4
1jli_	a.26.1.2	112	8	7.83	10.86	0.60611922	a.118.8.1	1
1ik0a	a.26.1.2	113	8.33	8.17	8.95	0.5257204	a.118.8.1	1
1f7ua	a.27.1.1	124	5.77	3.7	10.38	0.98319162	a.27.1.1	5
1li5a	a.27.1.1	87	2.67	4.06	9.14	0.9733483	a.27.1.1	5
1T8KA	a.28.1.1	77	2.38	3.74	5.2	0.99468691	a.28.1.1	4
1hy8A	a.28.1.1	76	2.27	2.82	3.57	0.99387814	a.28.1.1	4
1gxgA	a.28.2.1	85	4.79	5.83	10.42	0.81887617	a.28.2.1	4
1IMQ	a.28.2.1	86	4.46	4.92	5.56	0.83652045	a.28.2.1	4
1a43_	a.28.3.1	72	3.08	2.86	4.36	0.97692859	a.28.3.1	4
1AUM	a.28.3.1	70	3.49	3.49	5.16	0.8719037	a.28.3.1	4
1n72A	a.29.2.1	115	6.09	6.95	8.94	0.78074761	a.74.1.1	1

1f68a	a.29.2.1	103	4.39	4.14	6.6	0.95523043	a.29.2.1	5
1jqia	a.29.3.1	153	7.57	8.11	12.71	0.99819914	a.29.3.1	5
1ivha	a.29.3.1	151	5.01	5.33	5.78	0.99577086	a.29.3.1	4
1gm5a	a.29.4.1	99	5.38	7.2	11.81	0.7388583	a.7.2.1	1
d1jm6a1	a.29.5.1	167	10.07	9.59	12.08	0.98198421	a.29.5.1	4
1gkza	a.29.5.1	148	8.91	7.73	9.49	0.97203835	a.29.5.1	5
1c75a	a.3.1.1	71	3.74	3.85	6.76	0.65875318	a.178.1.1	1
1ropA	a.30.1.1	56	2.45	2.34	8.67	0.83605246	a.8.1.1	1
1b6q_	a.30.1.1	56	2.79	4.8	8.89	0.8335688	g.2.2.1	0
1joya	a.30.2.1	67	1.61	8.62	10.61	0.97619516	a.7.7.1	1
1b3qa	a.30.2.1	62	1.76	4.49	6.11	0.85166323	a.30.2.1	5
1r2aa	a.31.1.1	46	1.7	7.74	8.66	0.67151798	a.8.1.1	1
1ytfd	a.32.1.1	50	1.3	3.27	5.96	0.70462739	a.32.1.1	5
1ytfb	a.32.1.1	46	1.8	2.8	4.71	0.61631909	a.4.1.3	1
1ecia	a.33.1.1	37	1.22	3.61	4.4	0.32406557	a.4.10.1	1
1PRU	a.35.1.5	56	1.65	5.31	5.83	0.6823468	a.35.1.5	4
d1hq1a_	a.36.1.1	104	3.38	7.29	8.81	0.94761234	a.36.1.1	4
d1qb2a_	a.36.1.1	107	3.95	7.29	9.49	0.98412248	a.36.1.1	4
1k1va	a.37.1.1	41	0.92	2.51	3.74	0.6019454	a.37.1.1	5
1a0aa	a.38.1.1	63	3.35	8.62	11.52	0.78871816	a.30.2.1	1
1an4a	a.38.1.1	65	3.53	8.12	12.26	0.81694382	a.2.2.1	1
1F71A	a.39.1.5	67	1.42	2.44	4.16	0.91474083	a.39.1.5	4
1c3za	a.39.2.1	108	5.33	7.8	10.95	0.53713647	a.28.1.3	1
d1cpo_1	a.39.3.1	120	8.98	0	0	0.50306379	a.39.3.1	4
1iioa	a.39.4.1	84	5.36	4.64	4.64	0.9612805	a.39.4.1	5
1gdta	a.4.1.2	43	0.94	3.44	4.64	0.53914497	a.4.10.1	2
1mbj_	a.4.1.3	52	1.64	3.69	5.14	0.77606319	a.4.1.3	4
1k6ya	a.4.10.1	46	1.32	3.03	4.04	0.60097049	a.4.10.1	5
1ef4A	a.4.11.1	55	1.47	3.29	3.92	0.86175043	a.4.11.1	4
1jhga	a.4.12.1	101	3.51	6.3	7.24	0.8864778	a.4.12.1	5
1WRPR	a.4.12.1	107	3.82	7.46	7.76	0.9479786	a.4.12.1	4
1sfe_	a.4.2.1	84	5.62	4.76	8.95	0.78325892	a.4.2.1	5
1mgta	a.4.2.1	81	4.14	3.75	4.37	0.97575461	a.4.2.1	5
1ig6a	a.4.3.1	107	7.36	6.99	7.9	0.84002389	a.4.3.1	4

1kkxa	a.4.3.1	102	5.75	5.24	8.21	0.979682	a.4.3.1	5
2bby_	a.4.5.15	69	3.41	4	5.31	0.88012984	a.4.5.15	5
1QQIA	a.4.6.1	104	4.67	3.84	4.27	0.99172266	a.4.6.1	4
1gxqa	a.4.6.1	105	4.95	4.29	4.29	0.99464166	a.4.6.1	5
1FOW	a.4.7.1	76	3.55	3.34	4.55	0.93887071	a.4.7.1	4
1fjgr	a.4.8.1	73	5.5	5.74	8.38	0.68374619	a.4.8.1	5
1e3ha	a.4.9.1	83	2.34	2.95	4.94	0.99657517	a.4.9.1	5
1bkra	a.40.1.1	108	6.9	5.51	9.48	0.82865702	a.40.1.1	5
1lnsa	a.40.2.1	145	9.8	9.98	11.64	0.5978624	a.40.2.1	5
1a26_	a.41.1.1	135	9.4	8.15	12.66	0.70718048	f.36.1.1	0
d1gs0a1	a.41.1.1	134	8.54	9.83	13.35	0.64518332	d.56.1.2	0
1ycqa	a.42.1.1	88	4.9	4.91	9.57	0.36908784	a.13.1.1	1
1irqa	a.43.1.2	48	1.67	5.79	9.28	0.58420055	b.35.2.1	0
2cpga	a.43.1.2	43	2.14	3.56	5.07	0.42931976	a.5.1.1	1
1bed_	a.44.1.1	64	2.02	3.45	4.94	0.8909024	a.65.1.1	1
1fvka	a.44.1.1	64	2.1	2.85	3.48	0.94790143	a.44.1.1	4
1a0fa	a.45.1.1	121	5.16	4.6	5.22	0.98615848	a.45.1.1	5
1bmta	a.46.1.1	90	2.76	3.71	6.32	0.99704521	a.46.1.1	5
1khda	a.46.2.1	69	1.57	5.12	5.78	0.88155447	a.48.3.1	1
1brwa	a.46.2.1	70	1.94	4.42	6.73	0.94849615	a.46.2.1	5
d1bg1a1	a.47.1.1	186	8.97	36.61	38.31	0.99958213	a.47.1.1	4
1BR0A	a.47.2.1	120	4.95	4.08	5.78	0.99591235	a.47.2.1	4
2cbla	a.48.1.1	131	7.84	6.33	9.5	0.72495247	a.48.1.1	5
1de4c	a.48.2.1	148	8.71	7.69	10.5	0.91057434	a.48.2.1	5
1eo0a	a.48.3.1	77	4.47	5.14	8	0.74418681	a.46.2.1	1
1f6va	a.49.1.1	91	3.62	9.37	11.08	0.60653397	a.64.1.2	1
1ixsa	a.5.1.1	50	1.04	1.1	1.73	0.88946694	a.5.1.1	5
1cuk_	a.5.1.1	48	1.23	1.85	3.03	0.84254216	a.5.1.1	5
1DV0A	a.5.2.1	47	0.98	5.42	6.1	0.78640719	a.5.7.1	2
1f4ia	a.5.2.1	45	0.98	2.17	3.26	0.66753851	a.5.2.1	5
1aua_	a.5.3.1	93	5.16	6.45	9.72	0.95152428	a.5.3.1	5
1enwa	a.5.4.1	114	4.95	11.13	14.5	0.76219924	a.1.1.2	1
1EIJA	a.5.6.1	80	2.6	6.42	6.93	0.84146869	a.5.6.1	4
1C5A	a.50.1.1	73	3.29	9.36	13.79	0.88551685	a.50.1.1	4

1kjs_	a.50.1.1	74	3.58	3.9	4.25	0.91184651	a.50.1.1	4
1ocrh	a.51.1.1	79	4.26	4.89	6.04	0.91865986	a.51.1.1	5
1HYP	a.52.1.1	80	5.58	6.78	8.35	0.92314585	a.52.1.1	4
1l6ha	a.52.1.1	69	3.61	3.57	4.55	0.96718956	a.52.1.1	5
1adt_	a.54.1.1	90	5.46	7.77	12.36	0.9028147	a.112.1.1	1
1dp3a	a.55.1.2	55	2.27	6.43	7.77	0.59761275	a.4.1.7	1
1fo4a	a.56.1.1	73	4	4.35	6.59	0.82279195	a.46.2.1	1
1n62a	a.56.1.1	82	4.82	4.8	6.85	0.66802544	a.56.1.1	5
1dj8a	a.57.1.1	79	6.28	4.79	7.36	0.45401755	a.4.5.14	1
1af7_	a.58.1.1	81	2.8	3.38	6.06	0.98996194	a.58.1.1	5
1g1eb	a.59.1.1	89	4.71	5.76	6.31	0.95446612	a.59.1.1	5
1jbga	a.6.1.3	106	4.92	9.01	12.92	0.58961074	a.6.1.3	5
1dxsa	a.60.1.2	57	1.4	1.26	1.59	0.92878289	a.60.1.2	5
1f0mA	a.60.1.2	71	3.06	3.09	6.75	0.97624695	a.60.1.2	4
1zyma	a.60.10.1	123	3.72	3.94	4.98	0.9994429	a.60.10.1	5
1jyga	a.60.11.1	69	2.8	3.24	9.41	0.96350433	a.60.11.1	5
1jmsa	a.60.12.1	60	2.24	3.11	3.86	0.93638687	a.60.12.1	5
1jn3a	a.60.12.1	58	1.89	1.65	3.7	0.94219457	a.60.12.1	5
d1m6ya1	a.60.13.1	101	5.79	6.15	10.48	0.49299608	a.1.1.2	1
1KFTA	a.60.2.3	78	2.99	7.24	8.76	0.92937393	a.60.1.2	2
1doqa	a.60.3.1	69	1.95	2.57	3.74	0.98673395	a.60.3.1	5
1coo_	a.60.3.1	81	4.65	3.77	5.72	0.9739397	a.60.3.1	4
1B22A	a.60.4.1	114	6.02	10.78	12.07	0.68574696	a.60.1.2	2
2EZZA	a.60.5.1	89	3.11	4.17	10.29	0.95777902	a.74.1.1	1
1BNO	a.60.6.1	87	3.87	5.71	8.89	0.98357974	a.60.6.1	4
1xo1a	a.60.7.1	105	7.46	9.71	10.25	0.38028328	a.7.3.1	1
1a77_	a.60.7.1	108	6.84	13.91	15.64	0.40881725	a.36.1.1	1
1d8ba	a.60.8.1	81	5.23	5.11	6.64	0.67782477	a.60.2.1	2
1f44a	a.60.9.1	110	7.09	5.97	11.99	0.93461606	a.60.9.1	5
d1a0p_1	a.60.9.1	98	2.04	16.25	17.05	0.98475372	a.60.9.1	4
1bax_	a.61.1.3	94	4.23	4.27	4.65	0.99019853	a.61.1.3	5
1a6s_	a.61.1.4	87	2.9	4.45	4.72	0.97827768	a.61.1.4	5
1qgta	a.62.1.1	142	9.3	11.13	13.87	0.51941735	a.62.1.1	5
1eq1a	a.63.1.1	166	8.23	8.69	13.61	0.8681215	a.47.1.1	1

1AEP	a.63.1.1	161	9.8	11.27	17.84	0.74601611	a.7.4.1	1
1l9la	a.64.1.1	74	3.2	3.22	4.39	0.95891134	a.64.1.1	4
1nkl_	a.64.1.1	78	2.58	3.42	4.82	0.9658272	a.64.1.1	5
1e68a	a.64.2.1	70	2.59	3.96	4.34	0.97056654	a.64.2.1	5
1bo9a	a.65.1.1	73	2.02	3.31	7.63	0.98802726	a.65.1.1	5
1azsc	a.66.1.1	116	7.32	6.06	8.91	0.80440375	a.66.1.1	4
1cipa	a.66.1.1	121	8.14	6.39	11.04	0.77215227	a.66.1.1	5
1ej5a	a.68.1.1	107	6.77	6.82	10.5	0.95568165	a.68.1.1	5
1e79d	a.69.1.1	118	7.36	7.82	9.09	0.94715523	a.69.1.1	5
1e79a	a.69.1.1	131	7.2	8.95	12.16	0.79718917	a.77.1.3	1
d1fkma1	a.69.2.1	194	12.36	10.67	13.33	0.44740806	c.37.1.12	0
d1fkma2	a.69.2.1	188	12.09	15.57	22.8	0.23704097	f.13.1.1	0
d1jvsa1	a.69.3.1	99	3.89	6.13	7.02	0.99443434	a.69.3.1	4
1AJ3	a.7.1.1	110	2.95	10.59	13.05	0.99826959	a.7.4.1	2
1e2aA	a.7.2.1	102	2.82	3.26	3.96	0.99643009	a.7.2.1	4
1chua	a.7.3.1	111	6.29	7.99	12.67	0.81971721	a.24.7.1	1
1kf6a	a.7.3.1	134	8.99	9.33	9.5	0.49285704	a.74.1.3	1
1FEWA	a.7.4.1	184	10.69	15.65	16.89	0.98991693	a.7.4.1	4
1h7cA	a.7.5.1	108	3.55	16.52	17.47	0.99819332	a.7.5.1	4
1fjgt	a.7.6.1	99	3.49	4.9	7.55	0.99489342	a.7.6.1	5
1M62A	a.7.7.1	87	3.32	4	5.86	0.97317845	a.7.3.1	2
1M7KA	a.7.7.1	99	5.17	6.89	7.94	0.96202482	a.7.7.1	4
1abv_	a.70.1.1	105	3.38	3.24	3.64	0.99979364	a.70.1.1	5
1g7da	a.71.1.1	106	3.44	4.63	5.77	0.99683954	a.71.1.1	5
1m2oa	a.71.2.1	103	6.6	6.05	10.59	0.94936943	a.71.2.1	4
1m2vb	a.71.2.1	107	6.2	4.88	6.89	0.90599249	a.24.17.1	1
d1dvka_	a.72.1.1	169	10.03	21.47	24.07	0.85370805	a.27.1.1	1
1g03A	a.73.1.1	134	6.85	7.32	9.67	0.73632953	a.73.1.1	4
1GDZ	a.73.1.1	151	10.43	11.38	14.07	0.32908247	a.73.1.1	4
1AD6	a.74.1.3	185	11.47	10.26	11.15	0.53716171	a.74.1.3	4
1RSS	a.75.1.1	151	5.02	18.65	23.31	0.54713166	a.118.13.1	1
1hus_	a.75.1.1	155	7.36	15.4	20.08	0.61262343	a.133.1.3	1
1fx7a	a.76.1.1	80	3.47	5.34	7.23	0.86323712	a.76.1.1	5
1ngr_	a.77.1.2	85	4.13	4.76	6.84	0.85908822	a.77.1.2	5

1dgna	a.77.1.3	89	3.07	4.09	8.48	0.92392039	a.77.1.3	5
1hw1a	a.78.1.1	152	8.24	8.01	14	0.86534084	a.29.3.2	1
1ey1a	a.79.1.1	139	5.57	5.61	7.48	0.88359602	a.79.1.1	4
1TF0B	a.8.1.2	53	1.46	3.32	5.88	0.79499804	a.60.1.2	1
d1gvna_	a.8.2.1	88	5.31	6.62	11.88	0.65050359	a.27.1.1	1
1iqpa	a.80.1.1	95	3.18	2.18	5.44	0.99437423	a.80.1.1	5
d1a5t_1	a.80.1.1	123	4.83	11.22	14.65	0.95331752	a.1.1.2	1
1JWEA	a.81.1.1	114	5.09	8.04	11.65	0.78314209	a.81.1.1	4
1qh4a	a.83.1.1	101	7.67	7.94	12.16	0.51009156	a.60.5.1	1
1m15a	a.83.1.1	94	6.39	7.28	8.89	0.6881869	a.77.1.2	1
1hc2_	a.85.1.1	131	8.34	7.86	12.11	0.83532181	a.85.1.1	5
d1lla_1	a.85.1.1	108	6.65	12.4	14.3	0.95274841	a.85.1.1	4
d1kz7a1	a.87.1.1	195	11.88	9.79	14.96	0.68034169	a.63.1.1	1
1boua	a.88.1.1	132	6.72	7.47	12.12	0.73290651	a.88.1.1	5
2pde_	a.9.1.1	43	1.46	6.58	7.99	0.620554	a.9.1.1	4
1bbl_	a.9.1.1	37	1.28	3.22	4.79	0.24793174	a.5.1.1	1
1bgf_	a.90.1.1	124	5.37	6.21	12.1	0.93959061	a.90.1.1	5
1dk8a	a.91.1.1	147	8.28	9.13	18.08	0.72181314	a.24.13.1	1
1FQJB	a.91.1.1	147	8.48	9.78	14.48	0.4436977	a.118.8.1	1
1a9xa	a.92.1.1	153	8.3	9.38	10.76	0.99069494	a.92.1.1	5
1jj2o	a.94.1.1	143	9.33	21.31	24.11	0.52822612	a.94.1.1	5
1aa7a	a.95.1.1	158	10.43	9.83	12.42	0.88418846	a.95.1.1	5
1nkua	a.96.1.4	187	12.33	12.7	13.77	0.34193891	a.96.1.4	5
1j09a	a.97.1.1	163	7.18	9.03	13.8	0.69319846	a.97.1.1	5
1wiu_	b.1.1.4	93	4.01	6.56	10.94	0.92277441	b.1.1.4	5
3NCMA	b.1.1.4	92	4.72	6.91	14.08	0.74827239	b.61.3.1	1
1GYUA	b.1.10.2	140	9.72	10.24	15.95	0.51715735	b.61.1.1	1
1gyva	b.1.10.2	120	7.86	8.23	11.23	0.88941262	b.1.10.2	5
d1l4ia1	b.1.11.1	120	10.18	10.32	10.87	0.41881147	f.4.1.1	0
d1qpxa1	b.1.11.1	124	9.98	22.24	25.36	0.31041466	b.61.1.1	1
4kbpa	b.1.12.1	112	8.08	10.13	11.71	0.51941897	d.54.1.1	0
1dqia	b.1.13.1	124	10.04	9.77	11.28	0.23983169	d.131.1.2	0
1dfx_	b.1.13.1	89	6.33	6.15	8.56	0.94009584	b.61.4.1	1
1f00i	b.1.14.1	89	4.06	8.54	12.95	0.65040356	b.101.1.1	1

1cwva	b.1.14.1	91	7.18	6.89	9.83	0.82998329	b.24.1.1	1
1ifra	b.1.16.1	113	8.55	9.6	14.58	0.92129447	b.1.16.1	5
1ivtA	b.1.16.1	122	9.52	10.33	14.51	0.27613567	b.54.1.1	1
1M42A	b.1.18.17	102	9.85	8.97	11.24	0.46469297	b.1.18.1	3
1lmia	b.1.19.1	131	10.27	12.58	17.23	0.24251138	b.60.1.2	1
1ten_	b.1.2.1	89	3.11	2.84	6.55	0.99988597	b.1.2.1	5
1fna_	b.1.2.1	91	5.63	4.77	7.58	0.98132069	b.1.2.1	4
1o75a	b.1.20.1	82	3.94	6.76	10.82	0.90523787	d.47.1.1	0
1o75a	b.1.20.1	126	10.37	12.3	15.6	0.08680342	a.28.1.1	0
1b4ra	b.1.3.1	80	7.76	7.75	9.02	0.53040685	d.17.2.1	0
1jz8a	b.1.4.1	105	8.9	8.94	8.94	0.74615139	b.1.2.1	2
1bhga	b.1.4.1	103	6.01	6.96	8.1	0.91933743	b.1.4.1	5
1f13a	b.1.5.1	101	8.32	7.43	8.85	0.61603435	b.1.18.2	2
1l9na	b.1.5.1	99	8.3	7.58	9.01	0.68914347	b.1.1.2	2
1ncg_	b.1.6.1	99	6.96	7.41	10.62	0.77260862	d.19.1.1	0
1SUH	b.1.6.1	146	11.03	9.99	9.99	0.22993371	b.61.1.1	1
1HZKA	b.1.7.1	110	8.18	9.94	12.18	0.52720334	b.12.1.1	1
1ACX	b.1.7.1	108	7.25	9.43	13.3	0.70277705	b.61.4.1	1
1ej8a	b.1.8.1	140	10.37	12.5	16.04	0.15809286	a.118.8.1	0
1IBHA	b.1.8.1	151	11.56	10.43	13.57	0.22650896	b.1.8.1	4
1ijaa	b.100.1.1	148	11.07	11.01	11.34	0.60538061	b.60.1.2	1
1e44b	b.101.1.1	96	7.06	8.28	13.66	0.8818238	b.101.1.1	5
1k91a	b.104.1.1	37	3.85	7.14	10.06	0.01411067	d.28.1.1	0
1k9cA	b.104.1.1	74	8.21	13.31	15.78	0.13475447	a.60.5.1	0
1nj4a	b.105.1.1	93	6.58	8.36	13.31	0.64349528	b.105.1.1	5
1k0ha	b.106.1.2	117	8.09	12.53	16.33	0.48037618	b.61.4.1	1
1eara	b.107.1.1	74	4.41	5.32	7.53	0.94929963	b.107.1.1	4
1gmua	b.107.1.1	70	5.3	5.52	7.95	0.81629382	b.107.1.1	5
1f53a	b.11.1.4	84	6.69	7.75	9.52	0.53258031	b.101.1.1	1
1g6ea	b.11.1.6	87	6.64	9.62	11.66	0.7159472	d.50.2.1	0
1K8HA	b.111.1.1	133	9.59	11.5	15.1	0.28657491	c.16.1.1	0
1J1HA	b.111.1.1	144	10.56	12.88	14.6	0.34792488	b.111.1.1	4
1js8a	b.112.1.1	101	8.36	8.5	9.56	0.83653185	b.112.1.1	5
1k3xa	b.113.1.1	124	9.1	11.94	15.66	0.53515239	b.60.1.2	1

1ee8a	b.113.1.1	121	8.14	10.4	13.51	0.67924559	c.47.1.3	0
1m1ha	b.114.1.1	81	4.25	5.36	11.26	0.83266463	b.61.3.1	1
1gzta	b.115.1.1	114	8.29	8.58	10	0.73920662	b.61.4.1	1
1o70a	b.118.1.1	140	9.95	8.5	9.72	0.47770367	b.118.1.1	5
1lox_	b.12.1.1	111	8.39	9.15	10.6	0.37677348	d.85.1.1	0
d1bu8a1	b.12.1.2	114	8.17	16.06	19.67	0.64878067	b.12.1.1	3
d1phm_1	b.13.1.2	154	12.75	16.13	19.64	0.09477617	d.20.1.2	0
d1k5ja_	b.13.3.1	105	8.77	11.75	13.56	0.53337102	b.61.1.1	1
1shsa	b.15.1.1	115	8.08	10.97	13.36	0.72846299	b.15.1.1	5
1ejfa	b.15.1.2	110	8.78	6.87	8.67	0.57256043	b.15.1.2	5
1IFGA	b.16.1.1	140	12.2	12.9	15.76	0.11901303	d.32.1.3	0
1ECY	b.16.1.1	142	12.04	13.45	16.21	0.08441208	b.60.1.2	1
1GMMA	b.18.1.10	133	8.25	10.87	13.59	0.41377255	b.61.4.1	1
1l7la	b.18.1.16	121	10.09	10.72	12.36	0.17088338	a.27.1.1	0
1ahsa	b.19.1.1	126	10.05	10.92	13.38	0.32766821	b.60.1.2	1
1e5ba	b.2.2.1	87	3.09	7.4	12.85	0.97744025	b.61.4.1	1
1XBD	b.2.2.1	87	3.49	7.71	13.66	0.97557696	b.61.4.1	1
1AMX	b.2.3.1	180	12.56	21.06	26.03	0.14320907	b.2.4.1	2
1bv8A	b.2.4.1	138	10.47	8.28	10.64	0.60733991	b.2.4.1	4
1eanA	b.2.5.6	114	7.93	10.64	15.27	0.43417577	d.87.1.1	0
1CO1A	b.2.5.6	115	8.37	11.59	12.99	0.503352	b.60.1.2	1
1aly_	b.22.1.1	146	11.94	12.4	14.28	0.15977352	d.32.1.3	0
1sfp_	b.23.1.1	111	8.49	8.28	10.28	0.4575482	b.61.4.1	1
1n7oa	b.24.1.1	76	3.23	3.35	5.67	0.98138859	b.24.1.1	4
d1f1sa3	b.24.1.1	65	3.37	3.98	6.12	0.97731257	b.24.1.1	4
1lgpa	b.26.1.2	113	7.78	9.86	12.98	0.67008811	d.110.1.1	0
1k3jA	b.26.1.2	151	12.07	11.88	14.88	0.12051222	b.42.1.1	1
1IS3A	b.29.1.3	135	9.67	11.51	15.58	0.30916768	b.29.1.3	4
1j18a	b.3.1.1	99	7.75	8.51	14.08	0.59655507	d.1.1.2	0
1acz_	b.3.1.1	108	8.38	9.05	12.19	0.60229086	b.45.1.1	1
1h8la	b.3.2.1	79	2.93	4.56	5.84	0.96408871	b.3.2.1	5
1f86a	b.3.4.1	115	9.01	7.34	10.34	0.86134662	b.3.4.1	5
1d2oa	b.3.5.1	90	5.71	6.4	9.78	0.94219925	b.1.2.1	1
1d2pa	b.3.5.1	96	6	5.19	8.23	0.95351046	b.1.1.2	1

1rie_	b.33.1.1	127	9.78	8.78	12.05	0.36935173	b.33.1.1	5
1BYMA	b.34.1.2	97	6.48	8.75	13.72	0.85728032	b.34.1.2	4
1ixda	b.34.10.1	104	9.52	11.4	13.44	0.29629125	d.16.1.6	0
1lpla	b.34.10.1	95	7.15	9.7	12.63	0.66353369	b.61.4.1	1
1m9sa	b.34.11.1	75	6.35	7.91	11.36	0.72087118	b.1.2.1	1
1m9sa	b.34.11.1	86	5.3	7.22	8.93	0.732704	b.34.11.1	5
1BK2	b.34.2.1	57	3.36	3.43	6.79	0.95425274	b.34.2.1	4
1kikA	b.34.2.1	57	2.98	3.1	3.91	0.985885	b.34.2.1	4
1lvk_	b.34.3.1	46	2.27	3.52	4.23	0.9281916	b.34.3.1	5
d2mysa1	b.34.3.1	46	1.4	1.96	2.81	0.95959188	b.34.3.1	4
1vie_	b.34.4.1	60	4.77	6.71	9.2	0.69779803	d.58.23.1	0
d1rl2a1	b.34.5.3	70	2.55	6.54	10.75	0.8853059	b.114.1.1	1
1m1ga	b.34.5.4	58	1.73	5.13	7.74	0.94905441	g.18.1.1	0
3vub_	b.34.6.1	101	7.67	9.79	14.22	0.48106193	d.87.1.1	0
1c0ma	b.34.7.1	53	2.35	4.03	5.81	0.94176143	d.9.1.1	0
1ex4a	b.34.7.1	48	2.11	2.19	3.16	0.98022726	b.34.7.1	5
1hyoa	b.34.8.1	118	8.8	7.85	11.95	0.62884044	a.80.1.1	0
1G5VA	b.34.9.1	88	6.74	8.92	10.45	0.68819378	d.58.18.1	0
1khca	b.34.9.2	135	11	9.65	11.44	0.25603893	a.74.1.1	0
1p3ha	b.35.1.1	99	8.8	9.99	13.95	0.36022453	b.61.2.1	1
1g31a	b.35.1.1	107	9.06	10.09	14.06	0.43378625	c.47.1.2	0
1auua	b.35.2.1	55	3.7	4.24	6.48	0.97235197	b.35.2.1	5
1GQ4A	b.36.1.1	90	5.85	7.87	9.49	0.97612053	b.36.1.1	4
1g9oa	b.36.1.1	91	5.4	8.36	9.51	0.90843226	b.36.1.1	5
1FGP	b.37.1.1	70	3.65	7.77	10.76	0.63268612	g.3.11.2	0
1ljoa	b.38.1.1	75	3.29	4.86	9.32	0.97980706	b.38.1.1	5
1ib8a	b.38.2.1	74	3.78	6.58	7.94	0.89368291	b.38.2.1	5
1whi_	b.39.1.1	122	8.97	9.43	13.77	0.29720778	d.14.1.7	0
1c3ga	b.4.1.1	80	5.75	5.29	5.29	0.97160263	b.4.1.1	5
1c3ga	b.4.1.1	90	7.56	7.78	9.8	0.74733986	b.4.1.1	5
1ENA	b.40.1.1	135	10.08	8.94	13.5	0.48288031	c.37.1.19	0
1BOVA	b.40.2.1	69	2.89	5.36	6.69	0.99419587	b.40.2.1	4
1jb3a	b.40.3.2	127	10.75	11.73	13.39	0.23809197	a.24.16.1	0
1jc7A	b.40.3.2	129	11	11.54	12.78	0.21809035	a.29.2.1	0

1CSQ	b.40.4.5	67	3.47	3.08	3.28	0.99067884	b.40.4.5	4
1INO	b.40.5.1	175	12.63	11.63	14.09	0.29950723	b.40.5.1	4
2PRD	b.40.5.1	174	12.24	11.01	12.08	0.27958742	b.40.5.1	4
1h9ra	b.40.6.2	62	1.68	4.82	7.96	0.99588122	b.40.6.2	5
1k0sa	b.40.7.1	151	11.27	10.77	11.53	0.6698368	b.40.7.1	5
1k28a	b.40.8.1	124	10.49	11.13	11.13	0.17574034	c.84.1.1	0
1j6qa	b.40.9.1	100	6.37	6.23	11.74	0.70667243	b.40.9.1	5
1J6QA	b.40.9.1	136	10.48	11.04	13.88	0.18118142	b.60.1.3	1
2BFH	b.42.1.1	128	9.02	9.76	13.27	0.60353432	d.131.1.2	0
1IJTA	b.42.1.1	128	8.7	7.86	12.54	0.45069542	b.42.1.1	4
1mc9A	b.42.2.1	126	6.79	11.02	12.68	0.78954761	b.60.1.1	1
1knma	b.42.2.1	129	7.23	11.2	11.56	0.61571554	d.82.2.1	0
1jlxa	b.42.3.1	153	12.16	12.45	13.04	0.12084854	d.134.1.1	0
1jlxa	b.42.3.1	146	11.09	11.92	12.56	0.24847009	b.42.3.1	5
1TIE	b.42.4.1	172	12.49	15.93	18.16	0.1054876	b.60.1.2	1
1hcd_	b.42.5.2	118	9.96	10.66	13.53	0.39754697	b.60.1.2	1
1d1na	b.43.3.1	99	8.42	9.18	13.3	0.66444545	d.109.1.1	0
1kzla	b.43.4.3	92	4.98	4.95	5.16	0.99883672	b.43.4.3	5
1exma	b.44.1.1	93	8.48	7	12.58	0.51746239	b.44.1.1	5
d1kk1a2	b.44.1.1	89	6.12	5.41	8.42	0.98754863	b.44.1.1	4
1flma	b.45.1.1	122	10.66	10.94	14.01	0.11557189	a.45.1.1	0
1ejea	b.45.1.2	192	12.87	13.38	16.07	0.15559005	c.45.1.1	0
1fmta	b.46.1.1	108	7.85	7.12	10.93	0.62708585	b.46.1.1	5
1kxc_	b.47.1.3	158	11.12	10.35	14.11	0.35311793	b.47.1.3	4
2SNV	b.47.1.3	151	11.08	9.21	12.67	0.5584526	b.47.1.3	4
1bco_	b.48.1.1	80	5.48	5.6	6.81	0.76579857	b.48.1.1	5
1e79a	b.49.1.1	76	5.07	6.36	8.24	0.78509024	b.49.1.1	5
1e79d	b.49.1.1	73	4.03	5.56	8.22	0.86351606	b.49.1.1	5
1hoe_	b.5.1.1	74	5.83	8.79	10.93	0.80554499	g.18.1.1	0
3TLHA	b.50.1.1	99	5.97	10.53	11.29	0.64698169	c.78.2.1	0
1FB7A	b.50.1.1	99	6.72	11.01	11.76	0.708933	c.78.2.1	0
1bw3_	b.52.1.2	125	10.18	10.57	14.07	0.12971312	c.47.2.1	0
1cr5a	b.52.2.3	82	7.55	8.4	9.55	0.47465406	b.114.1.1	1
d1qcsa1	b.52.2.3	86	7.26	7.9	9.41	0.70316507	b.52.2.3	4

1dfup	b.53.1.1	94	4.85	5.27	7.89	0.88271955	b.53.1.1	5
1cl3A	b.54.1.1	138	11.29	11.66	15.53	0.19150957	a.1.1.2	0
1IO4D	b.54.1.1	141	11.64	9.76	14.85	0.15837063	d.218.1.1	0
1btn_	b.55.1.1	106	7.77	7.64	9.86	0.75960009	b.55.1.1	5
d1ytfd2	b.56.1.1	65	4.83	9.02	10.24	0.57863937	g.30.1.1	0
1ytfc	b.56.1.1	46	2.09	5.01	9.13	0.91958481	b.34.1.1	1
1pkla	b.58.1.1	99	7.15	10.15	13.07	0.66978348	b.58.1.1	5
1e0ta	b.58.1.1	98	6.51	9.14	13.09	0.57074246	b.1.1.4	1
d1ik9a1	b.59.1.1	117	9.71	18.08	20.02	0.79658648	d.13.1.1	0
1b3iA	b.6.1.1	97	6.47	7.72	9.46	0.86956567	b.61.4.1	1
1BXUA	b.6.1.1	91	6.72	7.81	8.14	0.94285781	b.48.1.1	1
1kzqa	b.6.2.1	124	9.47	9.29	13.17	0.37651614	b.61.4.1	1
1kzqa	b.6.2.1	129	10.26	9.3	11.21	0.21240044	d.110.3.1	0
1EIOA	b.60.1.2	127	5.87	3.72	5.32	0.99930356	b.60.1.2	4
2IZB	b.61.1.1	122	9.2	8.67	11.21	0.80728057	b.61.1.1	4
1PTSA	b.61.1.1	121	9.44	8.43	9.35	0.55128117	c.51.2.1	0
1jiwi	b.61.2.1	105	7.97	9.53	11.88	0.68478034	a.60.9.1	0
1smpi	b.61.2.1	100	7.72	9.89	11.97	0.51990589	b.61.2.1	4
1ei5a	b.61.3.1	82	3.68	8.86	11.31	0.60180756	b.61.4.1	2
1ei5a	b.61.3.1	103	8.95	9.43	12.3	0.28671967	d.95.2.2	0
1jmxa	b.61.4.1	119	10.03	8.09	13.84	0.58123298	b.61.4.1	4
1jjua	b.61.4.1	108	5.68	6.34	7.42	0.97660041	b.61.4.1	4
1k3ba	b.61.5.1	119	9.96	10.76	11.91	0.50548425	b.1.1.1	1
2nul_	b.62.1.1	163	12.15	11.72	15	0.09475947	b.62.1.1	4
2cpl_	b.62.1.1	164	12.32	12.33	13.93	0.24408825	b.62.1.1	5
1qtuA	b.63.1.1	115	9	10.44	12.84	0.32736769	b.63.1.1	4
1a1x_	b.63.1.1	106	8.53	9.44	12.7	0.45925141	b.40.4.4	1
1gp0a	b.64.1.1	133	10.57	11.14	12.86	0.26444678	b.60.1.2	1
1E6FA	b.64.1.1	143	11.06	15.59	18.45	0.33607623	b.60.1.1	1
1f3ua	b.65.1.1	118	9.72	12.05	16.03	0.29839631	b.65.1.1	5
1rlw_	b.7.1.1	126	10.55	9.11	12.85	0.31005496	d.129.3.1	0
1BYNA	b.7.1.2	128	10.55	7.7	9.6	0.41243537	b.7.1.2	4
d1l4ia2	b.7.2.1	85	4.4	15.72	18.75	0.77303585	b.1.1.4	1
1qpxa	b.7.2.1	91	6.41	7.4	12.17	0.60360747	b.1.2.1	1

1who_	b.7.3.1	94	4.94	7	9.78	0.70341506	d.93.1.1	0
d1dcea2	b.7.4.1	110	9.39	8.49	11.45	0.53636425	c.51.1.1	0
1lwha	b.71.1.1	50	2.09	4.88	6.88	0.83853889	b.34.5.4	1
1kwga	b.71.1.1	54	2.84	4.98	5.99	0.92243426	b.71.1.1	5
1e0lA	b.72.1.1	37	0.75	3.56	4.72	0.69427158	b.72.1.1	4
1aiw_	b.72.2.1	62	5.98	7.76	10.12	0.43593518	d.58.7.1	0
1ed7a	b.72.2.1	45	3.31	5.16	8.52	0.31249065	b.84.2.2	1
1dkga	b.73.1.1	59	3.8	6.45	9.99	0.75998975	d.71.1.1	0
1C3KA	b.77.3.1	147	11.05	12.56	15.89	0.21357593	b.77.3.1	4
1NPLA	b.78.1.1	109	6.06	9.22	13.73	0.68924649	b.60.1.2	1
1LB4A	b.8.1.1	159	12.92	13.16	15.53	0.24156126	b.8.1.1	4
1ee6a	b.80.1.1	197	13.29	15.21	18.36	0.03775024	b.29.1.6	1
1EZGA	b.80.2.1	84	8.2	10.32	11.13	0.42026388	a.58.1.1	0
1hf2a	b.80.3.1	107	7.3	9.05	11.85	0.42956208	b.71.1.1	1
1EWWA	b.81.2.1	90	8.03	10	11.62	0.56060627	d.93.1.1	0
1EP0A	b.82.1.1	185	13.63	13.4	17.23	0.04843318	d.14.1.3	0
1ft9a	b.82.3.1	132	7.44	9.72	13.75	0.81838643	d.224.1.1	0
1o7fa	b.82.3.2	124	8.09	8.52	12.54	0.51897125	c.20.1.1	0
2ARA	b.82.4.1	149	10.51	9.97	10.51	0.31798394	b.82.4.1	4
1wapa	b.82.5.1	68	4.72	6.89	8.58	0.74574957	b.82.5.1	5
1ig0a	b.82.6.1	96	8.08	10.38	13.31	0.35603701	b.93.1.1	1
1ig3a	b.82.6.1	85	7.3	8.46	8.99	0.75988074	b.71.1.1	1
1qiua	b.83.1.1	77	6.13	11.76	12.8	0.52083209	d.58.1.5	0
1kkea	b.83.1.2	143	12.07	12.45	14.98	0.30616645	d.58.39.1	0
1ghk_	b.84.1.1	79	6.4	7.54	9.75	0.80819752	b.84.1.1	5
1dd2a	b.84.1.1	77	3.07	4.07	6.41	0.99745297	b.84.1.1	5
1kjqa	b.84.2.1	74	3.98	4.35	10.61	0.83843254	b.84.2.1	5
1e2wa	b.84.2.2	64	2.62	5.81	7.66	0.97673799	b.84.2.2	5
1f3g_	b.84.3.1	150	9.61	12.3	16.13	0.15350013	b.60.1.1	1
1GZI	b.85.1.1	65	3.64	6.53	8.37	0.85992555	a.164.1.1	0
1OPS	b.85.1.1	64	4.75	6.61	8.24	0.65796389	a.4.1.1	0
1c5ea	b.85.2.1	95	6.81	10.08	12.89	0.68484444	b.61.4.1	1
4ubpb	b.85.3.1	122	9.41	13.31	17.34	0.17742487	d.105.1.1	0
1DUTA	b.85.4.1	133	10.82	13.78	16.89	0.19054995	b.60.1.2	1

1DUN	b.85.4.1	134	11.43	12.97	16.32	0.16775509	b.29.1.3	1
1g8la	b.85.6.1	83	5.98	8.12	9.37	0.57756458	d.68.4.1	0
1n3ja	b.85.7.2	119	7.95	10.56	12.78	0.65239472	b.60.1.2	1
1at0_	b.86.1.1	145	9.46	9.43	13.55	0.34924279	b.61.4.1	1
1jhcA	b.87.1.1	130	10.4	10.25	11.19	0.42554368	b.87.1.1	4
1fwqA	b.88.1.1	115	10.24	10.87	13.88	0.14869689	b.55.1.2	1
3ezma	b.89.1.1	101	8.87	9.39	10.62	0.36899843	a.74.1.1	0
1LOMA	b.89.1.1	101	8.87	11.96	14.09	0.39161313	a.60.13.1	0
1L5CA	b.9.1.1	92	8.41	8.06	12.18	0.15426407	c.47.1.3	0
1lkta	b.90.1.1	104	7.9	9.11	14.74	0.76720903	b.61.2.1	1
1qqha	b.91.1.1	144	9.14	10.98	14.93	0.40970211	b.91.1.1	5
1m7ja	b.92.1.6	61	4.57	7.21	10.68	0.72861078	d.42.1.2	0
1m7ja	b.92.1.6	55	3.98	6.17	10.43	0.74798245	b.34.5.4	1
1aqt_	b.93.1.1	85	5.16	5.29	9.91	0.91346745	b.93.1.1	5
1e79h	b.93.1.1	86	4.48	9.08	10.34	0.78707154	b.60.1.2	1
1JYTA	b.94.1.1	163	11.51	12.98	15.88	0.34194128	d.58.7.3	0
1JOBA	b.94.1.1	162	11.33	13.82	17.81	0.26004464	a.26.1.1	0
1KD6A	b.97.1.1	179	13.18	13.48	15.33	0.09057582	c.36.1.1	0
1dcea	c.10.2.2	124	9.5	9.59	10.7	0.83028611	c.10.2.2	5
d1hf2a2	c.102.1.1	99	7.47	7.14	9.84	0.91807639	c.102.1.1	4
d1ihna_	c.103.1.1	113	7.97	7.8	12.11	0.83462751	c.51.1.1	1
1jkea	c.110.1.1	145	10.6	10.38	12.95	0.47613729	d.155.1.2	0
1l1sA	c.114.1.1	113	4.86	9.43	10.88	0.99958508	c.114.1.1	4
d1jj2k_	c.12.1.1	150	10.78	23.92	28.12	0.46420937	c.61.1.2	1
1jj2n	c.12.1.1	115	7.7	8.52	11.47	0.81607722	c.12.1.1	5
1auz_	c.13.2.1	116	6.53	4.84	6.63	0.99165921	c.13.2.1	5
1cdza	c.15.1.1	96	7.26	7.64	9.1	0.81076869	c.102.1.1	1
1in1a	c.15.1.2	88	6.76	6.57	10.56	0.50573898	c.15.1.2	5
1muga	c.18.1.2	165	11.78	10.92	13.13	0.75100057	c.18.1.2	5
d1j5pa1	c.2.1.3	110	6.67	5.68	5.68	0.99478636	c.2.1.8	3
1J3AA	c.21.1.1	142	9.93	12.56	14.56	0.43871528	d.58.39.1	0
1tmy_	c.23.1.1	118	3.85	2.39	3	0.99985601	c.23.1.1	5
1eiwa	c.23.3.1	111	7.42	6.7	9.51	0.73428348	c.51.1.1	1
5NLL	c.23.5.1	138	6.41	5.18	6.03	0.99945198	c.23.5.1	4

6NUL	c.23.5.1	138	6.27	4.75	4.9	0.99982624	c.23.5.1	4
1B1A	c.23.6.1	137	6.84	5.25	5.72	0.99902152	c.23.6.1	4
1BE1	c.23.6.1	137	6.55	5.87	6.69	0.99732008	c.23.6.1	4
1CUE	c.23.9.1	197	12.73	10.55	12.53	0.95306786	c.23.9.1	4
1a9xa	c.24.1.1	138	8.43	8.54	9.1	0.93972787	c.23.1.2	1
2pia_	c.25.1.2	120	4.13	3.8	5.07	0.99902304	c.25.1.2	5
1ej2a	c.26.1.3	167	9.85	8.46	10.22	0.8221856	c.37.1.1	1
1dlja	c.26.3.1	108	6.35	6.42	9.48	0.75448731	d.68.1.1	0
1lvl_	c.3.1.5	115	5.63	4.78	5.14	0.99744944	c.3.1.5	5
1b6ra	c.30.1.1	78	4.28	6.68	8.79	0.7925022	c.47.1.5	1
1iow_	c.30.1.2	96	5.23	5.98	7.66	0.9772399	c.30.1.2	5
1d4oa	c.31.1.4	177	11.94	10.67	15.09	0.92363428	c.31.1.4	5
1IM5A	c.33.1.2	180	10.62	8.66	10.46	0.99581453	c.33.1.2	4
1im5a	c.33.1.2	179	10.3	9.14	10.24	0.93516805	c.33.1.2	5
1JR6A	c.37.1.14	142	10.99	18.39	21.67	0.89969403	c.1.2.2	1
1HUQA	c.37.1.8	164	9.56	9.41	9.87	0.98159439	c.37.1.8	4
1BLE	c.38.1.1	163	10.02	11.75	11.82	0.9677452	c.38.1.1	4
1l5aa	c.43.1.2	174	12.38	12.33	17.95	0.42156086	c.2.1.1	1
1jf8a	c.44.1.1	130	9.54	9.28	13.59	0.83952122	c.26.1.3	1
1ljlA	c.44.1.1	130	9.51	10.75	15.01	0.78023362	c.121.1.1	1
1IIBA	c.44.2.1	106	4.87	5.17	6.2	0.9913757	c.44.2.1	4
1h9cA	c.44.2.1	106	4.85	4.69	6.16	0.99229005	c.44.2.1	4
1mkp_	c.45.1.1	144	8.6	6.22	8.37	0.91764363	c.45.1.1	5
1ikzA	c.45.1.1	142	8.09	6.95	11.74	0.96180785	c.45.1.1	4
1GN0A	c.46.1.3	108	6.03	5.98	11.29	0.92340667	c.46.1.3	4
1iloa	c.47.1.1	77	3.02	2.16	3.5	0.98422139	c.47.1.1	5
1H75A	c.47.1.1	81	2.31	4.95	7.39	0.99083107	c.47.1.1	4
1qmha	c.47.2.1	95	5.19	6.82	11.89	0.60848627	c.47.2.1	5
d1itza3	c.48.1.1	136	7.68	8.64	12	0.86221474	c.59.1.1	1
1e0ta	c.49.1.1	117	4.43	3.7	3.72	0.99978752	c.49.1.1	5
1a3wa	c.49.1.1	134	7.4	4.76	7.65	0.9758172	c.49.1.1	4
d1j6ua1	c.5.1.1	89	5.45	2.33	4.35	0.99262172	c.5.1.1	4
2uaga	c.5.1.1	93	2.92	2.42	3.04	0.99878101	c.5.1.1	5
1qe0a	c.51.1.1	95	2.67	2.71	2.82	0.99880703	c.51.1.1	5

1h4vb	c.51.1.1	96	3.13	3.43	5.05	0.99111315	c.51.1.1	5
1crza	c.51.2.1	134	9.65	8.94	9.33	0.94968143	c.51.2.1	5
1nbwb	c.51.3.2	113	6.82	5.62	7.25	0.99603772	c.51.3.2	5
1HH1A	c.52.1.18	143	8.49	8.6	8.84	0.95659433	c.52.1.18	4
1a79a	c.52.2.1	97	6.72	6.6	6.6	0.72750297	c.52.2.1	5
1GHTA	c.53.1.1	105	4.88	4.74	5.59	0.97496404	c.53.1.1	4
1pdo_	c.54.1.1	129	5.89	4.86	6.25	0.99873347	c.54.1.1	5
1ilya	c.55.4.1	90	3.71	3.55	4.62	0.99593917	c.55.4.1	5
1o13A	c.55.5.1	136	9.07	16.64	19.22	0.94872448	c.55.5.1	4
1eo1a	c.55.5.1	124	6.92	7.25	7.25	0.99910619	c.55.5.1	5
1sfe_	c.55.7.1	81	3.02	5.84	8.03	0.6644606	c.55.7.1	5
d1qnta2	c.55.7.1	86	7.62	16.81	18.28	0.69146511	a.57.1.1	0
1jlja	c.57.1.1	169	10.95	9.22	11.04	0.87152675	c.57.1.1	4
1DI6A	c.57.1.1	195	13.5	15.75	18.51	0.50640372	c.65.1.1	1
1lu9a	c.58.1.4	96	5.51	5.78	11.33	0.82026051	c.95.1.1	1
d1npda2	c.58.1.5	106	5.85	5.33	8.74	0.95808431	c.58.1.4	3
1l1qa	c.61.1.1	181	11.74	11.85	14.04	0.92490932	c.61.1.1	5
1l1rA	c.61.1.1	179	11.79	10.86	13.26	0.93557397	c.61.1.1	4
1mjna	c.62.1.1	179	11.13	9.41	13.99	0.96142222	c.62.1.1	4
1ej0a	c.66.1.2	180	10.78	8.57	10.53	0.98728359	c.66.1.2	5
1ispa	c.69.1.18	179	10.74	9.52	9.76	0.98967936	c.69.1.18	5
1ra9_	c.71.1.1	159	11.39	12.29	13.16	0.63194315	c.25.1.3	1
1duvg	c.78.1.1	150	8.9	7.15	9.51	0.98561574	c.78.1.1	5
1ml4a	c.78.1.1	150	8.85	7.07	8.04	0.94147415	c.78.1.1	5
1b74a	c.78.2.1	105	4.96	4.08	6.99	0.99547399	c.78.2.1	5
1jfla	c.78.2.1	113	5.89	5.82	7.67	0.97874902	c.78.2.1	5
1kbla	c.8.1.1	133	8.91	8.92	13.56	0.73820324	c.8.1.1	5
1a9xb	c.8.3.1	151	10.09	12.51	15.36	0.41847523	c.37.1.17	1
1SRVA	c.8.5.1	145	9.1	7.86	10.47	0.92649552	c.23.1.1	1
1ass_	c.8.5.2	152	9.06	10.23	16.71	0.96757746	c.8.5.2	5
1k2yx	c.84.1.1	109	6.19	5.54	6.27	0.97162699	c.84.1.1	5
1k2yx	c.84.1.1	104	4.54	5.13	6.13	0.93104977	c.84.1.1	5
1ay7b	c.9.1.1	89	4.07	4.42	7.75	0.71920688	c.9.1.1	5
1jj2x	c.9.2.1	142	11.56	12.86	18.03	0.72802189	c.115.1.1	1

1GVCA	c.94.1.2	157	11.9	10.52	10.52	0.54274068	c.94.1.2	4
1gv8a	c.94.1.2	159	11.26	9.66	12.25	0.44675025	c.94.1.2	5
d1e8ca1	c.98.1.1	101	7.53	7.3	10.71	0.93148955	c.98.1.1	4
d1gg4a3	c.98.1.1	98	7.25	7.11	10.47	0.92401193	c.98.1.1	4
1knxa	c.98.2.1	132	8.49	7.7	14.18	0.96968628	c.98.2.1	5
1ko7a	c.98.2.1	129	8.29	8.14	11.68	0.96415818	c.98.2.1	4
1mgrA	d.1.1.2	97	8.65	9.24	11.29	0.5631412	d.1.1.2	4
1MGRA	d.1.1.2	99	8.62	9.52	12.93	0.55642793	d.1.1.2	4
2GCC	d.10.1.2	70	3.34	5.81	7.41	0.85772073	d.10.1.2	4
1KJKA	d.10.1.4	64	3.22	7.12	9.81	0.55256619	d.9.2.1	1
1CQUA	d.100.1.1	56	4.19	5.02	8.78	0.58780945	d.10.1.4	1
1qhka	d.100.1.2	47	1.02	3.28	4.09	0.67941385	d.100.1.2	5
d1e3ha6	d.101.1.1	96	6.88	7.94	14.3	0.66406153	d.222.1.1	1
d1e3ha5	d.101.1.1	111	6.81	8.83	10.76	0.86740234	d.101.1.1	4
1AVV	d.102.1.1	151	11.71	44.6	46.74	0.53416265	a.74.1.1	0
2nef_	d.102.1.1	136	10.62	10.97	12.48	0.43683263	a.80.1.1	0
1kyfa	d.105.1.1	114	8.42	10.13	13.66	0.7624927	d.105.1.1	5
1e42a	d.105.1.1	113	7.94	7.43	11.41	0.91703499	d.105.1.1	5
1qndA	d.106.1.1	123	5.96	7.44	14.47	0.81574074	d.14.1.3	1
1ikta	d.106.1.1	115	5.96	6.53	13.99	0.90287441	d.106.1.1	5
1Q2CA	d.108.1.1	162	10.16	9.74	11.18	0.85145567	d.108.1.1	4
1qsta	d.108.1.1	160	10.15	8.87	8.87	0.93557625	d.108.1.1	5
1kcqa	d.109.1.1	103	6.28	6.58	11.23	0.93756494	d.109.1.1	5
1svr_	d.109.1.1	94	5.33	4.22	5.03	0.98471644	d.109.1.1	4
1qmea	d.11.1.1	61	4.8	5.47	8.73	0.57761746	d.11.1.1	5
1qmea	d.11.1.1	58	2.85	4.21	9.97	0.61212955	d.11.1.1	5
2PRF	d.110.1.1	125	7.36	5.86	5.86	0.96686524	d.110.1.1	4
1acf_	d.110.1.1	125	7.63	6.05	7.43	0.95879276	d.110.1.1	5
1F9IA	d.110.3.1	125	7.83	8.29	9.89	0.48764012	d.110.3.1	4
1bywa	d.110.3.3	110	5.97	5.33	5.4	0.99288824	d.110.3.3	5
1h8ma	d.110.4.1	140	9.39	8.01	8.71	0.98823999	d.110.4.1	5
d1l3la2	d.110.5.1	168	10.59	9.8	12.72	0.83597811	c.57.1.1	0
1cfe_	d.111.1.1	135	9.75	10.24	11.65	0.62621913	a.1.1.2	0
1a3aa	d.112.1.1	145	7.7	7.6	9.66	0.81664948	d.112.1.1	5

1a6ja	d.112.1.1	150	8.42	7.27	8.62	0.82231297	d.112.1.1	5
1mut_	d.113.1.1	129	8.42	8.78	12.13	0.8195335	d.113.1.1	5
1dbuA	d.116.1.1	158	10.82	11.08	14.89	0.59758735	c.37.1.1	0
1lba_	d.118.1.1	146	10.38	9.37	10.53	0.89841675	c.16.1.1	0
1jj2r	d.12.1.1	81	3.27	3.35	4.9	0.98661791	d.12.1.1	5
1EHBA	d.120.1.1	82	4.72	4.76	10.31	0.44085122	a.35.1.1	0
1M2MA	d.120.1.1	82	4.3	5.52	9.33	0.95224613	d.120.1.1	4
1i5dA	d.122.1.3	190	11.22	11.21	14.2	0.84843977	d.122.1.3	4
1ID0A	d.122.1.3	152	8.42	13.29	17.6	0.63723165	d.185.1.1	1
1ucaa	d.124.1.1	190	13.48	12.8	16.54	0.62907644	d.124.1.1	5
1ytba	d.129.1.1	85	5.12	5.55	8.58	0.90913641	d.129.1.1	5
1aisa	d.129.1.1	92	6.77	9.12	12.23	0.73419119	d.129.1.1	5
3pmga	d.129.2.1	141	9.73	11.3	12.26	0.66619431	d.129.2.1	5
1k2yx	d.129.2.1	96	4.34	6.28	9.33	0.57701543	d.129.2.1	5
1QMRA	d.129.3.1	159	8.5	8.2	8.56	0.97965648	d.129.3.1	4
1ICXA	d.129.3.1	155	8.29	6.47	10.27	0.93735531	d.129.3.1	4
1F7WA	d.129.4.1	144	10.62	10.51	12.25	0.71997062	a.25.1.1	0
3RHN	d.13.1.1	115	7.73	7.07	7.62	0.91344665	d.13.1.2	3
d1mxa_1	d.130.1.1	102	5.72	10.14	13.75	0.96791074	d.130.1.1	4
1b77a	d.131.1.2	110	7.6	7.45	12.63	0.93698242	d.131.1.2	5
d1ge8a1	d.131.1.2	116	6.9	7.29	10.62	0.88146682	d.131.1.2	4
1DUJA	d.135.1.1	187	11.67	11.91	14.62	0.81058929	d.135.1.1	4
1byra	d.136.1.1	152	9.6	9.34	12.02	0.99448123	d.136.1.1	5
1g10a	d.137.1.1	102	4.65	7.26	13.12	0.89397406	d.137.1.1	5
1hqi_	d.137.1.1	90	5.66	6.37	7.73	0.98933064	d.137.1.1	4
1a6f_	d.14.1.2	113	5.99	6.28	10.12	0.82550306	d.52.4.1	1
1D6TA	d.14.1.2	117	6.56	7.82	12.19	0.66881971	d.58.20.1	1
1seia	d.140.1.1	130	8.47	8.28	12.05	0.91928956	d.140.1.1	5
d1i6ua_	d.140.1.1	129	9.29	7.96	10.94	0.95786711	d.140.1.1	4
1jj2e	d.141.1.1	79	2.19	1.61	4.18	0.99503776	d.141.1.1	5
1rl6a	d.141.1.1	75	1.91	1.68	2.68	0.98933762	d.141.1.1	5
1hska	d.146.1.1	109	6.67	10.02	13.8	0.84851298	d.146.1.1	5
1uxy_	d.146.1.1	142	9.87	10.02	12.99	0.50339423	d.146.1.1	5
1FXTB	d.15.1.1	76	2.59	2.52	5.4	0.98725459	d.15.1.1	4

1qf6a	d.15.10.1	61	3.13	4.09	4.5	0.73508382	d.15.3.2	2
d1ni3a2	d.15.10.2	82	4.68	6.75	11.62	0.43888754	a.4.5.17	0
1ip9a	d.15.2.2	85	6.91	8.11	11.62	0.57783784	d.15.2.2	5
1f0za	d.15.3.2	66	3.14	5.99	8.89	0.84670557	d.68.3.1	1
1jsbA	d.15.3.2	73	3.97	8.79	11.74	0.81407124	d.74.2.1	1
1DOX	d.15.4.1	96	7.08	7.12	10.45	0.51246756	d.15.4.2	3
1AWD	d.15.4.1	94	6.67	7.34	9.05	0.78633918	d.15.4.1	4
2sak_	d.15.5.1	121	8.84	11.45	14.89	0.67901135	d.15.5.1	5
1c76A	d.15.5.1	121	8.93	12.11	16.37	0.48364875	d.15.5.1	4
1m4va	d.15.6.1	104	8.08	8.17	10.46	0.70441275	d.15.6.1	4
3tss_	d.15.6.1	101	8.49	8.2	10.76	0.76699457	d.15.6.1	5
1GB1	d.15.7.1	56	1.47	2.01	3	0.87324491	d.15.7.1	4
1TIF	d.15.8.1	78	3.67	5.8	7.34	0.93393308	d.15.8.1	4
1f52a	d.15.9.1	100	7.4	7.44	10.85	0.66060273	b.59.1.1	0
1f7la	d.150.1.2	118	5.66	4.59	5.74	0.99931817	d.150.1.2	5
1jjwa	d.153.1.4	173	9.82	9.33	13.1	0.99465383	d.153.1.4	5
1an9a	d.16.1.3	93	7.34	7.93	10.42	0.50573155	c.52.2.1	0
1i8ta	d.16.1.7	69	6.04	7.24	8.45	0.47523228	d.205.1.1	1
1aiha	d.163.1.1	170	10.69	11.49	13.86	0.83409709	a.74.1.1	0
1AE9A	d.163.1.1	179	12.16	15.19	17.03	0.60055151	f.1.4.1	0
1mhda	d.164.1.1	123	9.19	8.02	11.99	0.62470134	d.164.1.1	5
1dtp_	d.166.1.1	190	13.35	14.93	15.88	0.42712076	c.58.1.1	0
1lryA	d.167.1.1	165	12.39	12.36	15.88	0.52015015	a.39.1.8	0
1g2aa	d.167.1.1	164	11.16	12.98	17.07	0.51461495	d.167.1.1	5
1qlaa	d.168.1.1	121	9.13	9.81	15.53	0.47001617	d.109.1.2	1
1chua	d.168.1.1	116	7.16	9.87	12.47	0.56312958	c.59.1.2	0
1tsg_	d.169.1.4	98	7.33	8.18	10.13	0.62656997	b.1.2.1	0
1FUWA	d.17.1.1	91	6.9	8.52	10.14	0.57811952	a.51.1.1	0
1mola	d.17.1.1	94	7.93	8.18	10.52	0.59039981	d.79.1.1	1
1ivwa	d.17.2.1	88	6.79	5.96	8.61	0.64193985	a.80.1.1	0
1ksia	d.17.2.1	93	6.32	5.64	6.23	0.9823249	d.17.2.1	5
1eeja	d.17.3.1	60	3.08	4.29	5.41	0.8606898	d.17.3.1	5
1DMNA	d.17.4.3	131	9.21	15.49	18.2	0.88516228	d.17.4.3	4
8CHO	d.17.4.3	125	8.74	7.13	13.11	0.89913912	d.17.4.3	4

1ugia	d.17.5.1	83	4.68	4.94	6.62	0.87109625	d.17.5.1	5
1BY2	d.170.1.1	119	9.53	13.04	13.24	0.40620498	b.43.4.3	0
1mwpa	d.170.2.1	96	7.09	8.58	12.49	0.49891436	a.60.13.1	0
1pcfa	d.18.1.1	66	3.12	5.88	8.31	0.75173462	d.87.1.1	1
d1l3aa_	d.18.1.2	166	12.28	0	0	0.37076868	d.162.1.1	1
1i50c	d.181.1.1	131	10.7	10.72	13.67	0.32941225	d.32.1.2	1
d1qgwa_	d.184.1.1	76	7.51	11.95	13.38	0.41290225	a.77.1.3	0
1jyoe	d.184.1.2	102	7.2	16.97	19.71	0.9278843	a.39.1.6	0
1j98A	d.185.1.2	157	10.74	11.04	11.31	0.63464706	d.96.1.2	1
1HYWA	d.186.1.1	68	2.26	5.18	6.15	0.7046915	d.10.1.2	1
1gd8a	d.188.1.1	105	5.78	4.94	11.73	0.811635	a.1.1.1	0
1h6ha	d.189.1.1	143	9.91	11.28	16.59	0.86653851	d.110.4.2	1
1kmda	d.189.1.1	117	9.6	7.56	13.49	0.78706846	a.78.1.1	0
1l8jA	d.19.1.1	170	11.92	9.65	11.52	0.92587213	d.19.1.1	4
1G2RA	d.192.1.1	100	7.08	6.3	13.4	0.80959307	d.192.1.1	4
1hufa	d.195.1.1	123	8.64	9.1	12.13	0.5629009	d.228.1.1	1
1k46A	d.195.1.1	124	8.97	9.81	13.92	0.79200406	d.195.1.1	4
1dl5a	d.197.1.1	104	7.8	9.5	13.56	0.79016437	a.7.6.1	0
1l2wi	d.198.1.1	57	3.33	15.93	17.53	0.45899803	a.60.1.2	0
d1k3sa_	d.198.1.1	113	6.42	8.02	9.68	0.62609048	d.198.1.1	4
1k8kd	d.198.2.1	120	7.67	8.27	9.5	0.89780887	d.198.2.1	5
1k8kd	d.198.2.1	164	10.11	13.25	19.1	0.92005471	d.198.2.1	5
1kafa	d.199.1.1	108	6.01	4.96	7.19	0.97951963	d.199.1.1	5
1iiza	d.2.1.2	120	8.65	7.56	12.7	0.51344335	d.2.1.2	5
1GD6A	d.2.1.2	119	8.84	8.86	12.66	0.45033279	d.93.1.1	1
1j74A	d.20.1.1	139	11.09	8.57	13.37	0.47920414	a.79.1.1	0
1m1xb	d.200.1.1	85	5.4	6.42	8.05	0.77652747	d.200.1.1	5
1kvna	d.201.1.1	104	7.74	6.91	12.3	0.73117775	a.29.4.1	0
1k0ra	d.202.1.1	104	3.77	4.74	7.39	0.9314408	d.202.1.1	5
1ji8a	d.203.1.1	111	6.03	5.32	5.38	0.99514061	d.203.1.1	5
1IMUA	d.204.1.1	107	5.33	7.39	8.64	0.8982115	d.50.2.1	1
1jg5a	d.205.1.1	83	5.93	7.09	7.9	0.54529927	a.60.5.1	0
1n91a	d.206.1.1	108	7.95	7.83	11.85	0.77587092	d.58.18.1	1
1j5uA	d.208.1.1	136	10.36	12.55	16.78	0.74580406	a.1.1.3	0

1jw3a	d.208.1.1	140	10.59	7.97	11.52	0.92425345	d.208.1.1	5
1jbia	d.209.1.1	100	8.29	10	12.66	0.55389175	d.131.1.2	1
d1bwza1	d.21.1.1	130	7.87	7.03	9.68	0.90495978	c.23.4.1	0
1gqza	d.21.1.1	144	9.08	9.82	12.55	0.99298398	d.21.1.1	5
1myo_	d.211.1.1	118	6.52	5.85	6.86	0.96426333	d.211.1.1	5
d1lm5a_	d.211.2.1	196	11.77	11.06	12.23	0.98877603	d.211.2.1	4
1lr0A	d.212.1.1	129	9.23	13.89	18.43	0.50980823	a.133.1.2	0
1iqoa	d.214.1.1	88	4.04	6.86	10.59	0.765841	d.58.18.1	1
1l9va	d.216.1.1	143	9.84	8.35	9.73	0.95345163	a.45.1.1	0
1h5pa	d.217.1.1	95	8.1	6.78	9.15	0.81084886	d.217.1.1	5
1j57a	d.221.1.1	143	10.97	9.05	13.21	0.54665316	c.2.1.5	0
1j8bA	d.222.1.1	112	5.28	10.81	12.17	0.84689089	a.2.7.1	0
1mbya	d.223.1.1	75	2.63	8.35	13.17	0.55959122	d.141.1.1	1
d1mzga_	d.224.1.1	144	6.93	8.31	10.59	0.86283637	d.224.1.1	4
1iwga	d.225.1.1	88	4.05	5.85	11.26	0.94602425	d.225.1.1	5
1iwga	d.225.1.1	92	6.25	10.94	15.33	0.46289098	d.1.1.2	1
1mk0a	d.226.1.1	97	6.82	6.85	9.39	0.62213789	a.118.11.1	0
1lrra	d.228.1.1	118	7.28	7.16	13.25	0.66360721	d.58.26.3	1
1hpwa	d.24.1.1	129	9.48	9.55	10.92	0.46347454	d.108.1.1	1
1dzoa	d.24.1.1	120	9.32	8.17	9.42	0.74352902	b.1.1.2	0
1eq3a	d.26.1.1	96	6.1	5.17	7.88	0.88127658	d.26.1.1	4
1FKF	d.26.1.1	107	9.25	8.52	10.42	0.92560827	d.26.1.1	4
3eipa	d.26.2.1	84	5.45	7.96	11.32	0.49167514	a.51.1.1	0
1kfwa	d.26.3.1	61	4.55	6.3	11.16	0.31163841	d.76.1.1	1
1ll7a	d.26.3.1	62	5.21	5.27	8.76	0.6255444	d.26.3.1	5
1XMQP	d.27.1.1	88	6.66	7.7	9.49	0.62784168	d.54.1.1	1
1QKFA	d.28.1.1	92	7.53	8.44	8.59	0.67707185	d.28.1.1	4
1qkfA	d.28.1.1	73	4.83	5.93	8.25	0.702663	c.30.1.1	0
1jj2w	d.29.1.1	82	4.05	2.94	4.22	0.98321087	d.29.1.1	5
1cv8_	d.3.1.1	173	12.48	11.05	14.24	0.41031085	a.25.1.1	0
1b33n	d.30.1.1	67	4.71	5.44	8.14	0.5382231	d.29.1.1	1
1cz4a	d.31.1.1	94	6.42	8.92	10.16	0.421908	d.93.1.1	1
1e32a	d.31.1.1	94	7.54	8.23	11.6	0.69947267	d.31.1.1	5
1bylA	d.32.1.2	122	8.93	7.73	9.22	0.89383489	d.32.1.3	3

1qtoa	d.32.1.2	122	7.67	5.86	7.8	0.97636073	d.32.1.2	5
1fx3a	d.33.1.1	143	9.02	10.41	17.13	0.82966743	a.25.1.1	0
1bm8_	d.34.1.1	99	6.95	4.26	4.26	0.99461631	d.34.1.1	5
1mb1_	d.34.1.1	98	6.72	5.79	11.17	0.79019275	d.34.1.1	4
d1jr1a2	d.37.1.1	43	1.2	84.01	85.37	0.39748868	g.3.7.4	0
d1jr1a3	d.37.1.1	55	3.47	36.23	37.15	0.73528071	d.37.1.1	4
1lo7a	d.38.1.1	140	10.12	10.92	14.45	0.62249299	d.81.1.5	1
1cmia	d.39.1.1	85	3.81	3.05	5.91	0.91709517	d.39.1.1	5
1emvB	d.4.1.1	131	9.37	9.22	11.99	0.87385885	d.4.1.1	4
1e7la	d.4.1.5	103	7.93	10.76	16.64	0.71953315	a.7.7.1	0
1COAI	d.40.1.1	64	2.33	4.16	7.37	0.52858878	d.40.1.1	4
1YPCI	d.40.1.1	64	2.5	4.1	6.77	0.66706771	d.40.1.1	4
1hlra	d.41.1.1	117	9.64	11.1	14.69	0.49481955	b.55.1.1	0
1jrob	d.41.1.1	122	10.25	12.97	16.54	0.48653541	c.47.1.8	0
1qapa	d.41.2.1	122	8.25	10.36	12.94	0.87737235	c.23.1.1	0
1qpoa	d.41.2.1	115	8.15	8.25	12.03	0.75652641	d.41.2.1	5
2tpt_	d.41.3.1	105	7.25	9.37	13.61	0.55893896	d.58.49.1	1
1brwa	d.41.3.1	103	8.53	10.47	12.63	0.57076619	c.23.1.1	0
1EOEA	d.42.1.2	100	6.16	6.27	11.06	0.76205306	d.42.1.2	4
1a68_	d.42.1.2	87	5.76	5.81	12.03	0.80574864	a.64.1.1	0
1TFE	d.43.1.1	145	8.41	9.19	12.94	0.82046137	d.43.1.1	4
1idsa	d.44.1.1	114	7.46	7.53	12.75	0.66367295	d.93.1.1	1
1ix9a	d.44.1.1	115	7.54	6.88	8.79	0.9688607	d.44.1.1	5
1CTF	d.45.1.1	74	2.34	5.79	7.81	0.84054457	d.45.1.1	4
1ekta	d.46.1.1	53	2.85	6.24	8.29	0.46150554	d.46.1.1	5
1mmsa	d.47.1.1	63	2.85	3.69	3.94	0.91684666	d.47.1.1	5
1AA3	d.48.1.1	63	1.5	2.5	2.96	0.92637795	d.48.1.1	4
d1e8oa_	d.49.1.1	74	2.58	3.17	7.54	0.97253773	d.49.1.1	4
d1e8ob_	d.49.1.1	94	5.98	14.66	18.23	0.90780028	d.74.1.1	1
1M58A	d.5.1.1	106	8.93	8.18	12.86	0.55874495	a.4.3.1	0
1onc_	d.5.1.1	104	9.32	9.92	12.3	0.43284863	d.93.1.1	1
1stu_	d.50.1.1	68	1.61	2.52	3.45	0.97346258	d.50.1.1	5
d1pda_2	d.50.2.1	88	3.09	0	0	0.9897342	d.50.2.1	4
1dq3a	d.50.3.1	79	4.75	4.55	6.68	0.85813519	d.50.3.1	5

1DT4A	d.51.1.1	73	4.79	3.05	4.4	0.96129439	d.51.1.1	4
1vig_	d.51.1.1	71	2.83	2.63	2.67	0.98788288	d.51.1.1	5
3proc	d.52.1.1	80	3.87	6.43	10.82	0.68875849	d.52.1.1	5
d3proc2	d.52.1.1	78	5.53	15.61	18.65	0.75083023	a.60.9.1	0
1gpma	d.52.2.1	121	8.94	6.46	8.06	0.88675539	d.52.2.1	5
1k0ra	d.52.3.1	67	2.67	2.72	2.72	0.96874623	d.52.3.1	5
d1e3ha4	d.52.3.1	54	1.52	29.66	31.25	0.68744595	d.51.1.1	1
1ib8a	d.52.4.1	90	4.51	4.49	6.46	0.99328658	d.52.4.1	5
1mkya	d.52.5.1	81	3.29	3.33	3.67	0.99278073	d.52.5.1	5
1iw5a	d.52.6.1	113	7.41	9.65	11.71	0.97053776	d.52.6.1	5
1fjgc	d.53.1.1	101	5.77	5.52	7.28	0.99138524	d.53.1.1	5
d1jpdx2	d.54.1.1	116	6.07	8.31	14.73	0.72682187	d.54.1.1	4
d1fhua2	d.54.1.1	99	6.69	13.09	15.43	0.89394749	d.54.1.1	4
1GIYS	d.55.1.1	113	7.56	10.71	14.53	0.98901909	d.55.1.1	4
1ghha	d.57.1.1	81	3.28	4.03	4.94	0.98230424	d.57.1.1	5
1FCA	d.58.1.1	55	3.57	3.07	3.78	0.94256984	d.58.1.1	4
2fdn_	d.58.1.1	55	3.45	3.06	3.56	0.89864835	d.58.1.1	5
1GXUA	d.58.10.1	91	5.51	12.99	15.21	0.99101883	d.58.3.1	2
2acy_	d.58.10.1	98	5.55	6.67	7.47	0.94209765	d.58.10.1	5
1n0ua	d.58.11.1	79	2.32	3.6	4.93	0.99242772	d.58.11.1	4
1fnma	d.58.11.1	79	2.14	2.36	4.22	0.99211789	d.58.11.1	5
1gh8a	d.58.12.1	89	5.47	5.13	6.51	0.8694734	d.80.1.3	1
1B64	d.58.12.1	91	6.56	7.2	9.7	0.90488888	d.58.7.1	2
1jjcb	d.58.13.1	104	5.29	5.96	10.75	0.77154363	d.58.13.1	5
1loua	d.58.14.1	97	3.79	5.64	5.91	0.96138568	c.30.1.2	0
1qjhA	d.58.14.1	93	4.7	4.81	6.74	0.97846148	c.30.1.2	0
1fjgj	d.58.15.1	98	5.77	7.72	15.82	0.71254344	d.58.18.2	2
1fvqa	d.58.17.1	72	2.01	2.6	3.48	0.99190097	d.58.17.1	4
1psda	d.58.18.1	84	3.96	3.95	7.39	0.99445855	d.58.18.1	5
1tdj_	d.58.18.2	88	3.56	4.05	4.75	0.98397716	d.58.18.2	5
1dqaa	d.58.20.1	117	5.71	6.79	9.15	0.91381681	d.58.20.1	5
1qaxa	d.58.20.1	110	4.63	5.37	8.1	0.9369216	d.58.20.1	5
1EKSA	d.58.21.1	161	10.61	23.04	28.9	0.58547331	d.129.3.1	1
1EKRA	d.58.21.1	161	10.2	22.62	26.1	0.56602349	d.41.5.1	1

1F2HA	d.58.22.1	169	11.49	13.18	16.66	0.82423927	d.58.22.1	4
1mla_	d.58.23.1	70	1.38	3.53	5.93	0.86731053	d.58.23.1	5
1nm2a	d.58.23.1	62	3.37	3.5	10.21	0.73075143	d.58.23.1	5
1ffgb	d.58.24.1	68	4.48	4.46	4.86	0.88399652	d.58.24.1	5
1FWP	d.58.24.1	139	11.41	13.14	14.39	0.38253208	c.51.2.1	0
1kp6a	d.58.25.1	79	7.02	7.33	9.45	0.53255611	d.64.1.1	1
1h72c	d.58.26.1	133	7.04	8.41	11.46	0.68456623	d.58.26.1	5
d1k47a2	d.58.26.4	135	7.01	9.43	10.9	0.81144457	d.58.26.4	4
1regx	d.58.27.1	122	9.15	10.14	12.71	0.82119523	d.58.27.1	5
1PBA	d.58.3.1	81	3.41	6.76	9.57	0.77548968	d.58.18.1	2
1itpa	d.58.3.2	77	6.11	6.29	7.17	0.70848117	d.58.3.2	5
1f9ya	d.58.30.1	158	10.72	9.88	12.06	0.559373	c.37.1.15	0
1hbnb	d.58.31.2	187	13.08	12.94	17.7	0.4352341	a.29.3.2	0
1ftra	d.58.33.1	148	10.19	12.14	13.37	0.61392903	d.108.1.1	1
d1qd1a2	d.58.34.1	146	10.4	13.33	13.33	0.82667495	d.58.34.1	4
1k8wa	d.58.35.2	65	4.07	5.56	10.67	0.44258728	a.112.1.1	0
d1kska1	d.58.35.3	65	4.75	6.21	10.04	0.60369137	d.74.2.1	1
d1aop_1	d.58.36.1	65	3.03	13.61	14.2	0.9518932	d.58.17.1	2
1aop_	d.58.36.1	80	4.1	3.94	11.03	0.89850201	d.58.17.1	2
1eara	d.58.38.1	68	3.39	2.01	2.89	0.9879144	d.58.38.1	5
1gmua	d.58.38.1	68	3.78	2.72	4.53	0.98257977	d.58.38.1	4
1mli_	d.58.4.1	96	5.24	6.12	8.32	0.4606941	c.13.2.1	0
d1o8ba2	d.58.40.1	72	3.31	5.23	6.78	0.95347142	d.58.17.1	2
1ivza	d.58.41.1	132	10.33	11.71	14.35	0.72005292	d.58.41.1	5
1mxma	d.58.43.1	101	3.9	8.45	13.99	0.98942161	d.58.18.3	2
1iwga	d.58.44.1	97	3.22	3.45	4.28	0.99711701	d.58.44.1	5
d1gtda_	d.58.45.1	82	2.44	8.1	10.94	0.72769343	d.58.4.2	2
2pii_	d.58.5.1	112	9.06	9.18	14.92	0.8692665	d.58.5.1	5
1NCL	d.58.6.1	150	10.97	9.97	11.85	0.53766887	c.78.1.1	0
1LEO	d.58.6.1	150	10.73	10.81	14.71	0.60926727	d.58.6.1	4
1hd0A	d.58.7.1	75	3.87	4.15	7.02	0.95356436	d.58.7.1	4
2msta	d.58.7.1	75	3.24	3.82	4.11	0.98501626	d.58.7.1	5
1A7GE	d.58.8.1	82	4.18	4.98	8.89	0.86646974	d.58.8.1	4
1BY9	d.58.8.1	81	4.96	10.89	12.49	0.52900741	d.58.4.2	2

1geha	d.58.9.1	125	9.41	9.52	11.14	0.54931585	d.58.9.1	5
d5ruba2	d.58.9.1	136	10.37	14.91	17.7	0.57023356	c.2.1.5	0
1bxya	d.59.1.1	60	3.74	3.28	4.84	0.82451429	d.59.1.1	5
1e1wA	d.6.1.1	104	8.78	8.15	12.8	0.8062154	d.110.4.1	1
1AG2	d.6.1.1	103	8.68	9.87	12.97	0.55573324	a.79.1.1	0
1jyha	d.60.1.3	155	10.81	12.26	14.6	0.61807947	c.49.1.1	0
1F32A	d.62.1.1	149	10.48	12.52	13.89	0.72737701	a.118.9.2	0
2if1_	d.64.1.1	126	8.43	12.99	16.88	0.6588929	d.64.1.1	5
1D1RA	d.64.1.1	116	7.08	9.32	11.03	0.88658251	d.64.1.1	4
1C05A	d.66.1.2	159	11.23	10.78	14.23	0.54006745	d.110.2.1	1
1JH3A	d.66.1.4	107	6.87	7.94	11.1	0.60093703	a.24.15.1	0
1f7ua	d.67.2.1	134	9.61	9.46	14.12	0.56170589	d.206.1.1	1
1iq0a	d.67.2.1	96	6.3	4.64	6.92	0.79134854	d.67.2.1	5
1GE9A	d.67.3.1	184	11.8	8.67	16.46	0.99808996	d.67.3.1	4
1dd5A	d.67.3.1	184	11.08	9.86	14.64	0.92275323	a.24.1.1	0
2IFEA	d.68.1.1	100	4.48	6.49	7.96	0.99530789	d.68.1.1	4
1tig_	d.68.1.1	88	2	2.86	3.21	0.9979156	d.68.1.1	5
1dcja	d.68.3.1	81	3.29	4.21	5.26	0.97425041	d.68.3.1	5
1JE3A	d.68.3.2	97	4.96	13.26	16.69	0.87934184	d.68.3.1	3
1ln4A	d.68.4.1	98	4.71	4.82	5.85	0.99400686	d.68.4.1	4
1hc7a	d.68.5.1	74	5.48	5.58	6.98	0.82669268	d.68.5.1	4
1h0xa	d.68.6.1	89	3.84	3.18	3.86	0.99614034	d.68.6.1	5
1MSZA	d.68.7.1	86	6.57	7.81	8.39	0.80778742	d.68.3.1	2
1e01a	d.7.1.1	48	1.98	2.24	3.1	0.87055069	d.7.1.1	5
1kpta	d.70.1.1	105	9.89	9.75	11.37	0.4352188	a.151.1.1	0
1ev0a	d.71.1.1	58	3.24	4.62	6.44	0.76321962	d.71.1.1	5
d1dwka2	d.72.1.1	70	3.28	6.91	11.62	0.46377713	d.72.1.1	4
1bwvs	d.73.1.1	138	8.51	9.32	11.97	0.7248458	d.109.1.2	1
1gk8i	d.73.1.1	125	7.68	10.05	11.35	0.62860666	d.73.1.1	5
1dcpa	d.74.1.1	99	4.38	4.41	8.4	0.99522961	d.74.1.1	5
1xxaa	d.74.2.1	71	3.1	3.84	4.45	0.98316295	d.74.2.1	4
1b4ba	d.74.2.1	71	3.03	2.53	7.99	0.98698311	d.74.2.1	5
1i50k	d.74.3.2	114	4.75	8.78	12.8	0.89878877	d.74.3.2	5
1a79a	d.75.1.1	74	4.66	4.06	5.84	0.70772231	d.75.1.1	5

d1e3ma4	d.75.2.1	115	7.08	7.24	7.58	0.80513665	d.75.2.1	4
d1ewqa4	d.75.2.1	120	7.73	7.38	10.68	0.70655949	c.37.1.22	0
1gyfa	d.76.1.1	62	3.47	5.25	7.07	0.4418585	d.37.1.1	1
1EIKA	d.78.1.1	77	5.17	5.6	10.96	0.54092667	a.60.2.1	0
1hmjA	d.78.1.1	68	2.39	4.72	6.34	0.73451956	d.78.1.1	4
1fnkA	d.79.1.2	127	9	9.98	13.8	0.5932387	d.79.1.2	4
1fnjA	d.79.1.2	127	8.93	11.7	12.48	0.66708289	d.79.1.2	4
1fsz_	d.79.2.1	125	7.5	7.45	12.18	0.95863623	d.79.2.1	5
1ck9a	d.79.3.1	104	6.4	6.51	9.54	0.92564232	d.79.3.1	4
1U3LA	d.79.5.1	159	10.88	11.88	14.47	0.80934766	d.79.5.1	4
4ubpa	d.8.1.1	100	4.25	5.14	8.14	0.98850272	d.8.1.1	5
1fim_	d.80.1.3	114	4.85	8.39	14.14	0.99796056	d.80.1.3	4
1dih_	d.81.1.3	110	7.98	7.13	13.4	0.61781499	a.74.1.2	0
1j5pa	d.81.1.3	103	6.27	6.74	11.24	0.91399665	d.81.1.3	5
1oaca	d.82.1.1	86	2.97	3.51	6.19	0.99442835	d.82.1.1	5
1ly7A	d.82.2.1	121	8.34	6.86	7.76	0.94350974	d.82.2.1	4
1ew4a	d.82.2.1	106	4.84	5.25	8.49	0.92775746	d.82.2.1	5
3ssi_	d.84.1.1	108	8.58	8.34	12.84	0.4545937	d.17.2.1	1
1MSC	d.85.1.1	129	9.22	9.31	14.22	0.83196528	d.85.1.1	4
1d7ya	d.87.1.1	97	6.42	6.44	8.47	0.80172011	d.87.1.1	5
1fcda	d.87.1.1	74	4.18	7.06	8.86	0.63880572	d.52.7.1	1
1fo4a	d.87.2.1	117	7.65	7.67	10.81	0.91366976	a.41.1.1	0
1n62c	d.87.2.1	109	5.44	6.3	8.47	0.78871931	a.24.13.1	0
1mnma	d.88.1.1	85	5.25	8.39	12.92	0.64566664	g.10.1.2	0
1egwa	d.88.1.1	71	3.48	8.79	10.25	0.64879622	d.88.1.1	5
1tbd_	d.89.1.1	134	9.21	9.56	12.08	0.75909466	f.36.1.1	0
1l2ma	d.89.1.4	118	8.01	10.68	13.58	0.68807768	d.89.1.4	5
2HCC	d.9.1.1	66	3.69	6.36	9.74	0.5883386	d.58.17.1	1
1SDF	d.9.1.1	67	3.45	6.78	11.73	0.74393034	d.40.1.1	1
1B4O	d.9.2.1	62	2.97	3.69	4.59	0.95341114	d.9.2.1	4
1SSO	d.9.2.1	62	2.77	3.43	7.53	0.92377291	d.9.2.1	4
1nox_	d.90.1.1	200	13.01	11.52	16	0.77652897	d.90.1.1	5
1dt9a	d.91.1.1	138	7.66	7.99	12.07	0.99082363	d.91.1.1	5
1c7ka	d.92.1.1	132	10.48	9.83	14	0.51459959	c.17.1.1	0

1qba_	d.92.2.1	137	10.36	12.2	14.45	0.69424414	d.92.2.1	5
d1k9da2	d.92.2.2	138	9.46	9.73	11.86	0.73213651	d.92.2.2	4
1opd_	d.94.1.1	85	3.22	3.51	10.17	0.9902566	d.94.1.1	5
1POH	d.94.1.1	85	3.16	4.26	10.07	0.68084732	d.68.3.1	1
d1j5ya2	d.94.2.1	107	3.97	5.78	9.67	0.7612036	c.2.1.3	0
1IBA	d.95.1.1	101	5.84	8.79	12.56	0.51324573	d.68.3.1	1
1AF5	d.95.2.1	126	8.07	10.66	13.5	0.7443009	d.95.2.1	4
1dhn_	d.96.1.3	121	7.76	8.8	10	0.87530692	d.96.1.3	5
1puc_	d.97.1.1	101	8.61	9	11.63	0.53273415	a.4.5.10	0
1div_	d.99.1.1	94	5.45	4.43	10.09	0.89293858	d.99.1.1	5
1i3ja	e.30.1.1	96	7.74	15.91	19.06	0.36148071	d.10.1.3	0
d1knza_	e.34.1.1	157	11.01	11.31	14.58	0.70796289	a.90.1.1	0
d1kyqa2	e.37.1.1	123	6.87	8.54	11.19	0.98338399	e.37.1.1	4
